# The Repertoire and Features of Human Platelet microRNAs

**DOI:** 10.1371/journal.pone.0050746

**Published:** 2012-12-04

**Authors:** Hélène Plé, Patricia Landry, Ashley Benham, Cristian Coarfa, Preethi H. Gunaratne, Patrick Provost

**Affiliations:** 1 Centre hospitalier universitaire de Québec Research Center/Centre hospitalier de l'Université Laval, Quebec, Quebec, Canada, and Faculty of Medicine, Université Laval, Quebec, Quebec, Canada; 2 Department of Biology and Biochemistry, University of Houston, Houston, Texas, United States of America; 3 Department of Pathology, Baylor College of Medicine, Houston, Texas, United States of America; 4 Human Genome Sequencing Center, Baylor College of Medicine, Houston, Texas, United States of America; University of Leuven, Belgium

## Abstract

Playing a central role in the maintenance of hemostasis as well as in thrombotic disorders, platelets contain a relatively diverse messenger RNA (mRNA) transcriptome as well as functional mRNA-regulatory microRNAs, suggesting that platelet mRNAs may be regulated by microRNAs. Here, we elucidated the complete repertoire and features of human platelet microRNAs by high-throughput sequencing. More than 492 different mature microRNAs were detected in human platelets, whereas the list of known human microRNAs was expanded further by the discovery of 40 novel microRNA sequences. As in nucleated cells, platelet microRNAs bear signs of post-transcriptional modifications, mainly terminal adenylation and uridylation. In vitro enzymatic assays demonstrated the ability of human platelets to uridylate microRNAs, which correlated with the presence of the uridyltransferase enzyme TUT4. We also detected numerous microRNA isoforms (isomiRs) resulting from imprecise Drosha and/or Dicer processing, in some cases more frequently than the reference microRNA sequence, including 5′ shifted isomiRs with redirected mRNA targeting abilities. This study unveils the existence of a relatively diverse and complex microRNA repertoire in human platelets, and represents a mandatory step towards elucidating the intraplatelet and extraplatelet role, function and importance of platelet microRNAs.

## Introduction

Platelets play a central role in the maintenance of hemostasis as well as in thrombotic disorders. Released into the circulation as cytoplasmic fragments by bone marrow megakaryocytes, circulating platelets retain a small amount of poly(A)+ RNA from their megakaryocytic precursors [1]. Initial microarray-based studies revealed that up to ∼32% of all human genes are present in platelets at the messenger RNA (mRNA) level [2], [3], [4], an observation confirmed and expanded recently in human and murine platelets by Rowley et al. [5]. Human platelets also possess the essential components of the mRNA translational machinery [6] and respond to physiological stimuli using biosynthetic processes that are regulated at the protein translational level [7], [8].

Recently, we reported that human blood platelets harbor a functional microRNA pathway as well as a relatively diverse array of microRNAs [9], an observation corroborated by independent groups [10], [11], [12]. Forming a class of small, 19- to 24-nucleotide (nt) non-coding RNA species and known as master regulators of mRNA translation [13], microRNAs derive from the sequential processing of longer RNA precursors by the ribonucleases Drosha and Dicer, as reviewed recently [14], [15], [16]. One or both strands of microRNA duplexes are subsequently loaded into an effector ribonucleoprotein (microRNP) complex containing an Argonaute (Ago) protein. Exerting effects that are mainly repressive, microRNAs act in concert through recognition of specific binding sites of imperfect complementarity, usually located in the 3′ untranslated region (UTR), by base pairing of their “seed” region (nt 2 to 8 in the 5′→3′ orientation). Predicted to regulate ∼60% of the genes in human, microRNAs are believed to be involved in most, if not all, physiological and pathological processes; one can thus anticipate that microRNAs will play a significant role in health and disease.

The role and importance of microRNAs in platelets is gradually emerging. After Kondkar et al. [17] reported findings that support a role for microRNA-96 in the regulation of VAMP8/endobrevin, which is overexpressed in hyperreactive human platelets, Nagalla et al. [10] described a subset of microRNAs that are differentially expressed between subjects grouped according to platelet aggregation to epinephrine. These studies unveiled a link between microRNA profiles and platelet reactivity, and suggest a role for small regulatory RNA species in modulating mRNA translation in platelets.

Moreover, the demonstrated delivery of microRNAs from one cell to another through microvesicles [18], together with the ability of activated platelets to release microparticles [19] and the recently reported ability of platelet-like particles to mediate intercellular mRNA transfer [20], suggest that platelet microRNAs may also fulfill extraplatelet role(s). Together, these observations prompted us to elucidate the complete repertoire and features of human platelet microRNAs by using a high-throughput sequencing (HTS) approach.

## Materials and Methods

### Ethic Statement

Blood collection from healthy volunteers (adult caucasians of both sexes from the immediate region of Quebec City) was approved by our institutional Human Ethics committee (i.e. Comité d'éthique de la recherche du CHUQ-Centre hospitalier de l'Université Laval). The participants provided their written informed consent to participate in this study, as approved by our institutional Human Ethics committee.

### Human Platelet Purification

Platelets were isolated from venous blood as previously described [9]. Briefly, blood from healthy volunteers was collected using sodium citrate (100 mM) as anticoagulant. Platelet-rich plasma (PRP) was obtained by centrifugation at 250 g for 20 min, followed by filtration through leukocyte depletion filters (Pall Corporation). The platelet suspension was subsequently incubated with CD45+ antibody coupled to magnetic microbeads (EasySep, StemCell Technologies), for negative selection of blood platelets. Highly purified platelets were harvested by centrifugation at 6,000 g for 1 min, and either directly lysed in TRIzol (Invitrogen) for RNA extraction or stored as frozen pellet for in vitro assays.

### Platelet RNA Extraction

Total RNA was extracted using TRIzol reagent (Invitrogen). The purity of each platelet preparation was assessed by PCR analysis of platelet (GPIIIa) and leukocyte (CD45) markers, as described previously [9]. Equal amounts of platelet total RNA, extracted from 5 (pool 1) or 14 (pool 2) healthy subjects, were pooled together, and purified further using an RNeasy Mini kit (Qiagen). The integrity of platelet RNA was assessed using Bioanalyzer 2100 (Agilent) prior to further analyses.

### Small RNA Library Construction and Sequencing

Small RNA libraries from pools 1 and 2 were constructed separately, each from 15 µg total RNA, by isolating RNA species between 18 and 30 nt in length using the DGE small RNA sample prep kit (Illumina), following the manufacturer's instructions. The labeled cDNAs were quantified with the Quant-iT PicoGreen dsDNA Kit (Invitrogen) and diluted to 3 pM for sequencing in a single lane on an Illumina 1G Genome Analyzer (Solexa), essentially as described previously [21].

### Raw Sequences Filtering

Raw sequences were filtered through serial quality control criteria. First, the presence of at least 6 nt of the 3′ Solexa adapter was verified. The sequence reads that did not comply with this criterion were discarded, whereas the others were trimmed to remove the adapter sequence harbored at the 3′ end. The remaining tags were further filtered regarding their length (>10 nt), copy number (>4 reads) and readability (>9 non-identified nucleotides, annotated N). Reads complying with all those criteria were subsequently defined as usable reads.

### Analysis of Annotated microRNAs

All the usable reads were aligned to pre-microRNAs extracted from miRBase database (release 18.0). Sequence tags that matched perfectly to more than one precursor were distributed equally among them. In order to account for Drosha and Dicer imperfect cleavage [22], any sequence tag that perfectly matched the pre-microRNA in the mature microRNA region, allowing up to 4 nt shift as compared to the reference mature microRNA position, was considered as a mature microRNA. The microRNA expression level was defined as the number of reads mapping each mature microRNA normalized to the total number of usable reads, considering that the overall number of small RNAs is invariant. The relative abundance of each microRNA was defined as the number of reads mapping each microRNA compared to the total number of reads mapping mature microRNAs.

### Identification of New microRNA Sequences

The analytical approach that we applied for the identification of novel microRNA species in human platelets is depicted in the flowchart shown in Figure S1 and has been described in detail previously [21], [23]. In brief, all the usable reads that did not match to known pre-microRNAs (unknown reads) were mapped to the human genome. For the reads that matched perfectly, the 100 nucleotides flanking each side of the sequence were extracted in order to test whether these resulting ∼220 nt genomic sequences could be transcribed and fold into pre-microRNA-like structures, using the Vienna RNA secondary structure prediction and comparison package. The hairpin structured sequences were trimmed down to remove the sequence portions that were not included in the stem-loop. These sequences were folded again and further tested for their ability to form pre-microRNA structures that meet the criteria previously established by Ambros et al. [24]. These sequences must form a characteristic stem-loop structure with a minimum free energy below −20 kcal/mol, and harbor the putative mature microRNA on either arm of the stem. Hairpin structures that fulfill all the conditions were considered as putative pre-microRNAs. All the unknown reads were subsequently re-aligned to the putative new precursors. These hairpins, from which the predicted mature microRNA sequence matched perfectly to detected reads, were classified as high-confidence pre-microRNA hairpins. If reads corresponding to the opposite strand were also detected, the high-confidence hairpins were considered as confirmed pre-microRNAs, as of miRBase 18.0.

### In vitro Terminal Uridylation and Adenylation Assays

Frozen platelet pellets from 3 different donors, as well as the megakaryoblastic cell line Meg-01 (obtained from ATCC) cultured as described previously [9], were used to assess their ability to perform 3′ terminal nucleotide additions to synthetic microRNA species in vitro. Before enzymatic assay, platelet and Meg-01 protein extracts were treated by RNase digestion in order to decrease the signal of endogenous RNA adenylation/uridylation. Uridylation and adenylation assays were performed on 20 µg of protein extracts, incubated with 10 pmol RNA substrates in the presence of α-^32^P labeled UTP or ATP, respectively. The RNAs were subsequently extracted and adenylated/uridylated RNA species were analyzed on denaturing 10% polyacrylamide gel.

### Western Blot Analysis of Terminal Nucleotidyltransferases

Expression of terminal nucleotidyltransferases in Meg-01 cells and human platelets was assessed by Western blot [9]. Protein extracts (20 µg) were separated by 10% SDS-PAGE, transferred to a polyvinylidene difluoride (PVDF) membrane and immunoblotted using mouse polyclonal anti-TUT4 (ab89165, Abcam) and rabbit polyclonal anti-GLD2 antibodies (ab103884, Abcam) used at the dilution recommended by the manufacturer.

### Reporter Gene Activity Assays

HEK293 cells (2×10^5^) were plated in 24-well plates 24 h before cotransfection with RNA duplexes encoding the reference miR-140-3p or the 1-nt 5′ shifted isomiR (0.2 to 20 pmol) and a psiCHECK (Promega) reporter construct (100 ng DNA) using Lipofectamine 2000. A binding site of perfect complementarity for both miR-140-3p isomiRs or the sequence of adenylate cyclase-associated protein 1 (CAP1) 3′UTR were inserted downstream of the *Renilla* luciferase (Rluc) gene of the psiCHECK reporter. A small interfering RNA (siRNA) targeting the green fluorescent protein (GFP) was used as a negative control, and results obtained with both isomiRs were normalized to those obtained with this non-targeting siRNA. Twenty four (24) h later, cells were harvested and protein extracts were prepared for measurement of Rluc and *Firefly* luciferase (Fluc) activities, essentially as described previously [9].

## Results

### The Small RNA Profile of Human Platelets

**Figure 1:**
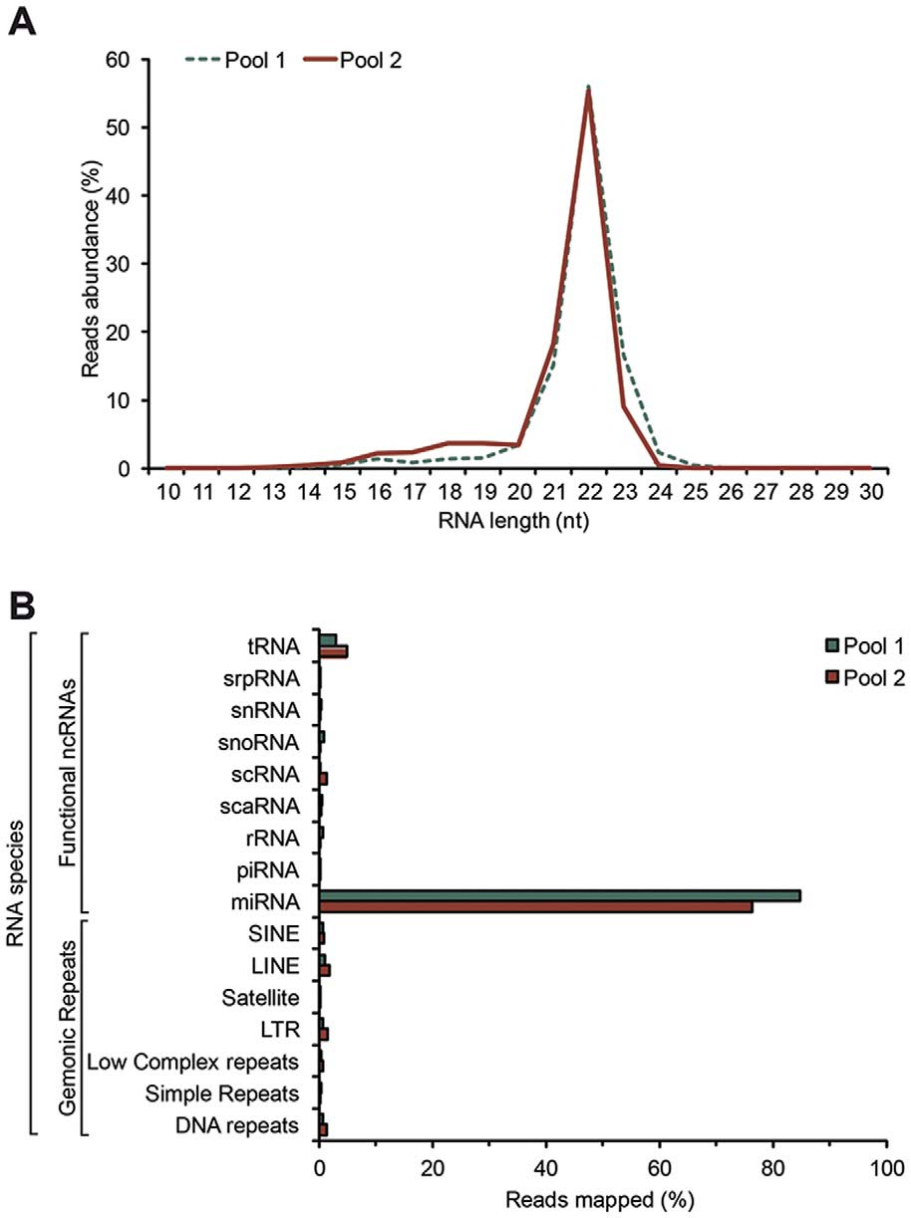
The small RNA profile of human platelets. (A) Size distribution of usable reads detected in each of the platelet RNA pool 1 and 2. Most of the RNA sequences are 21 to 23 nucleotides (nt) in length, representing 88% and 83% of the sequences obtained from pool 1 and pool 2, respectively. (B) Classification of the small RNA species isolated and sequenced from human platelet RNA pool 1 and 2. The proportion of sequences (%) matching to each RNA category is shown. MicroRNAs represent approximately 80% of the small RNA species between 18 and 30 nt in length in human platelets.

Two samples of platelet small RNAs, prepared by pooling equivalent amounts of RNA isolated from either 5 (pool 1) or 14 (pool 2) healthy subjects, were analyzed by HTS. Each pool yielded 3,970,224 and 6,366,352 raw sequences, respectively. From each pool, we retrieved 3,154,352 and 5,680,165 usable reads (for a total of 8.83 M usable reads), corresponding to 19,498 and 18,071 unique tags, respectively. The length distribution analysis of the usable reads, obtained from the 2 different pools, revealed that more than 90% of the sequences were in the 19- to 24-nt window of RNA sizes, which is characteristic of microRNAs (Figure 1A). The HTS datasets are available online in Database S1.

Genome mapping of all the usable reads allowed the assignment of the detected small RNA sequences to various categories among the classes of functional non-coding RNAs and genomic repeats. These analyses unveiled microRNAs as the most abundant small RNA species in human platelets, representing ∼80% of the sequenced RNAs (Figure 1B). Small RNAs originating from genomic repeats collectively represented ∼5% of all the small RNA sequences detected, whereas the other functional non-coding RNAs accounted for ∼6% of the sequenced RNAs. The nuclear localization of scaRNAs and snoRNAs, and the germline-specific expression of piRNAs probably explain their virtual absence in anucleate platelets. In addition, low representation of highly abundant RNA species, such as rRNA and tRNA, reflects the quality of our RNA preparations, as the RNA size window of our libraries (i.e. 18–30 nt) excludes integral rRNA and tRNA from the sequencing analyses.

These results confirm that human platelets are highly enriched for microRNAs, as compared to other classes of small RNAs. Moreover, the significant correlation obtained by plotting the microRNA expression data from Pool 1 versus Pool 2 (Figure S2) supports the validity of our RNA pooling approach involving a relatively small number of subjects, as well as merging of our data.

### The Annotated microRNA Profile of Human Platelets

Further analyses of our HTS data focused on characterizing the diversity and relative abundance of microRNAs in platelets. In order to establish the microRNA expression profile of human platelets, sequence reads were aligned to the pre-microRNAs annotated in miRBase database, release 18.0 (http://www.mirbase.org/). A total of 492 mature microRNA sequences were detected in our human platelet RNA preparations, out of 1870 mature microRNAs annotated in miRBase 18.0. Whereas the majority of mature microRNAs (285) could be detected in both Pool 1 and Pool 2, microRNAs that were detected in only one of the two pools represented less than 0.01% of all detected microRNAs, in terms of relative abundance, which might reflect the high variability and diversity of lowly expressed microRNA in human platelets.

**Figure 2:**
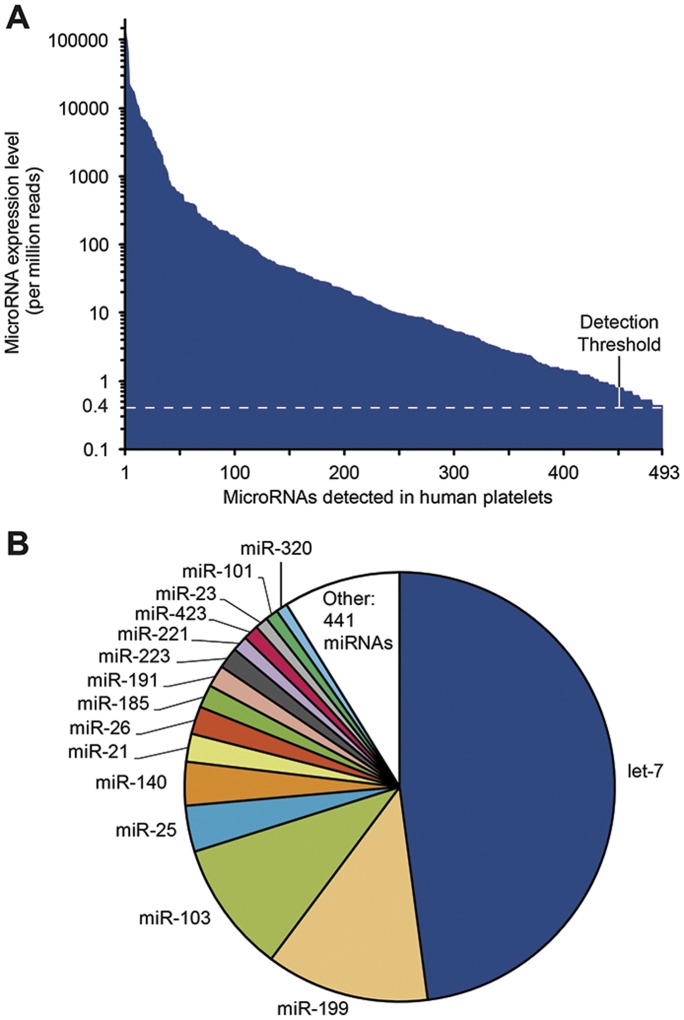
The annotated microRNA profile of human platelets. (A) The HTS profiling of platelet microRNAs, which is shown in order of decreasing number of reads. (B) Distribution of the 15 most abundant mature microRNA families detected in human platelets. The let-7 microRNA family represented 48% of a total of 492 different mature microRNAs, whereas the 15 most abundant microRNAs accounted for more than 90% of all platelet microRNAs.

As depicted in Figure 2A, platelet microRNA expression levels covered a range greater than 5 orders of magnitude, a range more than 2-fold greater than that reported using a microarray approach [9]. The most abundant microRNAs are members of the let-7 microRNA family, which represented 48% of the platelet microRNA content (Figure 2B). Other microRNA families were highly represented in human platelets, such as miR-103 and miR-25 (Table 1). Overall, the 15 most abundant microRNA families represented more than 90% of all microRNAs present in human platelets.

**Table 1:** The 15 most abundant microRNA families detected in human platelets.

MicroRNA Family	Mature microRNA	Expression level (per million reads)	Relative abundance
let-7	hsa-let-7f-5p	158765.9	47.9%
	hsa-let-7a-5p	118095.6	
	hsa-let-7g-5p	20681.4	
	hsa-let-7b-5p	18029.4	
	hsa-let-7d-5p	17526.2	
	hsa-let-7i-5p	6714.4	
	hsa-let-7e-5p	3711.6	
	hsa-let-7c	1231.3	
	hsa-mir-98	397.7	
	hsa-let-7d-3p	54.1	
	hsa-let-7i-3p	4.6	
	hsa-let-7b-3p	2.9	
	hsa-let-7a-3p	2.5	
mir-199	hsa-mir-199a-3p	95224.1	12.3%
	hsa-mir-199b-5p	51.2	
	hsa-mir-199a-5p	17.6	
mir-103	hsa-mir-103a-3p	63934.9	9.9%
	hsa-mir-107	6876.4	
	hsa-mir-103a-2-5p	1.4	
mir-25	hsa-mir-25-3p	19393.5	3.5%
	hsa-mir-92a-3p	4902.8	
	hsa-mir-25-5p	136.1	
	hsa-mir-92b-3p	56.4	
	hsa-mir-92a-1-5p	12.2	
	hsa-mir-92b-5p	1.6	
mir-140	hsa-mir-140-3p	22540.7	3.3%
	hsa-mir-140-5p	5.7	
mir-21	hsa-mir-21-5p	15428.8	2.1%
mir-26	hsa-mir-26b-5p	9511.2	2.1%
	hsa-mir-26a-5p	5428.6	
	hsa-mir-26a-1-3p	1.1	
mir-185	hsa-mir-185-5p	13029.3	1.7%
	hsa-mir-185-3p	99.0	
mir-191	hsa-mir-191-5p	11314.8	1.7%
	hsa-mir-191-3p	5.2	
mir-223	hsa-mir-223-3p	10861.4	1.6%
	hsa-mir-223-5p	157.2	
mir-221	hsa-mir-221-3p	7431.4	1.2%
	hsa-mir-221-5p	559.5	
	hsa-mir-222-3p	551.2	
mir-423	hsa-mir-423-5p	7507.1	1.1%
	hsa-mir-423-3p	688.1	
mir-23	hsa-mir-23a-3p	6276.4	1.0%
	hsa-mir-23b-3p	282.6	
	hsa-mir-23a-5p	12.9	
	hsa-mir-23b-5p	6.0	
mir-101	hsa-mir-101-3p	6752.1	1.0%
	hsa-mir-101-5p	1.7	
mir-320	hsa-mir-320a	6191.1	0.9%
	hsa-mir-320b	49.3	
	hsa-mir-320c	7.7	
	hsa-mir-320d	2.6	
Other	441 microRNAs	64002	8.8%

The data pertaining to the 50 most abundant platelet microRNAs, irrespective of their family, are compiled in Table S2 and show that individual microRNAs, such as miR-199a/b-3p, miR-140-3p and miR-21, also accounted for an important proportion of human platelet microRNAs.

### Discovery of Novel microRNAs in Human Platelets

One of the major advantages of HTS, as compared to microarray and PCR-based detection methods, is the possibility to discover new RNA sequences. The processing of microRNA precursors (pre-microRNAs) yields a microRNA duplex, from which one (or both) of the strand is incorporated in the microRNP effector complex, while the other one is generally believed to be degraded. As a consequence, only one of the 2 putative mature microRNA sequences is currently known for numerous microRNA duplexes (60% of all pre-microRNAs referenced in miRBase, release 18.0). In total, 15 novel mature sequences from known pre-microRNAs have been detected in human platelets (Table 2). These findings confirm the existence and localization of the putative Drosha and Dicer cleavage sites on the pre-microRNA sequences from which both mature microRNA strands derive, and that were predicted solely on the experimental cloning of one of the mature microRNA strands.

**Table 2:** Novel mature microRNAs derived from known microRNA precursors (pre-microRNAs).

Pre-microRNA	MicroRNA sequence	Reads detected	Strand
**hsa-mir-1537**	AGCUGUAAUUAGUCAGUUUUCU	17	5p
**hsa-mir-1908**	CCGGCCGCCGGCUCCGCCCCG	9	3p
**hsa-mir-1910**	GAGGCAGAAGCAGGAUGACA	32	3p
**hsa-mir-412**	UGGUCGACCAGUUGGAAAGUAAU	74	5p
**hsa-mir-433**	UACGGUGAGCCUGUCAUUAUUC	12	5p
**hsa-mir-450a-1**	AUUGGGAACAUUUUGCAUGUAU	16	3p
**hsa-mir-487a**	GUGGUUAUCCCUGCUGUGUUCG	37	5p
**hsa-mir-5189**	UGCCAACCGUCAGAGCCCAGA	27	3p
**hsa-mir-548e**	CAAAAGCAAUCGCGGUUUUUG	41	5p
**hsa-mir-548j**	CAAAAACUGCAUUACUUUUGC	28	3p
**hsa-mir-605**	AGAAGGCACUAUGAGAUUUAGA	31	3p
**hsa-mir-619**	GCUGGGAUUACAGGCAUGAGCC	5	5p
**hsa-mir-627**	UCUUUUCUUUGAGACUCACU	10	3p
**hsa-mir-758**	AUGGUUGACCAGAGAGCACACG	5	5p

In order to identify mature microRNAs from novel pre-microRNAs, we used the flowchart shown in Figure S1. Our approach yielded the discovery of 21 novel pre-microRNA sequences, including 4 for which both mature strands could be detected (Table 3). These microRNAs displayed a relatively low level of expression, and the majority of them could only be detected in one of the 2 pools, suggesting a certain degree of interindividual variations. Whether these microRNAs come from single individuals remains a possibility that would need to be assessed by investigating the platelet microRNAs of individual subjects. Of notice, most of these newly discovered microRNA hairpins originate from intronic sequences, which might be a characteristic of platelet microRNAs, whereas two of the new hairpins, i.e. hairpin 02 and 16, are expressed from the antisense strand of microRNAs miR-4433 and miR-5683, respectively.

**Table 3:** Novel mature microRNA sequences detected in human platelets.

Hairpin	Mature Sequence	Reads	Genomic position	Genomic context
**01**	**3p-AUGAAGCCUUCUCUGCCUUACG**	**6**	14(+): 72983574	RGS6 (Intron)
	**5p-GAGGGCAGAGCCAGCUUCCUGA**	**5**		
**02**	**3p-CAGGAGUGGGGGGUGGGACGU**	9130	2(−): 64567903	AL355732 (Intron)
	**5p-AUGUCCCACCCCCACUCCUGU**	34		hsa-miR-4433 (AS)
**03**	**3p-CUGCAGACUCGACCUCCCAGGC**	81	16(+): 24214507	PRKCB (Intron)
	**5p-CUGGGAGGUCAAGGCUGCAGU**	39		
**04**	**3p-AUCAUGUAUGAUACUGCAAACA**	25	17(+): 75085510	c17orf86 (intron)
	**5p-UUUGCAGUAACAGGUGUGAGCA**	23		/SCARNA16
**05**	**5p-AAAACUAGGACUGUGUGGUGUA**	21	12(−): 94965082	TMCC3 (3'-UTR)
**06**	**5p-AAGGGACAGGGAGGGUCGUGG**	50	2(+): 20803112	KLF7 (Intron)
**07**	**5p-AGAUGCCGGGGUCUCUGUGUGC**	12	20(+): 35990206	SRC (Intron)
**08**	**3p-CAGCGGAGCCUGGAGAGAAGG**	5	1(+): 12227067	TNRFSF1B (ORF)
**09**	**3p-CGUGGAGGACGAGGAGGAGGC**	12	11(+): 1901335	LSP1 (ORF)
**10**	**3p-CUACCCUCGGUCUGCUUACCACA**	7	8(−): 134058739	SLA/TG (Intron)
**11**	**3p-GACAAUUGUUGAUCUUGGGCCU**	5	4(+): 147329798	SLC10A7 (Intron)
**12**	**3p-GAUGCCUGGGAGUUGCGAUCUGC**	6	22(+): 43011374	RNU12
**13**	**5p-GUUUGGACAUAGUGUGGCUGG**	7	19(+): 2630721	GNG7 (Intron)
**14**	**3p-UACCUGGGAGACUGAGGUUGGA**	5	12(−):42717542	ZCRB1 (Intron)
**15**	**3p-UAUGUAGUAGUCAAAGGCAUUU**	9	1(−): 108439846	VAV3 (Intron)
**16**	**5p-UCAAAUGCAGAUCCUGACUUC**	9	6(−): 6169613	F13A1 (Intron)
			–	hsa-miR-5683 (AS)
**17**	**5p-UGAGGGACCCAGGACAGGAGA**	5	7(+): 100465663	TRIP6 (Intron)
**18**	**3p-UGAGGUGACCGCAGAUGGGAA**	5	16(+): 81567547	CMIP (Intron)
**19**	**3p-UUGGCUGGUCUCUGCUCCGCAG**	6	8(+): 27290930	PTK2B (Intron)
**20**	**5p-UUGGUGAGGACCCCAAGCUCGG**	6	14(−):65252381	SPTB (Intron)
**21**	**5p-UUUUAAGGACACUGAGGGAUC**	6	1(−): 86823348	ODF2L (Intron)

The existence of 5 of the novel microRNA candidates was validated by Northern blot hybridization (Figure S3). For Hairpin 03, a ∼75-nucleotide (nt) pre-microRNA, a ∼35-nt intermediate and the ∼21-nt mature microRNA (indicated by *) were only detected in the platelet RNA sample. A microRNA species migrating at ∼35 nt was also detected for hsa-miR-2115, and may represent a cleavage intermediate containing loop sequences resulting from asymmetrical processing of the pre-microRNA substrate [25], although this possibility remains to be confirmed.

The relatively weak intensity of the microRNA bands revealed by Northern blotting is consistent with the relatively low number of reads obtained from these microRNAs, which may explain why some of them were discovered only recently. qPCR analyses confirmed expression of 4 novel microRNAs detected by HTS in human platelets (Figure S4). These findings expand further the microRNA repertoire of human platelets and raises the possibility of platelet-specific microRNA sequences.

### Post-transcriptional Modifications of microRNAs in Human Platelets

**Figure 3:**
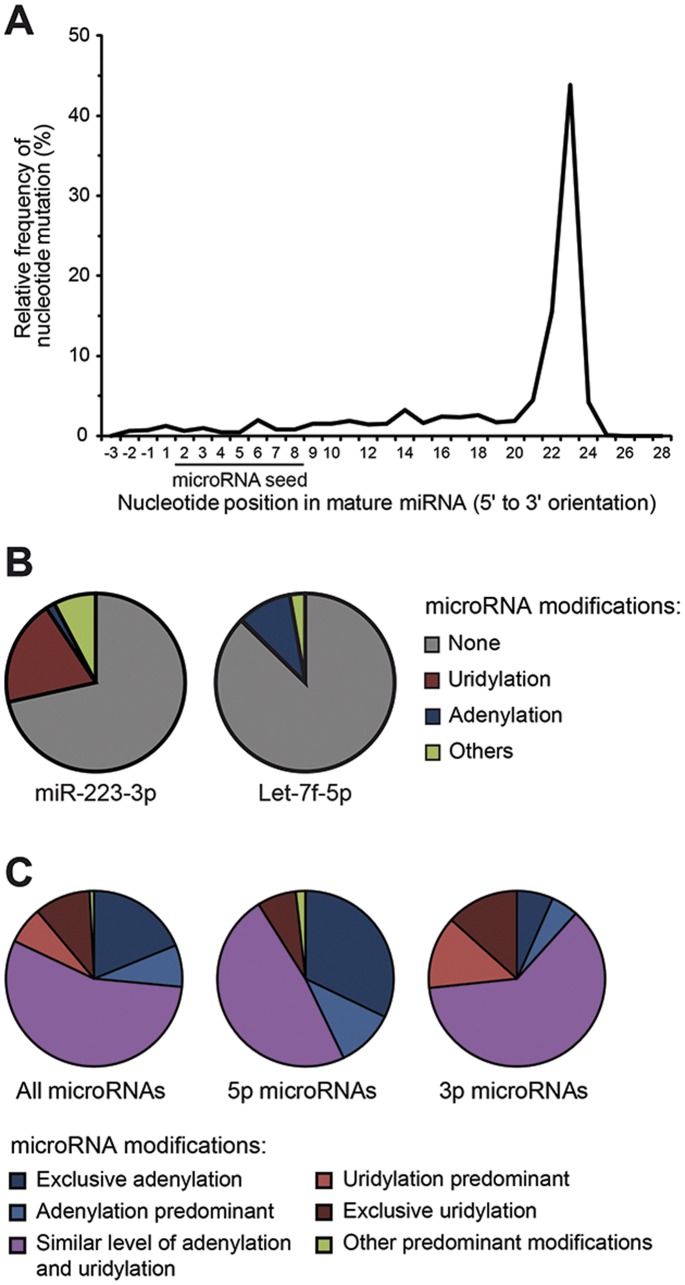
Post-transcriptional modifications of platelet microRNAs. (A) The relative frequency of nucleotide mutation ordered by position on the mature microRNA sequences. The majority of the nucleotide modifications were observed at the 3′ end of microRNAs, whereas the sequence of their seed region was relatively well preserved. (B) Analysis of post-transcriptional modifications affecting two highly expressed platelet microRNAs, miR-223-3p and let-7f-5p. Whereas miR-223-3p showed preferential uridylation, let-7f-5p exhibited predominant adenylation. These modifications represented 29% and 13% of the expressed forms of miR-223-3p and let-7f-5p, respectively. (C) Prevalence of the modifications observed in microRNAs that displayed more than 10% modified sequences in abundance. MicroRNAs for which adenylated/uridylated sequences were at least 10 times more abundant than other modifications were classified as predominant adenylated/uridylated microRNAs. Similar level (e.g. within a 10-fold range) of adenylated and uridylated forms were detected for the majority (56%) of microRNAs. Exclusive or predominant adenylation was observed for 43% of the 5p-microRNAs as compared to 12% of the 3p-microRNAs. On the contrary, exclusive or predominant uridylation was more frequent in 3p-microRNAs (27%), as compared to 5p-microRNA (7%).

Previous studies reported that mature microRNAs can be modified post-transcriptionally, a process that can influence their stability and/or mRNA target recognition. Most of these modifications consist in the 3′ terminal addition of nucleotides U or A through uridylation and adenylation reactions, respectively, and generally do not exceed 2 to 3 nucleotides additions. In order to document platelet microRNA modifications, the remaining tags that did not match exactly to any precursors were re-aligned by allowing up to 3 mismatches as compared to the canonical sequence. As depicted in Figure 3A, mutations were predominantly located at positions 21 to 24 of mature microRNAs, which is consistent with preferential 3′ end modifications. In contrast, nucleotides 2 to 8 forming the microRNA seed region were relatively well preserved, which tends to support a functional role for this motif in platelet microRNAs. We were able to detect adenylated as well as uridylated derivatives for the majority of the modified platelet microRNAs. Whereas some microRNAs showed preferential adenylation, like let-7f-5p (Figure 3B, left panel), other microRNAs displayed preferential uridylation, as in the case of miR-223-3p (Figure 3B, right panel). MicroRNA sequence editing events were also detected among platelet small RNA species, consistent with a previous study performed on mouse tissues [26], enhancing further the diversity of platelet microRNA sequences.

Pre-microRNA species may be uridylated [27], adenylated or cytidinylated [28]. Since our RNA sequencing was restricted to 18–30 nt RNA species, precursor 3′ end modifications could only be detected indirectly by analyzing the mature microRNA species released from the 3p strand. For this purpose, we analyzed whether the origin of mature microRNA from the precursor (e.g. deriving from 5p- or 3p- strand) influenced their terminal modification. The average relative abundance of modified 5p- and 3p-microRNA were respectively of 11% and 15%. These similar levels of modified 3p- and 5p-microRNAs suggest a minor contribution (4%) of precursor modifications and rather reflect that terminal modifications of platelet microRNAs occur predominantly on mature microRNA strands. Next, we analyzed whether the type of terminal modification was influenced by the strand origin of microRNAs. For that purpose, we focused our analyses on the microRNAs that were detected in both RNA pools and for which the modified forms accounted for more than 10% of the microRNA sequences, in terms of abundance. Similar levels of uridylated and adenylated forms were observed for the majority of these microRNAs (Figure 3C, left pie chart). However, microRNAs with predominant or exclusive adenylation more frequently derived from the 5p strand (43% of modified 5p microRNAs) (Figure 3C, center pie chart) versus microRNAs derived from the 3p strand (12% of modified 3p microRNAs) (Figure 3C, right pie chart). In contrast, microRNAs with predominant or exclusive uridylation more frequently derived from the 3p strand (27% of modified 3p microRNAs) (Figure 3C, right pie chart), as compared to microRNAs derived from the 5p strand (7% of modified 5p microRNAs) (Figure 3C, center pie chart). The relatively high frequency of adenylated 5p-microRNAs suggests that this modification occurs predominantly on mature microRNA species, whereas terminal uridylation, which is more frequent on 3p strand, might occur both at pre-microRNA and mature microRNA levels.

### MicroRNA Species may be Modified in Human Platelets

**Figure 4:**
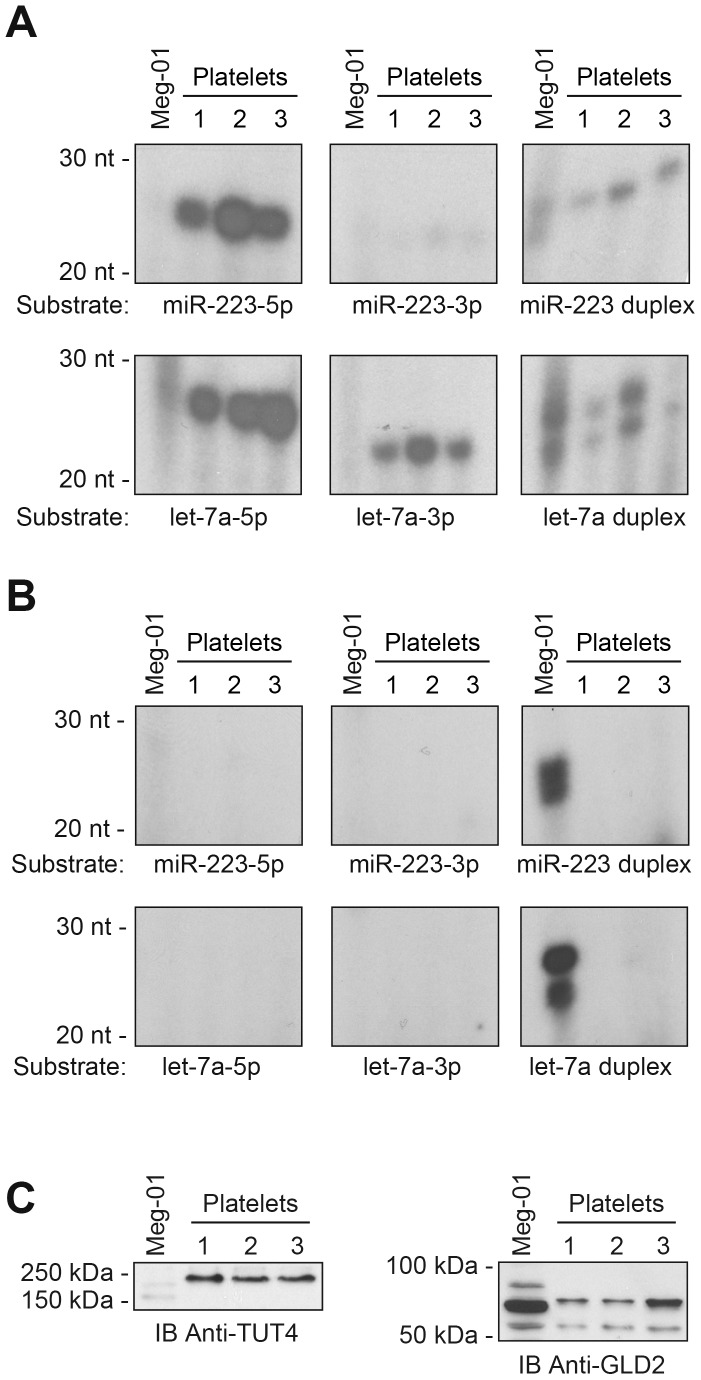
Platelets possess microRNA terminal nucleotidyltransferase activity. (A–B) Uridylation (A) and adenylation (B) assays were performed by incubating synthetic miR-223 or let-7a mature microRNAs or microRNA duplexes with protein extracts, prepared from Meg-01 cells or platelets isolated from 3 healthy volunteers, in the presence of α-^32^P-labeled UTP or ATP respectively, at 30°C for 90 min. Uridylated or adenylated forms of microRNAs were detected by denaturing PAGE and autoradiography. (C) Western blot detection of TUT4 uridyltransferase (left panel) and GLD2 adenyltransferase (right panel) enzymes in Meg-01 and platelet extracts using anti-TUT4 and anti-GLD2 antibodies, respectively.

In order to verify whether platelets can uridylate or adenylate microRNA species, we performed in vitro uridylation and adenylation assays by incubating synthetic mature microRNAs or duplexes of microRNAs derived from miR-223 and let-7a in the presence of platelet protein extracts. Our results indicate that platelets can uridylate single-stranded microRNAs, as compared to the megakaryoblastic cell line Meg-01 (Figure 4A). The uridylation activity varied among the microRNAs tested, which may be related to the nature of the 3′ extremity: the two microRNAs that were the most readily uridylated exhibit a terminal U (let-7a-5p and mir-223-5p) (Figure 4A, left panels). In comparison, let-7a-3p exhibiting a terminal C was uridylated to a lesser extent (Figure 4A, center lower panel), whereas miR-223-3p harboring a terminal A was barely uridylated (Figure 4A, center upper panel).

The capacity of platelets to uridylate microRNA duplexes was relatively lower than single-stranded microRNAs. In contrast, Meg-01 cells displayed a preferential uridylating activity for microRNA duplexes (Figure 4A). These results indicate that, in addition to inherit of uridylated microRNAs from its precursor megakyocyte cell, platelets can uridylate its microRNA species, which may account for a proportion of the uridylated microRNAs that we detected by HTS.

We were unable to detect significant microRNA adenylation activity in human platelets (Figure 4B), although other endogenous small RNA species could be adenylated (H.P. and P.P., data not shown). As observed with uridylation, Meg-01 cells displayed preferential adenylating activity for microRNA duplexes, as compared to single-stranded microRNAs (Figure 4B). The undetectable terminal adenyltransferase activity of human platelets suggests that the adenylated forms of microRNAs may be passed on to platelets from their megakaryocytic precursor cells.

As many as seven human nucleotidyltransferases have been involved in microRNA modifications [29], and specific catalytic activity on microRNAs was demonstrated for two of them, namely TUT-4 (ZCCHC11) [30] and GLD2 (PAPD4) [31]. Western blot analyses, aimed at detecting these two enzymes, revealed substantially higher levels of TUT4 expression in platelets compared to Meg-01 cells (Figure 4C, left panel). This may explain the similar difference in terms of uridyltransferase activity observed between these two cell types (Figure 4A).

On the other hand, the GLD2 adenyltransferase enzyme exhibited a higher expression level in Meg-01, as compared to platelets (Figure 4C, right panel), which is consistent with the higher level of adenyltransferase activity measured in vitro (Figure 4B).

### Detection of Numerous microRNA Isoforms in Human Platelets

**Figure 5:**
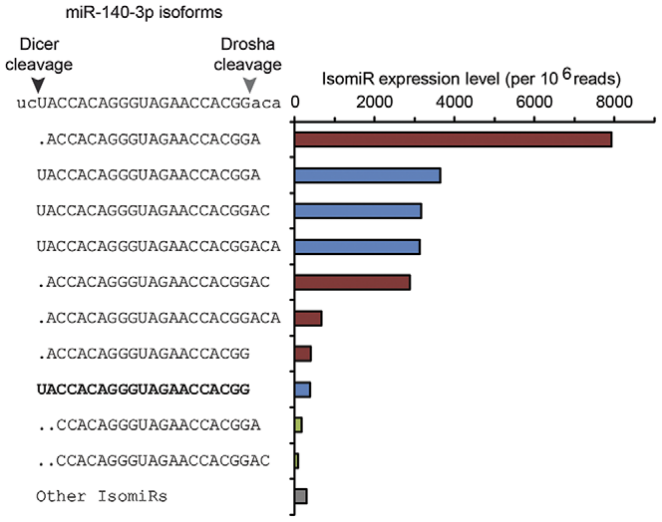
Detection of multiple microRNA isoforms in human platelets. The expression level of the main isoforms of miR-140-3p are shown. The expected mature microRNA sequence, as retrieved from miRBase, is highlighted in bold and does not correspond to the most frequently encountered isoform. As shown on the left, most of the miR-140-3p isoforms may result from a combination of imprecise processing by Drosha and/or Dicer, including the most abundant, whose cleavage sites on the pre-microRNA species are shifted towards the 3′ end by a single nucleotide. Of notice 2 major miR-140-3p isomiRs population coexist in platelets depending on 5′clivage by Dicer, either at the canonical position (blue bars) or harboring a 1 nt cleavage shift (red bars).

As reported previously in other human RNA samples [22], multiple microRNA isoforms (or isomiRs) were detected in human platelets. These isoforms result from the imperfect cleavage of the pre-microRNA species and/or the post-transcriptional modifications of microRNAs. Since a sizeable proportion of sequence reads aligning with some of the pre-microRNAs from miRBase were isomiRs, we were prompted to consider the microRNA sequences that perfectly matched the pre-microRNA in the mature microRNA region, allowing up to 4 nt shift as compared to the reference mature microRNA position. As exemplified with miR-140-3p, the most abundant sequences that match pre-microRNAs may represent an isomiR; here, the reference miR-140-3p sequence represents only a minority of the reads (Figure 5, in bold). In contrast to cleavage shifts at the 3′ extremity, which was observed for most, if not all, microRNAs, 5′ cleavage shifts were less frequent. This is in accordance with previous studies on nucleated cells [32] and reflects the functional importance of the 5′ sequence of platelet microRNAs, especially the positionining of the microRNA seed region.

### Redirection of Platelet microRNA Targeting Properties upon a 5′ Cleavage Shift

**Figure 6:**
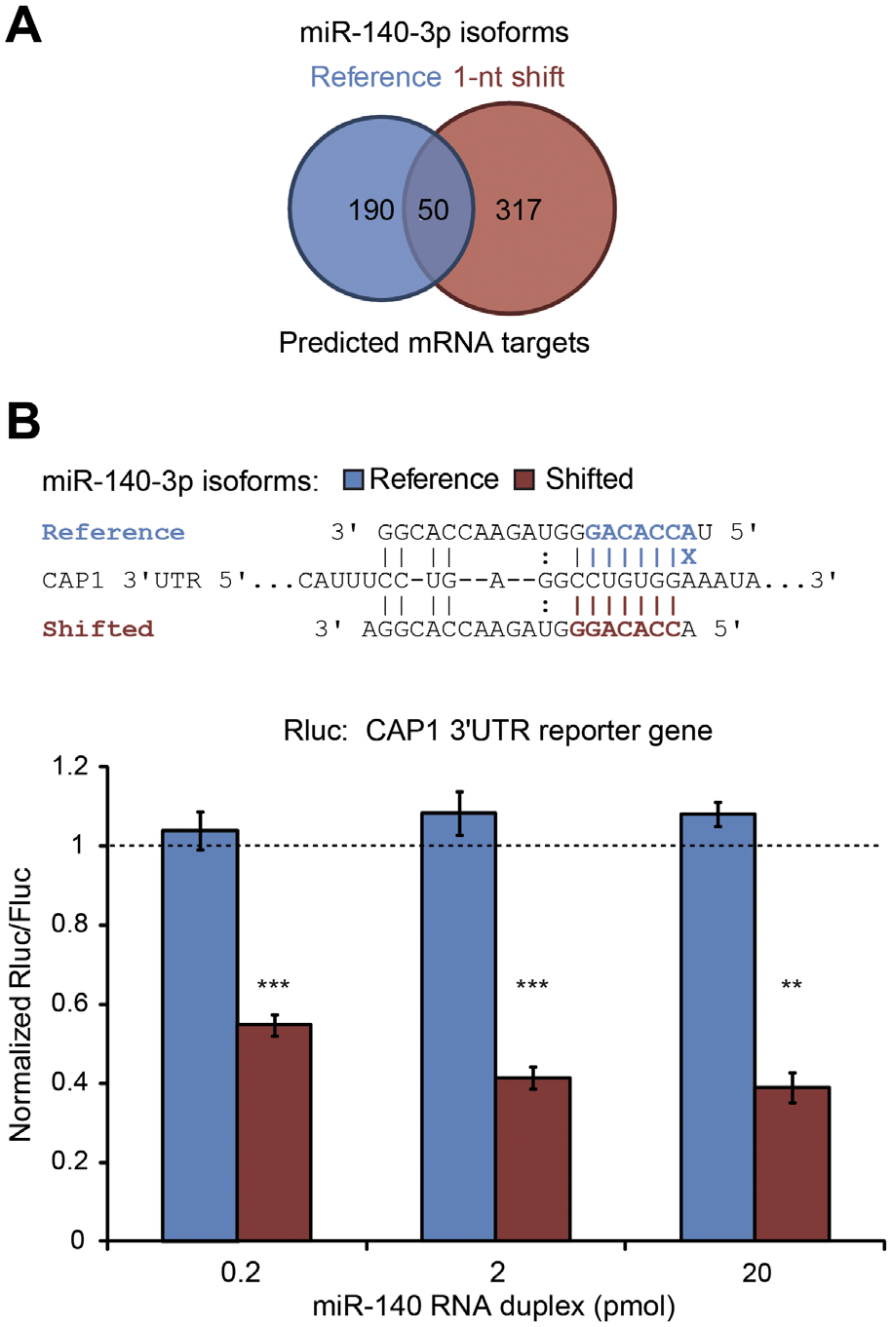
Redirection of platelet miR-140-3p targeting upon a 1-nt shift of the 5′ cleavage site. (A) mRNA target prediction for the reference mature miR-140-3p sequence and the miR-140-3p isomiR harboring a 1-nt 5′ shift using TargetScan. The reference isomiR potentially regulates 240 different mRNA targets, whereas the shifted isomiR potentially regulates 367 mRNA targets. Notably, only 50 of these mRNAs are predicted to be regulated by both forms of miR-140-3p. (B) Experimental validation of the target specificity of the 1-nt 5′ shifted isomiR for CAP1 mRNA. HEK 293 cells were co-transfected with miR-140 microRNA duplexes, containing either the reference microRNA sequence (Reference) or the most abundant 1-nt 5′ shifted isomiR (Shifted), and a reporter gene construct in which the adenylate cyclase-associated protein 1 (CAP1) 3′UTR was inserted downstream of the Rluc reporter gene. Base pairing complementarity between miR-140-3p microRNAs and their binding sites is shown in the upper panel. Base pairing involving the microRNA seed region is highlighted in color. The blue X denotes the loss of base pairing of nt 2 of the miR-140-3p reference isoform, which may explain its lower efficiency in regulating CAP1 mRNA 3′UTR expression. Rluc and Fluc activities were measured, and the values were normalized to those obtained with a non-relevant RNA duplex (n = 3 to 4 experiments, in duplicate) (lower panel). ** p<0.01, *** p<0.001 (Tukey-Kramer Multiple Comparisons Test).

A restricted number of microRNAs harbor 5′ shifted isomiRs at a relatively high frequency. Among them is miR-140-3p, which is one of the most abundant microRNA in platelets (please refer to Figure 2B). The detected miR-140-3p isoforms were distributed as follows: 46% without a 5′ shift and 54% with a 1-nt 5′ shift. This latter feature is of particular interest, as it modifies the composition of the microRNA seed sequence, which confers the mRNA specificity of microRNAs. mRNA target prediction analysis of the reference mature miR-140-3p sequence using TargetScan revealed that this microRNA potentially regulates 240 different mRNA targets, whereas the miR-140-3p isomiRs harboring a 1-nt 5′ shift potentially regulates 367 mRNA targets. Notably, only 50 of these mRNAs are predicted to be regulated by both forms of miR-140-3p (Figure 6A). Seven (7) mRNA targets that are predicted to be regulated by either miR-140-3p isoforms (Table S1) are present in platelets and are involved in platelet activation and degranulation, as referenced in the Gene Ontology (GEO) database.

In order to assess the functionality of the major miR-140-3p isomiR, which results from a 1-nt shift of the Drosha and Dicer cleavage sites in the 3′ orientation, we employed a reporter gene activity assay involving cotransfection of RNA duplexes encoding either the reference miR-140-3p or the major 1-nt shifted isomiR with a Rluc reporter construct in cultured HEK293 cells. Both miR-140 RNA duplexes generated functional miR-140-3p that could silence the activity of a Rluc reporter gene harboring a binding site perfectly complementary to either forms of miR-140-3p (Figure S5).

In order to demonstrate the mRNA target specificity of the 1-nt shifted miR-140-3p, either forms of miR-140-3p were cotransfected with a reporter construct containing adenylate cyclase-associated protein 1 (CAP1) mRNA 3′UTR sequence inserted downstream of the Rluc reporter gene. As shown in Figure 6B (in blue), the reference miR-140-3p sequence had no regulatory activity on the Rluc reporter gene placed under the control of CAP1 3′UTR, which is consistent with TargetScan prediction. In contrast, the 1-nt shifted miR-140-3p isomiR significantly reduced reporter gene expression controlled by the CAP1 3′UTR element (Figure 6B, in red). The differential regulatory effects of the reference and 1-nt shifted miR-140-3p microRNAs may be explained by the CAP1 mRNA regulatory abilites conferred by pairing of nt 2 of the 1-nt shifted miR-140-3p isomiR. Indeed, the architecture of microRNA:mRNA complexes usually involves perfect base pairing of the microRNA seed region, comprised of nucleotides 2 to 8 from the 5′ end, which is critical to the mRNA regulatory properties of microRNAs [33]. Having different seed regions, reference and 1-nt shifted microRNA sequences are thus most likely to target different mRNAs. These results suggest that the relative processing inaccuracy of Drosha and Dicer may modulate the mRNA targeting specificity and greatly expand the diversity of mRNAs targeted and regulated by platelet microRNAs.

## Discussion

This study presents the complete repertoire and features of human platelet microRNAs. In order to obtain data that would be most representative of the human population, we opted for a pooling strategy so to minimize the influence of inter-individual variability. Previous studies have shown it to be a valid alternative to biological replicates at much reduced cost for large-scale gene expression approaches [34], [35], [36].

The microRNA profile of human platelets is characterized by a small number of highly expressed microRNAs or microRNA families. Members of the let-7 family were the most abundant, representing 48% of all platelet microRNAs. This feature, observed in a terminally differentiated element, is consistent with a role for let-7 microRNAs in cell differentiation [37]. Although let-7a and let-7d have been shown to be down-regulated during in vitro CD34+ megakaryocytic differentiation [38], whether the level of let-7 microRNA family members change during the megakaryocyte-proplatelet-platelet transition remains to be determined. Other highly expressed platelet microRNAs, such as miR-223, have been previously involved in thrombopoiesis. Considered as a specific marker of the myeloid cell lineage, miR-223 expression is decreased when myeloid progenitor cells are committed into erythroid lineage and, conversely, up-regulated during megakaryocytic differentiation [38], [39], [40]. Similar decreases during erythroid differentiation were also described for other highly abundant platelet microRNAs, such as miR-103 [41] and miR-221 [42]. MicroRNAs of the miR-23a/27/24-2 cluster, which show a particularly high expression level, are also involved in myeloid commitment [43]. Human platelets thus harbor an abundant array of microRNAs involved in myeloid cell differentiation, megakaryocytopoiesis and thrombopoiesis. Likely representing a hallmark of terminal cell differentiation, the question as to whether these microRNAs remain biologically relevant and important to platelet function remains unanswered.

The diversity of platelet microRNA sequences is reflected both in terms of sequences and relative abundance, as the microRNA content of platelets covers a range greater than 5 orders of magnitude, i.e. more than 2-fold greater than that reported previously [9]. This may be related to the superior sensitivity of the HTS approach versus that of sequence-dependent, hybridization-based micro-array approaches. As well, the observed difference between the expression pattern of individual let-7 family members and of other highly expressed microRNAs (eg, miR-142, miR-185 and miR-126), versus that previously published by our group [9] may also pertain, in addition to interindividual variations, to the microRNA detection method used, i.e. microarray [9], a hybridization-based approach that may yield different results depending on the normalization procedure, versus the more advanced high-throughput sequencing technology (this study). More than 532 different microRNAs (or 28% of the 1870 human microRNAs annotated in miRBase, release 18.0) could be detected by HTS, including 40 novel, previously unannotated mature microRNAs, illustrating the rich diversity in platelet microRNA sequences. This is in accordance with microarray [10] and quantitative PCR [11] data reported by independent groups.

Like the analysis of the pre-microRNA species provided key insights and led to the discovery of the enzyme that produces them, i.e. Drosha [44], a detailed analysis of the platelet microRNA sequences would be expected to provide important insights into their origin, biogenesis and function. For instance, the relative enrichment of platelets in microRNAs, as compared to a nucleated blood cell of myeloid origin (eg, neutrophils) or their megakaryocytic precursor cells [9], is consistent with the absence of microRNA-degrading enzymes in platelets and/or the existence of a microRNA partitioning mechanism.

Platelet microRNAs are subjected to sequence variability and 3′ modifications, whereas the 5′ extremity is relatively well preserved, as observed in nucleated cells [32]. Modifications at the 3′ end of microRNAs may have functional implications, as they may affect microRNA biogenesis and stability. For example, uridylation of let-7 pre-microRNAs prevents their processing by Dicer and promotes microRNA degradation [45]. In contrast, 3′ terminal adenylation by GLD2 has been reported to specifically stabilize miR-122 expression levels in mouse liver [31]. Terminal modifications of mature microRNAs may also influence their mRNA target silencing efficiency, as demonstrated by the abrogation of miR-26b-mediated silencing of IL-6 expression upon uridylation by TUT4 [30]. As for microRNA adenylation by GLD2, it is thought to alter mRNA target silencing efficiency of microRNAs by decreasing their incorporation in Argonaute protein complexes [46].

The similar abundance of 3′ modifications sustained by microRNA species deriving from the 5p or 3p strands of pre-microRNAs suggests that most microRNA modifications are subsequent to pre-microRNA processing. However, we observed that the nature of the modifications varies depending on the strand origin of the microRNA, as the 3p-microRNAs were preferentially adenylated and the 5p-microRNAs preferentially uridylated. Although the molecular mechanisms underlying the differential, polar modifications of platelet microRNAs remain unclear, they are likely to occur during microRNA biogenesis, possibly before strands separation (i.e. at the microRNA duplex level), and to involve a specific structure/nucleotide context. These modifications are expected to influence the biogenesis, strand selection, stability and effectiveness of platelet microRNAs [27], [31], [46], which may be crucial for an anucleate element bereft of de novo transcription. The detection of microRNA terminal uridyltransferase (TUTase) activity in platelets, which appear to be distinct from that documented in the megakaryoblastic cell line Meg-01, correlates with the presence of TUT4. The higher level of TUT4 expression in platelets compared to Meg-01 cells might explain the increased TUTase activity observed *in vitro*. The presence and relative contribution of the other six known TUTase enzymes (TUT1 to TUT7) [27] to platelet microRNA terminal uridylation, however, remains to be determined.

In contrast, we were not able to detect microRNA adenylation activity in assays using platelet extracts in vitro, although it remains possible that the experimental setting/conditions may need to be optimized more thoroughly. The apparent lack of terminal adenyltransferase activity in human platelets suggests that the adenylated forms of platelet microRNAs are likely inherited, already modified, from their megakaryocytic precursor cells. This possibility is supported by the microRNA duplex adenylating properties of Meg-01 cells. Therefore, our results indicate that platelets would have the potential to modulate the activity and stability of its microRNA content through terminal uridylation, whereas the regulation of its microRNAs through adenylation events would be initiated in its megakaryocytic precursor cell.

We identified a restricted number of microRNAs harboring a relatively high rate of 5′ cleavage shift. This feature determines the composition of the microRNA seed sequence, which plays a critical role in mRNA recognition [33], thereby markedly expanding the repertoire of mRNAs that could be regulated upon expression of a single microRNA gene in human platelets. Redirection of the mRNA targeting abilities of 5′ shifted microRNAs, for which we used miR-140-3p as a proof-of-principle, is in accordance with a recent report in cardiomyocytes [47] and confirms the mRNA targeting specificity and functionality of the 5′ shifted platelet isomiRs. Interestingly, variations of the 5′ sequence of platelet microRNAs are less frequent than those observed at their 3′ extremity, a region that is dispensable for mRNA target recognition [33]. This feature, in association with a relatively well preserved microRNA seed sequence and functionality, is supportive of a potential role for microRNAs in platelet mRNA recognition and regulation.

In the present study, we analyzed the small RNA profile of resting platelets. However, Osman and Fälker [11] recently reported specific changes in the microRNA content of activated platelets, whereby 6 microRNAs were up- or down-regulated in response to a physiological agonist (thrombin). These results suggest a possible link between the activation status of platelets and their microRNA repertoire, whose alterations may induce agonist-specific, adapted changes in platelet function. In fact, the platelet microRNA profile may be modified as a consequence of the activation-induced release of microparticles containing microRNAs. Moreover, the demonstrated delivery of microRNAs from one cell to another through microvesicles [18], together with the ability of activated platelets to release microparticles [19] and the recently reported ability of platelet-like particles to mediate intercellular mRNA transfer [20], suggest that platelet microRNAs may also fulfill extraplatelet role(s). Platelet microRNAs may thus be delivered to a variety of cells in the cardiovascular system and have the potential to modulate gene expression in recipient cells. Whether platelet microRNAs exert their function predominantly in platelets or in other cells of the cardiovascular system warrants further investigations.

Considering the complexity (or plurality) of mRNA regulation by microRNAs, whereby (i) a single microRNAs may regulate hundreds of different mRNAs with varying efficiency, and (ii) each mRNA can be regulated by several microRNAs [48], the repertoire of potential platelet and extra-platelet mRNA targets of platelet microRNAs may be rather large. In fact, it may be as broad as the number of different cell types that may inherit of platelet microRNAs multiplied by the number of mRNAs expressed in these cells and potentially regulated by microRNAs. mRNA targets of platelet microRNAs can be predicted by a variety of Web-based bioinformatic tools, such as MicroCosm Targets (http://www.ebi.ac.uk/enright-srv/microcosm/htdocs/targets/v5/) [49], DIANA-microT-CDS (http://diana.cslab.ece.ntua.gr/) [50], PicTar (http://pictar.mdc-berlin.de/) [51], Miranda (www.microrna.org) [52], TargetScan (http://www.targetscan.org/) [53] and miRecords (http://mirecords.biolead.org) [54]. These tools combine different parameters of the sequence requirements for microRNA:mRNA binding and calculate the free energy of the interaction as a predictive method to identify mRNA targets with a relatively high degree of confidence. Serving as a guidance, these predictions remain to be validated experimentally which, in some cases, may represent a relatively challenging task.

In addition to provide a valuable resource to researchers interested in both the intraplatelet and extraplatelet role, function and importance of platelet microRNAs, our findings may have clinical applications, prior to which they would probably need to be expanded, by examining a larger set of subjects from different geographic location, nationality, origin, age and sex, at different periods of the year. However, the relatively strong correlation observed between our platelet microRNA data (in Quebec City) and those reported by the group of Dr. Paul F. Bray (in Philadelphia) [12] strengthens the possibility of using platelet microRNAs as universal biomarkers of platelet-related diseases. Although several issues will need to be addressed and circumvented, including that of inter-individual variations, the small RNA or microRNA profile of platelets sampled from individual subjects may eventually form the basis of personalized diagnosis and individualized therapy [55].

### Note

The novel microRNA sequences have been submitted to miRBase, which will assign names and annotate them upon acceptance of the manuscript for publication.

## Supporting Information

Figure S1 **Flowchart applied for the discovery of novel platelet microRNAs.**
(TIF)

Figure S2 **Correlation of the number of reads obtained for each microRNA sequences detected in Pool 1 and Pool 2.**
(TIF)

Figure S3 **Validation of selected novel platelet microRNAs by Northern blot hybridization.**
(TIF)

Figure S4 **Expression of 4 selected novel platelet microRNAs was confirmed by qPCR analyses.**
(TIF)

Figure S5 **Both the miR-140-3p reference sequence and its 1-nt 5′ shifted isomiR can silence Rluc reporter gene activity.**
(TIF)

Table S1 **Platelet messenger RNA (mRNA) targets of the two main isoforms of miR-140-3p.**
(TIF)

Table S2 **The 50 most abundant microRNAs detected in human platelets by high-throughput sequencing.**
(TIF)

Methods S1 (PDF)

Results S1 (PDF)

References S1 (PDF)

Database S1 (ZIP)
