# Supplementary figures and images for: The Repertoire and Features of Human Platelet microRNAs
Source: PLoS One. 2012 Dec 4;7(12):e50746. doi: 10.1371/journal.pone.0050746 (PMC3514217; doi:10.1371/journal.pone.0050746)

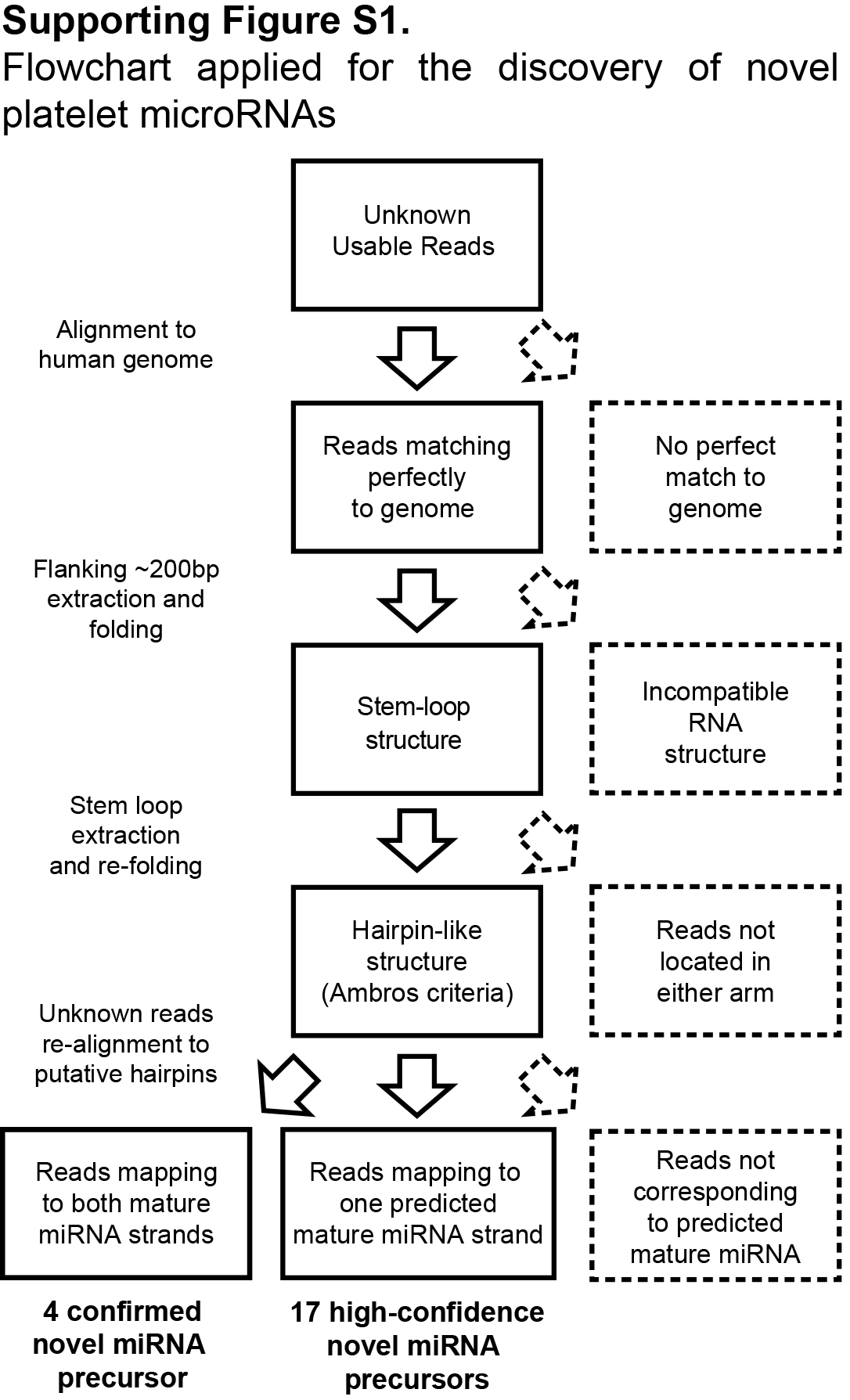

Supplement: Figure S1 — Flowchart applied for the discovery of novel platelet microRNAs. (TIF) [file pone.0050746.s001.tif]

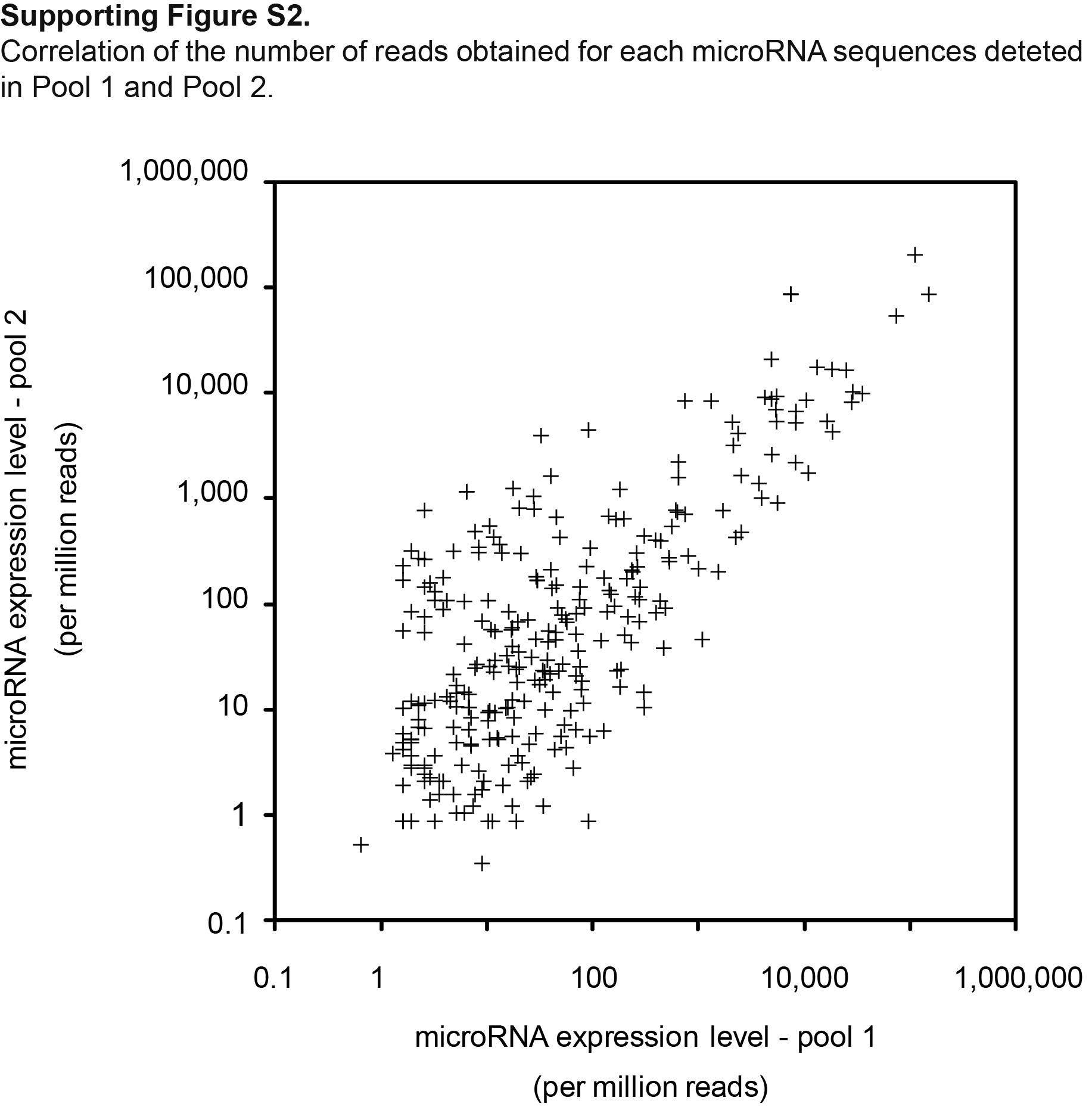

Supplement: Figure S2 — Correlation of the number of reads obtained for each microRNA sequences detected in Pool 1 and Pool 2. (TIF) [file pone.0050746.s002.tif]

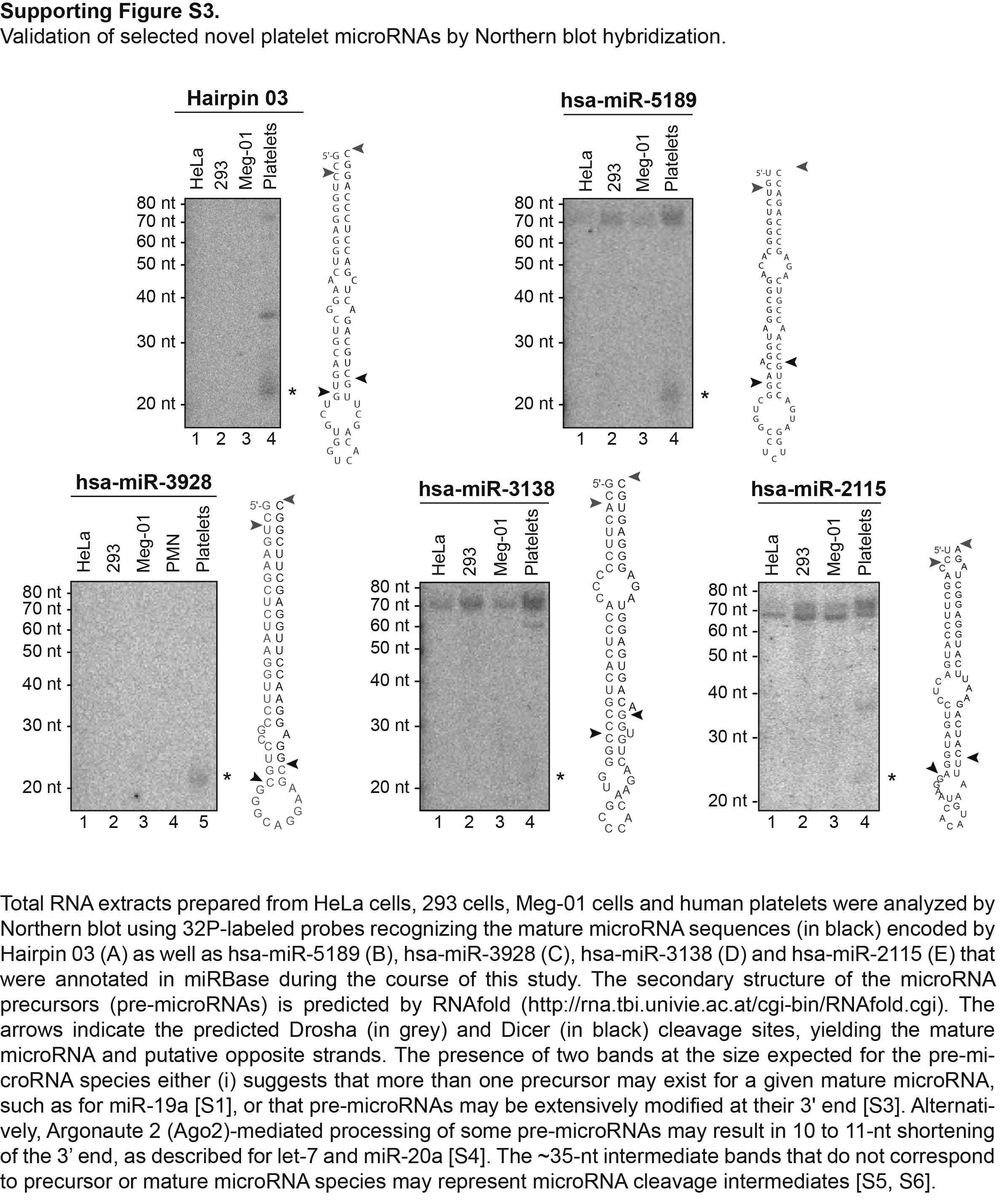

Supplement: Figure S3 — Validation of selected novel platelet microRNAs by Northern blot hybridization. (TIF) [file pone.0050746.s003.tif]

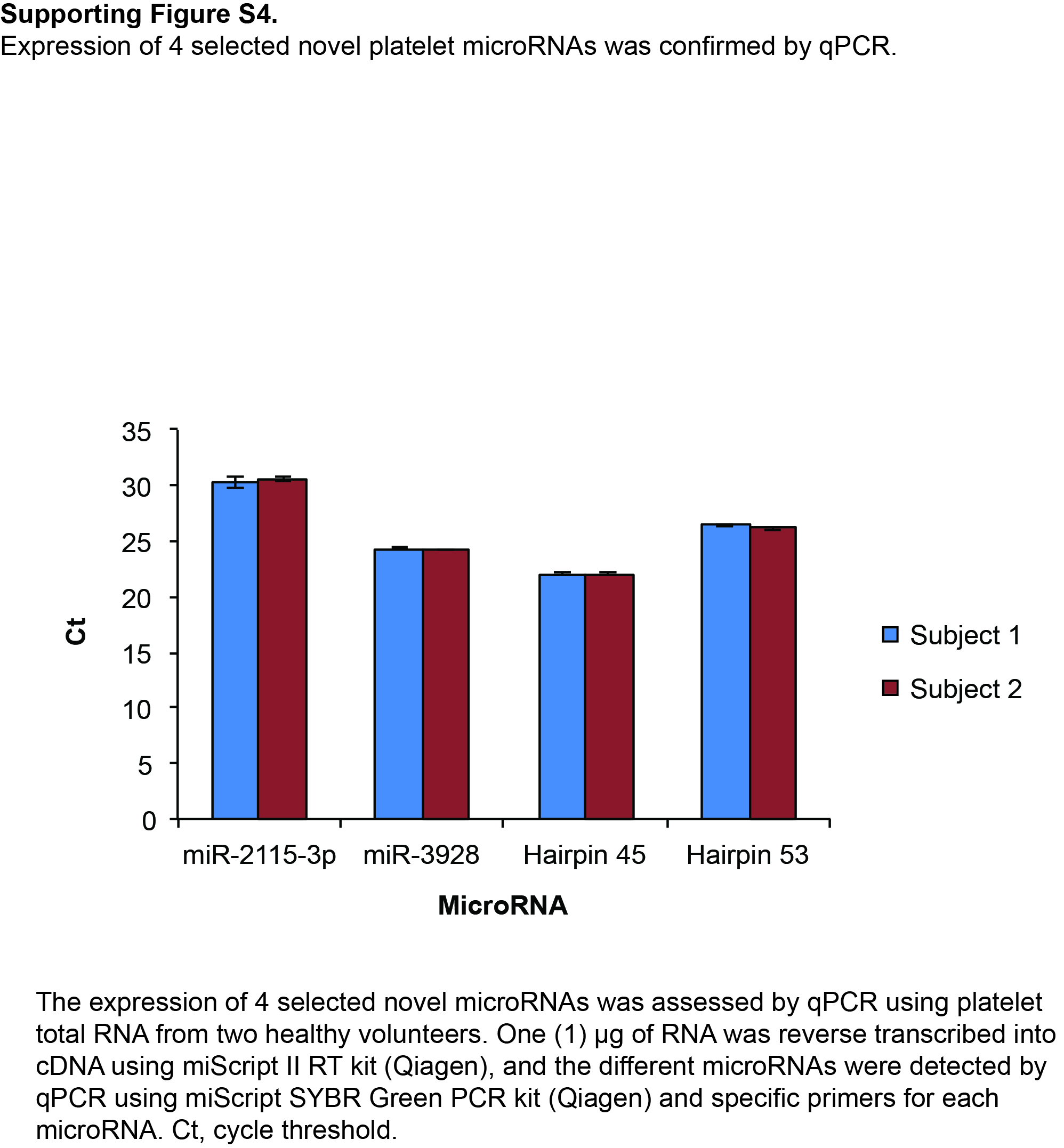

Supplement: Figure S4 — Expression of 4 selected novel platelet microRNAs was confirmed by qPCR analyses. (TIF) [file pone.0050746.s004.tif]

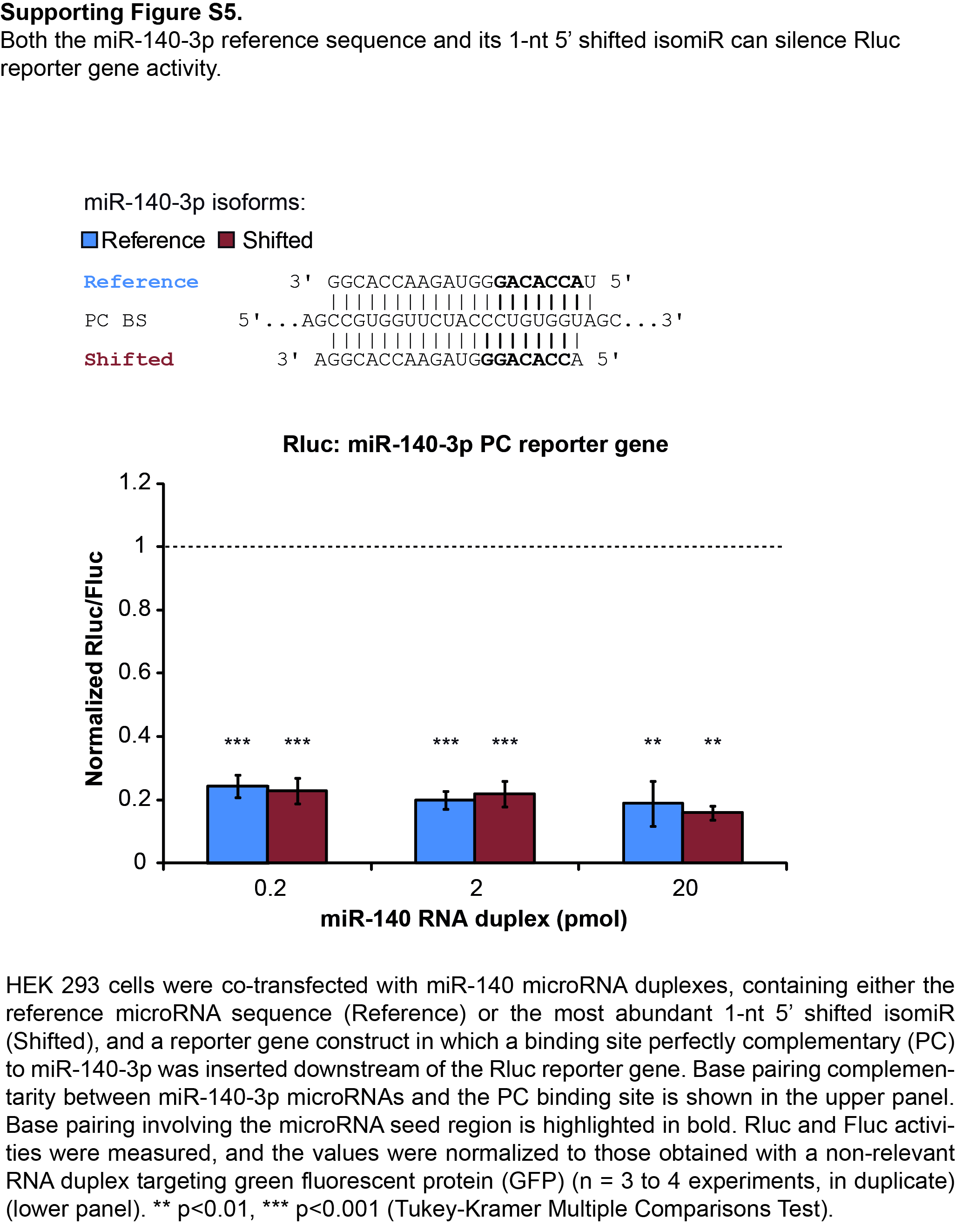

Supplement: Figure S5 — Both the miR-140-3p reference sequence and its 1-nt 5′ shifted isomiR can silence Rluc reporter gene activity. (TIF) [file pone.0050746.s005.tif]

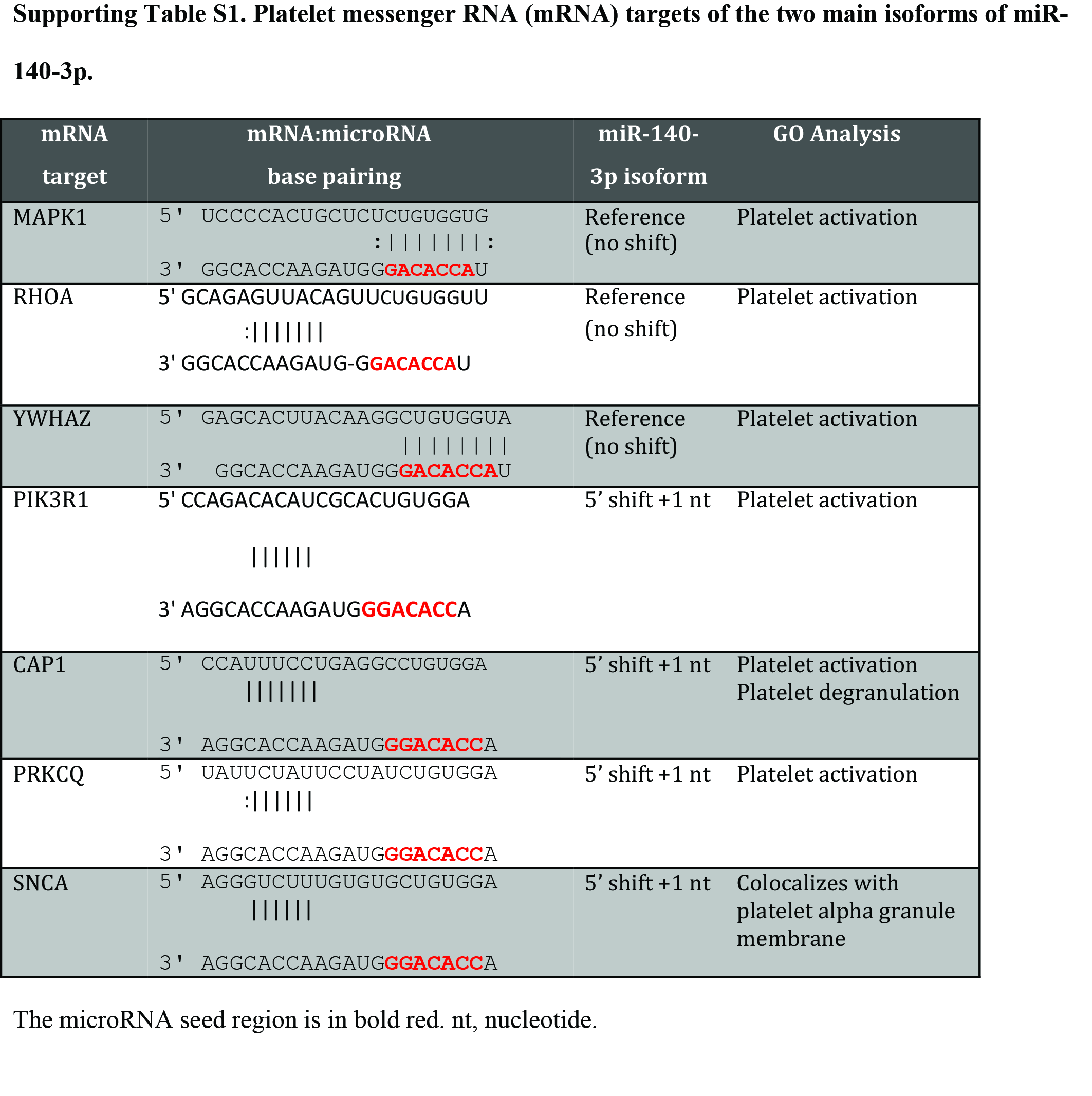

Supplement: Table S1 — Platelet messenger RNA (mRNA) targets of the two main isoforms of miR-140-3p. (TIF) [file pone.0050746.s006.tif]
