# Supplementary material for: The Repertoire and Features of Human Platelet microRNAs
Source: PLoS One. 2012 Dec 4;7(12):e50746. doi: 10.1371/journal.pone.0050746 (PMC3514217; doi:10.1371/journal.pone.0050746)
Supplement: Methods S1 — (PDF) [file pone.0050746.s008.pdf]

SUPPORTING METHODS

Northern blot analysis

HEK293, HeLa and the megakaryocytic cell line Meg-01 (ATCC) were maintained in culture, as previously described [S1]. Total RNA was extracted using TRIzol (Invitrogen). One (1) µg of total RNA was separated on a 12% (w/v) polyacrylamide gel containing 7 M urea and transferred to a nylon membrane, followed by detection using randomly ^{32}P-labeled RNA probes complementary to mature microRNA species using a Northern blot procedure improved for the detection of small RNAs [S2].

qPCR detection of novel microRNA

One (1) µg total RNA, extracted from purified platelets as described in the Materials and Methods section, was used for reverse transcription using miScript RT II kit (Qiagen). SYBR Green PCR kit (Qiagen) and specific primers for each microRNA (miScript Primer assay, Qiagen) were used to assess the expression of 4 selected novel microRNAs.
