# Supplementary material for: The Repertoire and Features of Human Platelet microRNAs
Source: PLoS One. 2012 Dec 4;7(12):e50746. doi: 10.1371/journal.pone.0050746 (PMC3514217; doi:10.1371/journal.pone.0050746)
Supplement: Results S1 — (PDF) [file pone.0050746.s009.pdf]

SUPPORTING RESULTS

Validation of five selected microRNAs by Northern blot

We elected to validate the existence of 5 of the novel microRNA candidates by Northern blot hybridization analysis of total RNA extracted from primary human platelets and 3 cultured human cell lines, using sequence-specific complementary ^{32}P-labeled probes. Four (4) of the 5 hairpins were annotated in miRBase during the course of our study, and named hsa-miR-5189, hsa-miR-3928, hsa-miR-3138 and hsa-miR-2115, respectively. Although the hybridization signal was relatively weak, which is consistent with their relatively low abundance, a ~21- or 22-nt RNA species could be detected solely in human platelets, not in HEK293, HeLa or Meg-01 cells (Supporting Figure S3). A larger RNA species, corresponding to the ~75-nt pre-microRNA, was detected in human platelets, but also in the cultured human cell lines for 3 of them. Expanding further the microRNA repertoire of human platelets, our findings raise the possibility of platelet-specific microRNA sequences, an assertion that would need to be verified more thoroughly by probing a much wider array of human tissues and cells.
