# Supplementary material for: The Repertoire and Features of Human Platelet microRNAs
Source: PLoS One. 2012 Dec 4;7(12):e50746. doi: 10.1371/journal.pone.0050746 (PMC3514217; doi:10.1371/journal.pone.0050746)
Supplement: References S1 — (PDF) [file pone.0050746.s010.pdf]

SUPPORTING REFERENCES

S1. Landry P, Plante I, Ouellet DL, Perron MP, Rousseau G, et al. (2009) Existence of a microRNA pathway in anucleate platelets. Nat Struct Mol Biol 16: 961-966.

S2. Pall GS, Codony-Servat C, Byrne J, Ritchie L, Hamilton A (2007) Carbodiimide-mediated cross-linking of RNA to nylon membranes improves the detection of siRNA, miRNA and piRNA by northern blot. Nucleic Acids Res 35: e60.

S3. Newman MA, Mani V, Hammond SM (2011) Deep sequencing of microRNA precursors reveals extensive 3' end modification. RNA 17: 1795-1803.

S4. Diederichs S, Haber DA (2007) Dual role for argonautes in microRNA processing and posttranscriptional regulation of microRNA expression. Cell 131: 1097-1108.

S5. Ouellet DL, Plante I, Landry P, Barat C, Janelle ME, et al. (2008) Identification of functional microRNAs released through asymmetrical processing of HIV-1 TAR element. Nucleic Acids Res 36: 2353-2365.

S6. Flores-Jasso CF, Arenas-Huertero C, Reyes JL, Contreras-Cubas C, Covarrubias A, et al. (2009) First step in pre-miRNAs processing by human Dicer. Acta pharmacologica Sinica 30: 1177-1185.
