# Supplementary material for: The Repertoire and Features of Human Platelet microRNAs
Source: PLoS One. 2012 Dec 4;7(12):e50746. doi: 10.1371/journal.pone.0050746 (PMC3514217; doi:10.1371/journal.pone.0050746)
Supplement: Database S1 — (ZIP) [file pone.0050746.s011.zip › Supporting Platelet microRNA sequence database S1/s4_sequence_s4run45.html]

Analysis ouput 

# s4run45

## Triming statistics

|  |  |  |  |  |  |  |  |  |  |  |  |  |  |
| --- | --- | --- | --- | --- | --- | --- | --- | --- | --- | --- | --- | --- | --- |
| Tissue | ADP5 Len. | ADP3 Len. | Total Reads | Unique Reads | Reads with adapter | Unique Reads with adapter | Length Filter(<10) | PolyN Filter(>9) | Copy Number Filter(<=4) | Usable Reads | Usable Unique Reads | Usable Percentage |  |
| s4\_sequence\_s4run45 | 0 | [TN][CN][GN][TN][AN][TN] | 3970224 | 570753 | 3445103 | 257116 | 765 | 685 | 289301 | 3154352 | 19498 | 0.794502274934613 |
| Sum(Avg) |  |  | 3970224 | 570753 | 3445103 | 257116 | 765 | 685 | 289301 | 3154352 | 19498 | 0.794502274934613 |

#### Abbreviations:

|  |  |  |
| --- | --- | --- |
| Tissue | Abbreviation. |  |
| s4\_sequence\_s4run45\_t\_f | \_ |

## Mismatch positions table

|  |  |  |  |  |  |  |  |  |  |  |  |  |  |  |  |  |  |  |  |  |  |  |  |  |  |  |  |  |  |  |  |  |  |  |  |  |  |  |
| --- | --- | --- | --- | --- | --- | --- | --- | --- | --- | --- | --- | --- | --- | --- | --- | --- | --- | --- | --- | --- | --- | --- | --- | --- | --- | --- | --- | --- | --- | --- | --- | --- | --- | --- | --- | --- | --- | --- |
| microRNA | Tissue | Exact | Loose | Pos. | Len. | MM\_Total | -2 | -1 | \_ | 1 | 2 | 3 | 4 | 5 | 6 | 7 | 8 | 9 | 10 | 11 | 12 | 13 | 14 | 15 | 16 | 17 | 18 | 19 | 20 | 21 | 22 | 23 | 24 | 25 | 26 | 27 | 28 |  |
| hsa-let-7a-3p(hsa-let-7a-1) | \_ | 160630 | 24 | 56 | 21 | 24 |  |  |  |  |  |  |  |  |  |  |  |  |  |  |  |  |  |  |  |  |  |  |  |  | 24 |  |  |  |  |  |  |
| hsa-let-7a-3p(hsa-let-7a-3) | \_ | 160033 | 24 | 51 | 21 | 24 |  |  |  |  |  |  |  |  |  |  |  |  |  |  |  |  |  |  |  |  |  |  |  |  | 24 |  |  |  |  |  |  |
| hsa-let-7a-5p(hsa-let-7a-1) | \_ | 160630 | 27267 | 5 | 22 | 29137 |  |  | 83 | 643 | 26 | 162 | 20 | 8 | 1554 | 86 | 29 | 180 | 70 | 17 | 981 | 958 | 389 | 90 | 527 | 1697 | 929 | 300 | 224 | 558 | 2710 | 16896 |  |  |  |  |  |
| hsa-let-7a-5p(hsa-let-7a-2) | \_ | 160384 | 27329 | 4 | 22 | 29171 |  | 8 | 83 | 643 | 8 | 162 | 20 | 8 | 1554 | 86 | 29 | 180 | 70 | 17 | 994 | 958 | 389 | 90 | 527 | 1697 | 929 | 300 | 224 | 558 | 2710 | 16927 |  |  |  |  |  |
| hsa-let-7a-5p(hsa-let-7a-3) | \_ | 160033 | 27267 | 3 | 22 | 29137 |  |  | 83 | 643 | 26 | 162 | 20 | 8 | 1554 | 86 | 29 | 180 | 70 | 17 | 981 | 958 | 389 | 90 | 527 | 1697 | 929 | 300 | 224 | 558 | 2710 | 16896 |  |  |  |  |  |
| hsa-let-7b-3p(hsa-let-7b) | \_ | 88148 | 28 | 59 | 22 | 28 |  |  |  |  |  |  |  |  |  |  |  |  |  |  |  |  |  |  |  |  |  |  |  | 5 | 23 |  |  |  |  |  |  |
| hsa-let-7b-5p(hsa-let-7b) | \_ | 88148 | 30751 | 5 | 22 | 36325 |  |  | 32 | 210 | 11 | 130 | 5 | 12 | 967 | 49 | 15 | 485 | 53 |  | 314 | 687 | 320 | 73 | 633 | 174 | 1181 | 3289 | 185 | 1426 | 8598 | 17459 | 13 |  | 2 |  | 2 |
| hsa-let-7c(hsa-let-7c) | \_ | 5350 | 1659 | 10 | 22 | 1797 |  |  |  | 18 |  | 23 |  |  | 76 |  |  | 28 | 16 |  | 132 | 22 | 5 |  | 28 | 168 | 236 | 313 | 61 | 17 | 139 | 515 |  |  |  |  |  |
| hsa-let-7d-3p(hsa-let-7d) | \_ | 57640 | 22 | 61 | 22 | 22 |  |  |  |  |  |  |  |  |  |  |  |  |  |  |  |  |  |  |  |  |  |  |  |  | 17 | 5 |  |  |  |  |  |
| hsa-let-7d-5p(hsa-let-7d) | \_ | 57640 | 11922 | 7 | 22 | 12915 |  |  | 7 | 1027 | 6 | 40 | 6 |  | 580 | 29 | 11 | 225 | 17 |  | 32 | 860 | 153 | 62 | 449 | 579 | 98 | 386 | 61 | 412 | 1705 | 6170 |  |  |  |  |  |
| hsa-let-7e-5p(hsa-let-7e) | \_ | 6534 | 2286 | 7 | 22 | 2601 |  |  |  | 1 |  | 32 |  |  | 166 |  |  | 428 | 7 |  | 368 | 339 | 7 |  | 51 | 98 | 80 | 46 |  | 240 | 70 | 668 |  |  |  |  |  |
| hsa-let-7f-2-3p(hsa-let-7f-2) | \_ | 183152 | 11 | 57 | 22 | 11 |  |  |  |  |  |  |  |  |  |  |  |  |  |  |  |  |  |  |  |  |  |  |  |  | 11 |  |  |  |  |  |  |
| hsa-let-7f-5p(hsa-let-7f-1) | \_ | 170445 | 24323 | 6 | 22 | 25662 |  |  | 219 | 128 | 37 | 165 | 31 | 18 | 1456 | 85 | 42 | 403 | 75 | 22 | 1345 | 127 | 361 | 106 | 571 | 614 | 245 | 333 | 235 | 505 | 2092 | 16439 | 8 |  |  |  |  |
| hsa-let-7f-5p(hsa-let-7f-2) | \_ | 183152 | 24491 | 7 | 22 | 25809 |  |  | 224 | 134 | 37 | 173 | 38 | 18 | 1559 | 90 | 42 | 438 | 75 | 22 | 983 | 125 | 382 | 111 | 604 | 637 | 262 | 356 | 251 | 530 | 2271 | 16439 | 8 |  |  |  |  |
| hsa-let-7g-5p(hsa-let-7g) | \_ | 78337 | 14504 | 4 | 22 | 14951 |  |  |  | 76 | 7 | 77 | 16 | 21 | 723 | 21 | 16 | 214 | 33 | 6 | 855 | 17 | 146 | 89 | 205 | 220 | 1245 | 302 | 77 | 238 | 981 | 9366 |  |  |  |  |  |
| hsa-let-7i-3p(hsa-let-7i) | \_ | 13289 | 12 | 61 | 22 | 12 |  |  |  |  |  |  |  |  |  |  |  |  |  |  |  |  |  |  |  |  |  |  |  |  |  | 12 |  |  |  |  |  |
| hsa-let-7i-5p(hsa-let-7i) | \_ | 13289 | 10159 | 5 | 22 | 10958 |  |  |  | 8 |  | 12 |  |  | 139 |  |  | 53 |  |  | 580 |  | 23 | 18 | 48 | 136 | 843 | 175 | 24 | 57 | 529 | 8313 |  |  |  |  |  |
| hsa-mir-1(hsa-mir-1-1) | \_ | 139 | 2 | 45 | 22 | 4 |  |  |  |  |  |  |  |  |  |  |  |  |  |  |  |  |  |  |  |  |  |  | 2 |  | 2 |  |  |  |  |  |  |
| hsa-mir-1(hsa-mir-1-2) | \_ | 145 | 2 | 52 | 22 | 4 |  |  |  |  |  |  |  |  |  |  |  |  |  |  |  |  |  |  |  |  |  |  | 2 |  | 2 |  |  |  |  |  |  |
| hsa-mir-100-5p(hsa-mir-100) | \_ | 7 | 6 | 12 | 22 | 6 |  |  |  |  |  |  |  |  |  |  |  |  |  |  |  |  |  |  |  |  |  |  |  |  |  | 6 |  |  |  |  |  |
| hsa-mir-101-3p(hsa-mir-101-1) | \_ | 12850 | 1748 | 46 | 21 | 1749 |  |  | 5 | 2 |  | 8 | 5 |  | 31 | 2 | 13 |  | 2 | 37 | 3 | 77 | 6 | 7 | 6 | 99 | 8 | 18 | 71 | 66 | 1281 | 2 |  |  |  |  |  |
| hsa-mir-101-3p(hsa-mir-101-2) | \_ | 13393 | 1748 | 48 | 21 | 1749 |  |  | 5 | 2 |  | 8 | 5 |  | 31 | 2 | 13 |  | 2 | 37 | 3 | 77 | 6 | 7 | 6 | 99 | 8 | 18 | 71 | 66 | 1281 | 2 |  |  |  |  |  |
| hsa-mir-103a-3p(hsa-mir-103a-1) | \_ | 89232 | 25937 | 47 | 23 | 28353 |  | 6 | 33 | 37 | 24 | 308 | 59 | 32 | 302 | 81 | 200 | 44 | 24 | 737 | 48 | 845 | 1025 | 180 | 67 | 229 | 1457 | 432 | 855 | 301 | 3788 | 8606 | 8625 | 2 | 6 |  |  |
| hsa-mir-103a-3p(hsa-mir-103a-2) | \_ | 94461 | 25931 | 47 | 23 | 28341 |  | 6 | 33 | 37 | 24 | 308 | 59 | 32 | 302 | 81 | 200 | 44 | 24 | 737 | 48 | 845 | 1025 | 180 | 67 | 229 | 1457 | 432 | 855 | 301 | 3788 | 8606 | 8619 | 2 |  |  |  |
| hsa-mir-106b-3p(hsa-mir-106b) | \_ | 982 | 143 | 51 | 22 | 177 |  |  |  |  |  |  |  |  |  |  |  |  |  |  |  |  |  |  |  |  |  |  |  | 9 | 123 | 45 |  |  |  |  |  |
| hsa-mir-106b-5p(hsa-mir-106b) | \_ | 982 | 99 | 11 | 21 | 99 |  |  |  |  |  |  |  |  |  |  |  |  |  |  |  |  |  | 5 |  |  |  |  |  | 38 | 56 |  |  |  |  |  |  |
| hsa-mir-107(hsa-mir-107) | \_ | 15560 | 4388 | 49 | 23 | 5130 |  | 2 | 5 |  |  | 6 | 2 |  | 6 |  | 2 |  |  | 42 |  | 40 | 42 | 1 | 1 |  | 57 | 10 | 49 | 48 | 3769 | 969 | 79 |  |  |  |  |
| hsa-mir-1250(hsa-mir-1250) | \_ | 56 | 11 | 23 | 21 | 11 |  |  |  |  |  |  |  |  |  |  |  |  |  |  |  |  |  |  |  |  |  |  |  |  | 11 |  |  |  |  |  |  |
| hsa-mir-1255b-5p(hsa-mir-1255b-1) | \_ | 6 | 2 | 2 | 22 | 2 |  |  |  |  |  |  |  |  |  |  |  |  |  |  |  |  |  |  |  |  |  |  |  |  |  | 2 |  |  |  |  |  |
| hsa-mir-1255b-5p(hsa-mir-1255b-2) | \_ | 6 | 2 | 5 | 22 | 2 |  |  |  |  |  |  |  |  |  |  |  |  |  |  |  |  |  |  |  |  |  |  |  |  |  | 2 |  |  |  |  |  |
| hsa-mir-126-3p(hsa-mir-126) | \_ | 118 | 33 | 51 | 22 | 33 |  |  |  |  |  |  |  |  |  |  |  |  |  |  |  |  |  |  |  |  |  |  |  |  |  | 33 |  |  |  |  |  |
| hsa-mir-126-5p(hsa-mir-126) | \_ | 118 | 28 | 14 | 21 | 28 |  |  |  |  |  |  |  |  |  |  |  |  |  |  |  |  |  |  |  |  |  |  |  |  | 28 |  |  |  |  |  |  |
| hsa-mir-1260a(hsa-mir-1260a) | \_ |  | 217 | 13 | 18 | 223 |  |  |  |  |  |  |  | 4 |  |  |  | 217 |  |  |  |  | 2 |  |  |  |  |  |  |  |  |  |  |  |  |  |  |
| hsa-mir-1260b(hsa-mir-1260b) | \_ | 5 | 262 | 9 | 19 | 301 |  |  |  |  |  |  |  | 4 |  |  |  | 262 |  |  |  |  | 2 |  |  |  |  | 28 | 5 |  |  |  |  |  |  |  |  |
| hsa-mir-1261(hsa-mir-1261) | \_ |  | 277 | 4 | 19 | 460 |  |  |  |  | 18 |  |  | 12 |  |  |  |  |  |  | 247 | 18 |  |  |  | 12 | 19 |  | 134 |  |  |  |  |  |  |  |  |
| hsa-mir-127-3p(hsa-mir-127) | \_ | 8 | 5 | 56 | 22 | 5 |  |  |  |  |  |  |  |  |  |  |  |  |  |  |  |  |  |  |  |  |  |  |  |  |  | 5 |  |  |  |  |  |
| hsa-mir-1273c(hsa-mir-1273c) | \_ |  | 17 | 9 | 22 | 23 |  |  |  |  |  |  |  |  |  |  |  |  |  |  |  |  |  |  |  |  |  |  |  |  | 6 | 17 |  |  |  |  |  |
| hsa-mir-1275(hsa-mir-1275) | \_ | 12 | 7 | 17 | 17 | 7 |  |  |  |  |  |  |  |  |  |  |  |  |  |  |  |  |  |  |  | 7 |  |  |  |  |  |  |  |  |  |  |  |
| hsa-mir-1278(hsa-mir-1278) | \_ | 123 | 5 | 49 | 22 | 5 |  |  |  |  |  |  |  |  |  |  |  |  |  |  |  |  |  |  |  |  |  |  |  |  |  | 5 |  |  |  |  |  |
| hsa-mir-128(hsa-mir-128-1) | \_ | 1382 | 259 | 49 | 21 | 275 |  |  |  |  |  |  |  |  |  |  |  |  |  |  |  |  |  | 19 |  |  | 3 |  |  | 55 | 198 |  |  |  |  |  |  |
| hsa-mir-128(hsa-mir-128-2) | \_ | 630 | 235 | 51 | 21 | 251 |  |  |  |  |  |  |  |  |  |  |  |  |  |  |  |  |  | 11 |  |  | 3 |  |  | 39 | 198 |  |  |  |  |  |  |
| hsa-mir-1280(hsa-mir-1280) | \_ |  | 138 | 4 | 17 | 143 |  |  | 138 |  |  |  |  |  |  |  |  |  |  |  |  |  |  |  | 5 |  |  |  |  |  |  |  |  |  |  |  |  |
| hsa-mir-1285-3p(hsa-mir-1285-1) | \_ | 3 | 5 | 50 | 22 | 5 |  |  |  |  |  |  |  |  |  |  |  |  |  |  |  |  |  |  |  |  |  |  |  | 2 |  | 3 |  |  |  |  |  |
| hsa-mir-1285-3p(hsa-mir-1285-2) | \_ | 3 | 5 | 51 | 22 | 5 |  |  |  |  |  |  |  |  |  |  |  |  |  |  |  |  |  |  |  |  |  |  |  | 2 |  | 3 |  |  |  |  |  |
| hsa-mir-1287(hsa-mir-1287) | \_ |  | 5 | 15 | 22 | 5 |  |  |  |  |  |  |  |  |  |  |  |  |  |  |  |  |  |  |  |  |  |  |  |  |  | 5 |  |  |  |  |  |
| hsa-mir-1301(hsa-mir-1301) | \_ | 53 | 16 | 47 | 24 | 16 |  |  |  |  |  |  |  |  |  |  |  |  |  |  |  |  |  |  |  |  |  |  |  |  |  |  | 16 |  |  |  |  |
| hsa-mir-1304-3p(hsa-mir-1304) | \_ | 50 | 5 | 52 | 22 | 5 |  |  |  |  |  |  |  |  |  |  |  |  |  |  |  | 5 |  |  |  |  |  |  |  |  |  |  |  |  |  |  |  |
| hsa-mir-1307-3p(hsa-mir-1307) | \_ | 703 | 409 | 79 | 22 | 471 |  |  |  |  |  |  |  |  |  |  |  |  |  |  |  |  |  | 5 |  |  | 40 | 5 |  | 20 | 85 | 316 |  |  |  |  |  |
| hsa-mir-1307-5p(hsa-mir-1307) | \_ | 703 | 22 | 40 | 21 | 27 |  |  |  |  |  |  |  |  |  |  |  |  |  |  |  |  |  |  |  |  |  |  |  | 5 | 22 |  |  |  |  |  |  |
| hsa-mir-130a-3p(hsa-mir-130a) | \_ | 87 | 28 | 54 | 22 | 33 |  |  |  |  |  |  |  |  |  |  |  |  |  |  |  |  |  |  |  |  |  |  |  |  | 10 | 23 |  |  |  |  |  |
| hsa-mir-130b-3p(hsa-mir-130b) | \_ | 135 | 82 | 50 | 22 | 82 |  |  |  |  |  |  |  |  |  |  |  |  |  |  |  |  |  |  |  |  |  |  |  |  | 35 | 47 |  |  |  |  |  |
| hsa-mir-132-3p(hsa-mir-132) | \_ | 17 | 14 | 58 | 22 | 14 |  |  |  |  |  |  |  |  |  |  |  |  |  |  |  |  |  |  |  |  |  |  |  |  |  | 14 |  |  |  |  |  |
| hsa-mir-140-3p(hsa-mir-140) | \_ | 111380 | 31930 | 61 | 21 | 33545 |  |  | 5 | 32 | 26 | 232 | 156 | 18 | 183 | 48 | 17 | 44 |  | 1067 | 36 | 10 | 275 | 193 | 1131 | 655 | 253 | 626 | 67 | 69 | 1135 | 27267 |  |  |  |  |  |
| hsa-mir-140-5p(hsa-mir-140) | \_ | 111380 | 70 | 22 | 22 | 78 |  |  |  |  |  |  |  |  |  |  |  |  |  |  |  |  |  |  |  |  |  |  |  |  | 27 | 51 |  |  |  |  |  |
| hsa-mir-142-3p(hsa-mir-142) | \_ | 16803 | 329 | 51 | 23 | 379 |  | 7 | 7 |  |  |  |  |  |  |  |  |  |  |  |  |  |  | 5 |  |  |  |  |  |  |  | 42 | 258 | 60 |  |  |  |
| hsa-mir-142-5p(hsa-mir-142) | \_ | 16803 | 1635 | 15 | 21 | 1685 |  | 34 | 242 | 151 |  |  |  |  |  |  | 65 |  |  |  |  |  |  | 103 |  | 116 | 35 |  | 560 | 93 |  | 286 |  |  |  |  |  |
| hsa-mir-143-3p(hsa-mir-143) | \_ | 8070 | 2294 | 60 | 21 | 2583 |  |  |  |  |  |  |  |  | 9 |  |  |  |  |  |  | 49 | 5 | 5 | 51 | 11 |  | 56 | 288 | 1416 | 693 |  |  |  |  |  |  |
| hsa-mir-143-5p(hsa-mir-143) | \_ | 8070 | 291 | 26 | 22 | 378 |  |  |  |  |  |  |  |  |  |  |  |  |  |  |  |  |  |  |  |  |  |  |  | 17 | 254 | 107 |  |  |  |  |  |
| hsa-mir-145-3p(hsa-mir-145) | \_ | 392 | 38 | 53 | 22 | 49 |  |  |  |  |  |  |  |  |  |  |  |  |  |  |  |  |  |  |  |  |  |  |  |  |  | 38 | 11 |  |  |  |  |
| hsa-mir-145-5p(hsa-mir-145) | \_ | 392 | 28 | 15 | 23 | 28 |  |  |  |  |  |  |  |  |  |  |  |  |  |  |  |  |  |  |  |  |  |  |  |  | 7 | 14 | 7 |  |  |  |  |
| hsa-mir-146a-5p(hsa-mir-146a) | \_ | 41 | 23 | 20 | 22 | 23 |  |  |  |  |  |  |  |  |  |  |  |  |  |  |  |  |  |  |  |  |  |  |  |  |  | 23 |  |  |  |  |  |
| hsa-mir-146b-5p(hsa-mir-146b) | \_ | 427 | 118 | 8 | 22 | 136 |  |  |  |  |  |  |  |  |  |  |  |  |  |  |  |  |  |  |  |  |  |  |  |  | 53 | 83 |  |  |  |  |  |
| hsa-mir-148a-3p(hsa-mir-148a) | \_ | 1672 | 313 | 43 | 22 | 427 |  |  |  |  |  |  |  | 25 |  |  |  |  |  |  |  |  |  |  |  | 12 |  |  |  |  | 250 | 140 |  |  |  |  |  |
| hsa-mir-148b-3p(hsa-mir-148b) | \_ | 550 | 130 | 62 | 22 | 154 |  |  |  |  |  |  |  | 5 |  |  |  |  |  |  |  |  |  |  |  |  |  |  |  |  | 52 | 97 |  |  |  |  |  |
| hsa-mir-150-3p(hsa-mir-150) | \_ | 108 | 18 | 50 | 22 | 24 |  |  |  |  |  |  |  |  |  |  |  |  |  |  |  |  |  |  |  |  |  |  |  | 5 | 13 | 6 |  |  |  |  |  |
| hsa-mir-150-5p(hsa-mir-150) | \_ | 108 | 6 | 15 | 22 | 6 |  |  |  |  |  |  |  |  |  |  |  |  |  |  |  |  |  |  |  |  |  |  |  |  |  | 6 |  |  |  |  |  |
| hsa-mir-151a-3p(hsa-mir-151a) | \_ | 106 | 8 | 46 | 21 | 8 |  |  |  |  |  |  |  |  |  |  |  |  |  |  |  |  |  |  |  |  |  |  |  |  | 8 |  |  |  |  |  |  |
| hsa-mir-152(hsa-mir-152) | \_ | 125 | 41 | 53 | 21 | 41 |  |  |  |  |  |  |  |  |  |  |  |  |  |  |  |  |  |  |  |  |  |  |  |  | 41 |  |  |  |  |  |  |
| hsa-mir-155-5p(hsa-mir-155) | \_ | 36 | 37 | 3 | 23 | 42 |  |  |  |  |  |  |  |  |  |  |  |  |  |  |  |  |  |  |  |  |  |  |  |  | 28 | 14 |  |  |  |  |  |
| hsa-mir-15a-5p(hsa-mir-15a) | \_ | 875 | 150 | 13 | 22 | 150 |  |  |  |  |  |  |  |  |  |  |  |  |  |  |  |  |  |  |  |  |  |  |  |  | 37 | 113 |  |  |  |  |  |
| hsa-mir-15b-3p(hsa-mir-15b) | \_ | 1641 | 32 | 57 | 22 | 32 |  |  |  |  |  |  |  |  |  |  |  |  |  |  |  |  |  |  |  |  |  |  |  |  |  | 32 |  |  |  |  |  |
| hsa-mir-15b-5p(hsa-mir-15b) | \_ | 1641 | 113 | 19 | 22 | 107 |  |  | 6 |  |  |  |  |  |  |  |  |  |  |  |  |  |  |  |  | 5 |  |  |  | 35 | 6 | 55 |  |  |  |  |  |
| hsa-mir-16-2-3p(hsa-mir-16-2) | \_ | 6227 | 16 | 52 | 22 | 16 |  |  |  |  |  |  |  |  |  |  |  |  |  |  |  |  |  |  |  |  |  |  |  |  | 16 |  |  |  |  |  |  |
| hsa-mir-16-5p(hsa-mir-16-1) | \_ | 6215 | 1704 | 13 | 22 | 1783 |  |  |  | 2 |  |  | 8 |  |  | 21 |  | 7 |  | 18 |  | 13 | 5 | 11 | 2 | 7 | 6 | 2 | 5 | 55 | 411 | 1210 |  |  |  |  |  |
| hsa-mir-16-5p(hsa-mir-16-2) | \_ | 6227 | 1704 | 9 | 22 | 1783 |  |  |  | 2 |  |  | 8 |  |  | 21 |  | 7 |  | 18 |  | 13 | 5 | 11 | 2 | 7 | 6 | 2 | 5 | 55 | 411 | 1210 |  |  |  |  |  |
| hsa-mir-17-3p(hsa-mir-17) | \_ | 636 | 112 | 50 | 22 | 112 |  |  |  |  |  |  |  |  |  |  |  |  |  |  |  |  |  |  |  |  |  |  |  |  |  | 112 |  |  |  |  |  |
| hsa-mir-17-5p(hsa-mir-17) | \_ | 636 | 254 | 13 | 23 | 254 |  |  |  |  |  |  |  |  |  |  |  |  |  |  |  |  |  | 6 |  |  |  |  |  | 6 |  | 196 | 46 |  |  |  |  |
| hsa-mir-181a-2-3p(hsa-mir-181a-2) | \_ | 5956 | 12 | 76 | 22 | 12 |  |  |  |  |  |  |  |  |  |  |  |  |  |  |  |  |  |  |  |  |  |  |  | 6 |  | 6 |  |  |  |  |  |
| hsa-mir-181a-3p(hsa-mir-181a-1) | \_ | 6071 | 24 | 63 | 22 | 24 |  |  |  |  |  |  |  |  |  |  |  |  |  |  |  |  |  |  |  |  |  |  |  | 17 | 7 |  |  |  |  |  |  |
| hsa-mir-181a-5p(hsa-mir-181a-1) | \_ | 6071 | 1146 | 23 | 23 | 1216 |  |  |  |  |  | 4 |  |  |  | 7 |  |  | 9 |  | 6 |  |  | 7 | 10 |  |  | 117 | 8 | 76 | 50 | 802 | 120 |  |  |  |  |
| hsa-mir-181a-5p(hsa-mir-181a-2) | \_ | 5956 | 1146 | 38 | 23 | 1216 |  |  |  |  |  | 4 |  |  |  | 7 |  |  | 9 |  | 6 |  |  | 7 | 10 |  |  | 117 | 8 | 76 | 50 | 802 | 120 |  |  |  |  |
| hsa-mir-181b-5p(hsa-mir-181b-1) | \_ | 380 | 188 | 35 | 23 | 225 |  |  |  |  |  |  |  |  |  |  |  |  |  |  |  |  |  |  |  |  |  |  |  |  | 76 | 117 | 32 |  |  |  |  |
| hsa-mir-181b-5p(hsa-mir-181b-2) | \_ | 404 | 188 | 15 | 23 | 225 |  |  |  |  |  |  |  |  |  |  |  |  |  |  |  |  |  |  |  |  |  |  |  |  | 76 | 117 | 32 |  |  |  |  |
| hsa-mir-181c-3p(hsa-mir-181c) | \_ | 471 | 165 | 64 | 22 | 183 |  |  |  |  |  |  |  |  |  |  |  |  |  |  |  |  |  |  |  |  |  |  |  |  |  | 159 | 24 |  |  |  |  |
| hsa-mir-181c-5p(hsa-mir-181c) | \_ | 471 | 12 | 26 | 22 | 12 |  |  |  |  |  |  |  |  |  |  |  |  |  |  |  |  |  |  |  |  | 7 |  |  |  |  | 5 |  |  |  |  |  |
| hsa-mir-181d(hsa-mir-181d) | \_ | 622 | 313 | 35 | 23 | 356 |  |  |  |  |  |  |  |  |  |  |  |  |  |  |  |  |  |  |  |  |  |  |  |  | 61 | 205 | 90 |  |  |  |  |
| hsa-mir-185-3p(hsa-mir-185) | \_ | 15455 | 61 | 49 | 22 | 86 |  |  |  |  |  |  |  |  |  |  |  |  |  |  |  |  |  |  |  |  |  |  |  | 5 | 56 | 25 |  |  |  |  |  |
| hsa-mir-185-5p(hsa-mir-185) | \_ | 15455 | 5368 | 14 | 22 | 5686 |  |  | 7 | 6 |  |  | 19 |  | 10 |  | 5 |  | 5 |  |  | 103 | 75 |  | 351 | 24 | 367 | 187 | 125 | 23 | 379 | 4000 |  |  |  |  |  |
| hsa-mir-186-5p(hsa-mir-186) | \_ | 1897 | 364 | 14 | 22 | 395 |  |  |  |  |  |  |  |  |  |  |  |  |  |  |  |  |  |  |  |  |  |  |  | 6 | 234 | 155 |  |  |  |  |  |
| hsa-mir-191-5p(hsa-mir-191) | \_ | 58047 | 16682 | 15 | 23 | 17795 |  | 5 | 5 | 123 | 22 | 5 | 97 | 9 | 6 | 82 | 26 | 22 | 604 | 1027 | 486 | 122 | 210 | 77 | 24 | 67 | 346 | 119 | 17 | 319 | 614 | 4845 | 8213 | 303 |  |  |  |
| hsa-mir-192-5p(hsa-mir-192) | \_ | 17507 | 1357 | 23 | 21 | 1357 |  |  |  | 26 | 6 |  |  | 27 | 9 |  |  |  |  |  |  | 18 | 43 | 14 |  | 96 | 19 | 13 | 60 | 156 | 744 | 126 |  |  |  |  |  |
| hsa-mir-193a-3p(hsa-mir-193a) | \_ | 326 | 18 | 54 | 22 | 18 |  |  |  |  |  |  |  |  |  |  |  |  |  |  |  |  |  |  |  |  |  |  |  |  | 9 | 9 |  |  |  |  |  |
| hsa-mir-193a-5p(hsa-mir-193a) | \_ | 326 | 49 | 20 | 22 | 49 |  |  |  |  |  |  |  | 6 |  |  |  |  |  |  |  |  |  |  |  |  |  |  |  |  | 14 | 29 |  |  |  |  |  |
| hsa-mir-196b-5p(hsa-mir-196b) | \_ | 109 | 171 | 14 | 22 | 171 |  |  |  |  |  |  |  |  |  |  |  |  |  |  |  |  |  |  |  |  |  |  |  |  | 122 | 49 |  |  |  |  |  |
| hsa-mir-197-3p(hsa-mir-197) | \_ | 107 | 12 | 47 | 22 | 12 |  |  |  |  |  |  |  |  |  |  |  |  |  |  |  |  |  |  |  |  |  |  |  |  | 12 |  |  |  |  |  |  |
| hsa-mir-197-5p(hsa-mir-197) | \_ | 107 | 5 | 8 | 23 | 5 |  |  |  |  |  |  |  |  |  |  |  |  |  |  |  |  |  |  |  |  |  |  |  |  | 5 |  |  |  |  |  |  |
| hsa-mir-1976(hsa-mir-1976) | \_ |  | 5 | 32 | 20 | 10 |  |  |  |  |  |  |  |  |  |  |  |  |  |  |  |  |  |  |  |  |  |  |  | 5 | 5 |  |  |  |  |  |  |
| hsa-mir-199a-3p(hsa-mir-199a-1) | \_ | 7910 | 1585 | 46 | 22 | 1656 |  |  |  |  | 1 | 1 |  | 50 |  |  | 30 | 2 |  |  | 3 | 15 | 23 | 27 | 3 | 2 |  |  | 39 | 75 | 477 | 908 |  |  |  |  |  |
| hsa-mir-199a-3p(hsa-mir-199a-2) | \_ | 7910 | 1585 | 69 | 22 | 1656 |  |  |  |  | 1 | 1 |  | 50 |  |  | 30 | 2 |  |  | 3 | 15 | 23 | 27 | 3 | 2 |  |  | 39 | 75 | 477 | 908 |  |  |  |  |  |
| hsa-mir-199a-3p(hsa-mir-199b) | \_ | 8233 | 1585 | 64 | 22 | 1656 |  |  |  |  | 1 | 1 |  | 50 |  |  | 30 | 2 |  |  | 3 | 15 | 23 | 27 | 3 | 2 |  |  | 39 | 75 | 477 | 908 |  |  |  |  |  |
| hsa-mir-199b-3p(hsa-mir-199a-1) | \_ | 7910 | 1585 | 46 | 22 | 1656 |  |  |  |  | 1 | 1 |  | 50 |  |  | 30 | 2 |  |  | 3 | 15 | 23 | 27 | 3 | 2 |  |  | 39 | 75 | 477 | 908 |  |  |  |  |  |
| hsa-mir-199b-3p(hsa-mir-199a-2) | \_ | 7910 | 1585 | 69 | 22 | 1656 |  |  |  |  | 1 | 1 |  | 50 |  |  | 30 | 2 |  |  | 3 | 15 | 23 | 27 | 3 | 2 |  |  | 39 | 75 | 477 | 908 |  |  |  |  |  |
| hsa-mir-199b-3p(hsa-mir-199b) | \_ | 8233 | 1585 | 64 | 22 | 1656 |  |  |  |  | 1 | 1 |  | 50 |  |  | 30 | 2 |  |  | 3 | 15 | 23 | 27 | 3 | 2 |  |  | 39 | 75 | 477 | 908 |  |  |  |  |  |
| hsa-mir-199b-5p(hsa-mir-199b) | \_ | 8233 | 20 | 25 | 23 | 20 |  |  |  |  |  |  |  |  |  |  |  |  |  |  |  |  |  |  |  |  |  |  |  |  |  | 15 | 5 |  |  |  |  |
| hsa-mir-19b-3p(hsa-mir-19b-1) | \_ | 55 | 7 | 53 | 23 | 7 |  |  |  |  |  |  |  |  |  |  |  |  |  |  |  |  |  |  |  |  |  |  |  |  |  | 7 |  |  |  |  |  |
| hsa-mir-19b-3p(hsa-mir-19b-2) | \_ | 55 | 7 | 61 | 23 | 7 |  |  |  |  |  |  |  |  |  |  |  |  |  |  |  |  |  |  |  |  |  |  |  |  |  | 7 |  |  |  |  |  |
| hsa-mir-200b-3p(hsa-mir-200b) | \_ | 5 | 7 | 56 | 22 | 7 |  |  |  |  |  |  |  |  |  |  |  |  |  |  |  |  |  |  |  |  |  |  |  |  |  |  | 7 |  |  |  |  |
| hsa-mir-20a-5p(hsa-mir-20a) | \_ | 126 | 68 | 7 | 23 | 68 |  |  |  |  |  |  |  |  |  |  |  |  |  |  |  |  |  |  |  |  |  |  |  |  |  | 40 | 28 |  |  |  |  |
| hsa-mir-21-5p(hsa-mir-21) | \_ | 41674 | 2600 | 7 | 22 | 2620 |  | 10 | 68 | 29 |  |  | 91 | 25 | 5 | 12 | 30 | 82 | 28 | 11 | 15 | 133 | 80 | 37 | 128 | 41 | 26 | 30 | 37 | 51 | 173 | 1394 | 84 |  |  |  |  |
| hsa-mir-2110(hsa-mir-2110) | \_ | 131 | 17 | 7 | 22 | 17 |  |  |  |  |  |  |  |  |  |  |  |  |  |  |  |  |  |  |  |  |  |  |  |  |  | 17 |  |  |  |  |  |
| hsa-mir-2115-3p(hsa-mir-2115) | \_ | 202 | 52 | 57 | 22 | 52 |  |  |  |  |  |  |  |  |  |  |  |  |  |  |  |  |  |  |  |  |  |  |  |  | 21 | 31 |  |  |  |  |  |
| hsa-mir-215(hsa-mir-215) | \_ | 5 | 28 | 26 | 21 | 28 |  |  |  | 9 |  |  |  |  |  |  |  |  |  |  |  |  |  |  |  |  |  |  | 19 |  |  |  |  |  |  |  |  |
| hsa-mir-22-3p(hsa-mir-22) | \_ | 1170 | 83 | 52 | 22 | 83 |  |  |  |  |  |  |  |  |  |  | 7 |  |  | 6 |  |  | 11 |  |  |  |  |  |  | 5 |  | 54 |  |  |  |  |  |
| hsa-mir-22-5p(hsa-mir-22) | \_ | 1170 | 30 | 14 | 22 | 30 |  |  |  |  |  |  |  |  |  |  |  |  |  |  |  |  |  |  |  |  |  |  |  |  | 24 | 6 |  |  |  |  |  |
| hsa-mir-221-3p(hsa-mir-221) | \_ | 20585 | 5962 | 64 | 23 | 7256 |  |  |  |  | 5 | 22 | 13 | 9 | 12 | 10 | 19 |  |  | 87 | 56 | 18 | 83 | 323 | 23 | 37 | 10 | 15 | 835 | 164 | 1307 | 2926 | 1282 |  |  |  |  |
| hsa-mir-221-5p(hsa-mir-221) | \_ | 20585 | 192 | 24 | 22 | 192 |  |  |  |  | 7 | 10 | 27 |  |  |  |  |  |  |  |  |  |  |  | 21 |  |  | 7 |  |  | 20 | 100 |  |  |  |  |  |
| hsa-mir-222-3p(hsa-mir-222) | \_ | 1740 | 258 | 68 | 21 | 273 |  |  |  |  |  |  |  |  |  |  |  |  |  |  |  |  |  |  |  |  |  |  |  | 59 | 214 |  |  |  |  |  |  |
| hsa-mir-223-3p(hsa-mir-223) | \_ | 51725 | 17061 | 67 | 22 | 17942 |  | 15 | 104 | 58 | 19 | 283 | 147 | 7 | 13 | 96 | 5 | 5 |  | 80 | 88 | 111 | 145 | 137 | 33 | 79 | 449 | 601 | 955 | 713 | 449 | 11256 | 2094 |  |  |  |  |
| hsa-mir-223-5p(hsa-mir-223) | \_ | 51725 | 8 | 25 | 22 | 8 |  |  |  |  |  |  |  | 8 |  |  |  |  |  |  |  |  |  |  |  |  |  |  |  |  |  |  |  |  |  |  |  |
| hsa-mir-2355-3p(hsa-mir-2355) | \_ | 293 | 5 | 53 | 22 | 5 |  |  |  |  |  |  |  |  |  |  |  |  |  |  |  |  |  |  |  |  |  | 5 |  |  |  |  |  |  |  |  |  |
| hsa-mir-2355-5p(hsa-mir-2355) | \_ | 293 | 5 | 10 | 21 | 5 |  |  |  |  |  |  | 5 |  |  |  |  |  |  |  |  |  |  |  |  |  |  |  |  |  |  |  |  |  |  |  |  |
| hsa-mir-2392(hsa-mir-2392) | \_ |  | 880 | 60 | 20 | 1760 |  |  |  |  |  |  |  |  |  |  |  |  | 330 | 550 |  |  |  | 330 | 550 |  |  |  |  |  |  |  |  |  |  |  |  |
| hsa-mir-23a-3p(hsa-mir-23a) | \_ | 34315 | 3156 | 44 | 21 | 3193 |  |  |  | 8 | 29 | 81 | 5 | 34 |  | 7 | 38 |  | 167 | 62 | 5 | 11 | 38 | 21 | 336 | 105 | 31 | 14 | 1006 | 976 | 204 | 15 |  |  |  |  |  |
| hsa-mir-23a-5p(hsa-mir-23a) | \_ | 34315 | 5 | 8 | 22 | 5 |  |  |  |  |  |  |  |  |  |  |  |  |  |  |  |  |  |  |  |  |  |  |  |  |  | 5 |  |  |  |  |  |
| hsa-mir-23b-3p(hsa-mir-23b) | \_ | 815 | 87 | 57 | 21 | 46 |  |  |  |  |  |  |  |  |  |  |  |  |  |  |  |  |  |  |  | 5 |  | 14 | 17 | 10 |  |  |  |  |  |  |  |
| hsa-mir-24-2-5p(hsa-mir-24-2) | \_ | 3561 | 8 | 12 | 22 | 8 |  |  |  |  |  |  |  |  |  |  |  |  |  |  |  |  |  |  |  |  |  |  |  |  | 8 |  |  |  |  |  |  |
| hsa-mir-24-3p(hsa-mir-24-1) | \_ | 3357 | 7092 | 43 | 22 | 7401 |  |  | 9 | 4 |  | 3 | 19 | 11 | 16 |  |  |  | 3 | 7 | 3 |  | 61 | 15 | 4 | 5 | 48 | 7 | 59 | 27 | 308 | 6781 | 11 |  |  |  |  |
| hsa-mir-24-3p(hsa-mir-24-2) | \_ | 3561 | 7068 | 49 | 22 | 7377 |  |  | 9 | 4 |  | 3 | 19 | 11 | 16 |  |  |  | 3 | 7 | 3 |  | 61 | 15 | 4 | 5 | 48 | 7 | 59 | 27 | 308 | 6757 | 11 |  |  |  |  |
| hsa-mir-25-3p(hsa-mir-25) | \_ | 90811 | 19404 | 51 | 22 | 20709 |  |  |  | 194 | 70 | 53 | 142 | 44 | 214 | 85 | 316 | 18 | 38 | 33 | 242 | 447 | 292 | 631 | 103 | 171 | 1512 | 1575 | 239 | 590 | 7777 | 5923 |  |  |  |  |  |
| hsa-mir-25-5p(hsa-mir-25) | \_ | 90811 | 28 | 13 | 21 | 28 |  |  |  |  |  |  |  |  |  |  |  |  |  |  |  |  |  |  |  |  |  |  |  |  | 28 |  |  |  |  |  |  |
| hsa-mir-26a-5p(hsa-mir-26a-1) | \_ | 8611 | 2815 | 9 | 22 | 2930 |  |  | 428 | 5 | 3 | 23 | 3 | 2 |  | 19 |  |  | 4 | 52 | 36 | 18 | 11 | 3 | 19 | 72 | 8 | 4 | 5 | 79 | 262 | 1874 |  |  |  |  |  |
| hsa-mir-26a-5p(hsa-mir-26a-2) | \_ | 8611 | 2841 | 13 | 22 | 2982 |  |  | 428 | 5 | 3 | 23 | 3 | 2 |  | 19 |  |  | 4 | 52 | 36 | 18 | 11 | 3 | 19 | 72 | 8 | 4 | 5 | 79 | 262 | 1900 | 26 |  |  |  |  |
| hsa-mir-26b-5p(hsa-mir-26b) | \_ | 32812 | 1480 | 11 | 21 | 1483 |  |  | 7 | 33 | 9 | 55 | 25 | 17 |  | 93 |  | 6 | 7 | 46 | 38 | 94 | 75 | 27 | 120 | 54 | 27 | 16 | 35 | 282 | 403 | 14 |  |  |  |  |  |
| hsa-mir-27a-3p(hsa-mir-27a) | \_ | 8005 | 991 | 50 | 21 | 1150 |  |  | 5 |  |  | 11 | 5 | 5 | 6 |  | 40 |  | 6 | 5 |  |  | 21 |  | 53 |  | 49 | 16 | 45 | 666 | 217 |  |  |  |  |  |  |
| hsa-mir-27b-3p(hsa-mir-27b) | \_ | 562 | 160 | 60 | 21 | 192 |  |  |  |  |  |  |  |  |  |  |  |  |  |  |  |  |  |  |  |  |  | 90 | 3 | 88 | 11 |  |  |  |  |  |  |
| hsa-mir-28-3p(hsa-mir-28) | \_ | 658 | 174 | 53 | 22 | 174 |  |  |  |  |  |  |  |  |  |  |  |  |  |  |  |  |  | 5 |  |  |  |  |  |  |  | 155 | 14 |  |  |  |  |
| hsa-mir-28-5p(hsa-mir-28) | \_ | 658 | 87 | 13 | 22 | 94 |  |  |  |  |  |  |  |  |  |  |  |  |  |  |  |  |  |  |  |  |  |  |  |  | 20 | 74 |  |  |  |  |  |
| hsa-mir-29a-3p(hsa-mir-29a) | \_ | 25925 | 2058 | 41 | 22 | 2089 |  |  | 11 | 16 |  | 5 | 49 | 8 | 68 | 89 |  | 12 | 3 | 23 | 7 | 97 | 66 | 41 | 35 | 68 | 6 | 5 | 286 | 18 | 347 | 829 |  |  |  |  |  |
| hsa-mir-29b-3p(hsa-mir-29b-1) | \_ | 277 | 13 | 50 | 23 | 13 |  |  |  |  |  |  |  |  |  |  |  |  |  |  |  |  |  |  |  |  |  |  |  |  |  | 9 | 4 |  |  |  |  |
| hsa-mir-29b-3p(hsa-mir-29b-2) | \_ | 297 | 13 | 51 | 23 | 13 |  |  |  |  |  |  |  |  |  |  |  |  |  |  |  |  |  |  |  |  |  |  |  |  |  | 9 | 4 |  |  |  |  |
| hsa-mir-29c-3p(hsa-mir-29c) | \_ | 1377 | 130 | 53 | 22 | 122 |  |  |  |  |  |  |  |  | 5 |  |  |  | 3 |  |  | 6 |  |  |  | 5 |  |  | 10 |  | 40 | 53 |  |  |  |  |  |
| hsa-mir-30a-3p(hsa-mir-30a) | \_ | 49 | 225 | 46 | 22 | 377 |  |  |  |  |  |  |  |  |  |  |  |  |  |  |  |  |  |  |  |  | 220 |  |  |  | 150 | 7 |  |  |  |  |  |
| hsa-mir-30a-5p(hsa-mir-30a) | \_ | 49 | 9 | 5 | 22 | 9 |  |  |  |  |  |  |  |  |  |  |  |  |  |  |  |  |  |  |  |  |  |  |  |  |  | 9 |  |  |  |  |  |
| hsa-mir-30b-3p(hsa-mir-30b) | \_ | 114 | 17 | 54 | 22 | 17 |  |  |  |  |  |  |  |  |  |  |  |  |  |  |  |  |  |  |  |  |  |  |  |  | 17 |  |  |  |  |  |  |
| hsa-mir-30b-5p(hsa-mir-30b) | \_ | 114 | 13 | 16 | 22 | 13 |  |  |  |  |  |  |  |  |  |  |  |  |  |  |  |  |  |  |  |  |  |  |  |  |  | 13 |  |  |  |  |  |
| hsa-mir-30c-1-3p(hsa-mir-30c-1) | \_ | 504 | 16 | 55 | 22 | 16 |  |  |  |  |  |  |  |  |  |  |  |  |  |  |  |  |  |  |  |  |  |  |  | 5 | 11 |  |  |  |  |  |  |
| hsa-mir-30c-5p(hsa-mir-30c-1) | \_ | 504 | 38 | 16 | 23 | 38 |  |  |  |  |  |  |  |  |  |  |  |  |  |  |  |  |  |  |  |  |  |  |  |  |  | 12 | 26 |  |  |  |  |
| hsa-mir-30c-5p(hsa-mir-30c-2) | \_ | 288 | 38 | 6 | 23 | 38 |  |  |  |  |  |  |  |  |  |  |  |  |  |  |  |  |  |  |  |  |  |  |  |  |  | 12 | 26 |  |  |  |  |
| hsa-mir-30d-5p(hsa-mir-30d) | \_ | 2014 | 1319 | 5 | 22 | 1342 |  |  |  |  |  |  |  |  |  |  |  |  |  | 12 |  | 3 |  |  |  |  |  |  |  |  | 87 | 1240 |  |  |  |  |  |
| hsa-mir-30e-3p(hsa-mir-30e) | \_ | 10146 | 9216 | 58 | 22 | 10029 |  |  |  | 49 | 73 |  |  | 24 |  |  | 79 | 5 |  |  | 11 |  | 5 | 71 |  | 5 | 21 | 217 | 41 | 133 | 5566 | 3722 | 7 |  |  |  |  |
| hsa-mir-30e-5p(hsa-mir-30e) | \_ | 10146 | 545 | 16 | 22 | 545 |  |  |  |  |  |  |  |  |  |  |  |  |  | 13 | 3 |  |  |  |  |  |  |  |  |  |  | 499 | 30 |  |  |  |  |
| hsa-mir-3135b(hsa-mir-3135b) | \_ |  | 59 | 6 | 22 | 118 |  |  |  |  |  |  |  |  |  | 59 | 59 |  |  |  |  |  |  |  |  |  |  |  |  |  |  |  |  |  |  |  |  |
| hsa-mir-3141(hsa-mir-3141) | \_ |  | 20 | 9 | 19 | 40 |  |  |  | 20 |  |  |  |  |  |  |  |  |  |  |  | 20 |  |  |  |  |  |  |  |  |  |  |  |  |  |  |  |
| hsa-mir-3154(hsa-mir-3154) | \_ | 8 | 12 | 53 | 22 | 12 |  |  |  |  |  |  |  |  |  |  |  |  |  |  |  |  |  |  |  |  |  |  |  | 7 |  | 5 |  |  |  |  |  |
| hsa-mir-3168(hsa-mir-3168) | \_ |  | 792 | 8 | 17 | 1532 |  |  | 64 |  |  |  |  |  |  |  |  |  |  |  |  |  | 792 |  | 13 | 8 | 655 |  |  |  |  |  |  |  |  |  |  |
| hsa-mir-3182(hsa-mir-3182) | \_ |  | 9 | 3 | 17 | 18 |  |  |  |  | 9 |  |  |  | 9 |  |  |  |  |  |  |  |  |  |  |  |  |  |  |  |  |  |  |  |  |  |  |
| hsa-mir-32-5p(hsa-mir-32) | \_ | 47 | 13 | 5 | 22 | 13 |  |  |  |  |  |  |  |  |  |  |  |  |  |  |  |  |  |  |  |  |  |  |  |  |  | 13 |  |  |  |  |  |
| hsa-mir-320a(hsa-mir-320a) | \_ | 16844 | 8180 | 47 | 22 | 9112 |  |  |  |  |  |  |  |  | 14 |  |  |  |  | 342 |  |  | 282 | 12 | 174 | 15 | 18 | 21 | 537 | 7 | 2500 | 4808 | 382 |  |  |  |  |
| hsa-mir-320b(hsa-mir-320b-1) | \_ | 67 | 136 | 38 | 22 | 185 |  |  |  |  |  |  |  |  |  |  |  |  |  |  |  |  |  |  |  |  |  | 9 | 63 | 7 | 60 | 46 |  |  |  |  |  |
| hsa-mir-320b(hsa-mir-320b-2) | \_ | 72 | 136 | 71 | 22 | 185 |  |  |  |  |  |  |  |  |  |  |  |  |  |  |  |  |  |  |  |  |  | 9 | 63 | 7 | 60 | 46 |  |  |  |  |  |
| hsa-mir-320c(hsa-mir-320c-1) | \_ | 5 | 69 | 49 | 20 | 78 |  |  |  |  |  |  |  |  |  |  |  |  |  |  |  |  |  |  |  |  |  | 18 | 55 | 5 |  |  |  |  |  |  |  |
| hsa-mir-320c(hsa-mir-320c-2) | \_ | 5 | 42 | 30 | 20 | 48 |  |  |  |  |  |  |  |  |  |  |  |  |  |  |  |  |  |  |  |  |  | 9 | 39 |  |  |  |  |  |  |  |  |
| hsa-mir-320d(hsa-mir-320d-1) | \_ | 2 | 11 | 29 | 19 | 11 |  |  |  |  |  |  |  |  |  |  |  |  |  |  |  |  |  |  |  |  |  |  | 11 |  |  |  |  |  |  |  |  |
| hsa-mir-320d(hsa-mir-320d-2) | \_ | 2 | 11 | 29 | 19 | 11 |  |  |  |  |  |  |  |  |  |  |  |  |  |  |  |  |  |  |  |  |  |  | 11 |  |  |  |  |  |  |  |  |
| hsa-mir-328(hsa-mir-328) | \_ | 25 | 18 | 47 | 22 | 18 |  |  |  |  |  |  |  |  |  |  |  |  |  |  |  |  |  |  |  |  |  |  |  |  |  | 18 |  |  |  |  |  |
| hsa-mir-330-3p(hsa-mir-330) | \_ | 2329 | 819 | 56 | 23 | 867 |  |  |  |  |  |  |  |  |  |  |  |  |  |  |  |  |  | 5 | 5 |  | 7 | 10 |  |  | 74 | 270 | 277 | 219 |  |  |  |
| hsa-mir-331-3p(hsa-mir-331) | \_ | 90 | 36 | 60 | 21 | 36 |  |  |  |  |  |  |  |  |  |  |  |  |  |  |  |  |  |  |  |  |  |  |  | 10 | 26 |  |  |  |  |  |  |
| hsa-mir-338-5p(hsa-mir-338) | \_ | 496 | 14 | 5 | 22 | 14 |  |  |  |  |  |  |  |  |  |  |  |  |  |  |  |  |  |  |  |  |  |  |  |  | 14 |  |  |  |  |  |  |
| hsa-mir-339-3p(hsa-mir-339) | \_ | 100 | 85 | 49 | 23 | 112 |  |  |  |  |  |  |  |  |  |  |  |  |  |  |  |  |  |  |  |  |  |  |  |  |  | 60 | 52 |  |  |  |  |
| hsa-mir-33a-5p(hsa-mir-33a) | \_ | 440 | 101 | 5 | 21 | 94 |  |  |  |  |  |  |  |  |  |  |  |  |  |  |  |  |  |  |  |  |  |  | 11 |  | 83 |  |  |  |  |  |  |
| hsa-mir-340-5p(hsa-mir-340) | \_ | 7424 | 980 | 15 | 22 | 1048 |  |  | 9 |  |  |  |  |  |  |  |  |  |  |  |  |  | 34 |  | 18 | 58 |  |  | 29 | 35 | 303 | 562 |  |  |  |  |  |
| hsa-mir-342-3p(hsa-mir-342) | \_ | 200 | 20 | 60 | 23 | 20 |  |  |  |  |  |  |  |  |  |  |  |  |  |  |  |  |  |  |  |  |  |  |  |  |  |  | 20 |  |  |  |  |
| hsa-mir-345-5p(hsa-mir-345) | \_ | 61 | 698 | 17 | 22 | 951 |  |  |  |  |  |  |  |  |  |  |  |  |  |  |  |  | 5 |  |  |  |  |  |  | 19 | 292 | 635 |  |  |  |  |  |
| hsa-mir-34c-5p(hsa-mir-34c) | \_ | 237 | 134 | 12 | 23 | 134 |  |  |  |  |  |  |  |  |  |  |  |  |  |  |  |  |  |  |  |  |  |  |  |  |  | 77 | 57 |  |  |  |  |
| hsa-mir-361-5p(hsa-mir-361) | \_ | 68 | 52 | 5 | 22 | 52 |  |  |  |  |  |  |  |  |  |  |  |  |  |  |  |  |  |  |  |  |  |  |  |  | 36 | 16 |  |  |  |  |  |
| hsa-mir-3615(hsa-mir-3615) | \_ | 50 | 5 | 50 | 21 | 10 |  |  |  |  |  |  |  |  |  |  |  |  |  |  |  |  |  |  |  |  |  |  |  | 5 | 5 |  |  |  |  |  |  |
| hsa-mir-363-3p(hsa-mir-363) | \_ | 145 | 57 | 49 | 22 | 57 |  |  |  |  |  |  |  |  |  |  |  |  |  |  |  |  |  |  |  |  |  |  |  | 7 |  | 50 |  |  |  |  |  |
| hsa-mir-3676-5p(hsa-mir-3676) | \_ | 5 | 6 | 26 | 15 | 6 |  |  |  |  |  |  |  |  |  |  |  |  |  |  |  |  |  |  | 6 |  |  |  |  |  |  |  |  |  |  |  |  |
| hsa-mir-3690(hsa-mir-3690) | \_ | 542 | 24 | 9 | 23 | 24 |  |  |  |  |  |  |  |  |  |  |  |  |  |  |  |  |  |  |  |  |  |  |  |  |  | 7 | 17 |  |  |  |  |
| hsa-mir-371b-5p(hsa-mir-371b) | \_ | 75 | 8 | 6 | 22 | 8 |  |  |  |  |  |  |  |  |  |  |  |  |  |  |  |  |  |  |  |  |  |  |  |  | 8 |  |  |  |  |  |  |
| hsa-mir-374a-3p(hsa-mir-374a) | \_ | 1509 | 82 | 41 | 22 | 82 |  |  |  |  |  |  |  |  |  |  |  |  |  |  |  |  | 5 |  |  |  |  |  |  | 11 | 10 | 56 |  |  |  |  |  |
| hsa-mir-374a-5p(hsa-mir-374a) | \_ | 1509 | 71 | 11 | 22 | 78 |  |  |  |  |  |  |  |  |  |  |  |  |  |  |  |  |  |  |  |  |  |  |  |  | 7 | 71 |  |  |  |  |  |
| hsa-mir-374b-5p(hsa-mir-374b) | \_ | 549 | 127 | 10 | 22 | 127 |  |  |  |  |  |  |  |  |  |  |  |  |  |  |  |  |  |  |  |  |  |  |  |  |  | 127 |  |  |  |  |  |
| hsa-mir-378a-3p(hsa-mir-378a) | \_ | 2491 | 2303 | 42 | 21 | 2395 |  |  |  |  |  |  |  |  |  | 5 |  |  |  |  |  |  | 28 | 34 |  |  | 21 | 5 |  | 17 | 1912 | 373 |  |  |  |  |  |
| hsa-mir-378c(hsa-mir-378c) | \_ | 373 | 94 | 10 | 25 | 89 |  |  |  |  |  |  |  |  |  |  |  |  |  |  |  |  |  |  |  |  |  |  |  |  |  |  | 89 |  |  |  |  |
| hsa-mir-378f(hsa-mir-378f) | \_ |  | 17 | 51 | 20 | 17 |  |  |  |  |  |  |  |  |  |  |  |  |  |  |  |  | 17 |  |  |  |  |  |  |  |  |  |  |  |  |  |  |
| hsa-mir-378i(hsa-mir-378i) | \_ |  | 126 | 6 | 21 | 134 |  |  |  |  |  |  |  |  |  |  |  | 126 |  |  |  |  |  | 4 |  |  |  |  |  |  |  | 4 |  |  |  |  |  |
| hsa-mir-3928(hsa-mir-3928) | \_ | 13 | 141 | 36 | 22 | 163 |  |  |  |  |  |  |  |  |  |  |  |  |  |  |  |  |  |  |  |  |  |  |  |  | 33 | 119 | 11 |  |  |  |  |
| hsa-mir-421(hsa-mir-421) | \_ | 236 | 89 | 47 | 23 | 89 |  |  |  |  |  |  |  |  |  |  |  |  |  |  |  |  |  |  |  |  |  |  |  |  |  | 67 | 22 |  |  |  |  |
| hsa-mir-423-3p(hsa-mir-423) | \_ | 28070 | 787 | 52 | 23 | 888 |  |  |  |  |  |  |  |  |  |  | 16 |  |  |  |  |  |  | 18 | 6 | 6 | 14 |  | 70 | 38 | 131 | 219 | 370 |  |  |  |  |
| hsa-mir-423-5p(hsa-mir-423) | \_ | 28070 | 5311 | 16 | 23 | 6086 |  |  |  | 17 |  | 6 |  |  |  |  | 27 |  |  | 5 |  | 137 | 22 | 368 | 12 | 115 | 5 | 254 | 361 | 198 | 508 | 2384 | 1649 | 18 |  |  |  |
| hsa-mir-424-3p(hsa-mir-424) | \_ | 1812 | 134 | 47 | 21 | 134 |  |  |  |  |  |  |  |  |  |  |  |  |  |  |  |  |  |  |  |  |  |  |  | 18 | 59 | 57 |  |  |  |  |  |
| hsa-mir-424-5p(hsa-mir-424) | \_ | 1812 | 42 | 10 | 22 | 42 |  |  |  |  |  |  |  |  |  |  |  |  |  |  |  |  |  |  |  |  |  |  |  |  |  | 42 |  |  |  |  |  |
| hsa-mir-425-3p(hsa-mir-425) | \_ | 2241 | 299 | 54 | 22 | 340 |  |  |  |  |  |  |  |  |  |  |  |  |  |  |  |  | 5 |  |  | 7 |  |  | 11 | 126 | 157 | 34 |  |  |  |  |  |
| hsa-mir-425-5p(hsa-mir-425) | \_ | 2241 | 179 | 13 | 23 | 179 |  |  |  |  |  |  |  |  |  |  |  |  |  |  |  |  |  |  |  | 7 |  |  | 12 |  | 7 | 73 | 70 | 10 |  |  |  |
| hsa-mir-4286(hsa-mir-4286) | \_ | 654 | 37 | 10 | 17 | 37 |  |  |  |  |  | 8 | 8 |  |  |  |  |  |  |  |  |  | 14 |  |  |  | 7 |  |  |  |  |  |  |  |  |  |  |
| hsa-mir-432-5p(hsa-mir-432) | \_ | 26 | 20 | 13 | 23 | 20 |  |  |  |  |  |  |  |  |  |  |  |  |  |  |  |  |  |  |  |  |  |  |  |  |  | 20 |  |  |  |  |  |
| hsa-mir-4433-3p(hsa-mir-4433) | \_ | 168 | 27 | 50 | 21 | 27 |  |  |  |  |  |  |  |  |  |  |  |  |  |  |  |  |  |  |  |  | 13 |  |  |  | 14 |  |  |  |  |  |  |
| hsa-mir-4443(hsa-mir-4443) | \_ | 54 | 74 | 8 | 17 | 105 |  | 26 | 66 | 13 |  |  |  |  |  |  |  |  |  |  |  |  |  |  |  |  |  |  |  |  |  |  |  |  |  |  |  |
| hsa-mir-4448(hsa-mir-4448) | \_ |  | 13 | 60 | 20 | 20 |  |  |  |  |  |  |  |  | 13 |  |  |  |  |  |  |  |  |  |  |  |  |  |  | 7 |  |  |  |  |  |  |  |
| hsa-mir-4486(hsa-mir-4486) | \_ |  | 6 | 4 | 17 | 6 |  |  | 6 |  |  |  |  |  |  |  |  |  |  |  |  |  |  |  |  |  |  |  |  |  |  |  |  |  |  |  |  |
| hsa-mir-4508(hsa-mir-4508) | \_ | 8 | 281 | 8 | 17 | 281 |  | 281 |  |  |  |  |  |  |  |  |  |  |  |  |  |  |  |  |  |  |  |  |  |  |  |  |  |  |  |  |  |
| hsa-mir-450a-5p(hsa-mir-450a-1) | \_ | 84 | 2 | 17 | 22 | 2 |  |  |  |  |  |  |  |  |  |  |  |  |  |  |  |  |  |  |  |  |  |  |  |  | 2 |  |  |  |  |  |  |
| hsa-mir-450a-5p(hsa-mir-450a-2) | \_ | 127 | 2 | 22 | 22 | 2 |  |  |  |  |  |  |  |  |  |  |  |  |  |  |  |  |  |  |  |  |  |  |  |  | 2 |  |  |  |  |  |  |
| hsa-mir-450b-5p(hsa-mir-450b) | \_ | 81 | 27 | 10 | 22 | 27 |  |  |  |  |  |  |  |  |  |  |  |  |  |  |  |  |  |  |  |  |  |  |  |  |  | 27 |  |  |  |  |  |
| hsa-mir-4510(hsa-mir-4510) | \_ |  | 282 | 7 | 22 | 409 |  |  |  |  |  |  |  |  |  |  |  |  |  |  | 3 | 125 |  |  | 7 | 43 |  | 196 |  | 9 |  | 26 |  |  |  |  |  |
| hsa-mir-4531(hsa-mir-4531) | \_ |  | 7 | 27 | 17 | 7 |  |  |  |  |  |  |  |  | 7 |  |  |  |  |  |  |  |  |  |  |  |  |  |  |  |  |  |  |  |  |  |  |
| hsa-mir-4651(hsa-mir-4651) | \_ |  | 8 | 9 | 20 | 8 |  |  |  |  |  |  |  |  |  |  |  |  |  |  |  |  |  |  |  |  |  |  |  | 8 |  |  |  |  |  |  |  |
| hsa-mir-4772-3p(hsa-mir-4772) | \_ | 66 | 20 | 47 | 22 | 20 |  |  |  |  |  |  |  |  |  |  |  |  |  |  |  |  |  |  |  |  |  |  |  |  |  | 20 |  |  |  |  |  |
| hsa-mir-4772-5p(hsa-mir-4772) | \_ | 66 | 10 | 11 | 22 | 10 |  |  |  |  |  |  |  |  |  |  |  |  |  |  |  |  |  |  |  |  |  |  |  | 5 |  | 5 |  |  |  |  |  |
| hsa-mir-484(hsa-mir-484) | \_ | 54 | 7 | 7 | 22 | 7 |  |  |  |  |  |  |  |  |  |  |  |  |  |  |  |  |  |  |  |  |  |  |  |  |  | 7 |  |  |  |  |  |
| hsa-mir-486-3p(hsa-mir-486) | \_ | 69 | 9 | 45 | 21 | 9 |  |  |  |  |  |  |  |  |  |  |  |  |  |  |  |  |  |  |  |  |  |  |  |  | 9 |  |  |  |  |  |  |
| hsa-mir-486-5p(hsa-mir-486) | \_ | 69 | 15 | 3 | 22 | 15 |  |  |  |  |  |  |  |  |  |  |  |  |  |  |  |  |  |  |  |  |  |  |  |  |  | 15 |  |  |  |  |  |
| hsa-mir-502-3p(hsa-mir-502) | \_ | 110 | 9 | 51 | 22 | 9 |  |  |  |  |  |  |  |  |  |  |  |  |  |  |  |  |  |  |  |  |  |  |  |  |  | 9 |  |  |  |  |  |
| hsa-mir-503(hsa-mir-503) | \_ | 19 | 114 | 5 | 23 | 194 |  |  |  |  |  |  |  |  |  |  |  |  |  |  |  |  |  |  |  |  |  |  | 29 | 114 |  | 51 |  |  |  |  |  |
| hsa-mir-504(hsa-mir-504) | \_ | 87 | 25 | 12 | 22 | 25 |  |  |  |  |  |  |  |  |  |  |  |  |  |  |  |  |  |  |  |  |  |  |  | 5 |  | 20 |  |  |  |  |  |
| hsa-mir-505-5p(hsa-mir-505) | \_ | 295 | 8 | 14 | 22 | 8 |  |  |  |  |  |  |  |  |  |  |  |  |  |  |  |  |  |  |  |  |  |  |  |  |  | 8 |  |  |  |  |  |
| hsa-mir-532-5p(hsa-mir-532) | \_ | 883 | 115 | 19 | 22 | 115 |  |  |  |  |  |  |  |  |  |  |  |  |  |  |  |  |  |  |  |  |  | 6 |  |  | 6 | 103 |  |  |  |  |  |
| hsa-mir-542-3p(hsa-mir-542) | \_ | 252 | 25 | 52 | 22 | 25 |  |  |  |  |  |  |  |  |  |  |  |  |  |  |  |  |  |  |  |  |  |  |  |  |  | 25 |  |  |  |  |  |
| hsa-mir-548d-5p(hsa-mir-548d-1) | \_ | 14 | 9 | 24 | 22 | 9 |  |  |  |  |  |  |  |  |  |  | 9 |  |  |  |  |  |  |  |  |  |  |  |  |  |  |  |  |  |  |  |  |
| hsa-mir-548d-5p(hsa-mir-548d-2) | \_ | 14 | 9 | 24 | 22 | 9 |  |  |  |  |  |  |  |  |  |  | 9 |  |  |  |  |  |  |  |  |  |  |  |  |  |  |  |  |  |  |  |  |
| hsa-mir-548e(hsa-mir-548e) | \_ | 588 | 114 | 52 | 22 | 114 |  |  |  |  |  |  |  |  |  |  |  |  |  |  |  |  |  |  |  |  |  |  |  |  |  | 114 |  |  |  |  |  |
| hsa-mir-548t-5p(hsa-mir-548t) | \_ | 10 | 7 | 9 | 21 | 7 |  |  |  |  |  |  |  |  |  |  |  |  |  |  |  |  |  |  |  |  |  |  |  |  | 7 |  |  |  |  |  |  |
| hsa-mir-550a-5p(hsa-mir-550a-1) | \_ |  | 12 | 21 | 23 | 18 |  |  |  |  |  |  |  |  |  |  |  |  |  |  |  |  |  |  |  |  |  |  |  |  | 12 | 6 |  |  |  |  |  |
| hsa-mir-550a-5p(hsa-mir-550a-2) | \_ |  | 12 | 21 | 23 | 18 |  |  |  |  |  |  |  |  |  |  |  |  |  |  |  |  |  |  |  |  |  |  |  |  | 12 | 6 |  |  |  |  |  |
| hsa-mir-574-3p(hsa-mir-574) | \_ | 82 | 28 | 60 | 22 | 28 |  |  |  |  |  |  |  |  |  |  |  |  |  |  |  |  |  |  |  |  |  |  |  |  | 7 | 21 |  |  |  |  |  |
| hsa-mir-576-3p(hsa-mir-576) | \_ | 151 | 6 | 54 | 22 | 6 |  |  |  |  |  |  |  |  |  |  |  |  |  |  |  |  |  |  |  |  |  |  |  |  |  | 6 |  |  |  |  |  |
| hsa-mir-582-3p(hsa-mir-582) | \_ | 214 | 24 | 52 | 22 | 36 |  |  |  |  |  |  |  |  |  |  |  |  |  |  |  |  |  |  |  |  |  |  |  | 12 | 12 | 12 |  |  |  |  |  |
| hsa-mir-582-5p(hsa-mir-582) | \_ | 214 | 8 | 15 | 23 | 8 |  |  |  |  |  |  |  |  |  |  |  |  |  |  |  |  |  |  |  |  |  |  |  |  |  | 8 |  |  |  |  |  |
| hsa-mir-589-5p(hsa-mir-589) | \_ | 48 | 21 | 23 | 22 | 27 |  |  |  |  |  |  |  |  |  |  |  |  |  |  |  |  |  |  |  |  |  |  |  |  | 16 | 11 |  |  |  |  |  |
| hsa-mir-598(hsa-mir-598) | \_ | 152 | 9 | 60 | 22 | 9 |  |  |  |  |  |  |  |  |  |  |  |  |  |  |  |  |  |  |  |  |  |  |  |  |  | 9 |  |  |  |  |  |
| hsa-mir-618(hsa-mir-618) | \_ | 60 | 6 | 15 | 23 | 6 |  |  |  |  |  |  |  |  |  |  |  |  |  |  |  |  |  |  |  |  |  |  |  |  |  | 6 |  |  |  |  |  |
| hsa-mir-628-5p(hsa-mir-628) | \_ | 165 | 33 | 22 | 22 | 33 |  |  |  |  |  |  |  |  |  |  |  |  |  |  |  |  |  |  |  |  |  |  |  |  |  | 33 |  |  |  |  |  |
| hsa-mir-629-5p(hsa-mir-629) | \_ | 145 | 5 | 21 | 21 | 5 |  |  |  |  |  |  |  |  |  |  |  |  |  |  |  |  |  |  |  |  |  |  | 5 |  |  |  |  |  |  |  |  |
| hsa-mir-642a-3p(hsa-mir-642a) | \_ | 10 | 8 | 50 | 22 | 8 |  |  |  |  |  |  |  |  |  |  |  |  |  |  |  |  |  |  |  |  |  |  |  |  | 8 |  |  |  |  |  |  |
| hsa-mir-651(hsa-mir-651) | \_ | 16 | 12 | 15 | 22 | 12 |  |  |  |  |  |  |  |  |  |  |  |  |  |  |  |  |  |  |  |  |  |  |  |  |  | 12 |  |  |  |  |  |
| hsa-mir-652-3p(hsa-mir-652) | \_ | 232 | 425 | 60 | 21 | 425 |  |  |  |  |  |  |  |  |  |  |  |  |  |  |  |  |  |  |  |  |  |  |  | 6 | 419 |  |  |  |  |  |  |
| hsa-mir-664-5p(hsa-mir-664) | \_ | 785 | 32 | 10 | 24 | 32 |  |  |  |  |  |  |  |  |  |  |  |  |  |  |  |  |  |  |  |  |  |  |  |  |  | 10 | 22 |  |  |  |  |
| hsa-mir-7-1-3p(hsa-mir-7-1) | \_ | 138 | 26 | 65 | 22 | 26 |  |  |  |  |  |  |  |  |  |  |  |  |  |  |  |  |  |  |  |  |  |  |  |  | 8 | 18 |  |  |  |  |  |
| hsa-mir-720(hsa-mir-720) | \_ | 447 | 43 | 26 | 17 | 43 |  |  |  | 34 |  |  |  |  |  |  |  |  |  |  |  |  |  | 9 |  |  |  |  |  |  |  |  |  |  |  |  |  |
| hsa-mir-744-5p(hsa-mir-744) | \_ | 2313 | 1361 | 10 | 22 | 1394 |  |  |  |  |  |  |  |  |  |  |  |  |  |  |  |  | 16 |  |  |  | 12 |  | 5 | 194 | 46 | 1116 | 5 |  |  |  |  |
| hsa-mir-766-3p(hsa-mir-766) | \_ | 33 | 14 | 64 | 22 | 14 |  |  |  |  |  |  |  |  |  |  |  |  |  |  |  |  |  |  |  |  |  |  |  |  | 5 | 9 |  |  |  |  |  |
| hsa-mir-766-5p(hsa-mir-766) | \_ | 33 | 6 | 28 | 22 | 6 |  |  |  |  |  |  |  |  |  |  |  |  |  |  |  |  |  |  |  |  |  |  |  |  |  | 6 |  |  |  |  |  |
| hsa-mir-769-5p(hsa-mir-769) | \_ | 15 | 15 | 29 | 22 | 21 |  |  |  |  |  |  |  |  |  |  |  |  |  |  |  |  |  |  |  |  |  |  |  |  | 15 | 6 |  |  |  |  |  |
| hsa-mir-92a-1-5p(hsa-mir-92a-1) | \_ | 2277 | 18 | 10 | 23 | 18 |  |  |  |  |  |  |  |  |  |  |  |  |  |  |  |  |  |  |  |  |  |  |  |  | 10 | 8 |  |  |  |  |  |
| hsa-mir-92a-3p(hsa-mir-92a-1) | \_ | 2277 | 1189 | 47 | 22 | 1302 |  |  | 3 | 39 |  |  |  |  | 7 |  | 8 |  |  |  |  | 4 | 74 | 27 | 2 |  | 13 | 11 |  | 19 | 118 | 943 | 34 |  |  |  |  |
| hsa-mir-92a-3p(hsa-mir-92a-2) | \_ | 1903 | 1121 | 47 | 22 | 1234 |  |  | 3 | 39 |  |  |  |  | 7 |  | 8 |  |  |  |  | 4 | 68 | 27 | 2 |  | 13 | 11 |  | 19 | 100 | 912 | 21 |  |  |  |  |
| hsa-mir-92b-3p(hsa-mir-92b) | \_ | 10 | 5 | 60 | 22 | 10 |  |  |  |  |  |  |  |  |  |  |  |  |  |  |  |  |  |  |  |  |  |  |  |  | 5 | 5 |  |  |  |  |  |
| hsa-mir-93-3p(hsa-mir-93) | \_ | 4842 | 7 | 49 | 22 | 7 |  |  |  |  |  |  |  |  |  |  |  |  |  |  |  |  |  |  |  |  |  |  |  |  |  | 7 |  |  |  |  |  |
| hsa-mir-93-5p(hsa-mir-93) | \_ | 4842 | 1945 | 10 | 23 | 1997 |  |  |  |  |  |  |  |  | 25 |  | 7 |  |  | 8 |  | 12 | 5 | 20 |  | 31 | 19 |  | 5 | 100 | 29 | 995 | 741 |  |  |  |  |
| hsa-mir-941(hsa-mir-941-1) | \_ | 384 | 253 | 70 | 23 | 264 |  |  |  |  |  |  |  |  |  |  |  |  |  |  |  | 4 |  |  |  | 1 | 1 |  |  | 2 | 3 | 205 | 48 |  |  |  |  |
| hsa-mir-941(hsa-mir-941-2) | \_ | 370 | 253 | 14 | 23 | 264 |  |  |  |  |  |  |  |  |  |  |  |  |  |  |  | 4 |  |  |  | 1 | 1 |  |  | 2 | 3 | 205 | 48 |  |  |  |  |
| hsa-mir-941(hsa-mir-941-3) | \_ | 370 | 253 | 14 | 23 | 264 |  |  |  |  |  |  |  |  |  |  |  |  |  |  |  | 4 |  |  |  | 1 | 1 |  |  | 2 | 3 | 205 | 48 |  |  |  |  |
| hsa-mir-941(hsa-mir-941-4) | \_ | 370 | 253 | 14 | 23 | 264 |  |  |  |  |  |  |  |  |  |  |  |  |  |  |  | 4 |  |  |  | 1 | 1 |  |  | 2 | 3 | 205 | 48 |  |  |  |  |
| hsa-mir-98(hsa-mir-98) | \_ | 1221 | 322 | 21 | 22 | 373 |  |  |  | 1 |  | 1 |  |  | 4 |  |  |  |  | 230 | 5 | 2 |  |  | 2 | 8 | 3 | 5 |  |  | 21 | 91 |  |  |  |  |  |
| hsa-mir-99a-5p(hsa-mir-99a) | \_ | 33 | 10 | 12 | 22 | 10 |  |  |  |  |  |  |  |  |  |  |  |  |  |  |  |  |  |  |  |  |  |  |  |  |  | 10 |  |  |  |  |  |
| hsa-mir-99b-3p(hsa-mir-99b) | \_ | 317 | 9 | 44 | 22 | 9 |  |  |  |  |  |  |  |  |  |  |  |  |  |  |  |  |  |  |  |  |  |  |  |  |  | 9 |  |  |  |  |  |
| hsa-mir-99b-5p(hsa-mir-99b) | \_ | 317 | 93 | 6 | 22 | 93 |  |  |  |  |  |  |  |  |  |  |  |  |  |  |  |  |  |  |  |  |  |  |  |  |  | 93 |  |  |  |  |  |
| Sum(Avg) |  | 3165096 | 460166 | 8267 | 5601 | 497721 |  | 400 | 2442 | 4528 | 511 | 2614 | 1135 | 774 | 11658 | 1378 | 1611 | 3727 | 1761 | 5452 | 9017 | 8652 | 7755 | 3928 | 7616 | 10430 | 14335 | 11473 | 9960 | 13941 | 73529 | 254459 | 34011 | 614 | 8 |  | 2 |

## Detected matches

|  |  |  |  |  |
| --- | --- | --- | --- | --- |
| microRNA | Exact | Loose | Total |  |
| hsa-let-7a-2-3p(hsa-let-7a-2) | 160384 | 0 | 160384 |
| hsa-let-7a-3p(hsa-let-7a-1) | 160630 | 24 | 160654 |
| hsa-let-7a-3p(hsa-let-7a-3) | 160033 | 24 | 160057 |
| hsa-let-7a-5p(hsa-let-7a-1) | 160630 | 27267 | 187897 |
| hsa-let-7a-5p(hsa-let-7a-2) | 160384 | 27329 | 187713 |
| hsa-let-7a-5p(hsa-let-7a-3) | 160033 | 27267 | 187300 |
| hsa-let-7b-3p(hsa-let-7b) | 88148 | 28 | 88176 |
| hsa-let-7b-5p(hsa-let-7b) | 88148 | 30751 | 118899 |
| hsa-let-7c(hsa-let-7c) | 5350 | 1659 | 7009 |
| hsa-let-7d-3p(hsa-let-7d) | 57640 | 22 | 57662 |
| hsa-let-7d-5p(hsa-let-7d) | 57640 | 11922 | 69562 |
| hsa-let-7e-3p(hsa-let-7e) | 6534 | 0 | 6534 |
| hsa-let-7e-5p(hsa-let-7e) | 6534 | 2286 | 8820 |
| hsa-let-7f-1-3p(hsa-let-7f-1) | 170445 | 0 | 170445 |
| hsa-let-7f-2-3p(hsa-let-7f-2) | 183152 | 11 | 183163 |
| hsa-let-7f-5p(hsa-let-7f-1) | 170445 | 24323 | 194768 |
| hsa-let-7f-5p(hsa-let-7f-2) | 183152 | 24491 | 207643 |
| hsa-let-7g-3p(hsa-let-7g) | 78337 | 0 | 78337 |
| hsa-let-7g-5p(hsa-let-7g) | 78337 | 14504 | 92841 |
| hsa-let-7i-3p(hsa-let-7i) | 13289 | 12 | 13301 |
| hsa-let-7i-5p(hsa-let-7i) | 13289 | 10159 | 23448 |
| hsa-mir-1(hsa-mir-1-1) | 139 | 2 | 141 |
| hsa-mir-1(hsa-mir-1-2) | 145 | 2 | 147 |
| hsa-mir-100-3p(hsa-mir-100) | 7 | 0 | 7 |
| hsa-mir-100-5p(hsa-mir-100) | 7 | 6 | 13 |
| hsa-mir-101-3p(hsa-mir-101-1) | 12850 | 1748 | 14598 |
| hsa-mir-101-3p(hsa-mir-101-2) | 13393 | 1748 | 15141 |
| hsa-mir-101-5p(hsa-mir-101-1) | 12850 | 0 | 12850 |
| hsa-mir-103a-2-5p(hsa-mir-103a-2) | 94461 | 0 | 94461 |
| hsa-mir-103a-3p(hsa-mir-103a-1) | 89232 | 25937 | 115169 |
| hsa-mir-103a-3p(hsa-mir-103a-2) | 94461 | 25931 | 120392 |
| hsa-mir-106b-3p(hsa-mir-106b) | 982 | 143 | 1125 |
| hsa-mir-106b-5p(hsa-mir-106b) | 982 | 99 | 1081 |
| hsa-mir-107(hsa-mir-107) | 15560 | 4388 | 19948 |
| hsa-mir-10a-3p(hsa-mir-10a) | 32 | 0 | 32 |
| hsa-mir-10a-5p(hsa-mir-10a) | 32 | 0 | 32 |
| hsa-mir-1250(hsa-mir-1250) | 56 | 11 | 67 |
| hsa-mir-1255a(hsa-mir-1255a) | 193 | 0 | 193 |
| hsa-mir-1255b-2-3p(hsa-mir-1255b-2) | 6 | 0 | 6 |
| hsa-mir-1255b-5p(hsa-mir-1255b-1) | 6 | 2 | 8 |
| hsa-mir-1255b-5p(hsa-mir-1255b-2) | 6 | 2 | 8 |
| hsa-mir-1256(hsa-mir-1256) | 16 | 0 | 16 |
| hsa-mir-125a-3p(hsa-mir-125a) | 80 | 0 | 80 |
| hsa-mir-125a-5p(hsa-mir-125a) | 80 | 0 | 80 |
| hsa-mir-125b-1-3p(hsa-mir-125b-1) | 4 | 0 | 4 |
| hsa-mir-125b-2-3p(hsa-mir-125b-2) | 4 | 0 | 4 |
| hsa-mir-125b-5p(hsa-mir-125b-1) | 4 | 0 | 4 |
| hsa-mir-125b-5p(hsa-mir-125b-2) | 4 | 0 | 4 |
| hsa-mir-126-3p(hsa-mir-126) | 118 | 33 | 151 |
| hsa-mir-126-5p(hsa-mir-126) | 118 | 28 | 146 |
| hsa-mir-1260a(hsa-mir-1260a) | 0 | 217 | 217 |
| hsa-mir-1260b(hsa-mir-1260b) | 5 | 262 | 267 |
| hsa-mir-1261(hsa-mir-1261) | 0 | 277 | 277 |
| hsa-mir-1262(hsa-mir-1262) | 15 | 0 | 15 |
| hsa-mir-127-3p(hsa-mir-127) | 8 | 5 | 13 |
| hsa-mir-127-5p(hsa-mir-127) | 8 | 0 | 8 |
| hsa-mir-1270(hsa-mir-1270-1) | 15 | 0 | 15 |
| hsa-mir-1270(hsa-mir-1270-2) | 15 | 0 | 15 |
| hsa-mir-1273c(hsa-mir-1273c) | 0 | 17 | 17 |
| hsa-mir-1275(hsa-mir-1275) | 12 | 7 | 19 |
| hsa-mir-1277-3p(hsa-mir-1277) | 125 | 0 | 125 |
| hsa-mir-1277-5p(hsa-mir-1277) | 125 | 0 | 125 |
| hsa-mir-1278(hsa-mir-1278) | 123 | 5 | 128 |
| hsa-mir-128(hsa-mir-128-1) | 1382 | 259 | 1641 |
| hsa-mir-128(hsa-mir-128-2) | 630 | 235 | 865 |
| hsa-mir-1280(hsa-mir-1280) | 0 | 138 | 138 |
| hsa-mir-1284(hsa-mir-1284) | 6 | 0 | 6 |
| hsa-mir-1285-3p(hsa-mir-1285-1) | 3 | 5 | 8 |
| hsa-mir-1285-3p(hsa-mir-1285-2) | 3 | 5 | 8 |
| hsa-mir-1285-5p(hsa-mir-1285-1) | 3 | 0 | 3 |
| hsa-mir-1287(hsa-mir-1287) | 0 | 5 | 5 |
| hsa-mir-1291(hsa-mir-1291) | 5 | 0 | 5 |
| hsa-mir-1294(hsa-mir-1294) | 32 | 0 | 32 |
| hsa-mir-1297(hsa-mir-1297) | 2 | 0 | 2 |
| hsa-mir-1301(hsa-mir-1301) | 53 | 16 | 69 |
| hsa-mir-1304-3p(hsa-mir-1304) | 50 | 5 | 55 |
| hsa-mir-1304-5p(hsa-mir-1304) | 50 | 0 | 50 |
| hsa-mir-1306-3p(hsa-mir-1306) | 53 | 0 | 53 |
| hsa-mir-1306-5p(hsa-mir-1306) | 53 | 0 | 53 |
| hsa-mir-1307-3p(hsa-mir-1307) | 703 | 409 | 1112 |
| hsa-mir-1307-5p(hsa-mir-1307) | 703 | 22 | 725 |
| hsa-mir-130a-3p(hsa-mir-130a) | 87 | 28 | 115 |
| hsa-mir-130a-5p(hsa-mir-130a) | 87 | 0 | 87 |
| hsa-mir-130b-3p(hsa-mir-130b) | 135 | 82 | 217 |
| hsa-mir-130b-5p(hsa-mir-130b) | 135 | 0 | 135 |
| hsa-mir-132-3p(hsa-mir-132) | 17 | 14 | 31 |
| hsa-mir-132-5p(hsa-mir-132) | 17 | 0 | 17 |
| hsa-mir-1322(hsa-mir-598) | 152 | 0 | 152 |
| hsa-mir-134(hsa-mir-134) | 12 | 0 | 12 |
| hsa-mir-139-3p(hsa-mir-139) | 11 | 0 | 11 |
| hsa-mir-139-5p(hsa-mir-139) | 11 | 0 | 11 |
| hsa-mir-140-3p(hsa-mir-140) | 111380 | 31930 | 143310 |
| hsa-mir-140-5p(hsa-mir-140) | 111380 | 70 | 111450 |
| hsa-mir-142-3p(hsa-mir-142) | 16803 | 329 | 17132 |
| hsa-mir-142-5p(hsa-mir-142) | 16803 | 1635 | 18438 |
| hsa-mir-143-3p(hsa-mir-143) | 8070 | 2294 | 10364 |
| hsa-mir-143-5p(hsa-mir-143) | 8070 | 291 | 8361 |
| hsa-mir-144-3p(hsa-mir-144) | 62 | 0 | 62 |
| hsa-mir-144-5p(hsa-mir-144) | 62 | 0 | 62 |
| hsa-mir-145-3p(hsa-mir-145) | 392 | 38 | 430 |
| hsa-mir-145-5p(hsa-mir-145) | 392 | 28 | 420 |
| hsa-mir-146a-3p(hsa-mir-146a) | 41 | 0 | 41 |
| hsa-mir-146a-5p(hsa-mir-146a) | 41 | 23 | 64 |
| hsa-mir-146b-3p(hsa-mir-146b) | 427 | 0 | 427 |
| hsa-mir-146b-5p(hsa-mir-146b) | 427 | 118 | 545 |
| hsa-mir-148a-3p(hsa-mir-148a) | 1672 | 313 | 1985 |
| hsa-mir-148a-5p(hsa-mir-148a) | 1672 | 0 | 1672 |
| hsa-mir-148b-3p(hsa-mir-148b) | 550 | 130 | 680 |
| hsa-mir-148b-5p(hsa-mir-148b) | 550 | 0 | 550 |
| hsa-mir-150-3p(hsa-mir-150) | 108 | 18 | 126 |
| hsa-mir-150-5p(hsa-mir-150) | 108 | 6 | 114 |
| hsa-mir-151a-3p(hsa-mir-151a) | 106 | 8 | 114 |
| hsa-mir-151a-5p(hsa-mir-151a) | 106 | 0 | 106 |
| hsa-mir-151b(hsa-mir-151a) | 106 | 0 | 106 |
| hsa-mir-152(hsa-mir-152) | 125 | 41 | 166 |
| hsa-mir-1537(hsa-mir-1537) | 71 | 0 | 71 |
| hsa-mir-155-3p(hsa-mir-155) | 36 | 0 | 36 |
| hsa-mir-155-5p(hsa-mir-155) | 36 | 37 | 73 |
| hsa-mir-15a-3p(hsa-mir-15a) | 875 | 0 | 875 |
| hsa-mir-15a-5p(hsa-mir-15a) | 875 | 150 | 1025 |
| hsa-mir-15b-3p(hsa-mir-15b) | 1641 | 32 | 1673 |
| hsa-mir-15b-5p(hsa-mir-15b) | 1641 | 113 | 1754 |
| hsa-mir-16-1-3p(hsa-mir-16-1) | 6215 | 0 | 6215 |
| hsa-mir-16-2-3p(hsa-mir-16-2) | 6227 | 16 | 6243 |
| hsa-mir-16-5p(hsa-mir-16-1) | 6215 | 1704 | 7919 |
| hsa-mir-16-5p(hsa-mir-16-2) | 6227 | 1704 | 7931 |
| hsa-mir-17-3p(hsa-mir-17) | 636 | 112 | 748 |
| hsa-mir-17-5p(hsa-mir-17) | 636 | 254 | 890 |
| hsa-mir-181a-2-3p(hsa-mir-181a-2) | 5956 | 12 | 5968 |
| hsa-mir-181a-3p(hsa-mir-181a-1) | 6071 | 24 | 6095 |
| hsa-mir-181a-5p(hsa-mir-181a-1) | 6071 | 1146 | 7217 |
| hsa-mir-181a-5p(hsa-mir-181a-2) | 5956 | 1146 | 7102 |
| hsa-mir-181b-3p(hsa-mir-181b-1) | 380 | 0 | 380 |
| hsa-mir-181b-5p(hsa-mir-181b-1) | 380 | 188 | 568 |
| hsa-mir-181b-5p(hsa-mir-181b-2) | 404 | 188 | 592 |
| hsa-mir-181c-3p(hsa-mir-181c) | 471 | 165 | 636 |
| hsa-mir-181c-5p(hsa-mir-181c) | 471 | 12 | 483 |
| hsa-mir-181d(hsa-mir-181d) | 622 | 313 | 935 |
| hsa-mir-185-3p(hsa-mir-185) | 15455 | 61 | 15516 |
| hsa-mir-185-5p(hsa-mir-185) | 15455 | 5368 | 20823 |
| hsa-mir-186-3p(hsa-mir-186) | 1897 | 0 | 1897 |
| hsa-mir-186-5p(hsa-mir-186) | 1897 | 364 | 2261 |
| hsa-mir-18a-3p(hsa-mir-18a) | 37 | 0 | 37 |
| hsa-mir-18a-5p(hsa-mir-18a) | 37 | 0 | 37 |
| hsa-mir-191-3p(hsa-mir-191) | 58047 | 0 | 58047 |
| hsa-mir-191-5p(hsa-mir-191) | 58047 | 16682 | 74729 |
| hsa-mir-1910(hsa-mir-1910) | 32 | 0 | 32 |
| hsa-mir-192-3p(hsa-mir-192) | 17507 | 0 | 17507 |
| hsa-mir-192-5p(hsa-mir-192) | 17507 | 1357 | 18864 |
| hsa-mir-193a-3p(hsa-mir-193a) | 326 | 18 | 344 |
| hsa-mir-193a-5p(hsa-mir-193a) | 326 | 49 | 375 |
| hsa-mir-194-3p(hsa-mir-194-2) | 169 | 0 | 169 |
| hsa-mir-194-5p(hsa-mir-194-1) | 6 | 0 | 6 |
| hsa-mir-194-5p(hsa-mir-194-2) | 169 | 0 | 169 |
| hsa-mir-196b-3p(hsa-mir-196b) | 109 | 0 | 109 |
| hsa-mir-196b-5p(hsa-mir-196b) | 109 | 171 | 280 |
| hsa-mir-197-3p(hsa-mir-197) | 107 | 12 | 119 |
| hsa-mir-197-5p(hsa-mir-197) | 107 | 5 | 112 |
| hsa-mir-1976(hsa-mir-1976) | 0 | 5 | 5 |
| hsa-mir-199a-3p(hsa-mir-199a-1) | 7910 | 1585 | 9495 |
| hsa-mir-199a-3p(hsa-mir-199a-2) | 7910 | 1585 | 9495 |
| hsa-mir-199a-3p(hsa-mir-199b) | 8233 | 1585 | 9818 |
| hsa-mir-199a-5p(hsa-mir-199a-1) | 7910 | 0 | 7910 |
| hsa-mir-199a-5p(hsa-mir-199a-2) | 7910 | 0 | 7910 |
| hsa-mir-199b-3p(hsa-mir-199a-1) | 7910 | 1585 | 9495 |
| hsa-mir-199b-3p(hsa-mir-199a-2) | 7910 | 1585 | 9495 |
| hsa-mir-199b-3p(hsa-mir-199b) | 8233 | 1585 | 9818 |
| hsa-mir-199b-5p(hsa-mir-199b) | 8233 | 20 | 8253 |
| hsa-mir-19b-1-5p(hsa-mir-19b-1) | 55 | 0 | 55 |
| hsa-mir-19b-2-5p(hsa-mir-19b-2) | 55 | 0 | 55 |
| hsa-mir-19b-3p(hsa-mir-19b-1) | 55 | 7 | 62 |
| hsa-mir-19b-3p(hsa-mir-19b-2) | 55 | 7 | 62 |
| hsa-mir-200b-3p(hsa-mir-200b) | 5 | 7 | 12 |
| hsa-mir-200b-5p(hsa-mir-200b) | 5 | 0 | 5 |
| hsa-mir-200c-3p(hsa-mir-200c) | 9 | 0 | 9 |
| hsa-mir-200c-5p(hsa-mir-200c) | 9 | 0 | 9 |
| hsa-mir-203(hsa-mir-203) | 32 | 0 | 32 |
| hsa-mir-20a-3p(hsa-mir-20a) | 126 | 0 | 126 |
| hsa-mir-20a-5p(hsa-mir-20a) | 126 | 68 | 194 |
| hsa-mir-21-3p(hsa-mir-21) | 41674 | 0 | 41674 |
| hsa-mir-21-5p(hsa-mir-21) | 41674 | 2600 | 44274 |
| hsa-mir-210(hsa-mir-210) | 16 | 0 | 16 |
| hsa-mir-2110(hsa-mir-2110) | 131 | 17 | 148 |
| hsa-mir-2115-3p(hsa-mir-2115) | 202 | 52 | 254 |
| hsa-mir-2115-5p(hsa-mir-2115) | 202 | 0 | 202 |
| hsa-mir-212-3p(hsa-mir-212) | 21 | 0 | 21 |
| hsa-mir-212-5p(hsa-mir-212) | 21 | 0 | 21 |
| hsa-mir-215(hsa-mir-215) | 5 | 28 | 33 |
| hsa-mir-216b(hsa-mir-216b) | 9 | 0 | 9 |
| hsa-mir-22-3p(hsa-mir-22) | 1170 | 83 | 1253 |
| hsa-mir-22-5p(hsa-mir-22) | 1170 | 30 | 1200 |
| hsa-mir-221-3p(hsa-mir-221) | 20585 | 5962 | 26547 |
| hsa-mir-221-5p(hsa-mir-221) | 20585 | 192 | 20777 |
| hsa-mir-222-3p(hsa-mir-222) | 1740 | 258 | 1998 |
| hsa-mir-222-5p(hsa-mir-222) | 1740 | 0 | 1740 |
| hsa-mir-223-3p(hsa-mir-223) | 51725 | 17061 | 68786 |
| hsa-mir-223-5p(hsa-mir-223) | 51725 | 8 | 51733 |
| hsa-mir-224-3p(hsa-mir-224) | 8 | 0 | 8 |
| hsa-mir-224-5p(hsa-mir-224) | 8 | 0 | 8 |
| hsa-mir-2355-3p(hsa-mir-2355) | 293 | 5 | 298 |
| hsa-mir-2355-5p(hsa-mir-2355) | 293 | 5 | 298 |
| hsa-mir-2392(hsa-mir-2392) | 0 | 880 | 880 |
| hsa-mir-23a-3p(hsa-mir-23a) | 34315 | 3156 | 37471 |
| hsa-mir-23a-5p(hsa-mir-23a) | 34315 | 5 | 34320 |
| hsa-mir-23b-3p(hsa-mir-23b) | 815 | 87 | 902 |
| hsa-mir-23b-5p(hsa-mir-23b) | 815 | 0 | 815 |
| hsa-mir-24-1-5p(hsa-mir-24-1) | 3357 | 0 | 3357 |
| hsa-mir-24-2-5p(hsa-mir-24-2) | 3561 | 8 | 3569 |
| hsa-mir-24-3p(hsa-mir-24-1) | 3357 | 7092 | 10449 |
| hsa-mir-24-3p(hsa-mir-24-2) | 3561 | 7068 | 10629 |
| hsa-mir-25-3p(hsa-mir-25) | 90811 | 19404 | 110215 |
| hsa-mir-25-5p(hsa-mir-25) | 90811 | 28 | 90839 |
| hsa-mir-26a-1-3p(hsa-mir-26a-1) | 8611 | 0 | 8611 |
| hsa-mir-26a-2-3p(hsa-mir-26a-2) | 8611 | 0 | 8611 |
| hsa-mir-26a-5p(hsa-mir-26a-1) | 8611 | 2815 | 11426 |
| hsa-mir-26a-5p(hsa-mir-26a-2) | 8611 | 2841 | 11452 |
| hsa-mir-26b-3p(hsa-mir-26b) | 32812 | 0 | 32812 |
| hsa-mir-26b-5p(hsa-mir-26b) | 32812 | 1480 | 34292 |
| hsa-mir-27a-3p(hsa-mir-27a) | 8005 | 991 | 8996 |
| hsa-mir-27a-5p(hsa-mir-27a) | 8005 | 0 | 8005 |
| hsa-mir-27b-3p(hsa-mir-27b) | 562 | 160 | 722 |
| hsa-mir-27b-5p(hsa-mir-27b) | 562 | 0 | 562 |
| hsa-mir-28-3p(hsa-mir-28) | 658 | 174 | 832 |
| hsa-mir-28-5p(hsa-mir-28) | 658 | 87 | 745 |
| hsa-mir-29a-3p(hsa-mir-29a) | 25925 | 2058 | 27983 |
| hsa-mir-29a-5p(hsa-mir-29a) | 25925 | 0 | 25925 |
| hsa-mir-29b-1-5p(hsa-mir-29b-1) | 277 | 0 | 277 |
| hsa-mir-29b-2-5p(hsa-mir-29b-2) | 297 | 0 | 297 |
| hsa-mir-29b-3p(hsa-mir-29b-1) | 277 | 13 | 290 |
| hsa-mir-29b-3p(hsa-mir-29b-2) | 297 | 13 | 310 |
| hsa-mir-29c-3p(hsa-mir-29c) | 1377 | 130 | 1507 |
| hsa-mir-29c-5p(hsa-mir-29c) | 1377 | 0 | 1377 |
| hsa-mir-301a-3p(hsa-mir-301a) | 96 | 0 | 96 |
| hsa-mir-301a-5p(hsa-mir-301a) | 96 | 0 | 96 |
| hsa-mir-30a-3p(hsa-mir-30a) | 49 | 225 | 274 |
| hsa-mir-30a-5p(hsa-mir-30a) | 49 | 9 | 58 |
| hsa-mir-30b-3p(hsa-mir-30b) | 114 | 17 | 131 |
| hsa-mir-30b-5p(hsa-mir-30b) | 114 | 13 | 127 |
| hsa-mir-30c-1-3p(hsa-mir-30c-1) | 504 | 16 | 520 |
| hsa-mir-30c-2-3p(hsa-mir-30c-2) | 288 | 0 | 288 |
| hsa-mir-30c-5p(hsa-mir-30c-1) | 504 | 38 | 542 |
| hsa-mir-30c-5p(hsa-mir-30c-2) | 288 | 38 | 326 |
| hsa-mir-30d-3p(hsa-mir-30d) | 2014 | 0 | 2014 |
| hsa-mir-30d-5p(hsa-mir-30d) | 2014 | 1319 | 3333 |
| hsa-mir-30e-3p(hsa-mir-30e) | 10146 | 9216 | 19362 |
| hsa-mir-30e-5p(hsa-mir-30e) | 10146 | 545 | 10691 |
| hsa-mir-3130-3p(hsa-mir-3130-1) | 11 | 0 | 11 |
| hsa-mir-3130-3p(hsa-mir-3130-2) | 11 | 0 | 11 |
| hsa-mir-3130-5p(hsa-mir-3130-1) | 11 | 0 | 11 |
| hsa-mir-3130-5p(hsa-mir-3130-2) | 11 | 0 | 11 |
| hsa-mir-3135b(hsa-mir-3135b) | 0 | 59 | 59 |
| hsa-mir-3136-3p(hsa-mir-3136) | 8 | 0 | 8 |
| hsa-mir-3136-5p(hsa-mir-3136) | 8 | 0 | 8 |
| hsa-mir-3138(hsa-mir-3138) | 7 | 0 | 7 |
| hsa-mir-3140-3p(hsa-mir-3140) | 6 | 0 | 6 |
| hsa-mir-3140-5p(hsa-mir-3140) | 6 | 0 | 6 |
| hsa-mir-3141(hsa-mir-3141) | 0 | 20 | 20 |
| hsa-mir-3143(hsa-mir-3143) | 35 | 0 | 35 |
| hsa-mir-3154(hsa-mir-3154) | 8 | 12 | 20 |
| hsa-mir-3168(hsa-mir-3168) | 0 | 792 | 792 |
| hsa-mir-3179(hsa-mir-3179-1) | 5 | 0 | 5 |
| hsa-mir-3179(hsa-mir-3179-2) | 5 | 0 | 5 |
| hsa-mir-3179(hsa-mir-3179-3) | 5 | 0 | 5 |
| hsa-mir-3182(hsa-mir-3182) | 0 | 9 | 9 |
| hsa-mir-32-3p(hsa-mir-32) | 47 | 0 | 47 |
| hsa-mir-32-5p(hsa-mir-32) | 47 | 13 | 60 |
| hsa-mir-3202(hsa-mir-3202-2) | 6 | 0 | 6 |
| hsa-mir-320a(hsa-mir-320a) | 16844 | 8180 | 25024 |
| hsa-mir-320b(hsa-mir-320b-1) | 67 | 136 | 203 |
| hsa-mir-320b(hsa-mir-320b-2) | 72 | 136 | 208 |
| hsa-mir-320c(hsa-mir-320c-1) | 5 | 69 | 74 |
| hsa-mir-320c(hsa-mir-320c-2) | 5 | 42 | 47 |
| hsa-mir-320d(hsa-mir-320d-1) | 2 | 11 | 13 |
| hsa-mir-320d(hsa-mir-320d-2) | 2 | 11 | 13 |
| hsa-mir-323a-3p(hsa-mir-323a) | 5 | 0 | 5 |
| hsa-mir-323a-5p(hsa-mir-323a) | 5 | 0 | 5 |
| hsa-mir-323b-3p(hsa-mir-323b) | 7 | 0 | 7 |
| hsa-mir-323b-5p(hsa-mir-323b) | 7 | 0 | 7 |
| hsa-mir-324-3p(hsa-mir-324) | 47 | 0 | 47 |
| hsa-mir-324-5p(hsa-mir-324) | 47 | 0 | 47 |
| hsa-mir-328(hsa-mir-328) | 25 | 18 | 43 |
| hsa-mir-330-3p(hsa-mir-330) | 2329 | 819 | 3148 |
| hsa-mir-330-5p(hsa-mir-330) | 2329 | 0 | 2329 |
| hsa-mir-331-3p(hsa-mir-331) | 90 | 36 | 126 |
| hsa-mir-331-5p(hsa-mir-331) | 90 | 0 | 90 |
| hsa-mir-335-3p(hsa-mir-335) | 65 | 0 | 65 |
| hsa-mir-335-5p(hsa-mir-335) | 65 | 0 | 65 |
| hsa-mir-338-3p(hsa-mir-338) | 496 | 0 | 496 |
| hsa-mir-338-5p(hsa-mir-338) | 496 | 14 | 510 |
| hsa-mir-339-3p(hsa-mir-339) | 100 | 85 | 185 |
| hsa-mir-339-5p(hsa-mir-339) | 100 | 0 | 100 |
| hsa-mir-33a-3p(hsa-mir-33a) | 440 | 0 | 440 |
| hsa-mir-33a-5p(hsa-mir-33a) | 440 | 101 | 541 |
| hsa-mir-33b-3p(hsa-mir-33b) | 24 | 0 | 24 |
| hsa-mir-33b-5p(hsa-mir-33b) | 24 | 0 | 24 |
| hsa-mir-340-3p(hsa-mir-340) | 7424 | 0 | 7424 |
| hsa-mir-340-5p(hsa-mir-340) | 7424 | 980 | 8404 |
| hsa-mir-342-3p(hsa-mir-342) | 200 | 20 | 220 |
| hsa-mir-342-5p(hsa-mir-342) | 200 | 0 | 200 |
| hsa-mir-345-3p(hsa-mir-345) | 61 | 0 | 61 |
| hsa-mir-345-5p(hsa-mir-345) | 61 | 698 | 759 |
| hsa-mir-34c-3p(hsa-mir-34c) | 237 | 0 | 237 |
| hsa-mir-34c-5p(hsa-mir-34c) | 237 | 134 | 371 |
| hsa-mir-3605-3p(hsa-mir-3605) | 106 | 0 | 106 |
| hsa-mir-3605-5p(hsa-mir-3605) | 106 | 0 | 106 |
| hsa-mir-361-3p(hsa-mir-361) | 68 | 0 | 68 |
| hsa-mir-361-5p(hsa-mir-361) | 68 | 52 | 120 |
| hsa-mir-3614-3p(hsa-mir-3614) | 243 | 0 | 243 |
| hsa-mir-3614-5p(hsa-mir-3614) | 243 | 0 | 243 |
| hsa-mir-3615(hsa-mir-3615) | 50 | 5 | 55 |
| hsa-mir-362-3p(hsa-mir-362) | 38 | 0 | 38 |
| hsa-mir-362-5p(hsa-mir-362) | 38 | 0 | 38 |
| hsa-mir-363-3p(hsa-mir-363) | 145 | 57 | 202 |
| hsa-mir-363-5p(hsa-mir-363) | 145 | 0 | 145 |
| hsa-mir-365a-3p(hsa-mir-365a) | 27 | 0 | 27 |
| hsa-mir-365a-3p(hsa-mir-365b) | 27 | 0 | 27 |
| hsa-mir-365a-5p(hsa-mir-365a) | 27 | 0 | 27 |
| hsa-mir-365b-3p(hsa-mir-365a) | 27 | 0 | 27 |
| hsa-mir-365b-3p(hsa-mir-365b) | 27 | 0 | 27 |
| hsa-mir-365b-5p(hsa-mir-365b) | 27 | 0 | 27 |
| hsa-mir-3676-3p(hsa-mir-3676) | 5 | 0 | 5 |
| hsa-mir-3676-5p(hsa-mir-3676) | 5 | 6 | 11 |
| hsa-mir-369-3p(hsa-mir-369) | 8 | 0 | 8 |
| hsa-mir-369-5p(hsa-mir-369) | 8 | 0 | 8 |
| hsa-mir-3690(hsa-mir-3690) | 542 | 24 | 566 |
| hsa-mir-371b-3p(hsa-mir-371b) | 75 | 0 | 75 |
| hsa-mir-371b-5p(hsa-mir-371b) | 75 | 8 | 83 |
| hsa-mir-374a-3p(hsa-mir-374a) | 1509 | 82 | 1591 |
| hsa-mir-374a-5p(hsa-mir-374a) | 1509 | 71 | 1580 |
| hsa-mir-374b-3p(hsa-mir-374b) | 549 | 0 | 549 |
| hsa-mir-374b-5p(hsa-mir-374b) | 549 | 127 | 676 |
| hsa-mir-378a-3p(hsa-mir-378a) | 2491 | 2303 | 4794 |
| hsa-mir-378a-5p(hsa-mir-378a) | 2491 | 0 | 2491 |
| hsa-mir-378c(hsa-mir-378c) | 373 | 94 | 467 |
| hsa-mir-378d(hsa-mir-378d-1) | 16 | 0 | 16 |
| hsa-mir-378d(hsa-mir-378d-2) | 16 | 0 | 16 |
| hsa-mir-378f(hsa-mir-378f) | 0 | 17 | 17 |
| hsa-mir-378i(hsa-mir-378i) | 0 | 126 | 126 |
| hsa-mir-379-3p(hsa-mir-379) | 5 | 0 | 5 |
| hsa-mir-379-5p(hsa-mir-379) | 5 | 0 | 5 |
| hsa-mir-382-3p(hsa-mir-382) | 26 | 0 | 26 |
| hsa-mir-382-5p(hsa-mir-382) | 26 | 0 | 26 |
| hsa-mir-3909(hsa-mir-3909) | 16 | 0 | 16 |
| hsa-mir-3928(hsa-mir-3928) | 13 | 141 | 154 |
| hsa-mir-409-3p(hsa-mir-409) | 13 | 0 | 13 |
| hsa-mir-409-5p(hsa-mir-409) | 13 | 0 | 13 |
| hsa-mir-410(hsa-mir-410) | 8 | 0 | 8 |
| hsa-mir-421(hsa-mir-421) | 236 | 89 | 325 |
| hsa-mir-423-3p(hsa-mir-423) | 28070 | 787 | 28857 |
| hsa-mir-423-5p(hsa-mir-423) | 28070 | 5311 | 33381 |
| hsa-mir-424-3p(hsa-mir-424) | 1812 | 134 | 1946 |
| hsa-mir-424-5p(hsa-mir-424) | 1812 | 42 | 1854 |
| hsa-mir-425-3p(hsa-mir-425) | 2241 | 299 | 2540 |
| hsa-mir-425-5p(hsa-mir-425) | 2241 | 179 | 2420 |
| hsa-mir-4286(hsa-mir-4286) | 654 | 37 | 691 |
| hsa-mir-429(hsa-mir-429) | 5 | 0 | 5 |
| hsa-mir-432-3p(hsa-mir-432) | 26 | 0 | 26 |
| hsa-mir-432-5p(hsa-mir-432) | 26 | 20 | 46 |
| hsa-mir-433(hsa-mir-433) | 15 | 0 | 15 |
| hsa-mir-4433-3p(hsa-mir-4433) | 168 | 27 | 195 |
| hsa-mir-4433-5p(hsa-mir-4433) | 168 | 0 | 168 |
| hsa-mir-4443(hsa-mir-4443) | 54 | 74 | 128 |
| hsa-mir-4448(hsa-mir-4448) | 0 | 13 | 13 |
| hsa-mir-4454(hsa-mir-4454) | 8 | 0 | 8 |
| hsa-mir-4477b(hsa-mir-4477b) | 5 | 0 | 5 |
| hsa-mir-4486(hsa-mir-4486) | 0 | 6 | 6 |
| hsa-mir-4487(hsa-mir-4487) | 33 | 0 | 33 |
| hsa-mir-4500(hsa-mir-4500) | 6 | 0 | 6 |
| hsa-mir-4508(hsa-mir-4508) | 8 | 281 | 289 |
| hsa-mir-450a-3p(hsa-mir-450a-2) | 127 | 0 | 127 |
| hsa-mir-450a-5p(hsa-mir-450a-1) | 84 | 2 | 86 |
| hsa-mir-450a-5p(hsa-mir-450a-2) | 127 | 2 | 129 |
| hsa-mir-450b-3p(hsa-mir-450b) | 81 | 0 | 81 |
| hsa-mir-450b-5p(hsa-mir-450b) | 81 | 27 | 108 |
| hsa-mir-4510(hsa-mir-4510) | 0 | 282 | 282 |
| hsa-mir-451a(hsa-mir-451a) | 670 | 0 | 670 |
| hsa-mir-4531(hsa-mir-4531) | 0 | 7 | 7 |
| hsa-mir-454-3p(hsa-mir-454) | 7 | 0 | 7 |
| hsa-mir-454-5p(hsa-mir-454) | 7 | 0 | 7 |
| hsa-mir-4651(hsa-mir-4651) | 0 | 8 | 8 |
| hsa-mir-4676-3p(hsa-mir-4676) | 5 | 0 | 5 |
| hsa-mir-4676-5p(hsa-mir-4676) | 5 | 0 | 5 |
| hsa-mir-4724-3p(hsa-mir-4724) | 20 | 0 | 20 |
| hsa-mir-4724-5p(hsa-mir-4724) | 20 | 0 | 20 |
| hsa-mir-4750(hsa-mir-4750) | 5 | 0 | 5 |
| hsa-mir-4772-3p(hsa-mir-4772) | 66 | 20 | 86 |
| hsa-mir-4772-5p(hsa-mir-4772) | 66 | 10 | 76 |
| hsa-mir-4773(hsa-mir-4773-1) | 3 | 0 | 3 |
| hsa-mir-4773(hsa-mir-4773-2) | 3 | 0 | 3 |
| hsa-mir-4791(hsa-mir-4791) | 26 | 0 | 26 |
| hsa-mir-484(hsa-mir-484) | 54 | 7 | 61 |
| hsa-mir-485-3p(hsa-mir-485) | 16 | 0 | 16 |
| hsa-mir-485-5p(hsa-mir-485) | 16 | 0 | 16 |
| hsa-mir-486-3p(hsa-mir-486) | 69 | 9 | 78 |
| hsa-mir-486-5p(hsa-mir-486) | 69 | 15 | 84 |
| hsa-mir-487b(hsa-mir-487b) | 12 | 0 | 12 |
| hsa-mir-491-3p(hsa-mir-491) | 6 | 0 | 6 |
| hsa-mir-491-5p(hsa-mir-491) | 6 | 0 | 6 |
| hsa-mir-495(hsa-mir-495) | 24 | 0 | 24 |
| hsa-mir-499a-3p(hsa-mir-499a) | 105 | 0 | 105 |
| hsa-mir-499a-5p(hsa-mir-499a) | 105 | 0 | 105 |
| hsa-mir-499b-3p(hsa-mir-499b) | 5 | 0 | 5 |
| hsa-mir-499b-5p(hsa-mir-499b) | 5 | 0 | 5 |
| hsa-mir-500a-3p(hsa-mir-500a) | 70 | 0 | 70 |
| hsa-mir-500a-5p(hsa-mir-500a) | 70 | 0 | 70 |
| hsa-mir-500b(hsa-mir-500a) | 70 | 0 | 70 |
| hsa-mir-501-3p(hsa-mir-501) | 6 | 0 | 6 |
| hsa-mir-501-5p(hsa-mir-501) | 6 | 0 | 6 |
| hsa-mir-5010-3p(hsa-mir-5010) | 37 | 0 | 37 |
| hsa-mir-5010-5p(hsa-mir-5010) | 37 | 0 | 37 |
| hsa-mir-502-3p(hsa-mir-502) | 110 | 9 | 119 |
| hsa-mir-502-5p(hsa-mir-502) | 110 | 0 | 110 |
| hsa-mir-503(hsa-mir-503) | 19 | 114 | 133 |
| hsa-mir-504(hsa-mir-504) | 87 | 25 | 112 |
| hsa-mir-505-3p(hsa-mir-505) | 295 | 0 | 295 |
| hsa-mir-505-5p(hsa-mir-505) | 295 | 8 | 303 |
| hsa-mir-5187-3p(hsa-mir-5187) | 21 | 0 | 21 |
| hsa-mir-5187-5p(hsa-mir-5187) | 21 | 0 | 21 |
| hsa-mir-532-3p(hsa-mir-532) | 883 | 0 | 883 |
| hsa-mir-532-5p(hsa-mir-532) | 883 | 115 | 998 |
| hsa-mir-539-3p(hsa-mir-539) | 34 | 0 | 34 |
| hsa-mir-539-5p(hsa-mir-539) | 34 | 0 | 34 |
| hsa-mir-542-3p(hsa-mir-542) | 252 | 25 | 277 |
| hsa-mir-542-5p(hsa-mir-542) | 252 | 0 | 252 |
| hsa-mir-543(hsa-mir-543) | 43 | 0 | 43 |
| hsa-mir-548aa(hsa-mir-548t) | 10 | 0 | 10 |
| hsa-mir-548ad(hsa-mir-548ad) | 6 | 0 | 6 |
| hsa-mir-548ae(hsa-mir-548ae-2) | 15 | 0 | 15 |
| hsa-mir-548ak(hsa-mir-548ak) | 10 | 0 | 10 |
| hsa-mir-548am-3p(hsa-mir-548am) | 7 | 0 | 7 |
| hsa-mir-548am-5p(hsa-mir-548am) | 7 | 0 | 7 |
| hsa-mir-548am-5p(hsa-mir-548c) | 7 | 0 | 7 |
| hsa-mir-548am-5p(hsa-mir-548o-2) | 21 | 0 | 21 |
| hsa-mir-548ap-3p(hsa-mir-548t) | 10 | 0 | 10 |
| hsa-mir-548ap-5p(hsa-mir-548j) | 93 | 0 | 93 |
| hsa-mir-548au-3p(hsa-mir-548au) | 30 | 0 | 30 |
| hsa-mir-548au-5p(hsa-mir-548am) | 7 | 0 | 7 |
| hsa-mir-548au-5p(hsa-mir-548c) | 7 | 0 | 7 |
| hsa-mir-548au-5p(hsa-mir-548o-2) | 21 | 0 | 21 |
| hsa-mir-548av-3p(hsa-mir-548o) | 14 | 0 | 14 |
| hsa-mir-548av-3p(hsa-mir-548o-2) | 21 | 0 | 21 |
| hsa-mir-548av-5p(hsa-mir-548k) | 246 | 0 | 246 |
| hsa-mir-548c-3p(hsa-mir-548c) | 7 | 0 | 7 |
| hsa-mir-548c-5p(hsa-mir-548am) | 7 | 0 | 7 |
| hsa-mir-548c-5p(hsa-mir-548c) | 7 | 0 | 7 |
| hsa-mir-548c-5p(hsa-mir-548o-2) | 21 | 0 | 21 |
| hsa-mir-548d-3p(hsa-mir-548d-1) | 14 | 0 | 14 |
| hsa-mir-548d-3p(hsa-mir-548d-2) | 14 | 0 | 14 |
| hsa-mir-548d-5p(hsa-mir-548d-1) | 14 | 9 | 23 |
| hsa-mir-548d-5p(hsa-mir-548d-2) | 14 | 9 | 23 |
| hsa-mir-548e(hsa-mir-548e) | 588 | 114 | 702 |
| hsa-mir-548f(hsa-mir-548f-2) | 3 | 0 | 3 |
| hsa-mir-548f(hsa-mir-548f-3) | 3 | 0 | 3 |
| hsa-mir-548j(hsa-mir-548j) | 93 | 0 | 93 |
| hsa-mir-548k(hsa-mir-548k) | 246 | 0 | 246 |
| hsa-mir-548l(hsa-mir-548l) | 54 | 0 | 54 |
| hsa-mir-548o-3p(hsa-mir-548o) | 14 | 0 | 14 |
| hsa-mir-548o-3p(hsa-mir-548o-2) | 21 | 0 | 21 |
| hsa-mir-548o-5p(hsa-mir-548am) | 7 | 0 | 7 |
| hsa-mir-548o-5p(hsa-mir-548c) | 7 | 0 | 7 |
| hsa-mir-548o-5p(hsa-mir-548o-2) | 21 | 0 | 21 |
| hsa-mir-548t-3p(hsa-mir-548t) | 10 | 0 | 10 |
| hsa-mir-548t-5p(hsa-mir-548t) | 10 | 7 | 17 |
| hsa-mir-548u(hsa-mir-548u) | 9 | 0 | 9 |
| hsa-mir-548w(hsa-mir-548w) | 30 | 0 | 30 |
| hsa-mir-550a-5p(hsa-mir-550a-1) | 0 | 12 | 12 |
| hsa-mir-550a-5p(hsa-mir-550a-2) | 0 | 12 | 12 |
| hsa-mir-551a(hsa-mir-551a) | 63 | 0 | 63 |
| hsa-mir-556-3p(hsa-mir-556) | 11 | 0 | 11 |
| hsa-mir-556-5p(hsa-mir-556) | 11 | 0 | 11 |
| hsa-mir-574-3p(hsa-mir-574) | 82 | 28 | 110 |
| hsa-mir-574-5p(hsa-mir-574) | 82 | 0 | 82 |
| hsa-mir-576-3p(hsa-mir-576) | 151 | 6 | 157 |
| hsa-mir-576-5p(hsa-mir-576) | 151 | 0 | 151 |
| hsa-mir-582-3p(hsa-mir-582) | 214 | 24 | 238 |
| hsa-mir-582-5p(hsa-mir-582) | 214 | 8 | 222 |
| hsa-mir-584-3p(hsa-mir-584) | 101 | 0 | 101 |
| hsa-mir-584-5p(hsa-mir-584) | 101 | 0 | 101 |
| hsa-mir-589-3p(hsa-mir-589) | 48 | 0 | 48 |
| hsa-mir-589-5p(hsa-mir-589) | 48 | 21 | 69 |
| hsa-mir-590-3p(hsa-mir-590) | 43 | 0 | 43 |
| hsa-mir-590-5p(hsa-mir-590) | 43 | 0 | 43 |
| hsa-mir-598(hsa-mir-598) | 152 | 9 | 161 |
| hsa-mir-618(hsa-mir-618) | 60 | 6 | 66 |
| hsa-mir-619(hsa-mir-619) | 5 | 0 | 5 |
| hsa-mir-625-3p(hsa-mir-625) | 50 | 0 | 50 |
| hsa-mir-625-5p(hsa-mir-625) | 50 | 0 | 50 |
| hsa-mir-628-3p(hsa-mir-628) | 165 | 0 | 165 |
| hsa-mir-628-5p(hsa-mir-628) | 165 | 33 | 198 |
| hsa-mir-629-3p(hsa-mir-629) | 145 | 0 | 145 |
| hsa-mir-629-5p(hsa-mir-629) | 145 | 5 | 150 |
| hsa-mir-637(hsa-mir-637) | 8 | 0 | 8 |
| hsa-mir-641(hsa-mir-641) | 15 | 0 | 15 |
| hsa-mir-642a-3p(hsa-mir-642a) | 10 | 8 | 18 |
| hsa-mir-642a-5p(hsa-mir-642a) | 10 | 0 | 10 |
| hsa-mir-644b-3p(hsa-mir-644b) | 10 | 0 | 10 |
| hsa-mir-651(hsa-mir-651) | 16 | 12 | 28 |
| hsa-mir-652-3p(hsa-mir-652) | 232 | 425 | 657 |
| hsa-mir-652-5p(hsa-mir-652) | 232 | 0 | 232 |
| hsa-mir-659-3p(hsa-mir-659) | 15 | 0 | 15 |
| hsa-mir-659-5p(hsa-mir-659) | 15 | 0 | 15 |
| hsa-mir-660-3p(hsa-mir-660) | 70 | 0 | 70 |
| hsa-mir-660-5p(hsa-mir-660) | 70 | 0 | 70 |
| hsa-mir-664-3p(hsa-mir-664) | 785 | 0 | 785 |
| hsa-mir-664-5p(hsa-mir-664) | 785 | 32 | 817 |
| hsa-mir-7-1-3p(hsa-mir-7-1) | 138 | 26 | 164 |
| hsa-mir-7-2-3p(hsa-mir-7-2) | 48 | 0 | 48 |
| hsa-mir-7-5p(hsa-mir-7-1) | 138 | 0 | 138 |
| hsa-mir-7-5p(hsa-mir-7-2) | 48 | 0 | 48 |
| hsa-mir-7-5p(hsa-mir-7-3) | 48 | 0 | 48 |
| hsa-mir-720(hsa-mir-720) | 447 | 43 | 490 |
| hsa-mir-744-3p(hsa-mir-744) | 2313 | 0 | 2313 |
| hsa-mir-744-5p(hsa-mir-744) | 2313 | 1361 | 3674 |
| hsa-mir-766-3p(hsa-mir-766) | 33 | 14 | 47 |
| hsa-mir-766-5p(hsa-mir-766) | 33 | 6 | 39 |
| hsa-mir-769-3p(hsa-mir-769) | 15 | 0 | 15 |
| hsa-mir-769-5p(hsa-mir-769) | 15 | 15 | 30 |
| hsa-mir-873-3p(hsa-mir-873) | 57 | 0 | 57 |
| hsa-mir-873-5p(hsa-mir-873) | 57 | 0 | 57 |
| hsa-mir-889(hsa-mir-889) | 19 | 0 | 19 |
| hsa-mir-9-3p(hsa-mir-9-1) | 7 | 0 | 7 |
| hsa-mir-9-3p(hsa-mir-9-2) | 7 | 0 | 7 |
| hsa-mir-9-3p(hsa-mir-9-3) | 7 | 0 | 7 |
| hsa-mir-9-5p(hsa-mir-9-1) | 7 | 0 | 7 |
| hsa-mir-9-5p(hsa-mir-9-2) | 7 | 0 | 7 |
| hsa-mir-9-5p(hsa-mir-9-3) | 7 | 0 | 7 |
| hsa-mir-92a-1-5p(hsa-mir-92a-1) | 2277 | 18 | 2295 |
| hsa-mir-92a-2-5p(hsa-mir-92a-2) | 1903 | 0 | 1903 |
| hsa-mir-92a-3p(hsa-mir-92a-1) | 2277 | 1189 | 3466 |
| hsa-mir-92a-3p(hsa-mir-92a-2) | 1903 | 1121 | 3024 |
| hsa-mir-92b-3p(hsa-mir-92b) | 10 | 5 | 15 |
| hsa-mir-92b-5p(hsa-mir-92b) | 10 | 0 | 10 |
| hsa-mir-93-3p(hsa-mir-93) | 4842 | 7 | 4849 |
| hsa-mir-93-5p(hsa-mir-93) | 4842 | 1945 | 6787 |
| hsa-mir-935(hsa-mir-935) | 11 | 0 | 11 |
| hsa-mir-941(hsa-mir-941-1) | 384 | 253 | 637 |
| hsa-mir-941(hsa-mir-941-2) | 370 | 253 | 623 |
| hsa-mir-941(hsa-mir-941-3) | 370 | 253 | 623 |
| hsa-mir-941(hsa-mir-941-4) | 370 | 253 | 623 |
| hsa-mir-942(hsa-mir-942) | 10 | 0 | 10 |
| hsa-mir-98(hsa-mir-98) | 1221 | 322 | 1543 |
| hsa-mir-99a-3p(hsa-mir-99a) | 33 | 0 | 33 |
| hsa-mir-99a-5p(hsa-mir-99a) | 33 | 10 | 43 |
| hsa-mir-99b-3p(hsa-mir-99b) | 317 | 9 | 326 |
| hsa-mir-99b-5p(hsa-mir-99b) | 317 | 93 | 410 |
| Sum(Avg) | 3959056 | 460166 | 4419222 |

  

|  |
| --- |
| hsa-let-7a-2-3p(hsa-let-7a-2) AGGTTGAGGTAGTAGGTTGTATAGTTTAGAATTACATCAAGGGAGATAACTGTACAGCCTCCTAGCTTTCCT (((..(((.(((.(((((((((((((.....(..(.....)..)...))))))))))))).))).))).))) (-25.20) \*\*\*\*\*\*\*\*\*\*\*\*\*\*\*\*\*\*\*\*\*\*\*\*\*\*\*\*\*\*\*\*\*\*\*\*\*\*\*\*\*\*\*\*\*\*\*\*\*CTGTACAGCCTCCTAGCTTTCC\* T  M |
| ....TGAGGTAGTAGGTTGTATAGTT............................................... 120200   | 120200; |
| ....TGAGGTAGTAGGTTGTATAGT................................................ 19848   | 19848; |
| ....TGAGGTAGTAGGTTGTATAGTTT.............................................. 12180   | 12180; |
| ....TGAGGTAGTAGGTTGTATAG................................................. 2746   | 2746; |
| ....TGAGGTAGTAGGNTGTATAGTT............................................... 1804   | 1804; |
| ....TGAGGTAGTAGGTTGNATAGTT............................................... 563   | 563; |
| ....TGAGGTAGTAGGTNNTATAGTT............................................... 529   | 529; |
| ....TGAGGTAGTAGGTTGTATAGTTTA............................................. 410   | 410; |
| ....TGAGGTAGTAGGTTGTATA.................................................. 322   | 322; |
| ....TGAGGTAGTAGGNTGTATAGT................................................ 278   | 278; |
| ....TGAGGTAGTAGGTTNTATAGTT............................................... 212   | 212; |
| ....TGAGGTAGTAGGTTGTATANTT............................................... 172   | 172; |
| ....TGAGGTAGTAGGNTGTATAGTTT.............................................. 168   | 168; |
| .....GAGGTAGTAGGTTGTATAGTT............................................... 158   | 158; |
| ....TGAGGTAGTAGGTTGTAT................................................... 114   | 114; |
| ....TGAGGTAGTAGGTTGNATAGT................................................ 75   | 75; |
| ....TGAGGTAGTAGGTTGNATAGTTT.............................................. 72   | 72; |
| ....TGAGGTAGTAGGTNNTATAGT................................................ 71   | 71; |
| ....TGAGGTAGTAGG......................................................... 63   | 63; |
| ....TGAGGTAGTAGGTNNTATAGTTT.............................................. 44   | 44; |
| ....TGAGGTAGTAGGTTGTATANNT............................................... 42   | 42; |
| ....TGAGGTAGTAGGTTGTA.................................................... 35   | 35; |
| ....TGAGGTAGTAGGNTGTATAG................................................. 34   | 34; |
| ....TGAGGTAGTAGGTTGTATANT................................................ 28   | 28; |
| .....GAGGTAGTAGGTTGTATAGTTT.............................................. 28   | 28; |
| .....GAGGTAGTAGGTTGTATAGT................................................ 27   | 27; |
| ....TGAGGTAGTAGGTTNTATAGT................................................ 25   | 25; |
| ...TTGAGGTAGTAGGTTGTATAGT................................................ 25   | 25; |
| ....TGAGGTAGTAGGTTNTATAGTTT.............................................. 18   | 18; |
| ....TGAGGTAGTAGGTTGT..................................................... 17   | 17; |
| ....TGAGGTAGTAGGTTGTATANTTT.............................................. 15   | 15; |
| ......AGGTAGTAGGTTGTATAGTT............................................... 13   | 13; |
| ....TGAGGTAGTAGGTTGNATAG................................................. 11   | 11; |
| ....TGAGGTAGTAGGTNNTATAG................................................. 10   | 10; |
| ....TGAGGTAGTAGGTTGTATANNTT.............................................. 9   | 9; |
| ...TTGAGGTAGTAGGTTGTATAG................................................. 5   | 5; |
| ....TGAGGTAGTAGGTT....................................................... 4   | 4; |
| .....GAGGTAGTAGGTTGTATAG................................................. 3   | 3; |
| ....TGAGGTAGTAGGTTG...................................................... 3   | 3; |
| ......AGGTAGTAGGTTGTATAGT................................................ 2   | 2; |
| .......GGTAGTAGGTTGTATAGTT............................................... 1   | 1; |
| ------------------------------------------------------------------------ 160384 |
| ------------------------------------------------------------------------ 160384 |
| hsa-let-7a-3p(hsa-let-7a-1) TGGGATGAGGTAGTAGGTTGTATAGTTTTAGGGTCACACCCACCACTGGGAGATAACTATACAATCTACTGTCTTTCCTA (((((.(((..((((((((((((((((...(((.....))).((....))....))))))))))))))))..)))))))) (-35.60) \*\*\*\*\*\*\*\*\*\*\*\*\*\*\*\*\*\*\*\*\*\*\*\*\*\*\*\*\*\*\*\*\*\*\*\*\*\*\*\*\*\*\*\*\*\*\*\*\*\*\*\*\*\*\*\*CTATACAATCTACTGTCTTTC\*\*\* T  M |
| .....TGAGGTAGTAGGTTGTATAGTT...................................................... 120200   | 120200; |
| .....TGAGGTAGTAGGTTGTATAGT....................................................... 19848   | 19848; |
| .....TGAGGTAGTAGGTTGTATAGTTT..................................................... 12180   | 12180; |
| .....TGAGGTAGTAGGTTGTATAG........................................................ 2746   | 2746; |
| .....TGAGGTAGTAGGNTGTATAGTT...................................................... 1804   | 1804; |
| .....TGAGGTAGTAGGTTGTATAGTTTT.................................................... 670   | 670; |
| .....TGAGGTAGTAGGTTGNATAGTT...................................................... 563   | 563; |
| .....TGAGGTAGTAGGTNNTATAGTT...................................................... 529   | 529; |
| .....TGAGGTAGTAGGTTGTATA......................................................... 322   | 322; |
| .....TGAGGTAGTAGGNTGTATAGT....................................................... 278   | 278; |
| .....TGAGGTAGTAGGTTNTATAGTT...................................................... 212   | 212; |
| .....TGAGGTAGTAGGTTGTATANTT...................................................... 172   | 172; |
| .....TGAGGTAGTAGGNTGTATAGTTT..................................................... 168   | 168; |
| ......GAGGTAGTAGGTTGTATAGTT...................................................... 158   | 158; |
| .....TGAGGTAGTAGGTTGTAT.......................................................... 114   | 114; |
| .....TGAGGTAGTAGGTTGNATAGT....................................................... 75   | 75; |
| .....TGAGGTAGTAGGTTGNATAGTTT..................................................... 72   | 72; |
| .....TGAGGTAGTAGGTNNTATAGT....................................................... 71   | 71; |
| .....TGAGGTAGTAGG................................................................ 63   | 63; |
| .....TGAGGTAGTAGGTNNTATAGTTT..................................................... 44   | 44; |
| .....TGAGGTAGTAGGTTGTATANNT...................................................... 42   | 42; |
| .....TGAGGTAGTAGGTTGTA........................................................... 35   | 35; |
| .....TGAGGTAGTAGGNTGTATAG........................................................ 34   | 34; |
| .....TGAGGTAGTAGGTTGTATANT....................................................... 28   | 28; |
| ......GAGGTAGTAGGTTGTATAGTTT..................................................... 28   | 28; |
| ......GAGGTAGTAGGTTGTATAGT....................................................... 27   | 27; |
| .....TGAGGTAGTAGGTTNTATAGT....................................................... 25   | 25; |
| .....TGAGGTAGTAGGTTNTATAGTTT..................................................... 18   | 18; |
| .....TGAGGTAGTAGGTTGT............................................................ 17   | 17; |
| .....TGAGGTAGTAGGTTGTATANTTT..................................................... 15   | 15; |
| .......AGGTAGTAGGTTGTATAGTT...................................................... 13   | 13; |
| .....TGAGGTAGTAGGTTGNATAG........................................................ 11   | 11; |
| .....TGAGGTAGTAGGTNNTATAG........................................................ 10   | 10; |
| .....TGAGGTAGTAGGTTGTATANNTT..................................................... 9   | 9; |
| ....ATGAGGTAGTAGGTTGTATAGTT...................................................... 8   | 8; |
| ........................................................CTATACAATCTACTGTCTTTC....\* 8   | 8; |
| .....TGAGGTAGTAGGTT.............................................................. 4   | 4; |
| ......GAGGTAGTAGGTTGTATAG........................................................ 3   | 3; |
| .....TGAGGTAGTAGGTTG............................................................. 3   | 3; |
| .......AGGTAGTAGGTTGTATAGT....................................................... 2   | 2; |
| ........GGTAGTAGGTTGTATAGTT...................................................... 1   | 1; |
| -------------------------------------------------------------------------------- 160630 |
| ........................................................CTATACAATCTACTGTCTTTCt... 24   | 24; |
| -------------------------------------------------------------------------------- 24 |
| -------------------------------------------------------------------------------- 160654 |
| hsa-let-7a-3p(hsa-let-7a-3) GGGTGAGGTAGTAGGTTGTATAGTTTGGGGCTCTGCCCTGCTATGGGATAACTATACAATCTACTGTCTTTCCT (((.(((..((((((((((((((((((((((...)))))).........))))))))))))))))..))).))) (-34.40) \*\*\*\*\*\*\*\*\*\*\*\*\*\*\*\*\*\*\*\*\*\*\*\*\*\*\*\*\*\*\*\*\*\*\*\*\*\*\*\*\*\*\*\*\*\*\*\*\*\*\*CTATACAATCTACTGTCTTTC\*\* T  M |
| ...TGAGGTAGTAGGTTGTATAGTT.................................................. 120200   | 120200; |
| ...TGAGGTAGTAGGTTGTATAGT................................................... 19848   | 19848; |
| ...TGAGGTAGTAGGTTGTATAGTTT................................................. 12180   | 12180; |
| ...TGAGGTAGTAGGTTGTATAG.................................................... 2746   | 2746; |
| ...TGAGGTAGTAGGNTGTATAGTT.................................................. 1804   | 1804; |
| ...TGAGGTAGTAGGTTGNATAGTT.................................................. 563   | 563; |
| ...TGAGGTAGTAGGTNNTATAGTT.................................................. 529   | 529; |
| ...TGAGGTAGTAGGTTGTATA..................................................... 322   | 322; |
| ...TGAGGTAGTAGGNTGTATAGT................................................... 278   | 278; |
| ...TGAGGTAGTAGGTTNTATAGTT.................................................. 212   | 212; |
| ...TGAGGTAGTAGGTTGTATANTT.................................................. 172   | 172; |
| ...TGAGGTAGTAGGNTGTATAGTTT................................................. 168   | 168; |
| ....GAGGTAGTAGGTTGTATAGTT.................................................. 158   | 158; |
| ...TGAGGTAGTAGGTTGTAT...................................................... 114   | 114; |
| ...TGAGGTAGTAGGTTGNATAGT................................................... 75   | 75; |
| ...TGAGGTAGTAGGTTGNATAGTTT................................................. 72   | 72; |
| ...TGAGGTAGTAGGTNNTATAGT................................................... 71   | 71; |
| ...TGAGGTAGTAGG............................................................ 63   | 63; |
| ...TGAGGTAGTAGGTNNTATAGTTT................................................. 44   | 44; |
| ...TGAGGTAGTAGGTTGTATANNT.................................................. 42   | 42; |
| ..GTGAGGTAGTAGGTTGTATAGT................................................... 37   | 37; |
| ...TGAGGTAGTAGGTTGTA....................................................... 35   | 35; |
| ...TGAGGTAGTAGGNTGTATAG.................................................... 34   | 34; |
| ....GAGGTAGTAGGTTGTATAGTTT................................................. 28   | 28; |
| ...TGAGGTAGTAGGTTGTATANT................................................... 28   | 28; |
| ....GAGGTAGTAGGTTGTATAGT................................................... 27   | 27; |
| ...TGAGGTAGTAGGTTNTATAGT................................................... 25   | 25; |
| ...TGAGGTAGTAGGTTNTATAGTTT................................................. 18   | 18; |
| ...TGAGGTAGTAGGTTGT........................................................ 17   | 17; |
| ..GTGAGGTAGTAGGTTGTATAGTT.................................................. 16   | 16; |
| ...TGAGGTAGTAGGTTGTATANTTT................................................. 15   | 15; |
| ...TGAGGTAGTAGGTTGTATAGTTTG................................................ 14   | 14; |
| .....AGGTAGTAGGTTGTATAGTT.................................................. 13   | 13; |
| ...TGAGGTAGTAGGTTGNATAG.................................................... 11   | 11; |
| ...TGAGGTAGTAGGTNNTATAG.................................................... 10   | 10; |
| ...TGAGGTAGTAGGTTGTATANNTT................................................. 9   | 9; |
| ...................................................CTATACAATCTACTGTCTTTC...\* 8   | 8; |
| .....AGGTAGTAGGTTGTATAGTTTG................................................ 8   | 8; |
| ..GTGAGGTAGTAGGTTGTATAG.................................................... 6   | 6; |
| ...TGAGGTAGTAGGTT.......................................................... 4   | 4; |
| ...TGAGGTAGTAGGTTG......................................................... 3   | 3; |
| ....GAGGTAGTAGGTTGTATAG.................................................... 3   | 3; |
| .....AGGTAGTAGGTTGTATAGT................................................... 2   | 2; |
| ......GGTAGTAGGTTGTATAGTT.................................................. 1   | 1; |
| -------------------------------------------------------------------------- 160033 |
| ...................................................CTATACAATCTACTGTCTTTCt.. 24   | 24; |
| -------------------------------------------------------------------------- 24 |
| -------------------------------------------------------------------------- 160057 |
| hsa-let-7a-5p(hsa-let-7a-1) TGGGATGAGGTAGTAGGTTGTATAGTTTTAGGGTCACACCCACCACTGGGAGATAACTATACAATCTACTGTCTTTCCTA (((((.(((..((((((((((((((((...(((.....))).((....))....))))))))))))))))..)))))))) (-35.60) \*\*\*\*\*TGAGGTAGTAGGTTGTATAGTT\*\*\*\*\*\*\*\*\*\*\*\*\*\*\*\*\*\*\*\*\*\*\*\*\*\*\*\*\*\*\*\*\*\*\*\*\*\*\*\*\*\*\*\*\*\*\*\*\*\*\*\*\* T  M |
| .....TGAGGTAGTAGGTTGTATAGTT......................................................\* 120200   | 120200; |
| .....TGAGGTAGTAGGTTGTATAGT....................................................... 19848   | 19848; |
| .....TGAGGTAGTAGGTTGTATAGTTT..................................................... 12180   | 12180; |
| .....TGAGGTAGTAGGTTGTATAG........................................................ 2746   | 2746; |
| .....TGAGGTAGTAGGNTGTATAGTT...................................................... 1804   | 1804; |
| .....TGAGGTAGTAGGTTGTATAGTTTT.................................................... 670   | 670; |
| .....TGAGGTAGTAGGTTGNATAGTT...................................................... 563   | 563; |
| .....TGAGGTAGTAGGTNNTATAGTT...................................................... 529   | 529; |
| .....TGAGGTAGTAGGTTGTATA......................................................... 322   | 322; |
| .....TGAGGTAGTAGGNTGTATAGT....................................................... 278   | 278; |
| .....TGAGGTAGTAGGTTNTATAGTT...................................................... 212   | 212; |
| .....TGAGGTAGTAGGTTGTATANTT...................................................... 172   | 172; |
| .....TGAGGTAGTAGGNTGTATAGTTT..................................................... 168   | 168; |
| ......GAGGTAGTAGGTTGTATAGTT...................................................... 158   | 158; |
| .....TGAGGTAGTAGGTTGTAT.......................................................... 114   | 114; |
| .....TGAGGTAGTAGGTTGNATAGT....................................................... 75   | 75; |
| .....TGAGGTAGTAGGTTGNATAGTTT..................................................... 72   | 72; |
| .....TGAGGTAGTAGGTNNTATAGT....................................................... 71   | 71; |
| .....TGAGGTAGTAGG................................................................ 63   | 63; |
| .....TGAGGTAGTAGGTNNTATAGTTT..................................................... 44   | 44; |
| .....TGAGGTAGTAGGTTGTATANNT...................................................... 42   | 42; |
| .....TGAGGTAGTAGGTTGTA........................................................... 35   | 35; |
| .....TGAGGTAGTAGGNTGTATAG........................................................ 34   | 34; |
| .....TGAGGTAGTAGGTTGTATANT....................................................... 28   | 28; |
| ......GAGGTAGTAGGTTGTATAGTTT..................................................... 28   | 28; |
| ......GAGGTAGTAGGTTGTATAGT....................................................... 27   | 27; |
| .....TGAGGTAGTAGGTTNTATAGT....................................................... 25   | 25; |
| .....TGAGGTAGTAGGTTNTATAGTTT..................................................... 18   | 18; |
| .....TGAGGTAGTAGGTTGT............................................................ 17   | 17; |
| .....TGAGGTAGTAGGTTGTATANTTT..................................................... 15   | 15; |
| .......AGGTAGTAGGTTGTATAGTT...................................................... 13   | 13; |
| .....TGAGGTAGTAGGTTGNATAG........................................................ 11   | 11; |
| .....TGAGGTAGTAGGTNNTATAG........................................................ 10   | 10; |
| .....TGAGGTAGTAGGTTGTATANNTT..................................................... 9   | 9; |
| ........................................................CTATACAATCTACTGTCTTTC.... 8   | 8; |
| ....ATGAGGTAGTAGGTTGTATAGTT...................................................... 8   | 8; |
| .....TGAGGTAGTAGGTT.............................................................. 4   | 4; |
| ......GAGGTAGTAGGTTGTATAG........................................................ 3   | 3; |
| .....TGAGGTAGTAGGTTG............................................................. 3   | 3; |
| .......AGGTAGTAGGTTGTATAGT....................................................... 2   | 2; |
| ........GGTAGTAGGTTGTATAGTT...................................................... 1   | 1; |
| -------------------------------------------------------------------------------- 160630 |
| .....TGAGGTAGTAGGTTGTATAGTTa..................................................... 14818   | 14818; |
| .....TGAGGTAGTAGGTTGTATAGTa...................................................... 1403   | 1403; |
| .....TGAGGTAGTAGGTTGTgTAGTT...................................................... 931   | 931; |
| .....TGAGGgAGTAGGTTGTATAGTT...................................................... 859   | 859; |
| .....TGAGGTAGTAGGTTGTATAGTaa..................................................... 842   | 842; |
| .....TGAGGTAGTAGtTTGTATAGTT...................................................... 800   | 800; |
| .....TGAGGTAGTAGGTTGTAcAGTT...................................................... 556   | 556; |
| .....aGAGGTAGTAGGTTGTATAGTT...................................................... 425   | 425; |
| .....TGAGGTAGTAGGgTGTATAGTT...................................................... 372   | 372; |
| .....TGAGGTAGTAGGTTGTATAGTTg..................................................... 359   | 359; |
| .....TGAGGTAGTAGGTTGgATAGTT...................................................... 289   | 289; |
| .....TGAGGTAGTAGGTTGTATAGTaT..................................................... 198   | 198; |
| .....TGAGGTAGTAGGTTGTATtGTT...................................................... 161   | 161; |
| .....TGAGGTAGTAGGTTGTATAGa....................................................... 148   | 148; |
| .....TGAGGTAGTAGGTTGTATAGgT...................................................... 139   | 139; |
| .....TGAGGTAGTAGGTTGTtTAGTT...................................................... 132   | 132; |
| .....TGAGGTAGgAGGTTGTATAGTTT..................................................... 130   | 130; |
| .....TGAGGTAGTAGGcTGTATAGTT...................................................... 129   | 129; |
| .....TGAGGTAGTAGGTTGTATAtTT...................................................... 128   | 128; |
| .....TGAGGgAGTAGGTTGTATAGT....................................................... 119   | 119; |
| .....TGAGGTAGTAGGTTGTgTAGTTT..................................................... 118   | 118; |
| .....TGAGGTAGTAGGTaNTATAGTT...................................................... 116   | 116; |
| .....TGAGGgAGTAGGTTGTATAGTTa..................................................... 105   | 105; |
| .....TGAGGTAGTAGGTTGTgTAGT....................................................... 103   | 103; |
| .....TGAGGTAGTAGGTTGTgTAGTTa..................................................... 100   | 100; |
| .....TGAGGcAGTAGGTTGTATAGTT...................................................... 99   | 99; |
| .....TGAGGgAGTAGGTTGTATAGTTT..................................................... 92   | 92; |
| .....TGAGGTAGTAGGTcNTATAGTT...................................................... 85   | 85; |
| .....TGAGGTAGTAGGgTGTATAGT....................................................... 77   | 77; |
| ....cTGAGGTAGTAGGTTGTATAGTT...................................................... 71   | 71; |
| .....TGAGGTAGTAGGNTGTATAGTTa..................................................... 69   | 69; |
| .....TGgGGTAGTAGGTTGTATAGTT...................................................... 59   | 59; |
| .....TGAGGTAGTAGGTTGTATAGaa...................................................... 58   | 58; |
| .....TGAGGTAGTAGGaTGTATAGTT...................................................... 57   | 57; |
| .....aGAGGTAGTAGGTTGTATAGTTT..................................................... 56   | 56; |
| .....TGAGGTAGTAGGgTGTATAGTTa..................................................... 56   | 56; |
| .....TGAGGTAGTAGGTTGTAgAGTT...................................................... 55   | 55; |
| .....TGAGGTAGTAGGTTGTcTAGTT...................................................... 51   | 51; |
| .....TGAGGTAGTAGGTTGTATAGTg...................................................... 48   | 48; |
| .....TGAGGTAGTAGGTTGgATAGT....................................................... 47   | 47; |
| .....TGAGGTAGTAGGgTGTATAGTTT..................................................... 43   | 43; |
| .....TGcGGTAGTAGGTTGTATAGTT...................................................... 42   | 42; |
| .....TGAGGTAGTAGGTTGTAcAGTTa..................................................... 42   | 42; |
| .....TGAGGTAGTAGGTTGTAcAGT....................................................... 41   | 41; |
| .....TGAGGTAGTAGtTTGTATAGTTa..................................................... 41   | 41; |
| .....TGAGGTAGTAGGTTGTATAGcT...................................................... 40   | 40; |
| .....TGAGGTAGTAGGTTGTA--GTT...................................................... 39   | 39; |
| ......GAGGTAGTAGGTTGTATAGTTa..................................................... 39   | 39; |
| .....TGAGGTAGTAGGTTGTATAGTTc..................................................... 39   | 39; |
| .....TGAGGTAGTAGGTTGNAaAGTT...................................................... 39   | 39; |
| .....aGAGGTAGTAGGTTGTATAGT....................................................... 38   | 38; |
| .....TGAGGgAGTAGGgTGTATAGTT...................................................... 37   | 37; |
| .....TGAGGTAGTAGGTTGgATAGTTa..................................................... 37   | 37; |
| .....cGAGGTAGTAGGTTGTATAGTT...................................................... 37   | 37; |
| .....TGAGGTAGTAGGTTGTATcGTT...................................................... 37   | 37; |
| .....TGAGGTgGTAGGTTGTATAGTT...................................................... 35   | 35; |
| .....TGAGGTAGTAGGTTtTATAGTT...................................................... 35   | 35; |
| .....TGAGGTAGTAGtTTGTATAGT....................................................... 34   | 34; |
| .....TGAGGTAGTAGcTTGTATAGTT...................................................... 32   | 32; |
| .....aGAGGTAGTAGGTTGTATAGTTa..................................................... 30   | 30; |
| .....TGAGGTAGTAGGTgGTATAGTT...................................................... 29   | 29; |
| .....TGAGGTAGTAGGTTGNAcAGTT...................................................... 28   | 28; |
| .....TGAGGTAGTAGGTTGgATAGTTT..................................................... 28   | 28; |
| .....TGAGGTAGTAGGTTGTATAGg....................................................... 28   | 28; |
| .....TGAGGTAGTAGtTTGTATAGTTT..................................................... 28   | 28; |
| .....TGAGGTAGTgGGTTGTATAGTT...................................................... 27   | 27; |
| .....TGAGGTAGTAGGTgNTATAGTT...................................................... 26   | 26; |
| .....TGAGGTAGTAGGTTGTATAGTc...................................................... 26   | 26; |
| .....TGAGGTAGTAGGTTGTAcAGTTT..................................................... 26   | 26; |
| .....TGAGGTAGTAGGTTGTATAGaT...................................................... 24   | 24; |
| .....TGAGGTAGcAGGTTGTATAGTT...................................................... 24   | 24; |
| .....TGAGGaAGTAGGTTGTATAGTT...................................................... 24   | 24; |
| .....TGAGGTAGTAGGTcGTATAGTT...................................................... 24   | 24; |
| .....TGAGGTAGTAGGTTGTATAtT....................................................... 24   | 24; |
| .....TGAGGTAGTAGGcTGTATAGT....................................................... 24   | 24; |
| .....TGAGGTAGTAGGTTGTATtGT....................................................... 23   | 23; |
| .....TGAGGTAGTAG-TTGTATAGTT...................................................... 23   | 23; |
| .....TGAGGTAGTAGGTTGaATAGTT...................................................... 22   | 22; |
| .....TGAGGTAGTAGGTTGNATAGTTa..................................................... 21   | 21; |
| .....TGAGGTcGTAGGTTGTATAGTT...................................................... 20   | 20; |
| .....TGAGGTAGTAGGTT--ATAGTT...................................................... 20   | 20; |
| .....TGAGGTAGTAGGTTGTtTAGTTa..................................................... 19   | 19; |
| .....TGAGGTAGTAGGTTGTATtGTTT..................................................... 19   | 19; |
| .....TGAGGTAGTAGGTTGTATtGTTa..................................................... 19   | 19; |
| .....TGAGGTAGTAGGTTGTATAGTga..................................................... 19   | 19; |
| .....TGAGGTAGTAGGT-GTATAGTT...................................................... 18   | 18; |
| .....TGAGGTAGTAGGcTGTATAGTTa..................................................... 18   | 18; |
| .....TGAGGgAGTAGGTTGgATAGTT...................................................... 18   | 18; |
| .....TGAGGTAGTAGGTTGTtTAGT....................................................... 18   | 18; |
| .....TGAGGTAGTA--TTGTATAGTT...................................................... 17   | 17; |
| .....TGAGGgAGTAGGTTGTgTAGTT...................................................... 17   | 17; |
| .....TGAGGTAGTAGGTTGNAgAGTT...................................................... 17   | 17; |
| .....TGAGGTAGTcGGTTGTATAGTT...................................................... 17   | 17; |
| .....TGAGGTAGTAGGTTGTATAGgTT..................................................... 16   | 16; |
| .....TGAGGTAGTAGGTTGNcTAGTT...................................................... 16   | 16; |
| .....TGAGGTAGTAGGTTcTATAGTT...................................................... 16   | 16; |
| .....TGAGGTAGTAGGgTGTATAGgT...................................................... 15   | 15; |
| .....TGAGGTAGTAGGTTGTgcAGTT...................................................... 15   | 15; |
| .....TGAGGTAGTAGGTNNTATAGTTa..................................................... 15   | 15; |
| .....TtAGGTAGTAGGTTGTATAGTT...................................................... 15   | 15; |
| .....TGAGGcAGTAGGTTGTATAGT....................................................... 14   | 14; |
| .....TGAGGTAGTAGGTTGTATAGgTa..................................................... 14   | 14; |
| .....TGAGGTAGTAGGgTGgATAGTT...................................................... 14   | 14; |
| .....TGAGGTAGTAGGTTGTATAcTT...................................................... 14   | 14; |
| .....TGAGGTAtTAGGTTGTATAGTT...................................................... 13   | 13; |
| .....TGAGGTAGTAGGTTaTATAGTT...................................................... 13   | 13; |
| .....TGAGGTAGTAGGTcNTATAGT....................................................... 13   | 13; |
| .....TGAGGgAGTAGGTTGTATAGTa...................................................... 13   | 13; |
| .....TGAGGTAGTAGGTTGTATAGTgT..................................................... 13   | 13; |
| .....TGAGGTAGTAGGTaNTATAGT....................................................... 13   | 13; |
| .....TGgGGTAGTAGGTTGTATAGT....................................................... 13   | 13; |
| .....TGAGGTAGTAGGTTGTATAaa....................................................... 12   | 12; |
| .....TGAGGTAGTAGGcTGTATAGTTT..................................................... 12   | 12; |
| .....TGAGGTAGTAGGTTGTATAGTag..................................................... 12   | 12; |
| .....TGAGGcAGTAGGTTGTATAGTTT..................................................... 12   | 12; |
| .....TGAGGTAGTAGGTTGTtTAGTTT..................................................... 12   | 12; |
| .....TGAGGTAGTAGGTTGTcTAGT....................................................... 11   | 11; |
| .....TGAGGTAGTAGGTTGTAT-GTTT..................................................... 11   | 11; |
| .....TGAGGT--TAGGTTGTATAGTT...................................................... 11   | 11; |
| ....cTGAGGTAGTAGGTTGTATAGT....................................................... 11   | 11; |
| .....TGAGGTAGTAGGaTGTATAGT....................................................... 11   | 11; |
| .....TGAGGTAGTAGGTTGTA--GTTT..................................................... 10   | 10; |
| .....TGAGGcAGTAGGTTGTATAGTTa..................................................... 10   | 10; |
| .....TGgGGTAGTAGGTTGTATAGTTT..................................................... 10   | 10; |
| .....TGAGGTAGTAGGTaNTATAGTTT..................................................... 10   | 10; |
| .....TGAGGTAGTAGGTaNTATAGTTa..................................................... 10   | 10; |
| .....TGAGGTAGTAGGTTGTATAtTTT..................................................... 10   | 10; |
| .....TGAGGTAcTAGGTTGTATAGTT...................................................... 9   | 9; |
| .....TGAGGgAGTAGGTTGTAcAGTT...................................................... 9   | 9; |
| .....TGAGGgAGTAGGTTGaATAGTT...................................................... 9   | 9; |
| .....TGAGGTAGTAGGaTGTATAGTTa..................................................... 9   | 9; |
| .....TGAG-TAGTAGGTTGTATAGTT...................................................... 9   | 9; |
| .....TGAGGTAGTAGGTTGTcTAGTTa..................................................... 9   | 9; |
| .....TGAtGTAGTAGGTTGTATAGTT...................................................... 9   | 9; |
| .....gGAGGTAGTAGGTTGTATAGTT...................................................... 9   | 9; |
| .....TGAGGTAGTAGGNTGTATAGTa...................................................... 8   | 8; |
| .....TGAGGTAGTAGGTTGTAgAGT....................................................... 8   | 8; |
| .....TGAGGTAGTAGGTTGggTAGTT...................................................... 8   | 8; |
| .....TGAGGTAGTAGGTcNTATAGTTa..................................................... 8   | 8; |
| .....TGAGGTAGTAGGTTGNgTAGTT...................................................... 8   | 8; |
| .....TGAGGTAGTAGGTTGTAgAGTTT..................................................... 8   | 8; |
| .....aGAGGTAGTAGGTTGTATAGTa...................................................... 8   | 8; |
| .....TGAGGTAGTAG-TTGTATAGTTa..................................................... 8   | 8; |
| .....TGAGGTAGTAGGTTGNAaAGTTa..................................................... 8   | 8; |
| .....TGgGGTAGTAGGTTGTATAGTTa..................................................... 7   | 7; |
| .....TGAGGTAGTAGGTTGTATAaT....................................................... 7   | 7; |
| .....TGAGGTAGTAGGTTGTATAaTT...................................................... 7   | 7; |
| .....TGAGGTAGTAtGTTGTATAGTT...................................................... 7   | 7; |
| .....TGAGGgAGTAGGgTGTATAGT....................................................... 7   | 7; |
| .....TGAGGTAGTAGGTTGTgTAGTa...................................................... 7   | 7; |
| .....TGAGGTAGTAGGTTGTATAGcTT..................................................... 6   | 6; |
| .....TGcGGTAGTAGGTTGTATAGT....................................................... 6   | 6; |
| .....TGAGGTAGTgGGTTGTATAGT....................................................... 6   | 6; |
| .....TGAGGgAGTAGGgTGTATAGTTT..................................................... 6   | 6; |
| .....TGAGGTgGTAGGTTGTATAGTTT..................................................... 6   | 6; |
| .....TGAGGTgGTAGGTTGTATAGT....................................................... 6   | 6; |
| .....TGAGGTAGTAGGTTGTATAGc....................................................... 6   | 6; |
| .....TGAGGcAGTAGGTTGgATAGTT...................................................... 6   | 6; |
| .....TGAGGTAGTAGGTTNTATAGTTa..................................................... 6   | 6; |
| .....TGAGGgAGTAGtTTGTATAGTT...................................................... 6   | 6; |
| .....TaAGGTAGTAGGTTGTATAGTT...................................................... 6   | 6; |
| .....TGAGGTcGTAGGTTGTATAGT....................................................... 6   | 6; |
| .....TGAGGgAGTAGGTTGTATAGcT...................................................... 6   | 6; |
| .....TGAGGTAGTAGGTTGTtTtGTT...................................................... 6   | 6; |
| .....TGAGGTAGaAGGTTGTATAGTT...................................................... 6   | 6; |
| .....TGAGGTAGTAGGTTGTATANTTa..................................................... 6   | 6; |
| .....TGAGGTAGTAGGTTGTgTAGTTg..................................................... 6   | 6; |
| .....TGAGGTAGTAGGTTGgATAGgT...................................................... 6   | 6; |
| .....TGAGGTAGTAGGTT-TATAGTT...................................................... 6   | 6; |
| .....TGAGGTAGTAGGTgGTATAGT....................................................... 6   | 6; |
| .....TGAGGTAGTAGGTTGNAaAGTTT..................................................... 5   | 5; |
| .....TGAGGTAGTAGGTTGaATAGT....................................................... 5   | 5; |
| .....TGAGGTAGTAGGTTGNcaAGTT...................................................... 5   | 5; |
| .....TGAGGTAGTAGGTTGNAaAGT....................................................... 5   | 5; |
| .....TGAGGTAGTAaGTTGTATAGTT...................................................... 5   | 5; |
| .....TGAGGTAGTAGtTTGTATtGTT...................................................... 5   | 5; |
| .....TGAGGTAGTAGGaTGTATAGTTT..................................................... 5   | 5; |
| .....TGAaGTAGTAGGTTGTATAGTT...................................................... 5   | 5; |
| .....aGAGGTAGTAGGTTGTgTAGTT...................................................... 5   | 5; |
| .....TGAGGTAGTAGGNTGTAcAGTT...................................................... 5   | 5; |
| .....TGAGGTAGTAGGTTtTATAGTTa..................................................... 5   | 5; |
| .....cGAGGTAGTAGGTTGTATAGT....................................................... 5   | 5; |
| .....TGAGGTAGTAGGTTGTATAGTca..................................................... 5   | 5; |
| .....TGAGGTAGTAGGTTGTcTAGTTT..................................................... 5   | 5; |
| .....TGAGGgAGTAGcTTGTATAGTT...................................................... 5   | 5; |
| .....TGAGGTAGTAGtTTGTgTAGTT...................................................... 5   | 5; |
| .....TGAGGTAGTAGGT-GTATAGTTa..................................................... 5   | 5; |
| .....TGAGGTAGTAGGTaGTATAGTT...................................................... 5   | 5; |
| .....TGAGGTAGTAGGTcGTATAGT....................................................... 5   | 5; |
| .....TGAGGTAaTAGGTTGTATAGTT...................................................... 5   | 5; |
| .....TGAGGTAGTAGGTTGTATcGTTa..................................................... 5   | 5; |
| .....TGAGGTAGgAGGgTGTATAGTTT..................................................... 5   | 5; |
| .....TGAGGcAGTAGGgTGTATAGTT...................................................... 5   | 5; |
| .....TGAGGTAGTAGGTaNTgTAGTT...................................................... 5   | 5; |
| .....TGAGGTAGTAGGcTGgATAGTT...................................................... 5   | 5; |
| .....TGAGGTAGTAGGNTGTgTAGTT...................................................... 5   | 5; |
| .....TGAGGTAGTAGGTTtTATAGT....................................................... 5   | 5; |
| .....TGcGGTAGTAGGTTGTATAGTTa..................................................... 5   | 5; |
| .....TGAGGTAGTAGGTTtTATAGTTT..................................................... 4   | 4; |
| .....TGAGGTAGTAGGTgNTATAGT....................................................... 4   | 4; |
| .....TGAGGcAGTAGGTTGTgTAGTT...................................................... 4   | 4; |
| .....TGAGGgAGTAGGaTGTATAGTTT..................................................... 4   | 4; |
| .....TGAGGTAGTAGGTTGTgTAtTT...................................................... 4   | 4; |
| .....TGAGGcAGTAGGTTGTATAGgT...................................................... 4   | 4; |
| .....TGAGGTAGTAGGTTcTATAGT....................................................... 4   | 4; |
| .....TGAGGTAGTAGGgTGTgTAGTT...................................................... 4   | 4; |
| .....TGAGGTAGTAGGTTGNtTAGTT...................................................... 4   | 4; |
| .....TGAGGTAGcAGGTTGTATAGTTa..................................................... 4   | 4; |
| .....TGAGGTAGTAGGTTGTAgAGgT...................................................... 4   | 4; |
| .....TGAGGTAGcAGGTTGTATAGT....................................................... 4   | 4; |
| .....TGAGGTAGTAGGTTGTATcGT....................................................... 4   | 4; |
| .....TGAGGTAGTAGGTgGTATAGTTa..................................................... 4   | 4; |
| .....TGAGGTAGTAGGTTGTAgAGTTa..................................................... 4   | 4; |
| .....TGAGGTAGTAGGTTGTAcAGTa...................................................... 4   | 4; |
| .....TGAcGTAGTAGGTTGTATAGTT...................................................... 4   | 4; |
| .....TGAGGTgGTAGGTTGTATAGTTa..................................................... 4   | 4; |
| .....TGAGtTAGTAGGTTGTATAGTT...................................................... 4   | 4; |
| .....TGAGGgAGTAGGTTGTAgAGTT...................................................... 4   | 4; |
| .....cGAGGTAGTAGGTTGTATAGTTa..................................................... 4   | 4; |
| .....TGAGGTAGTAGGgTGTATAGTa...................................................... 4   | 4; |
| .....TGcGGTAGTAGGTTGTATAGTTT..................................................... 4   | 4; |
| .....TGAGGTAGTAGGTcNTATAGTTT..................................................... 4   | 4; |
| .....aGAGGgAGTAGGTTGTATAGTT...................................................... 4   | 4; |
| .....TGAGGaAGTAGGTTGTATAGT....................................................... 4   | 4; |
| .....TGAGGgAGTgGGTTGTATAGTT...................................................... 3   | 3; |
| .....TGAGGTAGTAGGTTGTATAGaTT..................................................... 3   | 3; |
| .....TGAGGTAGTAGGTTGTtTAGgT...................................................... 3   | 3; |
| .....TGAGGTAGcAGGTTGTATAGTTT..................................................... 3   | 3; |
| .....TGAGGTAGTAGGTTGNcTAGT....................................................... 3   | 3; |
| .....TGAGGgAGTAGGTTGTtTAGTT...................................................... 3   | 3; |
| .....TGgGGTAGTAGGTTGTgTAGTT...................................................... 3   | 3; |
| .....TGAGGTAGTAGtTTGNATAGTT...................................................... 3   | 3; |
| .....TGAGGTAGTAGGTTaTATAGT....................................................... 3   | 3; |
| .....TGAGGTAGTAGGTgNTATAGTTa..................................................... 3   | 3; |
| .....TGAGGTAGTAGGTgGTATAGTTT..................................................... 3   | 3; |
| .....TGAGGTAGTAGGTTGTtTAtTT...................................................... 3   | 3; |
| .....TGAGGTAGTAGcTTGTATAGTTT..................................................... 3   | 3; |
| .....TGAGGTAGTAGGggGTATAGTT...................................................... 3   | 3; |
| .....TGAGGTAGTAGGTTGTAaAGTT...................................................... 3   | 3; |
| .....TGAGGTAGTAGGTTGNAcAGTTa..................................................... 3   | 3; |
| .....TGAGGTAGTAGGgTGTATAGg....................................................... 3   | 3; |
| .....TGAGGTAGTgGGTTGTATAGTTT..................................................... 3   | 3; |
| .....TGAGGgAGTAGGTTGgATAGT....................................................... 3   | 3; |
| .....TGAGGTAGTAcGTTGTATAGTT...................................................... 3   | 3; |
| .....TGAGGTAGTAGGgTGTtTAGTT...................................................... 3   | 3; |
| .....TGAGGTAGTAGGNaGTATAGTT...................................................... 3   | 3; |
| .....TGAGGTAGTAGGTTGNAgAGTTa..................................................... 3   | 3; |
| .....TGAGGTAGTAGGTTGTATAGTcT..................................................... 3   | 3; |
| .....aGAGGTAGTAGGNTGTATAGTT...................................................... 3   | 3; |
| .....TGAGGTAGTAGtNTGTATAGTT...................................................... 3   | 3; |
| .....gGAGGTAGTAGGTTGTATAGT....................................................... 3   | 3; |
| .....TGAGGTAGTAGGTTGNAcAGT....................................................... 3   | 3; |
| .....TtAGGTAGTAGGTTGTATAGT....................................................... 3   | 3; |
| .....TGAGGTAGTAGGTTGTgcAGTTT..................................................... 3   | 3; |
| .....TGAGGTAGTgGGTTGTATAGTTa..................................................... 3   | 3; |
| .....TGAGGTAGTtGGTTGTATAGTT...................................................... 3   | 3; |
| .....TGAGGTAGTAGGNTGTATAGTaa..................................................... 3   | 3; |
| .....TGgGGTAGTAGGTTGTAgAGTT...................................................... 3   | 3; |
| .....TGAGGTAGTAGGTTGTATAGga...................................................... 3   | 3; |
| .....TGAGGTAGTAGGTcGTATAGTTa..................................................... 3   | 3; |
| .....TGtGGTAGTAGGTTGTATAGTT...................................................... 3   | 3; |
| .....TGAGGTtGTAGGTTGTATAGTT...................................................... 3   | 3; |
| .....TGAGGTAGTcGGTTGTATAGTTT..................................................... 3   | 3; |
| .....TGAGGTAGTAGtTTGTATAGTa...................................................... 3   | 3; |
| .....TGAGGgAGTAGGTTGTATAGgT...................................................... 3   | 3; |
| .....TGAGGTAGTAGGNTGTATAGgT...................................................... 3   | 3; |
| .....cGAGGTAGTAGGTTGTATAGTTT..................................................... 3   | 3; |
| .....TGAGGTAGTAGcTTGTATAGT....................................................... 3   | 3; |
| .....TGAGGgAGTAGGTTGTATtGTT...................................................... 3   | 3; |
| .....TGAGGTAGTAGGaTGaATAGTT...................................................... 2   | 2; |
| .....TGAGGTAGTAGGTTGTATAtGT...................................................... 2   | 2; |
| .....TGAGGTAGTAGGTTGgATtGTT...................................................... 2   | 2; |
| .....TGAGGTAGTAGGNTcTATAGTT...................................................... 2   | 2; |
| .....TGAGGTAGTAGGTTGNAcAGTTT..................................................... 2   | 2; |
| .....TGAGGTAGTAGGTTGNATAGTa...................................................... 2   | 2; |
| .....TGAGGTAGTAGGTTGaATAGTTT..................................................... 2   | 2; |
| .....TGAGGTAGTAGGTTGTATcGTTT..................................................... 2   | 2; |
| .....TGAGGTAGTAGGTTGTATAGcTa..................................................... 2   | 2; |
| .....TGAGGTAGTAGGNTGTATAGTTg..................................................... 2   | 2; |
| .....TGAGGgAGTAGGTTGTATAGTTg..................................................... 2   | 2; |
| .....TGAGGcAGTAGGcTGTATAGTT...................................................... 2   | 2; |
| .....TGAGaTAGTAGGTTGTATAGTT...................................................... 2   | 2; |
| .....TGgGGTAGTAGGTTGTtTAGTT...................................................... 2   | 2; |
| .....TGAGGTAGTAGGTNNTATAGTa...................................................... 2   | 2; |
| .....TGAGGTAGTAGGTTGNAaAGTa...................................................... 2   | 2; |
| .....TGAGGgAGTAGGcTGTATAGTT...................................................... 2   | 2; |
| .....TGAGGTAGTAGGTTGaATAGTTa..................................................... 2   | 2; |
| .....TGAGGaAGTAGGTTGTATAGTTT..................................................... 2   | 2; |
| ......GAGGTAGTAGGTTGTATAGTa...................................................... 2   | 2; |
| .....TGAGGTcGTAGGTTGTATAGTTT..................................................... 2   | 2; |
| .....aGAGGTAGTAGGTTGTATAtTT...................................................... 2   | 2; |
| .....TGAGGTAGTAGGgTGTATAGgTT..................................................... 2   | 2; |
| .....TGAGGTAGTAGGTTGTActGTT...................................................... 2   | 2; |
| .....TGAGGgAGTAGGTTGTATAGTg...................................................... 2   | 2; |
| .....TGAGGTAGTAGGTTGTgTAGTaT..................................................... 2   | 2; |
| .....TGAGGTAGTAGGgTGgATAGTTT..................................................... 2   | 2; |
| .....TGAGGTAGTAGGTTGTcTAtTT...................................................... 2   | 2; |
| .....TGAGGTAGTAtGTTGTATAGTTa..................................................... 2   | 2; |
| .....TGAGGTAGTAGGTTGgATAtTT...................................................... 2   | 2; |
| .....aGAGGTAGTAGGgTGTATAGTT...................................................... 2   | 2; |
| .....TGAGGTAGTAGGTaNTcTAGTT...................................................... 2   | 2; |
| .....TGAtGTAGTAGGTTGTATAGT....................................................... 2   | 2; |
| .....TGAGGgAGTAGGTTGgATAGTTT..................................................... 2   | 2; |
| .....TGAGGTAGTAGGcTGTATAGgT...................................................... 2   | 2; |
| .....aGAGGTAGTAGGTTGTATAGTaT..................................................... 2   | 2; |
| .....TGAGGTAGTAGcTTGTATAGTTa..................................................... 2   | 2; |
| .....TGAGGaAGTAGGTTGTATAGTTa..................................................... 2   | 2; |
| .....TGAGGTAGTAGGTTGTATAtTa...................................................... 2   | 2; |
| .....TGAGGTAGTAGGgTGgATAGT....................................................... 2   | 2; |
| .....TGAGGTAGTAGGNTGTATAGaT...................................................... 2   | 2; |
| .....TGAGGTcGTAGGTTGTATAGTTa..................................................... 2   | 2; |
| .....TGAGGgAGTAGGTTGTATAGTaT..................................................... 2   | 2; |
| .....TGAGGTAGTAGGTTcTATAGTTa..................................................... 2   | 2; |
| .....TGAGGTAGTAGGTTGggTAGTTT..................................................... 2   | 2; |
| .....TGAGGTAGTAGGcTGTtTAGTT...................................................... 2   | 2; |
| .....TGAGGTAGTcGGTTGTATAGTTa..................................................... 2   | 2; |
| .....TGAGGTAGTAGGcTGTgTAGTT...................................................... 2   | 2; |
| .....TGAGGgAGTAGGNTGTATAGTT...................................................... 2   | 2; |
| .....aGAGGTAGTAGGTTGTcTAGTT...................................................... 2   | 2; |
| .....TGAGcTAGTAGGTTGTATAGTT...................................................... 2   | 2; |
| .....TGAGGcAGTAGGTTGTAgAGTT...................................................... 2   | 2; |
| .......AGGTAGTAGGTTGTATAGTTa..................................................... 2   | 2; |
| .....TGAGGTAGTcGGTTGTATAGT....................................................... 2   | 2; |
| .....TGAGGTAGTAGGTTGNgTAGT....................................................... 2   | 2; |
| .....TGAGGTAGTAGGgTGTATAGTTg..................................................... 2   | 2; |
| .....TGAGGgAGaAGGTTGTATAGTT...................................................... 2   | 2; |
| .....TGAGGggGTAGGTTGTATAGTT...................................................... 2   | 2; |
| .....TGAGGTAGTAGGTTGgATAGTg...................................................... 2   | 2; |
| .....TGAGGTAGTAGGTNNTgTAGTT...................................................... 2   | 2; |
| .....TGAGGgAGTAGGTTGTATAtTT...................................................... 2   | 2; |
| .....TGAGGTAGTAGGTaNTATAGTa...................................................... 2   | 2; |
| .....TGAGGTAGTAGGTTGgAcAGTT...................................................... 2   | 2; |
| .....TGAGGTAGTAGGTTGNcTAGTTa..................................................... 2   | 2; |
| .....TGAGGTAGTAGGTTGNAgAGTTT..................................................... 2   | 2; |
| .....TGAGGTAGTAGGcTGTATAGTa...................................................... 2   | 2; |
| .....TGAGGTAGTAGGNTGgATAGTT...................................................... 2   | 2; |
| .....TcAGGTAGTAGGTTGTATAGTT...................................................... 2   | 2; |
| .....TGAGGTAtTAGGTTGTATAGTTa..................................................... 2   | 2; |
| .....TGAGGTAGTAGGTTGTATAGag...................................................... 1   | 1; |
| .....TGAGGTAGTAGGTTGgtTAGTT...................................................... 1   | 1; |
| .....gGAGGTAGTAGGTTGTATAGTTT..................................................... 1   | 1; |
| .....TGAGGTAGTAGtTTGTATAtTT...................................................... 1   | 1; |
| .....TGAGGgAGTAGGTTGTgTAGT....................................................... 1   | 1; |
| .....TGAGGTAGTAGGTTGTAcAGgT...................................................... 1   | 1; |
| .....TGAGGTAGTAGcTTGTgTAGTT...................................................... 1   | 1; |
| .....TGAGGTAGTAGtTTGgATAGTT...................................................... 1   | 1; |
| .....TGAGGTAGTAGGTcGTATAGTTT..................................................... 1   | 1; |
| .....TGAGGTAGTAGGTTGNgTAGTTa..................................................... 1   | 1; |
| .....TGAGGTAGTAGGNTGTATAGTaT..................................................... 1   | 1; |
| .....TGAGGTAGTAGGTTGNtcAGTT...................................................... 1   | 1; |
| .....TGAGGTAGTAGGTTGTAcAGTTg..................................................... 1   | 1; |
| .....TGAGGaAGTAGGNTGTATAGTT...................................................... 1   | 1; |
| .....gGAGGTAGTAGGTTGTATAGTTa..................................................... 1   | 1; |
| .....TGAGGTAGTAGGTTGTtgAGTT...................................................... 1   | 1; |
| .....TGAGGTAGTAGGTTGTATAcTa...................................................... 1   | 1; |
| .....TGAGGTAGTAGtTTNTATAGTT...................................................... 1   | 1; |
| .....TGAGGTAGTAGGNTGaATAGTT...................................................... 1   | 1; |
| .....TGAGGgAGTAGGTTGTATcGTT...................................................... 1   | 1; |
| .....TGAGGTAGTAGtTTGTATcGTT...................................................... 1   | 1; |
| .....TGcGGTAGTAGGgTGTATAGTT...................................................... 1   | 1; |
| .....TGAGGTAGTAGGTTGNAgAGT....................................................... 1   | 1; |
| .....TGAGGaAGTAGGTTGTAgAGTT...................................................... 1   | 1; |
| .....TGAGGTAGTAGGcTGTATtGTT...................................................... 1   | 1; |
| .....TGAGGgAGgAGGTTGTATAGTTT..................................................... 1   | 1; |
| .....TGAGGTAGTAGtTaNTATAGTT...................................................... 1   | 1; |
| .....TGAGGgAGTAGGTTtTATAGTT...................................................... 1   | 1; |
| .....TGAGGTAGTAGGTTGNgcAGTT...................................................... 1   | 1; |
| .....TGAGGTAGTAGtTTGTATAGgT...................................................... 1   | 1; |
| .....TGgGGTAGTAGGTTGTATtGTT...................................................... 1   | 1; |
| .....TGAGGTAGTAGGgTGTATcGTT...................................................... 1   | 1; |
| .....TGgGGgAGTAGGTTGTATAGTT...................................................... 1   | 1; |
| .....TGAGGTAGTAGGTTGgATAGTa...................................................... 1   | 1; |
| .....aGAGGTAGTAGGTTGTATtGTT...................................................... 1   | 1; |
| .....TGAGGTAGTAGGTTNTgTAGTT...................................................... 1   | 1; |
| .....TGAGGgAGTAGGTTGTATAGcTT..................................................... 1   | 1; |
| .....TGAGGTAGTAGGTTGNgTAGTTT..................................................... 1   | 1; |
| .....TGAGGTAGTAGGTcNTgTAGTT...................................................... 1   | 1; |
| .....TGAGGTAGTAGGaTGTgTAGTT...................................................... 1   | 1; |
| .....TGAGGTAGTAGGTTGNccAGTT...................................................... 1   | 1; |
| .....TGAGGTAGTAGGTTGTAgtGTT...................................................... 1   | 1; |
| .....TGAGGTAGTAGGaTGTATAGcT...................................................... 1   | 1; |
| ......GAGGTAGTAGGTTGTATAGTaT..................................................... 1   | 1; |
| ....cTGAGGTAGTAGNTTGTATAGTT...................................................... 1   | 1; |
| .....TGAGGTAGTAGGTTGggTAGT....................................................... 1   | 1; |
| .....aGAGGTAGTAGGTTGTATAGTTg..................................................... 1   | 1; |
| .....aGAGGTAGTAGGTTGTtTAGTT...................................................... 1   | 1; |
| .....TGAGGTAGTAGtTNNTATAGTT...................................................... 1   | 1; |
| .....TGAGGTAGTAGGTTGNgaAGTT...................................................... 1   | 1; |
| .....TGAGGTAGTAGGTTGTgTAGaT...................................................... 1   | 1; |
| .....TGAGGTAGgAGGTTGTgTAGTTT..................................................... 1   | 1; |
| .....TGAGGgAGTAGGTTGTgTAGTTT..................................................... 1   | 1; |
| .....TGgGGTAGTAGGTTGgATAGTT...................................................... 1   | 1; |
| .....TGAGGTAGTAGGTTGTATtGTa...................................................... 1   | 1; |
| .....TGAGGTAGTgGGTTGTgTAGTT...................................................... 1   | 1; |
| .....TGAGGTAGTAGGTTGTATctTT...................................................... 1   | 1; |
| .....TGAGGTAGTAGGTTGTgcAGT....................................................... 1   | 1; |
| .....TGcGGgAGTAGGTTGTATAGTT...................................................... 1   | 1; |
| .....TGAGGTAGTAGGgTGTATtGTT...................................................... 1   | 1; |
| .....TGAGGgAGTAGGTTGTATtGT....................................................... 1   | 1; |
| .....TGAGGTAGTAGGTTGTtTtGTTT..................................................... 1   | 1; |
| -------------------------------------------------------------------------------- 27267 |
| -------------------------------------------------------------------------------- 187897 |
| hsa-let-7a-5p(hsa-let-7a-2) AGGTTGAGGTAGTAGGTTGTATAGTTTAGAATTACATCAAGGGAGATAACTGTACAGCCTCCTAGCTTTCCT (((..(((.(((.(((((((((((((.....(..(.....)..)...))))))))))))).))).))).))) (-25.20) \*\*\*\*TGAGGTAGTAGGTTGTATAGTT\*\*\*\*\*\*\*\*\*\*\*\*\*\*\*\*\*\*\*\*\*\*\*\*\*\*\*\*\*\*\*\*\*\*\*\*\*\*\*\*\*\*\*\*\*\* T  M |
| ....TGAGGTAGTAGGTTGTATAGTT...............................................\* 120200   | 120200; |
| ....TGAGGTAGTAGGTTGTATAGT................................................ 19848   | 19848; |
| ....TGAGGTAGTAGGTTGTATAGTTT.............................................. 12180   | 12180; |
| ....TGAGGTAGTAGGTTGTATAG................................................. 2746   | 2746; |
| ....TGAGGTAGTAGGNTGTATAGTT............................................... 1804   | 1804; |
| ....TGAGGTAGTAGGTTGNATAGTT............................................... 563   | 563; |
| ....TGAGGTAGTAGGTNNTATAGTT............................................... 529   | 529; |
| ....TGAGGTAGTAGGTTGTATAGTTTA............................................. 410   | 410; |
| ....TGAGGTAGTAGGTTGTATA.................................................. 322   | 322; |
| ....TGAGGTAGTAGGNTGTATAGT................................................ 278   | 278; |
| ....TGAGGTAGTAGGTTNTATAGTT............................................... 212   | 212; |
| ....TGAGGTAGTAGGTTGTATANTT............................................... 172   | 172; |
| ....TGAGGTAGTAGGNTGTATAGTTT.............................................. 168   | 168; |
| .....GAGGTAGTAGGTTGTATAGTT............................................... 158   | 158; |
| ....TGAGGTAGTAGGTTGTAT................................................... 114   | 114; |
| ....TGAGGTAGTAGGTTGNATAGT................................................ 75   | 75; |
| ....TGAGGTAGTAGGTTGNATAGTTT.............................................. 72   | 72; |
| ....TGAGGTAGTAGGTNNTATAGT................................................ 71   | 71; |
| ....TGAGGTAGTAGG......................................................... 63   | 63; |
| ....TGAGGTAGTAGGTNNTATAGTTT.............................................. 44   | 44; |
| ....TGAGGTAGTAGGTTGTATANNT............................................... 42   | 42; |
| ....TGAGGTAGTAGGTTGTA.................................................... 35   | 35; |
| ....TGAGGTAGTAGGNTGTATAG................................................. 34   | 34; |
| ....TGAGGTAGTAGGTTGTATANT................................................ 28   | 28; |
| .....GAGGTAGTAGGTTGTATAGTTT.............................................. 28   | 28; |
| .....GAGGTAGTAGGTTGTATAGT................................................ 27   | 27; |
| ....TGAGGTAGTAGGTTNTATAGT................................................ 25   | 25; |
| ...TTGAGGTAGTAGGTTGTATAGT................................................ 25   | 25; |
| ....TGAGGTAGTAGGTTNTATAGTTT.............................................. 18   | 18; |
| ....TGAGGTAGTAGGTTGT..................................................... 17   | 17; |
| ....TGAGGTAGTAGGTTGTATANTTT.............................................. 15   | 15; |
| ......AGGTAGTAGGTTGTATAGTT............................................... 13   | 13; |
| ....TGAGGTAGTAGGTTGNATAG................................................. 11   | 11; |
| ....TGAGGTAGTAGGTNNTATAG................................................. 10   | 10; |
| ....TGAGGTAGTAGGTTGTATANNTT.............................................. 9   | 9; |
| ...TTGAGGTAGTAGGTTGTATAG................................................. 5   | 5; |
| ....TGAGGTAGTAGGTT....................................................... 4   | 4; |
| .....GAGGTAGTAGGTTGTATAG................................................. 3   | 3; |
| ....TGAGGTAGTAGGTTG...................................................... 3   | 3; |
| ......AGGTAGTAGGTTGTATAGT................................................ 2   | 2; |
| .......GGTAGTAGGTTGTATAGTT............................................... 1   | 1; |
| ------------------------------------------------------------------------ 160384 |
| ....TGAGGTAGTAGGTTGTATAGTTa.............................................. 14818   | 14818; |
| ....TGAGGTAGTAGGTTGTATAGTa............................................... 1403   | 1403; |
| ....TGAGGTAGTAGGTTGTgTAGTT............................................... 931   | 931; |
| ....TGAGGgAGTAGGTTGTATAGTT............................................... 859   | 859; |
| ....TGAGGTAGTAGGTTGTATAGTaa.............................................. 842   | 842; |
| ....TGAGGTAGTAGtTTGTATAGTT............................................... 800   | 800; |
| ....TGAGGTAGTAGGTTGTAcAGTT............................................... 556   | 556; |
| ....aGAGGTAGTAGGTTGTATAGTT............................................... 425   | 425; |
| ....TGAGGTAGTAGGgTGTATAGTT............................................... 372   | 372; |
| ....TGAGGTAGTAGGTTGTATAGTTg.............................................. 359   | 359; |
| ....TGAGGTAGTAGGTTGgATAGTT............................................... 289   | 289; |
| ....TGAGGTAGTAGGTTGTATAGTaT.............................................. 198   | 198; |
| ....TGAGGTAGTAGGTTGTATtGTT............................................... 161   | 161; |
| ....TGAGGTAGTAGGTTGTATAGa................................................ 148   | 148; |
| ....TGAGGTAGTAGGTTGTATAGgT............................................... 139   | 139; |
| ....TGAGGTAGTAGGTTGTtTAGTT............................................... 132   | 132; |
| ....TGAGGTAGgAGGTTGTATAGTTT.............................................. 130   | 130; |
| ....TGAGGTAGTAGGcTGTATAGTT............................................... 129   | 129; |
| ....TGAGGTAGTAGGTTGTATAtTT............................................... 128   | 128; |
| ....TGAGGgAGTAGGTTGTATAGT................................................ 119   | 119; |
| ....TGAGGTAGTAGGTTGTgTAGTTT.............................................. 118   | 118; |
| ....TGAGGTAGTAGGTaNTATAGTT............................................... 116   | 116; |
| ....TGAGGgAGTAGGTTGTATAGTTa.............................................. 105   | 105; |
| ....TGAGGTAGTAGGTTGTgTAGT................................................ 103   | 103; |
| ....TGAGGTAGTAGGTTGTgTAGTTa.............................................. 100   | 100; |
| ....TGAGGcAGTAGGTTGTATAGTT............................................... 99   | 99; |
| ....TGAGGgAGTAGGTTGTATAGTTT.............................................. 92   | 92; |
| ....TGAGGTAGTAGGTcNTATAGTT............................................... 85   | 85; |
| ....TGAGGTAGTAGGgTGTATAGT................................................ 77   | 77; |
| ...cTGAGGTAGTAGGTTGTATAGTT............................................... 71   | 71; |
| ....TGAGGTAGTAGGNTGTATAGTTa.............................................. 69   | 69; |
| ....TGgGGTAGTAGGTTGTATAGTT............................................... 59   | 59; |
| ....TGAGGTAGTAGGTTGTATAGaa............................................... 58   | 58; |
| ....TGAGGTAGTAGGaTGTATAGTT............................................... 57   | 57; |
| ....TGAGGTAGTAGGgTGTATAGTTa.............................................. 56   | 56; |
| ....aGAGGTAGTAGGTTGTATAGTTT.............................................. 56   | 56; |
| ....TGAGGTAGTAGGTTGTAgAGTT............................................... 55   | 55; |
| ....TGAGGTAGTAGGTTGTcTAGTT............................................... 51   | 51; |
| ....TGAGGTAGTAGGTTGTATAGTg............................................... 48   | 48; |
| ....TGAGGTAGTAGGTTGgATAGT................................................ 47   | 47; |
| ....TGAGGTAGTAGGgTGTATAGTTT.............................................. 43   | 43; |
| ....TGcGGTAGTAGGTTGTATAGTT............................................... 42   | 42; |
| ....TGAGGTAGTAGGTTGTAcAGTTa.............................................. 42   | 42; |
| ....TGAGGTAGTAGtTTGTATAGTTa.............................................. 41   | 41; |
| ....TGAGGTAGTAGGTTGTAcAGT................................................ 41   | 41; |
| ....TGAGGTAGTAGGTTGTATAGcT............................................... 40   | 40; |
| ....TGAGGTAGTAGGTTGTA--GTT............................................... 39   | 39; |
| ....TGAGGTAGTAGGTTGNAaAGTT............................................... 39   | 39; |
| ....TGAGGTAGTAGGTTGTATAGTTc.............................................. 39   | 39; |
| .....GAGGTAGTAGGTTGTATAGTTa.............................................. 39   | 39; |
| ....aGAGGTAGTAGGTTGTATAGT................................................ 38   | 38; |
| ....TGAGGgAGTAGGgTGTATAGTT............................................... 37   | 37; |
| ....cGAGGTAGTAGGTTGTATAGTT............................................... 37   | 37; |
| ....TGAGGTAGTAGGTTGTATcGTT............................................... 37   | 37; |
| ....TGAGGTAGTAGGTTGgATAGTTa.............................................. 37   | 37; |
| ....TGAGGTgGTAGGTTGTATAGTT............................................... 35   | 35; |
| ....TGAGGTAGTAGGTTtTATAGTT............................................... 35   | 35; |
| ....TGAGGTAGTAGtTTGTATAGT................................................ 34   | 34; |
| ....TGAGGTAGTAGcTTGTATAGTT............................................... 32   | 32; |
| ...TT-AGGTAGTAGGTTGTATAGTT............................................... 30   | 30; |
| ....aGAGGTAGTAGGTTGTATAGTTa.............................................. 30   | 30; |
| ....TGAGGTAGTAGGTgGTATAGTT............................................... 29   | 29; |
| ....TGAGGTAGTAGGTTGTATAGg................................................ 28   | 28; |
| ....TGAGGTAGTAGGTTGgATAGTTT.............................................. 28   | 28; |
| ....TGAGGTAGTAGGTTGNAcAGTT............................................... 28   | 28; |
| ....TGAGGTAGTAGtTTGTATAGTTT.............................................. 28   | 28; |
| ....TGAGGTAGTgGGTTGTATAGTT............................................... 27   | 27; |
| ....TGAGGTAGTAGGTgNTATAGTT............................................... 26   | 26; |
| ....TGAGGTAGTAGGTTGTATAGTc............................................... 26   | 26; |
| ....TGAGGTAGTAGGTTGTAcAGTTT.............................................. 26   | 26; |
| ....TGAGGaAGTAGGTTGTATAGTT............................................... 24   | 24; |
| ....TGAGGTAGTAGGcTGTATAGT................................................ 24   | 24; |
| ....TGAGGTAGcAGGTTGTATAGTT............................................... 24   | 24; |
| ....TGAGGTAGTAGGTTGTATAGaT............................................... 24   | 24; |
| ....TGAGGTAGTAGGTTGTATAtT................................................ 24   | 24; |
| ....TGAGGTAGTAGGTcGTATAGTT............................................... 24   | 24; |
| ....TGAGGTAGTAG-TTGTATAGTT............................................... 23   | 23; |
| ....TGAGGTAGTAGGTTGTATtGT................................................ 23   | 23; |
| ....TGAGGTAGTAGGTTGaATAGTT............................................... 22   | 22; |
| ....TGAGGTAGTAGGTTGNATAGTTa.............................................. 21   | 21; |
| ....TGAGGTcGTAGGTTGTATAGTT............................................... 20   | 20; |
| ....TGAGGTAGTAGGTT--ATAGTT............................................... 20   | 20; |
| .....GAGGTAGTAGGTTGTATAGTTaA............................................. 20   | 20; |
| ....TGAGGTAGTAGGTTGTATAGTga.............................................. 19   | 19; |
| ....TGAGGTAGTAGGTTGTtTAGTTa.............................................. 19   | 19; |
| ....TGAGGTAGTAGGTTGTATtGTTT.............................................. 19   | 19; |
| ....TGAGGTAGTAGGTTGTATtGTTa.............................................. 19   | 19; |
| ....TGAGGTAGTAGGTTGTtTAGT................................................ 18   | 18; |
| ....TGAGGgAGTAGGTTGgATAGTT............................................... 18   | 18; |
| ....TGAGGTAGTAGGcTGTATAGTTa.............................................. 18   | 18; |
| ....TGAGGTAGTAGGT-GTATAGTT............................................... 18   | 18; |
| ....TGAGGTAGTcGGTTGTATAGTT............................................... 17   | 17; |
| ....TGAGGTAGTAGGTTGNAgAGTT............................................... 17   | 17; |
| ....TGAGGgAGTAGGTTGTgTAGTT............................................... 17   | 17; |
| ....TGAGGTAGTA--TTGTATAGTT............................................... 17   | 17; |
| ....TGAGGTAGTAGGTTcTATAGTT............................................... 16   | 16; |
| ....TGAGGTAGTAGGTTGTATAGgTT.............................................. 16   | 16; |
| ....TGAGGTAGTAGGTTGNcTAGTT............................................... 16   | 16; |
| ....TGAGGTAGTAGGTNNTATAGTTa.............................................. 15   | 15; |
| ....TGAGGTAGTAGGTTGTgcAGTT............................................... 15   | 15; |
| ....TGAGGTAGTAGGgTGTATAGgT............................................... 15   | 15; |
| ....TGAGGTAGTAGGTTGTATAGgTa.............................................. 14   | 14; |
| ....TGAGGTAGTAGGTTGTATAcTT............................................... 14   | 14; |
| ....TGAGGcAGTAGGTTGTATAGT................................................ 14   | 14; |
| ....TGAGGTAGTAGGgTGgATAGTT............................................... 14   | 14; |
| ....TGAGGTAGTAGGTaNTATAGT................................................ 13   | 13; |
| ....TGgGGTAGTAGGTTGTATAGT................................................ 13   | 13; |
| ....TGAGGTAGTAGGTTaTATAGTT............................................... 13   | 13; |
| ....TGAGGTAGTAGGTcNTATAGT................................................ 13   | 13; |
| ....TGAGGgAGTAGGTTGTATAGTa............................................... 13   | 13; |
| ....TGAGGTAGTAGGTTGTATAGTgT.............................................. 13   | 13; |
| ....TGAGGTAtTAGGTTGTATAGTT............................................... 13   | 13; |
| ....TGAGGTAGTAGGTTGTtTAGTTT.............................................. 12   | 12; |
| ....TGAGGTAGTAGGTTGTATAGTag.............................................. 12   | 12; |
| ....TGAGGTAGTAGGcTGTATAGTTT.............................................. 12   | 12; |
| ....TGAGGcAGTAGGTTGTATAGTTT.............................................. 12   | 12; |
| ....TGAGGTAGTAGGTTGTATAaa................................................ 12   | 12; |
| ...cTGAGGTAGTAGGTTGTATAGT................................................ 11   | 11; |
| ....TGAGGT--TAGGTTGTATAGTT............................................... 11   | 11; |
| ....TGAGGTAGTAGGTTGTcTAGT................................................ 11   | 11; |
| ....TGAGGTAGTAGGTTGTAT-GTTT.............................................. 11   | 11; |
| ....TGAGGTAGTAGGaTGTATAGT................................................ 11   | 11; |
| ....TGAGGcAGTAGGTTGTATAGTTa.............................................. 10   | 10; |
| ....TGAGGTAGTAGGTaNTATAGTTT.............................................. 10   | 10; |
| ....TGAGGTAGTAGGTTGTA--GTTT.............................................. 10   | 10; |
| ....TGAGGTAGTAGGTaNTATAGTTa.............................................. 10   | 10; |
| ....TGgGGTAGTAGGTTGTATAGTTT.............................................. 10   | 10; |
| ....TGAGGTAGTAGGTTGTATAtTTT.............................................. 10   | 10; |
| ....TGAGGgAGTAGGTTGaATAGTT............................................... 9   | 9; |
| ....TGAGGgAGTAGGTTGTAcAGTT............................................... 9   | 9; |
| ....TGAtGTAGTAGGTTGTATAGTT............................................... 9   | 9; |
| ....TGAGGTAGTAGGTTGTcTAGTTa.............................................. 9   | 9; |
| ....TGAGGTAcTAGGTTGTATAGTT............................................... 9   | 9; |
| ....TGAG-TAGTAGGTTGTATAGTT............................................... 9   | 9; |
| ....gGAGGTAGTAGGTTGTATAGTT............................................... 9   | 9; |
| ....TGAGGTAGTAGGaTGTATAGTTa.............................................. 9   | 9; |
| ..tTTGAGGTAGTAGGTTGTATAG................................................. 8   | 8; |
| .....GAGGTAGTAGaTTGTATAGTTaA............................................. 8   | 8; |
| ....TGAGGTAGTAG-TTGTATAGTTa.............................................. 8   | 8; |
| ....TGAGGTAGTAGGTcNTATAGTTa.............................................. 8   | 8; |
| ....TGAGGTAGTAGGTTGNgTAGTT............................................... 8   | 8; |
| ....TGAGGTAGTAGGTTGTAgAGTTT.............................................. 8   | 8; |
| ....TGAGGTAGTAGGTTGNAaAGTTa.............................................. 8   | 8; |
| ....TGAGGTAGTAGGTTGTAgAGT................................................ 8   | 8; |
| ....aGAGGTAGTAGGTTGTATAGTa............................................... 8   | 8; |
| ....TGAGGTAGTAGGTTGggTAGTT............................................... 8   | 8; |
| ....TGAGGTAGTAGGNTGTATAGTa............................................... 8   | 8; |
| ....TGAGGTAGTAGGTTGTgTAGTa............................................... 7   | 7; |
| ....TGAGGTAGTAGGTTGTATAaT................................................ 7   | 7; |
| ....TGgGGTAGTAGGTTGTATAGTTa.............................................. 7   | 7; |
| ....TGAGGTAGTAGGTTGTATAaTT............................................... 7   | 7; |
| ....TGAGGTAGTAtGTTGTATAGTT............................................... 7   | 7; |
| ....TGAGGgAGTAGGgTGTATAGT................................................ 7   | 7; |
| ....TGcGGTAGTAGGTTGTATAGT................................................ 6   | 6; |
| ....TGAGGTAGTAGGTTGgATAGgT............................................... 6   | 6; |
| ....TGAGGgAGTAGGTTGTATAGcT............................................... 6   | 6; |
| ....TGAGGTAGTAGGTTGTATAGcTT.............................................. 6   | 6; |
| ....TGAGGTAGTAGGTTGTATAGc................................................ 6   | 6; |
| ....TGAGGTcGTAGGTTGTATAGT................................................ 6   | 6; |
| ....TGAGGTAGTAGGTTGTtTtGTT............................................... 6   | 6; |
| ....TaAGGTAGTAGGTTGTATAGTT............................................... 6   | 6; |
| ....TGAGGgAGTAGtTTGTATAGTT............................................... 6   | 6; |
| ....TGAGGTAGaAGGTTGTATAGTT............................................... 6   | 6; |
| ....TGAGGTAGTAGGTTGTATANTTa.............................................. 6   | 6; |
| ....TGAGGTgGTAGGTTGTATAGT................................................ 6   | 6; |
| ....TGAGGTAGTAGGTTGTgTAGTTg.............................................. 6   | 6; |
| ....TGAGGTgGTAGGTTGTATAGTTT.............................................. 6   | 6; |
| ...TT-AGGTAGTAGGTTGTATAGT................................................ 6   | 6; |
| ....TGAGGgAGTAGGgTGTATAGTTT.............................................. 6   | 6; |
| ....TGAGGcAGTAGGTTGgATAGTT............................................... 6   | 6; |
| ....TGAGGTAGTAGGTT-TATAGTT............................................... 6   | 6; |
| ....TGAGGTAGTAGGTgGTATAGT................................................ 6   | 6; |
| ....TGAGGTAGTgGGTTGTATAGT................................................ 6   | 6; |
| ....TGAGGTAGTAGGTTNTATAGTTa.............................................. 6   | 6; |
| ....TGAGGTAGgAGGgTGTATAGTTT.............................................. 5   | 5; |
| ....TGAGGTAGTAGGTTtTATAGT................................................ 5   | 5; |
| ....TGAGGTAaTAGGTTGTATAGTT............................................... 5   | 5; |
| ....TGAGGTAGTAGGTTGaATAGT................................................ 5   | 5; |
| ....TGAGGTAGTAGtTTGTATtGTT............................................... 5   | 5; |
| ....TGAGGTAGTAGGTTtTATAGTTa.............................................. 5   | 5; |
| ....TGAGGTAGTAGGcTGgATAGTT............................................... 5   | 5; |
| ....TGAaGTAGTAGGTTGTATAGTT............................................... 5   | 5; |
| ....TGAGGTAGTAGGNTGTgTAGTT............................................... 5   | 5; |
| ....TGAGGTAGTAGtTTGTgTAGTT............................................... 5   | 5; |
| ..GTTGAGGTAGTAGaTTGTATA.................................................. 5   | 5; |
| ....aGAGGTAGTAGGTTGTgTAGTT............................................... 5   | 5; |
| ....TGAGGTAGTAGGNTGTAcAGTT............................................... 5   | 5; |
| ....TGAGGTAGTAGGTTGNAaAGTTT.............................................. 5   | 5; |
| ....TGAGGgAGTAGcTTGTATAGTT............................................... 5   | 5; |
| ....cGAGGTAGTAGGTTGTATAGT................................................ 5   | 5; |
| ....TGAGGcAGTAGGgTGTATAGTT............................................... 5   | 5; |
| ....TGAGGTAGTAaGTTGTATAGTT............................................... 5   | 5; |
| ....TGAGGTAGTAGGT-GTATAGTTa.............................................. 5   | 5; |
| ....TGAGGTAGTAGGaTGTATAGTTT.............................................. 5   | 5; |
| ....TGAGGTAGTAGGTTGTATcGTTa.............................................. 5   | 5; |
| ....TGAGGTAGTAGGTaGTATAGTT............................................... 5   | 5; |
| ....TGAGGTAGTAGGTcGTATAGT................................................ 5   | 5; |
| ....TGAGGTAGTAGGTTGTcTAGTTT.............................................. 5   | 5; |
| ....TGAGGTAGTAGGTTGNAaAGT................................................ 5   | 5; |
| ....TGAGGTAGTAGGTTGTATAGTca.............................................. 5   | 5; |
| ....TGcGGTAGTAGGTTGTATAGTTa.............................................. 5   | 5; |
| ....TGAGGTAGTAGGTTGNcaAGTT............................................... 5   | 5; |
| ....TGAGGTAGTAGGTaNTgTAGTT............................................... 5   | 5; |
| ....TGAGGTAGcAGGTTGTATAGTTa.............................................. 4   | 4; |
| ....TGAGGgAGTAGGTTGTAgAGTT............................................... 4   | 4; |
| ....aGAGGgAGTAGGTTGTATAGTT............................................... 4   | 4; |
| ....TGAcGTAGTAGGTTGTATAGTT............................................... 4   | 4; |
| ....TGAGGcAGTAGGTTGTgTAGTT............................................... 4   | 4; |
| ....TGAGGTAGTAGGTTGTAcAGTa............................................... 4   | 4; |
| ....TGAGGgAGTAGGaTGTATAGTTT.............................................. 4   | 4; |
| ....TGAGGTAGcAGGTTGTATAGT................................................ 4   | 4; |
| ....TGcGGTAGTAGGTTGTATAGTTT.............................................. 4   | 4; |
| ....cGAGGTAGTAGGTTGTATAGTTa.............................................. 4   | 4; |
| ....TGAGGTAGTAGGTcNTATAGTTT.............................................. 4   | 4; |
| ....TGAGGTAGTAGGTgNTATAGT................................................ 4   | 4; |
| ....TGAGGTAGTAGGTgGTATAGTTa.............................................. 4   | 4; |
| ....TGAGGaAGTAGGTTGTATAGT................................................ 4   | 4; |
| ....TGAGGTAGTAGGTTGTATcGT................................................ 4   | 4; |
| ....TGAGGTAGTAGGTTGTAgAGgT............................................... 4   | 4; |
| ....TGAGGTAGTAGGgTGTgTAGTT............................................... 4   | 4; |
| ....TGAGGTAGTAGGTTcTATAGT................................................ 4   | 4; |
| ....TGAGGTAGTAGGTTtTATAGTTT.............................................. 4   | 4; |
| ....TGAGGTAGTAGGTTGNtTAGTT............................................... 4   | 4; |
| ....TGAGGTAGTAGGgTGTATAGTa............................................... 4   | 4; |
| ....TGAGGTAGTAGGTTGTAgAGTTa.............................................. 4   | 4; |
| ....TGAGGTgGTAGGTTGTATAGTTa.............................................. 4   | 4; |
| ....TGAGtTAGTAGGTTGTATAGTT............................................... 4   | 4; |
| ....TGAGGTAGTAGGTTGTgTAtTT............................................... 4   | 4; |
| ....TGAGGcAGTAGGTTGTATAGgT............................................... 4   | 4; |
| ....TGAGGgAGTAGGTTGTtTAGTT............................................... 3   | 3; |
| ....TGAGGTAGTAGGTTGNAcAGT................................................ 3   | 3; |
| ....TGAGGTAGTcGGTTGTATAGTTT.............................................. 3   | 3; |
| ....TGAGGTAGTAGGggGTATAGTT............................................... 3   | 3; |
| ....TGtGGTAGTAGGTTGTATAGTT............................................... 3   | 3; |
| ....cGAGGTAGTAGGTTGTATAGTTT.............................................. 3   | 3; |
| ....TGAGGTAGcAGGTTGTATAGTTT.............................................. 3   | 3; |
| ....TGAGGTAGTAGGNTGTATAGgT............................................... 3   | 3; |
| ....TGAGGTAGTAGGTTGTtTAtTT............................................... 3   | 3; |
| ....TGAGGTAGTAGGTTGTATAGga............................................... 3   | 3; |
| ....TGAGGTAGTAGGTTGNcTAGT................................................ 3   | 3; |
| ....TGAGGgAGTgGGTTGTATAGTT............................................... 3   | 3; |
| ....TGAGGTAGTAGGTTaTATAGT................................................ 3   | 3; |
| ....TGAGGTAGTAGGTTGTATAGaTT.............................................. 3   | 3; |
| ....TGAGGTAGTAGcTTGTATAGTTT.............................................. 3   | 3; |
| ....aGAGGTAGTAGGNTGTATAGTT............................................... 3   | 3; |
| ....TGAGGTAGTAGGNTGTATAGTaa.............................................. 3   | 3; |
| ....TGAGGTAGTAGGgTGTATAGg................................................ 3   | 3; |
| ....gGAGGTAGTAGGTTGTATAGT................................................ 3   | 3; |
| ....TGAGGTAGTAGtNTGTATAGTT............................................... 3   | 3; |
| ....TGAGGgAGTAGGTTGTATtGTT............................................... 3   | 3; |
| ....TGAGGgAGTAGGTTGgATAGT................................................ 3   | 3; |
| ....TGAGGTAGTgGGTTGTATAGTTT.............................................. 3   | 3; |
| ....TGgGGTAGTAGGTTGTAgAGTT............................................... 3   | 3; |
| ....TGAGGTAGTAGGTTGTgcAGTTT.............................................. 3   | 3; |
| ....TGAGGTAGTAGGTTGTATAGTcT.............................................. 3   | 3; |
| ....TGAGGTAGTgGGTTGTATAGTTa.............................................. 3   | 3; |
| ....TGAGGTAGTAGGTTGNAgAGTTa.............................................. 3   | 3; |
| ....TGAGGTAGTAGGTgGTATAGTTT.............................................. 3   | 3; |
| ....TGAGGTAGTtGGTTGTATAGTT............................................... 3   | 3; |
| ....TGAGGTAGTAGGTTGTtTAGgT............................................... 3   | 3; |
| ....TGAGGTAGTAGtTTGNATAGTT............................................... 3   | 3; |
| ....TGgGGTAGTAGGTTGTgTAGTT............................................... 3   | 3; |
| ....TGAGGTAGTAGGNaGTATAGTT............................................... 3   | 3; |
| ....TGAGGTAGTAGGTTGTAaAGTT............................................... 3   | 3; |
| ....TGAGGTAGTAGGgTGTtTAGTT............................................... 3   | 3; |
| ....TGAGGTtGTAGGTTGTATAGTT............................................... 3   | 3; |
| ....TGAGGTAGTAGcTTGTATAGT................................................ 3   | 3; |
| ....TGAGGTAGTAGtTTGTATAGTa............................................... 3   | 3; |
| ....TGAGGgAGTAGGTTGTATAGgT............................................... 3   | 3; |
| ....TGAGGTAGTAGGTgNTATAGTTa.............................................. 3   | 3; |
| ....TGAGGTAGTAGGTTGNAcAGTTa.............................................. 3   | 3; |
| ....TGAGGTAGTAGGTcGTATAGTTa.............................................. 3   | 3; |
| ....TGAGGTAGTAcGTTGTATAGTT............................................... 3   | 3; |
| .......GGTAGTAGGTTGTATAGTTgA............................................. 3   | 3; |
| ....TGAGGTAGTAGGTTGNATAGTa............................................... 2   | 2; |
| ....TGAGGTAGTAGGTTGggTAGTTT.............................................. 2   | 2; |
| ....TGAGGTAGTAGGTTGTATcGTTT.............................................. 2   | 2; |
| ....TGgGGTAGTAGGTTGTtTAGTT............................................... 2   | 2; |
| ....aGAGGTAGTAGGTTGTATAtTT............................................... 2   | 2; |
| ....TGAGGTAGTAGGTTGNAcAGTTT.............................................. 2   | 2; |
| ....TGAGGTAGTAGGaTGaATAGTT............................................... 2   | 2; |
| ....aGAGGTAGTAGGgTGTATAGTT............................................... 2   | 2; |
| ....TGAGGTAGTAGGNTGTATAGaT............................................... 2   | 2; |
| ....TGAGGgAGTAGGTTGTATAGTTg.............................................. 2   | 2; |
| ....TGAGGTAGTAGGTTGgAcAGTT............................................... 2   | 2; |
| ....TGAGGTAGTAGGTTGNAgAGTTT.............................................. 2   | 2; |
| ....TGAGcTAGTAGGTTGTATAGTT............................................... 2   | 2; |
| ....TGAGGgAGTAGGTTGTATAGTaT.............................................. 2   | 2; |
| ....TGAGGTAGTAGGTTGaATAGTTa.............................................. 2   | 2; |
| ....TGAGGTAGTAGGcTGTtTAGTT............................................... 2   | 2; |
| ....TGAGGggGTAGGTTGTATAGTT............................................... 2   | 2; |
| ....aGAGGTAGTAGGTTGTATAGTaT.............................................. 2   | 2; |
| ....TGAGGaAGTAGGTTGTATAGTTT.............................................. 2   | 2; |
| ....TGAGGTAGTAGGTTGTgTAGTaT.............................................. 2   | 2; |
| ....TGAGGTAGTAGGgTGTATAGgTT.............................................. 2   | 2; |
| ....TGAGGTAGTAGGTNNTgTAGTT............................................... 2   | 2; |
| ....TGAGGgAGTAGGTTGTATAGTg............................................... 2   | 2; |
| ....TGAGGTAGTAGGTTGaATAGTTT.............................................. 2   | 2; |
| ....TGAGGTAGTAGGTTGgATAGTg............................................... 2   | 2; |
| ....TGAGaTAGTAGGTTGTATAGTT............................................... 2   | 2; |
| ....TGAGGgAGTAGGcTGTATAGTT............................................... 2   | 2; |
| ....TGAGGTcGTAGGTTGTATAGTTT.............................................. 2   | 2; |
| ....TGAGGTAGTAGGTTGNcTAGTTa.............................................. 2   | 2; |
| ....TGAGGTAGTAtGTTGTATAGTTa.............................................. 2   | 2; |
| ....TGAGGTAGTAGGTTGTATAtGT............................................... 2   | 2; |
| ....TGAGGTAGTAGGTaNTATAGTa............................................... 2   | 2; |
| ....aGAGGTAGTAGGTTGTcTAGTT............................................... 2   | 2; |
| ....TGAGGTAGTAGGTTGTcTAtTT............................................... 2   | 2; |
| ....TGAGGgAGTAGGTTGTATAtTT............................................... 2   | 2; |
| ....TGAGGgAGTAGGTTGgATAGTTT.............................................. 2   | 2; |
| ......AGGTAGTAGGTTGTATAGTTa.............................................. 2   | 2; |
| ....TGAGGaAGTAGGTTGTATAGTTa.............................................. 2   | 2; |
| ....TcAGGTAGTAGGTTGTATAGTT............................................... 2   | 2; |
| ....TGAGGTAGTAGGTaNTcTAGTT............................................... 2   | 2; |
| ....TGAGGTAGTAGGTTGNAaAGTa............................................... 2   | 2; |
| ....TGAGGTAGTAGGNTGTATAGTTg.............................................. 2   | 2; |
| ....TGAGGTcGTAGGTTGTATAGTTa.............................................. 2   | 2; |
| ....TGAGGTAGTAGGgTGgATAGTTT.............................................. 2   | 2; |
| ....TGAGGTAGTAGGTTGTATAGcTa.............................................. 2   | 2; |
| ....TGAGGcAGTAGGcTGTATAGTT............................................... 2   | 2; |
| ....TGAtGTAGTAGGTTGTATAGT................................................ 2   | 2; |
| ....TGAGGTAGTAGGTTGgATAtTT............................................... 2   | 2; |
| ....TGAGGTAGTAGGgTGgATAGT................................................ 2   | 2; |
| ....TGAGGTAGTAGcTTGTATAGTTa.............................................. 2   | 2; |
| ....TGAGGTAGTcGGTTGTATAGTTa.............................................. 2   | 2; |
| ....TGAGGTAGTAGGNTcTATAGTT............................................... 2   | 2; |
| ....TGAGGTAGTAGGcTGTgTAGTT............................................... 2   | 2; |
| ....TGAGGTAGTcGGTTGTATAGT................................................ 2   | 2; |
| ....TGAGGgAGTAGGNTGTATAGTT............................................... 2   | 2; |
| ....TGAGGTAGTAGGTTGgATtGTT............................................... 2   | 2; |
| ....TGAGGcAGTAGGTTGTAgAGTT............................................... 2   | 2; |
| ....TGAGGTAGTAGGgTGTATAGTTg.............................................. 2   | 2; |
| ....TGAGGTAGTAGGcTGTATAGgT............................................... 2   | 2; |
| ....TGAGGTAGTAGGTTcTATAGTTa.............................................. 2   | 2; |
| ....TGAGGTAGTAGGTTGTATAtTa............................................... 2   | 2; |
| ....TGAGGTAGTAGGTTGTActGTT............................................... 2   | 2; |
| ....TGAGGTAGTAGGNTGgATAGTT............................................... 2   | 2; |
| ....TGAGGTAGTAGGTTGNgTAGT................................................ 2   | 2; |
| ....TGAGGTAtTAGGTTGTATAGTTa.............................................. 2   | 2; |
| ....TGAGGgAGaAGGTTGTATAGTT............................................... 2   | 2; |
| .....GAGGTAGTAGGTTGTATAGTa............................................... 2   | 2; |
| ....TGAGGTAGTAGGcTGTATAGTa............................................... 2   | 2; |
| ....TGAGGTAGTAGGTNNTATAGTa............................................... 2   | 2; |
| ....TGAGGgAGTAGGTTGTATcGTT............................................... 1   | 1; |
| ....TGgGGTAGTAGGTTGgATAGTT............................................... 1   | 1; |
| ....TGAGGTAGTgGGTTGTgTAGTT............................................... 1   | 1; |
| ....TGAGGTAGTAGGTTGTgcAGT................................................ 1   | 1; |
| ....TGAGGTAGTAGGTTGTATctTT............................................... 1   | 1; |
| ....TGAGGTAGTAGGTTGTAcAGTTg.............................................. 1   | 1; |
| ....TGAGGTAGTAGGgTGTATtGTT............................................... 1   | 1; |
| ....TGAGGTAGTAGGTTGTAcAGgT............................................... 1   | 1; |
| ....TGAGGgAGTAGGTTGTATtGT................................................ 1   | 1; |
| ....TGAGGTAGTAGGTTGggTAGT................................................ 1   | 1; |
| ....aGAGGTAGTAGGTTGTATtGTT............................................... 1   | 1; |
| ....TGAGGTAGTAGGTcNTgTAGTT............................................... 1   | 1; |
| ....TGAGGTAGTAGtTTGgATAGTT............................................... 1   | 1; |
| ....TGAGGTAGTAGGaTGTATAGcT............................................... 1   | 1; |
| ....TGAGGTAGTAGtTTGTATAGgT............................................... 1   | 1; |
| ....TGAGGaAGTAGGTTGTAgAGTT............................................... 1   | 1; |
| ....TGAGGTAGTAGGTTGNAgAGT................................................ 1   | 1; |
| ....TGAGGTAGTAGGTTGTtgAGTT............................................... 1   | 1; |
| ....TGAGGTAGTAGGTTGTtTtGTTT.............................................. 1   | 1; |
| ....TGAGGTAGTAGtTTNTATAGTT............................................... 1   | 1; |
| ....TGAGGTAGTAGGTTNTgTAGTT............................................... 1   | 1; |
| ....TGAGGgAGTAGGTTGTgTAGT................................................ 1   | 1; |
| ....TGAGGaAGTAGGNTGTATAGTT............................................... 1   | 1; |
| .....GAGGTAGTAGGTTGTATAGTaT.............................................. 1   | 1; |
| ...cTGAGGTAGTAGNTTGTATAGTT............................................... 1   | 1; |
| ....TGAGGTAGTAGGcTGTATtGTT............................................... 1   | 1; |
| ....TGAGGgAGTAGGTTGTgTAGTTT.............................................. 1   | 1; |
| ....TGgGGTAGTAGGTTGTATtGTT............................................... 1   | 1; |
| ....TGAGGTAGTAGGTTGNgTAGTTT.............................................. 1   | 1; |
| ....TGAGGgAGTAGGTTGTATAGcTT.............................................. 1   | 1; |
| ....TGAGGTAGTAGGTTGTAgtGTT............................................... 1   | 1; |
| ....TGAGGTAGTAGGTTGTATtGTa............................................... 1   | 1; |
| ....TGAGGTAGTAGGTTGNgaAGTT............................................... 1   | 1; |
| ....TGAGGTAGTAGtTaNTATAGTT............................................... 1   | 1; |
| ....TGAGGTAGTAGtTTGTATAtTT............................................... 1   | 1; |
| ....TGAGGTAGTAGGTcGTATAGTTT.............................................. 1   | 1; |
| ....TGgGGgAGTAGGTTGTATAGTT............................................... 1   | 1; |
| ....TGAGGTAGTAGGNTGaATAGTT............................................... 1   | 1; |
| ....TGAGGTAGTAGGgTGTATcGTT............................................... 1   | 1; |
| ....TGcGGgAGTAGGTTGTATAGTT............................................... 1   | 1; |
| ....TGAGGgAGgAGGTTGTATAGTTT.............................................. 1   | 1; |
| ....TGAGGTAGTAGcTTGTgTAGTT............................................... 1   | 1; |
| ....gGAGGTAGTAGGTTGTATAGTTT.............................................. 1   | 1; |
| ....TGAGGTAGTAGGTTGTATAGag............................................... 1   | 1; |
| ....TGAGGTAGTAGGTTGgATAGTa............................................... 1   | 1; |
| ....TGcGGTAGTAGGgTGTATAGTT............................................... 1   | 1; |
| ....TGAGGTAGTAGGTTGTATAcTa............................................... 1   | 1; |
| ....TGAGGTAGTAGGTTGgtTAGTT............................................... 1   | 1; |
| ....TGAGGTAGTAGtTNNTATAGTT............................................... 1   | 1; |
| ....TGAGGTAGgAGGTTGTgTAGTTT.............................................. 1   | 1; |
| ....TGAGGTAGTAGGTTGNgcAGTT............................................... 1   | 1; |
| ....gGAGGTAGTAGGTTGTATAGTTa.............................................. 1   | 1; |
| ....TGAGGTAGTAGGTTGNccAGTT............................................... 1   | 1; |
| ....TGAGGTAGTAGGTTGNtcAGTT............................................... 1   | 1; |
| ....TGAGGTAGTAGtTTGTATcGTT............................................... 1   | 1; |
| ....TGAGGgAGTAGGTTtTATAGTT............................................... 1   | 1; |
| ....TGAGGTAGTAGGNTGTATAGTaT.............................................. 1   | 1; |
| ....aGAGGTAGTAGGTTGTtTAGTT............................................... 1   | 1; |
| ....TGAGGTAGTAGGaTGTgTAGTT............................................... 1   | 1; |
| ....TGAGGTAGTAGGTTGTgTAGaT............................................... 1   | 1; |
| ....TGAGGTAGTAGGTTGNgTAGTTa.............................................. 1   | 1; |
| ....aGAGGTAGTAGGTTGTATAGTTg.............................................. 1   | 1; |
| ------------------------------------------------------------------------ 27329 |
| ------------------------------------------------------------------------ 187713 |
| hsa-let-7a-5p(hsa-let-7a-3) GGGTGAGGTAGTAGGTTGTATAGTTTGGGGCTCTGCCCTGCTATGGGATAACTATACAATCTACTGTCTTTCCT (((.(((..((((((((((((((((((((((...)))))).........))))))))))))))))..))).))) (-34.40) \*\*\*TGAGGTAGTAGGTTGTATAGTT\*\*\*\*\*\*\*\*\*\*\*\*\*\*\*\*\*\*\*\*\*\*\*\*\*\*\*\*\*\*\*\*\*\*\*\*\*\*\*\*\*\*\*\*\*\*\*\*\* T  M |
| ...TGAGGTAGTAGGTTGTATAGTT..................................................\* 120200   | 120200; |
| ...TGAGGTAGTAGGTTGTATAGT................................................... 19848   | 19848; |
| ...TGAGGTAGTAGGTTGTATAGTTT................................................. 12180   | 12180; |
| ...TGAGGTAGTAGGTTGTATAG.................................................... 2746   | 2746; |
| ...TGAGGTAGTAGGNTGTATAGTT.................................................. 1804   | 1804; |
| ...TGAGGTAGTAGGTTGNATAGTT.................................................. 563   | 563; |
| ...TGAGGTAGTAGGTNNTATAGTT.................................................. 529   | 529; |
| ...TGAGGTAGTAGGTTGTATA..................................................... 322   | 322; |
| ...TGAGGTAGTAGGNTGTATAGT................................................... 278   | 278; |
| ...TGAGGTAGTAGGTTNTATAGTT.................................................. 212   | 212; |
| ...TGAGGTAGTAGGTTGTATANTT.................................................. 172   | 172; |
| ...TGAGGTAGTAGGNTGTATAGTTT................................................. 168   | 168; |
| ....GAGGTAGTAGGTTGTATAGTT.................................................. 158   | 158; |
| ...TGAGGTAGTAGGTTGTAT...................................................... 114   | 114; |
| ...TGAGGTAGTAGGTTGNATAGT................................................... 75   | 75; |
| ...TGAGGTAGTAGGTTGNATAGTTT................................................. 72   | 72; |
| ...TGAGGTAGTAGGTNNTATAGT................................................... 71   | 71; |
| ...TGAGGTAGTAGG............................................................ 63   | 63; |
| ...TGAGGTAGTAGGTNNTATAGTTT................................................. 44   | 44; |
| ...TGAGGTAGTAGGTTGTATANNT.................................................. 42   | 42; |
| ..GTGAGGTAGTAGGTTGTATAGT................................................... 37   | 37; |
| ...TGAGGTAGTAGGTTGTA....................................................... 35   | 35; |
| ...TGAGGTAGTAGGNTGTATAG.................................................... 34   | 34; |
| ....GAGGTAGTAGGTTGTATAGTTT................................................. 28   | 28; |
| ...TGAGGTAGTAGGTTGTATANT................................................... 28   | 28; |
| ....GAGGTAGTAGGTTGTATAGT................................................... 27   | 27; |
| ...TGAGGTAGTAGGTTNTATAGT................................................... 25   | 25; |
| ...TGAGGTAGTAGGTTNTATAGTTT................................................. 18   | 18; |
| ...TGAGGTAGTAGGTTGT........................................................ 17   | 17; |
| ..GTGAGGTAGTAGGTTGTATAGTT.................................................. 16   | 16; |
| ...TGAGGTAGTAGGTTGTATANTTT................................................. 15   | 15; |
| ...TGAGGTAGTAGGTTGTATAGTTTG................................................ 14   | 14; |
| .....AGGTAGTAGGTTGTATAGTT.................................................. 13   | 13; |
| ...TGAGGTAGTAGGTTGNATAG.................................................... 11   | 11; |
| ...TGAGGTAGTAGGTNNTATAG.................................................... 10   | 10; |
| ...TGAGGTAGTAGGTTGTATANNTT................................................. 9   | 9; |
| .....AGGTAGTAGGTTGTATAGTTTG................................................ 8   | 8; |
| ...................................................CTATACAATCTACTGTCTTTC... 8   | 8; |
| ..GTGAGGTAGTAGGTTGTATAG.................................................... 6   | 6; |
| ...TGAGGTAGTAGGTT.......................................................... 4   | 4; |
| ...TGAGGTAGTAGGTTG......................................................... 3   | 3; |
| ....GAGGTAGTAGGTTGTATAG.................................................... 3   | 3; |
| .....AGGTAGTAGGTTGTATAGT................................................... 2   | 2; |
| ......GGTAGTAGGTTGTATAGTT.................................................. 1   | 1; |
| -------------------------------------------------------------------------- 160033 |
| ...TGAGGTAGTAGGTTGTATAGTTa................................................. 14818   | 14818; |
| ...TGAGGTAGTAGGTTGTATAGTa.................................................. 1403   | 1403; |
| ...TGAGGTAGTAGGTTGTgTAGTT.................................................. 931   | 931; |
| ...TGAGGgAGTAGGTTGTATAGTT.................................................. 859   | 859; |
| ...TGAGGTAGTAGGTTGTATAGTaa................................................. 842   | 842; |
| ...TGAGGTAGTAGtTTGTATAGTT.................................................. 800   | 800; |
| ...TGAGGTAGTAGGTTGTAcAGTT.................................................. 556   | 556; |
| ...aGAGGTAGTAGGTTGTATAGTT.................................................. 425   | 425; |
| ...TGAGGTAGTAGGgTGTATAGTT.................................................. 372   | 372; |
| ...TGAGGTAGTAGGTTGTATAGTTg................................................. 359   | 359; |
| ...TGAGGTAGTAGGTTGgATAGTT.................................................. 289   | 289; |
| ...TGAGGTAGTAGGTTGTATAGTaT................................................. 198   | 198; |
| ...TGAGGTAGTAGGTTGTATtGTT.................................................. 161   | 161; |
| ...TGAGGTAGTAGGTTGTATAGa................................................... 148   | 148; |
| ...TGAGGTAGTAGGTTGTATAGgT.................................................. 139   | 139; |
| ...TGAGGTAGTAGGTTGTtTAGTT.................................................. 132   | 132; |
| ...TGAGGTAGgAGGTTGTATAGTTT................................................. 130   | 130; |
| ...TGAGGTAGTAGGcTGTATAGTT.................................................. 129   | 129; |
| ...TGAGGTAGTAGGTTGTATAtTT.................................................. 128   | 128; |
| ...TGAGGgAGTAGGTTGTATAGT................................................... 119   | 119; |
| ...TGAGGTAGTAGGTTGTgTAGTTT................................................. 118   | 118; |
| ...TGAGGTAGTAGGTaNTATAGTT.................................................. 116   | 116; |
| ...TGAGGgAGTAGGTTGTATAGTTa................................................. 105   | 105; |
| ...TGAGGTAGTAGGTTGTgTAGT................................................... 103   | 103; |
| ...TGAGGTAGTAGGTTGTgTAGTTa................................................. 100   | 100; |
| ...TGAGGcAGTAGGTTGTATAGTT.................................................. 99   | 99; |
| ...TGAGGgAGTAGGTTGTATAGTTT................................................. 92   | 92; |
| ...TGAGGTAGTAGGTcNTATAGTT.................................................. 85   | 85; |
| ...TGAGGTAGTAGGgTGTATAGT................................................... 77   | 77; |
| ..cTGAGGTAGTAGGTTGTATAGTT.................................................. 71   | 71; |
| ...TGAGGTAGTAGGNTGTATAGTTa................................................. 69   | 69; |
| ...TGgGGTAGTAGGTTGTATAGTT.................................................. 59   | 59; |
| ...TGAGGTAGTAGGTTGTATAGaa.................................................. 58   | 58; |
| ...TGAGGTAGTAGGaTGTATAGTT.................................................. 57   | 57; |
| ...aGAGGTAGTAGGTTGTATAGTTT................................................. 56   | 56; |
| ...TGAGGTAGTAGGgTGTATAGTTa................................................. 56   | 56; |
| ...TGAGGTAGTAGGTTGTAgAGTT.................................................. 55   | 55; |
| ...TGAGGTAGTAGGTTGTcTAGTT.................................................. 51   | 51; |
| ...TGAGGTAGTAGGTTGTATAGTg.................................................. 48   | 48; |
| ...TGAGGTAGTAGGTTGgATAGT................................................... 47   | 47; |
| ...TGAGGTAGTAGGgTGTATAGTTT................................................. 43   | 43; |
| ...TGcGGTAGTAGGTTGTATAGTT.................................................. 42   | 42; |
| ...TGAGGTAGTAGGTTGTAcAGTTa................................................. 42   | 42; |
| ...TGAGGTAGTAGtTTGTATAGTTa................................................. 41   | 41; |
| ...TGAGGTAGTAGGTTGTAcAGT................................................... 41   | 41; |
| ...TGAGGTAGTAGGTTGTATAGcT.................................................. 40   | 40; |
| ...TGAGGTAGTAGGTTGTATAGTTc................................................. 39   | 39; |
| ....GAGGTAGTAGGTTGTATAGTTa................................................. 39   | 39; |
| ...TGAGGTAGTAGGTTGNAaAGTT.................................................. 39   | 39; |
| ...TGAGGTAGTAGGTTGTA--GTT.................................................. 39   | 39; |
| ...aGAGGTAGTAGGTTGTATAGT................................................... 38   | 38; |
| ...TGAGGTAGTAGGTTGTATcGTT.................................................. 37   | 37; |
| ...cGAGGTAGTAGGTTGTATAGTT.................................................. 37   | 37; |
| ...TGAGGTAGTAGGTTGgATAGTTa................................................. 37   | 37; |
| ...TGAGGgAGTAGGgTGTATAGTT.................................................. 37   | 37; |
| ...TGAGGTgGTAGGTTGTATAGTT.................................................. 35   | 35; |
| ...TGAGGTAGTAGGTTtTATAGTT.................................................. 35   | 35; |
| ...TGAGGTAGTAGtTTGTATAGT................................................... 34   | 34; |
| ...TGAGGTAGTAGcTTGTATAGTT.................................................. 32   | 32; |
| ...aGAGGTAGTAGGTTGTATAGTTa................................................. 30   | 30; |
| ...TGAGGTAGTAGGTgGTATAGTT.................................................. 29   | 29; |
| ...TGAGGTAGTAGGTTGgATAGTTT................................................. 28   | 28; |
| ...TGAGGTAGTAGGTTGTATAGg................................................... 28   | 28; |
| ...TGAGGTAGTAGtTTGTATAGTTT................................................. 28   | 28; |
| ...TGAGGTAGTAGGTTGNAcAGTT.................................................. 28   | 28; |
| ...TGAGGTAGTgGGTTGTATAGTT.................................................. 27   | 27; |
| ...TGAGGTAGTAGGTgNTATAGTT.................................................. 26   | 26; |
| ...TGAGGTAGTAGGTTGTAcAGTTT................................................. 26   | 26; |
| ...TGAGGTAGTAGGTTGTATAGTc.................................................. 26   | 26; |
| ...TGAGGTAGTAGGTTGTATAtT................................................... 24   | 24; |
| ...TGAGGTAGTAGGTcGTATAGTT.................................................. 24   | 24; |
| ...TGAGGTAGTAGGcTGTATAGT................................................... 24   | 24; |
| ...TGAGGaAGTAGGTTGTATAGTT.................................................. 24   | 24; |
| ...TGAGGTAGTAGGTTGTATAGaT.................................................. 24   | 24; |
| ...TGAGGTAGcAGGTTGTATAGTT.................................................. 24   | 24; |
| ...TGAGGTAGTAGGTTGTATtGT................................................... 23   | 23; |
| ...TGAGGTAGTAG-TTGTATAGTT.................................................. 23   | 23; |
| ...TGAGGTAGTAGGTTGaATAGTT.................................................. 22   | 22; |
| ...TGAGGTAGTAGGTTGNATAGTTa................................................. 21   | 21; |
| ...TGAGGTcGTAGGTTGTATAGTT.................................................. 20   | 20; |
| ...TGAGGTAGTAGGTT--ATAGTT.................................................. 20   | 20; |
| ...TGAGGTAGTAGGTTGTATtGTTT................................................. 19   | 19; |
| ...TGAGGTAGTAGGTTGTATAGTga................................................. 19   | 19; |
| ...TGAGGTAGTAGGTTGTATtGTTa................................................. 19   | 19; |
| ...TGAGGTAGTAGGTTGTtTAGTTa................................................. 19   | 19; |
| ...TGAGGTAGTAGGcTGTATAGTTa................................................. 18   | 18; |
| ...TGAGGTAGTAGGTTGTtTAGT................................................... 18   | 18; |
| ...TGAGGTAGTAGGT-GTATAGTT.................................................. 18   | 18; |
| ...TGAGGgAGTAGGTTGgATAGTT.................................................. 18   | 18; |
| ...TGAGGTAGTAGGTTGNAgAGTT.................................................. 17   | 17; |
| ...TGAGGgAGTAGGTTGTgTAGTT.................................................. 17   | 17; |
| ...TGAGGTAGTA--TTGTATAGTT.................................................. 17   | 17; |
| ...TGAGGTAGTcGGTTGTATAGTT.................................................. 17   | 17; |
| ...TGAGGTAGTAGGTTcTATAGTT.................................................. 16   | 16; |
| ...TGAGGTAGTAGGTTGTATAGgTT................................................. 16   | 16; |
| ...TGAGGTAGTAGGTTGNcTAGTT.................................................. 16   | 16; |
| ...TtAGGTAGTAGGTTGTATAGTT.................................................. 15   | 15; |
| ...TGAGGTAGTAGGgTGTATAGgT.................................................. 15   | 15; |
| ...TGAGGTAGTAGGTTGTgcAGTT.................................................. 15   | 15; |
| ...TGAGGTAGTAGGTNNTATAGTTa................................................. 15   | 15; |
| ...TGAGGTAGTAGGTTGTATAcTT.................................................. 14   | 14; |
| ...TGAGGcAGTAGGTTGTATAGT................................................... 14   | 14; |
| ...TGAGGTAGTAGGgTGgATAGTT.................................................. 14   | 14; |
| ...TGAGGTAGTAGGTTGTATAGgTa................................................. 14   | 14; |
| ...TGAGGgAGTAGGTTGTATAGTa.................................................. 13   | 13; |
| ...TGAGGTAGTAGGTTaTATAGTT.................................................. 13   | 13; |
| ...TGAGGTAGTAGGTaNTATAGT................................................... 13   | 13; |
| ...TGAGGTAGTAGGTTGTATAGTgT................................................. 13   | 13; |
| ...TGAGGTAtTAGGTTGTATAGTT.................................................. 13   | 13; |
| ...TGgGGTAGTAGGTTGTATAGT................................................... 13   | 13; |
| ...TGAGGTAGTAGGTcNTATAGT................................................... 13   | 13; |
| ...TGAGGTAGTAGGcTGTATAGTTT................................................. 12   | 12; |
| ...TGAGGcAGTAGGTTGTATAGTTT................................................. 12   | 12; |
| ...TGAGGTAGTAGGTTGTtTAGTTT................................................. 12   | 12; |
| ...TGAGGTAGTAGGTTGTATAaa................................................... 12   | 12; |
| ...TGAGGTAGTAGGTTGTATAGTag................................................. 12   | 12; |
| ..cTGAGGTAGTAGGTTGTATAGT................................................... 11   | 11; |
| ...TGAGGTAGTAGGaTGTATAGT................................................... 11   | 11; |
| ...TGAGGTAGTAGGTTGTAT-GTTT................................................. 11   | 11; |
| ...TGAGGT--TAGGTTGTATAGTT.................................................. 11   | 11; |
| ...TGAGGTAGTAGGTTGTcTAGT................................................... 11   | 11; |
| ...TGgGGTAGTAGGTTGTATAGTTT................................................. 10   | 10; |
| ...TGAGGcAGTAGGTTGTATAGTTa................................................. 10   | 10; |
| ...TGAGGTAGTAGGTaNTATAGTTa................................................. 10   | 10; |
| ...TGAGGTAGTAGGTaNTATAGTTT................................................. 10   | 10; |
| ...TGAGGTAGTAGGTTGTATAtTTT................................................. 10   | 10; |
| ...TGAGGTAGTAGGTTGTA--GTTT................................................. 10   | 10; |
| ...gGAGGTAGTAGGTTGTATAGTT.................................................. 9   | 9; |
| ...TGAGGTAGTAGGaTGTATAGTTa................................................. 9   | 9; |
| ...TGAtGTAGTAGGTTGTATAGTT.................................................. 9   | 9; |
| ...TGAGGTAGTAGGTTGTcTAGTTa................................................. 9   | 9; |
| ...TGAGGgAGTAGGTTGaATAGTT.................................................. 9   | 9; |
| ...TGAG-TAGTAGGTTGTATAGTT.................................................. 9   | 9; |
| ...TGAGGgAGTAGGTTGTAcAGTT.................................................. 9   | 9; |
| ...TGAGGTAcTAGGTTGTATAGTT.................................................. 9   | 9; |
| ...TGAGGTAGTAGGTTGTAgAGTTT................................................. 8   | 8; |
| ...TGAGGTAGTAGGNTGTATAGTa.................................................. 8   | 8; |
| ...TGAGGTAGTAGGTTGTAgAGT................................................... 8   | 8; |
| ...TGAGGTAGTAGGTTGNgTAGTT.................................................. 8   | 8; |
| ...aGAGGTAGTAGGTTGTATAGTa.................................................. 8   | 8; |
| ...TGAGGTAGTAGGTcNTATAGTTa................................................. 8   | 8; |
| ...TGAGGTAGTAGGTTGNAaAGTTa................................................. 8   | 8; |
| ...TGAGGTAGTAG-TTGTATAGTTa................................................. 8   | 8; |
| ...TGAGGTAGTAGGTTGggTAGTT.................................................. 8   | 8; |
| ...TGAGGTAGTAGGTTGTgTAGTa.................................................. 7   | 7; |
| ...TGAGGgAGTAGGgTGTATAGT................................................... 7   | 7; |
| ...TGAGGTAGTAGGTTGTATAaT................................................... 7   | 7; |
| ...TGAGGTAGTAGGTTGTATAaTT.................................................. 7   | 7; |
| ...TGAGGTAGTAtGTTGTATAGTT.................................................. 7   | 7; |
| ...TGgGGTAGTAGGTTGTATAGTTa................................................. 7   | 7; |
| ...TGAGGgAGTAGtTTGTATAGTT.................................................. 6   | 6; |
| ...TGAGGTAGTAGGTTNTATAGTTa................................................. 6   | 6; |
| ...TGcGGTAGTAGGTTGTATAGT................................................... 6   | 6; |
| ...TGAGGTAGTgGGTTGTATAGT................................................... 6   | 6; |
| ...TGAGGTAGTAGGTTGgATAGgT.................................................. 6   | 6; |
| ...TGAGGTAGTAGGTT-TATAGTT.................................................. 6   | 6; |
| ...TGAGGTAGaAGGTTGTATAGTT.................................................. 6   | 6; |
| ...TGAGGTAGTAGGTgGTATAGT................................................... 6   | 6; |
| ...TaAGGTAGTAGGTTGTATAGTT.................................................. 6   | 6; |
| ...TGAGGcAGTAGGTTGgATAGTT.................................................. 6   | 6; |
| ...TGAGGgAGTAGGTTGTATAGcT.................................................. 6   | 6; |
| ...TGAGGTAGTAGGTTGTATAGcTT................................................. 6   | 6; |
| ...TGAGGTgGTAGGTTGTATAGTTT................................................. 6   | 6; |
| ...TGAGGTAGTAGGTTGTATANTTa................................................. 6   | 6; |
| ...TGAGGTAGTAGGTTGTgTAGTTg................................................. 6   | 6; |
| ...TGAGGTAGTAGGTTGTATAGc................................................... 6   | 6; |
| ...TGAGGTcGTAGGTTGTATAGT................................................... 6   | 6; |
| ...TGAGGTAGTAGGTTGTtTtGTT.................................................. 6   | 6; |
| ...TGAGGgAGTAGGgTGTATAGTTT................................................. 6   | 6; |
| ...TGAGGTgGTAGGTTGTATAGT................................................... 6   | 6; |
| ...TGAGGTAGTAGGTTGNAaAGTTT................................................. 5   | 5; |
| ...TGAGGcAGTAGGgTGTATAGTT.................................................. 5   | 5; |
| ...TGAGGTAGTAGGTTGTcTAGTTT................................................. 5   | 5; |
| ...TGAGGTAGTAGGTcGTATAGT................................................... 5   | 5; |
| ...TGAGGTAGTAGGTTtTATAGT................................................... 5   | 5; |
| ...TGAGGTAGTAGtTTGTgTAGTT.................................................. 5   | 5; |
| ...TGAGGTAGTAGGaTGTATAGTTT................................................. 5   | 5; |
| ...TGAGGTAGgAGGgTGTATAGTTT................................................. 5   | 5; |
| ...TGAGGTAGTAGGTTGNAaAGT................................................... 5   | 5; |
| ...TGAGGTAGTAGGTTGNcaAGTT.................................................. 5   | 5; |
| ...TGAGGTAGTAGGTaNTgTAGTT.................................................. 5   | 5; |
| ...TGAGGTAaTAGGTTGTATAGTT.................................................. 5   | 5; |
| ...TGAGGTAGTAaGTTGTATAGTT.................................................. 5   | 5; |
| ...TGAaGTAGTAGGTTGTATAGTT.................................................. 5   | 5; |
| ...TGAGGTAGTAGGTTGTATAGTca................................................. 5   | 5; |
| ...TGAGGTAGTAGGNTGTgTAGTT.................................................. 5   | 5; |
| ...TGAGGTAGTAGGNTGTAcAGTT.................................................. 5   | 5; |
| ...TGAGGTAGTAGGTaGTATAGTT.................................................. 5   | 5; |
| ...TGAGGTAGTAGGTTGaATAGT................................................... 5   | 5; |
| ...TGAGGTAGTAGtTTGTATtGTT.................................................. 5   | 5; |
| ...cGAGGTAGTAGGTTGTATAGT................................................... 5   | 5; |
| ...TGAGGTAGTAGGTTtTATAGTTa................................................. 5   | 5; |
| ...TGAGGTAGTAGGTTGTATcGTTa................................................. 5   | 5; |
| ...aGAGGTAGTAGGTTGTgTAGTT.................................................. 5   | 5; |
| ...TGAGGTAGTAGGcTGgATAGTT.................................................. 5   | 5; |
| ...TGAGGgAGTAGcTTGTATAGTT.................................................. 5   | 5; |
| ...TGcGGTAGTAGGTTGTATAGTTa................................................. 5   | 5; |
| ...TGAGGTAGTAGGT-GTATAGTTa................................................. 5   | 5; |
| ...TGAGGTAGTAGGTTGNtTAGTT.................................................. 4   | 4; |
| ...TGAcGTAGTAGGTTGTATAGTT.................................................. 4   | 4; |
| ...TGAGGcAGTAGGTTGTgTAGTT.................................................. 4   | 4; |
| ...TGAGGgAGTAGGTTGTAgAGTT.................................................. 4   | 4; |
| ...TGAGGTAGTAGGgTGTATAGTa.................................................. 4   | 4; |
| ...TGAGGTAGTAGGTTGTAcAGTa.................................................. 4   | 4; |
| ...cGAGGTAGTAGGTTGTATAGTTa................................................. 4   | 4; |
| ...TGAGGTAGTAGGTTGTAgAGTTa................................................. 4   | 4; |
| ...TGAGGTAGcAGGTTGTATAGTTa................................................. 4   | 4; |
| ...TGcGGTAGTAGGTTGTATAGTTT................................................. 4   | 4; |
| ...TGAGGaAGTAGGTTGTATAGT................................................... 4   | 4; |
| ...TGAGGTAGTAGGTTcTATAGT................................................... 4   | 4; |
| ...TGAGGgAGTAGGaTGTATAGTTT................................................. 4   | 4; |
| ...TGAGGcAGTAGGTTGTATAGgT.................................................. 4   | 4; |
| ...TGAGGTAGTAGGTTGTATcGT................................................... 4   | 4; |
| ...TGAGGTAGTAGGTgGTATAGTTa................................................. 4   | 4; |
| ...TGAGGTAGTAGGTTGTAgAGgT.................................................. 4   | 4; |
| ...TGAGGTAGTAGGgTGTgTAGTT.................................................. 4   | 4; |
| ...TGAGtTAGTAGGTTGTATAGTT.................................................. 4   | 4; |
| ...TGAGGTgGTAGGTTGTATAGTTa................................................. 4   | 4; |
| ...TGAGGTAGcAGGTTGTATAGT................................................... 4   | 4; |
| ...TGAGGTAGTAGGTTtTATAGTTT................................................. 4   | 4; |
| ...TGAGGTAGTAGGTcNTATAGTTT................................................. 4   | 4; |
| ...TGAGGTAGTAGGTTGTgTAtTT.................................................. 4   | 4; |
| ...TGAGGTAGTAGGTgNTATAGT................................................... 4   | 4; |
| ...aGAGGgAGTAGGTTGTATAGTT.................................................. 4   | 4; |
| ...TGAGGTAGTAGGTTGTATAGaTT................................................. 3   | 3; |
| ...TGAGGTAGTAGGTTGTtTAtTT.................................................. 3   | 3; |
| ...TGAGGTAGTAGtNTGTATAGTT.................................................. 3   | 3; |
| ...gGAGGTAGTAGGTTGTATAGT................................................... 3   | 3; |
| ...TGAGGTAGTAGGTTGNcTAGT................................................... 3   | 3; |
| ...TGAGGgAGTAGGTTGTATtGTT.................................................. 3   | 3; |
| ...TGAGGTAGTAGGTgGTATAGTTT................................................. 3   | 3; |
| ...TGAGGTAGTAGGTTGNAcAGT................................................... 3   | 3; |
| ...TGAGGTAGTAGGTTGTgcAGTTT................................................. 3   | 3; |
| ...TGAGGTAGTAcGTTGTATAGTT.................................................. 3   | 3; |
| ...TGAGGTAGTAGGTTGNAgAGTTa................................................. 3   | 3; |
| ...TGAGGTAGTAGGggGTATAGTT.................................................. 3   | 3; |
| ...TGAGGTAGTAGGTgNTATAGTTa................................................. 3   | 3; |
| ...TGAGGTAGcAGGTTGTATAGTTT................................................. 3   | 3; |
| ...TGgGGTAGTAGGTTGTAgAGTT.................................................. 3   | 3; |
| ...TGAGGTAGTAGGTTaTATAGT................................................... 3   | 3; |
| ...TGAGGTAGTgGGTTGTATAGTTa................................................. 3   | 3; |
| ...cGAGGTAGTAGGTTGTATAGTTT................................................. 3   | 3; |
| ...TGAGGgAGTAGGTTGTATAGgT.................................................. 3   | 3; |
| ...TGAGGTAGTAGGNaGTATAGTT.................................................. 3   | 3; |
| ...TGAGGTAGTAGGTTGTtTAGgT.................................................. 3   | 3; |
| ...TGAGGTAGTAGGNTGTATAGgT.................................................. 3   | 3; |
| ...TGAGGTAGTAGcTTGTATAGTTT................................................. 3   | 3; |
| ...TGAGGgAGTgGGTTGTATAGTT.................................................. 3   | 3; |
| ...TGAGGTAGTAGtTTGNATAGTT.................................................. 3   | 3; |
| ...TGAGGgAGTAGGTTGgATAGT................................................... 3   | 3; |
| ...TGAGGTAGTAGGgTGTtTAGTT.................................................. 3   | 3; |
| ...TGAGGTAGTtGGTTGTATAGTT.................................................. 3   | 3; |
| ...TGAGGTAGTAGGTTGTATAGTcT................................................. 3   | 3; |
| ...TGAGGTtGTAGGTTGTATAGTT.................................................. 3   | 3; |
| ...TGAGGTAGTgGGTTGTATAGTTT................................................. 3   | 3; |
| ...TGAGGTAGTAGGTTGTAaAGTT.................................................. 3   | 3; |
| ...TGAGGTAGTAGGTTGTATAGga.................................................. 3   | 3; |
| ...TGtGGTAGTAGGTTGTATAGTT.................................................. 3   | 3; |
| ...aGAGGTAGTAGGNTGTATAGTT.................................................. 3   | 3; |
| ...TGAGGTAGTcGGTTGTATAGTTT................................................. 3   | 3; |
| ...TtAGGTAGTAGGTTGTATAGT................................................... 3   | 3; |
| ...TGAGGgAGTAGGTTGTtTAGTT.................................................. 3   | 3; |
| ...TGAGGTAGTAGGNTGTATAGTaa................................................. 3   | 3; |
| ...TGAGGTAGTAGcTTGTATAGT................................................... 3   | 3; |
| ...TGAGGTAGTAGGTcGTATAGTTa................................................. 3   | 3; |
| ...TGAGGTAGTAGtTTGTATAGTa.................................................. 3   | 3; |
| ...TGAGGTAGTAGGTTGNAcAGTTa................................................. 3   | 3; |
| ...TGAGGTAGTAGGgTGTATAGg................................................... 3   | 3; |
| ...TGgGGTAGTAGGTTGTgTAGTT.................................................. 3   | 3; |
| ...TGAGGTAGTAGGgTGTATAGTTg................................................. 2   | 2; |
| ...TGAGGTAtTAGGTTGTATAGTTa................................................. 2   | 2; |
| ...TGAGGTAGTAGGgTGTATAGgTT................................................. 2   | 2; |
| ...TGAGGTAGTAGGTTGaATAGTTa................................................. 2   | 2; |
| ...TGAGGTAGTAGGaTGaATAGTT.................................................. 2   | 2; |
| ...TGAGGTAGTAGGTTGgATtGTT.................................................. 2   | 2; |
| ....GAGGTAGTAGGTTGTATAGTa.................................................. 2   | 2; |
| ...TGAGGggGTAGGTTGTATAGTT.................................................. 2   | 2; |
| ...TGAGGgAGaAGGTTGTATAGTT.................................................. 2   | 2; |
| ...TGAGGgAGTAGGTTGTATAGTg.................................................. 2   | 2; |
| ...TGAGGTAGTAGGTaNTcTAGTT.................................................. 2   | 2; |
| ...TGgGGTAGTAGGTTGTtTAGTT.................................................. 2   | 2; |
| ...TGAGGgAGTAGGNTGTATAGTT.................................................. 2   | 2; |
| ...TGAGGTAGTAGGNTGgATAGTT.................................................. 2   | 2; |
| ...TGAGGTAGTAGGTTGNAaAGTa.................................................. 2   | 2; |
| ...TGAGGTAGTAGGNTcTATAGTT.................................................. 2   | 2; |
| ...TGAGGTAGTAGGTTGaATAGTTT................................................. 2   | 2; |
| ...TGAGGTAGTAGGTTGTgTAGTaT................................................. 2   | 2; |
| .....AGGTAGTAGGTTGTATAGTTa................................................. 2   | 2; |
| ...TGAGGTAGTcGGTTGTATAGTTa................................................. 2   | 2; |
| ...TGAGGTAGTAGGTTGNAcAGTTT................................................. 2   | 2; |
| ...TGAGGTAGTAGGcTGTATAGTa.................................................. 2   | 2; |
| ...TGAGGTcGTAGGTTGTATAGTTT................................................. 2   | 2; |
| ...TGAGGTAGTAGGTNNTgTAGTT.................................................. 2   | 2; |
| ...aGAGGTAGTAGGTTGTATAGTaT................................................. 2   | 2; |
| ...aGAGGTAGTAGGTTGTcTAGTT.................................................. 2   | 2; |
| ...TGAGGTAGTAGGTaNTATAGTa.................................................. 2   | 2; |
| ...TGAGGTAGTAtGTTGTATAGTTa................................................. 2   | 2; |
| ...TGAGGTAGTAGGTTGTATcGTTT................................................. 2   | 2; |
| ...TGAGcTAGTAGGTTGTATAGTT.................................................. 2   | 2; |
| ...TGAGGgAGTAGGTTGgATAGTTT................................................. 2   | 2; |
| ...TGAGGTAGTAGGTTGTcTAtTT.................................................. 2   | 2; |
| ...TGAtGTAGTAGGTTGTATAGT................................................... 2   | 2; |
| ...TGAGGTAGTAGGgTGgATAGT................................................... 2   | 2; |
| ...TGAGGTAGTAGGTTGggTAGTTT................................................. 2   | 2; |
| ...TGAGGgAGTAGGTTGTATAtTT.................................................. 2   | 2; |
| ...TGAGGcAGTAGGcTGTATAGTT.................................................. 2   | 2; |
| ...TGAGGTAGTAGGNTGTATAGaT.................................................. 2   | 2; |
| ...TGAGGaAGTAGGTTGTATAGTTT................................................. 2   | 2; |
| ...TGAGGTAGTAGGTTGNcTAGTTa................................................. 2   | 2; |
| ...TGAGGTAGTAGGcTGTATAGgT.................................................. 2   | 2; |
| ...TGAGGTAGTAGGTTGNgTAGT................................................... 2   | 2; |
| ...TGAGGTAGTAGGTTGNAgAGTTT................................................. 2   | 2; |
| ...TGAGGTAGTAGGTTGgAcAGTT.................................................. 2   | 2; |
| ...TGAGGTAGTAGGcTGTgTAGTT.................................................. 2   | 2; |
| ...TGAGGTAGTAGGgTGgATAGTTT................................................. 2   | 2; |
| ...TGAGGTAGTAGGNTGTATAGTTg................................................. 2   | 2; |
| ...TGAGGTAGTAGGTNNTATAGTa.................................................. 2   | 2; |
| ...TGAGGTAGTAGGTTGNATAGTa.................................................. 2   | 2; |
| ...aGAGGTAGTAGGTTGTATAtTT.................................................. 2   | 2; |
| ...TGAGGcAGTAGGTTGTAgAGTT.................................................. 2   | 2; |
| ...TGAGGgAGTAGGTTGTATAGTaT................................................. 2   | 2; |
| ...TGAGGgAGTAGGcTGTATAGTT.................................................. 2   | 2; |
| ...TGAGGTAGTAGcTTGTATAGTTa................................................. 2   | 2; |
| ...TGAGGgAGTAGGTTGTATAGTTg................................................. 2   | 2; |
| ...TGAGaTAGTAGGTTGTATAGTT.................................................. 2   | 2; |
| ...TGAGGaAGTAGGTTGTATAGTTa................................................. 2   | 2; |
| ...TcAGGTAGTAGGTTGTATAGTT.................................................. 2   | 2; |
| ...TGAGGTAGTAGGTTGTActGTT.................................................. 2   | 2; |
| ...TGAGGTAGTAGGTTGTATAGcTa................................................. 2   | 2; |
| ...TGAGGTAGTAGGTTGgATAGTg.................................................. 2   | 2; |
| ...TGAGGTAGTAGGTTGTATAtTa.................................................. 2   | 2; |
| ...TGAGGTAGTAGGTTcTATAGTTa................................................. 2   | 2; |
| ...aGAGGTAGTAGGgTGTATAGTT.................................................. 2   | 2; |
| ...TGAGGTAGTAGGTTGgATAtTT.................................................. 2   | 2; |
| ...TGAGGTAGTcGGTTGTATAGT................................................... 2   | 2; |
| ...TGAGGTAGTAGGTTGTATAtGT.................................................. 2   | 2; |
| ...TGAGGTAGTAGGcTGTtTAGTT.................................................. 2   | 2; |
| ...TGAGGTcGTAGGTTGTATAGTTa................................................. 2   | 2; |
| ...TGAGGTAGTAGGNTGaATAGTT.................................................. 1   | 1; |
| ...TGAGGTAGTAGGNTGTATAGTaT................................................. 1   | 1; |
| ...TGcGGgAGTAGGTTGTATAGTT.................................................. 1   | 1; |
| ...TGAGGTAGTAGGaTGTgTAGTT.................................................. 1   | 1; |
| ...TGAGGTAGTgGGTTGTgTAGTT.................................................. 1   | 1; |
| ...TGAGGTAGTAGcTTGTgTAGTT.................................................. 1   | 1; |
| ...TGAGGgAGTAGGTTGTgTAGTTT................................................. 1   | 1; |
| ...TGAGGaAGTAGGNTGTATAGTT.................................................. 1   | 1; |
| ...TGAGGTAGTAGGTTGTATtGTa.................................................. 1   | 1; |
| ...TGAGGTAGTAGtTTGTATAGgT.................................................. 1   | 1; |
| ...TGAGGTAGTAGGgTGTATcGTT.................................................. 1   | 1; |
| ...TGAGGTAGTAGGTTGTATctTT.................................................. 1   | 1; |
| ...TGAGGTAGTAGGTTGTATAcTa.................................................. 1   | 1; |
| ...TGAGGgAGTAGGTTGTATcGTT.................................................. 1   | 1; |
| ...TGAGGTAGTAGGTTGNccAGTT.................................................. 1   | 1; |
| ...TGAGGTAGTAGtTTGgATAGTT.................................................. 1   | 1; |
| ...TGAGGTAGTAGtTaNTATAGTT.................................................. 1   | 1; |
| ...TGAGGTAGTAGGcTGTATtGTT.................................................. 1   | 1; |
| ...TGAGGTAGTAGtTTGTATcGTT.................................................. 1   | 1; |
| ...TGAGGTAGTAGGTTGgATAGTa.................................................. 1   | 1; |
| ...TGgGGTAGTAGGTTGgATAGTT.................................................. 1   | 1; |
| ...TGAGGTAGTAGGTTGTgTAGaT.................................................. 1   | 1; |
| ....GAGGTAGTAGGTTGTATAGTaT................................................. 1   | 1; |
| ...TGAGGTAGTAGtTNNTATAGTT.................................................. 1   | 1; |
| ...TGAGGTAGTAGGTTGggTAGT................................................... 1   | 1; |
| ...TGAGGTAGTAGGTTGNgcAGTT.................................................. 1   | 1; |
| ...TGAGGTAGTAGGTTGTAcAGTTg................................................. 1   | 1; |
| ...TGgGGgAGTAGGTTGTATAGTT.................................................. 1   | 1; |
| ...TGAGGTAGTAGGTcNTgTAGTT.................................................. 1   | 1; |
| ...aGAGGTAGTAGGTTGTtTAGTT.................................................. 1   | 1; |
| ...aGAGGTAGTAGGTTGTATAGTTg................................................. 1   | 1; |
| ...TGAGGTAGTAGGTTGgtTAGTT.................................................. 1   | 1; |
| ...TGAGGTAGgAGGTTGTgTAGTTT................................................. 1   | 1; |
| ...TGAGGTAGTAGGTTGTAgtGTT.................................................. 1   | 1; |
| ...TGAGGTAGTAGGTTGNgTAGTTT................................................. 1   | 1; |
| ...TGAGGgAGTAGGTTGTATtGT................................................... 1   | 1; |
| ...TGcGGTAGTAGGgTGTATAGTT.................................................. 1   | 1; |
| ..cTGAGGTAGTAGNTTGTATAGTT.................................................. 1   | 1; |
| ...TGAGGTAGTAGGgTGTATtGTT.................................................. 1   | 1; |
| ...TGAGGgAGTAGGTTGTgTAGT................................................... 1   | 1; |
| ...TGAGGTAGTAGGTTGNAgAGT................................................... 1   | 1; |
| ...TGAGGTAGTAGGTTGTtgAGTT.................................................. 1   | 1; |
| ...TGAGGTAGTAGGTTGNtcAGTT.................................................. 1   | 1; |
| ...TGAGGaAGTAGGTTGTAgAGTT.................................................. 1   | 1; |
| ...TGAGGTAGTAGGTTGTtTtGTTT................................................. 1   | 1; |
| ...TGAGGgAGTAGGTTGTATAGcTT................................................. 1   | 1; |
| ...TGAGGTAGTAGGTTGNgTAGTTa................................................. 1   | 1; |
| ...TGAGGgAGTAGGTTtTATAGTT.................................................. 1   | 1; |
| ...TGAGGTAGTAGtTTGTATAtTT.................................................. 1   | 1; |
| ...gGAGGTAGTAGGTTGTATAGTTa................................................. 1   | 1; |
| ...TGAGGTAGTAGGaTGTATAGcT.................................................. 1   | 1; |
| ...TGAGGTAGTAGGTTNTgTAGTT.................................................. 1   | 1; |
| ...TGAGGTAGTAGGTcGTATAGTTT................................................. 1   | 1; |
| ...TGAGGTAGTAGGTTGTATAGag.................................................. 1   | 1; |
| ...TGAGGTAGTAGGTTGNgaAGTT.................................................. 1   | 1; |
| ...TGAGGgAGgAGGTTGTATAGTTT................................................. 1   | 1; |
| ...aGAGGTAGTAGGTTGTATtGTT.................................................. 1   | 1; |
| ...TGAGGTAGTAGtTTNTATAGTT.................................................. 1   | 1; |
| ...gGAGGTAGTAGGTTGTATAGTTT................................................. 1   | 1; |
| ...TGAGGTAGTAGGTTGTAcAGgT.................................................. 1   | 1; |
| ...TGAGGTAGTAGGTTGTgcAGT................................................... 1   | 1; |
| ...TGgGGTAGTAGGTTGTATtGTT.................................................. 1   | 1; |
| -------------------------------------------------------------------------- 27267 |
| -------------------------------------------------------------------------- 187300 |
| hsa-let-7b-3p(hsa-let-7b) CGGGGTGAGGTAGTAGGTTGTGTGGTTTCAGGGCAGTGATGTTGCCCCTCGGAAGATAACTATACAACCTACTGCCTTCCCTG (((((.(((((((((((((((((((((...((((((.....)))))).((....)).)))))))))))))))))))))))))) (-50.60) \*\*\*\*\*\*\*\*\*\*\*\*\*\*\*\*\*\*\*\*\*\*\*\*\*\*\*\*\*\*\*\*\*\*\*\*\*\*\*\*\*\*\*\*\*\*\*\*\*\*\*\*\*\*\*\*\*\*\*CTATACAACCTACTGCCTTCCC\*\* T  M |
| .....TGAGGTAGTAGGTTGTGTGGTT......................................................... 57882   | 57882; |
| .....TGAGGTAGTAGGTTGTGTGGTTT........................................................ 15145   | 15145; |
| .....TGAGGTAGTAGGTTGTGTGGT.......................................................... 10386   | 10386; |
| .....TGAGGTAGTAGGTTGTGTGG........................................................... 3248   | 3248; |
| .....TGAGGTAGTAGGTTGTGTG............................................................ 426   | 426; |
| .....TGAGGTAGTAGGNTGTGTGGTT......................................................... 191   | 191; |
| .....TGAGGTAGTAGGTNNTGTGGTT......................................................... 103   | 103; |
| .....TGAGGTAGTAGGNTGTGTGGTTT........................................................ 102   | 102; |
| .....TGAGGTAGTAGGTTGTGT............................................................. 94   | 94; |
| .....TGAGGTAGTAGGTTGTG.............................................................. 64   | 64; |
| .....TGAGGTAGTAGG................................................................... 63   | 63; |
| ......GAGGTAGTAGGTTGTGTGGTT......................................................... 60   | 60; |
| .....TGAGGTAGTAGGNTGTGTGGT.......................................................... 38   | 38; |
| ......GAGGTAGTAGGTTGTGTGGTTT........................................................ 37   | 37; |
| .....TGAGGTAGTAGGTNNTGTGGTTT........................................................ 35   | 35; |
| .....TGAGGTAGTAGGTTGTGTGNTT......................................................... 33   | 33; |
| .....TGAGGTAGTAGGTTGNGTGGTT......................................................... 32   | 32; |
| .....TGAGGTAGTAGGTTNTGTGGTT......................................................... 29   | 29; |
| .....TGAGGTAGTAGGTTGTGTGGTTTC....................................................... 26   | 26; |
| .....TGAGGTAGTAGGTNNTGTGGT.......................................................... 21   | 21; |
| ...........................................................CTATACAACCTACTGCCTTCC.... 18   | 18; |
| .....TGAGGTAGTAGGTTGT............................................................... 17   | 17; |
| .....TGAGGTAGTAGGTTGTGTGNTTT........................................................ 16   | 16; |
| .....TGAGGTAGTAGGTTGNGTGGTTT........................................................ 13   | 13; |
| .....TGAGGTAGTAGGTTNTGTGGTTT........................................................ 12   | 12; |
| .......AGGTAGTAGGTTGTGTGGTT......................................................... 11   | 11; |
| .....TGAGGTAGTAGGNTGTGTGG........................................................... 10   | 10; |
| ......GAGGTAGTAGGTTGTGTGG........................................................... 9   | 9; |
| ......GAGGTAGTAGGTTGTGTGGT.......................................................... 8   | 8; |
| .....TGAGGTAGTAGGTTNTGTGGT.......................................................... 7   | 7; |
| .....TGAGGTAGTAGGTTGTGTGNNT......................................................... 5   | 5; |
| .....TGAGGTAGTAGGTT................................................................. 4   | 4; |
| .....TGAGGTAGTAGGTTG................................................................ 3   | 3; |
| ----------------------------------------------------------------------------------- 88148 |
| ...........................................................CTATACAACCTACTGCCTTCCt... 23   | 23; |
| ...........................................................CTATACAACCTACTGCCTTCt.... 5   | 5; |
| ----------------------------------------------------------------------------------- 28 |
| ----------------------------------------------------------------------------------- 88176 |
| hsa-let-7b-5p(hsa-let-7b) CGGGGTGAGGTAGTAGGTTGTGTGGTTTCAGGGCAGTGATGTTGCCCCTCGGAAGATAACTATACAACCTACTGCCTTCCCTG (((((.(((((((((((((((((((((...((((((.....)))))).((....)).)))))))))))))))))))))))))) (-50.60) \*\*\*\*\*TGAGGTAGTAGGTTGTGTGGTT\*\*\*\*\*\*\*\*\*\*\*\*\*\*\*\*\*\*\*\*\*\*\*\*\*\*\*\*\*\*\*\*\*\*\*\*\*\*\*\*\*\*\*\*\*\*\*\*\*\*\*\*\*\*\*\* T  M |
| .....TGAGGTAGTAGGTTGTGTGGTT.........................................................\* 57882   | 57882; |
| .....TGAGGTAGTAGGTTGTGTGGTTT........................................................ 15145   | 15145; |
| .....TGAGGTAGTAGGTTGTGTGGT.......................................................... 10386   | 10386; |
| .....TGAGGTAGTAGGTTGTGTGG........................................................... 3248   | 3248; |
| .....TGAGGTAGTAGGTTGTGTG............................................................ 426   | 426; |
| .....TGAGGTAGTAGGNTGTGTGGTT......................................................... 191   | 191; |
| .....TGAGGTAGTAGGTNNTGTGGTT......................................................... 103   | 103; |
| .....TGAGGTAGTAGGNTGTGTGGTTT........................................................ 102   | 102; |
| .....TGAGGTAGTAGGTTGTGT............................................................. 94   | 94; |
| .....TGAGGTAGTAGGTTGTG.............................................................. 64   | 64; |
| .....TGAGGTAGTAGG................................................................... 63   | 63; |
| ......GAGGTAGTAGGTTGTGTGGTT......................................................... 60   | 60; |
| .....TGAGGTAGTAGGNTGTGTGGT.......................................................... 38   | 38; |
| ......GAGGTAGTAGGTTGTGTGGTTT........................................................ 37   | 37; |
| .....TGAGGTAGTAGGTNNTGTGGTTT........................................................ 35   | 35; |
| .....TGAGGTAGTAGGTTGTGTGNTT......................................................... 33   | 33; |
| .....TGAGGTAGTAGGTTGNGTGGTT......................................................... 32   | 32; |
| .....TGAGGTAGTAGGTTNTGTGGTT......................................................... 29   | 29; |
| .....TGAGGTAGTAGGTTGTGTGGTTTC....................................................... 26   | 26; |
| .....TGAGGTAGTAGGTNNTGTGGT.......................................................... 21   | 21; |
| ...........................................................CTATACAACCTACTGCCTTCC.... 18   | 18; |
| .....TGAGGTAGTAGGTTGT............................................................... 17   | 17; |
| .....TGAGGTAGTAGGTTGTGTGNTTT........................................................ 16   | 16; |
| .....TGAGGTAGTAGGTTGNGTGGTTT........................................................ 13   | 13; |
| .....TGAGGTAGTAGGTTNTGTGGTTT........................................................ 12   | 12; |
| .......AGGTAGTAGGTTGTGTGGTT......................................................... 11   | 11; |
| .....TGAGGTAGTAGGNTGTGTGG........................................................... 10   | 10; |
| ......GAGGTAGTAGGTTGTGTGG........................................................... 9   | 9; |
| ......GAGGTAGTAGGTTGTGTGGT.......................................................... 8   | 8; |
| .....TGAGGTAGTAGGTTNTGTGGT.......................................................... 7   | 7; |
| .....TGAGGTAGTAGGTTGTGTGNNT......................................................... 5   | 5; |
| .....TGAGGTAGTAGGTT................................................................. 4   | 4; |
| .....TGAGGTAGTAGGTTG................................................................ 3   | 3; |
| ----------------------------------------------------------------------------------- 88148 |
| .....TGAGGTAGTAGGTTGTGTGGTTa........................................................ 12426   | 12426; |
| .....TGAGGTAGTAGGTTGTGTGGTaa........................................................ 3478   | 3478; |
| .....TGAGGTAGTAGGTTGTGTGGTaT........................................................ 2522   | 2522; |
| .....TGAGGTAGTAGGTTGTGTGGTa......................................................... 1667   | 1667; |
| .....TGAGGTAGTAGGTTGTGTtGTT......................................................... 1185   | 1185; |
| .....TGAGGTAGTAGGTTGTGTaGTT......................................................... 931   | 931; |
| .....TGAGGTAGTAGGTTGTGcGGTT......................................................... 529   | 529; |
| .....TGAGGTAGTAGGTTGTGTGGa.......................................................... 472   | 472; |
| .....TGAGGTAGTAGGTTGTGTGGTTg........................................................ 400   | 400; |
| .....TGAGGTAGTAGGTTGTGTGGaa......................................................... 367   | 367; |
| .....TGAGGgAGTAGGTTGTGTGGTT......................................................... 366   | 366; |
| .....TGAGGTAGTAGGTTGTGTtGTTT........................................................ 267   | 267; |
| .....TGAGGTAGgAGGTTGTGTGGTT......................................................... 224   | 224; |
| .....TGAGGTAGTAGGgTGTGTGGTT......................................................... 194   | 194; |
| .....TGAGGgAGTAGGTTGTGTGGTTT........................................................ 183   | 183; |
| .....TGAGGTAGTAGGTTGTGTGGTag........................................................ 158   | 158; |
| .....TGAGGTAGTAGGTTGgGTGGTT......................................................... 152   | 152; |
| .....TGAGGTAGTAGGTTGTGgGGTT......................................................... 147   | 147; |
| .....TGAGGTAGTAGGTTGTGTtGT.......................................................... 133   | 133; |
| .....TGAGGTAGTAGGTTGTGcGGTTT........................................................ 130   | 130; |
| .....TGAGGTAGTAGGTTGTGTtGTTa........................................................ 129   | 129; |
| .....TGAGGTAGTAGGTTGTGTGGaT......................................................... 127   | 127; |
| .....TGAGGTAGTAGaTTGTGTGGTT......................................................... 126   | 126; |
| .....TGAGGTAGTAGGTTGTGTaGTTT........................................................ 118   | 118; |
| .....TGAGGTAGTAGGTTGTGTGGgT......................................................... 114   | 114; |
| .....TGAGGTAGTAGGTTGgGTGGTTT........................................................ 106   | 106; |
| .....TGAGGTAGTAGGgTGTGTGGTTT........................................................ 105   | 105; |
| .....TGAGGTAGTAGGTTGTGTaGT.......................................................... 103   | 103; |
| .....TGAGGTAGTAGGTTGTGTaGTTa........................................................ 100   | 100; |
| .....TGAGGTAGTAGGTTGTGTGGTTc........................................................ 93   | 93; |
| .....TGAGGgAGTAGGTTGTGTGGTTa........................................................ 92   | 92; |
| .....TGAGGTAGTAGGTTGTtTGGTT......................................................... 84   | 84; |
| .....aGAGGTAGTAGGTTGcGTGGTT......................................................... 82   | 82; |
| .....TGAGGTAGgAGGTTGTGTGGTTT........................................................ 78   | 78; |
| .....TGAGGTAGTAGGcTGTGTGGTT......................................................... 74   | 74; |
| .....TGAGGTAGTAGGTTGcGTGGTT......................................................... 74   | 74; |
| .....TGAGGTAGTAGGTTGTGTGGgTT........................................................ 72   | 72; |
| .....TGAGGTAGTAGGTTGTGTGtTT......................................................... 65   | 65; |
| .....TGAGGTAGTAGGTTGTGctGTTT........................................................ 64   | 64; |
| .....TGAGGTAGTAGGTTGTGcGGTTa........................................................ 63   | 63; |
| .....TGAGGTAGTAGGTaNTGTGGTT......................................................... 59   | 59; |
| .....TGAGGTAGTAGGTTGTGTGGTga........................................................ 57   | 57; |
| .....TGAGGTAGTAGGTTGTGgGGTTT........................................................ 55   | 55; |
| .....TGAGGTAGTAGGNTGTGTGGTTa........................................................ 54   | 54; |
| .....TGAGGTAGTAGGTTGTGTGGTgT........................................................ 53   | 53; |
| .....TGAGGcAGTAGGTTGTGTGGTT......................................................... 50   | 50; |
| .....TGAGGTAGTAGGTTGTGcGGT.......................................................... 50   | 50; |
| .....TGAGGTAGTAGGTTGTGTGGaaT........................................................ 50   | 50; |
| .....TGAGGTAGTAGtTTGTGTtGTTT........................................................ 49   | 49; |
| .....TGAGGTAGTAGGTTGTGTGGTg......................................................... 48   | 48; |
| .....TGAGGgAGTAGGTTGTGTGGT.......................................................... 46   | 46; |
| .....TGAGGTAGTAGGgTGTGTGGTTa........................................................ 45   | 45; |
| .....TGAGGTAGTAGGTcNTGTGGTT......................................................... 44   | 44; |
| .....TGAGGTAGTAGGgTGTGTGGT.......................................................... 41   | 41; |
| .....TGgGGTAGTAGGTTGTGTGGTT......................................................... 40   | 40; |
| .....TGAGGTAGTAGGTTGgGTGGTTa........................................................ 39   | 39; |
| .....TGAGGTAGTAGGTTGTGgGGT.......................................................... 36   | 36; |
| .....aGAGGTAGTAGGTTGTGTGGTT......................................................... 34   | 34; |
| .....TGAGGTAGTAGGaTGTGTGGTT......................................................... 34   | 34; |
| .....TGAGGTAGTAGGTTGTGTGtT.......................................................... 33   | 33; |
| .....TGAGGTAGTAGaTTGTGTGGTTT........................................................ 33   | 33; |
| .....TGAGGTAGTAGGcTGTGTGGTTT........................................................ 32   | 32; |
| .....TGcGGTAGTAGGTTGTGTGGTT......................................................... 29   | 29; |
| .....TGAGGTAGTAGGTaNTGTGGTTT........................................................ 29   | 29; |
| .....TGAGGTAGTAGGTcNTGTGGTTT........................................................ 28   | 28; |
| .....TGAGGTAGTAGGTTGgGTGGT.......................................................... 27   | 27; |
| ....cTGAGGTAGTAGGTTGTGTGGTT......................................................... 26   | 26; |
| .....TGAGGTAGTAGGTTGcGTGGTTT........................................................ 26   | 26; |
| .....TGAGGTAGgAGGTTGTGTGGTTa........................................................ 25   | 25; |
| .....TGAGGcAGTAGGTTGTGTGGTTT........................................................ 25   | 25; |
| .....aGAGGTAGTAGGTTGcGTGGTTT........................................................ 24   | 24; |
| .....TGAGGTAGTAGGTTGTGTGaa.......................................................... 24   | 24; |
| .....TGAGGTAGTAGGTTGTGTGtTTT........................................................ 23   | 23; |
| .....TGAGGTgGTAGGTTGTGTGGTT......................................................... 22   | 22; |
| .....TGAGGTAGTAGaTTGTGTGGTTa........................................................ 22   | 22; |
| .....TGAGGTAGTAGGTTGTGgGGTTa........................................................ 22   | 22; |
| .....TGAGGTAGTAGGTTGTGTGGg.......................................................... 21   | 21; |
| .....TGAGGTAGTAGtTTGTGTGGTT......................................................... 20   | 20; |
| .....TGAGGTAGTgGGTTGTGTGGTT......................................................... 20   | 20; |
| .....TGAGGTAGTAGGTTGTGTGGaTT........................................................ 19   | 19; |
| .....TGAGGTAGTAGGTTGTGTGGcT......................................................... 19   | 19; |
| .....TGAGGTAGTAGGTTGTGTGGTc......................................................... 19   | 19; |
| .....TGAGGTAGTAGGTTGTtTGGTTT........................................................ 19   | 19; |
| .....TGgGGTAGTAGGTTGTGTGGTTT........................................................ 18   | 18; |
| .....aGAGGTAGTAGGTTGTGTGGTTT........................................................ 18   | 18; |
| .....TGAGGTAGTAGGTTtTGTGGTT......................................................... 18   | 18; |
| .....TGAGGTAGgAGGgTGTGTGGTT......................................................... 18   | 18; |
| .....TGAGGTAGTAGGTNNTGTGGTTa........................................................ 18   | 18; |
| .....TGAGGgAGTAGGTTGTGTaGTT......................................................... 17   | 17; |
| .....TGAGGTAGTAGGTgGTGTGGTT......................................................... 17   | 17; |
| .....TGAGGTAGTAGGTTGTGTcGTT......................................................... 17   | 17; |
| .....TGAGGTAGcAGGTTGTGTGGTT......................................................... 17   | 17; |
| .....TGAGGTAGTAGGTTGaGTGGTT......................................................... 16   | 16; |
| .....TGAGGTAGgAGGTTGTGTGGT.......................................................... 16   | 16; |
| .....cGAGGTAGTAGGTTGTGTGGTT......................................................... 16   | 16; |
| .....TGAGGTAGTAGGTTGTGTGGTac........................................................ 16   | 16; |
| .....TGcGGTAGTAGGTTGTGTGGTTT........................................................ 16   | 16; |
| .....TGAGGTAGTAGGNTGTGTGGTaa........................................................ 16   | 16; |
| .....TGAGGTAGTAGGaTGTGTGGTTT........................................................ 15   | 15; |
| .....TGAGGTAGTAGGTTGTGcaGTT......................................................... 15   | 15; |
| .....TGAGGTAGTAGGTTGTGTGGgTa........................................................ 15   | 15; |
| .....TGAGGTAGgAGGgTGTGTGGTTT........................................................ 15   | 15; |
| .....TGAGGTAGTgGGTTGTGTGGTTT........................................................ 15   | 15; |
| .....TGAGGcAGTAGGTTGTGTGGTTa........................................................ 15   | 15; |
| .....TGAGGgAGTAGGTTGTGTGGTaT........................................................ 15   | 15; |
| .....TGAGGTAGTAGGcTGTGTGGTTa........................................................ 15   | 15; |
| .....TGAGGTAGTAGGTgNTGTGGTT......................................................... 14   | 14; |
| .....TGAGGTAGTAGaTTGTGTtGTT......................................................... 14   | 14; |
| .....TGAGGTAGgAGGTTGTGTGGgTT........................................................ 14   | 14; |
| .....TGAGGTAGTAGGTTGTtTGGTTa........................................................ 14   | 14; |
| .....TGAGGTAGTAGGTTGTGTGGcTT........................................................ 14   | 14; |
| .....TGAGGTAGTAGGTcGTGTGGTT......................................................... 14   | 14; |
| .....TGAGGTAGTAGGTgGTGTGGTTT........................................................ 13   | 13; |
| .....TGAGGTAGgAGGTTGTGTGGgT......................................................... 13   | 13; |
| .....TGAGGTAGTAGGTTGTtTGGT.......................................................... 12   | 12; |
| .....TGAGGgAGTAGGTgGTGTGGTT......................................................... 12   | 12; |
| .....TGAGGTAGTAGtTTGTGTGGTTT........................................................ 12   | 12; |
| .....TGAGGTAGTAGGgTGTGTGGgT......................................................... 12   | 12; |
| .....TGAGGTAGTAGGTTtTGTGGTTT........................................................ 12   | 12; |
| .....TGAGGTAGTAGGTTGTGTGGgaT........................................................ 12   | 12; |
| .....TGAGGgAGTAGGTTGTGcGGTT......................................................... 12   | 12; |
| .....TGAGGTAGTAGGTcGTGTGGTTT........................................................ 12   | 12; |
| .....TGAGGgAGTAGGTTGTGTGGTa......................................................... 12   | 12; |
| .....TGAGGTAGTAGGgTGgGTGGTT......................................................... 12   | 12; |
| .....TGAGGTAGTAGGTTGTcTGGTT......................................................... 11   | 11; |
| .....TGAG-TAGTAGGTTGTGTGGTT......................................................... 11   | 11; |
| .....TGAGGTAGTAGGTTGTGTGGTcT........................................................ 11   | 11; |
| .....TGAGGTAGTAGGgTGTGTGGTaT........................................................ 11   | 11; |
| .....TGAGGTAGgAGGTTGTGTtGTT......................................................... 10   | 10; |
| .....TGAGGTAGcAGGTTGTGTGGTTT........................................................ 10   | 10; |
| .....cGAGGTAGTAGGTTGTGTGGTTT........................................................ 10   | 10; |
| .....TGAGGTAGTAGGTTcTGTGGTT......................................................... 10   | 10; |
| .....TGAGGgAGTAGGTTGTGTtGTT......................................................... 10   | 10; |
| .....TGAGGTAGTAGGTcGTGTGGT.......................................................... 10   | 10; |
| .....TGAGGaAGTAGGTTGTGTGGTT......................................................... 10   | 10; |
| .....TGgGGTAGTAGGTTGTtGTGGT......................................................... 10   | 10; |
| .....TGAGGTAGTAGGTTGTGgGGgT......................................................... 9   | 9; |
| .....TGAGGgAGTAGGTTGgGTGGTTT........................................................ 9   | 9; |
| .....TGAGGTAtTAGGTTGTGTGGTT......................................................... 9   | 9; |
| .....TGAGGgAGTAGGgTGTGTGGTT......................................................... 9   | 9; |
| .....TGAGGTAGTAGGNTGTGTGGTaT........................................................ 9   | 9; |
| .....TGAGGTAGTAGaTTGTGTGGT.......................................................... 9   | 9; |
| .....TGAGGTAGaAGGTTGTGTGGTT......................................................... 9   | 9; |
| .....TGAGGTAGTAGGTaNTGTGGT.......................................................... 9   | 9; |
| .....TGAGGTAGTAGtTTGTGTGGTTa........................................................ 9   | 9; |
| .....TGAGGgAGTAGGTTGTGTGGcTT........................................................ 9   | 9; |
| .....TGAGGTAGTAGGTTGTGTGNTTa........................................................ 9   | 9; |
| .....TGAGGTAGTAGGTTGTG-GtT.......................................................... 9   | 9; |
| .....TGAGGTAGTAGGTaNTGTGGTTa........................................................ 9   | 9; |
| .....TGAGGTcGTAGGTTGTGTGGTTT........................................................ 9   | 9; |
| .....TGAGGgAGTAGGTTGgGTGGTT......................................................... 9   | 9; |
| .....TGAGGcAGTAGGTTGTGTGGT.......................................................... 9   | 9; |
| .....TGAGGTAGTAGGTTGNGcGGTT......................................................... 9   | 9; |
| .....TGAGGTAGTAGGTcNTGTGGTTa........................................................ 8   | 8; |
| .....TGAGGTAGTAGGTTGNGTaGTT......................................................... 8   | 8; |
| .....TGAGGTAGTAGGTTGTGTaGGT......................................................... 8   | 8; |
| ......GAGGTAGTAGGTTGTGTGGTTaT....................................................... 8   | 8; |
| .....TGAGGgAGTAGGgTGTGTGGTTT........................................................ 8   | 8; |
| .....TGAGGTAGTAGGcTGTGTGGT.......................................................... 8   | 8; |
| .....TGAGGTAGTAGGTTGTG-GtTT......................................................... 8   | 8; |
| .....TGAGGTAGTAGGTTGgGTaGTT......................................................... 8   | 8; |
| .....TGAGGTAGTAGGTTGTGTGaT.......................................................... 8   | 8; |
| .....TGAGGTAGTAGGTTGaGTGGTTT........................................................ 8   | 8; |
| .....TGAGGgAGgAGGTTGTGTGGTT......................................................... 8   | 8; |
| .....TGAGGTAGTAGGTTcTGTGGTTT........................................................ 7   | 7; |
| .....TGAGGTAGTAGGTgNTGTGGT.......................................................... 7   | 7; |
| .....TGAGGgAGTAGGTTGTGTGGcT......................................................... 7   | 7; |
| .....TGAGGTAGTAGGTTGTGTaGTa......................................................... 7   | 7; |
| ......GAGGTAGTAGGTTGTGTGGTTTt....................................................... 7   | 7; |
| .....TGAGGTgGTAGGTTGTGTGGTTT........................................................ 7   | 7; |
| .....TGAGGTAGTgGGTTGTGTGGTTa........................................................ 7   | 7; |
| .....TGAGGTAGTAGtTTGTGTGGT.......................................................... 7   | 7; |
| .....TGAGGTAGTAGGTgNTGTGGTTT........................................................ 7   | 7; |
| .....TGAGGTAGTAGGaTGTGTGGTTa........................................................ 7   | 7; |
| .....TGAGGgAGTAGGTTGTGgGGTT......................................................... 7   | 7; |
| .....TGAGGTAGTAGGTTGTGTGGga......................................................... 7   | 7; |
| .....TGcGGTAGTAGGTTGTGTGGTTa........................................................ 7   | 7; |
| .....TGAGGTAGgAGGTTGTGgGGTT......................................................... 7   | 7; |
| .....TGgGGTAGTAGGTTGTGTGGT.......................................................... 7   | 7; |
| .....TGAGGTAGTAGGTTNTGTGGTTa........................................................ 6   | 6; |
| ....cTGAGGTAGTAGGTTGTGTGG........................................................... 6   | 6; |
| .....TGAGGTAGTAGGTaGTGTGGTT......................................................... 6   | 6; |
| .....TGAGGTAGTAGGTTGTGTGGTca........................................................ 6   | 6; |
| .....TGAGGTAGgAGGgTGTGTGGT.......................................................... 6   | 6; |
| .....TGAGGTAGTAGGT-GTGTGGTT......................................................... 6   | 6; |
| ......GAGGTAGTAGGTTGTGTGGTTa........................................................ 6   | 6; |
| .....TGAGGTAGTAGGgTGgGTGGTTT........................................................ 6   | 6; |
| .....TGAGGTAGTAGGTcNTGTGGT.......................................................... 6   | 6; |
| .....TGAGGTAGTAGGTTGgGTGGTgT........................................................ 6   | 6; |
| .....TGAGGTAGTAGGTTGTGcGGTaT........................................................ 6   | 6; |
| .....TGAGGTAGTAGGTTGTtTtGTT......................................................... 6   | 6; |
| .....TGAGGTAcTAGGTTGTGTGGTT......................................................... 6   | 6; |
| .....TGAGGTAGTAGGTTGTGTaGTTg........................................................ 6   | 6; |
| .....TGAGGTAGgAGGTTGTGTGGTaT........................................................ 6   | 6; |
| .....TGAGGgAGTAGGTTGTtTGGTT......................................................... 6   | 6; |
| .....TGAGGTAGTAGGgTGTGTGGgTT........................................................ 6   | 6; |
| .....TGAGtTAGTAGGTTGTGTGGTTT........................................................ 6   | 6; |
| .....TtAGGTAGTAGGTTGTGTGGTT......................................................... 6   | 6; |
| .....TGAGGTAGTAGGTTGTGTGGag......................................................... 6   | 6; |
| .....TGAGGTAGTAGGTTaTGTGGTT......................................................... 6   | 6; |
| .....TGAGGTAGTAGGTTGTGTGta.......................................................... 6   | 6; |
| .....TGAGGTgGTAGGTTGTGTGGT.......................................................... 6   | 6; |
| .....aGAGGTAGTAGGTTGcGTGGT.......................................................... 6   | 6; |
| .....TGAGGTAGTAGGTNNTGTGGTaa........................................................ 6   | 6; |
| .....TGAGtTAGTAGGTTGTGTGGTT......................................................... 6   | 6; |
| .....TGAGGTAGTAGGTTGgGTGGgT......................................................... 5   | 5; |
| .....TGAGGaAGTAGGTTGTGTGGTTT........................................................ 5   | 5; |
| .....TGAGGTAGTAGGTTGaGTGGT.......................................................... 5   | 5; |
| .....TGAGGTAGTAGtTTGTGTaGTT......................................................... 5   | 5; |
| ......GAGGTAGTAGGTTGTGTGGTaa........................................................ 5   | 5; |
| .....TGAGGT--TAGGTTGTGTGGTT......................................................... 5   | 5; |
| .....TGAGGTAGTAGGTTaTGTGGTTa........................................................ 5   | 5; |
| .....TGAGGTAGTAGGTTaTGTGGTTT........................................................ 5   | 5; |
| .....TGAGGTAGTAGGTTtTGTGGTTa........................................................ 5   | 5; |
| .....TGAtGTAGTAGGTTGTGTGGTT......................................................... 5   | 5; |
| .....TGAGGTAGTAGGTTcTGTGGT.......................................................... 5   | 5; |
| .....aGAGGTAGTAGGTTGTGTGGTTa........................................................ 5   | 5; |
| .....TGAGGTAGTAGGTTGTGaGGTTT........................................................ 5   | 5; |
| .....TGAGGTAGTAGGTTGcGTGGT.......................................................... 5   | 5; |
| .....TGAGGTAGTAGGTTGTGTGaG.......................................................... 5   | 5; |
| .....TGAGGTAGTAGGTgGTGTGGT.......................................................... 5   | 5; |
| .....TGAGGTAGTtGGTTGTGTGGTT......................................................... 5   | 5; |
| .....TGAGGTAGTAGGTTGNGcGGTTT........................................................ 5   | 5; |
| .....TGAGGTAGTcGGTTGTGTGGTT......................................................... 5   | 5; |
| .....TGAGGTAGTAGGTTGNGTGGTTa........................................................ 5   | 5; |
| .....aGAGGTAGTAGGTTGTGTaGTT......................................................... 5   | 5; |
| ......GAGGTAGTAGGTTGTGTGGTaT........................................................ 5   | 5; |
| .....aGAGGTAGTAGGTTGTGTtGTT......................................................... 5   | 5; |
| .....TGAGGTAGTAGGNTGTGTaGTT......................................................... 5   | 5; |
| .....TGAGGTAGTAGGNTGTGTGGTa......................................................... 5   | 5; |
| .....TGAGGTAGTAGGTTGgGTGGTaT........................................................ 5   | 5; |
| .....TGAGGTcGTAGGTTGTGTGGTT......................................................... 5   | 5; |
| .....TGAGGTAGTAGGTaNTGTGGTaT........................................................ 5   | 5; |
| .....TGAGGTAGTAGGTaNTGTaGTT......................................................... 5   | 5; |
| .....TtAGGTAGTAGGTTGTGTGGTTa........................................................ 5   | 5; |
| .....cGAGGTAGTAGGTTGTGTGGTTa........................................................ 5   | 5; |
| .....TGAGGTAGTAGGTTGTGTGGcTa........................................................ 5   | 5; |
| .....TGAGGTAGgAGGTTGTGTtGTTT........................................................ 5   | 5; |
| ......GAGGTAGTAGGTTGTGTGGTTaa....................................................... 4   | 4; |
| .....TGAGGTAGTAGGTTGTcTGGTTT........................................................ 4   | 4; |
| .....TGAGGTAGTAGGgTGTGTaGTT......................................................... 4   | 4; |
| .....TGAGGTAGTAGGTTGNtTGGTTT........................................................ 4   | 4; |
| .....TGAGGcAGTAGGTTGTGTaGTT......................................................... 4   | 4; |
| .....TGAGGgAGTAGGaTGTGTGGTTT........................................................ 4   | 4; |
| .....TGAGGTAGTAGGTTGTGTatTT......................................................... 4   | 4; |
| .....TGgGGTAGTAGGTTGTGTaGTT......................................................... 3   | 3; |
| .....TGAGGTAGTAGGTTGTGgtGTT......................................................... 3   | 3; |
| .....TGAGGgAGTAGGgTGTGTGGT.......................................................... 3   | 3; |
| .....TGAGGTAGgAGGTTGTGTGGTTg........................................................ 3   | 3; |
| .....TGAGGTAGTAGGTTGTGcaGTTT........................................................ 3   | 3; |
| .....TGAGGgAGTAGGTTGTGTGGTTg........................................................ 3   | 3; |
| .....TGAGGTAGTAGaTTGTGTtGTTT........................................................ 3   | 3; |
| .....TGAGGTAGTAGGTTGgGTaGTTT........................................................ 2   | 2; |
| .....TGAGGTAGTAGGTTGNcTGGTT......................................................... 2   | 2; |
| .....TGAGGTAGTAGGTTGNGTaGT.......................................................... 2   | 2; |
| .....TGAGGTAGTAGGTTGTGTGGTTTnCtNtNG................................................. 2   | 2; |
| .....TGAGGTAGTAGGTNNTGTaGTT......................................................... 2   | 2; |
| .....TGAGGTAGTAGGTTGTGTaGTaT........................................................ 2   | 2; |
| .....TGAGGTAGTAGGcTGTGTaGTT......................................................... 2   | 2; |
| .....TGAGGTAGTAGaTTGTGTtGT.......................................................... 2   | 2; |
| .....TGAGGTAGTAGGTTGTGTtGTTg........................................................ 2   | 2; |
| .....TGAGGTAGTAGGTTNTGTaGTT......................................................... 1   | 1; |
| .....TGAGGTAGTAGGTTGNGcaGTT......................................................... 1   | 1; |
| .....TGAGGTAGTAGaTTGTtTGGTT......................................................... 1   | 1; |
| .....TGAGGTAGgAGGTTGTGTaGTTT........................................................ 1   | 1; |
| .....TGAGGTAGTAGGTcNTGTaGTT......................................................... 1   | 1; |
| .....TGAGGgAGTAGGTTGTGTaGT.......................................................... 1   | 1; |
| .....TGAGGTAGTAGGTTGTtTtGTTT........................................................ 1   | 1; |
| .....TGAGGTAGTAGGTTGNGTaGTTT........................................................ 1   | 1; |
| .....TGAGGTAGTAGGTTGNGTaGTTa........................................................ 1   | 1; |
| .....TGAGGTAGTAGcTTGTGTaGTT......................................................... 1   | 1; |
| .....TGAGGTAGTAGGTTGNGaaGTT......................................................... 1   | 1; |
| .....TGAGGTAGTAGtTTGTGcGGTTT........................................................ 1   | 1; |
| .....TGAGGTAGTAGGTTGgGTaGT.......................................................... 1   | 1; |
| .....TGAGGTAGTAGGTTGTGTaGaT......................................................... 1   | 1; |
| .....TGAGGTAGTgGGTTGTGTaGTT......................................................... 1   | 1; |
| .....TGAGGTAGTAGGaTGTGTaGTT......................................................... 1   | 1; |
| .....TGAGGgAGTAGGTTGTGTaGTTT........................................................ 1   | 1; |
| .....TGAGGTAGTAGGTTGTGcaGT.......................................................... 1   | 1; |
| ----------------------------------------------------------------------------------- 30751 |
| ----------------------------------------------------------------------------------- 118899 |
| hsa-let-7c(hsa-let-7c) GCATCCGGGTTGAGGTAGTAGGTTGTATGGTTTAGAGTTACACCCTGGGAGTTAACTGTACAACCTTCTAGCTTTCCTTGGAGC ((.((((((..(((.(((.(((((((((((((..((.(..(.....)..).))))))))))))))).))).)))..)))))))) (-33.50) \*\*\*\*\*\*\*\*\*\*TGAGGTAGTAGGTTGTATGGTT\*\*\*\*\*\*\*\*\*\*\*\*\*\*\*\*\*\*\*\*\*\*\*\*\*\*\*\*\*\*\*\*\*\*\*\*\*\*\*\*\*\*\*\*\*\*\*\*\*\*\*\* T  M |
| ..........TGAGGTAGTAGGTTGTATGGTT.....................................................\* 4004   | 4004; |
| ..........TGAGGTAGTAGGTTGTATGGTTT.................................................... 551   | 551; |
| ..........TGAGGTAGTAGGTTGTATGGT...................................................... 458   | 458; |
| ..........TGAGGTAGTAGGTTGTAT......................................................... 114   | 114; |
| ..........TGAGGTAGTAGG............................................................... 63   | 63; |
| ..........TGAGGTAGTAGGTTGTATGG....................................................... 49   | 49; |
| ..........TGAGGTAGTAGGTTGTA.......................................................... 35   | 35; |
| ..........TGAGGTAGTAGGNTGTATGGTT..................................................... 26   | 26; |
| ..........TGAGGTAGTAGGTTGT........................................................... 17   | 17; |
| ..........TGAGGTAGTAGGTTGNATGGTT..................................................... 11   | 11; |
| ..........TGAGGTAGTAGGNTGTATGGTTT.................................................... 8   | 8; |
| ..........TGAGGTAGTAGGTTGTATGGTTTA................................................... 7   | 7; |
| ..........TGAGGTAGTAGGTT............................................................. 4   | 4; |
| ..........TGAGGTAGTAGGTTG............................................................ 3   | 3; |
| ------------------------------------------------------------------------------------ 5350 |
| ..........TGAGGTAGTAGGTTGTATGGTTa.................................................... 395   | 395; |
| ..........TGAGGTAGTAGGTTGTAcGGTT..................................................... 170   | 170; |
| ..........TGAGGTAGTAGGTTGTATtGTT..................................................... 161   | 161; |
| ..........TGAGGTAGTAGaTTGTATGGTT..................................................... 89   | 89; |
| ..........TGAGGTAGTAGGTTGTtTGGTT..................................................... 84   | 84; |
| ..........TGAGGTAGTAGGTTGTATGGTa..................................................... 66   | 66; |
| ..........TGAGGgAGTAGGTTGTATGGTT..................................................... 39   | 39; |
| ..........TGAGGTAGTAGGTTGTA-GtT...................................................... 39   | 39; |
| ..........TGAGGTAGTAGGTTGTATcGTT..................................................... 37   | 37; |
| ..........TGAGGTAGTAGGTTGTATGGTaa.................................................... 33   | 33; |
| ..........TGAGGTAGTAGGTTGTATGGTaT.................................................... 32   | 32; |
| ..........TGAGGTAGgAGGTTGTATGGTT..................................................... 25   | 25; |
| ..........TGAGGTAGTAGGTTGTATtGT...................................................... 23   | 23; |
| ..........TGAGGTAGTAGGTTGTtTGGTTT.................................................... 19   | 19; |
| ..........TGAGGTAGTAGGTTGTATtGTTa.................................................... 19   | 19; |
| ..........TGAGGTAGTAGGTTGTATtGTTT.................................................... 19   | 19; |
| ..........TGAGGTAGTAGGgTGTATGGTT..................................................... 19   | 19; |
| ..........TGAGGTAGTAGGTTGTATGGTTg.................................................... 19   | 19; |
| ..........aGAGGTAGTAGGTTGTATGGTT..................................................... 17   | 17; |
| ..........TGAGGTAGTAGGTTGgATGGTT..................................................... 16   | 16; |
| ..........TGAGGTAGTAGGTTGTAcGGTTT.................................................... 15   | 15; |
| ..........TGAGGTAGTAGGTTGTtTGGTTa.................................................... 14   | 14; |
| ..........TGAGGTAGTAGGTTGTATtGGT..................................................... 13   | 13; |
| ..........TGAGGTAGTAGGTTGTAgGGTT..................................................... 13   | 13; |
| ..........TGAGGTAGTAGGTTGTAcGGT...................................................... 12   | 12; |
| ..........TGAGGTAGTAGGTTGTtTGGT...................................................... 12   | 12; |
| ..........TGAGGTAGTAGGTTGTAcGGTTa.................................................... 12   | 12; |
| ..........TGAGGTAGTAGGTTGTATGtTT..................................................... 11   | 11; |
| ..........TGAGGTAGTAGGTTGTcTGGTT..................................................... 11   | 11; |
| ..........TGAGGTAGTAGGTTGcATGGTT..................................................... 10   | 10; |
| ..........TGAGGTAGTAGGTTGTATGGa...................................................... 10   | 10; |
| ..........TGAGGTAGTAGGTTGTA-GtTT..................................................... 10   | 10; |
| ..........TGAGGTAGTAGaTTGTATGGT...................................................... 10   | 10; |
| ..........TGAGGTAGTtGGTTGTATGGTT..................................................... 9   | 9; |
| ..........TGAGGgAGTAGGTTGTATGGTTT.................................................... 9   | 9; |
| ..........TGAGGTAGTAGGTTGT--GGTT..................................................... 9   | 9; |
| ..........TGgGGTAGTAGGTTGTATGGTT..................................................... 9   | 9; |
| ..........TGAGGTAGTAGtTTGTATGGTT..................................................... 8   | 8; |
| ..........TGAGGTAGTAGaTTGTATGGTTa.................................................... 8   | 8; |
| ..........TGAGGTAGTAGGTTGT--GGTTT.................................................... 8   | 8; |
| ..........TGAGGTAGTAGGTTGTATGGaa..................................................... 7   | 7; |
| ..........TGcGGTAGTAGGTTGTATGGTT..................................................... 7   | 7; |
| ..........TGAGGTAGTgGGTTGTATGGTT..................................................... 7   | 7; |
| ..........TGgGGTAGTAGGTTGTAgGGTT..................................................... 6   | 6; |
| ..........TGAGGTAGTAGaTTGTATGGTTT.................................................... 6   | 6; |
| ..........TGAGGTAGTAGGTTGTtTtGTT..................................................... 6   | 6; |
| ..........TGAGGgAGTAGGTTGTtTGGTT..................................................... 6   | 6; |
| ..........TGAGGgAGTAGGTTGTATGGTTa.................................................... 6   | 6; |
| ..........TGAGGTAGTAGGTgGTATGGTT..................................................... 5   | 5; |
| ..........TGAGGcAGTAGGTTGTATGGTT..................................................... 5   | 5; |
| ..........TGAGGTAGTAGGTTGNAcGGTTT.................................................... 5   | 5; |
| ..........TGAGGTAGTAGGTTGTATcGTTa.................................................... 5   | 5; |
| ..........TGAGGTAGTAGtTTGTATtGTT..................................................... 5   | 5; |
| ...........GAGGTAGTAGGTTGTgTGGTTaA................................................... 4   | 4; |
| ..........TGAGGTAGTAGGTTGNtTGGTTT.................................................... 4   | 4; |
| ..........TGAGGTAGTAGGTTGTATcGT...................................................... 4   | 4; |
| ..........TGAGGTAGTAGGTTGTcTGGTTT.................................................... 4   | 4; |
| ..........TGAGGgAGTAGaTTGTATGGTT..................................................... 3   | 3; |
| ..........TGAGGgAGTAGGTTGTATtGTT..................................................... 3   | 3; |
| ..........TGAGGgAGTAGGTTGTATGGT...................................................... 3   | 3; |
| ..........TGAGGTAGgAGGTTGTATGGT...................................................... 2   | 2; |
| ..........TGAGGTAGTAGGTTGTATcGTTT.................................................... 2   | 2; |
| ..........TGAGGTAGTAGGTTGNcTGGTT..................................................... 2   | 2; |
| ..........TGAGGTAGTAGGTTGgATtGTT..................................................... 2   | 2; |
| ..........TGAGGTAGTAGGTTGTActGTT..................................................... 2   | 2; |
| ..........TGAGGTAGTAGGTTGTtTtGTTT.................................................... 1   | 1; |
| ..........TGgGGTAGTAGGTTGTATtGTT..................................................... 1   | 1; |
| ..........TGAGGTAGTAGGcTGTATtGTT..................................................... 1   | 1; |
| ..........TGAGGTAGTAGGgTGTATcGTT..................................................... 1   | 1; |
| ..........TGAGGTAGTAGtTTGTATcGTT..................................................... 1   | 1; |
| ..........TGAGGTAGTAGGTTGTAgtGTT..................................................... 1   | 1; |
| ..........TGAGGgAGTAGGTTGTATcGTT..................................................... 1   | 1; |
| ..........TGAGGTAGTAGGgTGTATtGTT..................................................... 1   | 1; |
| ..........TGAGGTAGTAGGTTGTATtGTa..................................................... 1   | 1; |
| ..........TGAGGTAGTAGaTTGTtTGGTT..................................................... 1   | 1; |
| ..........aGAGGTAGTAGGTTGTATtGTT..................................................... 1   | 1; |
| ..........TGAGGTAGgAGaTTGTATGGTT..................................................... 1   | 1; |
| ..........TGAGGgAGTAGGTTGTATtGT...................................................... 1   | 1; |
| ..........TGAGGTAGTAGGTTGTATctTT..................................................... 1   | 1; |
| ------------------------------------------------------------------------------------ 1659 |
| ------------------------------------------------------------------------------------ 7009 |
| hsa-let-7d-3p(hsa-let-7d) CCTAGGAAGAGGTAGTAGGTTGCATAGTTTTAGGGCAGGGATTTTGCCCACAAGGAGGTAACTATACGACCTGCTGCCTTTCTTAGG (((((((.((((((((((((((.((((((...((((((.....)))))).(.....)..)))))).))))))))))))))))))))) (-42.60) \*\*\*\*\*\*\*\*\*\*\*\*\*\*\*\*\*\*\*\*\*\*\*\*\*\*\*\*\*\*\*\*\*\*\*\*\*\*\*\*\*\*\*\*\*\*\*\*\*\*\*\*\*\*\*\*\*\*\*\*\*CTATACGACCTGCTGCCTTTCT\*\*\*\* T  M |
| .......AGAGGTAGTAGGTTGCATAGTT........................................................... 40680   | 40680; |
| .......AGAGGTAGTAGGTTGCATAGTTT.......................................................... 8432   | 8432; |
| .......AGAGGTAGTAGGTTGCATAGT............................................................ 5273   | 5273; |
| .......AGAGGTAGTAGGTTGCATAG............................................................. 1659   | 1659; |
| .......AGAGGTAGTAGGTTGCATAGTTTT......................................................... 317   | 317; |
| .......AGAGGTAGTAGGTTGCATA.............................................................. 235   | 235; |
| .......AGAGGTAGTAGGNTGCATAGTT........................................................... 216   | 216; |
| .............................................................CTATACGACCTGCTGCCTTTCT.....\* 150   | 150; |
| .......AGAGGTAGTAGGTTGCAT............................................................... 89   | 89; |
| ........GAGGTAGTAGGTTGCATAGTT........................................................... 85   | 85; |
| .......AGAGGTAGTAGGTTGNATAGTT........................................................... 81   | 81; |
| .............................................................CTATACGACCTGCTGCCTTTC...... 57   | 57; |
| .......AGAGGTAGTAGGTNNCATAGTT........................................................... 44   | 44; |
| .......AGAGGTAGTAGGNTGCATAGTTT.......................................................... 42   | 42; |
| .......AGAGGTAGTAGGTTGCA................................................................ 39   | 39; |
| .......AGAGGTAGTAGGNTGCATAGT............................................................ 30   | 30; |
| .......AGAGGTAGTAGGTTGCATANTT........................................................... 26   | 26; |
| .......AGAGGTAGTAGGTTGC................................................................. 26   | 26; |
| .......AGAGGTAGTAGGTTNCATAGTT........................................................... 18   | 18; |
| ........GAGGTAGTAGGTTGCATAGTTT.......................................................... 16   | 16; |
| .......AGAGGTAGTAGGNTGCATAG............................................................. 14   | 14; |
| .......AGAGGTAGTAGGTTGNATAGTTT.......................................................... 13   | 13; |
| ........GAGGTAGTAGGTTGCATAGT............................................................ 13   | 13; |
| ......AAGAGGTAGTAGGTTGCATAGT............................................................ 11   | 11; |
| ......AAGAGGTAGTAGGTTGCATAGTT........................................................... 10   | 10; |
| .......AGAGGTAGTAGGTNNCATAGTTT.......................................................... 9   | 9; |
| ..............................................................TATACGACCTGCTGCCTTTCT..... 8   | 8; |
| .......AGAGGTAGTAGGTTG.................................................................. 8   | 8; |
| .............................................................CTATACGACCTGCTGCCTTT....... 7   | 7; |
| .......AGAGGTAGTAGGTT................................................................... 6   | 6; |
| .......AGAGGTAGTAGGTNNCATAGT............................................................ 6   | 6; |
| .......AGAGGTAGTAGGTTGCATANTTT.......................................................... 5   | 5; |
| .......AGAGGTAGTAGGTTGNATAGT............................................................ 5   | 5; |
| .........AGGTAGTAGGTTGCATAGTT........................................................... 5   | 5; |
| ..............................................................TATACGACCTGCTGCCTTTC...... 5   | 5; |
| --------------------------------------------------------------------------------------- 57640 |
| .............................................................CTATACGACCTGCTGCCTTTCa..... 17   | 17; |
| .............................................................CTATACGACCTGCTGCCTTTCTa.... 5   | 5; |
| --------------------------------------------------------------------------------------- 22 |
| --------------------------------------------------------------------------------------- 57662 |
| hsa-let-7d-5p(hsa-let-7d) CCTAGGAAGAGGTAGTAGGTTGCATAGTTTTAGGGCAGGGATTTTGCCCACAAGGAGGTAACTATACGACCTGCTGCCTTTCTTAGG (((((((.((((((((((((((.((((((...((((((.....)))))).(.....)..)))))).))))))))))))))))))))) (-42.60) \*\*\*\*\*\*\*AGAGGTAGTAGGTTGCATAGTT\*\*\*\*\*\*\*\*\*\*\*\*\*\*\*\*\*\*\*\*\*\*\*\*\*\*\*\*\*\*\*\*\*\*\*\*\*\*\*\*\*\*\*\*\*\*\*\*\*\*\*\*\*\*\*\*\*\* T  M |
| .......AGAGGTAGTAGGTTGCATAGTT...........................................................\* 40680   | 40680; |
| .......AGAGGTAGTAGGTTGCATAGTTT.......................................................... 8432   | 8432; |
| .......AGAGGTAGTAGGTTGCATAGT............................................................ 5273   | 5273; |
| .......AGAGGTAGTAGGTTGCATAG............................................................. 1659   | 1659; |
| .......AGAGGTAGTAGGTTGCATAGTTTT......................................................... 317   | 317; |
| .......AGAGGTAGTAGGTTGCATA.............................................................. 235   | 235; |
| .......AGAGGTAGTAGGNTGCATAGTT........................................................... 216   | 216; |
| .............................................................CTATACGACCTGCTGCCTTTCT..... 150   | 150; |
| .......AGAGGTAGTAGGTTGCAT............................................................... 89   | 89; |
| ........GAGGTAGTAGGTTGCATAGTT........................................................... 85   | 85; |
| .......AGAGGTAGTAGGTTGNATAGTT........................................................... 81   | 81; |
| .............................................................CTATACGACCTGCTGCCTTTC...... 57   | 57; |
| .......AGAGGTAGTAGGTNNCATAGTT........................................................... 44   | 44; |
| .......AGAGGTAGTAGGNTGCATAGTTT.......................................................... 42   | 42; |
| .......AGAGGTAGTAGGTTGCA................................................................ 39   | 39; |
| .......AGAGGTAGTAGGNTGCATAGT............................................................ 30   | 30; |
| .......AGAGGTAGTAGGTTGCATANTT........................................................... 26   | 26; |
| .......AGAGGTAGTAGGTTGC................................................................. 26   | 26; |
| .......AGAGGTAGTAGGTTNCATAGTT........................................................... 18   | 18; |
| ........GAGGTAGTAGGTTGCATAGTTT.......................................................... 16   | 16; |
| .......AGAGGTAGTAGGNTGCATAG............................................................. 14   | 14; |
| .......AGAGGTAGTAGGTTGNATAGTTT.......................................................... 13   | 13; |
| ........GAGGTAGTAGGTTGCATAGT............................................................ 13   | 13; |
| ......AAGAGGTAGTAGGTTGCATAGT............................................................ 11   | 11; |
| ......AAGAGGTAGTAGGTTGCATAGTT........................................................... 10   | 10; |
| .......AGAGGTAGTAGGTNNCATAGTTT.......................................................... 9   | 9; |
| ..............................................................TATACGACCTGCTGCCTTTCT..... 8   | 8; |
| .......AGAGGTAGTAGGTTG.................................................................. 8   | 8; |
| .............................................................CTATACGACCTGCTGCCTTT....... 7   | 7; |
| .......AGAGGTAGTAGGTT................................................................... 6   | 6; |
| .......AGAGGTAGTAGGTNNCATAGT............................................................ 6   | 6; |
| .......AGAGGTAGTAGGTTGCATANTTT.......................................................... 5   | 5; |
| .......AGAGGTAGTAGGTTGNATAGT............................................................ 5   | 5; |
| .........AGGTAGTAGGTTGCATAGTT........................................................... 5   | 5; |
| ..............................................................TATACGACCTGCTGCCTTTC...... 5   | 5; |
| --------------------------------------------------------------------------------------- 57640 |
| .......AGAGGTAGTAGGTTGCATAGTTa.......................................................... 5125   | 5125; |
| .......AGAGGTAGTAGGTTGCATAGTa........................................................... 1011   | 1011; |
| .......tGAGGTAGTAGGTTGCATAGTT........................................................... 698   | 698; |
| .......AGAGGTAGTAGGTTGCATAGTTg.......................................................... 328   | 328; |
| .......AGAGGTAGTAGGgTGCATAGTT........................................................... 317   | 317; |
| .......AGAGGTAGTAGGTTGCATAGTaa.......................................................... 283   | 283; |
| .......AGAGGgAGTAGGTTGCATAGTT........................................................... 271   | 271; |
| .......AGAGGTAGTAGGTTGCATAGTaT.......................................................... 226   | 226; |
| .......AGAGGTAGTAGGTTGCgTAGTT........................................................... 191   | 191; |
| .......AGAGGTAGTAGGTTGgATAGTT........................................................... 138   | 138; |
| .......AGAGGTAGTAGGTTGaATAGTT........................................................... 137   | 137; |
| .......AGAGGTAGTAGGcTGCATAGTT........................................................... 122   | 122; |
| .......AGAGGTAGTAGGTTGCATAGgT........................................................... 112   | 112; |
| .......AGAGGTAGTAGGTTGCcTAGTT........................................................... 101   | 101; |
| .......AGAGGTAGgAGGTTGCATAGTT........................................................... 100   | 100; |
| .......AGAGGTAGTAGGTTGCATgGTT........................................................... 100   | 100; |
| .......tGAGGTAGTAGGTTGCATAGTTT.......................................................... 98   | 98; |
| .......AGAGGTAGTAGGTTGCATAGa............................................................ 93   | 93; |
| .......AGAGGTAGTAGGaTGCATAGTT........................................................... 77   | 77; |
| .......tGAGGTAGTAGGTTGCATAGT............................................................ 68   | 68; |
| .......AGAGGTAGTAGGTTGCATcGTT........................................................... 67   | 67; |
| .......AGAGGTAGTAGGgTGCATAGTTT.......................................................... 66   | 66; |
| .......tGAGGTAGTAGGTTGCATAGTTa.......................................................... 57   | 57; |
| .......AGAGGcAGTAGGTTGCATAGTT........................................................... 53   | 53; |
| .......AGAGGgAGTAGGTTGCATAGTTT.......................................................... 52   | 52; |
| .......AGAGGTAGTAGGTTGCATAGaa........................................................... 47   | 47; |
| .......AGAGGTAGTAGGTTGCATtGTT........................................................... 45   | 45; |
| .......AGAGGTAGTAGGTTGCATAtTT........................................................... 42   | 42; |
| .......AGAGGTAGTAGGTTGCATAGTg........................................................... 41   | 41; |
| .......AGAGGTAGTAGGgTGCATAGTTa.......................................................... 41   | 41; |
| .......AGAGGTAGTAGGTTGCtTAGTT........................................................... 40   | 40; |
| .......AGAGGTAGTAGGTgGCATAGTT........................................................... 40   | 40; |
| .......AGAGGTAGTAGGTTGCgTAGTTT.......................................................... 39   | 39; |
| .......AGAGGTAGTAGGTTGCATAGcT........................................................... 39   | 39; |
| .......AGAGGTAGTAGGTTGCgTAGTTa.......................................................... 38   | 38; |
| .......AGAGGTAGTAGGTcNCATAGTT........................................................... 35   | 35; |
| .......AGAGGTAGTAGGTTtCATAGTT........................................................... 33   | 33; |
| .......AGAGGTAGTAGGTaNCATAGTT........................................................... 33   | 33; |
| .......AGAGGgAGTAGGTTGCATAGTTa.......................................................... 32   | 32; |
| .......AGAGGTAGTAGGTTGCgTAGT............................................................ 31   | 31; |
| .......AGAGGgAGTAGGTTGCATAGT............................................................ 31   | 31; |
| .......AGAGGTAGTAGGTTGaATAGTTT.......................................................... 30   | 30; |
| .......AGAGGTAGTAGGgTGCATAGT............................................................ 30   | 30; |
| .......AGAGGTAGTAGGNTGCATAGTTa.......................................................... 29   | 29; |
| .......AGAGGTAGTAGGTTGCATAGTTc.......................................................... 28   | 28; |
| .......AGAGGTAGTAGGTTGgATAGTTT.......................................................... 28   | 28; |
| .......AGAGGTAGTAGGTTGCAgAGTT........................................................... 27   | 27; |
| .......AGAGGTAGTAGGcTGCATAGTTT.......................................................... 25   | 25; |
| .......AGAGGTAGTAGGTTGCATgGT............................................................ 24   | 24; |
| .......AGAGGTAGTAGGTTGCATgGTTa.......................................................... 23   | 23; |
| .......AGAGGTAGTAGGTTGCATAGTag.......................................................... 23   | 23; |
| .......AGAGGTAGgAGGTTGCATAGTTa.......................................................... 22   | 22; |
| .......tGAGGTAGTAGGTTGCgTAGTT........................................................... 22   | 22; |
| .......AGAGGTAGTAGGTTGCATAGaT........................................................... 22   | 22; |
| .......AGAGGTAGTAGGTTGCgTtGTT........................................................... 21   | 21; |
| .......AGAGGTAGTAGGTTGgATAGT............................................................ 21   | 21; |
| .......AGAGGTAGTAGGTTGCATgGTTT.......................................................... 21   | 21; |
| .......AGAGGTAGTAGGTTGCAcAGTT........................................................... 21   | 21; |
| .......AGAGGTAGgAGGTTGCATAGTTT.......................................................... 20   | 20; |
| .......AGgGGTAGTAGGTTGCATAGTT........................................................... 20   | 20; |
| .......AGAGGgAGTAGGgTGCATAGTT........................................................... 20   | 20; |
| .......AGAGGTAGTAGGgTGCATAGgT........................................................... 18   | 18; |
| .......tGAGGTAGTAGGNTGCATAGTT........................................................... 18   | 18; |
| .......AGAGGTAGgAGGgTGCATAGTT........................................................... 18   | 18; |
| .......AGAGGgAGTAGGTTGaATAGTT........................................................... 17   | 17; |
| .......AGAGGTAGTAGGTTGCATcGTTT.......................................................... 17   | 17; |
| .......AGAGGgAGgAGGTTGCATAGTT........................................................... 17   | 17; |
| ........GAGGTAGTAGGTTGCATAGTTa.......................................................... 16   | 16; |
| .......AGAGGTAGTAGGTTGCATtGTTT.......................................................... 16   | 16; |
| .......AGAGGTAGTAGGTTGNAcAGTT........................................................... 15   | 15; |
| .......AGAGGTAGTAGGcTGCATAGTTa.......................................................... 15   | 15; |
| .......AGAGGTAGTAGGcTGCATAGT............................................................ 15   | 15; |
| .......AGAGGTAGTAGGTTGaATAGT............................................................ 15   | 15; |
| .......AGAGGTgGTAGGTTGCATAGTT........................................................... 14   | 14; |
| .......AGAGGTAGTAGGTTGCATAGgTT.......................................................... 14   | 14; |
| .......AGAGGTAGTAGGTTGCcTAGT............................................................ 14   | 14; |
| .......AGAGGTAGTAGGTTGCtTAGTTa.......................................................... 14   | 14; |
| .......AGAGGTAGTAGGTTGNAaAGTT........................................................... 14   | 14; |
| .......AGAGGTAGTAGGTTGCATAGTc........................................................... 14   | 14; |
| .......AGcGGTAGTAGGTTGCATAGTT........................................................... 13   | 13; |
| .......AGAGGgAGTAGGaTGCATAGTT........................................................... 13   | 13; |
| .......AGAGGTAGTAGaTTGCATAGTT........................................................... 13   | 13; |
| .......AGAGGTAGcAGGTTGCATAGTT........................................................... 13   | 13; |
| .......AGAGGTAGTAGtTTGCATAGTT........................................................... 13   | 13; |
| .......AGAGGTAGTAGGTTGgATAGTTa.......................................................... 13   | 13; |
| .......AGAGGTAGTAGGTTGCATAGgTa.......................................................... 12   | 12; |
| .......gGAGGTAGTAGGTTGCATAGTT........................................................... 12   | 12; |
| .......AGAGGTAGTAGGTcGCATAGTT........................................................... 12   | 12; |
| .......AGAGGTAGgAGGgTGCATAGTTT.......................................................... 12   | 12; |
| .......AGAGGTAGTAGGTTcCATAGTT........................................................... 12   | 12; |
| .......cGAGGTAGTAGGTTGCATAGTT........................................................... 11   | 11; |
| .......AGAGGTAGTAGGTTGCATAcTT........................................................... 11   | 11; |
| .......AGAGGTAGgAGGTTGCATAGT............................................................ 11   | 11; |
| .......AGAGGTAGTAGGTTGCATAGg............................................................ 11   | 11; |
| .......AGAGGaAGTAGGTTGCATAGTT........................................................... 11   | 11; |
| .......AGAGGTAGTAGGaTGCATAGTTT.......................................................... 11   | 11; |
| .......AGAGGTAGTAGGTTGCATAGaaT.......................................................... 11   | 11; |
| .......AGAGGTAGTAGGTTGCcTAGTTa.......................................................... 10   | 10; |
| .......AGAGGTAGTAGGTTGaATAGTTa.......................................................... 10   | 10; |
| .......AGAGGTAGTAGGTTGCATcGT............................................................ 10   | 10; |
| .......AGAGGTAGTAGGTTGCATAGTga.......................................................... 10   | 10; |
| .......AGAGGTcGTAGGTTGCATAGTT........................................................... 10   | 10; |
| .......AGAGGTAGTAGGTTGNcTAGTT........................................................... 10   | 10; |
| .......tGAGGTAGTAGGTTGCATAGTa........................................................... 10   | 10; |
| .......AGAGGTAGTAGGTTGCcTAGTTT.......................................................... 10   | 10; |
| .......AGAGGTAGTAGGNTGCATAGTa........................................................... 10   | 10; |
| .......AGAGGTAGTAGGcTGgATAGTT........................................................... 10   | 10; |
| .......AGAGGTAGTcGGTTGCATAGTT........................................................... 10   | 10; |
| .......AGAGGTAGTAG-TTGCATAGTT........................................................... 10   | 10; |
| .......AGAGGTAGTAGGTTGCcTcGTT........................................................... 9   | 9; |
| .......AGAGGTAGTAGGgTGCgTAGTT........................................................... 9   | 9; |
| .......AGAGGTAGTAGGTaGCATAGTT........................................................... 8   | 8; |
| .......AGAGGTAGTAGGTTGCATtGT............................................................ 8   | 8; |
| .......AGAGGgAGTAGGTgGCATAGTT........................................................... 8   | 8; |
| .......AGAGGTAGTAGGTTGCATtGTTa.......................................................... 8   | 8; |
| .......AGAGGTAGTAGGTTGCATAaa............................................................ 8   | 8; |
| .......AGAGGcAGTAGGTTGCATAGTTT.......................................................... 8   | 8; |
| .......AGAGGgAGTAGGTTGaATAGTTT.......................................................... 7   | 7; |
| .......AGAGGTAGTAGGaTGaATAGTT........................................................... 7   | 7; |
| .......AGAGGTAGTAGGTTGCATAGc............................................................ 7   | 7; |
| .......AGAGGTAGTgGGTTGCATAGTT........................................................... 7   | 7; |
| .......AGAGGTAGTAGGTTGCtTgGTT........................................................... 7   | 7; |
| .......AGcGGTAGTAGGTTGCATAGTTT.......................................................... 7   | 7; |
| .......AGAGGTAGTAGGTTGNATAGTTa.......................................................... 7   | 7; |
| .......AGAGGTAGTAGGTTGCATAGTgT.......................................................... 7   | 7; |
| ......cAGAGGTAGTAGGTTGCATAGTT........................................................... 7   | 7; |
| .......AGAGGTAGTAGGTTaCATAGTT........................................................... 7   | 7; |
| .......AGAGGTAGTAGGTTGCtTAGTTT.......................................................... 7   | 7; |
| .......AGAGGTAGgAGGTTGCATAGgT........................................................... 7   | 7; |
| .......AGAGGTAGTAGGTTGNAgAGTT........................................................... 6   | 6; |
| .......tGAGGTAGTAGGTTGCATAGTTg.......................................................... 6   | 6; |
| .......AGAGGTAGTAGGTgNCATAGTT........................................................... 6   | 6; |
| .......tGAGGTAGTAGtTTGCATAGTT........................................................... 6   | 6; |
| .......AGAGGTAGTAGGTTGatTAGTT........................................................... 6   | 6; |
| .......AtAGGTAGTAGGTTGCATAGTT........................................................... 6   | 6; |
| .......tGAGGTAGTAGGTTGCATAGgT........................................................... 6   | 6; |
| .......AGAtGTAGTAGGTTGCATAGTT........................................................... 6   | 6; |
| .......AGAGGgAGTAGGcTGCATAGTT........................................................... 6   | 6; |
| .......AGAGGTAGTAGGggGCATAGTT........................................................... 6   | 6; |
| .......AGAGGTAGTAGGcTGCATAGTa........................................................... 6   | 6; |
| .......AGAGGTAtTAGGTTGCATAGTT........................................................... 6   | 6; |
| .......AGAGGgAGTAGGTTGCATAGTa........................................................... 6   | 6; |
| .......AGAGGcAGTAGGgTGCATAGTT........................................................... 6   | 6; |
| .......AGAGGgAGTAGGNTGCATAGTT........................................................... 6   | 6; |
| .......AGAGGcAGTAGGTTGCATAGT............................................................ 6   | 6; |
| .......AGAGGTAGTAGGTNNCATAGTTa.......................................................... 5   | 5; |
| .......AGAGGTAcTAGGTTGCATAGTT........................................................... 5   | 5; |
| .......AGAGGTAGTAGGTTtCATAGTTT.......................................................... 5   | 5; |
| .......AGAGGTAGTAGGTTGNAaAGTTT.......................................................... 5   | 5; |
| .......AGAGGgAGTAGGTTGCATcGTT........................................................... 5   | 5; |
| .......AGAGGTAGTAGGTTGCATANTTa.......................................................... 5   | 5; |
| .......tGAGGTAGTAGGTTGCAcAGTT........................................................... 5   | 5; |
| .......AGAGGTAGTAGGgTGgATAGTT........................................................... 5   | 5; |
| .......AGAGGTAGTAGGTcNCATAGTTa.......................................................... 5   | 5; |
| .......AGAGGTAGaAGGTTGCATAGTT........................................................... 5   | 5; |
| .......tGAGGTAGTAGGTTGCATcGTT........................................................... 5   | 5; |
| .......AGAGGTAGTAGGTTtCATAGTTa.......................................................... 5   | 5; |
| .......tGAGGTAGTAGGgTGCATAGTT........................................................... 5   | 5; |
| .......AGAGGgAGTAGGTTGgATAGTT........................................................... 5   | 5; |
| .......AGAGGTtGTAGGTTGCATAGTT........................................................... 5   | 5; |
| .......AGAGGTAGTAGGTTGNAcAGTTT.......................................................... 5   | 5; |
| .......AGAGGTAGTAGGTTGCATAGcTT.......................................................... 5   | 5; |
| --------------------------------------------------------------------------------------- 11922 |
| --------------------------------------------------------------------------------------- 69562 |
| hsa-let-7e-3p(hsa-let-7e) CCCGGGCTGAGGTAGGAGGTTGTATAGTTGAGGAGGACACCCAAGGAGATCACTATACGGCCTCCTAGCTTTCCCCAGG ((.(((..(((.((((((((((((((((.((...((....)).......)))))))))))))))))).)))..))).)) (-37.80) \*\*\*\*\*\*\*\*\*\*\*\*\*\*\*\*\*\*\*\*\*\*\*\*\*\*\*\*\*\*\*\*\*\*\*\*\*\*\*\*\*\*\*\*\*\*\*\*\*\*\*\*CTATACGGCCTCCTAGCTTTCC\*\*\*\*\* T  M |
| .......TGAGGTAGGAGGTTGTATAGTT................................................... 5690   | 5690; |
| .......TGAGGTAGGAGGTTGTATAGT.................................................... 460   | 460; |
| .......TGAGGTAGGAGGTTGTATAGTTG.................................................. 228   | 228; |
| .......TGAGGTAGGAGGTTGTATAG..................................................... 62   | 62; |
| .......TGAGGTAGGAGGNTGTATAGTT................................................... 26   | 26; |
| .......TGAGGTAGGAGGTTGTATAGTTGA................................................. 23   | 23; |
| .......TGAGGTAGGAGGTNNTATAGTT................................................... 13   | 13; |
| .......TGAGGTAGGAGGTTGTATA...................................................... 12   | 12; |
| ........GAGGTAGGAGGTTGTATAGTT................................................... 8   | 8; |
| .......TGAGGTAGGAGGTTGTAT....................................................... 7   | 7; |
| .......TGAGGTAGGAGGTTGNATAGTT................................................... 5   | 5; |
| ------------------------------------------------------------------------------- 6534 |
| ------------------------------------------------------------------------------- 6534 |
| hsa-let-7e-5p(hsa-let-7e) CCCGGGCTGAGGTAGGAGGTTGTATAGTTGAGGAGGACACCCAAGGAGATCACTATACGGCCTCCTAGCTTTCCCCAGG ((.(((..(((.((((((((((((((((.((...((....)).......)))))))))))))))))).)))..))).)) (-37.80) \*\*\*\*\*\*\*TGAGGTAGGAGGTTGTATAGTT\*\*\*\*\*\*\*\*\*\*\*\*\*\*\*\*\*\*\*\*\*\*\*\*\*\*\*\*\*\*\*\*\*\*\*\*\*\*\*\*\*\*\*\*\*\*\*\*\*\* T  M |
| .......TGAGGTAGGAGGTTGTATAGTT...................................................\* 5690   | 5690; |
| .......TGAGGTAGGAGGTTGTATAGT.................................................... 460   | 460; |
| .......TGAGGTAGGAGGTTGTATAGTTG.................................................. 228   | 228; |
| .......TGAGGTAGGAGGTTGTATAG..................................................... 62   | 62; |
| .......TGAGGTAGGAGGNTGTATAGTT................................................... 26   | 26; |
| .......TGAGGTAGGAGGTTGTATAGTTGA................................................. 23   | 23; |
| .......TGAGGTAGGAGGTNNTATAGTT................................................... 13   | 13; |
| .......TGAGGTAGGAGGTTGTATA...................................................... 12   | 12; |
| ........GAGGTAGGAGGTTGTATAGTT................................................... 8   | 8; |
| .......TGAGGTAGGAGGTTGTAT....................................................... 7   | 7; |
| .......TGAGGTAGGAGGTTGNATAGTT................................................... 5   | 5; |
| ------------------------------------------------------------------------------- 6534 |
| .......TGAGGTAGGAGGTTGTATAGTTa.................................................. 456   | 456; |
| .......TGAGGTAGtAGGTTGTATAGTTG.................................................. 359   | 359; |
| .......TGAGGTAGGAGaTTGTATAGTT................................................... 222   | 222; |
| .......TGAGGTAGGAGGgTGTATAGTT................................................... 167   | 167; |
| .......TGAGGTAGGAGGTTGTATAGTTt.................................................. 130   | 130; |
| .......TGAGGTAGGAGGTTGTATAGgT................................................... 74   | 74; |
| .......TGAGGgAGGAGGTTGTATAGTT................................................... 69   | 69; |
| .......TGAGGTAGGAGGgTGTATAGgT................................................... 64   | 64; |
| .......TGAGGTAGGAGGTTGTgTAGTT................................................... 45   | 45; |
| .......TGAGGTAGGAGGTTGTAcAGTT................................................... 38   | 38; |
| .......TGAGGTAGGAGaTTGTATAGgT................................................... 34   | 34; |
| .......TGAGGTAGGAGGTTGgATAGTT................................................... 31   | 31; |
| .......TGAGGTAGGAGGgTGTATAGT.................................................... 29   | 29; |
| .......TGAGGTAGGAGaTTGTATAGT.................................................... 27   | 27; |
| .......TGAGGTAGGAGGTTGTATAGTa................................................... 25   | 25; |
| .......TGAGGTAGGAGGTTGTATgGTT................................................... 25   | 25; |
| .......TGAGGTAGcAGGTTGTATAGTT................................................... 24   | 24; |
| .......TGAGGTAGGAGaTTGTATAGTTa.................................................. 24   | 24; |
| .......TGAGGTAGGAGGTTGTAgAGTT................................................... 22   | 22; |
| .......TGAGGTAGGAGGTTGTATAGTaa.................................................. 19   | 19; |
| .......TGAGGTAGGAGGTTGTtTAGTT................................................... 18   | 18; |
| .......TGAGGTAGGAGGTTGTATAGg.................................................... 18   | 18; |
| .......TGAGGcAGGAGGTTGTATAGTT................................................... 17   | 17; |
| .......TGAGGTAGGAGtTTGTATAGTT................................................... 17   | 17; |
| .......TGAGGTAGGAGGTTGcATAGTT................................................... 16   | 16; |
| .......TGAGGTAGGAGGgTGTATAGTTa.................................................. 16   | 16; |
| .......TGAGGgAGGAGGTTGTATAGT.................................................... 14   | 14; |
| .......TGAGGcAGGAGGgTGTATAGTT................................................... 13   | 13; |
| .......TGAGGTAGGAGGTTGTcTAGTT................................................... 13   | 13; |
| .......TGgGGTAGGAGGTTGTATAGTT................................................... 12   | 12; |
| .......TGAGGTAGtAGGTTGTATAGTaG.................................................. 12   | 12; |
| .......TGgGGgAGGAGGTTGTATAGTT................................................... 11   | 11; |
| .......TGAGGgAGGAGGgTGTATAGTT................................................... 11   | 11; |
| .......TGAGGTAGGAGGgTGTATAGg.................................................... 10   | 10; |
| .......TGAGGTAGGAGGTTGTATAGa.................................................... 10   | 10; |
| .......TGAGGTAGGAGGTTGTgTtGTT................................................... 10   | 10; |
| .......TGAGGTAGGAGGTTGTATAGgTT.................................................. 9   | 9; |
| .......TGAGGTAGGAGGcTGTATAGTT................................................... 8   | 8; |
| .......TGAGGTAGGgGGTTGTATAGTT................................................... 7   | 7; |
| .......TGcGGTAGGAGGTTGTATAGTT................................................... 7   | 7; |
| .......TGAGGTAGGAGGaTGTATAGTT................................................... 7   | 7; |
| .......TGAGGTAGGAGGTTGTAgAGTg................................................... 7   | 7; |
| .......TGAGGTAGGAGagTGTATAGTT................................................... 7   | 7; |
| .......TGAGGTAGGAGGTgGTATAGTT................................................... 7   | 7; |
| .......TGAGGTAGGAGGTTGTATAGgTa.................................................. 7   | 7; |
| .......TGAGGTAGtAGGTTGTgTAGTTG.................................................. 6   | 6; |
| .......TGAGGcAGGAGGTTGTATAGgT................................................... 6   | 6; |
| .......TGAGGTAGaAGGTTGTATAGTT................................................... 6   | 6; |
| .......TGAGGgAGGAGaTTGTATAGTT................................................... 6   | 6; |
| .......TGAGGgAGGAGGTTGTATAGTTa.................................................. 5   | 5; |
| .......TGAGGTAGGAGGTTGNAaAGTT................................................... 5   | 5; |
| .......TGAGGTAGGAGtTTGTAaAGTT................................................... 5   | 5; |
| .......TGAGGaAGGAGGTTGTATAGTT................................................... 5   | 5; |
| .......TGAGGTAGGAGGgTGTATAGTTt.................................................. 5   | 5; |
| .......TGAGGTAGGAGGTTGTATAGcT................................................... 5   | 5; |
| .......TGAGGTAGGAGGTTGTATAGTaT.................................................. 5   | 5; |
| .......TGAGGTAGGAGGTTGTATtGTT................................................... 5   | 5; |
| .......TGAGGTAGGAGaNTGTATAGTT................................................... 4   | 4; |
| .......TGAGGTAGcAGGTTGTATAGT.................................................... 4   | 4; |
| .......TGAGGTAGcAGGTTGTATAGTTa.................................................. 4   | 4; |
| .......TGAGGcAGGAGaTTGTATAGTT................................................... 4   | 4; |
| .......TGAGGTAGGAGGTTGTgTgGTTG.................................................. 3   | 3; |
| .......TGAGGTAGGAGaTTGgATAGTT................................................... 3   | 3; |
| .......TGAGGTAGGAGaTTGTATAGg.................................................... 3   | 3; |
| ..........GGTAGtAGGTTGTATAGTTGA................................................. 3   | 3; |
| .......TGAGGTAGtAGGgTGTATAGTTG.................................................. 2   | 2; |
| ........GAGGTAGGAGaTTGTATAGTT................................................... 2   | 2; |
| .......TGAGGTAGGAGtTTGTAgAGTT................................................... 2   | 2; |
| .......TGAGGTAGtAGGNTGTATAGTTG.................................................. 2   | 2; |
| .......TGAGGTAGGAGaTTGTcTAGTT................................................... 2   | 2; |
| .......TGcGGTAGGAGaTTGTATAGTT................................................... 2   | 2; |
| .......TGAGGgAGtAGGTTGTATAGTTG.................................................. 2   | 2; |
| .......TGAGGTAGGAGaTTGTATAGTa................................................... 2   | 2; |
| .......TGAGGTAGGAGGTTGTATgGT.................................................... 2   | 2; |
| .......TGAGGgAGaAGGTTGTATAGTT................................................... 2   | 2; |
| .......aGAGGTAGtAGGTTGTATAGTTG.................................................. 1   | 1; |
| .......TGAGGTAGtAGGTTGTAcAGTTG.................................................. 1   | 1; |
| .......TGAGGTAGGAGaTTGcATAGTT................................................... 1   | 1; |
| .......TGAGGTAGGAGaTTGTATgGTT................................................... 1   | 1; |
| .......TGAGGTAGGAGGTTGTgTAGTTt.................................................. 1   | 1; |
| .......TGAGGgAGGAGGTTGTATAGTTt.................................................. 1   | 1; |
| ------------------------------------------------------------------------------- 2286 |
| ------------------------------------------------------------------------------- 8820 |
| hsa-let-7f-1-3p(hsa-let-7f-1) TCAGAGTGAGGTAGTAGATTGTATAGTTGTGGGGTAGTGATTTTACCCTGTTCAGGAGATAACTATACAATCTATTGCCTTCCCTGA ((((.(..(((((((((((((((((((((((((((((.....))))))).........))))))))))))))))))))))..))))) (-43.30) \*\*\*\*\*\*\*\*\*\*\*\*\*\*\*\*\*\*\*\*\*\*\*\*\*\*\*\*\*\*\*\*\*\*\*\*\*\*\*\*\*\*\*\*\*\*\*\*\*\*\*\*\*\*\*\*\*\*\*\*\*\*CTATACAATCTATTGCCTTCCC\*\*\* T  M |
| ......TGAGGTAGTAGATTGTATAGTT............................................................ 146272   | 146272; |
| ......TGAGGTAGTAGATTGTATAGT............................................................. 16867   | 16867; |
| ......TGAGGTAGTAGATTGTATAG.............................................................. 1788   | 1788; |
| ......TGAGGTAGTAGANTGTATAGTT............................................................ 1594   | 1594; |
| ......TGAGGTAGTAGATTGTATAGTTG........................................................... 1219   | 1219; |
| .......GAGGTAGTAGATTGTATAGTT............................................................ 460   | 460; |
| ......TGAGGTAGTAGATTGNATAGTT............................................................ 427   | 427; |
| ......TGAGGTAGTAGATNNTATAGTT............................................................ 406   | 406; |
| ......TGAGGTAGTAGATTGTATA............................................................... 305   | 305; |
| ......TGAGGTAGTAGATTNTATAGTT............................................................ 170   | 170; |
| ......TGAGGTAGTAGATTGTAT................................................................ 167   | 167; |
| ......TGAGGTAGTAGANTGTATAGT............................................................. 156   | 156; |
| ......TGAGGTAGTAGATTGTATANTT............................................................ 148   | 148; |
| .......GAGGTAGTAGATTGTATAGT............................................................. 61   | 61; |
| ......TGAGGTAGTAGATTGTA................................................................. 55   | 55; |
| ......TGAGGTAGTAGATNNTATAGT............................................................. 52   | 52; |
| ......TGAGGTAGTAGA...................................................................... 45   | 45; |
| ......TGAGGTAGTAGATTGTATANNT............................................................ 30   | 30; |
| ......TGAGGTAGTAGATTGNATAGT............................................................. 29   | 29; |
| ......TGAGGTAGTAGATTGTATAGTTGT.......................................................... 29   | 29; |
| ......TGAGGTAGTAGATTGT.................................................................. 28   | 28; |
| ........AGGTAGTAGATTGTATAGTT............................................................ 23   | 23; |
| ......TGAGGTAGTAGATTNTATAGT............................................................. 18   | 18; |
| ........AGGTAGTAGATTGTATAGTTGT.......................................................... 17   | 17; |
| ......TGAGGTAGTAGANTGTATAG.............................................................. 15   | 15; |
| ........AGGTAGTAGATTGTATAGTTG........................................................... 13   | 13; |
| ......TGAGGTAGTAGATTGTATANT............................................................. 13   | 13; |
| .......GAGGTAGTAGATTGTATAG.............................................................. 7   | 7; |
| ......TGAGGTAGTAGATTGTATANN............................................................. 5   | 5; |
| ......TGAGGTAGTAGATNNTATAG.............................................................. 5   | 5; |
| ......TGAGGTAGTAGANTGTATAGTTG........................................................... 5   | 5; |
| .......GAGGTAGTAGATTGTATAGTTG........................................................... 5   | 5; |
| ........AGGTAGTAGATTGTATAGT............................................................. 4   | 4; |
| ......TGAGGTAGTAGATTG................................................................... 3   | 3; |
| ..........GTAGTAGATTGTATAGTT............................................................ 2   | 2; |
| .........GGTAGTAGATTGTATAGTT............................................................ 2   | 2; |
| --------------------------------------------------------------------------------------- 170445 |
| --------------------------------------------------------------------------------------- 170445 |
| hsa-let-7f-2-3p(hsa-let-7f-2) TGTGGGATGAGGTAGTAGATTGTATAGTTTTAGGGTCATACCCCATCTTGGAGATAACTATACAGTCTACTGTCTTTCCCACG .((((((.(((..((((((((((((((((...(((.....))).(((.....)))))))))))))))))))..))))))))). (-40.70) \*\*\*\*\*\*\*\*\*\*\*\*\*\*\*\*\*\*\*\*\*\*\*\*\*\*\*\*\*\*\*\*\*\*\*\*\*\*\*\*\*\*\*\*\*\*\*\*\*\*\*\*\*\*\*\*\*CTATACAGTCTACTGTCTTTCC\*\*\*\* T  M |
| .......TGAGGTAGTAGATTGTATAGTT....................................................... 146272   | 146272; |
| .......TGAGGTAGTAGATTGTATAGT........................................................ 16867   | 16867; |
| .......TGAGGTAGTAGATTGTATAGTTT...................................................... 13580   | 13580; |
| .......TGAGGTAGTAGATTGTATAG......................................................... 1788   | 1788; |
| .......TGAGGTAGTAGANTGTATAGTT....................................................... 1594   | 1594; |
| ........GAGGTAGTAGATTGTATAGTT....................................................... 460   | 460; |
| .......TGAGGTAGTAGATTGNATAGTT....................................................... 427   | 427; |
| .......TGAGGTAGTAGATNNTATAGTT....................................................... 406   | 406; |
| .......TGAGGTAGTAGATTGTATA.......................................................... 305   | 305; |
| .......TGAGGTAGTAGATTGTATAGTTTT..................................................... 189   | 189; |
| .......TGAGGTAGTAGATTNTATAGTT....................................................... 170   | 170; |
| .......TGAGGTAGTAGATTGTAT........................................................... 167   | 167; |
| .......TGAGGTAGTAGANTGTATAGT........................................................ 156   | 156; |
| .......TGAGGTAGTAGATTGTATANTT....................................................... 148   | 148; |
| .......TGAGGTAGTAGANTGTATAGTTT...................................................... 71   | 71; |
| ........GAGGTAGTAGATTGTATAGT........................................................ 61   | 61; |
| ........GAGGTAGTAGATTGTATAGTTT...................................................... 58   | 58; |
| .......TGAGGTAGTAGATTGTA............................................................ 55   | 55; |
| .......TGAGGTAGTAGATNNTATAGT........................................................ 52   | 52; |
| .......TGAGGTAGTAGA................................................................. 45   | 45; |
| ......ATGAGGTAGTAGATTGTATAGTT....................................................... 32   | 32; |
| .......TGAGGTAGTAGATTGTATANNT....................................................... 30   | 30; |
| .......TGAGGTAGTAGATTGNATAGT........................................................ 29   | 29; |
| .......TGAGGTAGTAGATTGT............................................................. 28   | 28; |
| .........AGGTAGTAGATTGTATAGTT....................................................... 23   | 23; |
| .......TGAGGTAGTAGATNNTATAGTTT...................................................... 23   | 23; |
| .......TGAGGTAGTAGATTGNATAGTTT...................................................... 22   | 22; |
| .......TGAGGTAGTAGATTNTATAGT........................................................ 18   | 18; |
| .......TGAGGTAGTAGANTGTATAG......................................................... 15   | 15; |
| .......TGAGGTAGTAGATTGTATANT........................................................ 13   | 13; |
| .......TGAGGTAGTAGATTGTATANTTT...................................................... 8   | 8; |
| ........GAGGTAGTAGATTGTATAG......................................................... 7   | 7; |
| ......ATGAGGTAGTAGATTGTATAGT........................................................ 6   | 6; |
| .......TGAGGTAGTAGATTNTATAGTTT...................................................... 6   | 6; |
| .......TGAGGTAGTAGATNNTATAG......................................................... 5   | 5; |
| .......TGAGGTAGTAGATTGTATANN........................................................ 5   | 5; |
| .........AGGTAGTAGATTGTATAGT........................................................ 4   | 4; |
| .......TGAGGTAGTAGATTG.............................................................. 3   | 3; |
| ...........GTAGTAGATTGTATAGTT....................................................... 2   | 2; |
| ..........GGTAGTAGATTGTATAGTT....................................................... 2   | 2; |
| ----------------------------------------------------------------------------------- 183152 |
| .........................................................CTATACAGTCTACTGTCTTTCt..... 11   | 11; |
| ----------------------------------------------------------------------------------- 11 |
| ----------------------------------------------------------------------------------- 183163 |
| hsa-let-7f-5p(hsa-let-7f-1) TCAGAGTGAGGTAGTAGATTGTATAGTTGTGGGGTAGTGATTTTACCCTGTTCAGGAGATAACTATACAATCTATTGCCTTCCCTGA ((((.(..(((((((((((((((((((((((((((((.....))))))).........))))))))))))))))))))))..))))) (-43.30) \*\*\*\*\*\*TGAGGTAGTAGATTGTATAGTT\*\*\*\*\*\*\*\*\*\*\*\*\*\*\*\*\*\*\*\*\*\*\*\*\*\*\*\*\*\*\*\*\*\*\*\*\*\*\*\*\*\*\*\*\*\*\*\*\*\*\*\*\*\*\*\*\*\*\* T  M |
| ......TGAGGTAGTAGATTGTATAGTT............................................................\* 146272   | 146272; |
| ......TGAGGTAGTAGATTGTATAGT............................................................. 16867   | 16867; |
| ......TGAGGTAGTAGATTGTATAG.............................................................. 1788   | 1788; |
| ......TGAGGTAGTAGANTGTATAGTT............................................................ 1594   | 1594; |
| ......TGAGGTAGTAGATTGTATAGTTG........................................................... 1219   | 1219; |
| .......GAGGTAGTAGATTGTATAGTT............................................................ 460   | 460; |
| ......TGAGGTAGTAGATTGNATAGTT............................................................ 427   | 427; |
| ......TGAGGTAGTAGATNNTATAGTT............................................................ 406   | 406; |
| ......TGAGGTAGTAGATTGTATA............................................................... 305   | 305; |
| ......TGAGGTAGTAGATTNTATAGTT............................................................ 170   | 170; |
| ......TGAGGTAGTAGATTGTAT................................................................ 167   | 167; |
| ......TGAGGTAGTAGANTGTATAGT............................................................. 156   | 156; |
| ......TGAGGTAGTAGATTGTATANTT............................................................ 148   | 148; |
| .......GAGGTAGTAGATTGTATAGT............................................................. 61   | 61; |
| ......TGAGGTAGTAGATTGTA................................................................. 55   | 55; |
| ......TGAGGTAGTAGATNNTATAGT............................................................. 52   | 52; |
| ......TGAGGTAGTAGA...................................................................... 45   | 45; |
| ......TGAGGTAGTAGATTGTATANNT............................................................ 30   | 30; |
| ......TGAGGTAGTAGATTGNATAGT............................................................. 29   | 29; |
| ......TGAGGTAGTAGATTGTATAGTTGT.......................................................... 29   | 29; |
| ......TGAGGTAGTAGATTGT.................................................................. 28   | 28; |
| ........AGGTAGTAGATTGTATAGTT............................................................ 23   | 23; |
| ......TGAGGTAGTAGATTNTATAGT............................................................. 18   | 18; |
| ........AGGTAGTAGATTGTATAGTTGT.......................................................... 17   | 17; |
| ......TGAGGTAGTAGANTGTATAG.............................................................. 15   | 15; |
| ........AGGTAGTAGATTGTATAGTTG........................................................... 13   | 13; |
| ......TGAGGTAGTAGATTGTATANT............................................................. 13   | 13; |
| .......GAGGTAGTAGATTGTATAG.............................................................. 7   | 7; |
| ......TGAGGTAGTAGATTGTATANN............................................................. 5   | 5; |
| ......TGAGGTAGTAGATNNTATAG.............................................................. 5   | 5; |
| ......TGAGGTAGTAGANTGTATAGTTG........................................................... 5   | 5; |
| .......GAGGTAGTAGATTGTATAGTTG........................................................... 5   | 5; |
| ........AGGTAGTAGATTGTATAGT............................................................. 4   | 4; |
| ......TGAGGTAGTAGATTG................................................................... 3   | 3; |
| ..........GTAGTAGATTGTATAGTT............................................................ 2   | 2; |
| .........GGTAGTAGATTGTATAGTT............................................................ 2   | 2; |
| --------------------------------------------------------------------------------------- 170445 |
| ......TGAGGTAGTAGATTGTATAGTTa........................................................... 15072   | 15072; |
| ......TGAGGTAGTAGATTGTATAGTa............................................................ 1276   | 1276; |
| ......TGAGGgAGTAGATTGTATAGTT............................................................ 905   | 905; |
| ......TGAGGTAGTAGtTTGTATAGTT............................................................ 800   | 800; |
| ......TGAGGTAGTAGATTGTATAGTaa........................................................... 613   | 613; |
| ......TGAGGTAGTAGgTTGTATAGTTG........................................................... 359   | 359; |
| ......TGAGGTAGTAGATTGgATAGTT............................................................ 330   | 330; |
| ......TGAGGTAGTAGATTGTgTAGTT............................................................ 281   | 281; |
| ......TGAGGTAGgAGATTGTATAGTT............................................................ 222   | 222; |
| ......TGAGGTAGTAGATTGTATAGgT............................................................ 183   | 183; |
| .....cTGAGGTAGTAGATTGTATAGTT............................................................ 173   | 173; |
| ......TGAGGTAGTAGATTGTATAtTT............................................................ 156   | 156; |
| ......TGAGGTAGTAGATaNTATAGTT............................................................ 127   | 127; |
| ......TGAGGgAGTAGATTGTATAGTTa........................................................... 110   | 110; |
| ......TGAGGcAGTAGATTGTATAGTT............................................................ 110   | 110; |
| ......TGAGGgAGTAGATTGTATAGT............................................................. 98   | 98; |
| ......TGAGGTAGTAGATTGTATtGTT............................................................ 97   | 97; |
| ......TGAGGTAGTAGATTGTATgGTT............................................................ 89   | 89; |
| ......TGAGGTAGTAGATTGTATAGa............................................................. 87   | 87; |
| ......TGAGGTAGTAGATcNTATAGTT............................................................ 81   | 81; |
| ......TGAGGTAGTAGANTGTATAGTTa........................................................... 77   | 77; |
| ......TGgGGTAGTAGATTGTATAGTT............................................................ 72   | 72; |
| ......TGAGGTAGTAGATTGcATAGTT............................................................ 70   | 70; |
| .......GAGGTAGTAGATTGTATAGTTa........................................................... 70   | 70; |
| ......TGAGGTAGTAGATTGTtTAGTT............................................................ 67   | 67; |
| ......cGAGGTAGTAGATTGTATAGTT............................................................ 59   | 59; |
| ......TGAGGTAGTAGATTGTcTAGTT............................................................ 57   | 57; |
| ......TGAGGTAGTAGATTtTATAGTT............................................................ 51   | 51; |
| ......TGcGGTAGTAGATTGTATAGTT............................................................ 49   | 49; |
| ......TGAGGTAGTAGATTGTATcGTT............................................................ 48   | 48; |
| ......TGAGGTAGTAGATTGTAcAGTT............................................................ 48   | 48; |
| ......TGAGGTAGTAGATTGNAaAGTT............................................................ 46   | 46; |
| ......TGAGGTAGTAGATTGTAgAGTT............................................................ 44   | 44; |
| ......TGAGGTAGTAGATTGTATAGTTc........................................................... 43   | 43; |
| ......TGAGGTAGTAGATTGTATAGcT............................................................ 43   | 43; |
| ......TGAGGTAGTAGATTGTATAGTg............................................................ 43   | 43; |
| ......TGAGGTAGTAGtTTGTATAGTTa........................................................... 41   | 41; |
| ......TGAGGTAGcAGATTGTATAGTT............................................................ 40   | 40; |
| ......aGAGGTAGTAGATTGTATAGTT............................................................ 39   | 39; |
| ......TGAGGTAGTAGAgTGTATAGTT............................................................ 38   | 38; |
| ......TGAGGTAGTAGATTGTA--GTT............................................................ 37   | 37; |
| ......TGAGGTAGTAGAcTGTATAGTT............................................................ 36   | 36; |
| ......TGAGGTAGTAGATTGgATAGT............................................................. 36   | 36; |
| ......TGAGGTgGTAGATTGTATAGTT............................................................ 35   | 35; |
| ......TGAGGTAGTAGATTGTgTAGTTa........................................................... 35   | 35; |
| ......TGAGGTAGgAGATTGTATAGgT............................................................ 34   | 34; |
| ......TGAGGTAGTAGtTTGTATAGT............................................................. 34   | 34; |
| ......TGAGGTAGTgGATTGTATAGTT............................................................ 32   | 32; |
| ......TGAGGTAGTAGcTTGTATAGTT............................................................ 32   | 32; |
| ......TGAGGTAGTAGATTGTATAGaa............................................................ 32   | 32; |
| ......TGAGGTcGTAGATTGTATAGTT............................................................ 31   | 31; |
| ......TGAGGTAGTAGATgNTATAGTT............................................................ 31   | 31; |
| ......TGAGGTAGTAGATTGTATAGTc............................................................ 30   | 30; |
| ......TGAGGTAGTAGATTGNAcAGTT............................................................ 29   | 29; |
| ......TGAGGTAGTAGATTGgATAGTTa........................................................... 28   | 28; |
| ......TGAGGTAGTAGATTGTgTAGT............................................................. 27   | 27; |
| ......TGAGGTAGTAGATcGTATAGTT............................................................ 27   | 27; |
| ......TGAGGTAGgAGATTGTATAGT............................................................. 27   | 27; |
| ......TGAGGaAGTAGATTGTATAGTT............................................................ 26   | 26; |
| ......TGAGGTAGgAGATTGTATAGTTa........................................................... 24   | 24; |
| ......TGAGGTAGTAGATTGTATAGTaG........................................................... 24   | 24; |
| ......TGAGGTAGTcGATTGTATAGTT............................................................ 24   | 24; |
| ......TGAGGTAGTAG-TTGTATAGTT............................................................ 23   | 23; |
| .....cTGAGGTAGTAGATTGTATAGT............................................................. 23   | 23; |
| ......TGAGGTAGTAGATTGTATAGgTa........................................................... 22   | 22; |
| ......TGAGGTAGTAGATgGTATAGTT............................................................ 22   | 22; |
| ......TGAGGTAGTAGATTGTATAtT............................................................. 22   | 22; |
| ......TGAG-TAGTAGATTGTATAGTT............................................................ 21   | 21; |
| ......TGAGGTAGTAGATTGTATAGg............................................................. 21   | 21; |
| ......TGAGGTAGTAGATTGNATAGTTa........................................................... 21   | 21; |
| ......TGAGGTAGTAGATTGTATAGaT............................................................ 20   | 20; |
| ......TGAGGTAGTAGATTGaATAGTT............................................................ 19   | 19; |
| ......TGAGGTAGTAGATTGTATtGTTa........................................................... 19   | 19; |
| ......TGAGGTAGTAGATNNTATAGTTa........................................................... 19   | 19; |
| ......TGAGGTAGTAGATTGTATAtTTa........................................................... 18   | 18; |
| ......TGAGGTAGTAGATTGNcTAGTT............................................................ 18   | 18; |
| ......TGAGGTAGTAGATTcTATAGTT............................................................ 17   | 17; |
| ......TGAGGTAGTAGATT--ATAGTT............................................................ 17   | 17; |
| ......TtAGGTAGTAGATTGTATAGTT............................................................ 17   | 17; |
| ......TGAGGTAGTA--TTGTATAGTT............................................................ 17   | 17; |
| ......TGAGGTAGTAGATTaTATAGTT............................................................ 16   | 16; |
| .....tTGAGGTAGTAGATTGTATAGT............................................................. 15   | 15; |
| ......TGAGGTAtTAGATTGTATAGTT............................................................ 15   | 15; |
| ......TGAGGTAGTAGATTGTgTtGTT............................................................ 14   | 14; |
| ......TGAGGTAGTAtATTGTATAGTT............................................................ 14   | 14; |
| ......TGAGGcAGTAGATTGTATAGT............................................................. 14   | 14; |
| ......TGAGGTAGTAGATaNTATAGTTa........................................................... 14   | 14; |
| ......TGAGGTAcTAGATTGTATAGTT............................................................ 13   | 13; |
| ......TGAGGcAGTAGATTGTATAGTTa........................................................... 13   | 13; |
| ......TGAGGTAGTAGATTGTATAcTT............................................................ 13   | 13; |
| ......TGAGGTAGTAGgTTGTATAGTaG........................................................... 12   | 12; |
| ......TGAaGTAGTAGATTGTATAGTT............................................................ 12   | 12; |
| ......TGAGGTAGTAGATTGTtTAGT............................................................. 12   | 12; |
| ......TGAGGTAGaAGATTGTATAGTT............................................................ 11   | 11; |
| ......TGAGGgAGTAGATTGTgTAGTT............................................................ 11   | 11; |
| ......TGAGGgAGTAGATTGgATAGTT............................................................ 11   | 11; |
| ......TGAGGcAGTAGATTGgATAGTT............................................................ 11   | 11; |
| ......TGAGGTAGTAGATTGTATAaTT............................................................ 11   | 11; |
| ......TaAGGTAGTAGATTGTATAGTT............................................................ 11   | 11; |
| ......TGAtGTAGTAGATTGTATAGTT............................................................ 11   | 11; |
| ......TGAGGgAGTAGATTGTATAGcT............................................................ 11   | 11; |
| ......TGAGGTAGTAGATTGNAgAGTT............................................................ 11   | 11; |
| ......TGAGGTAGTAGATaNTATAGT............................................................. 10   | 10; |
| ......TGAGGgAGTAGATTGTATAGgT............................................................ 10   | 10; |
| ......TGAGGTAGTAGATTGTATgGT............................................................. 10   | 10; |
| ......TGAGGTAGTAGATTGTATAGTga........................................................... 10   | 10; |
| ......TGAGGTAGTAGATTGTtTAGTTa........................................................... 10   | 10; |
| ......gGAGGTAGTAGATTGTATAGTT............................................................ 9   | 9; |
| ......TGAGGTAGTAGATTGT-TAGTT............................................................ 9   | 9; |
| .......GAGGgAGTAGATTGTATAGTT............................................................ 9   | 9; |
| ......TGAGGTAGTAGATcNTATAGT............................................................. 8   | 8; |
| ......TGAGGgAGTAGAgTGTATAGTT............................................................ 8   | 8; |
| ......TGgGGTAGTAGATTGTATAGT............................................................. 8   | 8; |
| ......TGAGGTAGTAGtTTGTATAGTTG........................................................... 8   | 8; |
| .......GAGGTAGTAGATTGTATAGTTaa.......................................................... 8   | 8; |
| ......TGAGGTAGTAG-TTGTATAGTTa........................................................... 8   | 8; |
| ......TGAGGTAGTAGATTGTAaAGTT............................................................ 8   | 8; |
| ......TGAGGTAaTAGATTGTATAGTT............................................................ 8   | 8; |
| ......TGAGGTAGTtGATTGTATAGTT............................................................ 8   | 8; |
| ......TGAGGTAGTAGATTGTATgGTTa........................................................... 8   | 8; |
| ......TGAGGTAGTAGATTNTATAGTTa........................................................... 8   | 8; |
| ......TGAGGTAGTAGATTGTcTAGTTa........................................................... 8   | 8; |
| ......TGAGGTAGTAGATTGggTAGTT............................................................ 8   | 8; |
| ......TGAGGgAGTAGATTGaATAGTT............................................................ 8   | 8; |
| ......TGAGGTAGTAGATTGTATtGT............................................................. 8   | 8; |
| ......TGAGGgAGTAGATTGTATAGTa............................................................ 8   | 8; |
| ......TGAGGTAGTAGATTGcATAGT............................................................. 8   | 8; |
| ......TGAGGgAGTAGANTGTATAGTT............................................................ 8   | 8; |
| ......TGAGGTAGTAGAgTGTATAGT............................................................. 8   | 8; |
| ......TGAGGTAGTAGANTGTATAGTa............................................................ 7   | 7; |
| ......TGAGGTAGTAGATT-TATAGTT............................................................ 7   | 7; |
| ......TGAGGTAGgAGAgTGTATAGTT............................................................ 7   | 7; |
| ......TGAGcTAGTAGATTGTATAGTT............................................................ 7   | 7; |
| ......TGgGGTAGTAGATTGTATAGTTa........................................................... 7   | 7; |
| ......TGAGGTAGTAGAcTGTATAGTTa........................................................... 7   | 7; |
| ......TGAGGTAGTAGATTGTATcGT............................................................. 7   | 7; |
| ......TGAGGTAGTAGATTGTATANTTa........................................................... 7   | 7; |
| ......TGAGGgAGTAGATgGTATAGTT............................................................ 7   | 7; |
| ......TGAGGgAGTAGATTGTATAGTTG........................................................... 7   | 7; |
| ......TGAGGTAGTAGgTTGTgTAGTTG........................................................... 6   | 6; |
| ......TGAGGTAGTAGATTGTATcGTTa........................................................... 6   | 6; |
| ......TGAGGTAGTAGATTGTAcAGTTa........................................................... 6   | 6; |
| ......TGAGGgAGTAGtTTGTATAGTT............................................................ 6   | 6; |
| ......TGAGGTAGTAGATcNTATAGTTa........................................................... 6   | 6; |
| ......TGAGGTAGTAGATTGTAgAGT............................................................. 6   | 6; |
| ......TGAGGTAGTAGATTGNcaAGTT............................................................ 6   | 6; |
| ......TGAGGTAGTAGATaGTATAGTT............................................................ 6   | 6; |
| ......TGAGGTAGTAGATTGNAaAGTTa........................................................... 6   | 6; |
| ......TGAGtTAGTAGATTGTATAGTT............................................................ 6   | 6; |
| ......TGAGGcAGTAGATTGTATAGgT............................................................ 6   | 6; |
| ......TGAGGgAGgAGATTGTATAGTT............................................................ 6   | 6; |
| ......cGAGGTAGTAGATTGTATAGT............................................................. 6   | 6; |
| ......TGAGGTAGTAGAT-GTATAGTT............................................................ 6   | 6; |
| ......TGcGGTAGTAGATTGTATAGTTa........................................................... 6   | 6; |
| ......TGAcGTAGTAGATTGTATAGTT............................................................ 6   | 6; |
| ......TGAGGTAGTAGAgTGTATAGTTa........................................................... 5   | 5; |
| ......TGAGGTAGTAGATTGTAgAGTTa........................................................... 5   | 5; |
| ......TGAGGTAGTAGATTGTcTAGT............................................................. 5   | 5; |
| ......TGAGGTAGTAGtTTGTgTAGTT............................................................ 5   | 5; |
| ......TGAGGTAGTAGtTTGTATtGTT............................................................ 5   | 5; |
| ......TGAGGTAGTAGATTGTAcAGT............................................................. 5   | 5; |
| ......TGAGGTAGTAaATTGTATAGTT............................................................ 5   | 5; |
| ......TGAGaTAGTAGATTGTATAGTT............................................................ 5   | 5; |
| ......TGAGGTgGTAGATTGTATAGT............................................................. 5   | 5; |
| ......TGAGGTAGTAGATTGNgTAGTT............................................................ 5   | 5; |
| ......TGAGGTAGTAGANTGaATAGTT............................................................ 5   | 5; |
| ......cGAGGTAGTAGATTGTATAGTTa........................................................... 5   | 5; |
| ......TGcGGTAGTAGATTGTATAGT............................................................. 5   | 5; |
| ......TGAGGgAGTAGcTTGTATAGTT............................................................ 5   | 5; |
| ......TGAGGTAGTAGATcGTATAGT............................................................. 5   | 5; |
| ......TGAGGTAGcAGATTGTATAGTTa........................................................... 5   | 5; |
| .....tTGAGGTAGTAGATTGTATAG.............................................................. 5   | 5; |
| ......TGAGGcAGTAGATTGTATAGTTG........................................................... 5   | 5; |
| ......TGAG-TAGTAGATTGTATAGTTa........................................................... 5   | 5; |
| ......TGgGGTAGTAGATTGTgTAGTT............................................................ 5   | 5; |
| ......TGAGGTAGTAGATgNTATAGT............................................................. 5   | 5; |
| ......TGAGGTAGTAGATTGTAgAGgT............................................................ 4   | 4; |
| .......GAGGTAGTAGATTGTATAGTaa........................................................... 4   | 4; |
| ......TGAGGTtGTAGATTGTATAGTT............................................................ 4   | 4; |
| ......TGAGGTcGTAGATTGTATAGT............................................................. 4   | 4; |
| ......TGAGGTAGTAGATTGTtTAGgT............................................................ 4   | 4; |
| ......TGtGGTAGTAGATTGTATAGTT............................................................ 4   | 4; |
| ......TGAGGaAGTAGATTGTATAGT............................................................. 4   | 4; |
| ......TGAGGTAGTAGATTcTATAGT............................................................. 4   | 4; |
| ......TGAGGTAGTAGAgTGTATAGgT............................................................ 4   | 4; |
| ......TGAGGTAGTAGATTGTATAaT............................................................. 4   | 4; |
| ......TGAGGTAGTAGATTGTATAGTca........................................................... 4   | 4; |
| ......TGAGGTAGTAGATTGgATtGTT............................................................ 4   | 4; |
| ......TGAGGTAGTAGATTGNAcAGT............................................................. 4   | 4; |
| ......TGAGGTAGTAGATcGTATAGTTa........................................................... 4   | 4; |
| ......TGAGGTAGTAGATTGcATAGTTa........................................................... 4   | 4; |
| ......TGAGGTAGTAGATTGNAcAGTTa........................................................... 4   | 4; |
| ......TGAGGTAGgAGANTGTATAGTT............................................................ 4   | 4; |
| ......TGAGGcAGgAGATTGTATAGTT............................................................ 4   | 4; |
| ......aGAGGTAGTAGATTGTATAGT............................................................. 4   | 4; |
| ......TGAGGTAGTAGAaTGTATAGTT............................................................ 4   | 4; |
| ......TGAGGTAGTAGATTGaATAGT............................................................. 3   | 3; |
| ......TGAGGTAGTAGATTGTATtGgT............................................................ 3   | 3; |
| ......TGAGGTAGTAGATTGTATAGaTa........................................................... 3   | 3; |
| ......TGAGGTAGTAGcTTGTATAGT............................................................. 3   | 3; |
| ......TGAGGTAGTAcATTGTATAGTT............................................................ 3   | 3; |
| ......TGAGGTAGgAGATTGgATAGTT............................................................ 3   | 3; |
| ......TGAGGTgGTAGATTGTATAGTTa........................................................... 3   | 3; |
| ......TGAGGTAGTAGANTGcATAGTT............................................................ 3   | 3; |
| ......TcAGGTAGTAGATTGTATAGTT............................................................ 3   | 3; |
| ......TGAGGTAGTAGATTGNATAGTa............................................................ 3   | 3; |
| ......TGAGGTAGTAGATTGgATAGgT............................................................ 3   | 3; |
| ......TGAGGTAtTAGATTGTATAGTTa........................................................... 3   | 3; |
| ......TGAGGTAGTAGATTtTATAGTTa........................................................... 3   | 3; |
| ......TGAGGgAGTAGATTGTtTAGTT............................................................ 3   | 3; |
| ......TGAGGaAGTAGATTGTATAGTTa........................................................... 3   | 3; |
| ......TGgGGTAGTAGATTGTtTAGTT............................................................ 3   | 3; |
| ......TGAGGgAGTAGATTGTATAtTT............................................................ 3   | 3; |
| ......TGAGGTAGTAGATTGTATAGcTa........................................................... 3   | 3; |
| ......TGAGGTAGTAGATTGTgTAGTa............................................................ 3   | 3; |
| ......TGAGGTAGTAGtTTGNATAGTT............................................................ 3   | 3; |
| ......TGAGGgAGTAGATTGTATAGTg............................................................ 3   | 3; |
| ......TGAGGTAGTAGtNTGTATAGTT............................................................ 3   | 3; |
| ......TtAGGTAGTAGATTGTATAGTTa........................................................... 3   | 3; |
| ......TGAGGTAGTAGATTGTtgAGTT............................................................ 3   | 3; |
| ......TGAGGTAGTAGATTGTtTtGTT............................................................ 3   | 3; |
| ......TGAGGgAGTAGATTaTATAGTT............................................................ 3   | 3; |
| ......TGAGGTcGTAGATTGTATAGTTa........................................................... 3   | 3; |
| ......TGAGGTAGTAGANTGgATAGTT............................................................ 3   | 3; |
| ......TGAGGTAGTAGAcTGTATAGT............................................................. 3   | 3; |
| ......aGAGGTAGTAGATTGTATAGTTa........................................................... 3   | 3; |
| ......TtAGGTAGTAGATTGTATAGT............................................................. 3   | 3; |
| ......TGAGGTAGTAGATTGTtTAGTa............................................................ 3   | 3; |
| ......TGAGGTAGgAGATTGTATAGg............................................................. 3   | 3; |
| ......TGAGGTAGTgGATTGTATAGT............................................................. 3   | 3; |
| ......TGAGGTAGTAGtTTGTATAGTa............................................................ 3   | 3; |
| ......TGAGGTAGTAGATTGNAaAGT............................................................. 3   | 3; |
| ......TGAGGgAGTAGATTGTAgAGTT............................................................ 3   | 3; |
| ......TGAGGTAGTAGANaGTATAGTT............................................................ 3   | 3; |
| ......TGAGGTAGTAGATTGNAgAGTTa........................................................... 3   | 3; |
| ......TGAGGTAGTAGAgTGgATAGTT............................................................ 3   | 3; |
| ......TGAGGTAGTAGATTGgATAGTg............................................................ 3   | 3; |
| ......TGAGGTAtTAGATTGTATAGT............................................................. 3   | 3; |
| ......TGAGGTAGcAGATTGTATAGT............................................................. 3   | 3; |
| ......TGAGGTAGTAGATTGTATAGga............................................................ 3   | 3; |
| ......TGAGGTAGTAGATTtTATAGT............................................................. 3   | 3; |
| ......TGAGGTAGTAGATTGTATAaa............................................................. 3   | 3; |
| ......TGAGGTAGTAGANTaTATAGTT............................................................ 3   | 3; |
| ......TGAGGgAGTAGATTGTATgGTT............................................................ 3   | 3; |
| ......TGAGGgAGTAGgTTGTATAGTTG........................................................... 2   | 2; |
| ......TGAGGTAGTAGATTGaATAGTTa........................................................... 2   | 2; |
| ......TGAGGTAGTAGATTGTATAGc............................................................. 2   | 2; |
| ......TGAGGgAGTAGATTtTATAGTT............................................................ 2   | 2; |
| ......TGAGGTAGTAGANTtTATAGTT............................................................ 2   | 2; |
| ......TGAGGTAGTAGtATTGTATAGTT........................................................... 2   | 2; |
| ......TGAGGTAGTAGATTGNcTAGTTa........................................................... 2   | 2; |
| ......TGAGGTAGTAGcTTGTATAGTTa........................................................... 2   | 2; |
| ......TGAGGTAGTAGATTGTgTtGT............................................................. 2   | 2; |
| ......TGAGGTAGgAGATTGTcTAGTT............................................................ 2   | 2; |
| ......TGAGGTAGTAGggTGTATAGTTG........................................................... 2   | 2; |
| ......TGAGGTAGTAGATTGgATAGTa............................................................ 2   | 2; |
| ......TGAGGTAGTAGATaNTgTAGTT............................................................ 2   | 2; |
| ......TGgGGTAGTAGATTGgATAGTT............................................................ 2   | 2; |
| ......TGAGGgAGTAGATTGTATtGTT............................................................ 2   | 2; |
| .......GAGGTAGTAGATTGTATAGTTaT.......................................................... 2   | 2; |
| .....cTGAGGTAGTAGATTGTATAGTa............................................................ 2   | 2; |
| ......TGAGGTAGTgGATTGTATAGTTa........................................................... 2   | 2; |
| ......TGAGGTAGTAGATTGNATAtTT............................................................ 2   | 2; |
| .......GAGGTAGgAGATTGTATAGTT............................................................ 2   | 2; |
| ......TGAtGTAGTAGATTGTATAGT............................................................. 2   | 2; |
| ......TGAGGcAGTgGATTGTATAGTT............................................................ 2   | 2; |
| ......TGAGGTAGTcGATTGTATAGTTa........................................................... 2   | 2; |
| ......gGAGGTAGTAGATTGTATAGTTa........................................................... 2   | 2; |
| ......TGAGGgAGTgGATTGTATAGTT............................................................ 2   | 2; |
| ......TGcGGTAGgAGATTGTATAGTT............................................................ 2   | 2; |
| ......TGgGGTAGTAGATTGTATAtTT............................................................ 2   | 2; |
| ......TGAGGgAGTAGATTGTATcGTT............................................................ 2   | 2; |
| ......TGAGGTAGTAGATaNTATAGTa............................................................ 2   | 2; |
| ......TGAGGTAGTAGATTaTATAGT............................................................. 2   | 2; |
| ......TGAGGTAGTAGgNTGTATAGTTG........................................................... 2   | 2; |
| ......TGAGGTAGTAGATTGNcTAGT............................................................. 2   | 2; |
| ......TGAGGcAGTAGAgTGTATAGTT............................................................ 2   | 2; |
| ......TGAGGTAGgAGATTGTATAGTa............................................................ 2   | 2; |
| ......TGAGGTAGTAGcTTGTgTAGTT............................................................ 1   | 1; |
| ......TGAGGTAGTAGtTTGTATAGgT............................................................ 1   | 1; |
| ......TGAGGTAGTAGtTTGTATAtTT............................................................ 1   | 1; |
| ......TGAGGTAGgAGATTGTATgGTT............................................................ 1   | 1; |
| ......TGAGGTAGTAGtTTGTATcGTT............................................................ 1   | 1; |
| ......aGAGGTAGTAGgTTGTATAGTTG........................................................... 1   | 1; |
| ......TGAGGTAGTAGtTTGgATAGTT............................................................ 1   | 1; |
| ......TGAGGTAGTAGtTaNTATAGTT............................................................ 1   | 1; |
| ......TGAGGTAGTAGtTNNTATAGTT............................................................ 1   | 1; |
| ......TGAGGTAGTAGtTTNTATAGTT............................................................ 1   | 1; |
| .....cTGAGGTAGTAGNTTGTATAGTT............................................................ 1   | 1; |
| ......TGAGGTAGgAGATTGcATAGTT............................................................ 1   | 1; |
| ......TGAGGTAGTAGgTTGTAcAGTTG........................................................... 1   | 1; |
| ......TGAGGTAGTAGATTGTtTgGTT............................................................ 1   | 1; |
| --------------------------------------------------------------------------------------- 24323 |
| --------------------------------------------------------------------------------------- 194768 |
| hsa-let-7f-5p(hsa-let-7f-2) TGTGGGATGAGGTAGTAGATTGTATAGTTTTAGGGTCATACCCCATCTTGGAGATAACTATACAGTCTACTGTCTTTCCCACG .((((((.(((..((((((((((((((((...(((.....))).(((.....)))))))))))))))))))..))))))))). (-40.70) \*\*\*\*\*\*\*TGAGGTAGTAGATTGTATAGTT\*\*\*\*\*\*\*\*\*\*\*\*\*\*\*\*\*\*\*\*\*\*\*\*\*\*\*\*\*\*\*\*\*\*\*\*\*\*\*\*\*\*\*\*\*\*\*\*\*\*\*\*\*\* T  M |
| .......TGAGGTAGTAGATTGTATAGTT.......................................................\* 146272   | 146272; |
| .......TGAGGTAGTAGATTGTATAGT........................................................ 16867   | 16867; |
| .......TGAGGTAGTAGATTGTATAGTTT...................................................... 13580   | 13580; |
| .......TGAGGTAGTAGATTGTATAG......................................................... 1788   | 1788; |
| .......TGAGGTAGTAGANTGTATAGTT....................................................... 1594   | 1594; |
| ........GAGGTAGTAGATTGTATAGTT....................................................... 460   | 460; |
| .......TGAGGTAGTAGATTGNATAGTT....................................................... 427   | 427; |
| .......TGAGGTAGTAGATNNTATAGTT....................................................... 406   | 406; |
| .......TGAGGTAGTAGATTGTATA.......................................................... 305   | 305; |
| .......TGAGGTAGTAGATTGTATAGTTTT..................................................... 189   | 189; |
| .......TGAGGTAGTAGATTNTATAGTT....................................................... 170   | 170; |
| .......TGAGGTAGTAGATTGTAT........................................................... 167   | 167; |
| .......TGAGGTAGTAGANTGTATAGT........................................................ 156   | 156; |
| .......TGAGGTAGTAGATTGTATANTT....................................................... 148   | 148; |
| .......TGAGGTAGTAGANTGTATAGTTT...................................................... 71   | 71; |
| ........GAGGTAGTAGATTGTATAGT........................................................ 61   | 61; |
| ........GAGGTAGTAGATTGTATAGTTT...................................................... 58   | 58; |
| .......TGAGGTAGTAGATTGTA............................................................ 55   | 55; |
| .......TGAGGTAGTAGATNNTATAGT........................................................ 52   | 52; |
| .......TGAGGTAGTAGA................................................................. 45   | 45; |
| ......ATGAGGTAGTAGATTGTATAGTT....................................................... 32   | 32; |
| .......TGAGGTAGTAGATTGTATANNT....................................................... 30   | 30; |
| .......TGAGGTAGTAGATTGNATAGT........................................................ 29   | 29; |
| .......TGAGGTAGTAGATTGT............................................................. 28   | 28; |
| .........AGGTAGTAGATTGTATAGTT....................................................... 23   | 23; |
| .......TGAGGTAGTAGATNNTATAGTTT...................................................... 23   | 23; |
| .......TGAGGTAGTAGATTGNATAGTTT...................................................... 22   | 22; |
| .......TGAGGTAGTAGATTNTATAGT........................................................ 18   | 18; |
| .......TGAGGTAGTAGANTGTATAG......................................................... 15   | 15; |
| .......TGAGGTAGTAGATTGTATANT........................................................ 13   | 13; |
| .......TGAGGTAGTAGATTGTATANTTT...................................................... 8   | 8; |
| ........GAGGTAGTAGATTGTATAG......................................................... 7   | 7; |
| ......ATGAGGTAGTAGATTGTATAGT........................................................ 6   | 6; |
| .......TGAGGTAGTAGATTNTATAGTTT...................................................... 6   | 6; |
| .......TGAGGTAGTAGATNNTATAG......................................................... 5   | 5; |
| .......TGAGGTAGTAGATTGTATANN........................................................ 5   | 5; |
| .........AGGTAGTAGATTGTATAGT........................................................ 4   | 4; |
| .......TGAGGTAGTAGATTG.............................................................. 3   | 3; |
| ...........GTAGTAGATTGTATAGTT....................................................... 2   | 2; |
| ..........GGTAGTAGATTGTATAGTT....................................................... 2   | 2; |
| ----------------------------------------------------------------------------------- 183152 |
| .......TGAGGTAGTAGATTGTATAGTTa...................................................... 15072   | 15072; |
| .......TGAGGTAGTAGATTGTATAGTa....................................................... 1276   | 1276; |
| .......TGAGGgAGTAGATTGTATAGTT....................................................... 905   | 905; |
| .......TGAGGTAGTAGtTTGTATAGTT....................................................... 800   | 800; |
| .......TGAGGTAGTAGATTGTATAGTaa...................................................... 613   | 613; |
| .......TGAGGTAGTAGATTGgATAGTT....................................................... 330   | 330; |
| .......TGAGGTAGTAGATTGTgTAGTT....................................................... 281   | 281; |
| .......TGAGGTAGgAGATTGTATAGTT....................................................... 222   | 222; |
| .......TGAGGTAGTAGATTGTATAGTaT...................................................... 202   | 202; |
| .......TGAGGTAGTAGATTGTATAGgT....................................................... 183   | 183; |
| ......cTGAGGTAGTAGATTGTATAGTT....................................................... 173   | 173; |
| .......TGAGGTAGTAGATTGTATAtTT....................................................... 156   | 156; |
| .......TGAGGTAGTAGATaNTATAGTT....................................................... 127   | 127; |
| .......TGAGGcAGTAGATTGTATAGTT....................................................... 110   | 110; |
| .......TGAGGgAGTAGATTGTATAGTTa...................................................... 110   | 110; |
| .......TGAGGgAGTAGATTGTATAGTTT...................................................... 105   | 105; |
| .......TGAGGgAGTAGATTGTATAGT........................................................ 98   | 98; |
| .......TGAGGTAGTAGATTGTATtGTT....................................................... 97   | 97; |
| .......TGAGGTAGTAGATTGTATgGTT....................................................... 89   | 89; |
| .......TGAGGTAGTAGATTGTATAGa........................................................ 87   | 87; |
| .......TGAGGTAGTAGATcNTATAGTT....................................................... 81   | 81; |
| .......TGAGGTAGTAGANTGTATAGTTa...................................................... 77   | 77; |
| .......TGgGGTAGTAGATTGTATAGTT....................................................... 72   | 72; |
| ........GAGGTAGTAGATTGTATAGTTa...................................................... 70   | 70; |
| .......TGAGGTAGTAGATTGcATAGTT....................................................... 70   | 70; |
| .......TGAGGTAGTAGATTGTtTAGTT....................................................... 67   | 67; |
| .......cGAGGTAGTAGATTGTATAGTT....................................................... 59   | 59; |
| .......TGAGGTAGTAGATTGTcTAGTT....................................................... 57   | 57; |
| .......TGAGGTAGTAGATTtTATAGTT....................................................... 51   | 51; |
| .......TGcGGTAGTAGATTGTATAGTT....................................................... 49   | 49; |
| .......TGAGGTAGTAGATTGTATcGTT....................................................... 48   | 48; |
| .......TGAGGTAGTAGATTGTAcAGTT....................................................... 48   | 48; |
| .......TGAGGTAGTAGATTGNAaAGTT....................................................... 46   | 46; |
| .......TGAGGTAGTAGATTGTAgAGTT....................................................... 44   | 44; |
| .......TGAGGTAGTAGATTGTATAGTTc...................................................... 43   | 43; |
| .......TGAGGTAGTAGATTGTATAGcT....................................................... 43   | 43; |
| .......TGAGGTAGTAGATTGTATAGTg....................................................... 43   | 43; |
| .......TGAGGTAGTAGtTTGTATAGTTa...................................................... 41   | 41; |
| .......TGAGGTAGcAGATTGTATAGTT....................................................... 40   | 40; |
| .......aGAGGTAGTAGATTGTATAGTT....................................................... 39   | 39; |
| .......TGAGGTAGTAGAgTGTATAGTT....................................................... 38   | 38; |
| .......TGAGGTAGTAGATTGTA--GTT....................................................... 37   | 37; |
| .......TGAGGTAGTAGAcTGTATAGTT....................................................... 36   | 36; |
| .......TGAGGTAGTAGATTGgATAGT........................................................ 36   | 36; |
| .......TGAGGTgGTAGATTGTATAGTT....................................................... 35   | 35; |
| .......TGAGGTAGTAGATTGTgTAGTTa...................................................... 35   | 35; |
| .......TGAGGTAGgAGATTGTATAGTTT...................................................... 35   | 35; |
| .......TGAGGTAGTAGtTTGTATAGT........................................................ 34   | 34; |
| .......TGAGGTAGgAGATTGTATAGgT....................................................... 34   | 34; |
| .......TGAGGTAGTAGcTTGTATAGTT....................................................... 32   | 32; |
| .......TGAGGTAGTAGATTGTATAGaa....................................................... 32   | 32; |
| .......TGAGGTAGTgGATTGTATAGTT....................................................... 32   | 32; |
| .......TGAGGTcGTAGATTGTATAGTT....................................................... 31   | 31; |
| .......TGAGGTAGTAGATgNTATAGTT....................................................... 31   | 31; |
| .......TGAGGTAGTAGATTGTATAGTc....................................................... 30   | 30; |
| .......TGAGGTAGTAGATTGNAcAGTT....................................................... 29   | 29; |
| .......TGAGGTAGTAGATTGgATAGTTT...................................................... 28   | 28; |
| .......TGAGGTAGTAGtTTGTATAGTTT...................................................... 28   | 28; |
| .......TGAGGTAGTAGATTGgATAGTTa...................................................... 28   | 28; |
| .......TGAGGTAGTAGATTGTgTAGT........................................................ 27   | 27; |
| .......TGAGGTAGTAGATcGTATAGTT....................................................... 27   | 27; |
| .......TGAGGTAGgAGATTGTATAGT........................................................ 27   | 27; |
| .......TGAGGaAGTAGATTGTATAGTT....................................................... 26   | 26; |
| .......TGAGGTAGTAGATTGTATAGgTT...................................................... 25   | 25; |
| .......TGAGGTAGgAGATTGTATAGTTa...................................................... 24   | 24; |
| .......TGAGGTAGTcGATTGTATAGTT....................................................... 24   | 24; |
| ......cTGAGGTAGTAGATTGTATAGT........................................................ 23   | 23; |
| .......TGAGGTAGTAG-TTGTATAGTT....................................................... 23   | 23; |
| .......TGAGGTAGTAGATTGTATAGgTa...................................................... 22   | 22; |
| .......TGAGGTAGTAGATTGTATAtT........................................................ 22   | 22; |
| .......TGAGGTAGTAGATgGTATAGTT....................................................... 22   | 22; |
| .......TGAGGTAGTAGATTGTATAGg........................................................ 21   | 21; |
| .......TGAG-TAGTAGATTGTATAGTT....................................................... 21   | 21; |
| .......TGAGGTAGTAGATTGNATAGTTa...................................................... 21   | 21; |
| .......TGAGGTAGTAGATTGTATAGaT....................................................... 20   | 20; |
| .......TGAGGTAGTAGATTGTgTAGTTT...................................................... 20   | 20; |
| .......TGAGGTAGTAGATNNTATAGTTa...................................................... 19   | 19; |
| .......TGAGGTAGTAGATTGTATtGTTa...................................................... 19   | 19; |
| .......TGAGGTAGTAGATTGaATAGTT....................................................... 19   | 19; |
| .......TGAGGTAGTAGATTGNcTAGTT....................................................... 18   | 18; |
| .......TGAGGTAGTAGATTGTATAtTTa...................................................... 18   | 18; |
| .......TGAGGTAGTAGATTcTATAGTT....................................................... 17   | 17; |
| .......TGAGGTAGTAGATT--ATAGTT....................................................... 17   | 17; |
| .......TGAGGTAGTA--TTGTATAGTT....................................................... 17   | 17; |
| .......TtAGGTAGTAGATTGTATAGTT....................................................... 17   | 17; |
| .......TGAGGTAGTAGATTaTATAGTT....................................................... 16   | 16; |
| .......TGAGGTAGTAGATTGTATAtTTT...................................................... 16   | 16; |
| ......tTGAGGTAGTAGATTGTATAGT........................................................ 15   | 15; |
| .......TGAGGTAtTAGATTGTATAGTT....................................................... 15   | 15; |
| .......TGAGGcAGTAGATTGTATAGT........................................................ 14   | 14; |
| .......TGAGGTAGTAtATTGTATAGTT....................................................... 14   | 14; |
| .......TGAGGTAGTAGATaNTATAGTTa...................................................... 14   | 14; |
| .......TGAGGTAGTAGATTGTgTtGTT....................................................... 14   | 14; |
| .......TGAGGTAGTAGATTGTATtGTTT...................................................... 14   | 14; |
| .......TGAGGTAcTAGATTGTATAGTT....................................................... 13   | 13; |
| .......TGAGGcAGTAGATTGTATAGTTa...................................................... 13   | 13; |
| .......TGAGGTAGTAGATTGTATAcTT....................................................... 13   | 13; |
| .......TGAGGTAGTAGATTGTATAGTgT...................................................... 13   | 13; |
| .......TGAGGTAGTAGATaNTATAGTTT...................................................... 13   | 13; |
| .......TGAGGcAGTAGATTGTATAGTTT...................................................... 12   | 12; |
| .......TGAGGTAGTAGATTGTtTAGT........................................................ 12   | 12; |
| .......TGAaGTAGTAGATTGTATAGTT....................................................... 12   | 12; |
| .......TGAGGgAGTAGATTGTgTAGTT....................................................... 11   | 11; |
| .......TGAGGcAGTAGATTGgATAGTT....................................................... 11   | 11; |
| .......TGAGGTAGaAGATTGTATAGTT....................................................... 11   | 11; |
| .......TGAtGTAGTAGATTGTATAGTT....................................................... 11   | 11; |
| .......TGAGGgAGTAGATTGgATAGTT....................................................... 11   | 11; |
| .......TGAGGTAGTAGATTGTATAaTT....................................................... 11   | 11; |
| .......TGAGGgAGTAGATTGTATAGcT....................................................... 11   | 11; |
| .......TGAGGTAGTAGATTGNAgAGTT....................................................... 11   | 11; |
| .......TaAGGTAGTAGATTGTATAGTT....................................................... 11   | 11; |
| .......TGAGGgAGTAGATTGTATAGgT....................................................... 10   | 10; |
| .......TGAGGTAGTAGATTGTATAGTga...................................................... 10   | 10; |
| .......TGAGGTAGTAGATTGTATgGT........................................................ 10   | 10; |
| .......TGAGGTAGTAGATaNTATAGT........................................................ 10   | 10; |
| .......TGAGGTAGTAGATTGTtTAGTTa...................................................... 10   | 10; |
| .......TGAGGTAGTAGATTGT-TAGTT....................................................... 9   | 9; |
| ........GAGGgAGTAGATTGTATAGTT....................................................... 9   | 9; |
| .......gGAGGTAGTAGATTGTATAGTT....................................................... 9   | 9; |
| .......TGAGGTAGTAGATTGggTAGTT....................................................... 8   | 8; |
| .......TGAGGTAGTAGATTNTATAGTTa...................................................... 8   | 8; |
| .......TGAGGTAGTAGATTGTATgGTTa...................................................... 8   | 8; |
| .......TGgGGTAGTAGATTGTATAGT........................................................ 8   | 8; |
| .......TGAGGTAGTAGATcNTATAGTTT...................................................... 8   | 8; |
| .......TGAGGgAGTAGANTGTATAGTT....................................................... 8   | 8; |
| .......TGAGGTAaTAGATTGTATAGTT....................................................... 8   | 8; |
| ........GAGGTAGTAGATTGTATAGTTaa..................................................... 8   | 8; |
| .......TGAGGgAGTAGATTGTATAGTa....................................................... 8   | 8; |
| .......TGAGGTAGTtGATTGTATAGTT....................................................... 8   | 8; |
| .......TGcGGTAGTAGATTGTATAGTTT...................................................... 8   | 8; |
| .......TGAGGgAGTAGAgTGTATAGTT....................................................... 8   | 8; |
| .......TGAGGTAGTAG-TTGTATAGTTa...................................................... 8   | 8; |
| .......TGAGGTAGTAGATTGTcTAGTTa...................................................... 8   | 8; |
| .......TGAGGTAGTAGAgTGTATAGT........................................................ 8   | 8; |
| .......TGAGGTAGTAGATTGcATAGT........................................................ 8   | 8; |
| .......TGAGGTAGTAGATTGTATtGT........................................................ 8   | 8; |
| .......TGAGGgAGTAGATTGaATAGTT....................................................... 8   | 8; |
| .......TGAGGTAGTAGATTGTAaAGTT....................................................... 8   | 8; |
| .......TGAGGTAGTAGATcNTATAGT........................................................ 8   | 8; |
| .......TGAGGTAGTAGAcTGTATAGTTa...................................................... 7   | 7; |
| .......cGAGGTAGTAGATTGTATAGTTT...................................................... 7   | 7; |
| .......TGAGGTAGgAGAgTGTATAGTT....................................................... 7   | 7; |
| .......TGAGGTAGTAGATT-TATAGTT....................................................... 7   | 7; |
| .......TGAGGTAGTAGATTGTAgAGTTT...................................................... 7   | 7; |
| .......TGAGGTAGTAGATTGTATcGT........................................................ 7   | 7; |
| .......TGAGGTAGTAGATTGTATANTTa...................................................... 7   | 7; |
| .......TGAtGTAGTAGATTGTATAGTTT...................................................... 7   | 7; |
| .......TGAGGTAGTAGANTGTATAGTa....................................................... 7   | 7; |
| .......TGgGGTAGTAGATTGTATAGTTa...................................................... 7   | 7; |
| .......TGAGGgAGTAGATgGTATAGTT....................................................... 7   | 7; |
| .......TGAGcTAGTAGATTGTATAGTT....................................................... 7   | 7; |
| .......TGcGGTAGTAGATTGTATAGTTa...................................................... 6   | 6; |
| .......TGAGGgAGTAGtTTGTATAGTT....................................................... 6   | 6; |
| .......TGAGGTAGTAGATTGTAcAGTTa...................................................... 6   | 6; |
| .......TGAGtTAGTAGATTGTATAGTT....................................................... 6   | 6; |
| .......TGAGGTAGTAGATTGNAaAGTTa...................................................... 6   | 6; |
| .......TGAGGTAGTAGAT-GTATAGTT....................................................... 6   | 6; |
| .......TGAGGcAGTAGATTGTATAGgT....................................................... 6   | 6; |
| .......TGAGGTAGTAGATTGNAaAGTTT...................................................... 6   | 6; |
| .......TGAGGTAGTAGATTGTATgGTTT...................................................... 6   | 6; |
| .......cGAGGTAGTAGATTGTATAGT........................................................ 6   | 6; |
| .......TGAGGTAGTAGATTGTtTAGTTT...................................................... 6   | 6; |
| .......TGAGGTAGTAGATcNTATAGTTa...................................................... 6   | 6; |
| .......TGAGGTAGTAGATTGNcaAGTT....................................................... 6   | 6; |
| .......TGAGGTAGTAGATTGTATcGTTa...................................................... 6   | 6; |
| .......TGAcGTAGTAGATTGTATAGTT....................................................... 6   | 6; |
| .......TGAGGTAGTAGATaGTATAGTT....................................................... 6   | 6; |
| .......TGAGGTAGTAGATTGTAgAGT........................................................ 6   | 6; |
| .......TGAGGgAGgAGATTGTATAGTT....................................................... 6   | 6; |
| .......TGAGGTAGTAGATgNTATAGT........................................................ 5   | 5; |
| .......TGAGaTAGTAGATTGTATAGTT....................................................... 5   | 5; |
| .......TGAGGTAGTAGATTtTATAGTTT...................................................... 5   | 5; |
| .......TGAGGgAGTAGcTTGTATAGTT....................................................... 5   | 5; |
| .......TGAG-TAGTAGATTGTATAGTTa...................................................... 5   | 5; |
| .......TGAGGTAGTAGtTTGTgTAGTT....................................................... 5   | 5; |
| .......TGAGGTAGTAGATTGTcTAGT........................................................ 5   | 5; |
| .......TGAGGTAGcAGATTGTATAGTTa...................................................... 5   | 5; |
| .......TGAGGTAGTAGATTGTAcAGT........................................................ 5   | 5; |
| .......TGAGGTgGTAGATTGTATAGTTT...................................................... 5   | 5; |
| .......TGgGGTAGTAGATTGTgTAGTT....................................................... 5   | 5; |
| .......cGAGGTAGTAGATTGTATAGTTa...................................................... 5   | 5; |
| .......TGAGGTAGTAaATTGTATAGTT....................................................... 5   | 5; |
| .......TGAGGTAGTAGAgTGTATAGTTa...................................................... 5   | 5; |
| .......TGAGGTgGTAGATTGTATAGT........................................................ 5   | 5; |
| .......TGAGGTAGTAGANTGaATAGTT....................................................... 5   | 5; |
| .......TGcGGTAGTAGATTGTATAGT........................................................ 5   | 5; |
| .......TGAGGTAGTAGATcGTATAGT........................................................ 5   | 5; |
| .....GtTGAGGTAGTAGATTGTATA.......................................................... 5   | 5; |
| .......TGAGGTAGTAGATTGNgTAGTT....................................................... 5   | 5; |
| .......TGAGGTAGTAGtTTGTATtGTT....................................................... 5   | 5; |
| ......tTGAGGTAGTAGATTGTATAG......................................................... 5   | 5; |
| .......TGAGGTAGTAGATTGcATAGTTT...................................................... 5   | 5; |
| .......TGAGGTAGTAGATTGTAgAGTTa...................................................... 5   | 5; |
| .......TGAGGTAGTAGAaTGTATAGTT....................................................... 4   | 4; |
| .......TGAGGTAGTAGATTGcATAGTTa...................................................... 4   | 4; |
| .......TGtGGTAGTAGATTGTATAGTT....................................................... 4   | 4; |
| .......TGAGGTAGTAGATTGNAcAGT........................................................ 4   | 4; |
| .......TGAGGTAGgAGANTGTATAGTT....................................................... 4   | 4; |
| .......TGAGGaAGTAGATTGTATAGT........................................................ 4   | 4; |
| .......TGAGGTAGTAGATTGTATAGTca...................................................... 4   | 4; |
| .......TGAGGTAGTAGAgTGTATAGgT....................................................... 4   | 4; |
| .......TGAGGTAGTAGATTcTATAGT........................................................ 4   | 4; |
| .......TGAGGTAGTAGATTGTtTAGgT....................................................... 4   | 4; |
| .......TGAGGcAGgAGATTGTATAGTT....................................................... 4   | 4; |
| .......TGAGGTAGTAGATTGgATtGTT....................................................... 4   | 4; |
| .......TGAGGTAGTAGATTGNAcAGTTa...................................................... 4   | 4; |
| .......TGAGGTAGTAGATTGTATAaT........................................................ 4   | 4; |
| ........GAGGTAGTAGATTGTATAGTaa...................................................... 4   | 4; |
| .......TGAGGTtGTAGATTGTATAGTT....................................................... 4   | 4; |
| .......aGAGGTAGTAGATTGTATAGT........................................................ 4   | 4; |
| .......TGAGGTAGTAGATTGTAgAGgT....................................................... 4   | 4; |
| .......TGAGGTcGTAGATTGTATAGT........................................................ 4   | 4; |
| .......TGAGGTAGTAGATcGTATAGTTa...................................................... 4   | 4; |
| .......TGAGGgAGTAGATTGTATAGTg....................................................... 3   | 3; |
| .......TGAGGTAGTAGATTtTATAGTTa...................................................... 3   | 3; |
| .......TGAGGTAGTAGATTGTgTtGTTT...................................................... 3   | 3; |
| .......TGAGGgAGTAGATTGTtTAGTT....................................................... 3   | 3; |
| .......TGAGGTAGTAGcTTGTATAGTTT...................................................... 3   | 3; |
| .......TGAGGTAGTAGATTGgATAGTg....................................................... 3   | 3; |
| .......TGAGGTAtTAGATTGTATAGTTa...................................................... 3   | 3; |
| .......TGAGGTAGTAGATTGTATAGga....................................................... 3   | 3; |
| .......TGAGGgAGTAGATTGTATAtTT....................................................... 3   | 3; |
| .......TGAGGTAGTAGATTGNATAGTa....................................................... 3   | 3; |
| .......TGAGGTAtTAGATTGTATAGT........................................................ 3   | 3; |
| .......TGAGGTAGTAcATTGTATAGTT....................................................... 3   | 3; |
| .......TGAGGTAGTAGATTGTATAaa........................................................ 3   | 3; |
| .......TGAGGTAGTAGATTGTgTAGTa....................................................... 3   | 3; |
| .......TGAGGTAGTAGATTGTAcAGTTT...................................................... 3   | 3; |
| .......TGAGGaAGTAGATTGTATAGTTa...................................................... 3   | 3; |
| .......TGAGGTAGTAGATTGTtTtGTT....................................................... 3   | 3; |
| .......TGAGGTAGgAGATTGTATAGg........................................................ 3   | 3; |
| .......TGAGGTAGTAGtTTGTATAGTa....................................................... 3   | 3; |
| .......TGAGGTAGTAGATTGaATAGT........................................................ 3   | 3; |
| .......TGAGGTgGTAGATTGTATAGTTa...................................................... 3   | 3; |
| .......TGAGGTAGTAGATTGNAgAGTTa...................................................... 3   | 3; |
| .......TGAGGTAGTAGANTGcATAGTT....................................................... 3   | 3; |
| .......TGAGGgAGTAGATTaTATAGTT....................................................... 3   | 3; |
| .......TtAGGTAGTAGATTGTATAGTTa...................................................... 3   | 3; |
| .......TGAGGTAGTAGATTGTATtGgT....................................................... 3   | 3; |
| .......aGAGGTAGTAGATTGTATAGTTa...................................................... 3   | 3; |
| .......TGAGGTAGTAGcTTGTATAGT........................................................ 3   | 3; |
| .......TGAGGTAGTAGANTaTATAGTT....................................................... 3   | 3; |
| .......TGAGGTAGTgGATTGTATAGT........................................................ 3   | 3; |
| .......TGAGGTcGTAGATTGTATAGTTa...................................................... 3   | 3; |
| .......TGAGGTAGcAGATTGTATAGT........................................................ 3   | 3; |
| .......TGAGGTAGgAGATTGgATAGTT....................................................... 3   | 3; |
| .......TGAGGTAGTAGATTGTATAGaTa...................................................... 3   | 3; |
| .......TtAGGTAGTAGATTGTATAGT........................................................ 3   | 3; |
| .......TGAGGTAGTAGAgTGgATAGTT....................................................... 3   | 3; |
| .......TGgGGTAGTAGATTGTtTAGTT....................................................... 3   | 3; |
| .......TGAGGTAGTAGANTGgATAGTT....................................................... 3   | 3; |
| .......TGAGGTAGTAGATTGTATAGcTa...................................................... 3   | 3; |
| .......TGAGGTAGTAGtNTGTATAGTT....................................................... 3   | 3; |
| .......TGAGGTAGTAGATTGgATAGgT....................................................... 3   | 3; |
| .......TcAGGTAGTAGATTGTATAGTT....................................................... 3   | 3; |
| .......TGAGGTAGTAGATTGNAaAGT........................................................ 3   | 3; |
| .......TGAGGTAGTAGATTGTtgAGTT....................................................... 3   | 3; |
| .......TGAGGTAGTAGATTGTtTAGTa....................................................... 3   | 3; |
| .......TGAGGgAGTAGATTGTAgAGTT....................................................... 3   | 3; |
| .......TGAGGTAGTAGtTTGNATAGTT....................................................... 3   | 3; |
| .......TGAGGTAGTAGATTtTATAGT........................................................ 3   | 3; |
| .......TGAGGTAGTAGANaGTATAGTT....................................................... 3   | 3; |
| .......TGAGGgAGTAGATTGTATgGTT....................................................... 3   | 3; |
| .......TGAGGTAGTAGAcTGTATAGT........................................................ 3   | 3; |
| .......TGAGGTAGTcGATTGTATAGTTa...................................................... 2   | 2; |
| .......TGAGGTAGTAGATTGNcTAGT........................................................ 2   | 2; |
| .......gGAGGTAGTAGATTGTATAGTTa...................................................... 2   | 2; |
| .......TGAGGTAGTAGATaNTgTAGTT....................................................... 2   | 2; |
| .......TGAGGTAGgAGATTGTcTAGTT....................................................... 2   | 2; |
| .......TGAGGTAGTgGATTGTATAGTTa...................................................... 2   | 2; |
| .......TGcGGTAGgAGATTGTATAGTT....................................................... 2   | 2; |
| .......TGAGGTAGTAGATTGTATAGc........................................................ 2   | 2; |
| .......TGAtGTAGTAGATTGTATAGT........................................................ 2   | 2; |
| .......TGgGGTAGTAGATTGTATAtTT....................................................... 2   | 2; |
| .......TGAGGTAGTAGcTTGTATAGTTa...................................................... 2   | 2; |
| .......TGAGGTAGgAGATTGTATAGTa....................................................... 2   | 2; |
| .......TGAGGcAGTgGATTGTATAGTT....................................................... 2   | 2; |
| .......TGAGGTAGTAGATTGNATAtTT....................................................... 2   | 2; |
| .......TGAGGTAGTAGATTGTgTtGT........................................................ 2   | 2; |
| .......TGAGGgAGTAGATTGTATtGTT....................................................... 2   | 2; |
| .......TGAGGTAGTAGtATTGTATAGTT...................................................... 2   | 2; |
| .......TGAGGTAGTAGATTGNcTAGTTa...................................................... 2   | 2; |
| ........GAGGTAGgAGATTGTATAGTT....................................................... 2   | 2; |
| .......TGAGGTAGTAGATTGaATAGTTa...................................................... 2   | 2; |
| .......TGAGGgAGTgGATTGTATAGTT....................................................... 2   | 2; |
| ......cTGAGGTAGTAGATTGTATAGTa....................................................... 2   | 2; |
| .......TGAGGTAGTAGATTGgATAGTa....................................................... 2   | 2; |
| .......TGAGGTAGTAGANTtTATAGTT....................................................... 2   | 2; |
| .......TGAGGgAGTAGATTtTATAGTT....................................................... 2   | 2; |
| .......TGAGGTAGTAGATaNTATAGTa....................................................... 2   | 2; |
| .......TGAGGTAGTAGATTaTATAGT........................................................ 2   | 2; |
| .......TGAGGgAGTAGATTGTATcGTT....................................................... 2   | 2; |
| ........GAGGTAGTAGATTGTATAGTTaT..................................................... 2   | 2; |
| .......TGgGGTAGTAGATTGgATAGTT....................................................... 2   | 2; |
| .......TGAGGTAGTAGATTGNAcAGTTT...................................................... 2   | 2; |
| .......TGAGGcAGTAGAgTGTATAGTT....................................................... 2   | 2; |
| ......cTGAGGTAGTAGNTTGTATAGTT....................................................... 1   | 1; |
| .......TGAGGTAGTAGtTNNTATAGTT....................................................... 1   | 1; |
| .......TGAGGTAGTAGtTTGgATAGTT....................................................... 1   | 1; |
| .......TGAGGTAGTAGtTTGTATAGgT....................................................... 1   | 1; |
| .......TGAGGTAGTAGATTGTtTgGTT....................................................... 1   | 1; |
| .......TGAGGTAGTAGtTTNTATAGTT....................................................... 1   | 1; |
| .......TGAGGTAGgAGATTGTATgGTT....................................................... 1   | 1; |
| .......TGAGGTAGTAGcTTGTgTAGTT....................................................... 1   | 1; |
| .......TGAGGTAGTAGtTTGTATcGTT....................................................... 1   | 1; |
| .......TGAGGTAGgAGATTGcATAGTT....................................................... 1   | 1; |
| .......TGAGGTAGTAGtTaNTATAGTT....................................................... 1   | 1; |
| .......TGAGGTAGTAGtTTGTATAtTT....................................................... 1   | 1; |
| ----------------------------------------------------------------------------------- 24491 |
| ----------------------------------------------------------------------------------- 207643 |
| hsa-let-7g-3p(hsa-let-7g) AGGCTGAGGTAGTAGTTTGTACAGTTTGAGGGTCTATGATACCACCCGGTACAGGAGATAACTGTACAGGCCACTGCCTTGCCA .(((.((((((((.((((((((((((.....((((.((.((((....))))))..)))))))))))))))).))))))))))). (-40.50) \*\*\*\*\*\*\*\*\*\*\*\*\*\*\*\*\*\*\*\*\*\*\*\*\*\*\*\*\*\*\*\*\*\*\*\*\*\*\*\*\*\*\*\*\*\*\*\*\*\*\*\*\*\*\*\*\*\*\*\*\*CTGTACAGGCCACTGCCTTGC\*\* T  M |
| ....TGAGGTAGTAGTTTGTACAGTT........................................................... 66145   | 66145; |
| ....TGAGGTAGTAGTTTGTACAGT............................................................ 7894   | 7894; |
| ....TGAGGTAGTAGTTTGTACAGTTT.......................................................... 2789   | 2789; |
| ....TGAGGTAGTAGTTTGTACAG............................................................. 438   | 438; |
| ....TGAGGTAGTAGTNTGTACAGTT........................................................... 270   | 270; |
| ...CTGAGGTAGTAGTTTGTACAGTT........................................................... 176   | 176; |
| ....TGAGGTAGTAGTTNNTACAGTT........................................................... 101   | 101; |
| ....TGAGGTAGTAGTTTGNACAGTT........................................................... 95   | 95; |
| .....GAGGTAGTAGTTTGTACAGTT........................................................... 85   | 85; |
| ....TGAGGTAGTAGTTTGTAC............................................................... 67   | 67; |
| ....TGAGGTAGTAGTTTGTACA.............................................................. 42   | 42; |
| ....TGAGGTAGTAGTTTNTACAGTT........................................................... 33   | 33; |
| ....TGAGGTAGTAGTNTGTACAGT............................................................ 32   | 32; |
| ....TGAGGTAGTAGTTTGTA................................................................ 29   | 29; |
| ....TGAGGTAGTAGTTTGTACANTT........................................................... 22   | 22; |
| .....GAGGTAGTAGTTTGTACAGT............................................................ 21   | 21; |
| ...CTGAGGTAGTAGTTTGTACAGT............................................................ 19   | 19; |
| ....TGAGGTAGTAGTTNNTACAGT............................................................ 14   | 14; |
| ....TGAGGTAGTAGTNTGTACAGTTT.......................................................... 14   | 14; |
| ...CTGAGGTAGTAGTTTGTACAGTTT.......................................................... 13   | 13; |
| ....TGAGGTAGTAGTTTGNACAGT............................................................ 8   | 8; |
| ....TGAGGTAGTAGTTTGTACANNT........................................................... 7   | 7; |
| ......AGGTAGTAGTTTGTACAGTT........................................................... 7   | 7; |
| ....TGAGGTAGTAGTTTGT................................................................. 7   | 7; |
| ....TGAGGTAGTAGT..................................................................... 4   | 4; |
| ....TGAGGTAGTAGTTTG.................................................................. 3   | 3; |
| ....TGAGGTAGTAGTTT................................................................... 2   | 2; |
| ------------------------------------------------------------------------------------ 78337 |
| ------------------------------------------------------------------------------------ 78337 |
| hsa-let-7g-5p(hsa-let-7g) AGGCTGAGGTAGTAGTTTGTACAGTTTGAGGGTCTATGATACCACCCGGTACAGGAGATAACTGTACAGGCCACTGCCTTGCCA .(((.((((((((.((((((((((((.....((((.((.((((....))))))..)))))))))))))))).))))))))))). (-40.50) \*\*\*\*TGAGGTAGTAGTTTGTACAGTT\*\*\*\*\*\*\*\*\*\*\*\*\*\*\*\*\*\*\*\*\*\*\*\*\*\*\*\*\*\*\*\*\*\*\*\*\*\*\*\*\*\*\*\*\*\*\*\*\*\*\*\*\*\*\*\*\*\* T  M |
| ....TGAGGTAGTAGTTTGTACAGTT...........................................................\* 66145   | 66145; |
| ....TGAGGTAGTAGTTTGTACAGT............................................................ 7894   | 7894; |
| ....TGAGGTAGTAGTTTGTACAGTTT.......................................................... 2789   | 2789; |
| ....TGAGGTAGTAGTTTGTACAG............................................................. 438   | 438; |
| ....TGAGGTAGTAGTNTGTACAGTT........................................................... 270   | 270; |
| ...CTGAGGTAGTAGTTTGTACAGTT........................................................... 176   | 176; |
| ....TGAGGTAGTAGTTNNTACAGTT........................................................... 101   | 101; |
| ....TGAGGTAGTAGTTTGNACAGTT........................................................... 95   | 95; |
| .....GAGGTAGTAGTTTGTACAGTT........................................................... 85   | 85; |
| ....TGAGGTAGTAGTTTGTAC............................................................... 67   | 67; |
| ....TGAGGTAGTAGTTTGTACA.............................................................. 42   | 42; |
| ....TGAGGTAGTAGTTTNTACAGTT........................................................... 33   | 33; |
| ....TGAGGTAGTAGTNTGTACAGT............................................................ 32   | 32; |
| ....TGAGGTAGTAGTTTGTA................................................................ 29   | 29; |
| ....TGAGGTAGTAGTTTGTACANTT........................................................... 22   | 22; |
| .....GAGGTAGTAGTTTGTACAGT............................................................ 21   | 21; |
| ...CTGAGGTAGTAGTTTGTACAGT............................................................ 19   | 19; |
| ....TGAGGTAGTAGTTNNTACAGT............................................................ 14   | 14; |
| ....TGAGGTAGTAGTNTGTACAGTTT.......................................................... 14   | 14; |
| ...CTGAGGTAGTAGTTTGTACAGTTT.......................................................... 13   | 13; |
| ....TGAGGTAGTAGTTTGNACAGT............................................................ 8   | 8; |
| ....TGAGGTAGTAGTTTGTACANNT........................................................... 7   | 7; |
| ......AGGTAGTAGTTTGTACAGTT........................................................... 7   | 7; |
| ....TGAGGTAGTAGTTTGT................................................................. 7   | 7; |
| ....TGAGGTAGTAGT..................................................................... 4   | 4; |
| ....TGAGGTAGTAGTTTG.................................................................. 3   | 3; |
| ....TGAGGTAGTAGTTT................................................................... 2   | 2; |
| ------------------------------------------------------------------------------------ 78337 |
| ....TGAGGTAGTAGTTTGTACAGTTa.......................................................... 8306   | 8306; |
| ....TGAGGTAGTAGTTTGTAtAGTT........................................................... 800   | 800; |
| ....TGAGGTAGTAGgTTGTACAGTT........................................................... 556   | 556; |
| ....TGAGGTAGTAGTTTGTACAGTa........................................................... 546   | 546; |
| ....TGAGGgAGTAGTTTGTACAGTT........................................................... 456   | 456; |
| ....TGAGGTAGTAGTTTGTACAGTTg.......................................................... 339   | 339; |
| ....TGAGGTAGTAGTTTGTACAGTaa.......................................................... 282   | 282; |
| ....TGAGGTAGTAGTT-GTACAGTT........................................................... 227   | 227; |
| ....TGAGGTAGgAGTTTGTACAGTT........................................................... 138   | 138; |
| ....TGAGGTAGTAGTTTGTAaAGTT........................................................... 129   | 129; |
| ....TGAGGTAGTAGTTTGgACAGTT........................................................... 117   | 117; |
| ....TGAGGTAGTAGTTTGTACgGTT........................................................... 100   | 100; |
| ....TGAGGTAGTAGTTTGTACAGgT........................................................... 96   | 96; |
| ....TGAGGTAGTAGTTTGTAgAGTT........................................................... 88   | 88; |
| ....TGAGGTAGTAGTTTGTACtGTT........................................................... 87   | 87; |
| ....TGAGGTAGTAGTTTGTgCAGTT........................................................... 86   | 86; |
| ....TGAGGTAGTAGTTTGTACAGTaT.......................................................... 76   | 76; |
| ....TGAGGTAGTAGTTTGTACAtTT........................................................... 58   | 58; |
| ....TGAGGgAGTAGTTTGTACAGT............................................................ 58   | 58; |
| ....TGAGGcAGTAGTTTGTACAGTT........................................................... 58   | 58; |
| ....TGAGGTAGTAGTTTtTACAGTT........................................................... 56   | 56; |
| ....TGAGGgAGTAGTTTGTACAGTTa.......................................................... 53   | 53; |
| ....TGAGGTAGTAGTTTGTACcGTT........................................................... 48   | 48; |
| ....TGAGGTAGTAGaTTGTACAGTT........................................................... 48   | 48; |
| ....TGAGGTAGTAGTTaNTACAGTT........................................................... 43   | 43; |
| ....TGAGGTAGTAGgTTGTACAGTTa.......................................................... 42   | 42; |
| ....TGAGGTAGTAGgTTGTACAGT............................................................ 41   | 41; |
| ....TGAGGTAGTAGTTTGTAtAGTTa.......................................................... 41   | 41; |
| ....TGgGGTAGTAGTTTGTACAGTT........................................................... 41   | 41; |
| ....cGAGGTAGTAGTTTGTACAGTT........................................................... 37   | 37; |
| ....TGAGGTAGTAGTNTGTACAGTTa.......................................................... 37   | 37; |
| ....TGAGGTAGTAGTTTGTACAGTg........................................................... 35   | 35; |
| ....TGAGGTAGTAGTTcNTACAGTT........................................................... 35   | 35; |
| ....TGAGGTAGTAGTTTGTAtAGT............................................................ 34   | 34; |
| ....TGAGGTAGTAGTTTGTtCAGTT........................................................... 33   | 33; |
| ....TGAGGTAGTAGTT-GTACAGTTa.......................................................... 31   | 31; |
| ....TGAGGTAGTAGTTTGTACAGcT........................................................... 31   | 31; |
| ....TGAGGTAGTAGaTTGNACAGTT........................................................... 29   | 29; |
| ....TGAGGTAGTAGgTTGNACAGTT........................................................... 28   | 28; |
| ....TGAGGTAGTAGTTTGTAtAGTTT.......................................................... 28   | 28; |
| ....TGAGGgAGTAGTTTGTACAGTTT.......................................................... 26   | 26; |
| ....TGAGGTAGTAGgTTGTACAGTTT.......................................................... 26   | 26; |
| ....TGcGGTAGTAGTTTGTACAGTT........................................................... 26   | 26; |
| ....TGAGGTAGTAGTTTGTACAGa............................................................ 25   | 25; |
| ....TGAGGTAGTgGTTTGTACAGTT........................................................... 24   | 24; |
| ....TGAGGTAGTAGTTTGTcCAGTT........................................................... 24   | 24; |
| ....TGAGGTAGTAGTT-GTACAGT............................................................ 23   | 23; |
| ....aGAGGTAGTAGTTTGTACAGTT........................................................... 23   | 23; |
| ....TGAGGTAGTAGTTTGcACAGTT........................................................... 20   | 20; |
| ....TGAGGTAGgAGTTTGTACAGT............................................................ 18   | 18; |
| ....TGAGGTAGTAGTTTGNACAGTTa.......................................................... 18   | 18; |
| ....TGAGGTAGcAGTTTGTACAGTT........................................................... 17   | 17; |
| ....TGAGGTAGgAGTTTGTACAGgT........................................................... 17   | 17; |
| ....TGAGGTAGgAGTTTGTACAGTTa.......................................................... 17   | 17; |
| ....TGAGGTAtTAGTTTGTACAGTT........................................................... 16   | 16; |
| ....TGAGGTAGTAGTTTGaACAGTT........................................................... 16   | 16; |
| .....GAGGTAGTAGTTTGTACAGTTa.......................................................... 16   | 16; |
| ....TGAGGTAGTAGTTTGNAaAGTT........................................................... 16   | 16; |
| ....TGAGGTAGTAGTTTGgACAGT............................................................ 16   | 16; |
| ....TGAGGTAGTAGgTTGTgCAGTT........................................................... 15   | 15; |
| ....TGAGGTAGTAGTTgGTACAGTT........................................................... 14   | 14; |
| ....TGAGGTAGTAGTTTGTAgAGTTa.......................................................... 14   | 14; |
| ....TGAGGTAGTAGTTTGTACAGTTc.......................................................... 14   | 14; |
| ....TGAGGTAGTAGTTTGTACAGg............................................................ 14   | 14; |
| ....TGAGGTAGTAGTTTGTAaAGT............................................................ 14   | 14; |
| ....TGAGGTAGTAGTTTGTACAGgTa.......................................................... 14   | 14; |
| ....TGAGGTAGTAGTTTGgACAGTTa.......................................................... 13   | 13; |
| ....TGAGGTAGTAGTTTGTACAGTc........................................................... 13   | 13; |
| ....TGAGGTgGTAGTTTGTACAGTT........................................................... 13   | 13; |
| ....TGAGGTAGTAGTTcGTACAGTT........................................................... 13   | 13; |
| ....TGAGtTAGTAGTTTGTACAGTT........................................................... 13   | 13; |
| ....TGAGGTAGTAGTTTcTACAGTT........................................................... 12   | 12; |
| ....TGAGGTAGTAGTTgNTACAGTT........................................................... 12   | 12; |
| ....TGAGGTAGTAGTTTGTAgAGT............................................................ 12   | 12; |
| ....TGAGGTAGTAGTcTGTACAGTT........................................................... 12   | 12; |
| ....TGAGGTAGTAGTTTaTACAGTT........................................................... 12   | 12; |
| ....TGAGGgAGTAGTTTGTACAGcT........................................................... 11   | 11; |
| ....TGAGGTAGTAGTTTGTACtGTTT.......................................................... 11   | 11; |
| ....TGAGGTAGTAGTTTGTgCAGTTT.......................................................... 11   | 11; |
| ....TGAGGTAGTAGTTNNTACAGTTa.......................................................... 10   | 10; |
| ....TGAGGTAGTAGTTTGTACtGT............................................................ 10   | 10; |
| ....TGAGGTAGTAGTTTGNcCAGTT........................................................... 10   | 10; |
| ....TGAGGTAGTAGTTTGTAaAGTTa.......................................................... 10   | 10; |
| ....TGAGGTAGTAGTTTGTACtGTTa.......................................................... 10   | 10; |
| ....TGAGGTAGTAGTT-GTACAGTTT.......................................................... 10   | 10; |
| ....TGAGGgAGTAGgTTGTACAGTT........................................................... 9   | 9; |
| ....TGAGGTAGTAGTTTGTACAGaT........................................................... 9   | 9; |
| ....TGAGGTAGTAGTTTGTACAGTag.......................................................... 9   | 9; |
| ....TGAtGTAGTAGTTTGTACAGTT........................................................... 9   | 9; |
| ....TGAGGTAGTcGTTTGTACAGTT........................................................... 9   | 9; |
| ....TGAGGTAGTAGTTTGTgCAGTTa.......................................................... 9   | 9; |
| ....TGAGGTAGTAGTTTtTACAGT............................................................ 9   | 9; |
| ....gGAGGTAGTAGTTTGTACAGTT........................................................... 9   | 9; |
| ....TGAGaTAGTAGTTTGTACAGTT........................................................... 8   | 8; |
| ....TGAGGgAGTAGTTTGTAaAGTT........................................................... 8   | 8; |
| ....TGAGGTcGTAGTTTGTACAGTT........................................................... 8   | 8; |
| ....TGAGGTAGTAGTTaNTACAGTTa.......................................................... 8   | 8; |
| ....TtAGGTAGTAGTTTGTACAGTT........................................................... 7   | 7; |
| ....TGAGGTAGTAGTTTGTACAGcTa.......................................................... 7   | 7; |
| ....TGAaGTAGTAGTTTGTACAGTT........................................................... 7   | 7; |
| ....TGAGGTAGTAGTTaGTACAGTT........................................................... 7   | 7; |
| ....TGAGGTAGTAGTTTGTACAtT............................................................ 7   | 7; |
| ....TGAGGTAGTAGTTcNTACAGT............................................................ 7   | 7; |
| ....TGAGGcAGTAGTTTGTACAGT............................................................ 7   | 7; |
| ....TGAGGcAGTAGTTTGTACAGTTa.......................................................... 7   | 7; |
| ....TGAGGTAGTAGTTTGTgCAGT............................................................ 7   | 7; |
| ....TGAGGgAGTAGTTTGTACAGTa........................................................... 7   | 7; |
| ....TGAGGTAGTAGTTTGTAaAGTTT.......................................................... 7   | 7; |
| ....TGAGGTAGTAGTTTGcACAGTTa.......................................................... 7   | 7; |
| ....TGAGGTAGTAGTTTGTACgGT............................................................ 7   | 7; |
| ....TGAGGTAGTAGcTTGTACAGTT........................................................... 7   | 7; |
| ....TGAGGaAGTAGTTTGTACAGTT........................................................... 7   | 7; |
| ....cGAGGTAGTAGTTTGTACAGT............................................................ 7   | 7; |
| ....TGAGGTAGTAtTTTGTACAGTT........................................................... 6   | 6; |
| ....TGAGGTAGTAGTTTNTACAGTTa.......................................................... 6   | 6; |
| ....TGAG-TAGTAGTTTGTACAGTT........................................................... 6   | 6; |
| ....TGAGGTAGTAGTTcNTACAGTTa.......................................................... 6   | 6; |
| ....TGAGGTAGTAGTTTGTACAcTT........................................................... 6   | 6; |
| ....TGAGGTAGTAGTTTGTACAGgTT.......................................................... 6   | 6; |
| ....TGAGGTAGTAGTTTGgACAGTTT.......................................................... 6   | 6; |
| ....TGAGGTAGTAGTTTGTACAGaa........................................................... 6   | 6; |
| ....TGAGGTAGTAGTTTGTACANTTa.......................................................... 6   | 6; |
| ....TGAGGgAGTAGTTTGTAtAGTT........................................................... 6   | 6; |
| ....TGAGGTAGTAGaTTGTACAGTTa.......................................................... 6   | 6; |
| ....TGAGGTAGTAGTTTGTACcGT............................................................ 6   | 6; |
| ....TGAGGTAGTAGTTTGTtCAGTTa.......................................................... 5   | 5; |
| ....TGAGGTAGgAGTTTGTAaAGTT........................................................... 5   | 5; |
| ....TGAGGTAGTAGTTTGTACgGTTT.......................................................... 5   | 5; |
| ....TGAGGTAGTAGTgTGTACAGTT........................................................... 5   | 5; |
| ....TGAGGTAGTAGTTTGTgtAGTT........................................................... 5   | 5; |
| ....TGgGGTAGTAGTTTGTACAGTTa.......................................................... 5   | 5; |
| ....TGAGGgAGTAGTTTGgACAGTT........................................................... 5   | 5; |
| ....TGAGGTAGTAGgNTGTACAGTT........................................................... 5   | 5; |
| ....TGtGGTAGTAGTTTGTACAGTT........................................................... 5   | 5; |
| ....TGAGGTAGTAGTTTGTcCAGTTa.......................................................... 5   | 5; |
| ....TGAGGTAGTAGTTTGTACAtTTa.......................................................... 5   | 5; |
| ....TGAGGTAGTAGTTTGTAttGTT........................................................... 5   | 5; |
| ....TGAGGTAGT--TTTGTACAGTT........................................................... 5   | 5; |
| ....TGAGGgAGTAGTTTGTACgGTT........................................................... 5   | 5; |
| ....TGAGGTAGTAGaTTGTACAGT............................................................ 5   | 5; |
| ....TGAGGTAGTAGTTTGTAgAGTTT.......................................................... 5   | 5; |
| ....TGAGGTAGTAGgTTGTACAGTa........................................................... 4   | 4; |
| ....TGAGGTAGTAGaTTGNACAGTTa.......................................................... 4   | 4; |
| ....TGAGGTAGTAGaTTGNACAGT............................................................ 4   | 4; |
| ....TGAGGTAGTAGTTTGTAtAGTa........................................................... 3   | 3; |
| ....TGAGGTAGTAGTNTGTAtAGTT........................................................... 3   | 3; |
| ....TGAGGTAGTAGgTTGNACAGTTa.......................................................... 3   | 3; |
| ....TGAGGTAGTAGaTTGTACAGTTT.......................................................... 3   | 3; |
| ....TGAGGTAGTAGgTTGTgCAGTTT.......................................................... 3   | 3; |
| ....TGAGGTAGTAGTTTGNAtAGTT........................................................... 3   | 3; |
| ....TGAGGTAGTAGgTTGNACAGT............................................................ 3   | 3; |
| ....TGAGGTAGTAGaTTGNACAGTTT.......................................................... 2   | 2; |
| ....TGAGGTAGgAGTTTGTAgAGTT........................................................... 2   | 2; |
| ....TGAGGTAGTAGTTTGTtCtGTTT.......................................................... 2   | 2; |
| ....TGAGGTAGTAGgTTGgACAGTT........................................................... 2   | 2; |
| ....TGAGGTAGTAGgTTGTACtGTT........................................................... 2   | 2; |
| ....TGAGGTAGTAGgTTGNACAGTTT.......................................................... 2   | 2; |
| ....TGAGGTAGTAGTTTGgACtGTT........................................................... 2   | 2; |
| ....TGAGGTAGTAGgTTGTACAGTTg.......................................................... 1   | 1; |
| ....TGAGGTAGTAGTTTGTAtAtTT........................................................... 1   | 1; |
| ....TGAGGTAGTAGTTaNTAtAGTT........................................................... 1   | 1; |
| ....TGAGGTAGTAGTTTGgAtAGTT........................................................... 1   | 1; |
| ....TGAGGTAGTAGgTTGNcCAGTT........................................................... 1   | 1; |
| ....TGAGGTAGTAGTTTNTAtAGTT........................................................... 1   | 1; |
| ....TGAGGTAGTAGgTTGNtCAGTT........................................................... 1   | 1; |
| ....TGAGGTAGTAGTTTGTAtcGTT........................................................... 1   | 1; |
| ....TGAGGTAGTAGgTTGTgCAGT............................................................ 1   | 1; |
| ....TGAGGTAGTAGgTTGNgCAGTT........................................................... 1   | 1; |
| ....TGAGGTAGTAGgTTGTACAGgT........................................................... 1   | 1; |
| ....TGAGGTAGTAGTTTGTgCgGTTT.......................................................... 1   | 1; |
| ....TGAGGTAGTAGTTTGTAtAGgT........................................................... 1   | 1; |
| ....TGAGGTAGTAGTTNNTAtAGTT........................................................... 1   | 1; |
| ------------------------------------------------------------------------------------ 14504 |
| ------------------------------------------------------------------------------------ 92841 |
| hsa-let-7i-3p(hsa-let-7i) CTGGCTGAGGTAGTAGTTTGTGCTGTTGGTCGGGTTGTGACATTGCCCGCTGTGGAGATAACTGCGCAAGCTACTGCCTTGCTA .((((.(((((((((((((((((.(((((.(((((.........)))))))........))).))))))))))))))))))))) (-39.60) \*\*\*\*\*\*\*\*\*\*\*\*\*\*\*\*\*\*\*\*\*\*\*\*\*\*\*\*\*\*\*\*\*\*\*\*\*\*\*\*\*\*\*\*\*\*\*\*\*\*\*\*\*\*\*\*\*\*\*\*\*CTGCGCAAGCTACTGCCTTGCT\* T  M |
| .....TGAGGTAGTAGTTTGTGCTGTT.......................................................... 8609   | 8609; |
| .....TGAGGTAGTAGTTTGTGCTGT........................................................... 3578   | 3578; |
| .....TGAGGTAGTAGTTTGTGCTG............................................................ 680   | 680; |
| .....TGAGGTAGTAGTTTGTGCTGTTG......................................................... 144   | 144; |
| .....TGAGGTAGTAGTTTGTGCT............................................................. 123   | 123; |
| .....TGAGGTAGTAGTNTGTGCTGTT.......................................................... 28   | 28; |
| .....TGAGGTAGTAGTTTGTGC.............................................................. 15   | 15; |
| ......GAGGTAGTAGTTTGTGCTGT........................................................... 14   | 14; |
| .............................................................CTGCGCAAGCTACTGCCTTG.... 12   | 12; |
| ......GAGGTAGTAGTTTGTGCTGTT.......................................................... 12   | 12; |
| .............................................................CTGCGCAAGCTACTGCCTTGCT..\* 12   | 12; |
| .....TGAGGTAGTAGTTNNTGCTGTT.......................................................... 10   | 10; |
| ....CTGAGGTAGTAGTTTGTGCTGTT.......................................................... 10   | 10; |
| .....TGAGGTAGTAGTNTGTGCTGT........................................................... 9   | 9; |
| .....TGAGGTAGTAGTTTGT................................................................ 7   | 7; |
| .....TGAGGTAGTAGTTTGNGCTGTT.......................................................... 7   | 7; |
| .....TGAGGTAGTAGTNTGTGCTG............................................................ 5   | 5; |
| .....TGAGGTAGTAGTTNNTGCTGT........................................................... 5   | 5; |
| .....TGAGGTAGTAGT.................................................................... 4   | 4; |
| .....TGAGGTAGTAGTTTG................................................................. 3   | 3; |
| .....TGAGGTAGTAGTTT.................................................................. 2   | 2; |
| ------------------------------------------------------------------------------------ 13289 |
| .............................................................CTGCGCAAGCTACTGCCTTGCTt. 12   | 12; |
| ------------------------------------------------------------------------------------ 12 |
| ------------------------------------------------------------------------------------ 13301 |
| hsa-let-7i-5p(hsa-let-7i) CTGGCTGAGGTAGTAGTTTGTGCTGTTGGTCGGGTTGTGACATTGCCCGCTGTGGAGATAACTGCGCAAGCTACTGCCTTGCTA .((((.(((((((((((((((((.(((((.(((((.........)))))))........))).))))))))))))))))))))) (-39.60) \*\*\*\*\*TGAGGTAGTAGTTTGTGCTGTT\*\*\*\*\*\*\*\*\*\*\*\*\*\*\*\*\*\*\*\*\*\*\*\*\*\*\*\*\*\*\*\*\*\*\*\*\*\*\*\*\*\*\*\*\*\*\*\*\*\*\*\*\*\*\*\*\* T  M |
| .....TGAGGTAGTAGTTTGTGCTGTT..........................................................\* 8609   | 8609; |
| .....TGAGGTAGTAGTTTGTGCTGT........................................................... 3578   | 3578; |
| .....TGAGGTAGTAGTTTGTGCTG............................................................ 680   | 680; |
| .....TGAGGTAGTAGTTTGTGCTGTTG......................................................... 144   | 144; |
| .....TGAGGTAGTAGTTTGTGCT............................................................. 123   | 123; |
| .....TGAGGTAGTAGTNTGTGCTGTT.......................................................... 28   | 28; |
| .....TGAGGTAGTAGTTTGTGC.............................................................. 15   | 15; |
| ......GAGGTAGTAGTTTGTGCTGT........................................................... 14   | 14; |
| .............................................................CTGCGCAAGCTACTGCCTTGCT.. 12   | 12; |
| .............................................................CTGCGCAAGCTACTGCCTTG.... 12   | 12; |
| ......GAGGTAGTAGTTTGTGCTGTT.......................................................... 12   | 12; |
| .....TGAGGTAGTAGTTNNTGCTGTT.......................................................... 10   | 10; |
| ....CTGAGGTAGTAGTTTGTGCTGTT.......................................................... 10   | 10; |
| .....TGAGGTAGTAGTNTGTGCTGT........................................................... 9   | 9; |
| .....TGAGGTAGTAGTTTGT................................................................ 7   | 7; |
| .....TGAGGTAGTAGTTTGNGCTGTT.......................................................... 7   | 7; |
| .....TGAGGTAGTAGTNTGTGCTG............................................................ 5   | 5; |
| .....TGAGGTAGTAGTTNNTGCTGT........................................................... 5   | 5; |
| .....TGAGGTAGTAGT.................................................................... 4   | 4; |
| .....TGAGGTAGTAGTTTG................................................................. 3   | 3; |
| .....TGAGGTAGTAGTTT.................................................................. 2   | 2; |
| ------------------------------------------------------------------------------------ 13289 |
| .....TGAGGTAGTAGTTTGTGCTGTTa......................................................... 3860   | 3860; |
| .....TGAGGTAGTAGTTTGTGCTGTTt......................................................... 3602   | 3602; |
| .....TGAGGTAGTAGTTTGTGtTGTT.......................................................... 508   | 508; |
| .....TGAGGTAGTAGTTTGTGCTGTaa......................................................... 301   | 301; |
| .....TGAGGTAGTAGgTTGTGCTGTT.......................................................... 284   | 284; |
| .....TGAGGTAGTAGTTTGTGCTGTa.......................................................... 131   | 131; |
| .....TGAGGTAGTAGTTTGTaCTGTT.......................................................... 87   | 87; |
| .....TGAGGTAGTAGTTTGTGCaGTT.......................................................... 86   | 86; |
| .....TGAGGTAGTAGgTTGTGCTGTTa......................................................... 84   | 84; |
[truncated: 1,252,916 more chars]
