# Supplementary material for: The Repertoire and Features of Human Platelet microRNAs
Source: PLoS One. 2012 Dec 4;7(12):e50746. doi: 10.1371/journal.pone.0050746 (PMC3514217; doi:10.1371/journal.pone.0050746)
Supplement: Database S1 — (ZIP) [file pone.0050746.s011.zip › Supporting Platelet microRNA sequence database S1/s4_sequence_s4run45.mir.html]

s4run45 Exact Matches (All miRNAs (both expressed and non-expressed) are listed in this table) 

| mature miRNA | Exact Match to miR (+4) | Exact Match to miR |
| --- | --- | --- |
| hsa-let-7a-2-3p | 0 | 0 |
| hsa-let-7a-3p | 16 | 16 |
| hsa-let-7a-5p | 468491 | 360600 |
| hsa-let-7b-3p | 18 | 0 |
| hsa-let-7b-5p | 87483 | 57882 |
| hsa-let-7c | 5305 | 4004 |
| hsa-let-7d-3p | 227 | 150 |
| hsa-let-7d-5p | 56904 | 40680 |
| hsa-let-7e-3p | 0 | 0 |
| hsa-let-7e-5p | 6490 | 5690 |
| hsa-let-7f-1-3p | 0 | 0 |
| hsa-let-7f-2-3p | 0 | 0 |
| hsa-let-7f-5p | 347326 | 292544 |
| hsa-let-7g-3p | 0 | 0 |
| hsa-let-7g-5p | 77741 | 66145 |
| hsa-let-7i-3p | 24 | 12 |
| hsa-let-7i-5p | 13201 | 8609 |
| hsa-mir-1 | 284 | 270 |
| hsa-mir-100-3p | 0 | 0 |
| hsa-mir-100-5p | 7 | 7 |
| hsa-mir-101-3p | 25864 | 3164 |
| hsa-mir-101-5p | 11 | 11 |
| hsa-mir-103a-2-5p | 9 | 0 |
| hsa-mir-103a-3p | 179782 | 102410 |
| hsa-mir-106b-3p | 159 | 38 |
| hsa-mir-106b-5p | 823 | 355 |
| hsa-mir-107 | 15276 | 2163 |
| hsa-mir-10a-3p | 0 | 0 |
| hsa-mir-10a-5p | 32 | 0 |
| hsa-mir-1250 | 56 | 28 |
| hsa-mir-1255a | 193 | 134 |
| hsa-mir-1255b-2-3p | 0 | 0 |
| hsa-mir-1255b-5p | 12 | 12 |
| hsa-mir-1256 | 16 | 16 |
| hsa-mir-125a-3p | 19 | 6 |
| hsa-mir-125a-5p | 61 | 32 |
| hsa-mir-125b-1-3p | 0 | 0 |
| hsa-mir-125b-2-3p | 0 | 0 |
| hsa-mir-125b-5p | 8 | 8 |
| hsa-mir-126-3p | 63 | 9 |
| hsa-mir-126-5p | 55 | 55 |
| hsa-mir-1260b | 5 | 0 |
| hsa-mir-1262 | 15 | 15 |
| hsa-mir-127-3p | 8 | 8 |
| hsa-mir-127-5p | 0 | 0 |
| hsa-mir-1270 | 30 | 30 |
| hsa-mir-1275 | 12 | 0 |
| hsa-mir-1277-3p | 125 | 116 |
| hsa-mir-1277-5p | 0 | 0 |
| hsa-mir-1278 | 123 | 81 |
| hsa-mir-128 | 2012 | 1116 |
| hsa-mir-1284 | 6 | 0 |
| hsa-mir-1285-3p | 6 | 6 |
| hsa-mir-1285-5p | 0 | 0 |
| hsa-mir-1291 | 5 | 0 |
| hsa-mir-1294 | 32 | 12 |
| hsa-mir-1297 | 2 | 0 |
| hsa-mir-1301 | 53 | 8 |
| hsa-mir-1304-3p | 0 | 0 |
| hsa-mir-1304-5p | 50 | 0 |
| hsa-mir-1306-3p | 37 | 0 |
| hsa-mir-1306-5p | 16 | 6 |
| hsa-mir-1307-3p | 562 | 225 |
| hsa-mir-1307-5p | 141 | 89 |
| hsa-mir-130a-3p | 87 | 76 |
| hsa-mir-130a-5p | 0 | 0 |
| hsa-mir-130b-3p | 128 | 112 |
| hsa-mir-130b-5p | 7 | 0 |
| hsa-mir-132-3p | 8 | 8 |
| hsa-mir-132-5p | 9 | 9 |
| hsa-mir-1322 | 0 | 0 |
| hsa-mir-134 | 12 | 12 |
| hsa-mir-139-3p | 6 | 0 |
| hsa-mir-139-5p | 5 | 0 |
| hsa-mir-140-3p | 110557 | 1233 |
| hsa-mir-140-5p | 29 | 18 |
| hsa-mir-142-3p | 1350 | 179 |
| hsa-mir-142-5p | 15364 | 1648 |
| hsa-mir-143-3p | 7890 | 1448 |
| hsa-mir-143-5p | 149 | 48 |
| hsa-mir-144-3p | 0 | 0 |
| hsa-mir-144-5p | 62 | 17 |
| hsa-mir-145-3p | 84 | 0 |
| hsa-mir-145-5p | 308 | 106 |
| hsa-mir-146a-3p | 0 | 0 |
| hsa-mir-146a-5p | 41 | 26 |
| hsa-mir-146b-3p | 0 | 0 |
| hsa-mir-146b-5p | 427 | 133 |
| hsa-mir-148a-3p | 1654 | 1190 |
| hsa-mir-148a-5p | 18 | 0 |
| hsa-mir-148b-3p | 517 | 353 |
| hsa-mir-148b-5p | 33 | 0 |
| hsa-mir-150-3p | 87 | 0 |
| hsa-mir-150-5p | 21 | 15 |
| hsa-mir-151a-3p | 86 | 24 |
| hsa-mir-151a-5p | 20 | 10 |
| hsa-mir-151b | 20 | 0 |
| hsa-mir-152 | 125 | 97 |
| hsa-mir-1537 | 54 | 42 |
| hsa-mir-155-3p | 0 | 0 |
| hsa-mir-155-5p | 36 | 16 |
| hsa-mir-15a-3p | 0 | 0 |
| hsa-mir-15a-5p | 875 | 456 |
| hsa-mir-15b-3p | 6 | 6 |
| hsa-mir-15b-5p | 1635 | 771 |
| hsa-mir-16-1-3p | 10 | 5 |
| hsa-mir-16-2-3p | 44 | 0 |
| hsa-mir-16-5p | 12302 | 9756 |
| hsa-mir-17-3p | 173 | 121 |
| hsa-mir-17-5p | 463 | 260 |
| hsa-mir-181a-2-3p | 119 | 24 |
| hsa-mir-181a-3p | 234 | 35 |
| hsa-mir-181a-5p | 11584 | 2742 |
| hsa-mir-181b-3p | 0 | 0 |
| hsa-mir-181b-5p | 784 | 34 |
| hsa-mir-181c-3p | 76 | 0 |
| hsa-mir-181c-5p | 395 | 43 |
| hsa-mir-181d | 622 | 103 |
| hsa-mir-185-3p | 141 | 0 |
| hsa-mir-185-5p | 15233 | 12331 |
| hsa-mir-186-3p | 0 | 0 |
| hsa-mir-186-5p | 1897 | 1005 |
| hsa-mir-18a-3p | 0 | 0 |
| hsa-mir-18a-5p | 37 | 0 |
| hsa-mir-191-3p | 7 | 0 |
| hsa-mir-191-5p | 57647 | 20478 |
| hsa-mir-1910 | 0 | 0 |
| hsa-mir-192-3p | 0 | 0 |
| hsa-mir-192-5p | 17386 | 7895 |
| hsa-mir-193a-3p | 42 | 32 |
| hsa-mir-193a-5p | 284 | 257 |
| hsa-mir-194-3p | 0 | 0 |
| hsa-mir-194-5p | 175 | 12 |
| hsa-mir-196b-3p | 0 | 0 |
| hsa-mir-196b-5p | 109 | 25 |
| hsa-mir-197-3p | 98 | 52 |
| hsa-mir-197-5p | 9 | 0 |
| hsa-mir-199a-3p | 23229 | 8268 |
| hsa-mir-199a-5p | 0 | 0 |
| hsa-mir-199b-3p | 23229 | 8268 |
| hsa-mir-199b-5p | 323 | 42 |
| hsa-mir-19b-1-5p | 0 | 0 |
| hsa-mir-19b-2-5p | 0 | 0 |
| hsa-mir-19b-3p | 110 | 42 |
| hsa-mir-200b-3p | 5 | 0 |
| hsa-mir-200b-5p | 0 | 0 |
| hsa-mir-200c-3p | 9 | 0 |
| hsa-mir-200c-5p | 0 | 0 |
| hsa-mir-203 | 32 | 0 |
| hsa-mir-20a-3p | 8 | 0 |
| hsa-mir-20a-5p | 118 | 68 |
| hsa-mir-21-3p | 236 | 29 |
| hsa-mir-21-5p | 41169 | 13090 |
| hsa-mir-210 | 16 | 16 |
| hsa-mir-2110 | 131 | 16 |
| hsa-mir-2115-3p | 193 | 54 |
| hsa-mir-2115-5p | 9 | 9 |
| hsa-mir-212-3p | 0 | 0 |
| hsa-mir-212-5p | 21 | 21 |
| hsa-mir-215 | 5 | 0 |
| hsa-mir-216b | 9 | 9 |
| hsa-mir-22-3p | 954 | 607 |
| hsa-mir-22-5p | 216 | 111 |
| hsa-mir-221-3p | 17075 | 2512 |
| hsa-mir-221-5p | 3382 | 2524 |
| hsa-mir-222-3p | 1740 | 286 |
| hsa-mir-222-5p | 0 | 0 |
| hsa-mir-223-3p | 51251 | 14700 |
| hsa-mir-223-5p | 0 | 0 |
| hsa-mir-224-3p | 0 | 0 |
| hsa-mir-224-5p | 8 | 0 |
| hsa-mir-2355-3p | 116 | 82 |
| hsa-mir-2355-5p | 177 | 28 |
| hsa-mir-23a-3p | 34022 | 3308 |
| hsa-mir-23a-5p | 75 | 60 |
| hsa-mir-23b-3p | 810 | 93 |
| hsa-mir-23b-5p | 5 | 0 |
| hsa-mir-24-1-5p | 0 | 0 |
| hsa-mir-24-2-5p | 215 | 0 |
| hsa-mir-24-3p | 6637 | 4692 |
| hsa-mir-25-3p | 89444 | 82205 |
| hsa-mir-25-5p | 721 | 109 |
| hsa-mir-26a-1-3p | 0 | 0 |
| hsa-mir-26a-2-3p | 0 | 0 |
| hsa-mir-26a-5p | 17048 | 15878 |
| hsa-mir-26b-3p | 0 | 0 |
| hsa-mir-26b-5p | 32610 | 1956 |
| hsa-mir-27a-3p | 7886 | 250 |
| hsa-mir-27a-5p | 99 | 91 |
| hsa-mir-27b-3p | 562 | 106 |
| hsa-mir-27b-5p | 0 | 0 |
| hsa-mir-28-3p | 260 | 235 |
| hsa-mir-28-5p | 398 | 242 |
| hsa-mir-29a-3p | 25747 | 21987 |
| hsa-mir-29a-5p | 10 | 5 |
| hsa-mir-29b-1-5p | 9 | 9 |
| hsa-mir-29b-2-5p | 13 | 6 |
| hsa-mir-29b-3p | 528 | 356 |
| hsa-mir-29c-3p | 1368 | 1280 |
| hsa-mir-29c-5p | 9 | 0 |
| hsa-mir-301a-3p | 8 | 0 |
| hsa-mir-301a-5p | 88 | 67 |
| hsa-mir-30a-3p | 16 | 0 |
| hsa-mir-30a-5p | 28 | 12 |
| hsa-mir-30b-3p | 60 | 0 |
| hsa-mir-30b-5p | 54 | 54 |
| hsa-mir-30c-1-3p | 216 | 9 |
| hsa-mir-30c-2-3p | 0 | 0 |
| hsa-mir-30c-5p | 576 | 96 |
| hsa-mir-30d-3p | 0 | 0 |
| hsa-mir-30d-5p | 2014 | 570 |
| hsa-mir-30e-3p | 7002 | 3858 |
| hsa-mir-30e-5p | 3112 | 196 |
| hsa-mir-3130-3p | 22 | 22 |
| hsa-mir-3130-5p | 0 | 0 |
| hsa-mir-3136-3p | 0 | 0 |
| hsa-mir-3136-5p | 8 | 0 |
| hsa-mir-3138 | 7 | 0 |
| hsa-mir-3140-3p | 6 | 0 |
| hsa-mir-3140-5p | 0 | 0 |
| hsa-mir-3143 | 35 | 0 |
| hsa-mir-3154 | 8 | 8 |
| hsa-mir-3179 | 15 | 15 |
| hsa-mir-32-3p | 14 | 0 |
| hsa-mir-32-5p | 33 | 8 |
| hsa-mir-3202 | 6 | 0 |
| hsa-mir-320a | 16779 | 10718 |
| hsa-mir-320b | 139 | 70 |
| hsa-mir-320c | 10 | 6 |
| hsa-mir-320d | 4 | 4 |
| hsa-mir-323a-3p | 5 | 5 |
| hsa-mir-323a-5p | 0 | 0 |
| hsa-mir-323b-3p | 7 | 0 |
| hsa-mir-323b-5p | 0 | 0 |
| hsa-mir-324-3p | 0 | 0 |
| hsa-mir-324-5p | 47 | 22 |
| hsa-mir-328 | 25 | 20 |
| hsa-mir-330-3p | 2329 | 1144 |
| hsa-mir-330-5p | 0 | 0 |
| hsa-mir-331-3p | 90 | 58 |
| hsa-mir-331-5p | 0 | 0 |
| hsa-mir-335-3p | 0 | 0 |
| hsa-mir-335-5p | 65 | 17 |
| hsa-mir-338-3p | 292 | 11 |
| hsa-mir-338-5p | 204 | 0 |
| hsa-mir-339-3p | 91 | 27 |
| hsa-mir-339-5p | 9 | 0 |
| hsa-mir-33a-3p | 0 | 0 |
| hsa-mir-33a-5p | 440 | 364 |
| hsa-mir-33b-3p | 0 | 0 |
| hsa-mir-33b-5p | 24 | 5 |
| hsa-mir-340-3p | 0 | 0 |
| hsa-mir-340-5p | 7379 | 5597 |
| hsa-mir-342-3p | 161 | 77 |
| hsa-mir-342-5p | 39 | 14 |
| hsa-mir-345-3p | 0 | 0 |
| hsa-mir-345-5p | 61 | 19 |
| hsa-mir-34c-3p | 0 | 0 |
| hsa-mir-34c-5p | 237 | 109 |
| hsa-mir-3605-3p | 0 | 0 |
| hsa-mir-3605-5p | 106 | 10 |
| hsa-mir-361-3p | 5 | 5 |
| hsa-mir-361-5p | 63 | 36 |
| hsa-mir-3614-3p | 75 | 17 |
| hsa-mir-3614-5p | 168 | 6 |
| hsa-mir-3615 | 50 | 0 |
| hsa-mir-362-3p | 11 | 11 |
| hsa-mir-362-5p | 27 | 12 |
| hsa-mir-363-3p | 145 | 40 |
| hsa-mir-363-5p | 0 | 0 |
| hsa-mir-365a-3p | 54 | 50 |
| hsa-mir-365a-5p | 0 | 0 |
| hsa-mir-365b-3p | 54 | 50 |
| hsa-mir-365b-5p | 0 | 0 |
| hsa-mir-3676-3p | 0 | 0 |
| hsa-mir-3676-5p | 5 | 5 |
| hsa-mir-369-3p | 8 | 8 |
| hsa-mir-369-5p | 0 | 0 |
| hsa-mir-3690 | 542 | 136 |
| hsa-mir-371b-3p | 0 | 0 |
| hsa-mir-371b-5p | 75 | 30 |
| hsa-mir-374a-3p | 1237 | 832 |
| hsa-mir-374a-5p | 272 | 130 |
| hsa-mir-374b-3p | 49 | 44 |
| hsa-mir-374b-5p | 500 | 356 |
| hsa-mir-378a-3p | 2491 | 1174 |
| hsa-mir-378a-5p | 0 | 0 |
| hsa-mir-378c | 373 | 0 |
| hsa-mir-378d | 32 | 8 |
| hsa-mir-379-3p | 0 | 0 |
| hsa-mir-379-5p | 5 | 5 |
| hsa-mir-382-3p | 0 | 0 |
| hsa-mir-382-5p | 26 | 26 |
| hsa-mir-3909 | 16 | 11 |
| hsa-mir-3928 | 13 | 13 |
| hsa-mir-409-3p | 13 | 7 |
| hsa-mir-409-5p | 0 | 0 |
| hsa-mir-410 | 8 | 8 |
| hsa-mir-421 | 236 | 33 |
| hsa-mir-423-3p | 1965 | 1232 |
| hsa-mir-423-5p | 25999 | 18415 |
| hsa-mir-424-3p | 857 | 565 |
| hsa-mir-424-5p | 955 | 99 |
| hsa-mir-425-3p | 727 | 9 |
| hsa-mir-425-5p | 1514 | 838 |
| hsa-mir-4286 | 654 | 12 |
| hsa-mir-429 | 5 | 5 |
| hsa-mir-432-3p | 0 | 0 |
| hsa-mir-432-5p | 26 | 10 |
| hsa-mir-433 | 15 | 15 |
| hsa-mir-4433-3p | 168 | 71 |
| hsa-mir-4433-5p | 0 | 0 |
| hsa-mir-4443 | 54 | 0 |
| hsa-mir-4454 | 8 | 0 |
| hsa-mir-4477b | 5 | 5 |
| hsa-mir-4487 | 33 | 0 |
| hsa-mir-4500 | 6 | 0 |
| hsa-mir-4508 | 8 | 0 |
| hsa-mir-450a-3p | 59 | 34 |
| hsa-mir-450a-5p | 136 | 58 |
| hsa-mir-450b-3p | 0 | 0 |
| hsa-mir-450b-5p | 81 | 0 |
| hsa-mir-451a | 670 | 280 |
| hsa-mir-454-3p | 7 | 7 |
| hsa-mir-454-5p | 0 | 0 |
| hsa-mir-4676-3p | 0 | 0 |
| hsa-mir-4676-5p | 5 | 5 |
| hsa-mir-4724-3p | 0 | 0 |
| hsa-mir-4724-5p | 20 | 20 |
| hsa-mir-4750 | 5 | 5 |
| hsa-mir-4772-3p | 33 | 33 |
| hsa-mir-4772-5p | 33 | 24 |
| hsa-mir-4773 | 6 | 6 |
| hsa-mir-4791 | 26 | 0 |
| hsa-mir-484 | 54 | 43 |
| hsa-mir-485-3p | 6 | 6 |
| hsa-mir-485-5p | 10 | 5 |
| hsa-mir-486-3p | 36 | 27 |
| hsa-mir-486-5p | 33 | 22 |
| hsa-mir-487b | 12 | 12 |
| hsa-mir-491-3p | 0 | 0 |
| hsa-mir-491-5p | 6 | 6 |
| hsa-mir-495 | 24 | 8 |
| hsa-mir-499a-3p | 0 | 0 |
| hsa-mir-499a-5p | 105 | 62 |
| hsa-mir-499b-3p | 0 | 0 |
| hsa-mir-499b-5p | 5 | 5 |
| hsa-mir-500a-3p | 70 | 5 |
| hsa-mir-500a-5p | 0 | 0 |
| hsa-mir-500b | 0 | 0 |
| hsa-mir-501-3p | 6 | 6 |
| hsa-mir-501-5p | 0 | 0 |
| hsa-mir-5010-3p | 0 | 0 |
| hsa-mir-5010-5p | 37 | 24 |
| hsa-mir-502-3p | 110 | 83 |
| hsa-mir-502-5p | 0 | 0 |
| hsa-mir-503 | 19 | 6 |
| hsa-mir-504 | 87 | 20 |
| hsa-mir-505-3p | 41 | 20 |
| hsa-mir-505-5p | 254 | 48 |
| hsa-mir-5187-3p | 0 | 0 |
| hsa-mir-5187-5p | 21 | 15 |
| hsa-mir-532-3p | 26 | 26 |
| hsa-mir-532-5p | 857 | 766 |
| hsa-mir-539-3p | 34 | 29 |
| hsa-mir-539-5p | 0 | 0 |
| hsa-mir-542-3p | 243 | 112 |
| hsa-mir-542-5p | 9 | 0 |
| hsa-mir-543 | 43 | 21 |
| hsa-mir-548aa | 0 | 0 |
| hsa-mir-548ad | 0 | 0 |
| hsa-mir-548ae | 0 | 0 |
| hsa-mir-548ak | 10 | 0 |
| hsa-mir-548am-3p | 0 | 0 |
| hsa-mir-548am-5p | 21 | 0 |
| hsa-mir-548ap-3p | 0 | 0 |
| hsa-mir-548ap-5p | 93 | 0 |
| hsa-mir-548au-3p | 0 | 0 |
| hsa-mir-548au-5p | 21 | 6 |
| hsa-mir-548av-3p | 28 | 0 |
| hsa-mir-548av-5p | 246 | 0 |
| hsa-mir-548c-3p | 0 | 0 |
| hsa-mir-548c-5p | 21 | 0 |
| hsa-mir-548d-3p | 0 | 0 |
| hsa-mir-548d-5p | 28 | 8 |
| hsa-mir-548e | 564 | 423 |
| hsa-mir-548f | 6 | 0 |
| hsa-mir-548j | 93 | 61 |
| hsa-mir-548k | 246 | 212 |
| hsa-mir-548l | 54 | 17 |
| hsa-mir-548o-3p | 28 | 10 |
| hsa-mir-548o-5p | 21 | 0 |
| hsa-mir-548t-3p | 0 | 0 |
| hsa-mir-548t-5p | 10 | 10 |
| hsa-mir-548u | 9 | 0 |
| hsa-mir-548w | 30 | 0 |
| hsa-mir-551a | 63 | 44 |
| hsa-mir-556-3p | 11 | 5 |
| hsa-mir-556-5p | 0 | 0 |
| hsa-mir-574-3p | 82 | 67 |
| hsa-mir-574-5p | 0 | 0 |
| hsa-mir-576-3p | 78 | 33 |
| hsa-mir-576-5p | 73 | 68 |
| hsa-mir-582-3p | 102 | 7 |
| hsa-mir-582-5p | 112 | 34 |
| hsa-mir-584-3p | 0 | 0 |
| hsa-mir-584-5p | 101 | 0 |
| hsa-mir-589-3p | 0 | 0 |
| hsa-mir-589-5p | 48 | 9 |
| hsa-mir-590-3p | 22 | 13 |
| hsa-mir-590-5p | 21 | 15 |
| hsa-mir-598 | 152 | 106 |
| hsa-mir-618 | 60 | 51 |
| hsa-mir-619 | 0 | 0 |
| hsa-mir-625-3p | 0 | 0 |
| hsa-mir-625-5p | 50 | 11 |
| hsa-mir-628-3p | 9 | 0 |
| hsa-mir-628-5p | 156 | 112 |
| hsa-mir-629-3p | 6 | 6 |
| hsa-mir-629-5p | 139 | 21 |
| hsa-mir-637 | 0 | 0 |
| hsa-mir-641 | 15 | 0 |
| hsa-mir-642a-3p | 10 | 10 |
| hsa-mir-642a-5p | 0 | 0 |
| hsa-mir-644b-3p | 0 | 0 |
| hsa-mir-651 | 16 | 9 |
| hsa-mir-652-3p | 217 | 115 |
| hsa-mir-652-5p | 8 | 0 |
| hsa-mir-659-3p | 0 | 0 |
| hsa-mir-659-5p | 15 | 15 |
| hsa-mir-660-3p | 11 | 11 |
| hsa-mir-660-5p | 59 | 27 |
| hsa-mir-664-3p | 40 | 35 |
| hsa-mir-664-5p | 745 | 0 |
| hsa-mir-7-1-3p | 90 | 43 |
| hsa-mir-7-2-3p | 0 | 0 |
| hsa-mir-7-5p | 144 | 42 |
| hsa-mir-720 | 447 | 155 |
| hsa-mir-744-3p | 0 | 0 |
| hsa-mir-744-5p | 2313 | 1572 |
| hsa-mir-766-3p | 14 | 7 |
| hsa-mir-766-5p | 19 | 5 |
| hsa-mir-769-3p | 0 | 0 |
| hsa-mir-769-5p | 15 | 15 |
| hsa-mir-873-3p | 0 | 0 |
| hsa-mir-873-5p | 57 | 16 |
| hsa-mir-889 | 19 | 19 |
| hsa-mir-9-3p | 6 | 6 |
| hsa-mir-9-5p | 15 | 12 |
| hsa-mir-92a-1-5p | 67 | 57 |
| hsa-mir-92a-2-5p | 0 | 0 |
| hsa-mir-92a-3p | 4101 | 3246 |
| hsa-mir-92b-3p | 10 | 0 |
| hsa-mir-92b-5p | 0 | 0 |
| hsa-mir-93-3p | 33 | 7 |
| hsa-mir-93-5p | 4794 | 3686 |
| hsa-mir-935 | 11 | 0 |
| hsa-mir-941 | 1460 | 960 |
| hsa-mir-942 | 10 | 0 |
| hsa-mir-98 | 1221 | 882 |
| hsa-mir-99a-3p | 0 | 0 |
| hsa-mir-99a-5p | 33 | 11 |
| hsa-mir-99b-3p | 22 | 10 |
| hsa-mir-99b-5p | 295 | 279 |
