# Supplementary material for: The Repertoire and Features of Human Platelet microRNAs
Source: PLoS One. 2012 Dec 4;7(12):e50746. doi: 10.1371/journal.pone.0050746 (PMC3514217; doi:10.1371/journal.pone.0050746)
Supplement: Database S1 — (ZIP) [file pone.0050746.s011.zip › Supporting Platelet microRNA sequence database S1/s7_sequence_s7run61.html]

Analysis ouput 

# s7run61

## Triming statistics

|  |  |  |  |  |  |  |  |  |  |  |  |  |  |
| --- | --- | --- | --- | --- | --- | --- | --- | --- | --- | --- | --- | --- | --- |
| Tissue | ADP5 Len. | ADP3 Len. | Total Reads | Unique Reads | Reads with adapter | Unique Reads with adapter | Length Filter(<10) | PolyN Filter(>9) | Copy Number Filter(<=4) | Usable Reads | Usable Unique Reads | Usable Percentage |  |
| s7\_sequence\_s7run61 | 0 | [TN][CN][GN][TN][AN][TN] | 6366352 | 306300 | 5860813 | 148953 | 5313 | 3441 | 171894 | 5680165 | 18071 | 0.892216610077482 |
| Sum(Avg) |  |  | 6366352 | 306300 | 5860813 | 148953 | 5313 | 3441 | 171894 | 5680165 | 18071 | 0.892216610077482 |

#### Abbreviations:

|  |  |  |
| --- | --- | --- |
| Tissue | Abbreviation. |  |
| s7\_sequence\_s7run61\_t\_f | \_ |

## Mismatch positions table

|  |  |  |  |  |  |  |  |  |  |  |  |  |  |  |  |  |  |  |  |  |  |  |  |  |  |  |  |  |  |  |  |  |  |  |  |  |
| --- | --- | --- | --- | --- | --- | --- | --- | --- | --- | --- | --- | --- | --- | --- | --- | --- | --- | --- | --- | --- | --- | --- | --- | --- | --- | --- | --- | --- | --- | --- | --- | --- | --- | --- | --- | --- |
| microRNA | Tissue | Exact | Loose | Pos. | Len. | MM\_Total | -2 | -1 | \_ | 1 | 2 | 3 | 4 | 5 | 6 | 7 | 8 | 9 | 10 | 11 | 12 | 13 | 14 | 15 | 16 | 17 | 18 | 19 | 20 | 21 | 22 | 23 | 24 | 25 | 26 |  |
| hsa-let-7a-3p(hsa-let-7a-1) | \_ | 174438 | 3 | 56 | 21 | 3 |  |  |  |  |  |  |  |  |  |  |  |  |  |  |  |  |  |  |  |  |  |  |  |  | 3 |  |  |  |  |
| hsa-let-7a-3p(hsa-let-7a-3) | \_ | 174518 | 3 | 51 | 21 | 3 |  |  |  |  |  |  |  |  |  |  |  |  |  |  |  |  |  |  |  |  |  |  |  |  | 3 |  |  |  |  |
| hsa-let-7a-5p(hsa-let-7a-1) | \_ | 174438 | 9386 | 5 | 22 | 8749 |  |  | 57 | 250 | 199 | 226 | 77 | 64 | 284 | 217 | 185 | 609 | 187 | 60 | 186 | 242 | 104 | 81 | 144 | 461 | 110 | 60 | 132 | 378 | 550 | 3886 |  |  |  |
| hsa-let-7a-5p(hsa-let-7a-2) | \_ | 174459 | 9446 | 4 | 22 | 8715 |  | 12 | 57 | 250 | 148 | 226 | 77 | 64 | 284 | 217 | 185 | 609 | 187 | 60 | 193 | 242 | 104 | 81 | 144 | 461 | 110 | 60 | 132 | 378 | 550 | 3884 |  |  |  |
| hsa-let-7a-5p(hsa-let-7a-3) | \_ | 174518 | 9386 | 3 | 22 | 8749 |  |  | 57 | 250 | 199 | 226 | 77 | 64 | 284 | 217 | 185 | 609 | 187 | 60 | 186 | 242 | 104 | 81 | 144 | 461 | 110 | 60 | 132 | 378 | 550 | 3886 |  |  |  |
| hsa-let-7b-5p(hsa-let-7b) | \_ | 47973 | 4596 | 5 | 22 | 4932 |  |  | 14 | 41 | 43 | 52 | 36 | 32 | 71 | 53 | 57 | 57 | 47 | 18 | 15 | 54 | 21 | 16 | 107 | 24 | 102 | 169 | 101 | 537 | 1128 | 2137 |  |  |  |
| hsa-let-7c(hsa-let-7c) | \_ | 4628 | 868 | 10 | 22 | 864 |  |  |  |  |  |  |  |  |  |  |  | 6 |  |  | 142 |  |  |  |  | 24 |  | 60 | 254 | 72 | 149 | 157 |  |  |  |
| hsa-let-7d-3p(hsa-let-7d) | \_ | 98375 | 11 | 61 | 22 | 11 |  |  |  |  |  |  |  |  |  |  |  |  |  |  |  |  |  |  |  |  |  |  |  |  | 11 |  |  |  |  |
| hsa-let-7d-5p(hsa-let-7d) | \_ | 98375 | 4673 | 7 | 22 | 4625 |  |  | 51 | 530 | 45 | 102 | 31 | 36 | 151 | 117 | 115 | 177 | 82 | 33 | 43 | 390 | 83 | 75 | 81 | 321 | 57 | 63 | 36 | 225 | 316 | 1465 |  |  |  |
| hsa-let-7e-5p(hsa-let-7e) | \_ | 31101 | 2931 | 7 | 22 | 3048 |  |  |  | 43 | 24 | 41 |  | 13 | 42 | 51 | 33 | 164 | 50 | 12 | 201 | 92 | 6 | 23 | 64 | 79 | 11 | 13 | 20 | 326 | 312 | 1428 |  |  |  |
| hsa-let-7f-2-3p(hsa-let-7f-2) | \_ | 617753 | 5 | 57 | 22 | 5 |  |  |  |  |  |  |  |  |  |  |  |  |  |  |  |  |  |  |  |  |  |  |  |  | 5 |  |  |  |  |
| hsa-let-7f-5p(hsa-let-7f-1) | \_ | 599505 | 25610 | 6 | 22 | 24481 |  | 12 | 402 | 597 | 744 | 806 | 281 | 260 | 1007 | 759 | 714 | 861 | 684 | 178 | 233 | 303 | 413 | 315 | 748 | 1623 | 348 | 430 | 438 | 1126 | 1284 | 9915 |  |  |  |
| hsa-let-7f-5p(hsa-let-7f-2) | \_ | 617753 | 25955 | 7 | 22 | 24807 |  | 19 | 409 | 608 | 741 | 824 | 288 | 260 | 1036 | 773 | 728 | 886 | 698 | 178 | 195 | 311 | 430 | 323 | 767 | 1668 | 359 | 437 | 438 | 1173 | 1343 | 9915 |  |  |  |
| hsa-let-7g-5p(hsa-let-7g) | \_ | 96425 | 6266 | 4 | 22 | 5074 |  |  |  | 109 | 124 | 129 | 55 | 42 | 181 | 108 | 118 | 155 | 93 | 27 | 239 | 43 | 57 | 73 | 111 | 205 | 203 | 137 | 26 | 167 | 227 | 2445 |  |  |  |
| hsa-let-7i-3p(hsa-let-7i) | \_ | 53344 | 7 | 61 | 22 | 7 |  |  |  |  |  |  |  |  |  |  |  |  |  |  |  |  |  |  |  |  |  |  |  |  |  | 7 |  |  |  |
| hsa-let-7i-5p(hsa-let-7i) | \_ | 53344 | 7008 | 5 | 22 | 6669 |  |  |  | 47 | 44 | 84 | 16 | 26 | 105 | 74 | 45 | 98 | 47 | 17 | 68 | 23 | 28 | 39 | 101 | 61 | 157 | 52 | 103 | 103 | 209 | 5122 |  |  |  |
| hsa-mir-1(hsa-mir-1-1) | \_ | 13241 | 368 | 45 | 22 | 368 |  |  | 6 | 10 | 7 | 7 | 14 | 9 | 6 | 2 | 12 | 9 | 11 | 10 | 3 | 8 | 13 |  | 27 | 30 | 6 | 2 | 25 | 5 | 119 | 27 |  |  |  |
| hsa-mir-1(hsa-mir-1-2) | \_ | 13338 | 368 | 52 | 22 | 368 |  |  | 6 | 10 | 7 | 7 | 14 | 9 | 6 | 2 | 12 | 9 | 11 | 10 | 3 | 8 | 13 |  | 27 | 30 | 6 | 2 | 25 | 5 | 119 | 27 |  |  |  |
| hsa-mir-101-3p(hsa-mir-101-1) | \_ | 15356 | 919 | 46 | 21 | 882 |  |  | 23 | 9 | 7 | 12 |  | 7 | 8 | 4 | 41 | 5 | 8 | 5 | 7 | 3 | 6 | 3 | 24 | 27 |  | 5 | 54 | 76 | 548 |  |  |  |  |
| hsa-mir-101-3p(hsa-mir-101-2) | \_ | 15488 | 919 | 48 | 21 | 882 |  |  | 23 | 9 | 7 | 12 |  | 7 | 8 | 4 | 41 | 5 | 8 | 5 | 7 | 3 | 6 | 3 | 24 | 27 |  | 5 | 54 | 76 | 548 |  |  |  |  |
| hsa-mir-103a-3p(hsa-mir-103a-1) | \_ | 159494 | 13593 | 47 | 23 | 13605 |  |  | 38 | 154 | 100 | 332 | 113 | 133 | 354 | 173 | 87 | 87 | 97 | 173 | 124 | 413 | 150 | 156 | 96 | 185 | 206 | 74 | 124 | 213 | 2317 | 6416 | 1287 | 3 |  |
| hsa-mir-103a-3p(hsa-mir-103a-2) | \_ | 162282 | 13604 | 47 | 23 | 13616 |  |  | 38 | 154 | 100 | 332 | 113 | 133 | 365 | 173 | 87 | 87 | 97 | 173 | 124 | 413 | 150 | 156 | 96 | 185 | 206 | 74 | 124 | 213 | 2317 | 6416 | 1287 | 3 |  |
| hsa-mir-106b-3p(hsa-mir-106b) | \_ | 1748 | 87 | 51 | 22 | 105 |  |  |  |  |  |  |  |  |  |  |  |  |  |  |  |  |  |  |  |  |  |  |  | 6 | 81 | 18 |  |  |  |
| hsa-mir-106b-5p(hsa-mir-106b) | \_ | 1748 | 103 | 11 | 21 | 103 |  |  |  |  |  |  |  |  |  |  |  |  |  |  |  |  |  |  |  | 9 |  |  |  | 12 | 82 |  |  |  |  |
| hsa-mir-107(hsa-mir-107) | \_ | 52279 | 5197 | 49 | 23 | 5390 |  |  | 9 | 22 |  | 50 | 14 | 7 | 89 | 32 | 7 | 18 | 14 | 31 | 13 | 87 | 25 | 25 | 18 | 42 | 36 | 13 | 31 | 43 | 2317 | 2351 | 96 |  |  |
| hsa-mir-10a-5p(hsa-mir-10a) | \_ | 629 | 15 | 21 | 23 | 7 |  |  |  |  |  |  |  |  |  |  |  |  |  |  |  |  |  |  |  |  |  |  |  |  | 7 |  |  |  |  |
| hsa-mir-122-5p(hsa-mir-122) | \_ | 13 | 5 | 14 | 22 | 5 |  |  |  |  |  |  |  |  |  |  |  |  |  |  |  |  |  |  |  |  |  |  |  |  |  | 5 |  |  |  |
| hsa-mir-1250(hsa-mir-1250) | \_ | 48 | 6 | 23 | 21 | 6 |  |  |  |  |  |  |  |  |  |  |  |  |  |  |  |  |  |  |  |  |  |  |  | 6 |  |  |  |  |  |
| hsa-mir-126-3p(hsa-mir-126) | \_ | 11971 | 1118 | 51 | 22 | 1120 |  |  |  |  | 5 |  |  |  | 22 | 27 |  |  |  |  |  |  |  |  |  |  | 6 |  |  |  | 12 | 1041 | 7 |  |  |
| hsa-mir-126-5p(hsa-mir-126) | \_ | 11971 | 1216 | 14 | 21 | 1125 |  |  |  | 11 | 5 |  |  |  |  |  |  | 40 | 10 |  |  | 5 |  | 5 | 16 | 8 | 14 | 7 | 30 | 22 | 952 |  |  |  |  |
| hsa-mir-1260a(hsa-mir-1260a) | \_ |  | 398 | 13 | 18 | 410 |  |  |  |  |  | 3 |  | 2 |  |  |  | 398 |  |  |  |  |  |  |  |  |  | 3 | 4 |  |  |  |  |  |  |
| hsa-mir-1260b(hsa-mir-1260b) | \_ | 34 | 1348 | 9 | 19 | 1532 |  |  | 6 |  |  | 12 | 28 | 10 |  |  |  | 1346 |  |  |  |  |  |  |  | 5 |  | 75 | 50 |  |  |  |  |  |  |
| hsa-mir-1261(hsa-mir-1261) | \_ |  | 180 | 4 | 19 | 284 |  |  |  |  |  |  |  |  |  |  |  |  |  |  | 180 |  |  |  |  |  |  |  | 104 |  |  |  |  |  |  |
| hsa-mir-127-3p(hsa-mir-127) | \_ | 4576 | 1229 | 56 | 22 | 1253 |  |  |  |  | 5 | 6 |  |  |  | 18 |  |  |  |  |  |  |  |  | 7 | 5 |  |  |  | 118 | 101 | 993 |  |  |  |
| hsa-mir-1271-5p(hsa-mir-1271) | \_ | 85 | 8 | 14 | 22 | 8 |  |  |  |  |  |  |  |  |  |  |  |  |  |  |  |  |  |  |  |  |  |  |  |  | 8 |  |  |  |  |
| hsa-mir-1273c(hsa-mir-1273c) | \_ | 6 | 7 | 9 | 22 | 7 |  |  |  |  |  |  |  |  |  |  |  |  |  |  |  |  |  |  |  |  |  |  |  |  |  | 7 |  |  |  |
| hsa-mir-128(hsa-mir-128-1) | \_ | 5461 | 702 | 49 | 21 | 691 |  |  |  | 5 | 5 |  | 5 |  |  |  |  |  |  | 5 | 4 |  |  | 5 | 22 | 5 | 46 | 6 | 5 | 147 | 431 |  |  |  |  |
| hsa-mir-128(hsa-mir-128-2) | \_ | 3776 | 610 | 51 | 21 | 621 |  |  |  | 5 | 5 |  | 5 |  |  |  |  |  |  | 5 | 4 |  |  | 5 | 11 | 5 | 37 | 6 | 5 | 97 | 431 |  |  |  |  |
| hsa-mir-1280(hsa-mir-1280) | \_ |  | 61 | 4 | 17 | 73 |  |  | 38 |  |  |  |  |  |  |  |  |  |  |  |  |  |  |  | 6 | 29 |  |  |  |  |  |  |  |  |  |
| hsa-mir-1285-3p(hsa-mir-1285-1) | \_ | 42 | 18 | 50 | 22 | 18 |  |  |  |  |  |  |  |  |  |  |  |  |  |  |  |  |  |  |  |  |  |  |  | 5 |  | 13 |  |  |  |
| hsa-mir-1285-3p(hsa-mir-1285-2) | \_ | 27 | 18 | 51 | 22 | 18 |  |  |  |  |  |  |  |  |  |  |  |  |  |  |  |  |  |  |  |  |  |  |  | 5 |  | 13 |  |  |  |
| hsa-mir-1287(hsa-mir-1287) | \_ | 240 | 21 | 15 | 22 | 21 |  |  |  |  |  |  |  |  |  |  |  |  |  |  |  |  |  |  |  |  |  |  |  |  | 6 | 15 |  |  |  |
| hsa-mir-1299(hsa-mir-1299) | \_ | 14 | 5 | 61 | 22 | 5 |  |  |  |  |  |  |  |  |  |  |  |  |  |  |  |  |  |  |  |  |  |  |  | 5 |  |  |  |  |  |
| hsa-mir-1301(hsa-mir-1301) | \_ | 327 | 35 | 47 | 24 | 35 |  |  |  |  |  |  |  |  |  |  |  |  |  |  |  |  |  |  |  |  |  |  |  |  |  |  | 6 |  | 29 |
| hsa-mir-1304-3p(hsa-mir-1304) | \_ | 17 | 49 | 52 | 22 | 49 |  |  |  |  |  |  |  |  |  |  |  |  |  |  |  | 49 |  |  |  |  |  |  |  |  |  |  |  |  |  |
| hsa-mir-1307-3p(hsa-mir-1307) | \_ | 7435 | 957 | 79 | 22 | 1009 |  |  |  |  |  |  |  |  |  |  |  |  |  |  |  |  |  |  |  |  | 102 |  |  | 51 | 186 | 661 | 9 |  |  |
| hsa-mir-1307-5p(hsa-mir-1307) | \_ | 7435 | 150 | 40 | 21 | 150 |  |  |  |  |  |  |  |  | 5 |  |  |  |  |  |  | 5 |  |  |  |  |  |  |  | 6 | 134 |  |  |  |  |
| hsa-mir-130a-3p(hsa-mir-130a) | \_ | 4616 | 351 | 54 | 22 | 386 |  |  |  | 12 | 5 |  |  |  |  |  |  |  |  |  |  |  |  |  |  |  |  |  | 6 |  | 284 | 79 |  |  |  |
| hsa-mir-130b-3p(hsa-mir-130b) | \_ | 874 | 62 | 50 | 22 | 62 |  |  |  |  |  |  |  |  |  |  |  |  |  |  |  |  |  |  |  |  |  |  |  |  | 31 | 31 |  |  |  |
| hsa-mir-132-3p(hsa-mir-132) | \_ | 25 | 19 | 58 | 22 | 24 |  |  |  |  |  |  |  |  |  |  |  |  |  |  |  |  |  |  |  |  |  |  |  |  |  | 19 | 5 |  |  |
| hsa-mir-134(hsa-mir-134) | \_ | 1025 | 272 | 7 | 22 | 282 |  |  |  |  |  |  |  |  |  |  |  |  |  |  |  |  |  |  |  |  |  |  |  | 28 | 138 | 116 |  |  |  |
| hsa-mir-136-5p(hsa-mir-136) | \_ | 162 | 6 | 14 | 23 | 6 |  |  |  |  |  |  |  |  |  |  |  |  |  |  |  |  |  |  |  |  |  |  |  |  |  |  | 6 |  |  |
| hsa-mir-139-3p(hsa-mir-139) | \_ | 806 | 38 | 43 | 22 | 50 |  |  |  |  |  |  |  |  |  |  |  |  |  |  |  |  |  |  |  |  |  |  |  |  | 19 | 19 | 12 |  |  |
| hsa-mir-139-5p(hsa-mir-139) | \_ | 806 | 14 | 6 | 22 | 14 |  |  |  |  |  |  |  |  |  |  |  |  |  |  |  |  |  |  |  |  |  |  |  |  | 7 | 7 |  |  |  |
| hsa-mir-140-3p(hsa-mir-140) | \_ | 57735 | 8359 | 61 | 21 | 8506 |  |  |  | 21 | 25 | 85 | 94 | 25 | 116 | 28 | 58 | 78 | 5 | 44 | 37 | 9 | 21 | 7 | 168 | 224 | 106 | 95 | 40 | 16 | 575 | 6629 |  |  |  |
| hsa-mir-142-3p(hsa-mir-142) | \_ | 15794 | 35 | 51 | 23 | 30 |  |  |  |  |  |  |  |  |  |  |  |  |  |  |  |  |  |  |  |  |  |  |  |  |  | 24 | 6 |  |  |
| hsa-mir-142-5p(hsa-mir-142) | \_ | 15794 | 1011 | 15 | 21 | 1003 |  | 99 | 154 | 102 |  |  |  |  |  |  |  |  |  |  |  |  |  | 46 | 5 | 55 | 71 | 21 | 331 | 22 |  | 97 |  |  |  |
| hsa-mir-143-3p(hsa-mir-143) | \_ | 2915 | 181 | 60 | 21 | 175 |  |  |  |  |  |  |  |  |  |  |  |  |  | 5 | 5 |  |  |  |  |  | 6 |  |  | 35 | 124 |  |  |  |  |
| hsa-mir-143-5p(hsa-mir-143) | \_ | 2915 | 9 | 26 | 22 | 9 |  |  |  |  |  |  |  |  |  |  |  |  |  |  |  |  |  |  |  |  |  |  |  |  | 9 |  |  |  |  |
| hsa-mir-144-3p(hsa-mir-144) | \_ | 475 | 39 | 51 | 20 | 39 |  |  |  |  |  |  |  |  |  | 17 |  |  |  |  |  |  |  |  |  |  |  |  |  | 22 |  |  |  |  |  |
| hsa-mir-146a-5p(hsa-mir-146a) | \_ | 2117 | 319 | 20 | 22 | 319 |  |  |  |  |  |  |  |  |  |  |  |  |  |  |  |  |  |  |  |  |  |  |  | 3 | 29 | 287 |  |  |  |
| hsa-mir-146b-5p(hsa-mir-146b) | \_ | 493 | 98 | 8 | 22 | 98 |  |  |  |  |  |  |  |  |  |  |  |  |  |  |  |  |  |  |  |  | 3 |  |  |  | 26 | 69 |  |  |  |
| hsa-mir-148a-3p(hsa-mir-148a) | \_ | 1485 | 100 | 43 | 22 | 134 |  |  |  |  |  |  |  |  |  |  |  | 5 |  |  |  |  |  |  |  | 9 |  |  |  |  | 72 | 48 |  |  |  |
| hsa-mir-148b-3p(hsa-mir-148b) | \_ | 3699 | 661 | 62 | 22 | 693 |  |  | 6 |  | 9 |  |  | 5 |  | 6 |  |  | 5 |  |  |  |  |  |  | 14 | 10 |  | 28 | 25 | 157 | 428 |  |  |  |
| hsa-mir-151a-3p(hsa-mir-151a) | \_ | 12826 | 907 | 46 | 21 | 823 |  |  |  | 25 |  | 17 |  |  | 5 |  |  |  |  |  |  |  | 6 | 21 |  | 5 | 11 |  | 16 | 86 | 619 | 12 |  |  |  |
| hsa-mir-151a-5p(hsa-mir-151a) | \_ | 12826 | 117 | 10 | 21 | 117 |  |  | 11 | 5 | 16 |  |  |  |  |  |  | 10 |  | 18 | 8 | 7 |  |  |  | 29 |  |  |  | 7 | 6 |  |  |  |  |
| hsa-mir-152(hsa-mir-152) | \_ | 9512 | 2531 | 53 | 21 | 2536 |  |  |  | 12 | 6 | 8 |  | 9 |  | 6 |  | 5 |  |  | 7 |  |  | 7 |  | 27 | 5 |  |  | 19 | 2425 |  |  |  |  |
| hsa-mir-155-5p(hsa-mir-155) | \_ | 130 | 42 | 3 | 23 | 42 |  |  |  |  |  |  |  |  |  |  |  |  |  |  |  |  |  |  |  |  |  |  |  |  | 42 |  |  |  |  |
| hsa-mir-15a-5p(hsa-mir-15a) | \_ | 843 | 17 | 13 | 22 | 17 |  |  |  |  |  |  |  |  |  |  |  |  |  |  |  |  |  |  |  |  |  |  |  |  | 11 | 6 |  |  |  |
| hsa-mir-15b-5p(hsa-mir-15b) | \_ | 1603 | 48 | 19 | 22 | 48 |  |  |  |  |  |  |  |  |  |  |  |  |  |  |  |  |  |  |  |  |  |  |  | 39 |  | 9 |  |  |  |
| hsa-mir-16-5p(hsa-mir-16-1) | \_ | 2989 | 229 | 13 | 22 | 187 |  |  |  |  |  | 3 | 4 |  |  | 11 |  | 16 |  | 3 | 5 |  |  | 4 |  | 5 |  |  | 5 | 6 | 41 | 84 |  |  |  |
| hsa-mir-16-5p(hsa-mir-16-2) | \_ | 2979 | 229 | 9 | 22 | 187 |  |  |  |  |  | 3 | 4 |  |  | 11 |  | 16 |  | 3 | 5 |  |  | 4 |  | 5 |  |  | 5 | 6 | 41 | 84 |  |  |  |
| hsa-mir-17-3p(hsa-mir-17) | \_ | 1126 | 58 | 50 | 22 | 58 |  |  |  |  |  |  |  |  |  |  |  |  |  |  |  |  |  |  |  |  |  |  |  |  |  | 44 | 14 |  |  |
| hsa-mir-17-5p(hsa-mir-17) | \_ | 1126 | 40 | 13 | 23 | 40 |  |  |  |  |  |  |  |  |  |  |  |  |  |  |  |  |  |  |  |  |  |  |  |  |  | 40 |  |  |  |
| hsa-mir-181a-3p(hsa-mir-181a-1) | \_ | 4683 | 7 | 63 | 22 | 7 |  |  |  |  |  |  |  |  |  |  |  |  |  |  |  |  |  |  |  |  |  |  |  | 7 |  |  |  |  |  |
| hsa-mir-181a-5p(hsa-mir-181a-1) | \_ | 4683 | 136 | 23 | 23 | 136 |  |  |  |  |  |  |  |  |  |  |  |  | 4 |  |  |  |  |  | 2 |  |  | 13 |  |  | 18 | 93 | 6 |  |  |
| hsa-mir-181a-5p(hsa-mir-181a-2) | \_ | 4366 | 136 | 38 | 23 | 136 |  |  |  |  |  |  |  |  |  |  |  |  | 4 |  |  |  |  |  | 2 |  |  | 13 |  |  | 18 | 93 | 6 |  |  |
| hsa-mir-181b-3p(hsa-mir-181b-1) | \_ | 342 | 12 | 75 | 21 | 12 |  |  |  |  |  |  |  |  |  |  |  |  |  |  |  |  |  |  |  |  |  |  |  | 12 |  |  |  |  |  |
| hsa-mir-181b-5p(hsa-mir-181b-1) | \_ | 342 | 50 | 35 | 23 | 50 |  |  |  |  |  |  |  |  |  |  |  |  |  |  |  |  |  |  |  |  |  |  |  |  | 20 | 30 |  |  |  |
| hsa-mir-181b-5p(hsa-mir-181b-2) | \_ | 337 | 50 | 15 | 23 | 50 |  |  |  |  |  |  |  |  |  |  |  |  |  |  |  |  |  |  |  |  |  |  |  |  | 20 | 30 |  |  |  |
| hsa-mir-181c-3p(hsa-mir-181c) | \_ | 443 | 106 | 64 | 22 | 106 |  |  |  |  |  |  |  |  |  |  |  |  |  |  |  |  |  |  |  |  |  |  |  |  |  | 106 |  |  |  |
| hsa-mir-181d(hsa-mir-181d) | \_ | 292 | 27 | 35 | 23 | 27 |  |  |  |  |  |  |  |  |  |  |  |  |  |  |  |  |  |  |  |  |  |  |  |  | 5 | 22 |  |  |  |
| hsa-mir-185-3p(hsa-mir-185) | \_ | 123307 | 74 | 49 | 22 | 86 |  |  |  |  |  |  |  |  |  |  |  |  |  |  |  |  |  |  |  |  |  |  |  | 6 | 61 | 19 |  |  |  |
| hsa-mir-185-5p(hsa-mir-185) | \_ | 123307 | 9752 | 14 | 22 | 9647 |  |  | 32 | 138 | 171 | 71 | 124 | 48 | 114 | 73 | 97 | 38 | 97 | 126 | 91 | 219 | 104 | 83 | 1109 | 281 | 554 | 282 | 136 | 172 | 1441 | 4046 |  |  |  |
| hsa-mir-186-5p(hsa-mir-186) | \_ | 4504 | 186 | 14 | 22 | 100 |  |  |  | 5 |  |  |  |  |  |  |  |  |  |  | 11 |  | 14 |  |  |  |  |  |  |  | 33 | 31 | 6 |  |  |
| hsa-mir-1908(hsa-mir-1908) | \_ | 360 | 277 | 11 | 21 | 288 |  |  |  |  |  |  |  |  |  |  |  |  |  |  |  |  |  |  |  |  |  |  |  | 41 | 247 |  |  |  |  |
| hsa-mir-191-5p(hsa-mir-191) | \_ | 25056 | 2904 | 15 | 23 | 3034 |  |  |  | 46 | 13 | 7 | 36 | 15 |  | 40 | 5 | 11 | 287 | 454 | 211 | 13 | 6 |  | 6 | 20 | 44 | 5 | 14 | 80 | 32 | 605 | 1025 | 59 |  |
| hsa-mir-192-5p(hsa-mir-192) | \_ | 5263 | 241 | 23 | 21 | 241 |  |  |  |  |  |  |  |  |  |  |  |  |  |  |  |  |  |  |  | 10 |  |  |  | 39 | 106 | 86 |  |  |  |
| hsa-mir-196b-5p(hsa-mir-196b) | \_ | 132 | 92 | 14 | 22 | 92 |  |  |  |  |  |  |  |  |  |  |  |  |  |  |  |  |  |  |  |  |  |  |  |  | 56 | 36 |  |  |  |
| hsa-mir-199a-3p(hsa-mir-199a-1) | \_ | 174869 | 7614 | 46 | 22 | 7213 |  | 2 | 41 | 106 | 90 | 74 | 52 | 91 | 46 | 80 | 134 | 83 | 68 | 106 | 103 | 119 | 125 | 91 | 41 | 129 | 44 | 57 | 155 | 69 | 2922 | 2385 |  |  |  |
| hsa-mir-199a-3p(hsa-mir-199a-2) | \_ | 174864 | 7627 | 69 | 22 | 7226 |  | 2 | 41 | 106 | 90 | 74 | 52 | 91 | 46 | 80 | 134 | 83 | 68 | 106 | 103 | 119 | 125 | 91 | 41 | 129 | 44 | 57 | 155 | 69 | 2922 | 2398 |  |  |  |
| hsa-mir-199a-3p(hsa-mir-199b) | \_ | 174764 | 7614 | 64 | 22 | 7213 |  | 2 | 41 | 106 | 90 | 74 | 52 | 91 | 46 | 80 | 134 | 83 | 68 | 106 | 103 | 119 | 125 | 91 | 41 | 129 | 44 | 57 | 155 | 69 | 2922 | 2385 |  |  |  |
| hsa-mir-199b-3p(hsa-mir-199a-1) | \_ | 174869 | 7614 | 46 | 22 | 7213 |  | 2 | 41 | 106 | 90 | 74 | 52 | 91 | 46 | 80 | 134 | 83 | 68 | 106 | 103 | 119 | 125 | 91 | 41 | 129 | 44 | 57 | 155 | 69 | 2922 | 2385 |  |  |  |
| hsa-mir-199b-3p(hsa-mir-199a-2) | \_ | 174864 | 7627 | 69 | 22 | 7226 |  | 2 | 41 | 106 | 90 | 74 | 52 | 91 | 46 | 80 | 134 | 83 | 68 | 106 | 103 | 119 | 125 | 91 | 41 | 129 | 44 | 57 | 155 | 69 | 2922 | 2398 |  |  |  |
| hsa-mir-199b-3p(hsa-mir-199b) | \_ | 174764 | 7614 | 64 | 22 | 7213 |  | 2 | 41 | 106 | 90 | 74 | 52 | 91 | 46 | 80 | 134 | 83 | 68 | 106 | 103 | 119 | 125 | 91 | 41 | 129 | 44 | 57 | 155 | 69 | 2922 | 2385 |  |  |  |
| hsa-mir-20a-5p(hsa-mir-20a) | \_ | 264 | 22 | 7 | 23 | 22 |  |  |  |  |  |  |  |  |  |  |  |  |  |  |  |  |  |  |  |  |  |  |  |  |  | 22 |  |  |  |
| hsa-mir-21-3p(hsa-mir-21) | \_ | 103661 | 27 | 45 | 21 | 27 |  |  |  |  |  |  |  |  |  |  |  |  |  |  |  |  |  |  |  |  |  |  |  |  | 27 |  |  |  |  |
| hsa-mir-21-5p(hsa-mir-21) | \_ | 103661 | 3339 | 7 | 22 | 3203 |  |  | 32 | 149 | 65 | 71 | 244 | 75 | 54 | 89 | 102 | 292 | 135 | 102 | 69 | 42 | 84 | 87 | 91 | 171 | 69 | 128 | 71 | 74 | 98 | 752 | 57 |  |  |
| hsa-mir-210(hsa-mir-210) | \_ | 82 | 5 | 65 | 22 | 5 |  |  |  |  |  |  |  |  |  |  |  |  |  |  |  |  |  |  |  |  |  |  |  |  |  | 5 |  |  |  |
| hsa-mir-2110(hsa-mir-2110) | \_ | 84 | 5 | 7 | 22 | 5 |  |  |  |  |  |  |  |  |  |  |  |  |  |  |  |  |  |  |  |  |  |  |  |  |  | 5 |  |  |  |
| hsa-mir-215(hsa-mir-215) | \_ | 24 | 11 | 26 | 21 | 11 |  |  |  | 6 |  |  |  |  |  |  |  |  |  |  |  |  |  |  |  |  |  |  | 5 |  |  |  |  |  |  |
| hsa-mir-22-3p(hsa-mir-22) | \_ | 2864 | 199 | 52 | 22 | 241 |  |  |  |  |  |  | 6 |  |  | 40 | 61 |  | 31 |  |  | 6 |  |  |  |  |  | 12 |  | 49 | 6 | 30 |  |  |  |
| hsa-mir-22-5p(hsa-mir-22) | \_ | 2864 | 9 | 14 | 22 | 9 |  |  |  |  |  |  |  |  |  |  |  |  |  |  |  |  |  |  |  |  |  |  |  | 9 |  |  |  |  |  |
| hsa-mir-221-3p(hsa-mir-221) | \_ | 54800 | 4172 | 64 | 23 | 4333 |  |  |  | 82 | 70 | 48 | 24 | 53 | 43 | 70 | 23 | 29 | 24 | 50 | 87 | 45 | 22 | 46 | 37 | 93 | 14 | 17 | 440 | 280 | 1558 | 787 | 391 |  |  |
| hsa-mir-222-3p(hsa-mir-222) | \_ | 3140 | 107 | 68 | 21 | 120 |  |  |  |  |  |  |  |  |  |  |  |  |  |  |  |  |  |  |  |  |  |  |  | 36 | 84 |  |  |  |  |
| hsa-mir-223-3p(hsa-mir-223) | \_ | 31613 | 5503 | 67 | 22 | 5497 |  |  |  | 12 | 56 | 13 | 85 | 12 | 10 |  | 9 | 10 | 9 | 19 | 39 | 35 | 14 | 9 | 6 | 41 | 187 | 316 | 593 | 429 | 139 | 3231 | 223 |  |  |
| hsa-mir-2355-5p(hsa-mir-2355) | \_ | 555 | 14 | 10 | 21 | 14 |  |  |  |  |  |  | 9 | 5 |  |  |  |  |  |  |  |  |  |  |  |  |  |  |  |  |  |  |  |  |  |
| hsa-mir-2392(hsa-mir-2392) | \_ |  | 12265 | 60 | 20 | 24484 |  |  |  |  |  |  |  |  |  |  |  |  | 5856 | 6378 | 6 |  |  | 5860 | 6384 |  |  |  |  |  |  |  |  |  |  |
| hsa-mir-23a-3p(hsa-mir-23a) | \_ | 10109 | 574 | 44 | 21 | 547 |  |  |  |  |  | 25 |  | 8 | 6 |  |  | 5 | 71 | 26 |  |  |  |  | 6 | 12 |  | 6 | 201 | 159 | 6 | 16 |  |  |  |
| hsa-mir-23b-3p(hsa-mir-23b) | \_ | 1811 | 64 | 57 | 21 | 37 |  |  |  |  |  |  |  |  |  |  |  |  | 7 |  |  |  |  |  |  |  |  | 6 | 14 | 10 |  |  |  |  |  |
| hsa-mir-24-3p(hsa-mir-24-1) | \_ | 9450 | 5565 | 43 | 22 | 5687 |  |  | 8 | 19 | 7 | 11 | 10 |  | 23 | 6 | 10 | 13 | 13 | 20 | 10 |  | 16 | 5 | 2 | 36 | 9 | 7 | 17 | 44 | 186 | 5206 | 9 |  |  |
| hsa-mir-24-3p(hsa-mir-24-2) | \_ | 9487 | 5565 | 49 | 22 | 5687 |  |  | 8 | 19 | 7 | 11 | 10 |  | 23 | 6 | 10 | 13 | 13 | 20 | 10 |  | 16 | 5 | 2 | 36 | 9 | 7 | 17 | 44 | 186 | 5206 | 9 |  |  |
| hsa-mir-25-3p(hsa-mir-25) | \_ | 60490 | 5600 | 51 | 22 | 5496 |  |  |  | 170 | 46 | 36 | 64 | 80 | 171 | 92 | 189 | 28 | 34 | 43 | 49 | 147 | 44 | 103 | 58 | 79 | 353 | 74 | 29 | 161 | 2663 | 783 |  |  |  |
| hsa-mir-26a-5p(hsa-mir-26a-1) | \_ | 15995 | 4269 | 9 | 22 | 4248 |  |  | 447 | 14 | 11 | 55 | 15 | 5 | 8 | 14 | 14 | 4 | 10 | 70 | 67 | 10 | 6 | 4 | 11 | 45 | 7 | 6 | 19 | 145 | 125 | 3136 |  |  |  |
| hsa-mir-26a-5p(hsa-mir-26a-2) | \_ | 15982 | 4269 | 13 | 22 | 4248 |  |  | 447 | 14 | 11 | 55 | 15 | 5 | 8 | 14 | 14 | 4 | 10 | 70 | 67 | 10 | 6 | 4 | 11 | 45 | 7 | 6 | 19 | 145 | 125 | 3136 |  |  |  |
| hsa-mir-26b-5p(hsa-mir-26b) | \_ | 50080 | 1546 | 11 | 21 | 1476 |  |  |  | 56 | 11 | 98 | 66 | 53 | 13 | 38 | 21 | 27 | 17 | 142 | 97 | 56 | 20 | 15 | 46 | 74 | 41 | 20 | 122 | 213 | 170 | 60 |  |  |  |
| hsa-mir-27a-3p(hsa-mir-27a) | \_ | 9647 | 947 | 50 | 21 | 1007 |  |  |  |  | 11 | 25 | 7 | 8 | 8 |  | 13 | 6 |  | 20 |  |  |  |  | 28 | 7 | 20 | 4 | 19 | 711 | 120 |  |  |  |  |
| hsa-mir-27b-3p(hsa-mir-27b) | \_ | 7126 | 358 | 60 | 21 | 376 |  |  |  |  |  | 11 |  | 16 |  |  |  | 6 |  |  |  |  |  |  | 5 |  | 20 | 55 | 17 | 157 | 74 | 15 |  |  |  |
| hsa-mir-28-3p(hsa-mir-28) | \_ | 1543 | 70 | 53 | 22 | 70 |  |  |  |  |  |  |  |  |  |  |  |  |  |  |  |  |  |  |  |  | 5 |  |  |  |  | 57 | 8 |  |  |
| hsa-mir-28-5p(hsa-mir-28) | \_ | 1543 | 13 | 13 | 22 | 13 |  |  |  |  |  |  |  |  |  |  |  |  |  |  |  |  |  |  |  |  |  |  |  |  | 8 | 5 |  |  |  |
| hsa-mir-29a-3p(hsa-mir-29a) | \_ | 12800 | 476 | 41 | 22 | 481 |  |  |  | 15 | 7 |  | 13 | 6 | 74 | 64 |  | 5 |  | 7 |  | 6 |  |  |  | 25 | 9 |  |  | 6 | 207 | 37 |  |  |  |
| hsa-mir-29c-3p(hsa-mir-29c) | \_ | 2366 | 83 | 53 | 22 | 58 |  |  |  |  |  |  |  |  | 10 |  |  |  |  |  |  |  |  |  |  |  |  |  |  |  | 41 |  | 7 |  |  |
| hsa-mir-30a-3p(hsa-mir-30a) | \_ | 493 | 28 | 46 | 22 | 28 |  |  |  |  |  |  |  |  |  |  |  |  |  |  |  |  |  |  |  |  | 7 |  |  |  | 21 |  |  |  |  |
| hsa-mir-30a-5p(hsa-mir-30a) | \_ | 493 | 9 | 5 | 22 | 9 |  |  |  |  |  |  |  |  |  |  |  |  |  |  |  |  |  |  |  |  |  |  |  |  |  | 9 |  |  |  |
| hsa-mir-30b-3p(hsa-mir-30b) | \_ | 332 | 5 | 54 | 22 | 5 |  |  |  |  |  |  |  |  |  |  |  |  |  |  |  |  |  |  |  |  |  |  |  |  |  | 5 |  |  |  |
| hsa-mir-30b-5p(hsa-mir-30b) | \_ | 332 | 16 | 16 | 22 | 16 |  |  |  |  |  |  |  |  |  |  |  |  |  |  |  |  |  |  |  |  |  |  |  |  |  | 16 |  |  |  |
| hsa-mir-30d-5p(hsa-mir-30d) | \_ | 12923 | 435 | 5 | 22 | 435 |  |  |  | 6 |  |  |  |  |  | 5 |  |  | 32 | 82 |  | 15 |  |  |  |  |  |  |  |  | 59 | 236 |  |  |  |
| hsa-mir-30e-3p(hsa-mir-30e) | \_ | 3731 | 966 | 58 | 22 | 983 |  |  |  | 13 | 29 |  |  |  |  |  |  |  |  |  |  |  |  |  |  |  |  | 5 |  | 7 | 798 | 131 |  |  |  |
| hsa-mir-30e-5p(hsa-mir-30e) | \_ | 3731 | 46 | 16 | 22 | 46 |  |  |  |  |  |  |  |  |  |  |  |  |  | 6 | 15 |  |  |  |  |  |  |  |  |  |  | 20 | 5 |  |  |
| hsa-mir-3120-3p(hsa-mir-3120) | \_ | 149 | 248 | 50 | 21 | 248 |  |  |  |  |  |  |  |  |  |  |  |  |  |  |  |  |  |  |  |  |  |  |  |  | 248 |  |  |  |  |
| hsa-mir-3123(hsa-mir-3123) | \_ | 43 | 694 | 48 | 17 | 1151 |  |  |  |  |  |  |  |  |  |  |  | 457 |  |  |  |  |  |  | 694 |  |  |  |  |  |  |  |  |  |  |
| hsa-mir-3124-5p(hsa-mir-3124) | \_ | 21 | 6 | 6 | 21 | 6 |  |  |  |  |  |  |  |  |  |  |  |  |  |  |  |  |  |  |  |  |  |  |  |  | 6 |  |  |  |  |
| hsa-mir-3135b(hsa-mir-3135b) | \_ |  | 251 | 6 | 22 | 502 |  |  |  |  |  |  |  |  |  | 251 | 251 |  |  |  |  |  |  |  |  |  |  |  |  |  |  |  |  |  |  |
| hsa-mir-3141(hsa-mir-3141) | \_ |  | 10 | 9 | 19 | 20 |  |  |  | 10 |  |  |  |  |  |  |  |  |  |  |  | 10 |  |  |  |  |  |  |  |  |  |  |  |  |  |
| hsa-mir-3168(hsa-mir-3168) | \_ | 178 | 5715 | 8 | 17 | 10403 |  |  | 627 | 12 | 6 |  |  |  |  |  |  |  |  |  |  |  | 5715 | 11 | 75 | 76 | 3830 | 51 |  |  |  |  |  |  |  |
| hsa-mir-3177-3p(hsa-mir-3177) | \_ | 20 | 5 | 53 | 21 | 5 |  |  |  |  |  |  |  |  |  |  |  |  |  |  |  |  |  |  |  |  |  |  |  |  | 5 |  |  |  |  |
| hsa-mir-3182(hsa-mir-3182) | \_ | 30 | 177 | 3 | 17 | 354 |  |  |  |  | 10 |  |  |  | 10 |  |  |  |  |  |  |  |  |  |  | 167 | 167 |  |  |  |  |  |  |  |  |
| hsa-mir-32-5p(hsa-mir-32) | \_ | 56 | 5 | 5 | 22 | 5 |  |  |  |  |  |  |  |  |  |  |  |  |  |  |  |  |  |  |  |  |  |  |  |  |  | 5 |  |  |  |
| hsa-mir-320a(hsa-mir-320a) | \_ | 40758 | 6755 | 47 | 22 | 6876 |  |  | 9 | 41 | 19 | 31 | 29 | 31 | 57 | 13 | 50 | 35 | 23 | 24 | 24 | 16 | 42 | 27 | 44 | 46 | 35 | 34 | 104 | 9 | 3471 | 2102 | 560 |  |  |
| hsa-mir-320b(hsa-mir-320b-1) | \_ | 169 | 45 | 38 | 22 | 56 |  |  |  |  |  |  |  |  |  |  |  |  |  |  |  |  |  |  |  |  |  | 2 | 18 | 9 | 23 | 4 |  |  |  |
| hsa-mir-320b(hsa-mir-320b-2) | \_ | 169 | 45 | 71 | 22 | 56 |  |  |  |  |  |  |  |  |  |  |  |  |  |  |  |  |  |  |  |  |  | 2 | 18 | 9 | 23 | 4 |  |  |  |
| hsa-mir-320c(hsa-mir-320c-1) | \_ | 35 | 104 | 49 | 20 | 106 |  |  |  |  |  |  |  |  |  |  |  |  |  |  |  |  |  |  |  |  |  | 7 | 83 | 16 |  |  |  |  |  |
| hsa-mir-320c(hsa-mir-320c-2) | \_ | 35 | 73 | 30 | 20 | 73 |  |  |  |  |  |  |  |  |  |  |  |  |  |  |  |  |  |  |  |  |  | 5 | 68 |  |  |  |  |  |  |
| hsa-mir-320d(hsa-mir-320d-1) | \_ | 11 | 11 | 29 | 19 | 11 |  |  |  |  |  |  |  |  |  |  |  |  |  |  |  |  |  |  |  |  |  |  | 11 |  |  |  |  |  |  |
| hsa-mir-320d(hsa-mir-320d-2) | \_ | 11 | 11 | 29 | 19 | 11 |  |  |  |  |  |  |  |  |  |  |  |  |  |  |  |  |  |  |  |  |  |  | 11 |  |  |  |  |  |  |
| hsa-mir-323a-3p(hsa-mir-323a) | \_ | 1382 | 51 | 50 | 21 | 51 |  |  |  |  |  |  |  |  |  |  |  |  |  |  |  |  |  |  |  |  | 6 |  |  | 45 |  |  |  |  |  |
| hsa-mir-323b-3p(hsa-mir-323b) | \_ | 1561 | 107 | 50 | 22 | 110 |  |  |  | 17 | 17 |  |  |  |  |  |  |  |  |  |  |  |  |  | 5 |  |  | 6 |  | 49 | 8 | 8 |  |  |  |
| hsa-mir-326(hsa-mir-326) | \_ | 9 | 201 | 59 | 20 | 201 |  |  |  |  |  |  |  |  |  |  |  |  |  |  |  |  |  |  |  |  |  |  |  | 201 |  |  |  |  |  |
| hsa-mir-328(hsa-mir-328) | \_ | 154 | 11 | 47 | 22 | 11 |  |  |  |  |  |  |  |  |  |  |  |  |  |  |  |  |  |  |  |  |  |  |  |  |  | 11 |  |  |  |
| hsa-mir-329(hsa-mir-329-1) | \_ | 38 | 15 | 49 | 22 | 15 |  |  |  |  |  |  |  |  |  |  |  |  |  |  |  |  |  |  |  |  |  |  |  | 2 | 13 |  |  |  |  |
| hsa-mir-329(hsa-mir-329-2) | \_ | 38 | 15 | 51 | 22 | 15 |  |  |  |  |  |  |  |  |  |  |  |  |  |  |  |  |  |  |  |  |  |  |  | 2 | 13 |  |  |  |  |
| hsa-mir-330-3p(hsa-mir-330) | \_ | 4100 | 370 | 56 | 23 | 376 |  |  |  |  |  |  |  |  |  |  |  |  |  | 7 |  |  |  |  |  |  |  |  |  | 6 | 30 | 217 | 64 | 52 |  |
| hsa-mir-330-5p(hsa-mir-330) | \_ | 4100 | 6 | 17 | 22 | 6 |  |  |  |  |  |  |  |  |  |  |  |  |  |  |  |  |  |  |  |  |  |  |  |  | 6 |  |  |  |  |
| hsa-mir-331-3p(hsa-mir-331) | \_ | 295 | 64 | 60 | 21 | 64 |  |  |  |  |  |  |  |  |  |  |  |  |  |  |  |  |  |  |  |  |  |  |  | 9 | 55 |  |  |  |  |
| hsa-mir-335-3p(hsa-mir-335) | \_ | 1766 | 5 | 51 | 22 | 5 |  |  |  |  |  |  |  |  |  |  |  |  |  |  |  |  |  |  |  |  |  |  |  |  | 5 |  |  |  |  |
| hsa-mir-335-5p(hsa-mir-335) | \_ | 1766 | 9 | 15 | 23 | 9 |  |  |  |  |  |  |  |  |  |  |  |  |  |  |  |  |  |  |  |  |  |  |  |  |  | 9 |  |  |  |
| hsa-mir-339-3p(hsa-mir-339) | \_ | 1956 | 417 | 49 | 23 | 458 |  |  |  |  |  |  |  |  |  |  |  |  |  |  |  |  |  |  |  |  |  |  |  |  | 72 | 205 | 170 | 11 |  |
| hsa-mir-339-5p(hsa-mir-339) | \_ | 1956 | 120 | 14 | 23 | 127 |  |  |  |  |  |  |  |  |  |  |  |  |  |  |  |  |  |  |  |  |  |  |  |  | 100 | 15 | 12 |  |  |
| hsa-mir-33a-5p(hsa-mir-33a) | \_ | 3956 | 256 | 5 | 21 | 256 |  |  |  |  |  |  |  |  |  |  |  |  |  |  |  |  |  |  |  |  |  |  | 18 | 30 | 208 |  |  |  |  |
| hsa-mir-340-5p(hsa-mir-340) | \_ | 24109 | 1130 | 15 | 22 | 1105 |  |  | 18 | 8 | 10 | 21 |  | 10 | 5 | 8 | 14 | 20 | 10 | 8 | 6 | 36 | 16 |  | 19 | 37 | 5 | 5 | 31 | 76 | 291 | 451 |  |  |  |
| hsa-mir-342-3p(hsa-mir-342) | \_ | 186 | 5 | 60 | 23 | 5 |  |  |  |  |  |  |  |  |  |  |  |  |  |  |  |  |  |  |  |  |  |  |  |  |  |  |  | 5 |  |
| hsa-mir-345-5p(hsa-mir-345) | \_ | 21 | 161 | 17 | 22 | 200 |  |  |  |  |  |  |  |  |  |  |  |  |  |  |  |  |  |  |  |  |  |  |  |  | 68 | 132 |  |  |  |
| hsa-mir-361-5p(hsa-mir-361) | \_ | 145 | 45 | 5 | 22 | 45 |  |  |  |  |  |  |  |  |  |  |  |  |  |  |  |  |  |  |  |  |  |  |  |  | 39 | 6 |  |  |  |
| hsa-mir-3615(hsa-mir-3615) | \_ | 148 | 41 | 50 | 21 | 67 |  |  |  |  |  |  |  |  |  |  |  |  |  |  |  |  |  |  |  |  |  |  |  | 26 | 41 |  |  |  |  |
| hsa-mir-363-3p(hsa-mir-363) | \_ | 535 | 240 | 49 | 22 | 254 |  |  |  |  |  |  |  |  |  |  |  |  |  |  |  |  |  |  |  |  |  |  |  | 12 |  | 228 | 14 |  |  |
| hsa-mir-3656(hsa-mir-3656) | \_ | 52 | 100 | 48 | 17 | 100 |  |  | 10 |  |  |  |  |  |  |  |  |  |  |  |  |  |  |  |  |  |  | 90 |  |  |  |  |  |  |  |
| hsa-mir-3676-5p(hsa-mir-3676) | \_ | 28 | 12 | 26 | 15 | 12 |  |  |  |  |  |  |  |  |  |  |  |  |  |  |  |  |  |  | 12 |  |  |  |  |  |  |  |  |  |  |
| hsa-mir-3677-3p(hsa-mir-3677) | \_ | 29 | 5 | 38 | 22 | 5 |  |  |  |  |  |  |  |  |  |  |  |  |  |  |  |  |  |  |  |  |  |  |  |  | 5 |  |  |  |  |
| hsa-mir-369-3p(hsa-mir-369) | \_ | 1780 | 6 | 43 | 21 | 6 |  |  |  |  |  |  |  |  |  |  |  |  |  |  |  |  |  |  |  |  |  |  |  |  | 6 |  |  |  |  |
| hsa-mir-369-5p(hsa-mir-369) | \_ | 1780 | 5 | 8 | 22 | 5 |  |  |  |  |  |  |  |  |  |  |  |  |  |  |  |  |  |  |  |  |  |  |  | 5 |  |  |  |  |  |
| hsa-mir-370(hsa-mir-370) | \_ | 18 | 22 | 47 | 22 | 22 |  |  |  |  |  |  |  |  |  |  |  |  |  |  |  |  |  |  |  |  |  |  |  |  | 6 | 16 |  |  |  |
| hsa-mir-374a-5p(hsa-mir-374a) | \_ | 1787 | 48 | 11 | 22 | 48 |  |  |  |  |  |  |  |  |  |  |  |  |  |  |  |  |  |  |  |  |  |  |  |  |  | 48 |  |  |  |
| hsa-mir-374b-5p(hsa-mir-374b) | \_ | 608 | 30 | 10 | 22 | 30 |  |  |  |  |  |  |  |  |  |  |  |  |  |  |  |  |  |  |  |  |  |  |  |  |  | 30 |  |  |  |
| hsa-mir-376a-3p(hsa-mir-376a-1) | \_ | 15 | 70 | 43 | 21 | 70 |  |  |  |  |  |  |  |  | 70 |  |  |  |  |  |  |  |  |  |  |  |  |  |  |  |  |  |  |  |  |
| hsa-mir-376a-3p(hsa-mir-376a-2) | \_ | 15 | 70 | 49 | 21 | 70 |  |  |  |  |  |  |  |  | 70 |  |  |  |  |  |  |  |  |  |  |  |  |  |  |  |  |  |  |  |  |
| hsa-mir-376b(hsa-mir-376b) | \_ | 72 | 6 | 61 | 22 | 6 |  |  |  |  |  |  |  |  | 6 |  |  |  |  |  |  |  |  |  |  |  |  |  |  |  |  |  |  |  |  |
| hsa-mir-376c(hsa-mir-376c) | \_ | 270 | 49 | 42 | 21 | 49 |  |  |  |  |  |  |  |  | 49 |  |  |  |  |  |  |  |  |  |  |  |  |  |  |  |  |  |  |  |  |
| hsa-mir-378a-3p(hsa-mir-378a) | \_ | 1642 | 419 | 42 | 21 | 419 |  |  |  |  |  |  |  |  |  |  |  |  |  |  |  |  |  |  |  |  |  |  |  | 18 | 343 | 58 |  |  |  |
| hsa-mir-378c(hsa-mir-378c) | \_ | 259 | 6 | 10 | 25 | 6 |  |  |  |  |  |  |  |  |  |  |  |  |  |  |  |  |  |  |  |  |  |  |  |  |  |  | 6 |  |  |
| hsa-mir-378f(hsa-mir-378f) | \_ | 1 | 18 | 51 | 20 | 18 |  |  |  |  |  |  |  |  |  |  |  |  |  |  |  |  | 18 |  |  |  |  |  |  |  |  |  |  |  |  |
| hsa-mir-378i(hsa-mir-378i) | \_ |  | 62 | 6 | 21 | 62 |  |  |  |  |  |  |  |  |  |  |  | 62 |  |  |  |  |  |  |  |  |  |  |  |  |  |  |  |  |  |
| hsa-mir-379-3p(hsa-mir-379) | \_ | 989 | 9 | 43 | 22 | 14 |  |  |  |  |  |  |  |  |  |  |  |  | 9 |  |  |  |  |  |  |  |  |  |  | 5 |  |  |  |  |  |
| hsa-mir-379-5p(hsa-mir-379) | \_ | 989 | 212 | 5 | 21 | 212 |  |  |  |  |  |  |  | 11 |  |  |  |  |  |  |  |  |  |  |  |  |  |  |  |  | 201 |  |  |  |  |
| hsa-mir-381(hsa-mir-381) | \_ | 78 | 5 | 48 | 22 | 5 |  |  |  |  |  |  | 5 |  |  |  |  |  |  |  |  |  |  |  |  |  |  |  |  |  |  |  |  |  |  |
| hsa-mir-382-5p(hsa-mir-382) | \_ | 1975 | 25 | 10 | 22 | 25 |  |  |  |  |  |  |  |  |  |  |  |  |  |  |  |  |  |  |  |  |  |  |  | 12 | 13 |  |  |  |  |
| hsa-mir-3928(hsa-mir-3928) | \_ | 76 | 59 | 36 | 22 | 59 |  |  |  |  |  |  |  |  |  |  |  |  |  |  |  |  |  |  |  |  |  |  |  |  | 11 | 48 |  |  |  |
| hsa-mir-409-3p(hsa-mir-409) | \_ | 854 | 20 | 46 | 22 | 20 |  |  |  |  |  |  |  |  |  |  |  |  |  |  |  |  |  |  |  |  | 9 | 11 |  |  |  |  |  |  |  |
| hsa-mir-410(hsa-mir-410) | \_ | 436 | 39 | 49 | 21 | 39 |  |  |  |  |  |  |  |  |  |  |  |  |  |  |  |  |  |  |  |  |  |  |  |  | 39 |  |  |  |  |
| hsa-mir-411-3p(hsa-mir-411) | \_ | 708 | 35 | 50 | 22 | 48 |  |  |  |  |  |  |  |  |  |  |  |  |  |  |  |  |  |  |  |  |  |  | 8 | 26 | 14 |  |  |  |  |
| hsa-mir-411-5p(hsa-mir-411) | \_ | 708 | 5 | 15 | 21 | 5 |  |  |  |  |  |  |  |  |  |  |  |  |  |  |  |  |  |  |  |  |  |  |  |  | 5 |  |  |  |  |
| hsa-mir-421(hsa-mir-421) | \_ | 146 | 13 | 47 | 23 | 13 |  |  |  |  |  |  |  |  |  |  |  |  |  |  |  |  |  |  |  |  |  |  |  |  |  |  | 13 |  |  |
| hsa-mir-423-3p(hsa-mir-423) | \_ | 43280 | 552 | 52 | 23 | 548 |  |  |  | 6 |  | 10 |  |  | 5 |  |  | 5 |  |  |  |  |  |  | 33 | 49 | 40 |  | 5 |  | 161 | 52 | 182 |  |  |
| hsa-mir-423-5p(hsa-mir-423) | \_ | 43280 | 1705 | 16 | 23 | 1901 |  |  |  | 28 | 20 | 27 | 22 | 47 | 16 | 26 | 25 | 32 |  | 9 | 5 | 27 | 8 | 47 | 51 | 85 | 16 | 79 | 76 | 242 | 347 | 518 | 143 | 5 |  |
| hsa-mir-424-3p(hsa-mir-424) | \_ | 452 | 10 | 47 | 21 | 10 |  |  |  |  |  |  |  |  |  |  |  |  |  |  |  |  |  |  |  |  |  |  |  |  | 10 |  |  |  |  |
| hsa-mir-425-3p(hsa-mir-425) | \_ | 1746 | 226 | 54 | 22 | 232 |  |  |  |  |  |  |  |  |  |  |  |  |  |  |  |  |  |  |  |  |  |  |  | 130 | 63 | 39 |  |  |  |
| hsa-mir-425-5p(hsa-mir-425) | \_ | 1746 | 8 | 13 | 23 | 8 |  |  |  |  |  |  |  |  |  |  |  |  |  |  |  |  |  |  |  |  |  |  |  |  |  | 8 |  |  |  |
| hsa-mir-4286(hsa-mir-4286) | \_ | 1002 | 31 | 10 | 17 | 31 |  |  |  |  |  |  | 10 |  |  |  |  | 8 |  |  |  |  |  |  |  |  | 13 |  |  |  |  |  |  |  |  |
| hsa-mir-4306(hsa-mir-4306) | \_ | 109 | 26 | 64 | 17 | 26 |  |  |  |  |  |  |  |  |  |  |  |  |  |  |  |  |  |  |  | 26 |  |  |  |  |  |  |  |  |  |
| hsa-mir-432-5p(hsa-mir-432) | \_ | 1989 | 575 | 13 | 23 | 586 |  |  |  |  |  |  |  |  |  |  |  |  |  |  |  |  |  |  |  |  |  |  |  |  | 61 | 517 | 8 |  |  |
| hsa-mir-433(hsa-mir-433) | \_ | 1840 | 80 | 63 | 22 | 80 |  |  |  |  |  |  |  |  |  |  |  |  |  |  |  |  |  | 6 |  |  |  |  |  | 13 | 40 | 21 |  |  |  |
| hsa-mir-4429(hsa-mir-4429) | \_ |  | 19 | 6 | 20 | 19 |  |  |  |  |  |  |  |  |  |  |  |  |  | 19 |  |  |  |  |  |  |  |  |  |  |  |  |  |  |  |
| hsa-mir-4433-3p(hsa-mir-4433) | \_ | 41 | 8754 | 50 | 21 | 17416 |  |  |  |  |  |  |  |  |  |  |  | 94 |  |  |  |  | 8655 |  |  |  |  |  | 10 | 8657 |  |  |  |  |  |
| hsa-mir-4433-5p(hsa-mir-4433) | \_ | 41 | 23 | 11 | 21 | 23 |  |  |  | 23 |  |  |  |  |  |  |  |  |  |  |  |  |  |  |  |  |  |  |  |  |  |  |  |  |  |
| hsa-mir-4435(hsa-mir-4435-1) | \_ | 37 | 4 | 9 | 22 | 4 |  |  |  |  |  |  |  |  |  |  |  |  |  |  |  |  |  |  |  |  |  |  |  |  |  | 4 |  |  |  |
| hsa-mir-4435(hsa-mir-4435-2) | \_ | 37 | 4 | 4 | 22 | 4 |  |  |  |  |  |  |  |  |  |  |  |  |  |  |  |  |  |  |  |  |  |  |  |  |  | 4 |  |  |  |
| hsa-mir-4443(hsa-mir-4443) | \_ | 71 | 16 | 8 | 17 | 26 |  | 10 | 16 |  |  |  |  |  |  |  |  |  |  |  |  |  |  |  |  |  |  |  |  |  |  |  |  |  |  |
| hsa-mir-4446-3p(hsa-mir-4446) | \_ | 678 | 163 | 42 | 22 | 173 |  |  |  |  |  |  |  |  |  |  |  |  |  |  |  | 5 |  |  |  |  |  |  |  | 52 | 72 | 44 |  |  |  |
| hsa-mir-4447(hsa-mir-4447) | \_ |  | 37 | 62 | 17 | 74 |  |  |  |  |  |  |  | 37 |  |  | 37 |  |  |  |  |  |  |  |  |  |  |  |  |  |  |  |  |  |  |
| hsa-mir-4448(hsa-mir-4448) | \_ | 21 | 87 | 60 | 20 | 136 |  |  |  |  |  |  |  |  | 87 |  |  | 10 |  |  |  |  |  |  |  |  |  |  |  | 39 |  |  |  |  |  |
| hsa-mir-4454(hsa-mir-4454) | \_ | 38 | 102 | 2 | 20 | 184 |  | 17 |  |  | 31 |  |  |  |  |  |  |  |  |  |  |  |  |  |  |  |  |  |  | 65 | 71 |  |  |  |  |
| hsa-mir-4455(hsa-mir-4455) | \_ |  | 126 | 3 | 17 | 173 |  |  | 22 |  |  |  |  |  |  |  |  | 126 |  |  |  |  |  |  |  |  | 25 |  |  |  |  |  |  |  |  |
| hsa-mir-4466(hsa-mir-4466) | \_ |  | 11 | 4 | 18 | 11 |  |  |  |  |  |  |  |  |  |  |  |  | 11 |  |  |  |  |  |  |  |  |  |  |  |  |  |  |  |  |
| hsa-mir-4483(hsa-mir-4483) | \_ |  | 28 | 38 | 17 | 56 |  |  |  |  |  | 28 |  |  |  |  |  |  |  |  |  |  |  |  |  | 28 |  |  |  |  |  |  |  |  |  |
| hsa-mir-4497(hsa-mir-4497) | \_ |  | 46 | 2 | 17 | 92 |  |  |  |  |  |  |  |  |  |  |  |  |  |  |  |  |  |  |  | 46 | 46 |  |  |  |  |  |  |  |  |
| hsa-mir-4508(hsa-mir-4508) | \_ | 1699 | 3707 | 8 | 17 | 3783 |  | 3701 |  |  |  |  |  |  |  |  |  |  |  |  | 5 |  |  | 43 | 34 |  |  |  |  |  |  |  |  |  |  |
| hsa-mir-4510(hsa-mir-4510) | \_ |  | 1 | 7 | 22 | 2 |  |  |  |  |  |  |  |  |  |  |  |  |  |  |  | 1 |  |  |  |  |  | 1 |  |  |  |  |  |  |  |
| hsa-mir-4516(hsa-mir-4516) | \_ | 51 | 31 | 1 | 17 | 38 |  |  | 7 | 7 |  |  |  |  |  |  |  |  |  |  |  |  |  |  |  |  | 24 |  |  |  |  |  |  |  |  |
| hsa-mir-4531(hsa-mir-4531) | \_ |  | 86 | 27 | 17 | 107 |  |  |  |  |  |  |  |  | 86 |  |  |  |  |  |  |  |  |  |  |  | 21 |  |  |  |  |  |  |  |  |
| hsa-mir-4750(hsa-mir-4750) | \_ |  | 12 | 2 | 22 | 12 |  |  |  |  |  |  |  |  |  |  |  |  |  |  |  |  |  |  |  |  |  |  |  |  |  | 12 |  |  |  |
| hsa-mir-4770(hsa-mir-4770) | \_ |  | 6 | 39 | 18 | 12 |  |  |  |  |  |  |  |  |  |  |  | 6 | 6 |  |  |  |  |  |  |  |  |  |  |  |  |  |  |  |  |
| hsa-mir-484(hsa-mir-484) | \_ | 343 | 58 | 7 | 22 | 58 |  |  |  |  |  |  |  |  |  |  |  |  |  |  |  |  |  |  |  |  |  |  |  | 8 | 12 | 38 |  |  |  |
| hsa-mir-485-3p(hsa-mir-485) | \_ | 2597 | 158 | 45 | 22 | 137 |  |  |  |  |  |  |  |  |  |  |  |  |  |  |  |  | 9 | 10 | 25 | 14 |  | 7 |  |  | 41 | 31 |  |  |  |
| hsa-mir-485-5p(hsa-mir-485) | \_ | 2597 | 31 | 8 | 22 | 31 |  |  |  |  |  |  |  |  |  |  |  |  |  |  |  | 15 |  |  |  |  |  |  |  |  | 9 | 7 |  |  |  |
| hsa-mir-486-3p(hsa-mir-486) | \_ | 5687 | 471 | 45 | 21 | 471 |  |  |  |  |  |  |  |  |  |  |  |  |  | 6 |  |  | 13 |  |  | 7 |  |  |  | 29 | 416 |  |  |  |  |
| hsa-mir-486-5p(hsa-mir-486) | \_ | 5687 | 1482 | 3 | 22 | 1602 |  |  |  |  | 24 | 28 |  |  |  |  |  |  |  |  |  |  |  |  | 12 | 57 | 35 | 18 |  | 15 | 579 | 834 |  |  |  |
| hsa-mir-487b(hsa-mir-487b) | \_ | 511 | 5 | 50 | 22 | 5 |  |  |  |  |  |  |  |  |  |  |  |  |  |  |  |  |  |  |  |  |  |  |  |  |  | 5 |  |  |  |
| hsa-mir-493-3p(hsa-mir-493) | \_ | 306 | 32 | 56 | 22 | 32 |  |  |  |  |  |  |  |  |  |  |  |  |  |  |  |  |  |  |  |  |  |  |  |  |  | 32 |  |  |  |
| hsa-mir-495(hsa-mir-495) | \_ | 2826 | 63 | 49 | 22 | 63 |  |  |  |  |  |  |  |  |  |  |  |  |  |  |  | 6 |  |  |  |  |  |  | 10 |  |  | 47 |  |  |  |
| hsa-mir-496(hsa-mir-496) | \_ | 163 | 49 | 55 | 22 | 49 |  |  |  |  |  |  |  |  |  |  |  |  |  |  |  |  |  |  |  |  |  |  |  |  |  | 49 |  |  |  |
| hsa-mir-5010-5p(hsa-mir-5010) | \_ | 316 | 8 | 20 | 22 | 8 |  |  |  |  |  |  |  |  |  |  |  |  |  |  |  |  |  |  |  |  |  |  |  |  |  | 8 |  |  |  |
| hsa-mir-503(hsa-mir-503) | \_ | 607 | 132 | 5 | 23 | 149 |  |  |  |  |  |  |  |  |  |  |  |  |  |  |  |  |  |  |  |  |  |  |  | 63 |  | 86 |  |  |  |
| hsa-mir-532-5p(hsa-mir-532) | \_ | 650 | 14 | 19 | 22 | 14 |  |  |  |  |  |  |  |  |  |  |  |  |  |  |  |  |  |  |  |  |  |  |  |  |  | 14 |  |  |  |
| hsa-mir-543(hsa-mir-543) | \_ | 1768 | 321 | 46 | 22 | 321 |  |  |  |  |  |  |  |  |  |  |  |  |  |  |  |  |  |  |  |  |  |  | 11 |  |  | 310 |  |  |  |
| hsa-mir-548j(hsa-mir-548j) | \_ | 999 | 9 | 28 | 22 | 9 |  |  |  |  |  |  |  |  |  |  |  |  |  |  |  |  |  |  |  |  |  |  |  |  |  | 9 |  |  |  |
| hsa-mir-551b-3p(hsa-mir-551b) | \_ | 11 | 11 | 60 | 21 | 11 |  |  |  |  |  |  |  |  |  |  |  |  |  |  |  |  |  |  |  |  |  |  |  |  | 11 |  |  |  |  |
| hsa-mir-574-3p(hsa-mir-574) | \_ | 180 | 11 | 60 | 22 | 11 |  |  |  |  |  |  |  |  |  |  |  |  |  |  |  |  |  |  |  |  |  |  |  |  | 6 | 5 |  |  |  |
| hsa-mir-584-5p(hsa-mir-584) | \_ | 23138 | 3541 | 15 | 22 | 3485 |  |  |  | 23 | 10 | 26 | 14 | 18 | 7 | 40 | 18 | 8 | 15 | 85 | 90 | 14 | 20 | 19 |  | 94 | 38 | 11 | 62 | 1497 | 1232 | 144 |  |  |  |
| hsa-mir-589-5p(hsa-mir-589) | \_ | 187 | 34 | 23 | 22 | 54 |  |  |  |  |  |  |  |  |  |  |  |  |  |  |  |  |  |  |  |  |  |  |  |  | 34 | 20 |  |  |  |
| hsa-mir-598(hsa-mir-598) | \_ | 2478 | 39 | 60 | 22 | 39 |  |  |  |  |  |  | 5 |  | 14 |  |  |  |  |  |  |  |  |  |  |  |  |  |  |  | 20 |  |  |  |  |
| hsa-mir-625-3p(hsa-mir-625) | \_ | 515 | 5 | 51 | 22 | 5 |  |  |  |  |  |  |  |  |  |  |  |  |  |  |  |  |  |  |  |  |  |  |  |  |  | 5 |  |  |  |
| hsa-mir-652-3p(hsa-mir-652) | \_ | 466 | 713 | 60 | 21 | 723 |  |  |  |  |  |  |  |  |  |  |  | 5 |  |  |  |  |  |  |  | 5 |  |  |  |  | 713 |  |  |  |  |
| hsa-mir-654-3p(hsa-mir-654) | \_ | 530 | 55 | 50 | 22 | 60 |  |  |  |  |  |  |  |  |  |  |  |  |  |  |  |  |  |  |  |  |  | 10 |  | 40 | 10 |  |  |  |  |
| hsa-mir-671-3p(hsa-mir-671) | \_ | 39 | 17 | 67 | 21 | 17 |  |  |  |  |  |  |  |  |  |  |  |  |  |  |  |  |  |  |  |  |  |  |  | 17 |  |  |  |  |  |
| hsa-mir-720(hsa-mir-720) | \_ | 774 | 598 | 26 | 17 | 684 |  |  | 83 | 539 |  |  |  |  |  |  |  |  |  |  |  |  |  | 40 | 7 |  | 15 |  |  |  |  |  |  |  |  |
| hsa-mir-744-5p(hsa-mir-744) | \_ | 49263 | 14545 | 10 | 22 | 14717 |  |  | 18 | 79 | 62 | 63 | 88 | 84 | 27 | 31 | 46 | 33 | 65 | 73 | 16 | 11 | 53 | 17 | 49 | 127 | 158 | 54 | 40 | 923 | 863 | 11737 |  |  |  |
| hsa-mir-766-5p(hsa-mir-766) | \_ | 153 | 6 | 28 | 22 | 6 |  |  |  |  |  |  |  |  |  |  |  |  |  |  |  |  |  |  |  |  |  |  |  |  |  | 6 |  |  |  |
| hsa-mir-769-5p(hsa-mir-769) | \_ | 124 | 20 | 29 | 22 | 20 |  |  |  |  |  |  |  |  |  |  |  |  |  |  |  |  |  |  |  |  |  |  |  |  | 7 | 13 |  |  |  |
| hsa-mir-92a-3p(hsa-mir-92a-1) | \_ | 25340 | 2239 | 47 | 22 | 2128 |  |  | 11 | 34 | 19 | 12 | 19 | 41 | 75 | 38 | 93 | 10 | 29 | 30 | 19 | 15 | 208 | 144 | 28 | 39 | 72 | 40 | 12 | 79 | 235 | 823 | 3 |  |  |
| hsa-mir-92a-3p(hsa-mir-92a-2) | \_ | 24425 | 2210 | 47 | 22 | 2099 |  |  | 11 | 34 | 19 | 12 | 19 | 41 | 75 | 38 | 93 | 10 | 29 | 30 | 19 | 15 | 208 | 144 | 28 | 39 | 72 | 40 | 12 | 79 | 218 | 811 | 3 |  |  |
| hsa-mir-92b-3p(hsa-mir-92b) | \_ | 649 | 368 | 60 | 22 | 438 |  |  |  |  |  |  |  |  |  |  |  |  |  |  |  |  |  |  |  |  |  |  |  | 92 | 284 | 62 |  |  |  |
| hsa-mir-93-3p(hsa-mir-93) | \_ | 1165 | 5 | 49 | 22 | 5 |  |  |  |  |  |  |  |  |  |  |  |  |  |  |  |  |  |  |  |  |  |  |  |  |  | 5 |  |  |  |
| hsa-mir-93-5p(hsa-mir-93) | \_ | 1165 | 54 | 10 | 23 | 54 |  |  |  |  |  |  |  |  |  |  |  |  |  |  |  |  |  |  |  |  |  |  |  | 6 |  | 29 | 19 |  |  |
| hsa-mir-940(hsa-mir-940) | \_ |  | 5 | 59 | 21 | 5 |  |  |  |  |  |  |  |  |  |  |  |  |  |  |  |  |  |  |  |  |  |  |  |  | 5 |  |  |  |  |
| hsa-mir-98(hsa-mir-98) | \_ | 2325 | 90 | 21 | 22 | 91 |  |  |  |  |  |  |  |  |  |  |  |  |  | 25 |  |  |  |  |  |  |  | 25 | 1 |  | 16 | 24 |  |  |  |
| hsa-mir-99a-5p(hsa-mir-99a) | \_ | 147 | 25 | 12 | 22 | 25 |  |  |  |  |  |  |  |  |  |  |  |  |  |  |  |  |  |  |  |  |  |  |  |  |  | 25 |  |  |  |
| hsa-mir-99b-3p(hsa-mir-99b) | \_ | 2007 | 12 | 44 | 22 | 12 |  |  |  |  |  |  |  |  |  |  |  |  |  |  |  |  |  |  |  |  |  |  |  |  |  | 12 |  |  |  |
| hsa-mir-99b-5p(hsa-mir-99b) | \_ | 2007 | 617 | 6 | 22 | 625 |  |  |  |  |  | 6 |  | 10 |  |  |  |  |  |  |  |  |  |  |  |  |  |  |  |  | 51 | 558 |  |  |  |
| Sum(Avg) |  | 6083735 | 344905 | 8596 | 5503 | 365294 |  | 3882 | 3457 | 5552 | 3927 | 4850 | 2597 | 2482 | 5929 | 4576 | 4709 | 8275 | 9787 | 9720 | 3868 | 4441 | 17807 | 8829 | 12126 | 9044 | 8690 | 3751 | 6150 | 22351 | 59257 | 133386 | 5684 | 138 | 29 |

## Detected matches

|  |  |  |  |  |
| --- | --- | --- | --- | --- |
| microRNA | Exact | Loose | Total |  |
| hsa-let-7a-2-3p(hsa-let-7a-2) | 174459 | 0 | 174459 |
| hsa-let-7a-3p(hsa-let-7a-1) | 174438 | 3 | 174441 |
| hsa-let-7a-3p(hsa-let-7a-3) | 174518 | 3 | 174521 |
| hsa-let-7a-5p(hsa-let-7a-1) | 174438 | 9386 | 183824 |
| hsa-let-7a-5p(hsa-let-7a-2) | 174459 | 9446 | 183905 |
| hsa-let-7a-5p(hsa-let-7a-3) | 174518 | 9386 | 183904 |
| hsa-let-7b-3p(hsa-let-7b) | 47973 | 0 | 47973 |
| hsa-let-7b-5p(hsa-let-7b) | 47973 | 4596 | 52569 |
| hsa-let-7c(hsa-let-7c) | 4628 | 868 | 5496 |
| hsa-let-7d-3p(hsa-let-7d) | 98375 | 11 | 98386 |
| hsa-let-7d-5p(hsa-let-7d) | 98375 | 4673 | 103048 |
| hsa-let-7e-3p(hsa-let-7e) | 31101 | 0 | 31101 |
| hsa-let-7e-5p(hsa-let-7e) | 31101 | 2931 | 34032 |
| hsa-let-7f-1-3p(hsa-let-7f-1) | 599505 | 0 | 599505 |
| hsa-let-7f-2-3p(hsa-let-7f-2) | 617753 | 5 | 617758 |
| hsa-let-7f-5p(hsa-let-7f-1) | 599505 | 25610 | 625115 |
| hsa-let-7f-5p(hsa-let-7f-2) | 617753 | 25955 | 643708 |
| hsa-let-7g-3p(hsa-let-7g) | 96425 | 0 | 96425 |
| hsa-let-7g-5p(hsa-let-7g) | 96425 | 6266 | 102691 |
| hsa-let-7i-3p(hsa-let-7i) | 53344 | 7 | 53351 |
| hsa-let-7i-5p(hsa-let-7i) | 53344 | 7008 | 60352 |
| hsa-mir-1(hsa-mir-1-1) | 13241 | 368 | 13609 |
| hsa-mir-1(hsa-mir-1-2) | 13338 | 368 | 13706 |
| hsa-mir-100-3p(hsa-mir-100) | 39 | 0 | 39 |
| hsa-mir-100-5p(hsa-mir-100) | 39 | 0 | 39 |
| hsa-mir-101-3p(hsa-mir-101-1) | 15356 | 919 | 16275 |
| hsa-mir-101-3p(hsa-mir-101-2) | 15488 | 919 | 16407 |
| hsa-mir-101-5p(hsa-mir-101-1) | 15356 | 0 | 15356 |
| hsa-mir-103a-2-5p(hsa-mir-103a-2) | 162282 | 0 | 162282 |
| hsa-mir-103a-3p(hsa-mir-103a-1) | 159494 | 13593 | 173087 |
| hsa-mir-103a-3p(hsa-mir-103a-2) | 162282 | 13604 | 175886 |
| hsa-mir-106a-3p(hsa-mir-106a) | 6 | 0 | 6 |
| hsa-mir-106a-5p(hsa-mir-106a) | 6 | 0 | 6 |
| hsa-mir-106b-3p(hsa-mir-106b) | 1748 | 87 | 1835 |
| hsa-mir-106b-5p(hsa-mir-106b) | 1748 | 103 | 1851 |
| hsa-mir-107(hsa-mir-107) | 52279 | 5197 | 57476 |
| hsa-mir-10a-3p(hsa-mir-10a) | 629 | 0 | 629 |
| hsa-mir-10a-5p(hsa-mir-10a) | 629 | 15 | 644 |
| hsa-mir-1178(hsa-mir-1178) | 7 | 0 | 7 |
| hsa-mir-1185-1-3p(hsa-mir-1185-1) | 402 | 0 | 402 |
| hsa-mir-1185-2-3p(hsa-mir-1185-2) | 172 | 0 | 172 |
| hsa-mir-1185-5p(hsa-mir-1185-1) | 402 | 0 | 402 |
| hsa-mir-1185-5p(hsa-mir-1185-2) | 172 | 0 | 172 |
| hsa-mir-1197(hsa-mir-1197) | 37 | 0 | 37 |
| hsa-mir-122-3p(hsa-mir-122) | 13 | 0 | 13 |
| hsa-mir-122-5p(hsa-mir-122) | 13 | 5 | 18 |
| hsa-mir-1250(hsa-mir-1250) | 48 | 6 | 54 |
| hsa-mir-1255a(hsa-mir-1255a) | 56 | 0 | 56 |
| hsa-mir-1255b-2-3p(hsa-mir-1255b-2) | 6 | 0 | 6 |
| hsa-mir-1255b-5p(hsa-mir-1255b-1) | 6 | 0 | 6 |
| hsa-mir-1255b-5p(hsa-mir-1255b-2) | 6 | 0 | 6 |
| hsa-mir-1256(hsa-mir-1256) | 61 | 0 | 61 |
| hsa-mir-125a-3p(hsa-mir-125a) | 396 | 0 | 396 |
| hsa-mir-125a-5p(hsa-mir-125a) | 396 | 0 | 396 |
| hsa-mir-125b-1-3p(hsa-mir-125b-1) | 33 | 0 | 33 |
| hsa-mir-125b-2-3p(hsa-mir-125b-2) | 33 | 0 | 33 |
| hsa-mir-125b-5p(hsa-mir-125b-1) | 33 | 0 | 33 |
| hsa-mir-125b-5p(hsa-mir-125b-2) | 33 | 0 | 33 |
| hsa-mir-126-3p(hsa-mir-126) | 11971 | 1118 | 13089 |
| hsa-mir-126-5p(hsa-mir-126) | 11971 | 1216 | 13187 |
| hsa-mir-1260a(hsa-mir-1260a) | 0 | 398 | 398 |
| hsa-mir-1260b(hsa-mir-1260b) | 34 | 1348 | 1382 |
| hsa-mir-1261(hsa-mir-1261) | 0 | 180 | 180 |
| hsa-mir-127-3p(hsa-mir-127) | 4576 | 1229 | 5805 |
| hsa-mir-127-5p(hsa-mir-127) | 4576 | 0 | 4576 |
| hsa-mir-1271-3p(hsa-mir-1271) | 85 | 0 | 85 |
| hsa-mir-1271-5p(hsa-mir-1271) | 85 | 8 | 93 |
| hsa-mir-1273c(hsa-mir-1273c) | 6 | 7 | 13 |
| hsa-mir-1277-3p(hsa-mir-1277) | 1309 | 11 | 1320 |
| hsa-mir-1277-5p(hsa-mir-1277) | 1309 | 0 | 1309 |
| hsa-mir-1278(hsa-mir-1278) | 125 | 0 | 125 |
| hsa-mir-128(hsa-mir-128-1) | 5461 | 702 | 6163 |
| hsa-mir-128(hsa-mir-128-2) | 3776 | 610 | 4386 |
| hsa-mir-1280(hsa-mir-1280) | 0 | 61 | 61 |
| hsa-mir-1284(hsa-mir-1284) | 16 | 0 | 16 |
| hsa-mir-1285-3p(hsa-mir-1285-1) | 42 | 18 | 60 |
| hsa-mir-1285-3p(hsa-mir-1285-2) | 27 | 18 | 45 |
| hsa-mir-1285-5p(hsa-mir-1285-1) | 42 | 0 | 42 |
| hsa-mir-1287(hsa-mir-1287) | 240 | 21 | 261 |
| hsa-mir-1291(hsa-mir-1291) | 5 | 0 | 5 |
| hsa-mir-1294(hsa-mir-1294) | 45 | 0 | 45 |
| hsa-mir-1297(hsa-mir-1297) | 3 | 0 | 3 |
| hsa-mir-1299(hsa-mir-1299) | 14 | 5 | 19 |
| hsa-mir-1301(hsa-mir-1301) | 327 | 35 | 362 |
| hsa-mir-1304-3p(hsa-mir-1304) | 17 | 49 | 66 |
| hsa-mir-1304-5p(hsa-mir-1304) | 17 | 0 | 17 |
| hsa-mir-1306-3p(hsa-mir-1306) | 82 | 0 | 82 |
| hsa-mir-1306-5p(hsa-mir-1306) | 82 | 0 | 82 |
| hsa-mir-1307-3p(hsa-mir-1307) | 7435 | 957 | 8392 |
| hsa-mir-1307-5p(hsa-mir-1307) | 7435 | 150 | 7585 |
| hsa-mir-130a-3p(hsa-mir-130a) | 4616 | 351 | 4967 |
| hsa-mir-130a-5p(hsa-mir-130a) | 4616 | 0 | 4616 |
| hsa-mir-130b-3p(hsa-mir-130b) | 874 | 62 | 936 |
| hsa-mir-130b-5p(hsa-mir-130b) | 874 | 0 | 874 |
| hsa-mir-132-3p(hsa-mir-132) | 25 | 19 | 44 |
| hsa-mir-132-5p(hsa-mir-132) | 25 | 0 | 25 |
| hsa-mir-1322(hsa-mir-598) | 2478 | 0 | 2478 |
| hsa-mir-133a(hsa-mir-133a-1) | 5 | 0 | 5 |
| hsa-mir-133a(hsa-mir-133a-2) | 5 | 0 | 5 |
| hsa-mir-134(hsa-mir-134) | 1025 | 272 | 1297 |
| hsa-mir-135a-3p(hsa-mir-135a-1) | 6 | 0 | 6 |
| hsa-mir-135a-5p(hsa-mir-135a-1) | 6 | 0 | 6 |
| hsa-mir-136-3p(hsa-mir-136) | 162 | 0 | 162 |
| hsa-mir-136-5p(hsa-mir-136) | 162 | 6 | 168 |
| hsa-mir-139-3p(hsa-mir-139) | 806 | 38 | 844 |
| hsa-mir-139-5p(hsa-mir-139) | 806 | 14 | 820 |
| hsa-mir-140-3p(hsa-mir-140) | 57735 | 8359 | 66094 |
| hsa-mir-140-5p(hsa-mir-140) | 57735 | 0 | 57735 |
| hsa-mir-142-3p(hsa-mir-142) | 15794 | 35 | 15829 |
| hsa-mir-142-5p(hsa-mir-142) | 15794 | 1011 | 16805 |
| hsa-mir-143-3p(hsa-mir-143) | 2915 | 181 | 3096 |
| hsa-mir-143-5p(hsa-mir-143) | 2915 | 9 | 2924 |
| hsa-mir-144-3p(hsa-mir-144) | 475 | 39 | 514 |
| hsa-mir-144-5p(hsa-mir-144) | 475 | 0 | 475 |
| hsa-mir-1468(hsa-mir-1468) | 5 | 0 | 5 |
| hsa-mir-146a-3p(hsa-mir-146a) | 2117 | 0 | 2117 |
| hsa-mir-146a-5p(hsa-mir-146a) | 2117 | 319 | 2436 |
| hsa-mir-146b-3p(hsa-mir-146b) | 493 | 0 | 493 |
| hsa-mir-146b-5p(hsa-mir-146b) | 493 | 98 | 591 |
| hsa-mir-148a-3p(hsa-mir-148a) | 1485 | 100 | 1585 |
| hsa-mir-148a-5p(hsa-mir-148a) | 1485 | 0 | 1485 |
| hsa-mir-148b-3p(hsa-mir-148b) | 3699 | 661 | 4360 |
| hsa-mir-148b-5p(hsa-mir-148b) | 3699 | 0 | 3699 |
| hsa-mir-151a-3p(hsa-mir-151a) | 12826 | 907 | 13733 |
| hsa-mir-151a-5p(hsa-mir-151a) | 12826 | 117 | 12943 |
| hsa-mir-151b(hsa-mir-151a) | 12826 | 0 | 12826 |
| hsa-mir-151b(hsa-mir-151b) | 171 | 0 | 171 |
| hsa-mir-152(hsa-mir-152) | 9512 | 2531 | 12043 |
| hsa-mir-154-3p(hsa-mir-154) | 168 | 0 | 168 |
| hsa-mir-154-5p(hsa-mir-154) | 168 | 0 | 168 |
| hsa-mir-155-3p(hsa-mir-155) | 130 | 0 | 130 |
| hsa-mir-155-5p(hsa-mir-155) | 130 | 42 | 172 |
| hsa-mir-15a-3p(hsa-mir-15a) | 843 | 0 | 843 |
| hsa-mir-15a-5p(hsa-mir-15a) | 843 | 17 | 860 |
| hsa-mir-15b-3p(hsa-mir-15b) | 1603 | 0 | 1603 |
| hsa-mir-15b-5p(hsa-mir-15b) | 1603 | 48 | 1651 |
| hsa-mir-16-1-3p(hsa-mir-16-1) | 2989 | 0 | 2989 |
| hsa-mir-16-2-3p(hsa-mir-16-2) | 2979 | 0 | 2979 |
| hsa-mir-16-5p(hsa-mir-16-1) | 2989 | 229 | 3218 |
| hsa-mir-16-5p(hsa-mir-16-2) | 2979 | 229 | 3208 |
| hsa-mir-17-3p(hsa-mir-17) | 1126 | 58 | 1184 |
| hsa-mir-17-5p(hsa-mir-17) | 1126 | 40 | 1166 |
| hsa-mir-181a-2-3p(hsa-mir-181a-2) | 4366 | 0 | 4366 |
| hsa-mir-181a-3p(hsa-mir-181a-1) | 4683 | 7 | 4690 |
| hsa-mir-181a-5p(hsa-mir-181a-1) | 4683 | 136 | 4819 |
| hsa-mir-181a-5p(hsa-mir-181a-2) | 4366 | 136 | 4502 |
| hsa-mir-181b-3p(hsa-mir-181b-1) | 342 | 12 | 354 |
| hsa-mir-181b-5p(hsa-mir-181b-1) | 342 | 50 | 392 |
| hsa-mir-181b-5p(hsa-mir-181b-2) | 337 | 50 | 387 |
| hsa-mir-181c-3p(hsa-mir-181c) | 443 | 106 | 549 |
| hsa-mir-181c-5p(hsa-mir-181c) | 443 | 0 | 443 |
| hsa-mir-181d(hsa-mir-181d) | 292 | 27 | 319 |
| hsa-mir-182-3p(hsa-mir-182) | 7 | 0 | 7 |
| hsa-mir-182-5p(hsa-mir-182) | 7 | 0 | 7 |
| hsa-mir-185-3p(hsa-mir-185) | 123307 | 74 | 123381 |
| hsa-mir-185-5p(hsa-mir-185) | 123307 | 9752 | 133059 |
| hsa-mir-186-3p(hsa-mir-186) | 4504 | 0 | 4504 |
| hsa-mir-186-5p(hsa-mir-186) | 4504 | 186 | 4690 |
| hsa-mir-18a-3p(hsa-mir-18a) | 168 | 0 | 168 |
| hsa-mir-18a-5p(hsa-mir-18a) | 168 | 0 | 168 |
| hsa-mir-1908(hsa-mir-1908) | 360 | 277 | 637 |
| hsa-mir-191-3p(hsa-mir-191) | 25056 | 0 | 25056 |
| hsa-mir-191-5p(hsa-mir-191) | 25056 | 2904 | 27960 |
| hsa-mir-192-3p(hsa-mir-192) | 5263 | 0 | 5263 |
| hsa-mir-192-5p(hsa-mir-192) | 5263 | 241 | 5504 |
| hsa-mir-193a-3p(hsa-mir-193a) | 5 | 0 | 5 |
| hsa-mir-193a-5p(hsa-mir-193a) | 5 | 0 | 5 |
| hsa-mir-194-3p(hsa-mir-194-2) | 21 | 0 | 21 |
| hsa-mir-194-5p(hsa-mir-194-1) | 4 | 0 | 4 |
| hsa-mir-194-5p(hsa-mir-194-2) | 21 | 0 | 21 |
| hsa-mir-195-3p(hsa-mir-195) | 16 | 0 | 16 |
| hsa-mir-195-5p(hsa-mir-195) | 16 | 0 | 16 |
| hsa-mir-196b-3p(hsa-mir-196b) | 132 | 0 | 132 |
| hsa-mir-196b-5p(hsa-mir-196b) | 132 | 92 | 224 |
| hsa-mir-197-3p(hsa-mir-197) | 99 | 0 | 99 |
| hsa-mir-197-5p(hsa-mir-197) | 99 | 0 | 99 |
| hsa-mir-199a-3p(hsa-mir-199a-1) | 174869 | 7614 | 182483 |
| hsa-mir-199a-3p(hsa-mir-199a-2) | 174864 | 7627 | 182491 |
| hsa-mir-199a-3p(hsa-mir-199b) | 174764 | 7614 | 182378 |
| hsa-mir-199a-5p(hsa-mir-199a-1) | 174869 | 0 | 174869 |
| hsa-mir-199a-5p(hsa-mir-199a-2) | 174864 | 0 | 174864 |
| hsa-mir-199b-3p(hsa-mir-199a-1) | 174869 | 7614 | 182483 |
| hsa-mir-199b-3p(hsa-mir-199a-2) | 174864 | 7627 | 182491 |
| hsa-mir-199b-3p(hsa-mir-199b) | 174764 | 7614 | 182378 |
| hsa-mir-199b-5p(hsa-mir-199b) | 174764 | 0 | 174764 |
| hsa-mir-19a-3p(hsa-mir-19a) | 5 | 0 | 5 |
| hsa-mir-19a-5p(hsa-mir-19a) | 5 | 0 | 5 |
| hsa-mir-19b-1-5p(hsa-mir-19b-1) | 55 | 0 | 55 |
| hsa-mir-19b-2-5p(hsa-mir-19b-2) | 55 | 0 | 55 |
| hsa-mir-19b-3p(hsa-mir-19b-1) | 55 | 0 | 55 |
| hsa-mir-19b-3p(hsa-mir-19b-2) | 55 | 0 | 55 |
| hsa-mir-200a-3p(hsa-mir-200a) | 6 | 0 | 6 |
| hsa-mir-200a-5p(hsa-mir-200a) | 6 | 0 | 6 |
| hsa-mir-200b-3p(hsa-mir-200b) | 11 | 0 | 11 |
| hsa-mir-200b-5p(hsa-mir-200b) | 11 | 0 | 11 |
| hsa-mir-200c-3p(hsa-mir-200c) | 18 | 0 | 18 |
| hsa-mir-200c-5p(hsa-mir-200c) | 18 | 0 | 18 |
| hsa-mir-203(hsa-mir-203) | 5 | 0 | 5 |
| hsa-mir-204-3p(hsa-mir-204) | 11 | 0 | 11 |
| hsa-mir-204-5p(hsa-mir-204) | 11 | 0 | 11 |
| hsa-mir-20a-3p(hsa-mir-20a) | 264 | 0 | 264 |
| hsa-mir-20a-5p(hsa-mir-20a) | 264 | 22 | 286 |
| hsa-mir-20b-3p(hsa-mir-20b) | 19 | 0 | 19 |
| hsa-mir-20b-5p(hsa-mir-20b) | 19 | 0 | 19 |
| hsa-mir-21-3p(hsa-mir-21) | 103661 | 27 | 103688 |
| hsa-mir-21-5p(hsa-mir-21) | 103661 | 3339 | 107000 |
| hsa-mir-210(hsa-mir-210) | 82 | 5 | 87 |
| hsa-mir-2110(hsa-mir-2110) | 84 | 5 | 89 |
| hsa-mir-215(hsa-mir-215) | 24 | 11 | 35 |
| hsa-mir-22-3p(hsa-mir-22) | 2864 | 199 | 3063 |
| hsa-mir-22-5p(hsa-mir-22) | 2864 | 9 | 2873 |
| hsa-mir-221-3p(hsa-mir-221) | 54800 | 4172 | 58972 |
| hsa-mir-221-5p(hsa-mir-221) | 54800 | 0 | 54800 |
| hsa-mir-222-3p(hsa-mir-222) | 3140 | 107 | 3247 |
| hsa-mir-222-5p(hsa-mir-222) | 3140 | 0 | 3140 |
| hsa-mir-223-3p(hsa-mir-223) | 31613 | 5503 | 37116 |
| hsa-mir-223-5p(hsa-mir-223) | 31613 | 0 | 31613 |
| hsa-mir-224-3p(hsa-mir-224) | 840 | 0 | 840 |
| hsa-mir-224-5p(hsa-mir-224) | 840 | 0 | 840 |
| hsa-mir-2355-3p(hsa-mir-2355) | 555 | 0 | 555 |
| hsa-mir-2355-5p(hsa-mir-2355) | 555 | 14 | 569 |
| hsa-mir-2392(hsa-mir-2392) | 0 | 12265 | 12265 |
| hsa-mir-23a-3p(hsa-mir-23a) | 10109 | 574 | 10683 |
| hsa-mir-23a-5p(hsa-mir-23a) | 10109 | 0 | 10109 |
| hsa-mir-23b-3p(hsa-mir-23b) | 1811 | 64 | 1875 |
| hsa-mir-23b-5p(hsa-mir-23b) | 1811 | 0 | 1811 |
| hsa-mir-24-1-5p(hsa-mir-24-1) | 9450 | 0 | 9450 |
| hsa-mir-24-2-5p(hsa-mir-24-2) | 9487 | 0 | 9487 |
| hsa-mir-24-3p(hsa-mir-24-1) | 9450 | 5565 | 15015 |
| hsa-mir-24-3p(hsa-mir-24-2) | 9487 | 5565 | 15052 |
| hsa-mir-25-3p(hsa-mir-25) | 60490 | 5600 | 66090 |
| hsa-mir-25-5p(hsa-mir-25) | 60490 | 0 | 60490 |
| hsa-mir-26a-1-3p(hsa-mir-26a-1) | 15995 | 0 | 15995 |
| hsa-mir-26a-2-3p(hsa-mir-26a-2) | 15982 | 0 | 15982 |
| hsa-mir-26a-5p(hsa-mir-26a-1) | 15995 | 4269 | 20264 |
| hsa-mir-26a-5p(hsa-mir-26a-2) | 15982 | 4269 | 20251 |
| hsa-mir-26b-3p(hsa-mir-26b) | 50080 | 0 | 50080 |
| hsa-mir-26b-5p(hsa-mir-26b) | 50080 | 1546 | 51626 |
| hsa-mir-27a-3p(hsa-mir-27a) | 9647 | 947 | 10594 |
| hsa-mir-27a-5p(hsa-mir-27a) | 9647 | 0 | 9647 |
| hsa-mir-27b-3p(hsa-mir-27b) | 7126 | 358 | 7484 |
| hsa-mir-27b-5p(hsa-mir-27b) | 7126 | 0 | 7126 |
| hsa-mir-28-3p(hsa-mir-28) | 1543 | 70 | 1613 |
| hsa-mir-28-5p(hsa-mir-28) | 1543 | 13 | 1556 |
| hsa-mir-2964a-3p(hsa-mir-2964a) | 17 | 0 | 17 |
| hsa-mir-2964a-5p(hsa-mir-2964a) | 17 | 0 | 17 |
| hsa-mir-299-3p(hsa-mir-299) | 40 | 0 | 40 |
| hsa-mir-299-5p(hsa-mir-299) | 40 | 0 | 40 |
| hsa-mir-29a-3p(hsa-mir-29a) | 12800 | 476 | 13276 |
| hsa-mir-29a-5p(hsa-mir-29a) | 12800 | 0 | 12800 |
| hsa-mir-29b-1-5p(hsa-mir-29b-1) | 67 | 0 | 67 |
| hsa-mir-29b-2-5p(hsa-mir-29b-2) | 67 | 0 | 67 |
| hsa-mir-29b-3p(hsa-mir-29b-1) | 67 | 0 | 67 |
| hsa-mir-29b-3p(hsa-mir-29b-2) | 67 | 0 | 67 |
| hsa-mir-29c-3p(hsa-mir-29c) | 2366 | 83 | 2449 |
| hsa-mir-29c-5p(hsa-mir-29c) | 2366 | 0 | 2366 |
| hsa-mir-301a-3p(hsa-mir-301a) | 123 | 0 | 123 |
| hsa-mir-301a-5p(hsa-mir-301a) | 123 | 0 | 123 |
| hsa-mir-30a-3p(hsa-mir-30a) | 493 | 28 | 521 |
| hsa-mir-30a-5p(hsa-mir-30a) | 493 | 9 | 502 |
| hsa-mir-30b-3p(hsa-mir-30b) | 332 | 5 | 337 |
| hsa-mir-30b-5p(hsa-mir-30b) | 332 | 16 | 348 |
| hsa-mir-30c-1-3p(hsa-mir-30c-1) | 189 | 0 | 189 |
| hsa-mir-30c-2-3p(hsa-mir-30c-2) | 74 | 0 | 74 |
| hsa-mir-30c-5p(hsa-mir-30c-1) | 189 | 0 | 189 |
| hsa-mir-30c-5p(hsa-mir-30c-2) | 74 | 0 | 74 |
| hsa-mir-30d-3p(hsa-mir-30d) | 12923 | 0 | 12923 |
| hsa-mir-30d-5p(hsa-mir-30d) | 12923 | 435 | 13358 |
| hsa-mir-30e-3p(hsa-mir-30e) | 3731 | 966 | 4697 |
| hsa-mir-30e-5p(hsa-mir-30e) | 3731 | 46 | 3777 |
| hsa-mir-31-3p(hsa-mir-31) | 7 | 0 | 7 |
| hsa-mir-31-5p(hsa-mir-31) | 7 | 0 | 7 |
| hsa-mir-3120-3p(hsa-mir-3120) | 149 | 248 | 397 |
| hsa-mir-3120-5p(hsa-mir-3120) | 149 | 0 | 149 |
| hsa-mir-3121-3p(hsa-mir-3121) | 7 | 0 | 7 |
| hsa-mir-3121-5p(hsa-mir-3121) | 7 | 0 | 7 |
| hsa-mir-3123(hsa-mir-3123) | 43 | 694 | 737 |
| hsa-mir-3124-3p(hsa-mir-3124) | 21 | 0 | 21 |
| hsa-mir-3124-5p(hsa-mir-3124) | 21 | 6 | 27 |
| hsa-mir-3130-3p(hsa-mir-3130-1) | 13 | 0 | 13 |
| hsa-mir-3130-3p(hsa-mir-3130-2) | 13 | 0 | 13 |
| hsa-mir-3130-5p(hsa-mir-3130-1) | 13 | 0 | 13 |
| hsa-mir-3130-5p(hsa-mir-3130-2) | 13 | 0 | 13 |
| hsa-mir-3135b(hsa-mir-3135b) | 0 | 251 | 251 |
| hsa-mir-3136-3p(hsa-mir-3136) | 16 | 0 | 16 |
| hsa-mir-3136-5p(hsa-mir-3136) | 16 | 0 | 16 |
| hsa-mir-3138(hsa-mir-3138) | 63 | 0 | 63 |
| hsa-mir-3140-3p(hsa-mir-3140) | 5 | 0 | 5 |
| hsa-mir-3140-5p(hsa-mir-3140) | 5 | 0 | 5 |
| hsa-mir-3141(hsa-mir-3141) | 0 | 10 | 10 |
| hsa-mir-3143(hsa-mir-3143) | 5 | 0 | 5 |
| hsa-mir-3161(hsa-mir-3161) | 10 | 0 | 10 |
| hsa-mir-3168(hsa-mir-3168) | 178 | 5715 | 5893 |
| hsa-mir-3174(hsa-mir-3174) | 17 | 0 | 17 |
| hsa-mir-3175(hsa-mir-3175) | 14 | 0 | 14 |
| hsa-mir-3177-3p(hsa-mir-3177) | 20 | 5 | 25 |
| hsa-mir-3177-5p(hsa-mir-3177) | 20 | 0 | 20 |
| hsa-mir-3182(hsa-mir-3182) | 30 | 177 | 207 |
| hsa-mir-3183(hsa-mir-3183) | 17 | 0 | 17 |
| hsa-mir-3190-3p(hsa-mir-3190) | 12 | 0 | 12 |
| hsa-mir-3190-5p(hsa-mir-3190) | 12 | 0 | 12 |
| hsa-mir-3191-3p(hsa-mir-3191) | 8 | 0 | 8 |
| hsa-mir-3191-5p(hsa-mir-3191) | 8 | 0 | 8 |
| hsa-mir-3196(hsa-mir-3196) | 19 | 0 | 19 |
| hsa-mir-3198(hsa-mir-3198-1) | 8 | 0 | 8 |
| hsa-mir-3198(hsa-mir-3198-2) | 8 | 0 | 8 |
| hsa-mir-32-3p(hsa-mir-32) | 56 | 0 | 56 |
| hsa-mir-32-5p(hsa-mir-32) | 56 | 5 | 61 |
| hsa-mir-320a(hsa-mir-320a) | 40758 | 6755 | 47513 |
| hsa-mir-320b(hsa-mir-320b-1) | 169 | 45 | 214 |
| hsa-mir-320b(hsa-mir-320b-2) | 169 | 45 | 214 |
| hsa-mir-320c(hsa-mir-320c-1) | 35 | 104 | 139 |
| hsa-mir-320c(hsa-mir-320c-2) | 35 | 73 | 108 |
| hsa-mir-320d(hsa-mir-320d-1) | 11 | 11 | 22 |
| hsa-mir-320d(hsa-mir-320d-2) | 11 | 11 | 22 |
| hsa-mir-323a-3p(hsa-mir-323a) | 1382 | 51 | 1433 |
| hsa-mir-323a-5p(hsa-mir-323a) | 1382 | 0 | 1382 |
| hsa-mir-323b-3p(hsa-mir-323b) | 1561 | 107 | 1668 |
| hsa-mir-323b-5p(hsa-mir-323b) | 1561 | 0 | 1561 |
| hsa-mir-324-3p(hsa-mir-324) | 73 | 0 | 73 |
| hsa-mir-324-5p(hsa-mir-324) | 73 | 0 | 73 |
| hsa-mir-326(hsa-mir-326) | 9 | 201 | 210 |
| hsa-mir-328(hsa-mir-328) | 154 | 11 | 165 |
| hsa-mir-329(hsa-mir-329-1) | 38 | 15 | 53 |
| hsa-mir-329(hsa-mir-329-2) | 38 | 15 | 53 |
| hsa-mir-330-3p(hsa-mir-330) | 4100 | 370 | 4470 |
| hsa-mir-330-5p(hsa-mir-330) | 4100 | 6 | 4106 |
| hsa-mir-331-3p(hsa-mir-331) | 295 | 64 | 359 |
| hsa-mir-331-5p(hsa-mir-331) | 295 | 0 | 295 |
| hsa-mir-335-3p(hsa-mir-335) | 1766 | 5 | 1771 |
| hsa-mir-335-5p(hsa-mir-335) | 1766 | 9 | 1775 |
| hsa-mir-337-3p(hsa-mir-337) | 5 | 0 | 5 |
| hsa-mir-337-5p(hsa-mir-337) | 5 | 0 | 5 |
| hsa-mir-338-3p(hsa-mir-338) | 48 | 0 | 48 |
| hsa-mir-338-5p(hsa-mir-338) | 48 | 0 | 48 |
| hsa-mir-339-3p(hsa-mir-339) | 1956 | 417 | 2373 |
| hsa-mir-339-5p(hsa-mir-339) | 1956 | 120 | 2076 |
| hsa-mir-33a-3p(hsa-mir-33a) | 3956 | 0 | 3956 |
| hsa-mir-33a-5p(hsa-mir-33a) | 3956 | 256 | 4212 |
| hsa-mir-33b-3p(hsa-mir-33b) | 142 | 0 | 142 |
| hsa-mir-33b-5p(hsa-mir-33b) | 142 | 0 | 142 |
| hsa-mir-340-3p(hsa-mir-340) | 24109 | 0 | 24109 |
| hsa-mir-340-5p(hsa-mir-340) | 24109 | 1130 | 25239 |
| hsa-mir-342-3p(hsa-mir-342) | 186 | 5 | 191 |
| hsa-mir-342-5p(hsa-mir-342) | 186 | 0 | 186 |
| hsa-mir-345-3p(hsa-mir-345) | 21 | 0 | 21 |
| hsa-mir-345-5p(hsa-mir-345) | 21 | 161 | 182 |
| hsa-mir-3605-3p(hsa-mir-3605) | 7 | 0 | 7 |
| hsa-mir-3605-5p(hsa-mir-3605) | 7 | 0 | 7 |
| hsa-mir-361-3p(hsa-mir-361) | 145 | 0 | 145 |
| hsa-mir-361-5p(hsa-mir-361) | 145 | 45 | 190 |
| hsa-mir-3615(hsa-mir-3615) | 148 | 41 | 189 |
| hsa-mir-363-3p(hsa-mir-363) | 535 | 240 | 775 |
| hsa-mir-363-5p(hsa-mir-363) | 535 | 0 | 535 |
| hsa-mir-3656(hsa-mir-3656) | 52 | 100 | 152 |
| hsa-mir-3676-3p(hsa-mir-3676) | 28 | 0 | 28 |
| hsa-mir-3676-5p(hsa-mir-3676) | 28 | 12 | 40 |
| hsa-mir-3677-3p(hsa-mir-3677) | 29 | 5 | 34 |
| hsa-mir-3677-5p(hsa-mir-3677) | 29 | 0 | 29 |
| hsa-mir-369-3p(hsa-mir-369) | 1780 | 6 | 1786 |
| hsa-mir-369-5p(hsa-mir-369) | 1780 | 5 | 1785 |
| hsa-mir-370(hsa-mir-370) | 18 | 22 | 40 |
| hsa-mir-374a-3p(hsa-mir-374a) | 1787 | 0 | 1787 |
| hsa-mir-374a-5p(hsa-mir-374a) | 1787 | 48 | 1835 |
| hsa-mir-374b-3p(hsa-mir-374b) | 608 | 0 | 608 |
| hsa-mir-374b-5p(hsa-mir-374b) | 608 | 30 | 638 |
| hsa-mir-375(hsa-mir-375) | 5 | 0 | 5 |
| hsa-mir-376a-3p(hsa-mir-376a-1) | 15 | 70 | 85 |
| hsa-mir-376a-3p(hsa-mir-376a-2) | 15 | 70 | 85 |
| hsa-mir-376a-5p(hsa-mir-376a-1) | 15 | 0 | 15 |
| hsa-mir-376b(hsa-mir-376b) | 72 | 6 | 78 |
| hsa-mir-376c(hsa-mir-376c) | 270 | 49 | 319 |
| hsa-mir-377-3p(hsa-mir-377) | 45 | 0 | 45 |
| hsa-mir-377-5p(hsa-mir-377) | 45 | 0 | 45 |
| hsa-mir-378a-3p(hsa-mir-378a) | 1642 | 419 | 2061 |
| hsa-mir-378a-5p(hsa-mir-378a) | 1642 | 0 | 1642 |
| hsa-mir-378b(hsa-mir-378b) | 1 | 0 | 1 |
| hsa-mir-378c(hsa-mir-378c) | 259 | 6 | 265 |
| hsa-mir-378d(hsa-mir-378d-1) | 27 | 0 | 27 |
| hsa-mir-378d(hsa-mir-378d-2) | 27 | 0 | 27 |
| hsa-mir-378e(hsa-mir-378e) | 1 | 0 | 1 |
| hsa-mir-378f(hsa-mir-378f) | 1 | 18 | 19 |
| hsa-mir-378i(hsa-mir-378i) | 0 | 62 | 62 |
| hsa-mir-379-3p(hsa-mir-379) | 989 | 9 | 998 |
| hsa-mir-379-5p(hsa-mir-379) | 989 | 212 | 1201 |
| hsa-mir-380-3p(hsa-mir-380) | 8 | 0 | 8 |
| hsa-mir-380-5p(hsa-mir-380) | 8 | 0 | 8 |
| hsa-mir-381(hsa-mir-381) | 78 | 5 | 83 |
| hsa-mir-382-3p(hsa-mir-382) | 1975 | 0 | 1975 |
| hsa-mir-382-5p(hsa-mir-382) | 1975 | 25 | 2000 |
| hsa-mir-3912(hsa-mir-3912) | 7 | 0 | 7 |
| hsa-mir-3920(hsa-mir-3920) | 5 | 0 | 5 |
| hsa-mir-3928(hsa-mir-3928) | 76 | 59 | 135 |
| hsa-mir-409-3p(hsa-mir-409) | 854 | 20 | 874 |
| hsa-mir-409-5p(hsa-mir-409) | 854 | 0 | 854 |
| hsa-mir-410(hsa-mir-410) | 436 | 39 | 475 |
| hsa-mir-411-3p(hsa-mir-411) | 708 | 35 | 743 |
| hsa-mir-411-5p(hsa-mir-411) | 708 | 5 | 713 |
| hsa-mir-412(hsa-mir-412) | 74 | 0 | 74 |
| hsa-mir-421(hsa-mir-421) | 146 | 13 | 159 |
| hsa-mir-423-3p(hsa-mir-423) | 43280 | 552 | 43832 |
| hsa-mir-423-5p(hsa-mir-423) | 43280 | 1705 | 44985 |
| hsa-mir-424-3p(hsa-mir-424) | 452 | 10 | 462 |
| hsa-mir-424-5p(hsa-mir-424) | 452 | 0 | 452 |
| hsa-mir-425-3p(hsa-mir-425) | 1746 | 226 | 1972 |
| hsa-mir-425-5p(hsa-mir-425) | 1746 | 8 | 1754 |
| hsa-mir-4286(hsa-mir-4286) | 1002 | 31 | 1033 |
| hsa-mir-4306(hsa-mir-4306) | 109 | 26 | 135 |
| hsa-mir-431-3p(hsa-mir-431) | 76 | 0 | 76 |
| hsa-mir-431-5p(hsa-mir-431) | 76 | 0 | 76 |
| hsa-mir-432-3p(hsa-mir-432) | 1989 | 0 | 1989 |
| hsa-mir-432-5p(hsa-mir-432) | 1989 | 575 | 2564 |
| hsa-mir-433(hsa-mir-433) | 1840 | 80 | 1920 |
| hsa-mir-4429(hsa-mir-4429) | 0 | 19 | 19 |
| hsa-mir-4433-3p(hsa-mir-4433) | 41 | 8754 | 8795 |
| hsa-mir-4433-5p(hsa-mir-4433) | 41 | 23 | 64 |
| hsa-mir-4435(hsa-mir-4435-1) | 37 | 4 | 41 |
| hsa-mir-4435(hsa-mir-4435-2) | 37 | 4 | 41 |
| hsa-mir-4436b-3p(hsa-mir-4436b-1) | 5 | 0 | 5 |
| hsa-mir-4436b-3p(hsa-mir-4436b-2) | 5 | 0 | 5 |
| hsa-mir-4436b-5p(hsa-mir-4436b-1) | 5 | 0 | 5 |
| hsa-mir-4436b-5p(hsa-mir-4436b-2) | 5 | 0 | 5 |
| hsa-mir-4443(hsa-mir-4443) | 71 | 16 | 87 |
| hsa-mir-4446-3p(hsa-mir-4446) | 678 | 163 | 841 |
| hsa-mir-4446-5p(hsa-mir-4446) | 678 | 0 | 678 |
| hsa-mir-4447(hsa-mir-4447) | 0 | 37 | 37 |
| hsa-mir-4448(hsa-mir-4448) | 21 | 87 | 108 |
| hsa-mir-4454(hsa-mir-4454) | 38 | 102 | 140 |
| hsa-mir-4455(hsa-mir-4455) | 0 | 126 | 126 |
| hsa-mir-4466(hsa-mir-4466) | 0 | 11 | 11 |
| hsa-mir-4469(hsa-mir-4469) | 14 | 0 | 14 |
| hsa-mir-4470(hsa-mir-4470) | 19 | 0 | 19 |
| hsa-mir-4483(hsa-mir-4483) | 0 | 28 | 28 |
| hsa-mir-4488(hsa-mir-4488) | 21 | 0 | 21 |
| hsa-mir-4492(hsa-mir-4492) | 53 | 0 | 53 |
| hsa-mir-4497(hsa-mir-4497) | 0 | 46 | 46 |
| hsa-mir-4500(hsa-mir-4500) | 29 | 0 | 29 |
| hsa-mir-4508(hsa-mir-4508) | 1699 | 3707 | 5406 |
| hsa-mir-450a-3p(hsa-mir-450a-2) | 17 | 0 | 17 |
| hsa-mir-450a-5p(hsa-mir-450a-1) | 12 | 0 | 12 |
| hsa-mir-450a-5p(hsa-mir-450a-2) | 17 | 0 | 17 |
| hsa-mir-450b-3p(hsa-mir-450b) | 13 | 0 | 13 |
| hsa-mir-450b-5p(hsa-mir-450b) | 13 | 0 | 13 |
| hsa-mir-4510(hsa-mir-4510) | 0 | 1 | 1 |
| hsa-mir-4516(hsa-mir-4516) | 51 | 31 | 82 |
| hsa-mir-451a(hsa-mir-451a) | 436 | 0 | 436 |
| hsa-mir-452-3p(hsa-mir-452) | 229 | 0 | 229 |
| hsa-mir-452-5p(hsa-mir-452) | 229 | 0 | 229 |
| hsa-mir-4523(hsa-mir-4523) | 21 | 0 | 21 |
| hsa-mir-4531(hsa-mir-4531) | 0 | 86 | 86 |
| hsa-mir-454-3p(hsa-mir-454) | 7 | 0 | 7 |
| hsa-mir-454-5p(hsa-mir-454) | 7 | 0 | 7 |
| hsa-mir-4659b-3p(hsa-mir-4659b) | 18 | 0 | 18 |
| hsa-mir-4659b-5p(hsa-mir-4659b) | 18 | 0 | 18 |
| hsa-mir-4665-3p(hsa-mir-4665) | 12 | 0 | 12 |
| hsa-mir-4665-5p(hsa-mir-4665) | 12 | 0 | 12 |
| hsa-mir-4667-3p(hsa-mir-4667) | 5 | 0 | 5 |
| hsa-mir-4667-5p(hsa-mir-4667) | 5 | 0 | 5 |
| hsa-mir-4707-3p(hsa-mir-4707) | 8 | 0 | 8 |
| hsa-mir-4707-5p(hsa-mir-4707) | 8 | 0 | 8 |
| hsa-mir-4708-3p(hsa-mir-4708) | 5 | 0 | 5 |
| hsa-mir-4708-5p(hsa-mir-4708) | 5 | 0 | 5 |
| hsa-mir-4734(hsa-mir-4734) | 11 | 0 | 11 |
| hsa-mir-4750(hsa-mir-4750) | 0 | 12 | 12 |
| hsa-mir-4765(hsa-mir-4765) | 5 | 0 | 5 |
| hsa-mir-4770(hsa-mir-4770) | 0 | 6 | 6 |
| hsa-mir-4791(hsa-mir-4791) | 5 | 0 | 5 |
| hsa-mir-484(hsa-mir-484) | 343 | 58 | 401 |
| hsa-mir-485-3p(hsa-mir-485) | 2597 | 158 | 2755 |
| hsa-mir-485-5p(hsa-mir-485) | 2597 | 31 | 2628 |
| hsa-mir-486-3p(hsa-mir-486) | 5687 | 471 | 6158 |
| hsa-mir-486-5p(hsa-mir-486) | 5687 | 1482 | 7169 |
| hsa-mir-487a(hsa-mir-487a) | 65 | 0 | 65 |
| hsa-mir-487b(hsa-mir-487b) | 511 | 5 | 516 |
| hsa-mir-490-3p(hsa-mir-490) | 10 | 0 | 10 |
| hsa-mir-490-5p(hsa-mir-490) | 10 | 0 | 10 |
| hsa-mir-491-3p(hsa-mir-491) | 5 | 0 | 5 |
| hsa-mir-491-5p(hsa-mir-491) | 5 | 0 | 5 |
| hsa-mir-493-3p(hsa-mir-493) | 306 | 32 | 338 |
| hsa-mir-493-5p(hsa-mir-493) | 306 | 0 | 306 |
| hsa-mir-494(hsa-mir-494) | 280 | 0 | 280 |
| hsa-mir-495(hsa-mir-495) | 2826 | 63 | 2889 |
| hsa-mir-496(hsa-mir-496) | 163 | 49 | 212 |
| hsa-mir-497-3p(hsa-mir-497) | 57 | 0 | 57 |
| hsa-mir-497-5p(hsa-mir-497) | 57 | 0 | 57 |
| hsa-mir-499a-3p(hsa-mir-499a) | 135 | 0 | 135 |
| hsa-mir-499a-5p(hsa-mir-499a) | 135 | 0 | 135 |
| hsa-mir-499b-3p(hsa-mir-499b) | 5 | 0 | 5 |
| hsa-mir-499b-5p(hsa-mir-499b) | 5 | 0 | 5 |
| hsa-mir-500a-3p(hsa-mir-500a) | 69 | 0 | 69 |
| hsa-mir-500a-5p(hsa-mir-500a) | 69 | 0 | 69 |
| hsa-mir-500b(hsa-mir-500a) | 69 | 0 | 69 |
| hsa-mir-501-3p(hsa-mir-501) | 21 | 0 | 21 |
| hsa-mir-501-5p(hsa-mir-501) | 21 | 0 | 21 |
| hsa-mir-5010-3p(hsa-mir-5010) | 316 | 0 | 316 |
| hsa-mir-5010-5p(hsa-mir-5010) | 316 | 8 | 324 |
| hsa-mir-502-3p(hsa-mir-502) | 57 | 0 | 57 |
| hsa-mir-502-5p(hsa-mir-502) | 57 | 0 | 57 |
| hsa-mir-503(hsa-mir-503) | 607 | 132 | 739 |
| hsa-mir-504(hsa-mir-504) | 14 | 0 | 14 |
| hsa-mir-505-3p(hsa-mir-505) | 66 | 0 | 66 |
| hsa-mir-505-5p(hsa-mir-505) | 66 | 0 | 66 |
| hsa-mir-5187-3p(hsa-mir-5187) | 80 | 0 | 80 |
| hsa-mir-5187-5p(hsa-mir-5187) | 80 | 0 | 80 |
| hsa-mir-5189(hsa-mir-5189) | 144 | 0 | 144 |
| hsa-mir-532-3p(hsa-mir-532) | 650 | 0 | 650 |
| hsa-mir-532-5p(hsa-mir-532) | 650 | 14 | 664 |
| hsa-mir-539-3p(hsa-mir-539) | 335 | 0 | 335 |
| hsa-mir-539-5p(hsa-mir-539) | 335 | 0 | 335 |
| hsa-mir-542-3p(hsa-mir-542) | 89 | 0 | 89 |
| hsa-mir-542-5p(hsa-mir-542) | 89 | 0 | 89 |
| hsa-mir-543(hsa-mir-543) | 1768 | 321 | 2089 |
| hsa-mir-548ad(hsa-mir-548ad) | 1 | 0 | 1 |
| hsa-mir-548ae(hsa-mir-548ae-2) | 1 | 0 | 1 |
| hsa-mir-548ah-3p(hsa-mir-548p) | 6 | 0 | 6 |
| hsa-mir-548am-3p(hsa-mir-548am) | 20 | 0 | 20 |
| hsa-mir-548am-5p(hsa-mir-548am) | 20 | 0 | 20 |
| hsa-mir-548am-5p(hsa-mir-548c) | 20 | 0 | 20 |
| hsa-mir-548am-5p(hsa-mir-548o-2) | 25 | 0 | 25 |
| hsa-mir-548ap-5p(hsa-mir-548j) | 999 | 0 | 999 |
| hsa-mir-548au-3p(hsa-mir-548au) | 20 | 0 | 20 |
| hsa-mir-548au-5p(hsa-mir-548am) | 20 | 0 | 20 |
| hsa-mir-548au-5p(hsa-mir-548c) | 20 | 0 | 20 |
| hsa-mir-548au-5p(hsa-mir-548o-2) | 25 | 0 | 25 |
| hsa-mir-548av-3p(hsa-mir-548o) | 5 | 0 | 5 |
| hsa-mir-548av-3p(hsa-mir-548o-2) | 25 | 0 | 25 |
| hsa-mir-548av-3p(hsa-mir-548p) | 6 | 0 | 6 |
| hsa-mir-548av-5p(hsa-mir-548k) | 107 | 0 | 107 |
| hsa-mir-548ax(hsa-mir-548ax) | 10 | 0 | 10 |
| hsa-mir-548c-3p(hsa-mir-548c) | 20 | 0 | 20 |
| hsa-mir-548c-5p(hsa-mir-548am) | 20 | 0 | 20 |
| hsa-mir-548c-5p(hsa-mir-548c) | 20 | 0 | 20 |
| hsa-mir-548c-5p(hsa-mir-548o-2) | 25 | 0 | 25 |
| hsa-mir-548d-3p(hsa-mir-548d-1) | 1 | 0 | 1 |
| hsa-mir-548d-3p(hsa-mir-548d-2) | 1 | 0 | 1 |
| hsa-mir-548d-5p(hsa-mir-548d-1) | 1 | 0 | 1 |
| hsa-mir-548d-5p(hsa-mir-548d-2) | 1 | 0 | 1 |
| hsa-mir-548e(hsa-mir-548e) | 111 | 0 | 111 |
| hsa-mir-548j(hsa-mir-548j) | 999 | 9 | 1008 |
| hsa-mir-548k(hsa-mir-548k) | 107 | 0 | 107 |
| hsa-mir-548l(hsa-mir-548l) | 7 | 0 | 7 |
| hsa-mir-548n(hsa-mir-548n) | 8 | 0 | 8 |
| hsa-mir-548o-3p(hsa-mir-548o) | 5 | 0 | 5 |
| hsa-mir-548o-3p(hsa-mir-548o-2) | 25 | 0 | 25 |
| hsa-mir-548o-5p(hsa-mir-548am) | 20 | 0 | 20 |
| hsa-mir-548o-5p(hsa-mir-548c) | 20 | 0 | 20 |
| hsa-mir-548o-5p(hsa-mir-548o-2) | 25 | 0 | 25 |
| hsa-mir-548p(hsa-mir-548p) | 6 | 0 | 6 |
| hsa-mir-550a-3-5p(hsa-mir-550a-1) | 2 | 0 | 2 |
| hsa-mir-550a-3-5p(hsa-mir-550a-2) | 2 | 0 | 2 |
| hsa-mir-550a-3-5p(hsa-mir-550a-3) | 2 | 0 | 2 |
| hsa-mir-550a-3p(hsa-mir-550a-1) | 2 | 0 | 2 |
| hsa-mir-550a-3p(hsa-mir-550a-2) | 2 | 0 | 2 |
| hsa-mir-550a-3p(hsa-mir-550a-3) | 2 | 0 | 2 |
| hsa-mir-550a-5p(hsa-mir-550a-1) | 2 | 0 | 2 |
| hsa-mir-550a-5p(hsa-mir-550a-2) | 2 | 0 | 2 |
| hsa-mir-551b-3p(hsa-mir-551b) | 11 | 11 | 22 |
| hsa-mir-551b-5p(hsa-mir-551b) | 11 | 0 | 11 |
| hsa-mir-552(hsa-mir-552) | 10 | 0 | 10 |
| hsa-mir-556-3p(hsa-mir-556) | 9 | 0 | 9 |
| hsa-mir-556-5p(hsa-mir-556) | 9 | 0 | 9 |
| hsa-mir-5586-3p(hsa-mir-5586) | 6 | 0 | 6 |
| hsa-mir-574-3p(hsa-mir-574) | 180 | 11 | 191 |
| hsa-mir-574-5p(hsa-mir-574) | 180 | 0 | 180 |
| hsa-mir-576-3p(hsa-mir-576) | 27 | 0 | 27 |
| hsa-mir-576-5p(hsa-mir-576) | 27 | 0 | 27 |
| hsa-mir-584-3p(hsa-mir-584) | 23138 | 0 | 23138 |
| hsa-mir-584-5p(hsa-mir-584) | 23138 | 3541 | 26679 |
| hsa-mir-589-3p(hsa-mir-589) | 187 | 0 | 187 |
| hsa-mir-589-5p(hsa-mir-589) | 187 | 34 | 221 |
| hsa-mir-590-3p(hsa-mir-590) | 64 | 0 | 64 |
| hsa-mir-590-5p(hsa-mir-590) | 64 | 0 | 64 |
| hsa-mir-598(hsa-mir-598) | 2478 | 39 | 2517 |
| hsa-mir-605(hsa-mir-605) | 31 | 0 | 31 |
| hsa-mir-624-3p(hsa-mir-624) | 11 | 0 | 11 |
| hsa-mir-624-5p(hsa-mir-624) | 11 | 0 | 11 |
| hsa-mir-625-3p(hsa-mir-625) | 515 | 5 | 520 |
| hsa-mir-625-5p(hsa-mir-625) | 515 | 0 | 515 |
| hsa-mir-627(hsa-mir-627) | 10 | 0 | 10 |
| hsa-mir-628-3p(hsa-mir-628) | 32 | 0 | 32 |
| hsa-mir-628-5p(hsa-mir-628) | 32 | 0 | 32 |
| hsa-mir-629-3p(hsa-mir-629) | 263 | 0 | 263 |
| hsa-mir-629-5p(hsa-mir-629) | 263 | 0 | 263 |
| hsa-mir-636(hsa-mir-636) | 26 | 0 | 26 |
| hsa-mir-639(hsa-mir-639) | 5 | 0 | 5 |
| hsa-mir-641(hsa-mir-641) | 9 | 0 | 9 |
| hsa-mir-643(hsa-mir-643) | 17 | 0 | 17 |
| hsa-mir-644b-3p(hsa-mir-644b) | 7 | 0 | 7 |
| hsa-mir-651(hsa-mir-651) | 6 | 0 | 6 |
| hsa-mir-652-3p(hsa-mir-652) | 466 | 713 | 1179 |
| hsa-mir-652-5p(hsa-mir-652) | 466 | 0 | 466 |
| hsa-mir-654-3p(hsa-mir-654) | 530 | 55 | 585 |
| hsa-mir-654-5p(hsa-mir-654) | 530 | 0 | 530 |
| hsa-mir-655(hsa-mir-655) | 12 | 0 | 12 |
| hsa-mir-656(hsa-mir-656) | 9 | 0 | 9 |
| hsa-mir-660-3p(hsa-mir-660) | 150 | 0 | 150 |
| hsa-mir-660-5p(hsa-mir-660) | 150 | 0 | 150 |
| hsa-mir-664-3p(hsa-mir-664) | 1191 | 0 | 1191 |
| hsa-mir-664-5p(hsa-mir-664) | 1191 | 0 | 1191 |
| hsa-mir-665(hsa-mir-665) | 18 | 0 | 18 |
| hsa-mir-671-3p(hsa-mir-671) | 39 | 17 | 56 |
| hsa-mir-671-5p(hsa-mir-671) | 39 | 0 | 39 |
| hsa-mir-7-1-3p(hsa-mir-7-1) | 34 | 0 | 34 |
| hsa-mir-7-5p(hsa-mir-7-1) | 34 | 0 | 34 |
| hsa-mir-720(hsa-mir-720) | 774 | 598 | 1372 |
| hsa-mir-744-3p(hsa-mir-744) | 49263 | 0 | 49263 |
| hsa-mir-744-5p(hsa-mir-744) | 49263 | 14545 | 63808 |
| hsa-mir-758(hsa-mir-758) | 23 | 0 | 23 |
| hsa-mir-760(hsa-mir-760) | 32 | 0 | 32 |
| hsa-mir-762(hsa-mir-762) | 7 | 0 | 7 |
| hsa-mir-766-3p(hsa-mir-766) | 153 | 0 | 153 |
| hsa-mir-766-5p(hsa-mir-766) | 153 | 6 | 159 |
| hsa-mir-769-3p(hsa-mir-769) | 124 | 0 | 124 |
| hsa-mir-769-5p(hsa-mir-769) | 124 | 20 | 144 |
| hsa-mir-874(hsa-mir-874) | 14 | 0 | 14 |
| hsa-mir-877-3p(hsa-mir-877) | 35 | 0 | 35 |
| hsa-mir-889(hsa-mir-889) | 240 | 0 | 240 |
| hsa-mir-9-3p(hsa-mir-9-1) | 13 | 0 | 13 |
| hsa-mir-9-3p(hsa-mir-9-2) | 13 | 0 | 13 |
| hsa-mir-9-3p(hsa-mir-9-3) | 13 | 0 | 13 |
| hsa-mir-9-5p(hsa-mir-9-1) | 13 | 0 | 13 |
| hsa-mir-9-5p(hsa-mir-9-2) | 13 | 0 | 13 |
| hsa-mir-9-5p(hsa-mir-9-3) | 13 | 0 | 13 |
| hsa-mir-92a-1-5p(hsa-mir-92a-1) | 25340 | 0 | 25340 |
| hsa-mir-92a-2-5p(hsa-mir-92a-2) | 24425 | 0 | 24425 |
| hsa-mir-92a-3p(hsa-mir-92a-1) | 25340 | 2239 | 27579 |
| hsa-mir-92a-3p(hsa-mir-92a-2) | 24425 | 2210 | 26635 |
| hsa-mir-92b-3p(hsa-mir-92b) | 649 | 368 | 1017 |
| hsa-mir-92b-5p(hsa-mir-92b) | 649 | 0 | 649 |
| hsa-mir-93-3p(hsa-mir-93) | 1165 | 5 | 1170 |
| hsa-mir-93-5p(hsa-mir-93) | 1165 | 54 | 1219 |
| hsa-mir-940(hsa-mir-940) | 0 | 5 | 5 |
| hsa-mir-941(hsa-mir-941-1) | 55 | 0 | 55 |
| hsa-mir-941(hsa-mir-941-2) | 55 | 0 | 55 |
| hsa-mir-941(hsa-mir-941-3) | 55 | 0 | 55 |
| hsa-mir-941(hsa-mir-941-4) | 55 | 0 | 55 |
| hsa-mir-942(hsa-mir-942) | 5 | 0 | 5 |
| hsa-mir-98(hsa-mir-98) | 2325 | 90 | 2415 |
| hsa-mir-99a-3p(hsa-mir-99a) | 147 | 0 | 147 |
| hsa-mir-99a-5p(hsa-mir-99a) | 147 | 25 | 172 |
| hsa-mir-99b-3p(hsa-mir-99b) | 2007 | 12 | 2019 |
| hsa-mir-99b-5p(hsa-mir-99b) | 2007 | 617 | 2624 |
| Sum(Avg) | 8374377 | 344916 | 8719293 |

  

|  |
| --- |
| hsa-let-7a-2-3p(hsa-let-7a-2) AGGTTGAGGTAGTAGGTTGTATAGTTTAGAATTACATCAAGGGAGATAACTGTACAGCCTCCTAGCTTTCCT (((..(((.(((.(((((((((((((.....(..(.....)..)...))))))))))))).))).))).))) (-25.20) \*\*\*\*\*\*\*\*\*\*\*\*\*\*\*\*\*\*\*\*\*\*\*\*\*\*\*\*\*\*\*\*\*\*\*\*\*\*\*\*\*\*\*\*\*\*\*\*\*CTGTACAGCCTCCTAGCTTTCC\* T  M |
| ....TGAGGTAGTAGGTTGTATAGTT............................................... 124184   | 124184; |
| ....TGAGGTAGTAGGTTGTATAGT................................................ 30655   | 30655; |
| ....TGAGGTAGTAGGTTGTATAGTTT.............................................. 5293   | 5293; |
| ....TGAGGTAGTAGGTTGTATAG................................................. 3487   | 3487; |
| ....TGAGGTANTAGGTTGTATAGTT............................................... 1278   | 1278; |
| ....TGAGGTAGTNGGTTGTATAGTT............................................... 1159   | 1159; |
| ....TGAGGTAGTAGGTTNTATAGTT............................................... 1017   | 1017; |
| ....TGAGGTAGTAGGTTGTATA.................................................. 974   | 974; |
| ....TGAGGTAGTAGGTTGTAT................................................... 707   | 707; |
| ....NGAGGTAGTAGGTTGTATAGTT............................................... 597   | 597; |
| ....TGAGGTAGTAGNTTGTATAGTT............................................... 391   | 391; |
| ....TGAGGTANTAGGTTGTATAGT................................................ 325   | 325; |
| ....TGAGGTAGTNGGTTGTATAGT................................................ 291   | 291; |
| ....TGAGGTAGTAGGTTNTATAGT................................................ 243   | 243; |
| .....GAGGTAGTAGGTTGTATAGTT............................................... 168   | 168; |
| ....TGAGGTAGTAGGTTGTA.................................................... 166   | 166; |
| ....TGAGGTAGTAGGTTGTNTAGTT............................................... 148   | 148; |
| ....TGAGGTAGNAGGTTGTATAGTT............................................... 139   | 139; |
| ....NGAGGTAGTAGGTTGTATAGT................................................ 135   | 135; |
| ....TGAGGTAGTAGGTTGNATAGTT............................................... 134   | 134; |
| ....TNAGGTAGTAGGTTGTATAGTT............................................... 133   | 133; |
| ....TGAGGNAGTAGGTTGTATAGTT............................................... 128   | 128; |
| ....TGAGNTAGTAGGTTGTATAGTT............................................... 126   | 126; |
| ....TGAGGTAGTAGGTTGTANAGTT............................................... 123   | 123; |
| ....TGNGGTAGTAGGTTGTATAGTT............................................... 121   | 121; |
| ....TGAGGTAGTAGGTNGTATAGTT............................................... 121   | 121; |
| ....TGAGGTAGTAGGTTGTATAGNT............................................... 121   | 121; |
| ....TGAGGTAGTANGTTGTATAGTT............................................... 119   | 119; |
| ....TGAGGTAGTAGGTTGTATANTT............................................... 118   | 118; |
| ....TGAGGTAGTAGGTTGTATAGTN............................................... 118   | 118; |
| ....TGAGGTAGTAGGNTGTATAGTT............................................... 117   | 117; |
| ....TGAGGTNGTAGGTTGTATAGTT............................................... 115   | 115; |
| ....TGANGTAGTAGGTTGTATAGTT............................................... 112   | 112; |
| ....TGAGGTAGTAGGTTGTATNGTT............................................... 110   | 110; |
| ....TGAGGTAGTAGNTTGTATAGT................................................ 82   | 82; |
| ....TGAGGTAGTAGG......................................................... 72   | 72; |
| ....TGAGGTAGTAGGTTGT..................................................... 60   | 60; |
| ......AGGTAGTAGGTTGTATAGTT............................................... 49   | 49; |
| ....TGAGGTANTAGGTTGTATAGTTT.............................................. 46   | 46; |
| .....GAGGTAGTAGGTTGTATAGT................................................ 43   | 43; |
| ....TGAGGTAGTAGGTTGTNTAGT................................................ 42   | 42; |
| ....TGAGGTAGTNGGTTGTATAGTTT.............................................. 41   | 41; |
| ....TGAGGTAGNAGGTTGTATAGT................................................ 38   | 38; |
| ....TGAGGTAGTAGGTTNTATAGTTT.............................................. 35   | 35; |
| ....TGAGGTAGTAGGTTGTATAGTTTA............................................. 35   | 35; |
| ....TNAGGTAGTAGGTTGTATAGT................................................ 34   | 34; |
| ....TGAGGTAGTAGGTTGNATAGT................................................ 33   | 33; |
| ....TGAGGTANTAGGTTGTATAG................................................. 33   | 33; |
| ....TGAGGTAGTAGGNTGTATAGT................................................ 33   | 33; |
| ....TGAGGTAGTAGGTTNTATAG................................................. 33   | 33; |
| ....TGAGGTAGTNGGTTGTATAG................................................. 31   | 31; |
| ....TGAGGTAGTAGGTNGTATAGT................................................ 31   | 31; |
| ....TGAGGTAGTAGGTTGTATAGN................................................ 30   | 30; |
| ....TGAGGTAGTAGGTTGTATNGT................................................ 29   | 29; |
| ....NGAGGTAGTAGGTTGTATAGTTT.............................................. 29   | 29; |
| ....TGAGGTAGTAGGTTGTATANT................................................ 28   | 28; |
| ....NGAGGTAGTAGGTTGTATAG................................................. 28   | 28; |
| ....TGAGGNAGTAGGTTGTATAGT................................................ 27   | 27; |
| ....TGAGGTAGTANGTTGTATAGT................................................ 27   | 27; |
| ....TGNGGTAGTAGGTTGTATAGT................................................ 25   | 25; |
| ...TTGAGGTAGTAGGTTGTATAGT................................................ 24   | 24; |
| ....TGAGNTAGTAGGTTGTATAGT................................................ 23   | 23; |
| ....TGAGGTAGTAGGTTGTANAGT................................................ 19   | 19; |
| ....TGANGTAGTAGGTTGTATAGT................................................ 19   | 19; |
| ....TGAGGTNGTAGGTTGTATAGT................................................ 18   | 18; |
| .....GAGGTAGTAGGTTGTATAGTTT.............................................. 15   | 15; |
| ....NGAGGTAGTAGGTTGTATA.................................................. 13   | 13; |
| ....TGAGGTAGTAGGTTG...................................................... 11   | 11; |
| ......AGGTAGTAGGTTGTATAGT................................................ 11   | 11; |
| ....TGAGGTAGTAGGTTGTATNGTTT.............................................. 10   | 10; |
| ....TGAGGTAGTAGNTTGTATAGTTT.............................................. 10   | 10; |
| ...TTGAGGTAGTAGGTTGTATAG................................................. 9   | 9; |
| ....TGAGGTAGTAGGTTNTAT................................................... 9   | 9; |
| ....TGAGGTAGTAGGTTNTATA.................................................. 8   | 8; |
| ....TGAGGTAGNAGGTTGTATAGTTT.............................................. 8   | 8; |
| ....TGAGGTAGTAGGTTGTATAGTNT.............................................. 7   | 7; |
| ....TGNGGTAGTAGGTTGTATAGTTT.............................................. 7   | 7; |
| ....TGAGGTAGTNGGTTGTATA.................................................. 7   | 7; |
| ....TGAGGTANTAGGTTGTAT................................................... 7   | 7; |
| ....TGAGGTAGTAGNTTGTATAG................................................. 7   | 7; |
| ....TGNGGTAGTAGGTTGTATAG................................................. 7   | 7; |
| ....TGAGGTAGTAGGTT....................................................... 6   | 6; |
| ....TGAGGTAGTAGGTTGTATAGTTN.............................................. 6   | 6; |
| ....TGAGNTAGTAGGTTGTATAGTTT.............................................. 5   | 5; |
| ....TGAGGTANTAGGTTGTATA.................................................. 5   | 5; |
| ....TGAGGTAGTAGGTTGTNTAGTTT.............................................. 5   | 5; |
| ....TGAGGTAGTAGGTTGNATAGTTT.............................................. 5   | 5; |
| ....TGAGGTAGTAGGT........................................................ 4   | 4; |
| ......AGGTAGTAGGTTGTATAGTTT.............................................. 3   | 3; |
| .....GAGGTAGTAGGTTGTATAG................................................. 3   | 3; |
| ....TGAGGTAGTAG.......................................................... 1   | 1; |
| ------------------------------------------------------------------------ 174459 |
| ------------------------------------------------------------------------ 174459 |
| hsa-let-7a-3p(hsa-let-7a-1) TGGGATGAGGTAGTAGGTTGTATAGTTTTAGGGTCACACCCACCACTGGGAGATAACTATACAATCTACTGTCTTTCCTA (((((.(((..((((((((((((((((...(((.....))).((....))....))))))))))))))))..)))))))) (-35.60) \*\*\*\*\*\*\*\*\*\*\*\*\*\*\*\*\*\*\*\*\*\*\*\*\*\*\*\*\*\*\*\*\*\*\*\*\*\*\*\*\*\*\*\*\*\*\*\*\*\*\*\*\*\*\*\*CTATACAATCTACTGTCTTTC\*\*\* T  M |
| .....TGAGGTAGTAGGTTGTATAGTT...................................................... 124184   | 124184; |
| .....TGAGGTAGTAGGTTGTATAGT....................................................... 30655   | 30655; |
| .....TGAGGTAGTAGGTTGTATAGTTT..................................................... 5293   | 5293; |
| .....TGAGGTAGTAGGTTGTATAG........................................................ 3487   | 3487; |
| .....TGAGGTANTAGGTTGTATAGTT...................................................... 1278   | 1278; |
| .....TGAGGTAGTNGGTTGTATAGTT...................................................... 1159   | 1159; |
| .....TGAGGTAGTAGGTTNTATAGTT...................................................... 1017   | 1017; |
| .....TGAGGTAGTAGGTTGTATA......................................................... 974   | 974; |
| .....TGAGGTAGTAGGTTGTAT.......................................................... 707   | 707; |
| .....NGAGGTAGTAGGTTGTATAGTT...................................................... 597   | 597; |
| .....TGAGGTAGTAGNTTGTATAGTT...................................................... 391   | 391; |
| .....TGAGGTANTAGGTTGTATAGT....................................................... 325   | 325; |
| .....TGAGGTAGTNGGTTGTATAGT....................................................... 291   | 291; |
| .....TGAGGTAGTAGGTTNTATAGT....................................................... 243   | 243; |
| ......GAGGTAGTAGGTTGTATAGTT...................................................... 168   | 168; |
| .....TGAGGTAGTAGGTTGTA........................................................... 166   | 166; |
| .....TGAGGTAGTAGGTTGTNTAGTT...................................................... 148   | 148; |
| .....TGAGGTAGNAGGTTGTATAGTT...................................................... 139   | 139; |
| .....NGAGGTAGTAGGTTGTATAGT....................................................... 135   | 135; |
| .....TGAGGTAGTAGGTTGNATAGTT...................................................... 134   | 134; |
| .....TNAGGTAGTAGGTTGTATAGTT...................................................... 133   | 133; |
| .....TGAGGNAGTAGGTTGTATAGTT...................................................... 128   | 128; |
| .....TGAGNTAGTAGGTTGTATAGTT...................................................... 126   | 126; |
| .....TGAGGTAGTAGGTTGTANAGTT...................................................... 123   | 123; |
| .....TGAGGTAGTAGGTTGTATAGNT...................................................... 121   | 121; |
| .....TGAGGTAGTAGGTNGTATAGTT...................................................... 121   | 121; |
| .....TGNGGTAGTAGGTTGTATAGTT...................................................... 121   | 121; |
| .....TGAGGTAGTANGTTGTATAGTT...................................................... 119   | 119; |
| .....TGAGGTAGTAGGTTGTATAGTN...................................................... 118   | 118; |
| .....TGAGGTAGTAGGTTGTATANTT...................................................... 118   | 118; |
| .....TGAGGTAGTAGGNTGTATAGTT...................................................... 117   | 117; |
| .....TGAGGTNGTAGGTTGTATAGTT...................................................... 115   | 115; |
| .....TGANGTAGTAGGTTGTATAGTT...................................................... 112   | 112; |
| .....TGAGGTAGTAGGTTGTATNGTT...................................................... 110   | 110; |
| .....TGAGGTAGTAGNTTGTATAGT....................................................... 82   | 82; |
| .....TGAGGTAGTAGG................................................................ 72   | 72; |
| .....TGAGGTAGTAGGTTGT............................................................ 60   | 60; |
| .......AGGTAGTAGGTTGTATAGTT...................................................... 49   | 49; |
| .....TGAGGTAGTAGGTTGTATAGTTTT.................................................... 47   | 47; |
| .....TGAGGTANTAGGTTGTATAGTTT..................................................... 46   | 46; |
| ......GAGGTAGTAGGTTGTATAGT....................................................... 43   | 43; |
| .....TGAGGTAGTAGGTTGTNTAGT....................................................... 42   | 42; |
| .....TGAGGTAGTNGGTTGTATAGTTT..................................................... 41   | 41; |
| .....TGAGGTAGNAGGTTGTATAGT....................................................... 38   | 38; |
| .....TGAGGTAGTAGGTTNTATAGTTT..................................................... 35   | 35; |
| .....TNAGGTAGTAGGTTGTATAGT....................................................... 34   | 34; |
| .....TGAGGTAGTAGGTTGNATAGT....................................................... 33   | 33; |
| .....TGAGGTAGTAGGTTNTATAG........................................................ 33   | 33; |
| .....TGAGGTANTAGGTTGTATAG........................................................ 33   | 33; |
| .....TGAGGTAGTAGGNTGTATAGT....................................................... 33   | 33; |
| .....TGAGGTAGTNGGTTGTATAG........................................................ 31   | 31; |
| .....TGAGGTAGTAGGTNGTATAGT....................................................... 31   | 31; |
| .....TGAGGTAGTAGGTTGTATAGN....................................................... 30   | 30; |
| .....TGAGGTAGTAGGTTGTATNGT....................................................... 29   | 29; |
| .....NGAGGTAGTAGGTTGTATAGTTT..................................................... 29   | 29; |
| .....TGAGGTAGTAGGTTGTATANT....................................................... 28   | 28; |
| .....NGAGGTAGTAGGTTGTATAG........................................................ 28   | 28; |
| .....TGAGGTAGTANGTTGTATAGT....................................................... 27   | 27; |
| .....TGAGGNAGTAGGTTGTATAGT....................................................... 27   | 27; |
| .....TGNGGTAGTAGGTTGTATAGT....................................................... 25   | 25; |
| .....TGAGNTAGTAGGTTGTATAGT....................................................... 23   | 23; |
| .....TGANGTAGTAGGTTGTATAGT....................................................... 19   | 19; |
| .....TGAGGTAGTAGGTTGTANAGT....................................................... 19   | 19; |
| .....TGAGGTNGTAGGTTGTATAGT....................................................... 18   | 18; |
| ......GAGGTAGTAGGTTGTATAGTTT..................................................... 15   | 15; |
| .....NGAGGTAGTAGGTTGTATA......................................................... 13   | 13; |
| .......AGGTAGTAGGTTGTATAGT....................................................... 11   | 11; |
| .....TGAGGTAGTAGGTTG............................................................. 11   | 11; |
| .....TGAGGTAGTAGNTTGTATAGTTT..................................................... 10   | 10; |
| .....TGAGGTAGTAGGTTGTATNGTTT..................................................... 10   | 10; |
| .....TGAGGTAGTAGGTTNTAT.......................................................... 9   | 9; |
| .....TGAGGTAGNAGGTTGTATAGTTT..................................................... 8   | 8; |
| .....TGAGGTAGTAGGTTNTATA......................................................... 8   | 8; |
| .....TGAGGTAGTAGNTTGTATAG........................................................ 7   | 7; |
| .....TGAGGTAGTAGGTTGTATAGTNT..................................................... 7   | 7; |
| .....TGNGGTAGTAGGTTGTATAGTTT..................................................... 7   | 7; |
| .....TGAGGTAGTNGGTTGTATA......................................................... 7   | 7; |
| .....TGNGGTAGTAGGTTGTATAG........................................................ 7   | 7; |
| .....TGAGGTANTAGGTTGTAT.......................................................... 7   | 7; |
| .....TGAGGTAGTAGGTTGTATAGTTN..................................................... 6   | 6; |
| .....TGAGGTAGTAGGTT.............................................................. 6   | 6; |
| .....TGAGNTAGTAGGTTGTATAGTTT..................................................... 5   | 5; |
| .....TGAGGTAGTAGGTTGNATAGTTT..................................................... 5   | 5; |
| .....TGAGGTAGTAGGTTGTNTAGTTT..................................................... 5   | 5; |
| .....TGAGGTANTAGGTTGTATA......................................................... 5   | 5; |
| .....TGAGGTAGTAGGT............................................................... 4   | 4; |
| ......GAGGTAGTAGGTTGTATAG........................................................ 3   | 3; |
| .......AGGTAGTAGGTTGTATAGTTT..................................................... 3   | 3; |
| .....TGAGGTAGTAG................................................................. 1   | 1; |
| -------------------------------------------------------------------------------- 174438 |
| ........................................................CTATACAATCTACTGTCTTTCt... 3   | 3; |
| -------------------------------------------------------------------------------- 3 |
| -------------------------------------------------------------------------------- 174441 |
| hsa-let-7a-3p(hsa-let-7a-3) GGGTGAGGTAGTAGGTTGTATAGTTTGGGGCTCTGCCCTGCTATGGGATAACTATACAATCTACTGTCTTTCCT (((.(((..((((((((((((((((((((((...)))))).........))))))))))))))))..))).))) (-34.40) \*\*\*\*\*\*\*\*\*\*\*\*\*\*\*\*\*\*\*\*\*\*\*\*\*\*\*\*\*\*\*\*\*\*\*\*\*\*\*\*\*\*\*\*\*\*\*\*\*\*\*CTATACAATCTACTGTCTTTC\*\* T  M |
| ...TGAGGTAGTAGGTTGTATAGTT.................................................. 124184   | 124184; |
| ...TGAGGTAGTAGGTTGTATAGT................................................... 30655   | 30655; |
| ...TGAGGTAGTAGGTTGTATAGTTT................................................. 5293   | 5293; |
| ...TGAGGTAGTAGGTTGTATAG.................................................... 3487   | 3487; |
| ...TGAGGTANTAGGTTGTATAGTT.................................................. 1278   | 1278; |
| ...TGAGGTAGTNGGTTGTATAGTT.................................................. 1159   | 1159; |
| ...TGAGGTAGTAGGTTNTATAGTT.................................................. 1017   | 1017; |
| ...TGAGGTAGTAGGTTGTATA..................................................... 974   | 974; |
| ...TGAGGTAGTAGGTTGTAT...................................................... 707   | 707; |
| ...NGAGGTAGTAGGTTGTATAGTT.................................................. 597   | 597; |
| ...TGAGGTAGTAGNTTGTATAGTT.................................................. 391   | 391; |
| ...TGAGGTANTAGGTTGTATAGT................................................... 325   | 325; |
| ...TGAGGTAGTNGGTTGTATAGT................................................... 291   | 291; |
| ...TGAGGTAGTAGGTTNTATAGT................................................... 243   | 243; |
| ....GAGGTAGTAGGTTGTATAGTT.................................................. 168   | 168; |
| ...TGAGGTAGTAGGTTGTA....................................................... 166   | 166; |
| ...TGAGGTAGTAGGTTGTNTAGTT.................................................. 148   | 148; |
| ...TGAGGTAGNAGGTTGTATAGTT.................................................. 139   | 139; |
| ...NGAGGTAGTAGGTTGTATAGT................................................... 135   | 135; |
| ...TGAGGTAGTAGGTTGNATAGTT.................................................. 134   | 134; |
| ...TNAGGTAGTAGGTTGTATAGTT.................................................. 133   | 133; |
| ...TGAGGNAGTAGGTTGTATAGTT.................................................. 128   | 128; |
| ...TGAGNTAGTAGGTTGTATAGTT.................................................. 126   | 126; |
| ...TGAGGTAGTAGGTTGTANAGTT.................................................. 123   | 123; |
| ...TGAGGTAGTAGGTNGTATAGTT.................................................. 121   | 121; |
| ...TGAGGTAGTAGGTTGTATAGNT.................................................. 121   | 121; |
| ...TGNGGTAGTAGGTTGTATAGTT.................................................. 121   | 121; |
| ...TGAGGTAGTANGTTGTATAGTT.................................................. 119   | 119; |
| ...TGAGGTAGTAGGTTGTATAGTN.................................................. 118   | 118; |
| ...TGAGGTAGTAGGTTGTATANTT.................................................. 118   | 118; |
| ...TGAGGTAGTAGGNTGTATAGTT.................................................. 117   | 117; |
| ...TGAGGTNGTAGGTTGTATAGTT.................................................. 115   | 115; |
| ...TGANGTAGTAGGTTGTATAGTT.................................................. 112   | 112; |
| ...TGAGGTAGTAGGTTGTATNGTT.................................................. 110   | 110; |
| ..GTGAGGTAGTAGGTTGTATAGT................................................... 86   | 86; |
| ...TGAGGTAGTAGNTTGTATAGT................................................... 82   | 82; |
| ...TGAGGTAGTAGG............................................................ 72   | 72; |
| ...TGAGGTAGTAGGTTGT........................................................ 60   | 60; |
| .....AGGTAGTAGGTTGTATAGTT.................................................. 49   | 49; |
| ...TGAGGTANTAGGTTGTATAGTTT................................................. 46   | 46; |
| ....GAGGTAGTAGGTTGTATAGT................................................... 43   | 43; |
| ...TGAGGTAGTAGGTTGTNTAGT................................................... 42   | 42; |
| ...TGAGGTAGTNGGTTGTATAGTTT................................................. 41   | 41; |
| ...TGAGGTAGNAGGTTGTATAGT................................................... 38   | 38; |
| ...TGAGGTAGTAGGTTNTATAGTTT................................................. 35   | 35; |
| ...TNAGGTAGTAGGTTGTATAGT................................................... 34   | 34; |
| ...TGAGGTANTAGGTTGTATAG.................................................... 33   | 33; |
| ...TGAGGTAGTAGGTTGNATAGT................................................... 33   | 33; |
| ...TGAGGTAGTAGGTTNTATAG.................................................... 33   | 33; |
| ...TGAGGTAGTAGGNTGTATAGT................................................... 33   | 33; |
| ...TGAGGTAGTAGGTNGTATAGT................................................... 31   | 31; |
| ...TGAGGTAGTNGGTTGTATAG.................................................... 31   | 31; |
| ...TGAGGTAGTAGGTTGTATAGN................................................... 30   | 30; |
| ...NGAGGTAGTAGGTTGTATAGTTT................................................. 29   | 29; |
| ...TGAGGTAGTAGGTTGTATNGT................................................... 29   | 29; |
| ...NGAGGTAGTAGGTTGTATAG.................................................... 28   | 28; |
| ...TGAGGTAGTAGGTTGTATANT................................................... 28   | 28; |
| ...TGAGGNAGTAGGTTGTATAGT................................................... 27   | 27; |
| ...TGAGGTAGTANGTTGTATAGT................................................... 27   | 27; |
| ...TGNGGTAGTAGGTTGTATAGT................................................... 25   | 25; |
| ...TGAGNTAGTAGGTTGTATAGT................................................... 23   | 23; |
| ..GTGAGGTAGTAGGTTGTATAG.................................................... 19   | 19; |
| ...TGANGTAGTAGGTTGTATAGT................................................... 19   | 19; |
| ...TGAGGTAGTAGGTTGTANAGT................................................... 19   | 19; |
| ...TGAGGTNGTAGGTTGTATAGT................................................... 18   | 18; |
| ....GAGGTAGTAGGTTGTATAGTTT................................................. 15   | 15; |
| ...NGAGGTAGTAGGTTGTATA..................................................... 13   | 13; |
| ...TGAGGTAGTAGGTTG......................................................... 11   | 11; |
| .....AGGTAGTAGGTTGTATAGT................................................... 11   | 11; |
| ...TGAGGTAGTAGNTTGTATAGTTT................................................. 10   | 10; |
| ...TGAGGTAGTAGGTTGTATNGTTT................................................. 10   | 10; |
| ...TGAGGTAGTAGGTTNTAT...................................................... 9   | 9; |
| .....AGGTAGTAGGTTGTATAGTTTG................................................ 8   | 8; |
| ...TGAGGTAGNAGGTTGTATAGTTT................................................. 8   | 8; |
| ..GTGAGGTAGTAGGTTGTATAGTT.................................................. 8   | 8; |
| ...TGAGGTAGTAGGTTNTATA..................................................... 8   | 8; |
| ...TGAGGTAGTAGGTTGTATAGTNT................................................. 7   | 7; |
| ...TGAGGTANTAGGTTGTAT...................................................... 7   | 7; |
| ...TGAGGTAGTAGNTTGTATAG.................................................... 7   | 7; |
| ...TGNGGTAGTAGGTTGTATAG.................................................... 7   | 7; |
| ...TGNGGTAGTAGGTTGTATAGTTT................................................. 7   | 7; |
| ...TGAGGTAGTNGGTTGTATA..................................................... 7   | 7; |
| ...TGAGGTAGTAGGTTGTATAGTTN................................................. 6   | 6; |
| ...TGAGGTAGTAGGTT.......................................................... 6   | 6; |
| ..GTGAGGTAGTAGGTTGTAT...................................................... 6   | 6; |
| ...TGAGNTAGTAGGTTGTATAGTTT................................................. 5   | 5; |
| ...TGAGGTAGTAGGTTGNATAGTTT................................................. 5   | 5; |
| ...TGAGGTAGTAGGTTGTNTAGTTT................................................. 5   | 5; |
| ...TGAGGTANTAGGTTGTATA..................................................... 5   | 5; |
| ...TGAGGTAGTAGGT........................................................... 4   | 4; |
| .....AGGTAGTAGGTTGTATAGTTT................................................. 3   | 3; |
| ....GAGGTAGTAGGTTGTATAG.................................................... 3   | 3; |
| ...TGAGGTAGTAG............................................................. 1   | 1; |
| -------------------------------------------------------------------------- 174518 |
| ...................................................CTATACAATCTACTGTCTTTCt.. 3   | 3; |
| -------------------------------------------------------------------------- 3 |
| -------------------------------------------------------------------------- 174521 |
| hsa-let-7a-5p(hsa-let-7a-1) TGGGATGAGGTAGTAGGTTGTATAGTTTTAGGGTCACACCCACCACTGGGAGATAACTATACAATCTACTGTCTTTCCTA (((((.(((..((((((((((((((((...(((.....))).((....))....))))))))))))))))..)))))))) (-35.60) \*\*\*\*\*TGAGGTAGTAGGTTGTATAGTT\*\*\*\*\*\*\*\*\*\*\*\*\*\*\*\*\*\*\*\*\*\*\*\*\*\*\*\*\*\*\*\*\*\*\*\*\*\*\*\*\*\*\*\*\*\*\*\*\*\*\*\*\* T  M |
| .....TGAGGTAGTAGGTTGTATAGTT......................................................\* 124184   | 124184; |
| .....TGAGGTAGTAGGTTGTATAGT....................................................... 30655   | 30655; |
| .....TGAGGTAGTAGGTTGTATAGTTT..................................................... 5293   | 5293; |
| .....TGAGGTAGTAGGTTGTATAG........................................................ 3487   | 3487; |
| .....TGAGGTANTAGGTTGTATAGTT...................................................... 1278   | 1278; |
| .....TGAGGTAGTNGGTTGTATAGTT...................................................... 1159   | 1159; |
| .....TGAGGTAGTAGGTTNTATAGTT...................................................... 1017   | 1017; |
| .....TGAGGTAGTAGGTTGTATA......................................................... 974   | 974; |
| .....TGAGGTAGTAGGTTGTAT.......................................................... 707   | 707; |
| .....NGAGGTAGTAGGTTGTATAGTT...................................................... 597   | 597; |
| .....TGAGGTAGTAGNTTGTATAGTT...................................................... 391   | 391; |
| .....TGAGGTANTAGGTTGTATAGT....................................................... 325   | 325; |
| .....TGAGGTAGTNGGTTGTATAGT....................................................... 291   | 291; |
| .....TGAGGTAGTAGGTTNTATAGT....................................................... 243   | 243; |
| ......GAGGTAGTAGGTTGTATAGTT...................................................... 168   | 168; |
| .....TGAGGTAGTAGGTTGTA........................................................... 166   | 166; |
| .....TGAGGTAGTAGGTTGTNTAGTT...................................................... 148   | 148; |
| .....TGAGGTAGNAGGTTGTATAGTT...................................................... 139   | 139; |
| .....NGAGGTAGTAGGTTGTATAGT....................................................... 135   | 135; |
| .....TGAGGTAGTAGGTTGNATAGTT...................................................... 134   | 134; |
| .....TNAGGTAGTAGGTTGTATAGTT...................................................... 133   | 133; |
| .....TGAGGNAGTAGGTTGTATAGTT...................................................... 128   | 128; |
| .....TGAGNTAGTAGGTTGTATAGTT...................................................... 126   | 126; |
| .....TGAGGTAGTAGGTTGTANAGTT...................................................... 123   | 123; |
| .....TGAGGTAGTAGGTTGTATAGNT...................................................... 121   | 121; |
| .....TGAGGTAGTAGGTNGTATAGTT...................................................... 121   | 121; |
| .....TGNGGTAGTAGGTTGTATAGTT...................................................... 121   | 121; |
| .....TGAGGTAGTANGTTGTATAGTT...................................................... 119   | 119; |
| .....TGAGGTAGTAGGTTGTATAGTN...................................................... 118   | 118; |
| .....TGAGGTAGTAGGTTGTATANTT...................................................... 118   | 118; |
| .....TGAGGTAGTAGGNTGTATAGTT...................................................... 117   | 117; |
| .....TGAGGTNGTAGGTTGTATAGTT...................................................... 115   | 115; |
| .....TGANGTAGTAGGTTGTATAGTT...................................................... 112   | 112; |
| .....TGAGGTAGTAGGTTGTATNGTT...................................................... 110   | 110; |
| .....TGAGGTAGTAGNTTGTATAGT....................................................... 82   | 82; |
| .....TGAGGTAGTAGG................................................................ 72   | 72; |
| .....TGAGGTAGTAGGTTGT............................................................ 60   | 60; |
| .......AGGTAGTAGGTTGTATAGTT...................................................... 49   | 49; |
| .....TGAGGTAGTAGGTTGTATAGTTTT.................................................... 47   | 47; |
| .....TGAGGTANTAGGTTGTATAGTTT..................................................... 46   | 46; |
| ......GAGGTAGTAGGTTGTATAGT....................................................... 43   | 43; |
| .....TGAGGTAGTAGGTTGTNTAGT....................................................... 42   | 42; |
| .....TGAGGTAGTNGGTTGTATAGTTT..................................................... 41   | 41; |
| .....TGAGGTAGNAGGTTGTATAGT....................................................... 38   | 38; |
| .....TGAGGTAGTAGGTTNTATAGTTT..................................................... 35   | 35; |
| .....TNAGGTAGTAGGTTGTATAGT....................................................... 34   | 34; |
| .....TGAGGTAGTAGGTTGNATAGT....................................................... 33   | 33; |
| .....TGAGGTAGTAGGTTNTATAG........................................................ 33   | 33; |
| .....TGAGGTANTAGGTTGTATAG........................................................ 33   | 33; |
| .....TGAGGTAGTAGGNTGTATAGT....................................................... 33   | 33; |
| .....TGAGGTAGTNGGTTGTATAG........................................................ 31   | 31; |
| .....TGAGGTAGTAGGTNGTATAGT....................................................... 31   | 31; |
| .....TGAGGTAGTAGGTTGTATAGN....................................................... 30   | 30; |
| .....TGAGGTAGTAGGTTGTATNGT....................................................... 29   | 29; |
| .....NGAGGTAGTAGGTTGTATAGTTT..................................................... 29   | 29; |
| .....TGAGGTAGTAGGTTGTATANT....................................................... 28   | 28; |
| .....NGAGGTAGTAGGTTGTATAG........................................................ 28   | 28; |
| .....TGAGGTAGTANGTTGTATAGT....................................................... 27   | 27; |
| .....TGAGGNAGTAGGTTGTATAGT....................................................... 27   | 27; |
| .....TGNGGTAGTAGGTTGTATAGT....................................................... 25   | 25; |
| .....TGAGNTAGTAGGTTGTATAGT....................................................... 23   | 23; |
| .....TGANGTAGTAGGTTGTATAGT....................................................... 19   | 19; |
| .....TGAGGTAGTAGGTTGTANAGT....................................................... 19   | 19; |
| .....TGAGGTNGTAGGTTGTATAGT....................................................... 18   | 18; |
| ......GAGGTAGTAGGTTGTATAGTTT..................................................... 15   | 15; |
| .....NGAGGTAGTAGGTTGTATA......................................................... 13   | 13; |
| .......AGGTAGTAGGTTGTATAGT....................................................... 11   | 11; |
| .....TGAGGTAGTAGGTTG............................................................. 11   | 11; |
| .....TGAGGTAGTAGNTTGTATAGTTT..................................................... 10   | 10; |
| .....TGAGGTAGTAGGTTGTATNGTTT..................................................... 10   | 10; |
| .....TGAGGTAGTAGGTTNTAT.......................................................... 9   | 9; |
| .....TGAGGTAGNAGGTTGTATAGTTT..................................................... 8   | 8; |
| .....TGAGGTAGTAGGTTNTATA......................................................... 8   | 8; |
| .....TGAGGTAGTAGNTTGTATAG........................................................ 7   | 7; |
| .....TGAGGTAGTAGGTTGTATAGTNT..................................................... 7   | 7; |
| .....TGNGGTAGTAGGTTGTATAGTTT..................................................... 7   | 7; |
| .....TGAGGTAGTNGGTTGTATA......................................................... 7   | 7; |
| .....TGNGGTAGTAGGTTGTATAG........................................................ 7   | 7; |
| .....TGAGGTANTAGGTTGTAT.......................................................... 7   | 7; |
| .....TGAGGTAGTAGGTTGTATAGTTN..................................................... 6   | 6; |
| .....TGAGGTAGTAGGTT.............................................................. 6   | 6; |
| .....TGAGNTAGTAGGTTGTATAGTTT..................................................... 5   | 5; |
| .....TGAGGTAGTAGGTTGNATAGTTT..................................................... 5   | 5; |
| .....TGAGGTAGTAGGTTGTNTAGTTT..................................................... 5   | 5; |
| .....TGAGGTANTAGGTTGTATA......................................................... 5   | 5; |
| .....TGAGGTAGTAGGT............................................................... 4   | 4; |
| ......GAGGTAGTAGGTTGTATAG........................................................ 3   | 3; |
| .......AGGTAGTAGGTTGTATAGTTT..................................................... 3   | 3; |
| .....TGAGGTAGTAG................................................................. 1   | 1; |
| -------------------------------------------------------------------------------- 174438 |
| .....TGAGGTAGTAGGTTGTATAGTTa..................................................... 3567   | 3567; |
| .....TGAGGTAGgAGGTTGTATAGTTT..................................................... 475   | 475; |
| .....TGAGGTAGTAGGTTGTATAGTa...................................................... 345   | 345; |
| .....TGAGGTAGTAGGTTGTA--GTT...................................................... 242   | 242; |
| .....TGAGGTAGTAGGTTGTcTAGTT...................................................... 164   | 164; |
| .....TGAGGTgGTAGGTTGTATAGTT...................................................... 130   | 130; |
| .....TGAGGTAGTA--TTGTATAGTT...................................................... 126   | 126; |
| .....TGAGGcAGTAGGTTGTATAGTT...................................................... 115   | 115; |
| .....TGAGGTAGTAGGTTGTgTAGTT...................................................... 114   | 114; |
| .....TGAGGTAGTAGGTTGTATAGTaa..................................................... 111   | 111; |
| .....TGgGGTAGTAGGTTGTATAGTT...................................................... 101   | 101; |
| .....aGAGGTAGTAGGTTGTATAGTT...................................................... 98   | 98; |
| .....TaAGGTAGTAGGTTGTATAGTT...................................................... 97   | 97; |
| .....cGAGGTAGTAGGTTGTATAGTT...................................................... 86   | 86; |
| .....TGAGGTAGTAGtTTGTATAGTT...................................................... 81   | 81; |
| .....TGAGGTAGTAGGTTGTATAGgT...................................................... 78   | 78; |
| .....TGAGGTAGTgGGTTGTATAGTT...................................................... 74   | 74; |
| .....TGAGGTAGTAGcTTGTATAGTT...................................................... 73   | 73; |
| .....TGAGGTAtTAGGTTGTATAGTT...................................................... 73   | 73; |
| .....TGAGGTAGTAGGTTGTATAGcT...................................................... 73   | 73; |
| .....TGAGGTAGTAGGTTGTATAGTTc..................................................... 70   | 70; |
| .....TGAGGTAGTAGGTTGTATAGa....................................................... 70   | 70; |
| .....TGAGGTAGTAGGT-GTATAGTT...................................................... 69   | 69; |
| .....TGAGGTAGTAGGTTGTtTAGTT...................................................... 69   | 69; |
| .....TGAGGTAGTAGGcTGTATAGTT...................................................... 68   | 68; |
| .....TGAGGTAGTAGGTTGTATAtTT...................................................... 67   | 67; |
| .....TGAGGTAGTAG-TTGTATAGTT...................................................... 67   | 67; |
| .....TGAGGaAGTAGGTTGTATAGTT...................................................... 65   | 65; |
| .....TGAGGTAGTAGGgTGTATAGTT...................................................... 65   | 65; |
| .....TGAGGTAGTAGGTT--ATAGTT...................................................... 62   | 62; |
| .....TGAGGTAGTAGGTcGTATAGTT...................................................... 59   | 59; |
| .....TGAGGTAGcAGGTTGTATAGTT...................................................... 56   | 56; |
| .....TGAGGTAGTAGGTTGgATAGTT...................................................... 54   | 54; |
| .....TGAGGTAGTAGGTTGTATAGaT...................................................... 53   | 53; |
| .....TGAGGTAGTAGGTTGTAcAGTT...................................................... 47   | 47; |
| .....TGAGGTAGTAGGTTGTcTAGT....................................................... 45   | 45; |
| .....TGAGGTAGTAGGaTGTATAGTT...................................................... 45   | 45; |
| ....cTGAGGTAGTAGGTTGTATAGTT...................................................... 45   | 45; |
| .....TGAG-TAGTAGGTTGTATAGTT...................................................... 45   | 45; |
| .....TGAGGTAGTAGGTTGTATAGTc...................................................... 44   | 44; |
| .....TGAGGTAGTAGGTTGTATAGTTg..................................................... 42   | 42; |
| .....TGAGGTAGTAGGTTGTAaAGTT...................................................... 42   | 42; |
| .....TGcGGTAGTAGGTTGTATAGTT...................................................... 42   | 42; |
| .....TGAGGTAGaAGGTTGTATAGTT...................................................... 40   | 40; |
| .....TGAGGTAGTtGGTTGTATAGTT...................................................... 38   | 38; |
| .....TGAGGT--TAGGTTGTATAGTT...................................................... 37   | 37; |
| .....TGtGGTAGTAGGTTGTATAGTT...................................................... 35   | 35; |
| .....TtAGGTAGTAGGTTGTATAGTT...................................................... 35   | 35; |
| .....TGAGGTAcTAGGTTGTATAGTT...................................................... 34   | 34; |
| .....TGAGGgAGTAGGTTGTATAGTT...................................................... 34   | 34; |
| .....TGAGGTAGTAGGTTGaATAGTT...................................................... 33   | 33; |
| .....TGAGGTAaTAGGTTGTATAGTT...................................................... 32   | 32; |
| .....TGAGGTAGTAGGTTaTATAGTT...................................................... 30   | 30; |
| .....TGAGGTAGTcGGTTGTATAGTT...................................................... 30   | 30; |
| .....TGAGGTAGTAGGTTGTgTAGT....................................................... 28   | 28; |
| .....TGAGGTAGTAGGTTGTATcGTT...................................................... 27   | 27; |
| .....TGAGGTgGTAGGTTGTATAGT....................................................... 27   | 27; |
| .....TGAaGTAGTAGGTTGTATAGTT...................................................... 26   | 26; |
| .....TGAGGcAGTAGGTTGTATAGT....................................................... 26   | 26; |
| .....TaAGGTAGTAGGTTGTATAGT....................................................... 25   | 25; |
| .....TGAGGTAGTgGGTTGTATAGT....................................................... 24   | 24; |
| .....TGAGaTAGTAGGTTGTATAGTT...................................................... 24   | 24; |
| .....TGAGGTAGTAGGTT--ATAGT....................................................... 24   | 24; |
| .....TGAGGTAGTAGGTTGTATAGTaT..................................................... 23   | 23; |
| .....TGAGGTAGTAGGTTGTATAcTT...................................................... 22   | 22; |
| .....TGAGGTAGTAGGTTGTATAGg....................................................... 21   | 21; |
| .....TGAGGTAGTAGGTaGTATAGTT...................................................... 21   | 21; |
| .....TGAGGTAGTA--TTGTATAGT....................................................... 21   | 21; |
| .....TGAtGTAGTAGGTTGTATAGTT...................................................... 21   | 21; |
| .....TGAGGTcGTAGGTTGTATAGTT...................................................... 21   | 21; |
| .....cGAGGTAGTAGGTTGTATAGT....................................................... 21   | 21; |
| .....aGAGGTAGTAGGTTGTATAGT....................................................... 21   | 21; |
| .....TGAGGTAGTAaGTTGTATAGTT...................................................... 20   | 20; |
| .....TGAGGTAtTAGGTTGTATAGT....................................................... 20   | 20; |
| .....TGAGGTAGTAGGgTGTATAGT....................................................... 19   | 19; |
| .....TGAGGTAGTAGGTTcTATAGTT...................................................... 19   | 19; |
| .....TGAGGTtGTAGGTTGTATAGTT...................................................... 19   | 19; |
| .....TGAGGTAGTAGGTTGTATtGTT...................................................... 18   | 18; |
| .....TGAGGTAGTAGGTTGT-TAGTT...................................................... 18   | 18; |
| .....TGAGGTAGTAtGTTGTATAGTT...................................................... 18   | 18; |
| .....TGgGGTAGTAGGTTGTATAGT....................................................... 18   | 18; |
| .....TGAGGTAGTAGGTTGTtTAGT....................................................... 18   | 18; |
| .....TGAGGaAGTAGGTTGTATAGT....................................................... 17   | 17; |
| .....TGAGcTAGTAGGTTGTATAGTT...................................................... 16   | 16; |
| .....TGAGGTAGTAGGTTGTATAaTT...................................................... 16   | 16; |
| .....TGAGGTANTAGGTTGTATAGTTa..................................................... 16   | 16; |
| .....TGAGGTAGTAGGT-GTATAGT....................................................... 16   | 16; |
| .....TGAGGTAGTAGGcTGTATAGT....................................................... 16   | 16; |
| .....TGAGGTAGTAGGTTGTATAGc....................................................... 15   | 15; |
| .....TGAGGTAGTAGGTTGgATAGT....................................................... 15   | 15; |
| .....TGAGGTAGTAGGTTGTATAGaa...................................................... 15   | 15; |
| .....TGAGGTAGcAGGTTGTATAGT....................................................... 14   | 14; |
| .....TGAGGTAGTAGGTTtTATAGTT...................................................... 14   | 14; |
| .....TGAGGTAGTAGGaTGTATAGT....................................................... 13   | 13; |
| .....TGAG-TAGTAGGTTGTATAGT....................................................... 13   | 13; |
| .....TGAGGTAGTAcGTTGTATAGTT...................................................... 12   | 12; |
| .....TGAGGTAGTAGtTTGTATAGT....................................................... 12   | 12; |
| .....TGAGGTAGTAGGTTGTATAtT....................................................... 12   | 12; |
| .....TGAGGTAGTAGGTcGTATAGT....................................................... 12   | 12; |
| .....TGcGGTAGTAGGTTGTATAGT....................................................... 12   | 12; |
| .....TGAcGTAGTAGGTTGTATAGTT...................................................... 12   | 12; |
| .....TGAGGTAGTNGGTTGTATAGTTa..................................................... 12   | 12; |
| .....TGAGGTAGTAGGTTGTAaAGT....................................................... 11   | 11; |
| .....TGAGGTAGTAGGTTGTA--GTTT..................................................... 11   | 11; |
| .....TGAGGTAGTAGGTTaTATAGT....................................................... 11   | 11; |
| .....TGAGtTAGTAGGTTGTATAGTT...................................................... 11   | 11; |
| .....TGAGGTAaTAGGTTGTATAGT....................................................... 11   | 11; |
| .....TcAGGTAGTAGGTTGTATAGTT...................................................... 10   | 10; |
| .....TGAGGTAGTAGGTTNTATAGTTa..................................................... 10   | 10; |
| .....TGAGGTAGTAGGTTGgATAGgT...................................................... 10   | 10; |
| .....TGAGGTAGTAGGTT-TATAGTT...................................................... 10   | 10; |
| .....TGAGGTAGTAGcTTGTATAGT....................................................... 10   | 10; |
| .....TGAGGTAcTAGGTTGTATAGT....................................................... 10   | 10; |
| .....TGAGGT-GTAGGTTGTATAGTT...................................................... 10   | 10; |
| .....TtAGGTAGTAGGTTGTATAGT....................................................... 10   | 10; |
| .....TGAGGTAGTAGGTTGaATAGT....................................................... 10   | 10; |
| .....TGtGGTAGTAGGTTGTATAGT....................................................... 9   | 9; |
| ....cTGAGGTAGTAGGTTGTATAGT....................................................... 9   | 9; |
| .....TGAGGTAGTtGGTTGTATAGT....................................................... 9   | 9; |
| .....TGAGGT--TAGGTTGTATAGT....................................................... 9   | 9; |
| .....TGAGGTAGT-G-TTGTATAGTT...................................................... 9   | 9; |
| .....gGAGGTAGTAGGTTGTATAGTT...................................................... 9   | 9; |
| .....TGAGGgAGTAGGTTGTATAGT....................................................... 8   | 8; |
| .....TGAGGcAGTAGGTTGTATAGTTT..................................................... 8   | 8; |
| ......GAGGTAGTAGGTTGTATAGTTa..................................................... 8   | 8; |
| .....TGAGGTAGTAGGTTGTAT-GT....................................................... 8   | 8; |
| .....TGAGGTAGTAGGTTGTATAaT....................................................... 8   | 8; |
| .....TGAGGTAGaAGGTTGTATAGT....................................................... 8   | 8; |
| .....TGAGaTAGTAGGTTGTATAGT....................................................... 8   | 8; |
| .....TGAGGTAGTAG-TTGTATAGT....................................................... 8   | 8; |
| .....TGAGGTAGTAGGTT--ATAGTTT..................................................... 8   | 8; |
| .....TGAGGTAGTAG-TTGTATAGTTT..................................................... 7   | 7; |
| .....TGAGGTAGTAGGTTGTAcAGT....................................................... 7   | 7; |
| .....NGAGGTAGTAGGTTGTATAGTTa..................................................... 7   | 7; |
| .....TGAGGTAGTAGGTTGTcTAGTTT..................................................... 7   | 7; |
| .....TG-GGTAGTAGGTTGTATAGTT...................................................... 7   | 7; |
| .....TGAGGTAGTAGGTTGTATcGT....................................................... 7   | 7; |
| ......tAGGTAGTAGGTTGTATAGTT...................................................... 7   | 7; |
| .....TGAtGTAGTAGGTTGTATAGT....................................................... 7   | 7; |
| .....TGAGGTAGTAGGTTGTATAGTg...................................................... 7   | 7; |
| .....TG-GGTAGTAGGTTGTATAGT....................................................... 6   | 6; |
| .....TGAaGTAGTAGGTTGTATAGT....................................................... 6   | 6; |
| .....TGAGGTAGTAGGTTGTATAGgTT..................................................... 6   | 6; |
| .....TGAGGTAGTcGGTTGTATAGT....................................................... 6   | 6; |
| .....TGAGGTAGTAGGTTGTATtGT....................................................... 6   | 6; |
| .....TGAGGTAGTAGGTTGTgTAGTTT..................................................... 6   | 6; |
| .....TGAGGTAGT-GGTTGTATAGTT...................................................... 6   | 6; |
| .....TGAGGTAGTAGGgTGgATAGTT...................................................... 6   | 6; |
| .....TGAGGTAGTAGGTgGTATAGTT...................................................... 6   | 6; |
| .....TGAGGTcGTAGGTTGTATAGT....................................................... 6   | 6; |
| .....TGAGG--GTAGGTTGTATAGTT...................................................... 6   | 6; |
| .....TGAGGTAGTAGGTTGgATAGaT...................................................... 5   | 5; |
| .....TGAGGTtGTAGGTTGTATAGT....................................................... 5   | 5; |
| .....TGAGGTAGTAaGTTGTATAGT....................................................... 5   | 5; |
| .....TGAGcTAGTAGGTTGTATAGT....................................................... 5   | 5; |
| .....TGAGGTAGTAGGTTGT-TAGT....................................................... 5   | 5; |
| .....aGAGGTAGTAGGTTGTATAGTTT..................................................... 4   | 4; |
| .....TGAGGTAGTAGGTaGTATAGT....................................................... 4   | 4; |
| .....TGAGGTAGTAGGTTGTtTAGTTT..................................................... 4   | 4; |
| .....TGAcGTAGTAGGTTGTATAGT....................................................... 4   | 4; |
| .....TGAGGTAGcAGGTTGTATAGTTT..................................................... 4   | 4; |
| .....TGAGGTgGTAGGTTGTATAGTTa..................................................... 4   | 4; |
| .....cGAGGTAGTAGGTTGTATAGTTT..................................................... 4   | 4; |
| .....TcAGGTAGTAGGTTGTATAGT....................................................... 4   | 4; |
| .....TGAGGTAGTAGGTTGTATAGaTT..................................................... 4   | 4; |
| .....TtAGGTAGTAGGTTGTATAGTTT..................................................... 4   | 4; |
| .....TGAGGTANgAGGTTGTATAGTTT..................................................... 3   | 3; |
| .....TaAGGTAGTAGGTTGTATAGTTT..................................................... 3   | 3; |
| .....gGAGGTAGTAGGTTGTATAGT....................................................... 3   | 3; |
| .....TGAGGTAGTAGGTTGTATAGcTT..................................................... 3   | 3; |
| .....TGAGGTAtTAGGTTGTATAGTTT..................................................... 3   | 3; |
| .....TGgGGTAGTAGGTTGTATAGTTT..................................................... 3   | 3; |
| .....TGAGGTAGTAcGTTGTATAGT....................................................... 3   | 3; |
| ....cTGAGGTAGTAGGTTGTATAG........................................................ 3   | 3; |
| .....TGAGGTAGTAGGTTcTATAGT....................................................... 3   | 3; |
| .....TGAGGTAGTAGGTTGTATAaa....................................................... 3   | 3; |
| .....TGAGGTAGTAGGgTGTATAGgT...................................................... 3   | 3; |
| .....TGAGGTAGTAGGTTGTcTAGTTa..................................................... 3   | 3; |
| .....TGAGGTgGTAGGTTGTATAGTTT..................................................... 3   | 3; |
| .....TGAGGTAGTAGGTTGTATAcT....................................................... 3   | 3; |
| .....TGAGGTAGTAGGTTGgATAGg....................................................... 3   | 3; |
| .....TGAGGTAGTAGGgTGTATAGTTT..................................................... 3   | 3; |
| .....TGAGGTAGTAGGTTNTATAGTa...................................................... 2   | 2; |
| .....aGAGGTAGTAGGTTGTATAGTTa..................................................... 2   | 2; |
| .....TGAGGTAGTgGGTTGTATAGTTa..................................................... 2   | 2; |
| .....TtAGGTAGTAGGTTGTATAGTTa..................................................... 2   | 2; |
| .....TGAGGTAGTAGcTTGTATAGTTT..................................................... 2   | 2; |
| .....TGAGGTAGTAGGcTGTATAGTTa..................................................... 2   | 2; |
| .....TGAGGTAGTAGtTTGTATAGTTT..................................................... 2   | 2; |
| .....TaAGGTAGTAGGTTGTATAGTTa..................................................... 2   | 2; |
| .....TGAGGTAGTAGGTTaTATAGTTT..................................................... 2   | 2; |
| .....TGAGGTAGTAGGTTGTATAGgTa..................................................... 2   | 2; |
| .....TGAGGTAGTAGGTTGTAgAGTT...................................................... 2   | 2; |
| .....TGAGGTAGTAGGTTGTATANTTa..................................................... 2   | 2; |
| .....TGAGGTAGTAtGTTGTATAGT....................................................... 2   | 2; |
| .....TGAGGTAGTAGGTTGaATAGaT...................................................... 2   | 2; |
| .....TGAGGTAGTgGGTTGTATAGTTT..................................................... 2   | 2; |
| .....TGAGGTAGTAGGTcGTATAGTTT..................................................... 2   | 2; |
| .....TGAGGaAGTAGGTTGTATAGTTT..................................................... 2   | 2; |
| .....TGAGGTAGTAGGTTGgATAGcT...................................................... 2   | 2; |
| .....cGAGGTAGTAGGTTGTATAGTTa..................................................... 2   | 2; |
| .....TGAGGTAGaAGGTTGTATAGgT...................................................... 2   | 2; |
| .....TGcGGTAGTAGGTTGTATAGTTT..................................................... 2   | 2; |
| .....TGAGGTAGaAGGTTGTATAGTTT..................................................... 2   | 2; |
| .....TGAGGTAGTAGGTTGTATAGag...................................................... 2   | 2; |
| ......GAGGTAGgAGGTTGTATAGTTT..................................................... 2   | 2; |
| .....TGAGGagGTAGGTTGTATAGTT...................................................... 2   | 2; |
| .....TGAGGTAGTAGGTTGTtTAGTTa..................................................... 2   | 2; |
| .....TGAGGcAGTAGGTTGTATAGTTa..................................................... 2   | 2; |
| .....TGAGGTAGcAGGTTGTATAGTTa..................................................... 2   | 2; |
| .....TGgGGTAGTAGGTTGTATAGTTa..................................................... 2   | 2; |
| .....TGAGGTAGTcGGTTGTATAGTTT..................................................... 2   | 2; |
| .....TGAGGTAtTAGGTTGTATAGTTa..................................................... 2   | 2; |
| .....TGAGGTAGTAGGTTtTATAGT....................................................... 2   | 2; |
| .....TGAGGTAGTAGGTTGgATAGTTT..................................................... 2   | 2; |
| .....TGAGGgAGTAGGTTGgATAGTT...................................................... 1   | 1; |
| .....TGAGGTAGNAGGTTGTATAGTTa..................................................... 1   | 1; |
| .....TGAGGcAGTAGcTTGTATAGTT...................................................... 1   | 1; |
| .....TGAGGgAGTAGGTTGTATAGTTT..................................................... 1   | 1; |
| .....TGAGGTAGgNGGTTGTATAGTTT..................................................... 1   | 1; |
| .....TGAGGTAGTAGcTTGTATAGTTa..................................................... 1   | 1; |
| .....TGAGGTAGTAGGgTGTATAGTTa..................................................... 1   | 1; |
| .....TGgGGTAGTAGGTTGTATAGgT...................................................... 1   | 1; |
| .....TGAGGTAGTAGGTTGTATttT....................................................... 1   | 1; |
| .....TGAGGTAGTAGGTTGgATAGa....................................................... 1   | 1; |
| .....TGAGGgAGTAGGgTGTATAGTT...................................................... 1   | 1; |
| .....TGAGGTAGTAGcTTGTATAGgT...................................................... 1   | 1; |
| .....TGANGTAGTAGGTTGTATAGTTa..................................................... 1   | 1; |
| .....TGAGGTAGTAGGTTGTATAGTgT..................................................... 1   | 1; |
| .....TGAGGTAGTAGGTTGTAcAGTTT..................................................... 1   | 1; |
| .....TGAGGTAGTAGGTTGTATAGcTa..................................................... 1   | 1; |
| .....TGcGGTAGTAGGTTGTATAGaT...................................................... 1   | 1; |
| .....TGAaGTAGTAGGTTGTATAGTTT..................................................... 1   | 1; |
| .....TGAGGTAGTAGcTTGTATcGTT...................................................... 1   | 1; |
| .....TGAGGTAGTAGNTTGTATAGTTa..................................................... 1   | 1; |
| .....TGAGGaAGTAGGTTGTATAGTTa..................................................... 1   | 1; |
| .....TGAGGTAGTAGtTTGTATAGTTa..................................................... 1   | 1; |
| .....TGAGGTNGTAGGTTGTATAGTTa..................................................... 1   | 1; |
| .....TGAGGTAGTAGcTTGTATAGaT...................................................... 1   | 1; |
| .....TGAGGTAGTAGGTTGTgTAGTTa..................................................... 1   | 1; |
| -------------------------------------------------------------------------------- 9386 |
| -------------------------------------------------------------------------------- 183824 |
| hsa-let-7a-5p(hsa-let-7a-2) AGGTTGAGGTAGTAGGTTGTATAGTTTAGAATTACATCAAGGGAGATAACTGTACAGCCTCCTAGCTTTCCT (((..(((.(((.(((((((((((((.....(..(.....)..)...))))))))))))).))).))).))) (-25.20) \*\*\*\*TGAGGTAGTAGGTTGTATAGTT\*\*\*\*\*\*\*\*\*\*\*\*\*\*\*\*\*\*\*\*\*\*\*\*\*\*\*\*\*\*\*\*\*\*\*\*\*\*\*\*\*\*\*\*\*\* T  M |
| ....TGAGGTAGTAGGTTGTATAGTT...............................................\* 124184   | 124184; |
| ....TGAGGTAGTAGGTTGTATAGT................................................ 30655   | 30655; |
| ....TGAGGTAGTAGGTTGTATAGTTT.............................................. 5293   | 5293; |
| ....TGAGGTAGTAGGTTGTATAG................................................. 3487   | 3487; |
| ....TGAGGTANTAGGTTGTATAGTT............................................... 1278   | 1278; |
| ....TGAGGTAGTNGGTTGTATAGTT............................................... 1159   | 1159; |
| ....TGAGGTAGTAGGTTNTATAGTT............................................... 1017   | 1017; |
| ....TGAGGTAGTAGGTTGTATA.................................................. 974   | 974; |
| ....TGAGGTAGTAGGTTGTAT................................................... 707   | 707; |
| ....NGAGGTAGTAGGTTGTATAGTT............................................... 597   | 597; |
| ....TGAGGTAGTAGNTTGTATAGTT............................................... 391   | 391; |
| ....TGAGGTANTAGGTTGTATAGT................................................ 325   | 325; |
| ....TGAGGTAGTNGGTTGTATAGT................................................ 291   | 291; |
| ....TGAGGTAGTAGGTTNTATAGT................................................ 243   | 243; |
| .....GAGGTAGTAGGTTGTATAGTT............................................... 168   | 168; |
| ....TGAGGTAGTAGGTTGTA.................................................... 166   | 166; |
| ....TGAGGTAGTAGGTTGTNTAGTT............................................... 148   | 148; |
| ....TGAGGTAGNAGGTTGTATAGTT............................................... 139   | 139; |
| ....NGAGGTAGTAGGTTGTATAGT................................................ 135   | 135; |
| ....TGAGGTAGTAGGTTGNATAGTT............................................... 134   | 134; |
| ....TNAGGTAGTAGGTTGTATAGTT............................................... 133   | 133; |
| ....TGAGGNAGTAGGTTGTATAGTT............................................... 128   | 128; |
| ....TGAGNTAGTAGGTTGTATAGTT............................................... 126   | 126; |
| ....TGAGGTAGTAGGTTGTANAGTT............................................... 123   | 123; |
| ....TGNGGTAGTAGGTTGTATAGTT............................................... 121   | 121; |
| ....TGAGGTAGTAGGTNGTATAGTT............................................... 121   | 121; |
| ....TGAGGTAGTAGGTTGTATAGNT............................................... 121   | 121; |
| ....TGAGGTAGTANGTTGTATAGTT............................................... 119   | 119; |
| ....TGAGGTAGTAGGTTGTATANTT............................................... 118   | 118; |
| ....TGAGGTAGTAGGTTGTATAGTN............................................... 118   | 118; |
| ....TGAGGTAGTAGGNTGTATAGTT............................................... 117   | 117; |
| ....TGAGGTNGTAGGTTGTATAGTT............................................... 115   | 115; |
| ....TGANGTAGTAGGTTGTATAGTT............................................... 112   | 112; |
| ....TGAGGTAGTAGGTTGTATNGTT............................................... 110   | 110; |
| ....TGAGGTAGTAGNTTGTATAGT................................................ 82   | 82; |
| ....TGAGGTAGTAGG......................................................... 72   | 72; |
| ....TGAGGTAGTAGGTTGT..................................................... 60   | 60; |
| ......AGGTAGTAGGTTGTATAGTT............................................... 49   | 49; |
| ....TGAGGTANTAGGTTGTATAGTTT.............................................. 46   | 46; |
| .....GAGGTAGTAGGTTGTATAGT................................................ 43   | 43; |
| ....TGAGGTAGTAGGTTGTNTAGT................................................ 42   | 42; |
| ....TGAGGTAGTNGGTTGTATAGTTT.............................................. 41   | 41; |
| ....TGAGGTAGNAGGTTGTATAGT................................................ 38   | 38; |
| ....TGAGGTAGTAGGTTNTATAGTTT.............................................. 35   | 35; |
| ....TGAGGTAGTAGGTTGTATAGTTTA............................................. 35   | 35; |
| ....TNAGGTAGTAGGTTGTATAGT................................................ 34   | 34; |
| ....TGAGGTAGTAGGTTGNATAGT................................................ 33   | 33; |
| ....TGAGGTANTAGGTTGTATAG................................................. 33   | 33; |
| ....TGAGGTAGTAGGNTGTATAGT................................................ 33   | 33; |
| ....TGAGGTAGTAGGTTNTATAG................................................. 33   | 33; |
| ....TGAGGTAGTNGGTTGTATAG................................................. 31   | 31; |
| ....TGAGGTAGTAGGTNGTATAGT................................................ 31   | 31; |
| ....TGAGGTAGTAGGTTGTATAGN................................................ 30   | 30; |
| ....TGAGGTAGTAGGTTGTATNGT................................................ 29   | 29; |
| ....NGAGGTAGTAGGTTGTATAGTTT.............................................. 29   | 29; |
| ....TGAGGTAGTAGGTTGTATANT................................................ 28   | 28; |
| ....NGAGGTAGTAGGTTGTATAG................................................. 28   | 28; |
| ....TGAGGNAGTAGGTTGTATAGT................................................ 27   | 27; |
| ....TGAGGTAGTANGTTGTATAGT................................................ 27   | 27; |
| ....TGNGGTAGTAGGTTGTATAGT................................................ 25   | 25; |
| ...TTGAGGTAGTAGGTTGTATAGT................................................ 24   | 24; |
| ....TGAGNTAGTAGGTTGTATAGT................................................ 23   | 23; |
| ....TGAGGTAGTAGGTTGTANAGT................................................ 19   | 19; |
| ....TGANGTAGTAGGTTGTATAGT................................................ 19   | 19; |
| ....TGAGGTNGTAGGTTGTATAGT................................................ 18   | 18; |
| .....GAGGTAGTAGGTTGTATAGTTT.............................................. 15   | 15; |
| ....NGAGGTAGTAGGTTGTATA.................................................. 13   | 13; |
| ....TGAGGTAGTAGGTTG...................................................... 11   | 11; |
| ......AGGTAGTAGGTTGTATAGT................................................ 11   | 11; |
| ....TGAGGTAGTAGGTTGTATNGTTT.............................................. 10   | 10; |
| ....TGAGGTAGTAGNTTGTATAGTTT.............................................. 10   | 10; |
| ...TTGAGGTAGTAGGTTGTATAG................................................. 9   | 9; |
| ....TGAGGTAGTAGGTTNTAT................................................... 9   | 9; |
| ....TGAGGTAGTAGGTTNTATA.................................................. 8   | 8; |
| ....TGAGGTAGNAGGTTGTATAGTTT.............................................. 8   | 8; |
| ....TGAGGTAGTAGGTTGTATAGTNT.............................................. 7   | 7; |
| ....TGNGGTAGTAGGTTGTATAGTTT.............................................. 7   | 7; |
| ....TGAGGTAGTNGGTTGTATA.................................................. 7   | 7; |
| ....TGAGGTANTAGGTTGTAT................................................... 7   | 7; |
| ....TGAGGTAGTAGNTTGTATAG................................................. 7   | 7; |
| ....TGNGGTAGTAGGTTGTATAG................................................. 7   | 7; |
| ....TGAGGTAGTAGGTT....................................................... 6   | 6; |
| ....TGAGGTAGTAGGTTGTATAGTTN.............................................. 6   | 6; |
| ....TGAGNTAGTAGGTTGTATAGTTT.............................................. 5   | 5; |
| ....TGAGGTANTAGGTTGTATA.................................................. 5   | 5; |
| ....TGAGGTAGTAGGTTGTNTAGTTT.............................................. 5   | 5; |
| ....TGAGGTAGTAGGTTGNATAGTTT.............................................. 5   | 5; |
| ....TGAGGTAGTAGGT........................................................ 4   | 4; |
| ......AGGTAGTAGGTTGTATAGTTT.............................................. 3   | 3; |
| .....GAGGTAGTAGGTTGTATAG................................................. 3   | 3; |
| ....TGAGGTAGTAG.......................................................... 1   | 1; |
| ------------------------------------------------------------------------ 174459 |
| ....TGAGGTAGTAGGTTGTATAGTTa.............................................. 3567   | 3567; |
| ....TGAGGTAGgAGGTTGTATAGTTT.............................................. 475   | 475; |
| ....TGAGGTAGTAGGTTGTATAGTa............................................... 345   | 345; |
| ....TGAGGTAGTAGGTTGTA--GTT............................................... 242   | 242; |
| ....TGAGGTAGTAGGTTGTcTAGTT............................................... 164   | 164; |
| ....TGAGGTgGTAGGTTGTATAGTT............................................... 130   | 130; |
| ....TGAGGTAGTA--TTGTATAGTT............................................... 126   | 126; |
| ....TGAGGcAGTAGGTTGTATAGTT............................................... 115   | 115; |
| ....TGAGGTAGTAGGTTGTgTAGTT............................................... 114   | 114; |
| ....TGAGGTAGTAGGTTGTATAGTaa.............................................. 111   | 111; |
| ....TGgGGTAGTAGGTTGTATAGTT............................................... 101   | 101; |
| ....aGAGGTAGTAGGTTGTATAGTT............................................... 98   | 98; |
| ....TaAGGTAGTAGGTTGTATAGTT............................................... 97   | 97; |
| ....cGAGGTAGTAGGTTGTATAGTT............................................... 86   | 86; |
| ....TGAGGTAGTAGtTTGTATAGTT............................................... 81   | 81; |
| ....TGAGGTAGTAGGTTGTATAGgT............................................... 78   | 78; |
| ....TGAGGTAGTgGGTTGTATAGTT............................................... 74   | 74; |
| ....TGAGGTAGTAGcTTGTATAGTT............................................... 73   | 73; |
| ....TGAGGTAtTAGGTTGTATAGTT............................................... 73   | 73; |
| ....TGAGGTAGTAGGTTGTATAGcT............................................... 73   | 73; |
| ...TT-AGGTAGTAGGTTGTATAGTT............................................... 71   | 71; |
| ....TGAGGTAGTAGGTTGTATAGa................................................ 70   | 70; |
| ....TGAGGTAGTAGGTTGTATAGTTc.............................................. 70   | 70; |
| ....TGAGGTAGTAGGTTGTtTAGTT............................................... 69   | 69; |
| ....TGAGGTAGTAGGT-GTATAGTT............................................... 69   | 69; |
| ....TGAGGTAGTAGGcTGTATAGTT............................................... 68   | 68; |
| ....TGAGGTAGTAGGTTGTATAtTT............................................... 67   | 67; |
| ....TGAGGTAGTAG-TTGTATAGTT............................................... 67   | 67; |
| ....TGAGGaAGTAGGTTGTATAGTT............................................... 65   | 65; |
| ....TGAGGTAGTAGGgTGTATAGTT............................................... 65   | 65; |
| ....TGAGGTAGTAGGTT--ATAGTT............................................... 62   | 62; |
| ....TGAGGTAGTAGGTcGTATAGTT............................................... 59   | 59; |
| ....TGAGGTAGcAGGTTGTATAGTT............................................... 56   | 56; |
| ....TGAGGTAGTAGGTTGgATAGTT............................................... 54   | 54; |
| ....TGAGGTAGTAGGTTGTATAGaT............................................... 53   | 53; |
| ....TGAGGTAGTAGGTTGTAcAGTT............................................... 47   | 47; |
| ....TGAGGTAGTAGGaTGTATAGTT............................................... 45   | 45; |
| ...cTGAGGTAGTAGGTTGTATAGTT............................................... 45   | 45; |
| ....TGAG-TAGTAGGTTGTATAGTT............................................... 45   | 45; |
| ....TGAGGTAGTAGGTTGTcTAGT................................................ 45   | 45; |
| ....TGAGGTAGTAGGTTGTATAGTc............................................... 44   | 44; |
| ....TGAGGTAGTAGGTTGTAaAGTT............................................... 42   | 42; |
| ....TGAGGTAGTAGGTTGTATAGTTg.............................................. 42   | 42; |
| ....TGcGGTAGTAGGTTGTATAGTT............................................... 42   | 42; |
| ....TGAGGTAGaAGGTTGTATAGTT............................................... 40   | 40; |
| ....TGAGGTAGTtGGTTGTATAGTT............................................... 38   | 38; |
| ....TGAGGT--TAGGTTGTATAGTT............................................... 37   | 37; |
| ....TGtGGTAGTAGGTTGTATAGTT............................................... 35   | 35; |
| ....TGAGGTAcTAGGTTGTATAGTT............................................... 34   | 34; |
| ....TGAGGgAGTAGGTTGTATAGTT............................................... 34   | 34; |
| ....TGAGGTAGTAGGTTGaATAGTT............................................... 33   | 33; |
| ....TGAGGTAaTAGGTTGTATAGTT............................................... 32   | 32; |
| ....TGAGGTAGTcGGTTGTATAGTT............................................... 30   | 30; |
| ....TGAGGTAGTAGGTTaTATAGTT............................................... 30   | 30; |
| ....TGAGGTAGTAGGTTGTgTAGT................................................ 28   | 28; |
| ....TGAGGTgGTAGGTTGTATAGT................................................ 27   | 27; |
| ....TGAGGTAGTAGGTTGTATcGTT............................................... 27   | 27; |
| ....TGAGGcAGTAGGTTGTATAGT................................................ 26   | 26; |
| ....TGAaGTAGTAGGTTGTATAGTT............................................... 26   | 26; |
| ....TaAGGTAGTAGGTTGTATAGT................................................ 25   | 25; |
| ....TGAGaTAGTAGGTTGTATAGTT............................................... 24   | 24; |
| ....TGAGGTAGTAGGTT--ATAGT................................................ 24   | 24; |
| ....TGAGGTAGTgGGTTGTATAGT................................................ 24   | 24; |
| ....TGAGGTAGTAGGTTGTATAGTaT.............................................. 23   | 23; |
| ....TGAGGTAGTAGGTTGTATAcTT............................................... 22   | 22; |
| ....TGAGGTAGTAGGTTGTATAGg................................................ 21   | 21; |
| ....TGAGGTcGTAGGTTGTATAGTT............................................... 21   | 21; |
| ....TGAGGTAGTAGGTaGTATAGTT............................................... 21   | 21; |
| ....TGAtGTAGTAGGTTGTATAGTT............................................... 21   | 21; |
| ....aGAGGTAGTAGGTTGTATAGT................................................ 21   | 21; |
| ...TT-AGGTAGTAGGTTGTATAGT................................................ 21   | 21; |
| ....TGAGGTAGTA--TTGTATAGT................................................ 21   | 21; |
| ....cGAGGTAGTAGGTTGTATAGT................................................ 21   | 21; |
| ....TGAGGTAtTAGGTTGTATAGT................................................ 20   | 20; |
| ....TGAGGTAGTAaGTTGTATAGTT............................................... 20   | 20; |
| ....TGAGGTAGTAGGgTGTATAGT................................................ 19   | 19; |
| ....TGAGGTAGTAGGTTcTATAGTT............................................... 19   | 19; |
| ....TGAGGTtGTAGGTTGTATAGTT............................................... 19   | 19; |
| ....TGgGGTAGTAGGTTGTATAGT................................................ 18   | 18; |
| ....TGAGGTAGTAGGTTGT-TAGTT............................................... 18   | 18; |
| ....TGAGGTAGTAGGTTGTtTAGT................................................ 18   | 18; |
| ....TGAGGTAGTAtGTTGTATAGTT............................................... 18   | 18; |
| ....TGAGGTAGTAGGTTGTATtGTT............................................... 18   | 18; |
| ....TGAGGaAGTAGGTTGTATAGT................................................ 17   | 17; |
| ....TGAGcTAGTAGGTTGTATAGTT............................................... 16   | 16; |
| ....TGAGGTAGTAGGT-GTATAGT................................................ 16   | 16; |
| ....TGAGGTAGTAGGTTGTATAaTT............................................... 16   | 16; |
| ....TGAGGTAGTAGGcTGTATAGT................................................ 16   | 16; |
| ....TGAGGTANTAGGTTGTATAGTTa.............................................. 16   | 16; |
| ....TGAGGTAGTAGGTTGTATAGc................................................ 15   | 15; |
| ....TGAGGTAGTAGGTTGTATAGaa............................................... 15   | 15; |
| ....TGAGGTAGTAGGTTGgATAGT................................................ 15   | 15; |
| ....TGAGGTAGTAGGTTtTATAGTT............................................... 14   | 14; |
| ....TGAGGTAGcAGGTTGTATAGT................................................ 14   | 14; |
| ....TGAG-TAGTAGGTTGTATAGT................................................ 13   | 13; |
| ....TGAGGTAGTAGGaTGTATAGT................................................ 13   | 13; |
| ....TGcGGTAGTAGGTTGTATAGT................................................ 12   | 12; |
| ....TGAGGTAGTAcGTTGTATAGTT............................................... 12   | 12; |
| ....TGAGGTAGTAGGTTGTATAtT................................................ 12   | 12; |
| ..tTTGAGGTAGTAGGTTGTATAG................................................. 12   | 12; |
| ....TGAGGTAGTAGGTcGTATAGT................................................ 12   | 12; |
| ....TGAcGTAGTAGGTTGTATAGTT............................................... 12   | 12; |
| ....TGAGGTAGTNGGTTGTATAGTTa.............................................. 12   | 12; |
| ....TGAGGTAGTAGtTTGTATAGT................................................ 12   | 12; |
| ....TGAGGTAGTAGGTTGTA--GTTT.............................................. 11   | 11; |
| ....TGAGGTAGTAGGTTGTAaAGT................................................ 11   | 11; |
| ....TGAGGTAaTAGGTTGTATAGT................................................ 11   | 11; |
| ....TGAGGTAGTAGGTTaTATAGT................................................ 11   | 11; |
| ....TGAGtTAGTAGGTTGTATAGTT............................................... 11   | 11; |
| ....TGAGGTAGTAGcTTGTATAGT................................................ 10   | 10; |
| ....TGAGGTAGTAGGTTGgATAGgT............................................... 10   | 10; |
| ....TGAGGTAcTAGGTTGTATAGT................................................ 10   | 10; |
| ....TcAGGTAGTAGGTTGTATAGTT............................................... 10   | 10; |
| ....TGAGGTAGTAGGTTNTATAGTTa.............................................. 10   | 10; |
| ....TGAGGTAGTAGGTT-TATAGTT............................................... 10   | 10; |
| ....TGAGGTAGTAGGTTGaATAGT................................................ 10   | 10; |
| ....TGAGGT-GTAGGTTGTATAGTT............................................... 10   | 10; |
| ....TGtGGTAGTAGGTTGTATAGT................................................ 9   | 9; |
| ....TGAGGTAGTtGGTTGTATAGT................................................ 9   | 9; |
| ....TGAGGTAGT-G-TTGTATAGTT............................................... 9   | 9; |
| ...cTGAGGTAGTAGGTTGTATAGT................................................ 9   | 9; |
| ....TGAGGT--TAGGTTGTATAGT................................................ 9   | 9; |
| ....gGAGGTAGTAGGTTGTATAGTT............................................... 9   | 9; |
| .....GAGGTAGTAGGTTGTATAGTTa.............................................. 8   | 8; |
| ....TGAGGcAGTAGGTTGTATAGTTT.............................................. 8   | 8; |
| ....TGAGGTAGTAGGTTGTAT-GT................................................ 8   | 8; |
| ....TGAGGTAGTAG-TTGTATAGT................................................ 8   | 8; |
| ....TGAGGTAGTAGGTTGTATAaT................................................ 8   | 8; |
| ....TGAGGTAGaAGGTTGTATAGT................................................ 8   | 8; |
| ....TGAGaTAGTAGGTTGTATAGT................................................ 8   | 8; |
| ....TGAGGgAGTAGGTTGTATAGT................................................ 8   | 8; |
| ....TGAGGTAGTAGGTT--ATAGTTT.............................................. 8   | 8; |
| ....TGAGGTAGTAG-TTGTATAGTTT.............................................. 7   | 7; |
| ....NGAGGTAGTAGGTTGTATAGTTa.............................................. 7   | 7; |
| ....TGAGGTAGTAGGTTGTATcGT................................................ 7   | 7; |
| ....TGAGGTAGTAGGTTGTATAGTg............................................... 7   | 7; |
| .....tAGGTAGTAGGTTGTATAGTT............................................... 7   | 7; |
| ....TGAtGTAGTAGGTTGTATAGT................................................ 7   | 7; |
| ....TGAGGTAGTAGGTTGTcTAGTTT.............................................. 7   | 7; |
| ..GTTGAGGTAGTAGaTTGTATA.................................................. 7   | 7; |
| ....TG-GGTAGTAGGTTGTATAGTT............................................... 7   | 7; |
| ....TGAGGTAGTAGGTTGTAcAGT................................................ 7   | 7; |
| ....TGAGGTAGTAGGgTGgATAGTT............................................... 6   | 6; |
| ....TGAGGTAGT-GGTTGTATAGTT............................................... 6   | 6; |
| ....TGAGGTcGTAGGTTGTATAGT................................................ 6   | 6; |
| ....TGAGGTAGTAGGTTGTATtGT................................................ 6   | 6; |
| ....TGAaGTAGTAGGTTGTATAGT................................................ 6   | 6; |
| ....TGAGGTAGTcGGTTGTATAGT................................................ 6   | 6; |
| ....TGAGGTAGTAGGTTGTATAGgTT.............................................. 6   | 6; |
| ....TGAGGTAGTAGGTTGTgTAGTTT.............................................. 6   | 6; |
| ....TG-GGTAGTAGGTTGTATAGT................................................ 6   | 6; |
| ....TGAGGTAGTAGGTgGTATAGTT............................................... 6   | 6; |
| ....TGAGG--GTAGGTTGTATAGTT............................................... 6   | 6; |
| ....TGAGGTAGTAGGTTGgATAGaT............................................... 5   | 5; |
| ....TGAGGTAGTAGGTTGT-TAGT................................................ 5   | 5; |
| ....TGAGcTAGTAGGTTGTATAGT................................................ 5   | 5; |
| ....TGAGGTtGTAGGTTGTATAGT................................................ 5   | 5; |
| ....TGAGGTAGTAaGTTGTATAGT................................................ 5   | 5; |
| ....TGAGGTAGcAGGTTGTATAGTTT.............................................. 4   | 4; |
| ....aGAGGTAGTAGGTTGTATAGTTT.............................................. 4   | 4; |
| ....TGAGGTAGTAGGTTGTATAGaTT.............................................. 4   | 4; |
| ....TcAGGTAGTAGGTTGTATAGT................................................ 4   | 4; |
| ....TGAcGTAGTAGGTTGTATAGT................................................ 4   | 4; |
| ....TGAGGTAGTAGGTaGTATAGT................................................ 4   | 4; |
| ....TGAGGTAGTAGGTTGTtTAGTTT.............................................. 4   | 4; |
| ....TGAGGTgGTAGGTTGTATAGTTa.............................................. 4   | 4; |
| ....cGAGGTAGTAGGTTGTATAGTTT.............................................. 4   | 4; |
| ....TGAGGTAGTAGGgTGTATAGgT............................................... 3   | 3; |
| ....TGAGGTAGTAGGgTGTATAGTTT.............................................. 3   | 3; |
| ....TGAGGTgGTAGGTTGTATAGTTT.............................................. 3   | 3; |
| ....TGAGGTAGTAGGTTGgATAGg................................................ 3   | 3; |
| ...cTGAGGTAGTAGGTTGTATAG................................................. 3   | 3; |
| ....TGAGGTANgAGGTTGTATAGTTT.............................................. 3   | 3; |
| ....TGAGGTAGTAGGTTGTcTAGTTa.............................................. 3   | 3; |
| ....TGgGGTAGTAGGTTGTATAGTTT.............................................. 3   | 3; |
| ....TGAGGTAGTAGGTTGTATAGcTT.............................................. 3   | 3; |
| ....TGAGGTAGTAGGTTGTATAaa................................................ 3   | 3; |
| ....TGAGGTAGTAcGTTGTATAGT................................................ 3   | 3; |
| ....gGAGGTAGTAGGTTGTATAGT................................................ 3   | 3; |
| ....TaAGGTAGTAGGTTGTATAGTTT.............................................. 3   | 3; |
| ....TGAGGTAGTAGGTTGTATAcT................................................ 3   | 3; |
| ....TGAGGTAtTAGGTTGTATAGTTT.............................................. 3   | 3; |
| ....TGAGGTAGTAGGTTcTATAGT................................................ 3   | 3; |
| ....TGAGGTAGTAGGcTGTATAGTTa.............................................. 2   | 2; |
| ....TGAGGTAGTAGGTTtTATAGT................................................ 2   | 2; |
| ....TGAGGTAGTAGGTTGgATAGcT............................................... 2   | 2; |
| ....TGAGGTAGTAGGTTGTAgAGTT............................................... 2   | 2; |
| ....TGAGGTAGTAGGTTGTATANTTa.............................................. 2   | 2; |
| ....TGAGGcAGTAGGTTGTATAGTTa.............................................. 2   | 2; |
| ....TGAGGTAGTgGGTTGTATAGTTT.............................................. 2   | 2; |
| ....TGAGGTAGTAGGTTNTATAGTa............................................... 2   | 2; |
| ....TGAGGTAGTAGGTTGgATAGTTT.............................................. 2   | 2; |
| ....TGAGGTAGTAGGTTaTATAGTTT.............................................. 2   | 2; |
| ....TGAGGaAGTAGGTTGTATAGTTT.............................................. 2   | 2; |
| ....TGAGGTAGTAGGTTGTATAGgTa.............................................. 2   | 2; |
| ....TGAGGTAGTAGcTTGTATAGTTT.............................................. 2   | 2; |
| ....TGAGGTAGTAGGTTGaATAGaT............................................... 2   | 2; |
| ....cGAGGTAGTAGGTTGTATAGTTa.............................................. 2   | 2; |
| ....TGAGGTAGTAGGTcGTATAGTTT.............................................. 2   | 2; |
| ....TGAGGTAGTAGGTTGTtTAGTTa.............................................. 2   | 2; |
| ....TGcGGTAGTAGGTTGTATAGTTT.............................................. 2   | 2; |
| ....TGAGGTAGcAGGTTGTATAGTTa.............................................. 2   | 2; |
| ....TGAGGTAGTAGtTTGTATAGTTT.............................................. 2   | 2; |
| ....TGAGGTAGTcGGTTGTATAGTTT.............................................. 2   | 2; |
| ....TGAGGTAGaAGGTTGTATAGgT............................................... 2   | 2; |
| ....TGAGGTAGTgGGTTGTATAGTTa.............................................. 2   | 2; |
| ....TGAGGTAtTAGGTTGTATAGTTa.............................................. 2   | 2; |
| ....TGAGGTAGaAGGTTGTATAGTTT.............................................. 2   | 2; |
| ....aGAGGTAGTAGGTTGTATAGTTa.............................................. 2   | 2; |
| ....TGgGGTAGTAGGTTGTATAGTTa.............................................. 2   | 2; |
| ....TaAGGTAGTAGGTTGTATAGTTa.............................................. 2   | 2; |
| ....TGAGGTAGTAtGTTGTATAGT................................................ 2   | 2; |
| ....TGAGGTAGTAGGTTGTATAGag............................................... 2   | 2; |
| ....TGAGGagGTAGGTTGTATAGTT............................................... 2   | 2; |
| .....GAGGTAGgAGGTTGTATAGTTT.............................................. 2   | 2; |
| ....TGAGGTAGTAGcTTGTATcGTT............................................... 1   | 1; |
| ....TGAGGTAGTAGcTTGTATAGTTa.............................................. 1   | 1; |
| ....TGcGGTAGTAGGTTGTATAGaT............................................... 1   | 1; |
| ....TGAGGTAGgNGGTTGTATAGTTT.............................................. 1   | 1; |
| ....TGAGGTAGTAGGTTGTAcAGTTT.............................................. 1   | 1; |
| ....TGAGGaAGTAGGTTGTATAGTTa.............................................. 1   | 1; |
| ....TGAGGTNGTAGGTTGTATAGTTa.............................................. 1   | 1; |
| ....TGAGGgAGTAGGgTGTATAGTT............................................... 1   | 1; |
| ....TGAaGTAGTAGGTTGTATAGTTT.............................................. 1   | 1; |
| ....TGAGGTAGTAGGTTGgATAGa................................................ 1   | 1; |
| ....TGAGGcAGTAGcTTGTATAGTT............................................... 1   | 1; |
| ....TGAGGTAGTAGtTTGTATAGTTa.............................................. 1   | 1; |
| ....TGAGGgAGTAGGTTGTATAGTTT.............................................. 1   | 1; |
| ....TGAGGTAGTAGcTTGTATAGaT............................................... 1   | 1; |
| ....TGAGGTAGTAGGTTGTgTAGTTa.............................................. 1   | 1; |
| ....TGAGGTAGTAGGTTGTATAGTgT.............................................. 1   | 1; |
| ....TGAGGTAGNAGGTTGTATAGTTa.............................................. 1   | 1; |
| ....TGAGGgAGTAGGTTGgATAGTT............................................... 1   | 1; |
| ....TGAGGTAGTAGGgTGTATAGTTa.............................................. 1   | 1; |
| ....TGAGGTAGTAGNTTGTATAGTTa.............................................. 1   | 1; |
| ....TGAGGTAGTAGGTTGTATAGcTa.............................................. 1   | 1; |
| ....TGAGGTAGTAGcTTGTATAGgT............................................... 1   | 1; |
| ....TGgGGTAGTAGGTTGTATAGgT............................................... 1   | 1; |
| ....TGAGGTAGTAGGTTGTATttT................................................ 1   | 1; |
| ....TGANGTAGTAGGTTGTATAGTTa.............................................. 1   | 1; |
| ------------------------------------------------------------------------ 9446 |
| ------------------------------------------------------------------------ 183905 |
| hsa-let-7a-5p(hsa-let-7a-3) GGGTGAGGTAGTAGGTTGTATAGTTTGGGGCTCTGCCCTGCTATGGGATAACTATACAATCTACTGTCTTTCCT (((.(((..((((((((((((((((((((((...)))))).........))))))))))))))))..))).))) (-34.40) \*\*\*TGAGGTAGTAGGTTGTATAGTT\*\*\*\*\*\*\*\*\*\*\*\*\*\*\*\*\*\*\*\*\*\*\*\*\*\*\*\*\*\*\*\*\*\*\*\*\*\*\*\*\*\*\*\*\*\*\*\*\* T  M |
| ...TGAGGTAGTAGGTTGTATAGTT..................................................\* 124184   | 124184; |
| ...TGAGGTAGTAGGTTGTATAGT................................................... 30655   | 30655; |
| ...TGAGGTAGTAGGTTGTATAGTTT................................................. 5293   | 5293; |
| ...TGAGGTAGTAGGTTGTATAG.................................................... 3487   | 3487; |
| ...TGAGGTANTAGGTTGTATAGTT.................................................. 1278   | 1278; |
| ...TGAGGTAGTNGGTTGTATAGTT.................................................. 1159   | 1159; |
| ...TGAGGTAGTAGGTTNTATAGTT.................................................. 1017   | 1017; |
| ...TGAGGTAGTAGGTTGTATA..................................................... 974   | 974; |
| ...TGAGGTAGTAGGTTGTAT...................................................... 707   | 707; |
| ...NGAGGTAGTAGGTTGTATAGTT.................................................. 597   | 597; |
| ...TGAGGTAGTAGNTTGTATAGTT.................................................. 391   | 391; |
| ...TGAGGTANTAGGTTGTATAGT................................................... 325   | 325; |
| ...TGAGGTAGTNGGTTGTATAGT................................................... 291   | 291; |
| ...TGAGGTAGTAGGTTNTATAGT................................................... 243   | 243; |
| ....GAGGTAGTAGGTTGTATAGTT.................................................. 168   | 168; |
| ...TGAGGTAGTAGGTTGTA....................................................... 166   | 166; |
| ...TGAGGTAGTAGGTTGTNTAGTT.................................................. 148   | 148; |
| ...TGAGGTAGNAGGTTGTATAGTT.................................................. 139   | 139; |
| ...NGAGGTAGTAGGTTGTATAGT................................................... 135   | 135; |
| ...TGAGGTAGTAGGTTGNATAGTT.................................................. 134   | 134; |
| ...TNAGGTAGTAGGTTGTATAGTT.................................................. 133   | 133; |
| ...TGAGGNAGTAGGTTGTATAGTT.................................................. 128   | 128; |
| ...TGAGNTAGTAGGTTGTATAGTT.................................................. 126   | 126; |
| ...TGAGGTAGTAGGTTGTANAGTT.................................................. 123   | 123; |
| ...TGAGGTAGTAGGTNGTATAGTT.................................................. 121   | 121; |
| ...TGAGGTAGTAGGTTGTATAGNT.................................................. 121   | 121; |
| ...TGNGGTAGTAGGTTGTATAGTT.................................................. 121   | 121; |
| ...TGAGGTAGTANGTTGTATAGTT.................................................. 119   | 119; |
| ...TGAGGTAGTAGGTTGTATAGTN.................................................. 118   | 118; |
| ...TGAGGTAGTAGGTTGTATANTT.................................................. 118   | 118; |
| ...TGAGGTAGTAGGNTGTATAGTT.................................................. 117   | 117; |
| ...TGAGGTNGTAGGTTGTATAGTT.................................................. 115   | 115; |
| ...TGANGTAGTAGGTTGTATAGTT.................................................. 112   | 112; |
| ...TGAGGTAGTAGGTTGTATNGTT.................................................. 110   | 110; |
| ..GTGAGGTAGTAGGTTGTATAGT................................................... 86   | 86; |
| ...TGAGGTAGTAGNTTGTATAGT................................................... 82   | 82; |
| ...TGAGGTAGTAGG............................................................ 72   | 72; |
| ...TGAGGTAGTAGGTTGT........................................................ 60   | 60; |
| .....AGGTAGTAGGTTGTATAGTT.................................................. 49   | 49; |
| ...TGAGGTANTAGGTTGTATAGTTT................................................. 46   | 46; |
| ....GAGGTAGTAGGTTGTATAGT................................................... 43   | 43; |
| ...TGAGGTAGTAGGTTGTNTAGT................................................... 42   | 42; |
| ...TGAGGTAGTNGGTTGTATAGTTT................................................. 41   | 41; |
| ...TGAGGTAGNAGGTTGTATAGT................................................... 38   | 38; |
| ...TGAGGTAGTAGGTTNTATAGTTT................................................. 35   | 35; |
| ...TNAGGTAGTAGGTTGTATAGT................................................... 34   | 34; |
| ...TGAGGTANTAGGTTGTATAG.................................................... 33   | 33; |
| ...TGAGGTAGTAGGTTGNATAGT................................................... 33   | 33; |
| ...TGAGGTAGTAGGTTNTATAG.................................................... 33   | 33; |
| ...TGAGGTAGTAGGNTGTATAGT................................................... 33   | 33; |
| ...TGAGGTAGTAGGTNGTATAGT................................................... 31   | 31; |
| ...TGAGGTAGTNGGTTGTATAG.................................................... 31   | 31; |
| ...TGAGGTAGTAGGTTGTATAGN................................................... 30   | 30; |
| ...NGAGGTAGTAGGTTGTATAGTTT................................................. 29   | 29; |
| ...TGAGGTAGTAGGTTGTATNGT................................................... 29   | 29; |
| ...NGAGGTAGTAGGTTGTATAG.................................................... 28   | 28; |
| ...TGAGGTAGTAGGTTGTATANT................................................... 28   | 28; |
| ...TGAGGNAGTAGGTTGTATAGT................................................... 27   | 27; |
| ...TGAGGTAGTANGTTGTATAGT................................................... 27   | 27; |
| ...TGNGGTAGTAGGTTGTATAGT................................................... 25   | 25; |
| ...TGAGNTAGTAGGTTGTATAGT................................................... 23   | 23; |
| ..GTGAGGTAGTAGGTTGTATAG.................................................... 19   | 19; |
| ...TGANGTAGTAGGTTGTATAGT................................................... 19   | 19; |
| ...TGAGGTAGTAGGTTGTANAGT................................................... 19   | 19; |
| ...TGAGGTNGTAGGTTGTATAGT................................................... 18   | 18; |
| ....GAGGTAGTAGGTTGTATAGTTT................................................. 15   | 15; |
| ...NGAGGTAGTAGGTTGTATA..................................................... 13   | 13; |
| ...TGAGGTAGTAGGTTG......................................................... 11   | 11; |
| .....AGGTAGTAGGTTGTATAGT................................................... 11   | 11; |
| ...TGAGGTAGTAGNTTGTATAGTTT................................................. 10   | 10; |
| ...TGAGGTAGTAGGTTGTATNGTTT................................................. 10   | 10; |
| ...TGAGGTAGTAGGTTNTAT...................................................... 9   | 9; |
| .....AGGTAGTAGGTTGTATAGTTTG................................................ 8   | 8; |
| ...TGAGGTAGNAGGTTGTATAGTTT................................................. 8   | 8; |
| ..GTGAGGTAGTAGGTTGTATAGTT.................................................. 8   | 8; |
| ...TGAGGTAGTAGGTTNTATA..................................................... 8   | 8; |
| ...TGAGGTAGTAGGTTGTATAGTNT................................................. 7   | 7; |
| ...TGAGGTANTAGGTTGTAT...................................................... 7   | 7; |
| ...TGAGGTAGTAGNTTGTATAG.................................................... 7   | 7; |
| ...TGNGGTAGTAGGTTGTATAG.................................................... 7   | 7; |
| ...TGNGGTAGTAGGTTGTATAGTTT................................................. 7   | 7; |
| ...TGAGGTAGTNGGTTGTATA..................................................... 7   | 7; |
| ...TGAGGTAGTAGGTTGTATAGTTN................................................. 6   | 6; |
| ...TGAGGTAGTAGGTT.......................................................... 6   | 6; |
| ..GTGAGGTAGTAGGTTGTAT...................................................... 6   | 6; |
| ...TGAGNTAGTAGGTTGTATAGTTT................................................. 5   | 5; |
| ...TGAGGTAGTAGGTTGNATAGTTT................................................. 5   | 5; |
| ...TGAGGTAGTAGGTTGTNTAGTTT................................................. 5   | 5; |
| ...TGAGGTANTAGGTTGTATA..................................................... 5   | 5; |
| ...TGAGGTAGTAGGT........................................................... 4   | 4; |
| .....AGGTAGTAGGTTGTATAGTTT................................................. 3   | 3; |
| ....GAGGTAGTAGGTTGTATAG.................................................... 3   | 3; |
| ...TGAGGTAGTAG............................................................. 1   | 1; |
| -------------------------------------------------------------------------- 174518 |
| ...TGAGGTAGTAGGTTGTATAGTTa................................................. 3567   | 3567; |
| ...TGAGGTAGgAGGTTGTATAGTTT................................................. 475   | 475; |
| ...TGAGGTAGTAGGTTGTATAGTa.................................................. 345   | 345; |
| ...TGAGGTAGTAGGTTGTA--GTT.................................................. 242   | 242; |
| ...TGAGGTAGTAGGTTGTcTAGTT.................................................. 164   | 164; |
| ...TGAGGTgGTAGGTTGTATAGTT.................................................. 130   | 130; |
| ...TGAGGTAGTA--TTGTATAGTT.................................................. 126   | 126; |
| ...TGAGGcAGTAGGTTGTATAGTT.................................................. 115   | 115; |
| ...TGAGGTAGTAGGTTGTgTAGTT.................................................. 114   | 114; |
| ...TGAGGTAGTAGGTTGTATAGTaa................................................. 111   | 111; |
| ...TGgGGTAGTAGGTTGTATAGTT.................................................. 101   | 101; |
| ...aGAGGTAGTAGGTTGTATAGTT.................................................. 98   | 98; |
| ...TaAGGTAGTAGGTTGTATAGTT.................................................. 97   | 97; |
| ...cGAGGTAGTAGGTTGTATAGTT.................................................. 86   | 86; |
| ...TGAGGTAGTAGtTTGTATAGTT.................................................. 81   | 81; |
| ...TGAGGTAGTAGGTTGTATAGgT.................................................. 78   | 78; |
| ...TGAGGTAGTgGGTTGTATAGTT.................................................. 74   | 74; |
| ...TGAGGTAtTAGGTTGTATAGTT.................................................. 73   | 73; |
| ...TGAGGTAGTAGcTTGTATAGTT.................................................. 73   | 73; |
| ...TGAGGTAGTAGGTTGTATAGcT.................................................. 73   | 73; |
| ...TGAGGTAGTAGGTTGTATAGTTc................................................. 70   | 70; |
| ...TGAGGTAGTAGGTTGTATAGa................................................... 70   | 70; |
| ...TGAGGTAGTAGGTTGTtTAGTT.................................................. 69   | 69; |
| ...TGAGGTAGTAGGT-GTATAGTT.................................................. 69   | 69; |
| ...TGAGGTAGTAGGcTGTATAGTT.................................................. 68   | 68; |
| ...TGAGGTAGTAG-TTGTATAGTT.................................................. 67   | 67; |
| ...TGAGGTAGTAGGTTGTATAtTT.................................................. 67   | 67; |
| ...TGAGGaAGTAGGTTGTATAGTT.................................................. 65   | 65; |
| ...TGAGGTAGTAGGgTGTATAGTT.................................................. 65   | 65; |
| ...TGAGGTAGTAGGTT--ATAGTT.................................................. 62   | 62; |
| ...TGAGGTAGTAGGTcGTATAGTT.................................................. 59   | 59; |
| ...TGAGGTAGcAGGTTGTATAGTT.................................................. 56   | 56; |
| ...TGAGGTAGTAGGTTGgATAGTT.................................................. 54   | 54; |
| ...TGAGGTAGTAGGTTGTATAGaT.................................................. 53   | 53; |
| ...TGAGGTAGTAGGTTGTAcAGTT.................................................. 47   | 47; |
| ...TGAGGTAGTAGGTTGTcTAGT................................................... 45   | 45; |
| ...TGAGGTAGTAGGaTGTATAGTT.................................................. 45   | 45; |
| ..cTGAGGTAGTAGGTTGTATAGTT.................................................. 45   | 45; |
| ...TGAG-TAGTAGGTTGTATAGTT.................................................. 45   | 45; |
| ...TGAGGTAGTAGGTTGTATAGTc.................................................. 44   | 44; |
| ...TGAGGTAGTAGGTTGTAaAGTT.................................................. 42   | 42; |
| ...TGcGGTAGTAGGTTGTATAGTT.................................................. 42   | 42; |
| ...TGAGGTAGTAGGTTGTATAGTTg................................................. 42   | 42; |
| ...TGAGGTAGaAGGTTGTATAGTT.................................................. 40   | 40; |
| ...TGAGGTAGTtGGTTGTATAGTT.................................................. 38   | 38; |
| ...TGAGGT--TAGGTTGTATAGTT.................................................. 37   | 37; |
| ...TtAGGTAGTAGGTTGTATAGTT.................................................. 35   | 35; |
| ...TGtGGTAGTAGGTTGTATAGTT.................................................. 35   | 35; |
| ...TGAGGgAGTAGGTTGTATAGTT.................................................. 34   | 34; |
| ...TGAGGTAcTAGGTTGTATAGTT.................................................. 34   | 34; |
| ...TGAGGTAGTAGGTTGaATAGTT.................................................. 33   | 33; |
| ...TGAGGTAaTAGGTTGTATAGTT.................................................. 32   | 32; |
| ...TGAGGTAGTAGGTTaTATAGTT.................................................. 30   | 30; |
| ...TGAGGTAGTcGGTTGTATAGTT.................................................. 30   | 30; |
| ...TGAGGTAGTAGGTTGTgTAGT................................................... 28   | 28; |
| ...TGAGGTgGTAGGTTGTATAGT................................................... 27   | 27; |
| ...TGAGGTAGTAGGTTGTATcGTT.................................................. 27   | 27; |
| ...TGAGGcAGTAGGTTGTATAGT................................................... 26   | 26; |
| ...TGAaGTAGTAGGTTGTATAGTT.................................................. 26   | 26; |
| ...TaAGGTAGTAGGTTGTATAGT................................................... 25   | 25; |
| ...TGAGaTAGTAGGTTGTATAGTT.................................................. 24   | 24; |
| ...TGAGGTAGTAGGTT--ATAGT................................................... 24   | 24; |
| ...TGAGGTAGTgGGTTGTATAGT................................................... 24   | 24; |
| ...TGAGGTAGTAGGTTGTATAGTaT................................................. 23   | 23; |
| ...TGAGGTAGTAGGTTGTATAcTT.................................................. 22   | 22; |
| ...TGAtGTAGTAGGTTGTATAGTT.................................................. 21   | 21; |
| ...cGAGGTAGTAGGTTGTATAGT................................................... 21   | 21; |
| ...aGAGGTAGTAGGTTGTATAGT................................................... 21   | 21; |
| ...TGAGGTAGTAGGTaGTATAGTT.................................................. 21   | 21; |
| ...TGAGGTcGTAGGTTGTATAGTT.................................................. 21   | 21; |
| ...TGAGGTAGTA--TTGTATAGT................................................... 21   | 21; |
| ...TGAGGTAGTAGGTTGTATAGg................................................... 21   | 21; |
| ...TGAGGTAGTAaGTTGTATAGTT.................................................. 20   | 20; |
| ...TGAGGTAtTAGGTTGTATAGT................................................... 20   | 20; |
| ...TGAGGTAGTAGGTTcTATAGTT.................................................. 19   | 19; |
| ...TGAGGTtGTAGGTTGTATAGTT.................................................. 19   | 19; |
| ...TGAGGTAGTAGGgTGTATAGT................................................... 19   | 19; |
| ...TGAGGTAGTAGGTTGT-TAGTT.................................................. 18   | 18; |
| ...TGAGGTAGTAtGTTGTATAGTT.................................................. 18   | 18; |
| ...TGAGGTAGTAGGTTGTATtGTT.................................................. 18   | 18; |
| ...TGgGGTAGTAGGTTGTATAGT................................................... 18   | 18; |
| ...TGAGGTAGTAGGTTGTtTAGT................................................... 18   | 18; |
| ...TGAGGaAGTAGGTTGTATAGT................................................... 17   | 17; |
| ...TGAGcTAGTAGGTTGTATAGTT.................................................. 16   | 16; |
| ...TGAGGTANTAGGTTGTATAGTTa................................................. 16   | 16; |
| ...TGAGGTAGTAGGcTGTATAGT................................................... 16   | 16; |
| ...TGAGGTAGTAGGT-GTATAGT................................................... 16   | 16; |
| ...TGAGGTAGTAGGTTGTATAaTT.................................................. 16   | 16; |
| ...TGAGGTAGTAGGTTGTATAGc................................................... 15   | 15; |
| ...TGAGGTAGTAGGTTGgATAGT................................................... 15   | 15; |
| ...TGAGGTAGTAGGTTGTATAGaa.................................................. 15   | 15; |
| ...TGAGGTAGcAGGTTGTATAGT................................................... 14   | 14; |
| ...TGAGGTAGTAGGTTtTATAGTT.................................................. 14   | 14; |
| ...TGAG-TAGTAGGTTGTATAGT................................................... 13   | 13; |
| ...TGAGGTAGTAGGaTGTATAGT................................................... 13   | 13; |
| ...TGAGGTAGTNGGTTGTATAGTTa................................................. 12   | 12; |
| ...TGAGGTAGTAGGTTGTATAtT................................................... 12   | 12; |
| ...TGAGGTAGTAcGTTGTATAGTT.................................................. 12   | 12; |
| ...TGAcGTAGTAGGTTGTATAGTT.................................................. 12   | 12; |
| ...TGcGGTAGTAGGTTGTATAGT................................................... 12   | 12; |
| ...TGAGGTAGTAGtTTGTATAGT................................................... 12   | 12; |
| ...TGAGGTAGTAGGTcGTATAGT................................................... 12   | 12; |
| ...TGAGGTAGTAGGTTaTATAGT................................................... 11   | 11; |
| ...TGAGtTAGTAGGTTGTATAGTT.................................................. 11   | 11; |
| ...TGAGGTAGTAGGTTGTA--GTTT................................................. 11   | 11; |
| ...TGAGGTAaTAGGTTGTATAGT................................................... 11   | 11; |
| ...TGAGGTAGTAGGTTGTAaAGT................................................... 11   | 11; |
| ...TGAGGTAGTAGGTTGaATAGT................................................... 10   | 10; |
| ...TGAGGTAGTAGGTTNTATAGTTa................................................. 10   | 10; |
| ...TGAGGTAGTAGGTTGgATAGgT.................................................. 10   | 10; |
| ...TGAGGTAGTAGGTT-TATAGTT.................................................. 10   | 10; |
| ...TtAGGTAGTAGGTTGTATAGT................................................... 10   | 10; |
| ...TGAGGTAcTAGGTTGTATAGT................................................... 10   | 10; |
| ...TGAGGT-GTAGGTTGTATAGTT.................................................. 10   | 10; |
| ...TcAGGTAGTAGGTTGTATAGTT.................................................. 10   | 10; |
| ...TGAGGTAGTAGcTTGTATAGT................................................... 10   | 10; |
| ...gGAGGTAGTAGGTTGTATAGTT.................................................. 9   | 9; |
| ...TGAGGTAGTtGGTTGTATAGT................................................... 9   | 9; |
| ..cTGAGGTAGTAGGTTGTATAGT................................................... 9   | 9; |
| ...TGAGGT--TAGGTTGTATAGT................................................... 9   | 9; |
| ...TGAGGTAGT-G-TTGTATAGTT.................................................. 9   | 9; |
| ...TGtGGTAGTAGGTTGTATAGT................................................... 9   | 9; |
| ...TGAGaTAGTAGGTTGTATAGT................................................... 8   | 8; |
| ....GAGGTAGTAGGTTGTATAGTTa................................................. 8   | 8; |
| ...TGAGGTAGTAGGTTGTATAaT................................................... 8   | 8; |
| ...TGAGGTAGTAG-TTGTATAGT................................................... 8   | 8; |
| ...TGAGGcAGTAGGTTGTATAGTTT................................................. 8   | 8; |
| ...TGAGGTAGTAGGTT--ATAGTTT................................................. 8   | 8; |
| ...TGAGGTAGaAGGTTGTATAGT................................................... 8   | 8; |
| ...TGAGGTAGTAGGTTGTAT-GT................................................... 8   | 8; |
| ...TGAGGgAGTAGGTTGTATAGT................................................... 8   | 8; |
| ...TGAGGTAGTAGGTTGTATcGT................................................... 7   | 7; |
| ...NGAGGTAGTAGGTTGTATAGTTa................................................. 7   | 7; |
| ...TGAGGTAGTAGGTTGTATAGTg.................................................. 7   | 7; |
| ...TGAGGTAGTAG-TTGTATAGTTT................................................. 7   | 7; |
| ...TG-GGTAGTAGGTTGTATAGTT.................................................. 7   | 7; |
| ...TGAtGTAGTAGGTTGTATAGT................................................... 7   | 7; |
| ...TGAGGTAGTAGGTTGTAcAGT................................................... 7   | 7; |
| ...TGAGGTAGTAGGTTGTcTAGTTT................................................. 7   | 7; |
| ....tAGGTAGTAGGTTGTATAGTT.................................................. 7   | 7; |
| ...TGAGGTAGTAGGgTGgATAGTT.................................................. 6   | 6; |
| ...TGAGGTAGTAGGTgGTATAGTT.................................................. 6   | 6; |
| ...TGAaGTAGTAGGTTGTATAGT................................................... 6   | 6; |
| ...TGAGGTAGTAGGTTGTATtGT................................................... 6   | 6; |
| ...TGAGG--GTAGGTTGTATAGTT.................................................. 6   | 6; |
| ...TGAGGTAGTcGGTTGTATAGT................................................... 6   | 6; |
| ...TGAGGTAGTAGGTTGTgTAGTTT................................................. 6   | 6; |
| ...TGAGGTcGTAGGTTGTATAGT................................................... 6   | 6; |
| ...TGAGGTAGTAGGTTGTATAGgTT................................................. 6   | 6; |
| ...TGAGGTAGT-GGTTGTATAGTT.................................................. 6   | 6; |
| ...TG-GGTAGTAGGTTGTATAGT................................................... 6   | 6; |
| ...TGAGGTAGTAaGTTGTATAGT................................................... 5   | 5; |
| ...TGAGGTtGTAGGTTGTATAGT................................................... 5   | 5; |
| ...TGAGGTAGTAGGTTGgATAGaT.................................................. 5   | 5; |
| ...TGAGGTAGTAGGTTGT-TAGT................................................... 5   | 5; |
| ...TGAGcTAGTAGGTTGTATAGT................................................... 5   | 5; |
| ...aGAGGTAGTAGGTTGTATAGTTT................................................. 4   | 4; |
| ...TGAGGTAGTAGGTaGTATAGT................................................... 4   | 4; |
| ...TcAGGTAGTAGGTTGTATAGT................................................... 4   | 4; |
| ...TGAGGTgGTAGGTTGTATAGTTa................................................. 4   | 4; |
| ...cGAGGTAGTAGGTTGTATAGTTT................................................. 4   | 4; |
| ...TGAcGTAGTAGGTTGTATAGT................................................... 4   | 4; |
| ...TGAGGTAGcAGGTTGTATAGTTT................................................. 4   | 4; |
| ...TGAGGTAGTAGGTTGTtTAGTTT................................................. 4   | 4; |
| ...TGAGGTAGTAGGTTGTATAGaTT................................................. 4   | 4; |
| ...TtAGGTAGTAGGTTGTATAGTTT................................................. 4   | 4; |
| ...TGAGGTAGTAGGTTGgATAGg................................................... 3   | 3; |
| ...TGAGGTAGTAGGTTGTcTAGTTa................................................. 3   | 3; |
| ...gGAGGTAGTAGGTTGTATAGT................................................... 3   | 3; |
| ...TGAGGTAGTAGGTTGTATAaa................................................... 3   | 3; |
| ...TGAGGTAGTAcGTTGTATAGT................................................... 3   | 3; |
| ...TGgGGTAGTAGGTTGTATAGTTT................................................. 3   | 3; |
| ...TGAGGTAGTAGGgTGTATAGgT.................................................. 3   | 3; |
| ...TaAGGTAGTAGGTTGTATAGTTT................................................. 3   | 3; |
| ...TGAGGTAGTAGGTTGTATAGcTT................................................. 3   | 3; |
| ...TGAGGTgGTAGGTTGTATAGTTT................................................. 3   | 3; |
| ...TGAGGTAGTAGGTTcTATAGT................................................... 3   | 3; |
| ...TGAGGTAGTAGGgTGTATAGTTT................................................. 3   | 3; |
| ...TGAGGTANgAGGTTGTATAGTTT................................................. 3   | 3; |
| ...TGAGGTAtTAGGTTGTATAGTTT................................................. 3   | 3; |
| ..cTGAGGTAGTAGGTTGTATAG.................................................... 3   | 3; |
| ...TGAGGTAGTAGGTTGTATAcT................................................... 3   | 3; |
| ...TGAGGTAtTAGGTTGTATAGTTa................................................. 2   | 2; |
| ...TGAGGTAGTgGGTTGTATAGTTT................................................. 2   | 2; |
| ...TGAGGTAGTAGGTTGTAgAGTT.................................................. 2   | 2; |
| ...TGAGGagGTAGGTTGTATAGTT.................................................. 2   | 2; |
| ...cGAGGTAGTAGGTTGTATAGTTa................................................. 2   | 2; |
| ...TGAGGTAGTAGGcTGTATAGTTa................................................. 2   | 2; |
| ...TGAGGTAGTAGGTTaTATAGTTT................................................. 2   | 2; |
| ...TGAGGaAGTAGGTTGTATAGTTT................................................. 2   | 2; |
| ...TGAGGTAGTAGcTTGTATAGTTT................................................. 2   | 2; |
| ...TtAGGTAGTAGGTTGTATAGTTa................................................. 2   | 2; |
| ...TGAGGTAGTcGGTTGTATAGTTT................................................. 2   | 2; |
| ...TGAGGTAGTAGtTTGTATAGTTT................................................. 2   | 2; |
| ...TGAGGTAGTAGGTTGTATAGag.................................................. 2   | 2; |
| ...aGAGGTAGTAGGTTGTATAGTTa................................................. 2   | 2; |
| ...TGAGGTAGTAGGTTGaATAGaT.................................................. 2   | 2; |
| ...TGAGGTAGTAGGTTGTATANTTa................................................. 2   | 2; |
| ....GAGGTAGgAGGTTGTATAGTTT................................................. 2   | 2; |
| ...TGAGGTAGTAGGTTGgATAGTTT................................................. 2   | 2; |
| ...TGAGGTAGTAtGTTGTATAGT................................................... 2   | 2; |
| ...TGAGGTAGTAGGTTGTtTAGTTa................................................. 2   | 2; |
| ...TaAGGTAGTAGGTTGTATAGTTa................................................. 2   | 2; |
| ...TGAGGTAGaAGGTTGTATAGgT.................................................. 2   | 2; |
| ...TGAGGcAGTAGGTTGTATAGTTa................................................. 2   | 2; |
| ...TGcGGTAGTAGGTTGTATAGTTT................................................. 2   | 2; |
| ...TGAGGTAGTAGGTTGTATAGgTa................................................. 2   | 2; |
| ...TGAGGTAGTgGGTTGTATAGTTa................................................. 2   | 2; |
| ...TGgGGTAGTAGGTTGTATAGTTa................................................. 2   | 2; |
| ...TGAGGTAGTAGGTTNTATAGTa.................................................. 2   | 2; |
| ...TGAGGTAGcAGGTTGTATAGTTa................................................. 2   | 2; |
| ...TGAGGTAGTAGGTTGgATAGcT.................................................. 2   | 2; |
| ...TGAGGTAGTAGGTcGTATAGTTT................................................. 2   | 2; |
| ...TGAGGTAGaAGGTTGTATAGTTT................................................. 2   | 2; |
| ...TGAGGTAGTAGGTTtTATAGT................................................... 2   | 2; |
| ...TGANGTAGTAGGTTGTATAGTTa................................................. 1   | 1; |
| ...TGAGGTAGgNGGTTGTATAGTTT................................................. 1   | 1; |
| ...TGAGGTAGTAGGTTGTATttT................................................... 1   | 1; |
| ...TGAGGTAGTAGNTTGTATAGTTa................................................. 1   | 1; |
| ...TGAGGTAGTAGGTTGTgTAGTTa................................................. 1   | 1; |
| ...TGAGGTNGTAGGTTGTATAGTTa................................................. 1   | 1; |
| ...TGAGGTAGTAGGTTGgATAGa................................................... 1   | 1; |
| ...TGAGGTAGTAGcTTGTATAGgT.................................................. 1   | 1; |
| ...TGAGGTAGTAGGTTGTAcAGTTT................................................. 1   | 1; |
| ...TGAGGTAGTAGtTTGTATAGTTa................................................. 1   | 1; |
| ...TGAaGTAGTAGGTTGTATAGTTT................................................. 1   | 1; |
| ...TGAGGTAGTAGcTTGTATAGaT.................................................. 1   | 1; |
| ...TGAGGgAGTAGGTTGTATAGTTT................................................. 1   | 1; |
| ...TGAGGTAGTAGcTTGTATcGTT.................................................. 1   | 1; |
| ...TGAGGTAGNAGGTTGTATAGTTa................................................. 1   | 1; |
| ...TGAGGTAGTAGGgTGTATAGTTa................................................. 1   | 1; |
| ...TGAGGTAGTAGGTTGTATAGTgT................................................. 1   | 1; |
| ...TGAGGcAGTAGcTTGTATAGTT.................................................. 1   | 1; |
| ...TGAGGgAGTAGGgTGTATAGTT.................................................. 1   | 1; |
| ...TGcGGTAGTAGGTTGTATAGaT.................................................. 1   | 1; |
| ...TGAGGTAGTAGcTTGTATAGTTa................................................. 1   | 1; |
| ...TGAGGTAGTAGGTTGTATAGcTa................................................. 1   | 1; |
| ...TGAGGaAGTAGGTTGTATAGTTa................................................. 1   | 1; |
| ...TGgGGTAGTAGGTTGTATAGgT.................................................. 1   | 1; |
| ...TGAGGgAGTAGGTTGgATAGTT.................................................. 1   | 1; |
| -------------------------------------------------------------------------- 9386 |
| -------------------------------------------------------------------------- 183904 |
| hsa-let-7b-3p(hsa-let-7b) CGGGGTGAGGTAGTAGGTTGTGTGGTTTCAGGGCAGTGATGTTGCCCCTCGGAAGATAACTATACAACCTACTGCCTTCCCTG (((((.(((((((((((((((((((((...((((((.....)))))).((....)).)))))))))))))))))))))))))) (-50.60) \*\*\*\*\*\*\*\*\*\*\*\*\*\*\*\*\*\*\*\*\*\*\*\*\*\*\*\*\*\*\*\*\*\*\*\*\*\*\*\*\*\*\*\*\*\*\*\*\*\*\*\*\*\*\*\*\*\*\*CTATACAACCTACTGCCTTCCC\*\* T  M |
| .....TGAGGTAGTAGGTTGTGTGGTT......................................................... 27832   | 27832; |
| .....TGAGGTAGTAGGTTGTGTGGT.......................................................... 11666   | 11666; |
| .....TGAGGTAGTAGGTTGTGTGGTTT........................................................ 3422   | 3422; |
| .....TGAGGTAGTAGGTTGTGTGG........................................................... 2955   | 2955; |
| .....TGAGGTAGTAGGTTGTGTG............................................................ 635   | 635; |
| .....TGAGGTAGTAGGTTGTGT............................................................. 393   | 393; |
| .....TGAGGTAGTAGGTTGTG.............................................................. 144   | 144; |
| .....TGAGGTANTAGGTTGTGTGGTT......................................................... 97   | 97; |
| .....TGAGGTAGTAGGTTNTGTGGTT......................................................... 85   | 85; |
| .....TGAGGTAGTNGGTTGTGTGGTT......................................................... 80   | 80; |
| .....TGAGGTAGTAGG................................................................... 72   | 72; |
| .....TGAGGTAGTAGGTTGT............................................................... 60   | 60; |
| .....NGAGGTAGTAGGTTGTGTGGTT......................................................... 56   | 56; |
| ......GAGGTAGTAGGTTGTGTGGTT......................................................... 44   | 44; |
| .....TGAGGTAGTNGGTTGTGTGGT.......................................................... 38   | 38; |
| .....TGAGGTANTAGGTTGTGTGGT.......................................................... 34   | 34; |
| .....TGAGGTAGTAGGTTNTGTGGT.......................................................... 26   | 26; |
| ......GAGGTAGTAGGTTGTGTGGT.......................................................... 20   | 20; |
| .....NGAGGTAGTAGGTTGTGTGGT.......................................................... 18   | 18; |
| .......AGGTAGTAGGTTGTGTGGTT......................................................... 17   | 17; |
| .....TNAGGTAGTAGGTTGTGTGGTT......................................................... 15   | 15; |
| .....TGAGGTANTAGGTTGTGTGGTTT........................................................ 14   | 14; |
| .....TGAGGTAGTAGGTTNTGTGGTTT........................................................ 13   | 13; |
| .....TGAGGTAGTAGGTTGNGTGGTT......................................................... 12   | 12; |
| .....TGAGGTAGTAGGTTG................................................................ 11   | 11; |
| .....TGAGGTAGTAGNTTGTGTGGTT......................................................... 10   | 10; |
| .....TGAGGTAGTAGGTTGTGTGGNT......................................................... 10   | 10; |
| .....TGAGGTAGTAGGTTGTGTGGTN......................................................... 10   | 10; |
| .....TGAGGTAGTAGGTTGTGNGGTT......................................................... 10   | 10; |
| .....TGAGGTAGTNGGTTGTGTGGTTT........................................................ 10   | 10; |
| .....TGAGNTAGTAGGTTGTGTGGTT......................................................... 9   | 9; |
| .....TGAGGNAGTAGGTTGTGTGGTT......................................................... 9   | 9; |
| .....TGAGGTAGTAGGTTGTNTGGTT......................................................... 9   | 9; |
| .....TGNGGTAGTAGGTTGTGTGGTT......................................................... 9   | 9; |
| .....TGAGGTAGNAGGTTGTGTGGTT......................................................... 9   | 9; |
| .....TGAGGTANTAGGTTGTGTGG........................................................... 8   | 8; |
| .....NGAGGTAGTAGGTTGTGTGG........................................................... 8   | 8; |
| .....TGAGGTAGTAGGTTGTGTGNTT......................................................... 8   | 8; |
| .....TGAGGTAGTANGTTGTGTGGTT......................................................... 8   | 8; |
| .....TGAGGTAGTNGGTTGTGTGG........................................................... 7   | 7; |
| .....TGAGGTNGTAGGTTGTGTGGTT......................................................... 7   | 7; |
| .....TGAGGTAGTAGGTNGTGTGGTT......................................................... 7   | 7; |
| .....NGAGGTAGTAGGTTGTGTGGTTT........................................................ 6   | 6; |
| .....TGNGGTAGTAGGTTGTGTGGT.......................................................... 6   | 6; |
| .....TGAGGTAGTAGGNTGTGTGGT.......................................................... 6   | 6; |
| .....TGAGGTAGTAGNTTGTGTGGT.......................................................... 6   | 6; |
| .....TGAGGTAGTAGGTT................................................................. 6   | 6; |
| .....TGAGGTAGTAGGNTGTGTGGTT......................................................... 6   | 6; |
| .....TGAGGNAGTAGGTTGTGTGGT.......................................................... 5   | 5; |
| .....TGAGGTAGTAGGTTGTGTGGN.......................................................... 5   | 5; |
| ......GAGGTAGTAGGTTGTGTGG........................................................... 5   | 5; |
| .....TGAGGTAGTAGGTTGNGTGGT.......................................................... 5   | 5; |
| .....TGANGTAGTAGGTTGTGTGGTT......................................................... 5   | 5; |
| .....TGAGGTAGTAGGT.................................................................. 4   | 4; |
| .....TGAGGTAGTAG.................................................................... 1   | 1; |
| ----------------------------------------------------------------------------------- 47973 |
| ----------------------------------------------------------------------------------- 47973 |
| hsa-let-7b-5p(hsa-let-7b) CGGGGTGAGGTAGTAGGTTGTGTGGTTTCAGGGCAGTGATGTTGCCCCTCGGAAGATAACTATACAACCTACTGCCTTCCCTG (((((.(((((((((((((((((((((...((((((.....)))))).((....)).)))))))))))))))))))))))))) (-50.60) \*\*\*\*\*TGAGGTAGTAGGTTGTGTGGTT\*\*\*\*\*\*\*\*\*\*\*\*\*\*\*\*\*\*\*\*\*\*\*\*\*\*\*\*\*\*\*\*\*\*\*\*\*\*\*\*\*\*\*\*\*\*\*\*\*\*\*\*\*\*\*\* T  M |
| .....TGAGGTAGTAGGTTGTGTGGTT.........................................................\* 27832   | 27832; |
| .....TGAGGTAGTAGGTTGTGTGGT.......................................................... 11666   | 11666; |
| .....TGAGGTAGTAGGTTGTGTGGTTT........................................................ 3422   | 3422; |
| .....TGAGGTAGTAGGTTGTGTGG........................................................... 2955   | 2955; |
| .....TGAGGTAGTAGGTTGTGTG............................................................ 635   | 635; |
| .....TGAGGTAGTAGGTTGTGT............................................................. 393   | 393; |
| .....TGAGGTAGTAGGTTGTG.............................................................. 144   | 144; |
| .....TGAGGTANTAGGTTGTGTGGTT......................................................... 97   | 97; |
| .....TGAGGTAGTAGGTTNTGTGGTT......................................................... 85   | 85; |
| .....TGAGGTAGTNGGTTGTGTGGTT......................................................... 80   | 80; |
| .....TGAGGTAGTAGG................................................................... 72   | 72; |
| .....TGAGGTAGTAGGTTGT............................................................... 60   | 60; |
| .....NGAGGTAGTAGGTTGTGTGGTT......................................................... 56   | 56; |
| ......GAGGTAGTAGGTTGTGTGGTT......................................................... 44   | 44; |
| .....TGAGGTAGTNGGTTGTGTGGT.......................................................... 38   | 38; |
| .....TGAGGTANTAGGTTGTGTGGT.......................................................... 34   | 34; |
| .....TGAGGTAGTAGGTTNTGTGGT.......................................................... 26   | 26; |
| ......GAGGTAGTAGGTTGTGTGGT.......................................................... 20   | 20; |
| .....NGAGGTAGTAGGTTGTGTGGT.......................................................... 18   | 18; |
| .......AGGTAGTAGGTTGTGTGGTT......................................................... 17   | 17; |
| .....TNAGGTAGTAGGTTGTGTGGTT......................................................... 15   | 15; |
| .....TGAGGTANTAGGTTGTGTGGTTT........................................................ 14   | 14; |
| .....TGAGGTAGTAGGTTNTGTGGTTT........................................................ 13   | 13; |
| .....TGAGGTAGTAGGTTGNGTGGTT......................................................... 12   | 12; |
| .....TGAGGTAGTAGGTTG................................................................ 11   | 11; |
| .....TGAGGTAGTAGNTTGTGTGGTT......................................................... 10   | 10; |
| .....TGAGGTAGTAGGTTGTGTGGNT......................................................... 10   | 10; |
| .....TGAGGTAGTAGGTTGTGTGGTN......................................................... 10   | 10; |
| .....TGAGGTAGTAGGTTGTGNGGTT......................................................... 10   | 10; |
| .....TGAGGTAGTNGGTTGTGTGGTTT........................................................ 10   | 10; |
| .....TGAGNTAGTAGGTTGTGTGGTT......................................................... 9   | 9; |
| .....TGAGGNAGTAGGTTGTGTGGTT......................................................... 9   | 9; |
| .....TGAGGTAGTAGGTTGTNTGGTT......................................................... 9   | 9; |
| .....TGNGGTAGTAGGTTGTGTGGTT......................................................... 9   | 9; |
| .....TGAGGTAGNAGGTTGTGTGGTT......................................................... 9   | 9; |
| .....TGAGGTANTAGGTTGTGTGG........................................................... 8   | 8; |
| .....NGAGGTAGTAGGTTGTGTGG........................................................... 8   | 8; |
| .....TGAGGTAGTAGGTTGTGTGNTT......................................................... 8   | 8; |
| .....TGAGGTAGTANGTTGTGTGGTT......................................................... 8   | 8; |
| .....TGAGGTAGTNGGTTGTGTGG........................................................... 7   | 7; |
| .....TGAGGTNGTAGGTTGTGTGGTT......................................................... 7   | 7; |
| .....TGAGGTAGTAGGTNGTGTGGTT......................................................... 7   | 7; |
| .....NGAGGTAGTAGGTTGTGTGGTTT........................................................ 6   | 6; |
| .....TGNGGTAGTAGGTTGTGTGGT.......................................................... 6   | 6; |
| .....TGAGGTAGTAGGNTGTGTGGT.......................................................... 6   | 6; |
| .....TGAGGTAGTAGNTTGTGTGGT.......................................................... 6   | 6; |
| .....TGAGGTAGTAGGTT................................................................. 6   | 6; |
| .....TGAGGTAGTAGGNTGTGTGGTT......................................................... 6   | 6; |
| .....TGAGGNAGTAGGTTGTGTGGT.......................................................... 5   | 5; |
| .....TGAGGTAGTAGGTTGTGTGGN.......................................................... 5   | 5; |
| ......GAGGTAGTAGGTTGTGTGG........................................................... 5   | 5; |
| .....TGAGGTAGTAGGTTGNGTGGT.......................................................... 5   | 5; |
| .....TGANGTAGTAGGTTGTGTGGTT......................................................... 5   | 5; |
| .....TGAGGTAGTAGGT.................................................................. 4   | 4; |
| .....TGAGGTAGTAG.................................................................... 1   | 1; |
| ----------------------------------------------------------------------------------- 47973 |
| .....TGAGGTAGTAGGTTGTGTGGTTa........................................................ 1769   | 1769; |
| .....TGAGGTAGTAGGTTGTGTGGTa......................................................... 529   | 529; |
| .....TGAGGTAGTAGGTTGTGTGGa.......................................................... 268   | 268; |
| .....TGAGGTAGTAGGTTGTGTGGTaa........................................................ 264   | 264; |
| .....TGAGGTAGTAGGTTGTGTGGTaT........................................................ 223   | 223; |
| .....TGAGGTAGTAGGTTGTGTaGTT......................................................... 114   | 114; |
| .....TGAGGTAGTAGGTTGTGTGGaa......................................................... 88   | 88; |
| .....TGAGGTAGTAGGTTGTGTGGTTc........................................................ 67   | 67; |
| .....TGAGGTAGTAGGTTGTGTGGgT......................................................... 60   | 60; |
| .....TGAGGTAGTAGGTTGTG-GtT.......................................................... 38   | 38; |
| .....TGAGGTAGTAGGTTGTGTGGg.......................................................... 36   | 36; |
| .....TGAGGTAGTAGGTTGTGTGtT.......................................................... 34   | 34; |
| .....TGAGGTAGTAGGTTGTGcGGTT......................................................... 29   | 29; |
| .....TGAGGTAGTAGGTTGTGTaGT.......................................................... 28   | 28; |
| .....TGAGGTAGTAGGTTGgGTGGTT......................................................... 28   | 28; |
| .....TaAGGTAGTAGGTTGTGTGGTT......................................................... 28   | 28; |
| .....TGAGGTgGTAGGTTGTGTGGTT......................................................... 28   | 28; |
| .....TGAGGTAGTAGGTTGTGgGGTT......................................................... 26   | 26; |
| .....TGAGGTAtTAGGTTGTGTGGTT......................................................... 26   | 26; |
| .....TGAGGTAGTAGGTTGTGTGGaT......................................................... 26   | 26; |
| .....TGgGGTAGTAGGTTGTGTGGTT......................................................... 24   | 24; |
| .....TGAGGTAGTAGGTTGcGTGGTT......................................................... 23   | 23; |
| .....cGAGGTAGTAGGTTGTGTGGTT......................................................... 22   | 22; |
| .....TGAGGTAGTAGGTTGTGTGGcT......................................................... 21   | 21; |
| .....TGAGGTAGTAGGTTGTGTGtTT......................................................... 20   | 20; |
| .....TGAGGTAGTgGGTTGTGTGGTT......................................................... 20   | 20; |
| .....TGAGGcAGTAGGTTGTGTGGTT......................................................... 20   | 20; |
| .....TGAGGTAGTAGGcTGTGTGGTT......................................................... 19   | 19; |
| .....TGAGGTAGTAGGTTGTGgGGT.......................................................... 18   | 18; |
| .....TGAGGTAGcAGGTTGTGTGGTT......................................................... 17   | 17; |
| .....TGAGGTAGTAGGTTGgGTGGT.......................................................... 16   | 16; |
| .....TGAGGTAGTAGGTTGTGTGGTTg........................................................ 15   | 15; |
| .....TGAGGTAGTAGGTTGTGTtGTT......................................................... 15   | 15; |
| .....TGAGGTAGTAGGTTGaGTGGTT......................................................... 15   | 15; |
| .....TGAGGTAGTAGGTTGTGTGGTc......................................................... 15   | 15; |
| .....TGAGGTAGgAGGTTGTGTGGTT......................................................... 14   | 14; |
| .....TGAGGaAGTAGGTTGTGTGGTT......................................................... 14   | 14; |
| .....TGAGGTAGTAGGgTGTGTGGTT......................................................... 13   | 13; |
| .....TGAGGTAGTAGGTTGTGaGGTT......................................................... 13   | 13; |
| .....TGAGGTAGTAGGTTGTGTGGgTT........................................................ 12   | 12; |
| .....TGAG-TAGTAGGTTGTGTGGTT......................................................... 11   | 11; |
| .....TGAGGTAGaAGGTTGTGTGGTT......................................................... 11   | 11; |
| .....TGAGGcAGTAGGTTGTGTGGT.......................................................... 11   | 11; |
| .....TGAGGT--TAGGTTGTGTGGTT......................................................... 11   | 11; |
| .....aGAGGTAGTAGGTTGTGTGGTT......................................................... 11   | 11; |
| .....TGAGGTAcTAGGTTGTGTGGTT......................................................... 11   | 11; |
| .....TGAGGTAGTAGaTTGTGTGGTT......................................................... 10   | 10; |
| .....TGAGGTgGTAGGTTGTGTGGT.......................................................... 10   | 10; |
| .....TGAGGTAGcAGGTTGTGTGGT.......................................................... 10   | 10; |
| .....TGAGcTAGTAGGTTGTGTGGTT......................................................... 10   | 10; |
| .....TGAGGTANTAGGTTGTGTGGTTa........................................................ 9   | 9; |
| .....TGAGGTAGTAGGTTGcGTGGT.......................................................... 9   | 9; |
| .....TGAGGTAGTAGGTTGTGcGGT.......................................................... 9   | 9; |
| .....TGAGGTAGTAtGTTGTGTGGTT......................................................... 9   | 9; |
| .....TGAGGTAGTAGGTTGTGTGGTg......................................................... 9   | 9; |
| .....TGAGGTAtTAGGTTGTGTGGT.......................................................... 9   | 9; |
| .....TGAGGTAGTAGGaTGTGTGGTT......................................................... 9   | 9; |
| .....TGAaGTAGTAGGTTGTGTGGTT......................................................... 9   | 9; |
| .....TGAtGTAGTAGGTTGTGTGGTT......................................................... 9   | 9; |
| .....TGAGGTAGTAaGTTGTGTGGTT......................................................... 9   | 9; |
| .....TGAGGTAGTAGGTTGTGTGaa.......................................................... 9   | 9; |
| .....TGAGGgAGTAGGTTGTGTGGTT......................................................... 8   | 8; |
| .....TGAGGTcGTAGGTTGTGTGGTT......................................................... 8   | 8; |
| .....TGAGGTAGTAGGTTGTtTGGTT......................................................... 8   | 8; |
| .....cGAGGTAGTAGGTTGTGTGGT.......................................................... 8   | 8; |
| .....TtAGGTAGTAGGTTGTGTGGTT......................................................... 8   | 8; |
| ....cTGAGGTAGTAGGTTGTGTGGTT......................................................... 8   | 8; |
| .....TGAGGTAGTtGGTTGTGTGGTT......................................................... 8   | 8; |
| .....TGAGGTAGTAGGTTGTcTGGTT......................................................... 8   | 8; |
| .....TGAGGTAGTgGGTTGTGTGGT.......................................................... 8   | 8; |
| .....TGAGtTAGTAGGTTGTGTGGTT......................................................... 8   | 8; |
| .....TGAGGTAGTAGGTcGTGTGGTT......................................................... 8   | 8; |
| .....TGgGGTAGTAGGTTGTGTGGT.......................................................... 8   | 8; |
| .....TGAGGTAGTAGGT-GTGTGGTT......................................................... 7   | 7; |
| .....TGAGGcAGTAGGTTGTGTGGTTT........................................................ 7   | 7; |
| .....TaAGGTAGTAGGTTGTGTGGT.......................................................... 7   | 7; |
| .....TGAGGTAGTAGGcTGTGTGGT.......................................................... 7   | 7; |
| .....TGAGGTtGTAGGTTGTGTGGTT......................................................... 7   | 7; |
| .....TGAGGTAGTNGGTTGTGTGGTTa........................................................ 7   | 7; |
| .....TGAGGTAGTAGGTaGTGTGGTT......................................................... 7   | 7; |
| .....TGAGaTAGTAGGTTGTGTGGT.......................................................... 7   | 7; |
| .....TGtGGTAGTAGGTTGTGTGGTT......................................................... 7   | 7; |
| .....TGcGGTAGTAGGTTGTGTGGTT......................................................... 7   | 7; |
| .....TGAGGTAGTAGGTTGTGTGGc.......................................................... 7   | 7; |
| .....TGAGaTAGTAGGTTGTGTGGTT......................................................... 7   | 7; |
| .....TGAcGTAGTAGGTTGTGTGGTT......................................................... 7   | 7; |
| .....TGAGGTAGTAGGTTGTGaGGT.......................................................... 7   | 7; |
| .....TGAGGTAGTAGGTTGT-TGGTT......................................................... 7   | 7; |
| .....TGAGGTAGTAGGT-GTGTGGT.......................................................... 6   | 6; |
| .....TGAGGTAGTAGGTTaTGTGGTT......................................................... 6   | 6; |
| .....TGAGGTAGTAGGgTGgGTGGT.......................................................... 6   | 6; |
| .....TGAcGTAGTAGGTTGTGTGGT.......................................................... 6   | 6; |
| .....TGAGGTAGTcGGTTGTGTGGT.......................................................... 6   | 6; |
| ....cTGAGGTAGTAGGTTGTGTGGT.......................................................... 6   | 6; |
| .....TGAGGTAGTAGGTTGTGTaGTTT........................................................ 6   | 6; |
| .....TGAGGTAGTAGGTcGTGTGGT.......................................................... 6   | 6; |
| .....TGcGGTAGTAGGTTGTGTGGT.......................................................... 6   | 6; |
| .....TGAGGgAGTAGGTTGTGTGGT.......................................................... 6   | 6; |
| .....TGAGGTAcTAGGTTGTGTGGT.......................................................... 6   | 6; |
| .....TGAGGTAGTAGGTTGgGTGGgT......................................................... 5   | 5; |
| .....TGAGGTAGTAGGTTaTGTGGT.......................................................... 5   | 5; |
| .....TGAGGTAGTAGGTTcTGTGGT.......................................................... 5   | 5; |
| .....TGAGGTAGTAGGTTGaGTGGT.......................................................... 5   | 5; |
| .....TGAGGTAGTAGGTTGTGTtGT.......................................................... 5   | 5; |
| .....TGAGGaAGTAGGTTGTGTGGT.......................................................... 5   | 5; |
| .....TGAGGTAGgAGGTTGTGTGGgT......................................................... 5   | 5; |
| .....TGAGGTAGTcGGTTGTGTGGTT......................................................... 5   | 5; |
| ......GAGGTAGTAGGTTGTGTGGTTa........................................................ 5   | 5; |
| .....TGAtGTAGTAGGTTGTGTGGT.......................................................... 5   | 5; |
| .....TGAGGTAaTAGGTTGTGTGGTT......................................................... 5   | 5; |
| .....TGAGGTAGTAGaTTGTGTGGT.......................................................... 5   | 5; |
| .....TGAGGTAGTAGGTTGTcTGGT.......................................................... 3   | 3; |
| .....TGAGGTAGTAGGTTGTtTGGT.......................................................... 3   | 3; |
| .....TGAGGTAGTAGGTTGTcTGGTTT........................................................ 2   | 2; |
| .....TGAGGTAGTAGGTTGTGTaGTTa........................................................ 1   | 1; |
| ----------------------------------------------------------------------------------- 4596 |
| ----------------------------------------------------------------------------------- 52569 |
| hsa-let-7c(hsa-let-7c) GCATCCGGGTTGAGGTAGTAGGTTGTATGGTTTAGAGTTACACCCTGGGAGTTAACTGTACAACCTTCTAGCTTTCCTTGGAGC ((.((((((..(((.(((.(((((((((((((..((.(..(.....)..).))))))))))))))).))).)))..)))))))) (-33.50) \*\*\*\*\*\*\*\*\*\*TGAGGTAGTAGGTTGTATGGTT\*\*\*\*\*\*\*\*\*\*\*\*\*\*\*\*\*\*\*\*\*\*\*\*\*\*\*\*\*\*\*\*\*\*\*\*\*\*\*\*\*\*\*\*\*\*\*\*\*\*\*\* T  M |
| ..........TGAGGTAGTAGGTTGTATGGTT.....................................................\* 2058   | 2058; |
| ..........TGAGGTAGTAGGTTGTATGGT...................................................... 935   | 935; |
| ..........TGAGGTAGTAGGTTGTAT......................................................... 707   | 707; |
| ..........TGAGGTAGTAGGTTGTATGG....................................................... 191   | 191; |
| ..........TGAGGTAGTAGGTTGTATGGTTT.................................................... 188   | 188; |
| ..........TGAGGTAGTAGGTTGTA.......................................................... 166   | 166; |
| ..........TGAGGTAGTAGGTTGTATNGTT..................................................... 110   | 110; |
| ..........TGAGGTAGTAGG............................................................... 72   | 72; |
| ..........TGAGGTAGTAGGTTGT........................................................... 60   | 60; |
| ..........TGAGGTAGTAGGTTGTATNGT...................................................... 29   | 29; |
| ..........TGAGGTAGTAGGTTGTATG........................................................ 21   | 21; |
| ..........TGAGGTAGTAGGTTG............................................................ 11   | 11; |
| ..........TGAGGTAGTAGGTTGTATNGTTT.................................................... 10   | 10; |
| ..........TGAGGTAGTAGGTTGTNTGGTT..................................................... 9   | 9; |
| ..........TGAGGTAGTAGGTTNTAT......................................................... 9   | 9; |
| ...........GAGGTAGTAGGTTGTATGGTT..................................................... 9   | 9; |
| .........TTGAGGTAGTAGGTTGTATGGT...................................................... 7   | 7; |
| ..........TGAGGTANTAGGTTGTAT......................................................... 7   | 7; |
| ..........TGAGGTAGTAGGTTNTATGGTT..................................................... 7   | 7; |
| ..........TGAGGTAGTNGGTTGTATGGTT..................................................... 6   | 6; |
| ..........TGAGGTAGTAGGTT............................................................. 6   | 6; |
| ..........TGAGGTANTAGGTTGTATGGT...................................................... 5   | 5; |
| ..........TGAGGTAGTAGGT.............................................................. 4   | 4; |
| ..........TGAGGTAGTAG................................................................ 1   | 1; |
| ------------------------------------------------------------------------------------ 4628 |
| ..........TGAGGTAGTAGGTTGTA-GtT...................................................... 242   | 242; |
| ..........TGAGGTAGTAGGTTGTATGGTTa.................................................... 129   | 129; |
| ..........TGAGGTAGTAGaTTGTATGGTT..................................................... 116   | 116; |
| ..........TGAGGTAGTAGGTTGTATGGTa..................................................... 102   | 102; |
| ..........TGAGGTAGTAGGTTGTATGGa...................................................... 56   | 56; |
| ..........TGAGGTAGTAGGTTGT--GGTT..................................................... 38   | 38; |
| ..........TGAGGTAGTAGGTTGTATcGTT..................................................... 27   | 27; |
| ..........TGAGGTAGTAGaTTGTATGGT...................................................... 19   | 19; |
| ..........TGAGGTAGTAGGTTGTATGGTaa.................................................... 19   | 19; |
| ..........TGAGGTAGTAGGTTGTATtGTT..................................................... 18   | 18; |
| ..........TGAGGTAGTAGGTTGTATGGaa..................................................... 16   | 16; |
| ..........TGAGGTAGTAGGTTGTATGGTaT.................................................... 12   | 12; |
| ..........TGAGGTAGTAGGTTGTA-GtTT..................................................... 11   | 11; |
| ..........TGAGGTAGTAGGTTGTcTGGTT..................................................... 8   | 8; |
| ..........TGAGGTAGTAGGTTGTtTGGTT..................................................... 8   | 8; |
| ..........TGAGGTAGTAGGTTGT-TGGTT..................................................... 7   | 7; |
| ..........TGAGGTAGTAGGTTGTATcGT...................................................... 7   | 7; |
| ..........TGAGGTAGgAGGTTGTATGGTT..................................................... 6   | 6; |
| ..........TGAGGTAGTAGGTTGTATtGT...................................................... 6   | 6; |
| ..........TGAGGTAGTAGGTTGTATGGTTg.................................................... 5   | 5; |
| ..........TGAGGTAGTAGaTTGTATGGTTa.................................................... 4   | 4; |
| ..........TGAGGTAGTAGGTTGTcTGGT...................................................... 3   | 3; |
| ..........TGAGGTAGTAGGTTGTtTGGT...................................................... 3   | 3; |
| ..........TGAGGTAGTAGaTTGTATGGTTT.................................................... 2   | 2; |
| ..........TGAGGTAGTAGGTTGTcTGGTTT.................................................... 2   | 2; |
| ..........TGAGGTAGTAGcTTGTATcGTT..................................................... 1   | 1; |
| ..........TGAGGTAGTAGGTTGTATttT...................................................... 1   | 1; |
| ------------------------------------------------------------------------------------ 868 |
| ------------------------------------------------------------------------------------ 5496 |
| hsa-let-7d-3p(hsa-let-7d) CCTAGGAAGAGGTAGTAGGTTGCATAGTTTTAGGGCAGGGATTTTGCCCACAAGGAGGTAACTATACGACCTGCTGCCTTTCTTAGG (((((((.((((((((((((((.((((((...((((((.....)))))).(.....)..)))))).))))))))))))))))))))) (-42.60) \*\*\*\*\*\*\*\*\*\*\*\*\*\*\*\*\*\*\*\*\*\*\*\*\*\*\*\*\*\*\*\*\*\*\*\*\*\*\*\*\*\*\*\*\*\*\*\*\*\*\*\*\*\*\*\*\*\*\*\*\*CTATACGACCTGCTGCCTTTCT\*\*\*\* T  M |
| .......AGAGGTAGTAGGTTGCATAGTT........................................................... 79335   | 79335; |
| .......AGAGGTAGTAGGTTGCATAGT............................................................ 9277   | 9277; |
| .......AGAGGTAGTAGGTTGCATAGTTT.......................................................... 4015   | 4015; |
| .......AGAGGTAGTAGGTTGCATAG............................................................. 1804   | 1804; |
| .......AGAGGTAGTAGGTTGCAT............................................................... 742   | 742; |
| .......AGAGGTAGTAGGTTGCATA.............................................................. 730   | 730; |
| ........GAGGTAGTAGGTTGCATAGTT........................................................... 396   | 396; |
| .......AGAGGTANTAGGTTGCATAGTT........................................................... 295   | 295; |
| .......AGAGGTAGTNGGTTGCATAGTT........................................................... 230   | 230; |
| .......AGAGGTAGTAGGTTNCATAGTT........................................................... 222   | 222; |
| .............................................................CTATACGACCTGCTGCCTTTCT.....\* 159   | 159; |
| .......NGAGGTAGTAGGTTGCATAGTT........................................................... 132   | 132; |
| .......AGAGGTAGTAGGTTGCA................................................................ 105   | 105; |
| .......AGAGGTAGTAGGTTGC................................................................. 63   | 63; |
| .......AGAGGTANTAGGTTGCATAGT............................................................ 43   | 43; |
| .......AGNGGTAGTAGGTTGCATAGTT........................................................... 42   | 42; |
| ........GAGGTAGTAGGTTGCATAGT............................................................ 38   | 38; |
| .......AGAGGTAGNAGGTTGCATAGTT........................................................... 33   | 33; |
| ..............................................................TATACGACCTGCTGCCTTTCT..... 32   | 32; |
| .......AGAGGTAGTNGGTTGCATAGT............................................................ 31   | 31; |
| .......AGAGGTAGTAGGTTGCNTAGTT........................................................... 30   | 30; |
| .......AGAGNTAGTAGGTTGCATAGTT........................................................... 29   | 29; |
| .......ANAGGTAGTAGGTTGCATAGTT........................................................... 28   | 28; |
| .......AGAGGTAGTAGGTTGCATAGNT........................................................... 28   | 28; |
| .......AGAGGTAGTAGGTTGCATAGTTTT......................................................... 28   | 28; |
| .......AGAGGTAGTAGGTTGCATANTT........................................................... 27   | 27; |
| .......AGANGTAGTAGGTTGCATAGTT........................................................... 26   | 26; |
| .......AGAGGNAGTAGGTTGCATAGTT........................................................... 26   | 26; |
| .......AGAGGTAGTAGGNTGCATAGTT........................................................... 25   | 25; |
| .......AGAGGTAGTAGGTTNCATAGT............................................................ 24   | 24; |
| .........AGGTAGTAGGTTGCATAGTT........................................................... 24   | 24; |
| .......NGAGGTAGTAGGTTGCATAGT............................................................ 24   | 24; |
| .......AGAGGTAGTAGGTTGCATAGTN........................................................... 22   | 22; |
| .......AGAGGTAGTANGTTGCATAGTT........................................................... 22   | 22; |
| .......AGAGGTNGTAGGTTGCATAGTT........................................................... 22   | 22; |
| ........GAGGTAGTAGGTTGCATAGTTT.......................................................... 21   | 21; |
| .......AGAGGTAGTAGGTTGCATNGTT........................................................... 20   | 20; |
| .......AGAGGTAGTAGGTTGNATAGTT........................................................... 19   | 19; |
| .......AGAGGTAGTAGGTTGCANAGTT........................................................... 19   | 19; |
| .......AGAGGTAGTAGNTTGCATAGTT........................................................... 19   | 19; |
| ......AAGAGGTAGTAGGTTGCATAGT............................................................ 15   | 15; |
| .......AGAGGTAGTAGGTNGCATAGTT........................................................... 15   | 15; |
| .............................................................CTATACGACCTGCTGCCTTTC...... 15   | 15; |
| .......AGAGGTANTAGGTTGCATAGTTT.......................................................... 14   | 14; |
| ......AAGAGGTAGTAGGTTGCATAGTT........................................................... 10   | 10; |
| .......AGAGGTAGTAGGTTG.................................................................. 10   | 10; |
| .......AGAGGTAGTAGGTTNCATAGTTT.......................................................... 10   | 10; |
| .......NGAGGTAGTAGGTTGCATAGTTT.......................................................... 10   | 10; |
| .......AGAGGTAGTNGGTTGCATAGTTT.......................................................... 9   | 9; |
| .......AGAGGTAGTAGGTTNCATAG............................................................. 7   | 7; |
| .......AGAGGTAGNAGGTTGCATAGTTT.......................................................... 6   | 6; |
| ........GAGGTAGTAGGTTGCATAG............................................................. 6   | 6; |
| .......AGAGGTAGTAGGTTGCNTAGT............................................................ 6   | 6; |
| .......AGAGGTANTAGGTTGCAT............................................................... 5   | 5; |
| ......AAGAGGTAGTAGGTTGCATAG............................................................. 5   | 5; |
| .......AGNGGTAGTAGGTTGCATAGT............................................................ 5   | 5; |
| ........GAGGTAGTAGGTTGCAT............................................................... 5   | 5; |
| ..........GGTAGTAGGTTGCATAGTT........................................................... 5   | 5; |
| .......AGAGGTAGTAGGTTGCATNGT............................................................ 5   | 5; |
| .......AGAGNTAGTAGGTTGCATAGT............................................................ 5   | 5; |
| --------------------------------------------------------------------------------------- 98375 |
| ..............................................................TATACGACCTGCTGCCTTTCa..... 6   | 6; |
| .............................................................CTATACGACCTGCTGCCTTTCa..... 5   | 5; |
| --------------------------------------------------------------------------------------- 11 |
| --------------------------------------------------------------------------------------- 98386 |
| hsa-let-7d-5p(hsa-let-7d) CCTAGGAAGAGGTAGTAGGTTGCATAGTTTTAGGGCAGGGATTTTGCCCACAAGGAGGTAACTATACGACCTGCTGCCTTTCTTAGG (((((((.((((((((((((((.((((((...((((((.....)))))).(.....)..)))))).))))))))))))))))))))) (-42.60) \*\*\*\*\*\*\*AGAGGTAGTAGGTTGCATAGTT\*\*\*\*\*\*\*\*\*\*\*\*\*\*\*\*\*\*\*\*\*\*\*\*\*\*\*\*\*\*\*\*\*\*\*\*\*\*\*\*\*\*\*\*\*\*\*\*\*\*\*\*\*\*\*\*\*\* T  M |
| .......AGAGGTAGTAGGTTGCATAGTT...........................................................\* 79335   | 79335; |
| .......AGAGGTAGTAGGTTGCATAGT............................................................ 9277   | 9277; |
| .......AGAGGTAGTAGGTTGCATAGTTT.......................................................... 4015   | 4015; |
| .......AGAGGTAGTAGGTTGCATAG............................................................. 1804   | 1804; |
| .......AGAGGTAGTAGGTTGCAT............................................................... 742   | 742; |
| .......AGAGGTAGTAGGTTGCATA.............................................................. 730   | 730; |
| ........GAGGTAGTAGGTTGCATAGTT........................................................... 396   | 396; |
| .......AGAGGTANTAGGTTGCATAGTT........................................................... 295   | 295; |
| .......AGAGGTAGTNGGTTGCATAGTT........................................................... 230   | 230; |
| .......AGAGGTAGTAGGTTNCATAGTT........................................................... 222   | 222; |
| .............................................................CTATACGACCTGCTGCCTTTCT..... 159   | 159; |
| .......NGAGGTAGTAGGTTGCATAGTT........................................................... 132   | 132; |
| .......AGAGGTAGTAGGTTGCA................................................................ 105   | 105; |
| .......AGAGGTAGTAGGTTGC................................................................. 63   | 63; |
| .......AGAGGTANTAGGTTGCATAGT............................................................ 43   | 43; |
| .......AGNGGTAGTAGGTTGCATAGTT........................................................... 42   | 42; |
| ........GAGGTAGTAGGTTGCATAGT............................................................ 38   | 38; |
| .......AGAGGTAGNAGGTTGCATAGTT........................................................... 33   | 33; |
| ..............................................................TATACGACCTGCTGCCTTTCT..... 32   | 32; |
| .......AGAGGTAGTNGGTTGCATAGT............................................................ 31   | 31; |
| .......AGAGGTAGTAGGTTGCNTAGTT........................................................... 30   | 30; |
| .......AGAGNTAGTAGGTTGCATAGTT........................................................... 29   | 29; |
| .......ANAGGTAGTAGGTTGCATAGTT........................................................... 28   | 28; |
| .......AGAGGTAGTAGGTTGCATAGNT........................................................... 28   | 28; |
| .......AGAGGTAGTAGGTTGCATAGTTTT......................................................... 28   | 28; |
| .......AGAGGTAGTAGGTTGCATANTT........................................................... 27   | 27; |
| .......AGANGTAGTAGGTTGCATAGTT........................................................... 26   | 26; |
| .......AGAGGNAGTAGGTTGCATAGTT........................................................... 26   | 26; |
| .......AGAGGTAGTAGGNTGCATAGTT........................................................... 25   | 25; |
| .......AGAGGTAGTAGGTTNCATAGT............................................................ 24   | 24; |
| .........AGGTAGTAGGTTGCATAGTT........................................................... 24   | 24; |
| .......NGAGGTAGTAGGTTGCATAGT............................................................ 24   | 24; |
| .......AGAGGTAGTAGGTTGCATAGTN........................................................... 22   | 22; |
| .......AGAGGTAGTANGTTGCATAGTT........................................................... 22   | 22; |
| .......AGAGGTNGTAGGTTGCATAGTT........................................................... 22   | 22; |
| ........GAGGTAGTAGGTTGCATAGTTT.......................................................... 21   | 21; |
| .......AGAGGTAGTAGGTTGCATNGTT........................................................... 20   | 20; |
| .......AGAGGTAGTAGGTTGNATAGTT........................................................... 19   | 19; |
| .......AGAGGTAGTAGGTTGCANAGTT........................................................... 19   | 19; |
| .......AGAGGTAGTAGNTTGCATAGTT........................................................... 19   | 19; |
| ......AAGAGGTAGTAGGTTGCATAGT............................................................ 15   | 15; |
| .......AGAGGTAGTAGGTNGCATAGTT........................................................... 15   | 15; |
| .............................................................CTATACGACCTGCTGCCTTTC...... 15   | 15; |
| .......AGAGGTANTAGGTTGCATAGTTT.......................................................... 14   | 14; |
| ......AAGAGGTAGTAGGTTGCATAGTT........................................................... 10   | 10; |
| .......AGAGGTAGTAGGTTG.................................................................. 10   | 10; |
| .......AGAGGTAGTAGGTTNCATAGTTT.......................................................... 10   | 10; |
| .......NGAGGTAGTAGGTTGCATAGTTT.......................................................... 10   | 10; |
| .......AGAGGTAGTNGGTTGCATAGTTT.......................................................... 9   | 9; |
| .......AGAGGTAGTAGGTTNCATAG............................................................. 7   | 7; |
| .......AGAGGTAGNAGGTTGCATAGTTT.......................................................... 6   | 6; |
| ........GAGGTAGTAGGTTGCATAG............................................................. 6   | 6; |
| .......AGAGGTAGTAGGTTGCNTAGT............................................................ 6   | 6; |
| .......AGAGGTANTAGGTTGCAT............................................................... 5   | 5; |
| ......AAGAGGTAGTAGGTTGCATAG............................................................. 5   | 5; |
| .......AGNGGTAGTAGGTTGCATAGT............................................................ 5   | 5; |
| ........GAGGTAGTAGGTTGCAT............................................................... 5   | 5; |
| ..........GGTAGTAGGTTGCATAGTT........................................................... 5   | 5; |
| .......AGAGGTAGTAGGTTGCATNGT............................................................ 5   | 5; |
| .......AGAGNTAGTAGGTTGCATAGT............................................................ 5   | 5; |
| --------------------------------------------------------------------------------------- 98375 |
| .......AGAGGTAGTAGGTTGCATAGTTa.......................................................... 1363   | 1363; |
| .......tGAGGTAGTAGGTTGCATAGTT........................................................... 387   | 387; |
| .......AGAGGTAGTAGGTTGCATAGTa........................................................... 245   | 245; |
| .......AGAGGTAGTAGGgTGCATAGTT........................................................... 194   | 194; |
| .......AGAGGTAGTAGGTTGCcTAGTT........................................................... 141   | 141; |
| .......AGAGGTgGTAGGTTGCATAGTT........................................................... 85   | 85; |
| .......AGAGGTAGTAGGTTGCgTAGTT........................................................... 83   | 83; |
| .......AGAGGTAGTAGGTTGCATAGgT........................................................... 82   | 82; |
| .......AGAGGTAGTAGGcTGCATAGTT........................................................... 69   | 69; |
| .......AGAGGcAGTAGGTTGCATAGTT........................................................... 68   | 68; |
| .......AGAGGTAGTAGGaTGCATAGTT........................................................... 61   | 61; |
| .......AGAGGTAGTAGGTcGCATAGTT........................................................... 61   | 61; |
| .......AGAGGTAtTAGGTTGCATAGTT........................................................... 60   | 60; |
| .......AGgGGTAGTAGGTTGCATAGTT........................................................... 55   | 55; |
| .......AGAGGTAGaAGGTTGCATAGTT........................................................... 55   | 55; |
| .......tGAGGTAGTAGGTTGCATAGT............................................................ 53   | 53; |
| .......AGAGGTAGTAGGTTGCtTAGTT........................................................... 49   | 49; |
| .......AGAGGTAGTAGGTTGCATAGaT........................................................... 48   | 48; |
| .......AGAGGTAGgAGGTTGCATAGTT........................................................... 46   | 46; |
| .......AGAGGaAGTAGGTTGCATAGTT........................................................... 45   | 45; |
| .......gGAGGTAGTAGGTTGCATAGTT........................................................... 45   | 45; |
| ......cAGAGGTAGTAGGTTGCATAGTT........................................................... 45   | 45; |
| .......AGAGGTAGTAGGTTGCATAGcT........................................................... 44   | 44; |
| .......AGAGGTAGcAGGTTGCATAGTT........................................................... 39   | 39; |
| .......AGAGGTAGTgGGTTGCATAGTT........................................................... 39   | 39; |
| .......AGAGGTAGTAGGTTGgATAGTT........................................................... 37   | 37; |
| .......AGAGGTAGTAGGTTGaATAGTT........................................................... 37   | 37; |
| .......AGAGGTAGTAGGTTaCATAGTT........................................................... 34   | 34; |
| .......AGAGGTAGTAGGgTGCATAGT............................................................ 34   | 34; |
| .......AtAGGTAGTAGGTTGCATAGTT........................................................... 31   | 31; |
| .......AGAGGTAGTAGGTTGCATAGTc........................................................... 28   | 28; |
| .......AGAGGTAGTAGGTTGCAaAGTT........................................................... 28   | 28; |
| .......AGAGGTAGTAGGTTGCATgGTT........................................................... 28   | 28; |
| .......AGAGGTAGTAGGTTGCATAGTTg.......................................................... 27   | 27; |
| ......AA-AGGTAGTAGGTTGCATAGTT........................................................... 25   | 25; |
| .......AGAGGTAGTtGGTTGCATAGTT........................................................... 25   | 25; |
| .......AGAGGTAGTAGGTTGCATcGTT........................................................... 25   | 25; |
| .......AGAGGTAaTAGGTTGCATAGTT........................................................... 24   | 24; |
| .......AGAGGTAGTAGGTTGCATAGa............................................................ 24   | 24; |
| .......AGAGGTAGTAGGTTtCATAGTT........................................................... 24   | 24; |
| .......AGAGaTAGTAGGTTGCATAGTT........................................................... 24   | 24; |
| .......AGAGGTAGTAG-TTGCATAGTT........................................................... 23   | 23; |
| .......AGAGGTAGTAGGTTGCATAGTaa.......................................................... 23   | 23; |
| .......AGAGGTAcTAGGTTGCATAGTT........................................................... 22   | 22; |
| .......AGAGGTAGTAGGTTGCAcAGTT........................................................... 22   | 22; |
| .......AGAG-TAGTAGGTTGCATAGTT........................................................... 21   | 21; |
| .......AGAGGTAGTAGGTTGCATAGTTc.......................................................... 20   | 20; |
| .......AGAGGTAGTAGGTTGCATAGTaT.......................................................... 20   | 20; |
| .......AGAGGTAGTAGGTTGCcTAGT............................................................ 20   | 20; |
| .......AGAaGTAGTAGGTTGCATAGTT........................................................... 20   | 20; |
| .......AGAGGTAGTAGGTTGCATAtTT........................................................... 20   | 20; |
| .......AGcGGTAGTAGGTTGCATAGTT........................................................... 18   | 18; |
| .......AGAGGTAGTAaGTTGCATAGTT........................................................... 18   | 18; |
| .......cGAGGTAGTAGGTTGCATAGTT........................................................... 17   | 17; |
| .......AGAGGgAGTAGGTTGCATAGTT........................................................... 17   | 17; |
| .......AGAGGTAGTAGGTTcCATAGTT........................................................... 17   | 17; |
| .......AGAGGTtGTAGGTTGCATAGTT........................................................... 16   | 16; |
| .......AGAGGTAGTAGtTTGCATAGTT........................................................... 16   | 16; |
| .......AGAGGTAGTAGaTTGCATAGTT........................................................... 16   | 16; |
| .......AGtGGTAGTAGGTTGCATAGTT........................................................... 16   | 16; |
| .......AGAGGT--TAGGTTGCATAGTT........................................................... 15   | 15; |
| .......AGAGGTAGTAtGTTGCATAGTT........................................................... 15   | 15; |
| .......AcAGGTAGTAGGTTGCATAGTT........................................................... 14   | 14; |
| .......AGAGGTAGTAGGTTGCcTAGTTT.......................................................... 14   | 14; |
| .......tGAGGTAGTAGGTTGCATAGTTT.......................................................... 13   | 13; |
| .......AGAGGTAGTcGGTTGCATAGTT........................................................... 13   | 13; |
| .......AGAtGTAGTAGGTTGCATAGTT........................................................... 11   | 11; |
| .......AGAGGTAGTAGGTaGCATAGTT........................................................... 11   | 11; |
| .......AGAGGTAGgAGGTTGCATAGT............................................................ 11   | 11; |
| .......AGAGGTAGTAGcTTGCATAGTT........................................................... 11   | 11; |
| .......AGAGGTcGTAGGTTGCATAGTT........................................................... 10   | 10; |
| .......AGAGGTAGTAGGTTGCATtGTT........................................................... 10   | 10; |
| .......AGAGGgAGTAGGgTGCATAGTT........................................................... 10   | 10; |
| .......AGAGGTAGTAGGTTGCATAcTT........................................................... 10   | 10; |
| .......AGAGGTAtTAGGTTGCATAGT............................................................ 9   | 9; |
| .......AGAGGTAGTAGGTTGCATAGgTT.......................................................... 9   | 9; |
| .......AGAGGTAGcAGGTTGCATAGTTT.......................................................... 9   | 9; |
| .......AGAGGTAGTAGGT-GCATAGTT........................................................... 9   | 9; |
| .......AGgGGTAGTAGGTTGCATAGT............................................................ 8   | 8; |
| .......AGAGGTAGTAGGTTGCgTAGT............................................................ 8   | 8; |
| .......tGAGGTAGTAGGTTGCATAGTTa.......................................................... 8   | 8; |
| .......gGAGGTAGTAGGTTGCATAGT............................................................ 7   | 7; |
| .......AGAGGTANTAGGTTGCATAGTTa.......................................................... 7   | 7; |
| .......AGAGGTAGTAGGTTGaATAGT............................................................ 7   | 7; |
| .......AGAGtTAGTAGGTTGCATAGTT........................................................... 7   | 7; |
| .......AGAGGTAGTAGGTTGCAaAGT............................................................ 7   | 7; |
| .......AGAGGTAGTAGGTTGCATAGc............................................................ 7   | 7; |
| .......AGAGGTAGTAGGTTNCATAGTTa.......................................................... 6   | 6; |
| ......tAGAGGTAGTAGGTTGCATAGT............................................................ 6   | 6; |
| .......AGAGGTAGcAGGTTGCATAGT............................................................ 6   | 6; |
| .......AGAGGTgGTAGGTTGCATAGT............................................................ 6   | 6; |
| .......AGAGGTAGTAGGTT-CATAGTT........................................................... 6   | 6; |
| ........GAGGTAGTAGGTTGCATAGTTa.......................................................... 6   | 6; |
| .......AGAGGTAGTAGGcTGCATAGT............................................................ 6   | 6; |
| .......AGAGGTAGTAGGTgGCATAGTT........................................................... 6   | 6; |
| .......AGAGGTAGTAGGTTGCATAaTT........................................................... 6   | 6; |
| .......AGAGGTAGTAGGaTGCATAGT............................................................ 6   | 6; |
| .......AGAGGTAGgAGGTTGCATAGgT........................................................... 6   | 6; |
| .......AGAGGTAG-AGGTTGCATAGTT........................................................... 6   | 6; |
| .......AGAGGcAGTAGGTTGCATAGT............................................................ 6   | 6; |
| .......AGAGGTAGTAGGTTGCtTAGT............................................................ 6   | 6; |
| .......AGAGGTAGTAGGggGCATAGTT........................................................... 5   | 5; |
| .......AGAGGTAGTNGGTTGCATAGTTa.......................................................... 5   | 5; |
| .......AGAGGaAGTAGGTTGCATAGT............................................................ 5   | 5; |
| .......AGAGcTAGTAGGTTGCATAGTT........................................................... 5   | 5; |
| .......AGAGGTAGTAGGTTGCATAGg............................................................ 5   | 5; |
| .......AGcGGTAGTAGGTTGCATAGT............................................................ 5   | 5; |
| .......AGAGGTAGgAGGgTGCATAGTT........................................................... 5   | 5; |
| .......AGAGGTAGTgGGTTGCATAGT............................................................ 5   | 5; |
| --------------------------------------------------------------------------------------- 4673 |
| --------------------------------------------------------------------------------------- 103048 |
| hsa-let-7e-3p(hsa-let-7e) CCCGGGCTGAGGTAGGAGGTTGTATAGTTGAGGAGGACACCCAAGGAGATCACTATACGGCCTCCTAGCTTTCCCCAGG ((.(((..(((.((((((((((((((((.((...((....)).......)))))))))))))))))).)))..))).)) (-37.80) \*\*\*\*\*\*\*\*\*\*\*\*\*\*\*\*\*\*\*\*\*\*\*\*\*\*\*\*\*\*\*\*\*\*\*\*\*\*\*\*\*\*\*\*\*\*\*\*\*\*\*\*CTATACGGCCTCCTAGCTTTCC\*\*\*\*\* T  M |
| .......TGAGGTAGGAGGTTGTATAGTT................................................... 21788   | 21788; |
| .......TGAGGTAGGAGGTTGTATAGT.................................................... 6905   | 6905; |
| .......TGAGGTAGGAGGTTGTATAG..................................................... 958   | 958; |
| .......TGAGGTAGGAGGTTGTATAGTTG.................................................. 264   | 264; |
| .......TGAGGTAGGAGGTTGTAT....................................................... 218   | 218; |
| .......TGAGGTAGGAGGTTGTATA...................................................... 159   | 159; |
| .......TGAGGTAGNAGGTTGTATAGTT................................................... 139   | 139; |
| .......TGAGGTANGAGGTTGTATAGTT................................................... 89   | 89; |
| ........GAGGTAGGAGGTTGTATAGTT................................................... 58   | 58; |
| .......TGAGGTAGGNGGTTGTATAGTT................................................... 57   | 57; |
| .......TGAGGTAGGAGGTTNTATAGTT................................................... 54   | 54; |
| .......TGAGGTAGGAGGTTGTA........................................................ 44   | 44; |
| .......TGAGGTAGNAGGTTGTATAGT.................................................... 38   | 38; |
| .......TGAGGTAGGAGG............................................................. 32   | 32; |
| .......NGAGGTAGGAGGTTGTATAGTT................................................... 26   | 26; |
| .......TGAGGTANGAGGTTGTATAGT.................................................... 26   | 26; |
| .......TGAGGTAGGNGGTTGTATAGT.................................................... 25   | 25; |
| ........GAGGTAGGAGGTTGTATAGT.................................................... 18   | 18; |
| .........AGGTAGGAGGTTGTATAGTT................................................... 16   | 16; |
| .......TGAGGTAGGAGGTTNTATAGT.................................................... 13   | 13; |
| .......NGAGGTAGGAGGTTGTATAGT.................................................... 12   | 12; |
| .......TGAGGTAGGAGGNTGTATAGTT................................................... 11   | 11; |
| .......TGAGGTAGGAGGTNGTATAGTT................................................... 10   | 10; |
| .......TGAGNTAGGAGGTTGTATAGTT................................................... 10   | 10; |
| .......TGAGGTAGGAGNTTGTATAGTT................................................... 10   | 10; |
| .......TGAGGTAGGAGGTTGNATAGTT................................................... 9   | 9; |
| .......TGNGGTAGGAGGTTGTATAGTT................................................... 8   | 8; |
| .......TGAGGNAGGAGGTTGTATAGTT................................................... 8   | 8; |
| .......TGAGGTAGGAGGTTGTANAGTT................................................... 8   | 8; |
| .......TGAGGTAGGAGGTTGT......................................................... 7   | 7; |
| .......TGAGGTAGGAGGTTGTATNGTT................................................... 7   | 7; |
| .......TGAGGTAGGAGGTTGTNTAGTT................................................... 7   | 7; |
| .......TGANGTAGGAGGTTGTATAGTT................................................... 7   | 7; |
| .......TGAGGTNGGAGGTTGTATAGTT................................................... 7   | 7; |
| .......TNAGGTAGGAGGTTGTATAGTT................................................... 6   | 6; |
| .......TGAGGTAGGAGGTTGTATAGNT................................................... 6   | 6; |
| .......TGAGGTAGGAGGTTGTATAGTTGA................................................. 6   | 6; |
| .......TGAGGTAGGAGGTTGTATAGTN................................................... 5   | 5; |
| .......TGANGTAGGAGGTTGTATAGT.................................................... 5   | 5; |
| .......TGAGGTAGGAGGTTNTATAG..................................................... 5   | 5; |
| .......TGAGGTAGGAGGTTGTATANTT................................................... 5   | 5; |
| ......CTGAGGTAGGAGGTTGTATAGTT................................................... 5   | 5; |
| .......TNAGGTAGGAGGTTGTATAGT.................................................... 5   | 5; |
| .......TGAGGTAGGAGGTTGTNTAGT.................................................... 5   | 5; |
| ------------------------------------------------------------------------------- 31101 |
| ------------------------------------------------------------------------------- 31101 |
| hsa-let-7e-5p(hsa-let-7e) CCCGGGCTGAGGTAGGAGGTTGTATAGTTGAGGAGGACACCCAAGGAGATCACTATACGGCCTCCTAGCTTTCCCCAGG ((.(((..(((.((((((((((((((((.((...((....)).......)))))))))))))))))).)))..))).)) (-37.80) \*\*\*\*\*\*\*TGAGGTAGGAGGTTGTATAGTT\*\*\*\*\*\*\*\*\*\*\*\*\*\*\*\*\*\*\*\*\*\*\*\*\*\*\*\*\*\*\*\*\*\*\*\*\*\*\*\*\*\*\*\*\*\*\*\*\*\* T  M |
| .......TGAGGTAGGAGGTTGTATAGTT...................................................\* 21788   | 21788; |
| .......TGAGGTAGGAGGTTGTATAGT.................................................... 6905   | 6905; |
| .......TGAGGTAGGAGGTTGTATAG..................................................... 958   | 958; |
| .......TGAGGTAGGAGGTTGTATAGTTG.................................................. 264   | 264; |
| .......TGAGGTAGGAGGTTGTAT....................................................... 218   | 218; |
| .......TGAGGTAGGAGGTTGTATA...................................................... 159   | 159; |
| .......TGAGGTAGNAGGTTGTATAGTT................................................... 139   | 139; |
| .......TGAGGTANGAGGTTGTATAGTT................................................... 89   | 89; |
| ........GAGGTAGGAGGTTGTATAGTT................................................... 58   | 58; |
| .......TGAGGTAGGNGGTTGTATAGTT................................................... 57   | 57; |
| .......TGAGGTAGGAGGTTNTATAGTT................................................... 54   | 54; |
| .......TGAGGTAGGAGGTTGTA........................................................ 44   | 44; |
| .......TGAGGTAGNAGGTTGTATAGT.................................................... 38   | 38; |
| .......TGAGGTAGGAGG............................................................. 32   | 32; |
| .......NGAGGTAGGAGGTTGTATAGTT................................................... 26   | 26; |
| .......TGAGGTANGAGGTTGTATAGT.................................................... 26   | 26; |
| .......TGAGGTAGGNGGTTGTATAGT.................................................... 25   | 25; |
| ........GAGGTAGGAGGTTGTATAGT.................................................... 18   | 18; |
| .........AGGTAGGAGGTTGTATAGTT................................................... 16   | 16; |
| .......TGAGGTAGGAGGTTNTATAGT.................................................... 13   | 13; |
| .......NGAGGTAGGAGGTTGTATAGT.................................................... 12   | 12; |
| .......TGAGGTAGGAGGNTGTATAGTT................................................... 11   | 11; |
| .......TGAGGTAGGAGGTNGTATAGTT................................................... 10   | 10; |
| .......TGAGNTAGGAGGTTGTATAGTT................................................... 10   | 10; |
| .......TGAGGTAGGAGNTTGTATAGTT................................................... 10   | 10; |
| .......TGAGGTAGGAGGTTGNATAGTT................................................... 9   | 9; |
| .......TGNGGTAGGAGGTTGTATAGTT................................................... 8   | 8; |
| .......TGAGGNAGGAGGTTGTATAGTT................................................... 8   | 8; |
| .......TGAGGTAGGAGGTTGTANAGTT................................................... 8   | 8; |
| .......TGAGGTAGGAGGTTGT......................................................... 7   | 7; |
| .......TGAGGTAGGAGGTTGTATNGTT................................................... 7   | 7; |
| .......TGAGGTAGGAGGTTGTNTAGTT................................................... 7   | 7; |
| .......TGANGTAGGAGGTTGTATAGTT................................................... 7   | 7; |
| .......TGAGGTNGGAGGTTGTATAGTT................................................... 7   | 7; |
| .......TNAGGTAGGAGGTTGTATAGTT................................................... 6   | 6; |
| .......TGAGGTAGGAGGTTGTATAGNT................................................... 6   | 6; |
| .......TGAGGTAGGAGGTTGTATAGTTGA................................................. 6   | 6; |
| .......TGAGGTAGGAGGTTGTATAGTN................................................... 5   | 5; |
| .......TGANGTAGGAGGTTGTATAGT.................................................... 5   | 5; |
| .......TGAGGTAGGAGGTTNTATAG..................................................... 5   | 5; |
| .......TGAGGTAGGAGGTTGTATANTT................................................... 5   | 5; |
| ......CTGAGGTAGGAGGTTGTATAGTT................................................... 5   | 5; |
| .......TNAGGTAGGAGGTTGTATAGT.................................................... 5   | 5; |
| .......TGAGGTAGGAGGTTGTNTAGT.................................................... 5   | 5; |
| ------------------------------------------------------------------------------- 31101 |
| .......TGAGGTAGGAGGTTGTATAGTTa.................................................. 867   | 867; |
| .......TGAGGTAGGAGGTTGTATAGTTt.................................................. 475   | 475; |
| .......TGAGGTAGGAGGTTGTATAGTa................................................... 203   | 203; |
| .......TGAGGTAGGAGaTTGTATAGTT................................................... 129   | 129; |
| .......TGAGGTAGGAGGTTGTATAGa.................................................... 100   | 100; |
| .......TGAGGTAGGAGGTTGTATAGgT................................................... 60   | 60; |
| .......TGAGGTAGGAGGTTGTATAGTaa.................................................. 59   | 59; |
| .......TGAGGTAGcAGGTTGTATAGTT................................................... 56   | 56; |
| .......TGAGGTAGGAGGgTGTATAGTT................................................... 45   | 45; |
| .......TGAGGTAGtAGGTTGTATAGTTG.................................................. 42   | 42; |
| .......TGAGGTAGaAGGTTGTATAGTT................................................... 40   | 40; |
| .......TGAGGTAGGAGGTTGTATAGaT................................................... 31   | 31; |
| .......TGAGGTAGGAGGTTGTATAGTaT.................................................. 30   | 30; |
| .......TGAGGTAGGAGGTTGgATAGTT................................................... 30   | 30; |
| .......TGAGGTAGGAGGTTGTATAGcT................................................... 26   | 26; |
| .......TGAGGTAGGAGGTTGTgTAGTT................................................... 25   | 25; |
| .......TGAGGTAGGAGGTTGTATAGg.................................................... 25   | 25; |
| .......TGAGGTAGGgGGTTGTATAGTT................................................... 23   | 23; |
| .......TGAGGTAGGAGGTTGTcTAGTT................................................... 22   | 22; |
| .......TGAGGTAGGAGaTTGTATAGT.................................................... 22   | 22; |
| .......TGAGGTAGGAGGTTGTATAGaa................................................... 20   | 20; |
| .......TGAGGTAtGAGGTTGTATAGTT................................................... 20   | 20; |
| .......cGAGGTAGGAGGTTGTATAGTT................................................... 20   | 20; |
| .......TGgGGTAGGAGGTTGTATAGTT................................................... 19   | 19; |
| .......TGAGGcAGGAGGTTGTATAGTT................................................... 19   | 19; |
| .......TGAGGTgGGAGGTTGTATAGTT................................................... 19   | 19; |
| .......TGAGGTAGGAGGTTGTATAGTTc.................................................. 16   | 16; |
| .......TaAGGTAGGAGGTTGTATAGTT................................................... 16   | 16; |
| .......TGAGGTAGGAGGgTGTATAGT.................................................... 15   | 15; |
| .......TGAGGTAGcAGGTTGTATAGT.................................................... 14   | 14; |
| .......TGAGGTcGGAGGTTGTATAGTT................................................... 13   | 13; |
| .......TGAGGTAGGAGcTTGTATAGTT................................................... 13   | 13; |
| .......TGAGGTAGGAGGTTGTA--GTT................................................... 12   | 12; |
| .......TGAGGTAGGAGGTTGTATAtTT................................................... 12   | 12; |
| .......TGAGGTAGGAGGTTGaATAGTT................................................... 12   | 12; |
| .......TGAGGTAGGAGGcTGTATAGTT................................................... 12   | 12; |
| .......TGAGGTAGGgGGTTGTATAGT.................................................... 11   | 11; |
| .......TGAGGTAGGAGGTTGTtTAGTT................................................... 11   | 11; |
| .......TGAGGTAGGAGGTTaTATAGTT................................................... 10   | 10; |
| .......TGAGGTAGGcGGTTGTATAGTT................................................... 10   | 10; |
| .......TGAGGaAGGAGGTTGTATAGTT................................................... 10   | 10; |
| .......TGAGGTAGGAGGTTGTcTAGT.................................................... 10   | 10; |
| .......cGAGGTAGGAGGTTGTATAGT.................................................... 9   | 9; |
| .......TGAGGTAGGAGaTTGTATAGgT................................................... 9   | 9; |
| .......TGAGGTAGGAGGTTGcATAGTT................................................... 9   | 9; |
| .......TGcGGTAGGAGGTTGTATAGTT................................................... 8   | 8; |
| .......TGAGGTAGGAGGTTGTATAaa.................................................... 8   | 8; |
| .......aGAGGTAGGAGGTTGTATAGT.................................................... 8   | 8; |
| .......TtAGGTAGGAGGTTGTATAGTT................................................... 8   | 8; |
| .......TGAGcTAGGAGGTTGTATAGTT................................................... 8   | 8; |
| .......TGAGGTAGaAGGTTGTATAGT.................................................... 8   | 8; |
| .......TGAGGTAGGAGGaTGTATAGTT................................................... 8   | 8; |
| .......TGAGGTAGGAaGTTGTATAGTT................................................... 7   | 7; |
| .......TGAGGTtGGAGGTTGTATAGTT................................................... 7   | 7; |
| .......TGAGGTAGGAGGTTGTATcGTT................................................... 7   | 7; |
| .......TGAGGTAGGAGGTT--ATAGTT................................................... 7   | 7; |
| .......TGtGGTAGGAGGTTGTATAGTT................................................... 7   | 7; |
| .......TGgGGTAGGAGGTTGTATAGT.................................................... 7   | 7; |
| .......TGAGGTAGGAGGTTtTATAGTT................................................... 7   | 7; |
| .......TGAGGTAtGAGGTTGTATAGT.................................................... 7   | 7; |
| .......TGAGGTAGGAGGcTGTATAGT.................................................... 7   | 7; |
| .......TGAGGgAGGAGGTTGTATAGTT................................................... 7   | 7; |
| .......TGAGGTAGGAGtTTGTATAGTT................................................... 7   | 7; |
| .......aGAGGTAGGAGGTTGTATAGTT................................................... 6   | 6; |
| .......TGAGGTAGGAGGTcGTATAGT.................................................... 6   | 6; |
| .......TGAGGTAcGAGGTTGTATAGTT................................................... 6   | 6; |
| .......TGAGGTAGGAGGTTGTATgGTT................................................... 6   | 6; |
| .......TGAGGTAGGAGGTTGTATAGgTT.................................................. 6   | 6; |
| .......TGAGGTAGGAGGTTGTAcAGTT................................................... 6   | 6; |
| .......TGAGGTAGGAGGTTGTgTAGT.................................................... 6   | 6; |
| .......TGAGGTAGGAGGTTGTATAGc.................................................... 6   | 6; |
| .......TGAGGTAGGtGGTTGTATAGTT................................................... 6   | 6; |
| .......TGAGGTAGGAGcTTGTATAGgT................................................... 6   | 6; |
| .......TGAGGTAGGAGGTTcTATAGTT................................................... 6   | 6; |
| .......TGAGGTAGGAGaTTGTATAGaT................................................... 5   | 5; |
| .......TGAGGTAGGAGGTTGTtTAGT.................................................... 5   | 5; |
| .......TGAGGcAGGAGGTTGTATAGT.................................................... 5   | 5; |
| .......TGAGGTAGGAGGTTGTAaAGTT................................................... 5   | 5; |
| .......TGAGaTAGGAGGTTGTATAGTT................................................... 5   | 5; |
| .......TGAGGTAGGAGGTTGgATAGaT................................................... 5   | 5; |
| .......TGAGGTAGGAGGTTGgATAGgT................................................... 5   | 5; |
| .......TGAG-TAGGAGGTTGTATAGTT................................................... 5   | 5; |
| .......TGAGGTAGGAGGaTGTATAGT.................................................... 5   | 5; |
| .......TGAGGTgGGAGGTTGTATAGgT................................................... 5   | 5; |
| .......TGAGGTcGGAGGTTGTATAGaT................................................... 5   | 5; |
| .......TGAGGTAGGAtGTTGTATAGTT................................................... 5   | 5; |
| .......TGAGGTAGGAGaTTGgATAGTT................................................... 3   | 3; |
| .......TGAGGTANGAGGTTGTATAGTTt.................................................. 3   | 3; |
| .......TGAGGTAGGAGaTTGTATAGTTa.................................................. 2   | 2; |
| .......TGAGGTAGaAGGTTGTATAGgT................................................... 2   | 2; |
| .......TGAGGTcGGAGaTTGTATAGTT................................................... 2   | 2; |
| .......TGAGGTAGGAGaTTGTATAGcT................................................... 2   | 2; |
| ........GAGGTAGGAGGTTGTATAGTTt.................................................. 2   | 2; |
| .......TGAGGTAGcAGGTTGTATAGTTa.................................................. 2   | 2; |
| .......TGAGGTAGGNGGTTGTATAGTTt.................................................. 1   | 1; |
| .......TGAGGTAGNAGGTTGTATAGTTa.................................................. 1   | 1; |
| .......TGAGGaAGGAGaTTGTATAGTT................................................... 1   | 1; |
| ------------------------------------------------------------------------------- 2931 |
| ------------------------------------------------------------------------------- 34032 |
| hsa-let-7f-1-3p(hsa-let-7f-1) TCAGAGTGAGGTAGTAGATTGTATAGTTGTGGGGTAGTGATTTTACCCTGTTCAGGAGATAACTATACAATCTATTGCCTTCCCTGA ((((.(..(((((((((((((((((((((((((((((.....))))))).........))))))))))))))))))))))..))))) (-43.30) \*\*\*\*\*\*\*\*\*\*\*\*\*\*\*\*\*\*\*\*\*\*\*\*\*\*\*\*\*\*\*\*\*\*\*\*\*\*\*\*\*\*\*\*\*\*\*\*\*\*\*\*\*\*\*\*\*\*\*\*\*\*CTATACAATCTATTGCCTTCCC\*\*\* T  M |
| ......TGAGGTAGTAGATTGTATAGTT............................................................ 483550   | 483550; |
| ......TGAGGTAGTAGATTGTATAGT............................................................. 77126   | 77126; |
| ......TGAGGTAGTAGATTGTATAG.............................................................. 9010   | 9010; |
| ......TGAGGTAGTAGATTGTAT................................................................ 3470   | 3470; |
| ......TGAGGTANTAGATTGTATAGTT............................................................ 3379   | 3379; |
| ......TGAGGTAGTAGATTGTATA............................................................... 3032   | 3032; |
| ......TGAGGTAGTNGATTGTATAGTT............................................................ 2959   | 2959; |
| ......TGAGGTAGTAGATTNTATAGTT............................................................ 2740   | 2740; |
| ......NGAGGTAGTAGATTGTATAGTT............................................................ 1577   | 1577; |
| .......GAGGTAGTAGATTGTATAGTT............................................................ 1383   | 1383; |
| ......TGAGGTAGTAGATTGTA................................................................. 768   | 768; |
| ......TGAGGTANTAGATTGTATAGT............................................................. 506   | 506; |
| ......TGAGGTAGTNGATTGTATAGT............................................................. 481   | 481; |
| ......TGAGGTAGTAGATTNTATAGT............................................................. 423   | 423; |
| ......TGAGGTAGTAGATTGTATAGTTG........................................................... 401   | 401; |
| ......TGAGGTAGTAGNTTGTATAGTT............................................................ 391   | 391; |
| ......TNAGGTAGTAGATTGTATAGTT............................................................ 368   | 368; |
| ......TGAGGTAGTAGATTGTNTAGTT............................................................ 368   | 368; |
| ......TGAGGTAGNAGATTGTATAGTT............................................................ 359   | 359; |
| ......TGAGGTAGTAGA...................................................................... 359   | 359; |
| ......TGAGNTAGTAGATTGTATAGTT............................................................ 316   | 316; |
| ......TGAGGTAGTAGATTGTATAGTN............................................................ 316   | 316; |
| ......TGAGGTNGTAGATTGTATAGTT............................................................ 313   | 313; |
| ......TGAGGTAGTAGATTGTANAGTT............................................................ 308   | 308; |
| ......TGAGGTAGTANATTGTATAGTT............................................................ 308   | 308; |
| ......TGAGGNAGTAGATTGTATAGTT............................................................ 308   | 308; |
| ......TGAGGTAGTAGATTGT.................................................................. 308   | 308; |
| ........AGGTAGTAGATTGTATAGTT............................................................ 307   | 307; |
| ......TGANGTAGTAGATTGTATAGTT............................................................ 306   | 306; |
| ......TGNGGTAGTAGATTGTATAGTT............................................................ 289   | 289; |
| ......TGAGGTAGTAGATTGTATAGNT............................................................ 282   | 282; |
| ......NGAGGTAGTAGATTGTATAGT............................................................. 281   | 281; |
| ......TGAGGTAGTAGATTGNATAGTT............................................................ 281   | 281; |
| ......TGAGGTAGTAGATNGTATAGTT............................................................ 281   | 281; |
| ......TGAGGTAGTAGANTGTATAGTT............................................................ 279   | 279; |
| ......TGAGGTAGTAGATTGTATANTT............................................................ 277   | 277; |
| ......TGAGGTAGTAGATTGTATNGTT............................................................ 277   | 277; |
| .......GAGGTAGTAGATTGTATAGT............................................................. 216   | 216; |
| ......TGAGGTAGTAGNTTGTATAGT............................................................. 82   | 82; |
| ......TGAGGTAGTAGATTGTNTAGT............................................................. 69   | 69; |
| ......TGAGGTANTAGATTGTATAG.............................................................. 68   | 68; |
| ......TGAGGTAGTAGATTG................................................................... 68   | 68; |
| ......TGAGGTAGNAGATTGTATAGT............................................................. 62   | 62; |
| ......TGAGGNAGTAGATTGTATAGT............................................................. 60   | 60; |
| ......TNAGGTAGTAGATTGTATAGT............................................................. 60   | 60; |
| ......TGAGGTAGTNGATTGTATAG.............................................................. 55   | 55; |
| ......TGAGGTAGTAGATNGTATAGT............................................................. 52   | 52; |
| ......TGAGGTNGTAGATTGTATAGT............................................................. 51   | 51; |
| ......TGAGGTAGTAGATTNTATAG.............................................................. 51   | 51; |
| ......TGAGGTAGTANATTGTATAGT............................................................. 50   | 50; |
| ......TGAGGTAGTAGATTGTATANT............................................................. 49   | 49; |
| ......TGANGTAGTAGATTGTATAGT............................................................. 48   | 48; |
| ......TGAGGTAGTAGATTGNATAGT............................................................. 47   | 47; |
| ......TGNGGTAGTAGATTGTATAGT............................................................. 47   | 47; |
| ......TGAGGTAGTAGANTGTATAGT............................................................. 47   | 47; |
| ......TGAGGTAGTAGATTGTATNGT............................................................. 45   | 45; |
| ......TGAGGTAGTAGATTGTANAGT............................................................. 45   | 45; |
| ......TGAGNTAGTAGATTGTATAGT............................................................. 44   | 44; |
| ......TGAGGTAGTAGATTGTATAGN............................................................. 40   | 40; |
| ......NGAGGTAGTAGATTGTATAG.............................................................. 39   | 39; |
| ........AGGTAGTAGATTGTATAGT............................................................. 34   | 34; |
| ......TGAGGTAGTAGATTNTATA............................................................... 25   | 25; |
| ......TGAGGTAGTNGATTGTAT................................................................ 21   | 21; |
| .......GAGGTAGTAGATTGTATAG.............................................................. 20   | 20; |
| ......TGAGGTAGTAGATTNTAT................................................................ 19   | 19; |
| ......TGAGGTANTAGATTGTAT................................................................ 19   | 19; |
| ......TGAGGTANTAGATTGTATA............................................................... 17   | 17; |
| ......TGAGGTAGTNGATTGTATA............................................................... 17   | 17; |
| ......NGAGGTAGTAGATTGTAT................................................................ 16   | 16; |
| ........AGGTAGTAGATTGTATAGTTGT.......................................................... 15   | 15; |
| ......TGAGGTAGTAGATT.................................................................... 14   | 14; |
| .......GAGGTAGNAGATTGTATAGTT............................................................ 12   | 12; |
| ......TGAGGTAGTAGATTGTNTAG.............................................................. 10   | 10; |
| ......TGAGGNAGTAGATTGTATAG.............................................................. 10   | 10; |
| .......GAGGTAGTANATTGTATAGTT............................................................ 10   | 10; |
| .......GAGGTAGTAGATTGNATAGTT............................................................ 10   | 10; |
| ......TGAGGTAGTAGATTGNATAG.............................................................. 9   | 9; |
| ......NGAGGTAGTAGATTGTATA............................................................... 9   | 9; |
| ......TGNGGTAGTAGATTGTATAG.............................................................. 8   | 8; |
| ......TGAGGTAGTAGATTGTATAGTTN........................................................... 8   | 8; |
| ......TGAGGTNGTAGATTGTATAG.............................................................. 8   | 8; |
| ......TGAGGTAGTAGAT..................................................................... 8   | 8; |
| .......NAGGTAGTAGATTGTATAGTT............................................................ 7   | 7; |
| ......TGAGGTAGTAGNTTGTATAG.............................................................. 7   | 7; |
| .......GAGGTAGTAGATTGTAT................................................................ 7   | 7; |
| ......TGAGGTAGTANATTGTATAG.............................................................. 6   | 6; |
| ......TGANGTAGTAGATTGTATAG.............................................................. 6   | 6; |
| ..........GTAGTAGATTGTATAGTT............................................................ 6   | 6; |
| ......TGAGGTAGTAGANTGTATAG.............................................................. 6   | 6; |
| ......TGAGGTANTAGATTGTA................................................................. 6   | 6; |
| ......TGAGGTAGTAGATNGTATAG.............................................................. 5   | 5; |
| ......TGAGNTAGTAGATTGTATAG.............................................................. 5   | 5; |
| .......GAGGTAGTAGATTGTATA............................................................... 5   | 5; |
| ......TGAGGTAGNAGATTGTATAG.............................................................. 5   | 5; |
| ......TGAGGTAGTAGATTGTATAGTTGT.......................................................... 5   | 5; |
| .........GGTAGTAGATTGTATAGTT............................................................ 5   | 5; |
| ......TNAGGTAGTAGATTGTATAG.............................................................. 5   | 5; |
| ......TGAGGTAGTAGATTGTATNG.............................................................. 5   | 5; |
| ......TGAGGTAGTAGATNGTATA............................................................... 5   | 5; |
| ............AGTAGATTGTATAGTT............................................................ 4   | 4; |
| ........AGGTAGTAGATTGTATAG.............................................................. 3   | 3; |
| ......TGAGGTAGTAG....................................................................... 1   | 1; |
| --------------------------------------------------------------------------------------- 599505 |
| --------------------------------------------------------------------------------------- 599505 |
| hsa-let-7f-2-3p(hsa-let-7f-2) TGTGGGATGAGGTAGTAGATTGTATAGTTTTAGGGTCATACCCCATCTTGGAGATAACTATACAGTCTACTGTCTTTCCCACG .((((((.(((..((((((((((((((((...(((.....))).(((.....)))))))))))))))))))..))))))))). (-40.70) \*\*\*\*\*\*\*\*\*\*\*\*\*\*\*\*\*\*\*\*\*\*\*\*\*\*\*\*\*\*\*\*\*\*\*\*\*\*\*\*\*\*\*\*\*\*\*\*\*\*\*\*\*\*\*\*\*CTATACAGTCTACTGTCTTTCC\*\*\*\* T  M |
| .......TGAGGTAGTAGATTGTATAGTT....................................................... 483550   | 483550; |
| .......TGAGGTAGTAGATTGTATAGT........................................................ 77126   | 77126; |
| .......TGAGGTAGTAGATTGTATAGTTT...................................................... 18085   | 18085; |
| .......TGAGGTAGTAGATTGTATAG......................................................... 9010   | 9010; |
| .......TGAGGTAGTAGATTGTAT........................................................... 3470   | 3470; |
| .......TGAGGTANTAGATTGTATAGTT....................................................... 3379   | 3379; |
| .......TGAGGTAGTAGATTGTATA.......................................................... 3032   | 3032; |
| .......TGAGGTAGTNGATTGTATAGTT....................................................... 2959   | 2959; |
| .......TGAGGTAGTAGATTNTATAGTT....................................................... 2740   | 2740; |
| .......NGAGGTAGTAGATTGTATAGTT....................................................... 1577   | 1577; |
| ........GAGGTAGTAGATTGTATAGTT....................................................... 1383   | 1383; |
| .......TGAGGTAGTAGATTGTA............................................................ 768   | 768; |
| .......TGAGGTANTAGATTGTATAGT........................................................ 506   | 506; |
| .......TGAGGTAGTNGATTGTATAGT........................................................ 481   | 481; |
| .......TGAGGTAGTAGATTNTATAGT........................................................ 423   | 423; |
| .......TGAGGTAGTAGNTTGTATAGTT....................................................... 391   | 391; |
| .......TNAGGTAGTAGATTGTATAGTT....................................................... 368   | 368; |
| .......TGAGGTAGTAGATTGTNTAGTT....................................................... 368   | 368; |
| .......TGAGGTAGNAGATTGTATAGTT....................................................... 359   | 359; |
| .......TGAGGTAGTAGA................................................................. 359   | 359; |
| .......TGAGNTAGTAGATTGTATAGTT....................................................... 316   | 316; |
| .......TGAGGTAGTAGATTGTATAGTN....................................................... 316   | 316; |
| .......TGAGGTNGTAGATTGTATAGTT....................................................... 313   | 313; |
| .......TGAGGTAGTAGATTGTANAGTT....................................................... 308   | 308; |
| .......TGAGGTAGTAGATTGT............................................................. 308   | 308; |
| .......TGAGGNAGTAGATTGTATAGTT....................................................... 308   | 308; |
| .......TGAGGTAGTANATTGTATAGTT....................................................... 308   | 308; |
| .........AGGTAGTAGATTGTATAGTT....................................................... 307   | 307; |
| .......TGANGTAGTAGATTGTATAGTT....................................................... 306   | 306; |
| .......TGNGGTAGTAGATTGTATAGTT....................................................... 289   | 289; |
| .......TGAGGTAGTAGATTGTATAGNT....................................................... 282   | 282; |
| .......TGAGGTAGTAGATTGNATAGTT....................................................... 281   | 281; |
| .......NGAGGTAGTAGATTGTATAGT........................................................ 281   | 281; |
| .......TGAGGTAGTAGATNGTATAGTT....................................................... 281   | 281; |
| .......TGAGGTAGTAGANTGTATAGTT....................................................... 279   | 279; |
| .......TGAGGTAGTAGATTGTATANTT....................................................... 277   | 277; |
| .......TGAGGTAGTAGATTGTATNGTT....................................................... 277   | 277; |
| ........GAGGTAGTAGATTGTATAGT........................................................ 216   | 216; |
| .......TGAGGTAGTAGNTTGTATAGT........................................................ 82   | 82; |
| ........GAGGTAGTAGATTGTATAGTTT...................................................... 79   | 79; |
| ......ATGAGGTAGTAGATTGTATAGTT....................................................... 79   | 79; |
| .......TGAGGTAGTAGATTGTNTAGT........................................................ 69   | 69; |
| .......TGAGGTAGTAGATTG.............................................................. 68   | 68; |
| .......TGAGGTANTAGATTGTATAG......................................................... 68   | 68; |
| .......TGAGGTAGNAGATTGTATAGT........................................................ 62   | 62; |
| .......TNAGGTAGTAGATTGTATAGT........................................................ 60   | 60; |
| .......TGAGGNAGTAGATTGTATAGT........................................................ 60   | 60; |
| .......TGAGGTANTAGATTGTATAGTTT...................................................... 58   | 58; |
| .......TGAGGTAGTNGATTGTATAGTTT...................................................... 55   | 55; |
| .......TGAGGTAGTNGATTGTATAG......................................................... 55   | 55; |
| ......ATGAGGTAGTAGATTGTATAGT........................................................ 54   | 54; |
| .......TGAGGTAGTAGATNGTATAGT........................................................ 52   | 52; |
| .......TGAGGTNGTAGATTGTATAGT........................................................ 51   | 51; |
| .......TGAGGTAGTAGATTNTATAG......................................................... 51   | 51; |
| .......TGAGGTAGTAGATTNTATAGTTT...................................................... 50   | 50; |
| .......TGAGGTAGTANATTGTATAGT........................................................ 50   | 50; |
| .......TGAGGTAGTAGATTGTATANT........................................................ 49   | 49; |
| .......TGANGTAGTAGATTGTATAGT........................................................ 48   | 48; |
| .......TGAGGTAGTAGATTGNATAGT........................................................ 47   | 47; |
| .......TGNGGTAGTAGATTGTATAGT........................................................ 47   | 47; |
| .......TGAGGTAGTAGANTGTATAGT........................................................ 47   | 47; |
| .......NGAGGTAGTAGATTGTATAGTTT...................................................... 45   | 45; |
| .......TGAGGTAGTAGATTGTANAGT........................................................ 45   | 45; |
| .......TGAGGTAGTAGATTGTATNGT........................................................ 45   | 45; |
| .......TGAGNTAGTAGATTGTATAGT........................................................ 44   | 44; |
| .......TGAGGTAGTAGATTGTATAGTTTT..................................................... 42   | 42; |
| .......TGAGGTAGTAGATTGTATAGN........................................................ 40   | 40; |
| .......NGAGGTAGTAGATTGTATAG......................................................... 39   | 39; |
| .........AGGTAGTAGATTGTATAGT........................................................ 34   | 34; |
| .......TGAGGTAGTAGATTNTATA.......................................................... 25   | 25; |
| .......TGAGGTAGTNGATTGTAT........................................................... 21   | 21; |
| ........GAGGTAGTAGATTGTATAG......................................................... 20   | 20; |
| .......TGAGGTANTAGATTGTAT........................................................... 19   | 19; |
| .......TGAGGTAGTAGATTNTAT........................................................... 19   | 19; |
| .......TGAGGTAGTNGATTGTATA.......................................................... 17   | 17; |
| .......TGAGGTANTAGATTGTATA.......................................................... 17   | 17; |
| .......NGAGGTAGTAGATTGTAT........................................................... 16   | 16; |
| .........AGGTAGTAGATTGTATAGTTT...................................................... 15   | 15; |
| .......TGAGGTAGTAGATT............................................................... 14   | 14; |
| ........GAGGTAGNAGATTGTATAGTT....................................................... 12   | 12; |
| .......TGAGGTAGTAGATTGTANAGTTT...................................................... 11   | 11; |
| .......TGAGGTAGTAGATTGTNTAG......................................................... 10   | 10; |
| .......TGAGGNAGTAGATTGTATAG......................................................... 10   | 10; |
| .......TGAGGTAGTAGNTTGTATAGTTT...................................................... 10   | 10; |
| ........GAGGTAGTANATTGTATAGTT....................................................... 10   | 10; |
| ........GAGGTAGTAGATTGNATAGTT....................................................... 10   | 10; |
| .......TGANGTAGTAGATTGTATAGTTT...................................................... 9   | 9; |
| .......TGAGGTAGTAGATTGNATAG......................................................... 9   | 9; |
| .......NGAGGTAGTAGATTGTATA.......................................................... 9   | 9; |
| .......TGAGGTAGTAGATTGNATAGTTT...................................................... 9   | 9; |
| .......TGAGGTNGTAGATTGTATAGTTT...................................................... 8   | 8; |
| .......TGAGGTNGTAGATTGTATAG......................................................... 8   | 8; |
| .......TGNGGTAGTAGATTGTATAG......................................................... 8   | 8; |
| .......TGAGGTAGTAGATTGTATANTTT...................................................... 8   | 8; |
| .......TGAGGTAGTAGAT................................................................ 8   | 8; |
| .......TGAGGTAGTAGATTGTATAGTTN...................................................... 8   | 8; |
| ......ATGAGGTAGTAGATTGTATAG......................................................... 8   | 8; |
| .......TGAGGTAGTAGNTTGTATAG......................................................... 7   | 7; |
| ........GAGGTAGTAGATTGTAT........................................................... 7   | 7; |
| ........NAGGTAGTAGATTGTATAGTT....................................................... 7   | 7; |
| .......TGAGGTAGNAGATTGTATAGTTT...................................................... 6   | 6; |
| .......TGAGGTAGTANATTGTATAG......................................................... 6   | 6; |
| .......TGAGGTANTAGATTGTA............................................................ 6   | 6; |
| .......TGAGGTAGTANATTGTATAGTTT...................................................... 6   | 6; |
| .......TGANGTAGTAGATTGTATAG......................................................... 6   | 6; |
| ...........GTAGTAGATTGTATAGTT....................................................... 6   | 6; |
| .......TGAGGTAGTAGATNGTATAGTTT...................................................... 6   | 6; |
| .......TGAGGTAGTAGANTGTATAG......................................................... 6   | 6; |
| .......TNAGGTAGTAGATTGTATAGTTT...................................................... 6   | 6; |
| .......TGAGGNAGTAGATTGTATAGTTT...................................................... 5   | 5; |
| ........GAGGTAGTAGATTGTATA.......................................................... 5   | 5; |
| .......TGAGGTAGTAGATTGTNTAGTTT...................................................... 5   | 5; |
| .......TGAGNTAGTAGATTGTATAG......................................................... 5   | 5; |
| .......TGNGGTAGTAGATTGTATAGTTT...................................................... 5   | 5; |
| ..........GGTAGTAGATTGTATAGTT....................................................... 5   | 5; |
| .......TGAGGTAGTAGATNGTATA.......................................................... 5   | 5; |
| .......TGAGGTAGTAGATTGTATNG......................................................... 5   | 5; |
| .......TGAGGTAGTAGATTGTATAGTNT...................................................... 5   | 5; |
| .......TNAGGTAGTAGATTGTATAG......................................................... 5   | 5; |
| .......TGAGGTAGNAGATTGTATAG......................................................... 5   | 5; |
| .......TGAGGTAGTAGATNGTATAG......................................................... 5   | 5; |
| .............AGTAGATTGTATAGTT....................................................... 4   | 4; |
| .........AGGTAGTAGATTGTATAG......................................................... 3   | 3; |
| .......TGAGGTAGTAG.................................................................. 1   | 1; |
| ----------------------------------------------------------------------------------- 617753 |
| .........................................................CTATACAGTCTACTGTCTTTCt..... 5   | 5; |
| ----------------------------------------------------------------------------------- 5 |
| ----------------------------------------------------------------------------------- 617758 |
| hsa-let-7f-5p(hsa-let-7f-1) TCAGAGTGAGGTAGTAGATTGTATAGTTGTGGGGTAGTGATTTTACCCTGTTCAGGAGATAACTATACAATCTATTGCCTTCCCTGA ((((.(..(((((((((((((((((((((((((((((.....))))))).........))))))))))))))))))))))..))))) (-43.30) \*\*\*\*\*\*TGAGGTAGTAGATTGTATAGTT\*\*\*\*\*\*\*\*\*\*\*\*\*\*\*\*\*\*\*\*\*\*\*\*\*\*\*\*\*\*\*\*\*\*\*\*\*\*\*\*\*\*\*\*\*\*\*\*\*\*\*\*\*\*\*\*\*\*\* T  M |
| ......TGAGGTAGTAGATTGTATAGTT............................................................\* 483550   | 483550; |
| ......TGAGGTAGTAGATTGTATAGT............................................................. 77126   | 77126; |
| ......TGAGGTAGTAGATTGTATAG.............................................................. 9010   | 9010; |
| ......TGAGGTAGTAGATTGTAT................................................................ 3470   | 3470; |
| ......TGAGGTANTAGATTGTATAGTT............................................................ 3379   | 3379; |
| ......TGAGGTAGTAGATTGTATA............................................................... 3032   | 3032; |
| ......TGAGGTAGTNGATTGTATAGTT............................................................ 2959   | 2959; |
| ......TGAGGTAGTAGATTNTATAGTT............................................................ 2740   | 2740; |
| ......NGAGGTAGTAGATTGTATAGTT............................................................ 1577   | 1577; |
| .......GAGGTAGTAGATTGTATAGTT............................................................ 1383   | 1383; |
| ......TGAGGTAGTAGATTGTA................................................................. 768   | 768; |
| ......TGAGGTANTAGATTGTATAGT............................................................. 506   | 506; |
| ......TGAGGTAGTNGATTGTATAGT............................................................. 481   | 481; |
| ......TGAGGTAGTAGATTNTATAGT............................................................. 423   | 423; |
| ......TGAGGTAGTAGATTGTATAGTTG........................................................... 401   | 401; |
| ......TGAGGTAGTAGNTTGTATAGTT............................................................ 391   | 391; |
| ......TNAGGTAGTAGATTGTATAGTT............................................................ 368   | 368; |
| ......TGAGGTAGTAGATTGTNTAGTT............................................................ 368   | 368; |
| ......TGAGGTAGNAGATTGTATAGTT............................................................ 359   | 359; |
| ......TGAGGTAGTAGA...................................................................... 359   | 359; |
| ......TGAGNTAGTAGATTGTATAGTT............................................................ 316   | 316; |
| ......TGAGGTAGTAGATTGTATAGTN............................................................ 316   | 316; |
| ......TGAGGTNGTAGATTGTATAGTT............................................................ 313   | 313; |
| ......TGAGGTAGTAGATTGTANAGTT............................................................ 308   | 308; |
| ......TGAGGTAGTANATTGTATAGTT............................................................ 308   | 308; |
| ......TGAGGNAGTAGATTGTATAGTT............................................................ 308   | 308; |
| ......TGAGGTAGTAGATTGT.................................................................. 308   | 308; |
| ........AGGTAGTAGATTGTATAGTT............................................................ 307   | 307; |
| ......TGANGTAGTAGATTGTATAGTT............................................................ 306   | 306; |
| ......TGNGGTAGTAGATTGTATAGTT............................................................ 289   | 289; |
| ......TGAGGTAGTAGATTGTATAGNT............................................................ 282   | 282; |
| ......NGAGGTAGTAGATTGTATAGT............................................................. 281   | 281; |
| ......TGAGGTAGTAGATTGNATAGTT............................................................ 281   | 281; |
| ......TGAGGTAGTAGATNGTATAGTT............................................................ 281   | 281; |
| ......TGAGGTAGTAGANTGTATAGTT............................................................ 279   | 279; |
| ......TGAGGTAGTAGATTGTATANTT............................................................ 277   | 277; |
| ......TGAGGTAGTAGATTGTATNGTT............................................................ 277   | 277; |
| .......GAGGTAGTAGATTGTATAGT............................................................. 216   | 216; |
| ......TGAGGTAGTAGNTTGTATAGT............................................................. 82   | 82; |
| ......TGAGGTAGTAGATTGTNTAGT............................................................. 69   | 69; |
| ......TGAGGTANTAGATTGTATAG.............................................................. 68   | 68; |
| ......TGAGGTAGTAGATTG................................................................... 68   | 68; |
| ......TGAGGTAGNAGATTGTATAGT............................................................. 62   | 62; |
| ......TGAGGNAGTAGATTGTATAGT............................................................. 60   | 60; |
| ......TNAGGTAGTAGATTGTATAGT............................................................. 60   | 60; |
| ......TGAGGTAGTNGATTGTATAG.............................................................. 55   | 55; |
| ......TGAGGTAGTAGATNGTATAGT............................................................. 52   | 52; |
| ......TGAGGTNGTAGATTGTATAGT............................................................. 51   | 51; |
| ......TGAGGTAGTAGATTNTATAG.............................................................. 51   | 51; |
| ......TGAGGTAGTANATTGTATAGT............................................................. 50   | 50; |
| ......TGAGGTAGTAGATTGTATANT............................................................. 49   | 49; |
| ......TGANGTAGTAGATTGTATAGT............................................................. 48   | 48; |
| ......TGAGGTAGTAGATTGNATAGT............................................................. 47   | 47; |
| ......TGNGGTAGTAGATTGTATAGT............................................................. 47   | 47; |
| ......TGAGGTAGTAGANTGTATAGT............................................................. 47   | 47; |
| ......TGAGGTAGTAGATTGTATNGT............................................................. 45   | 45; |
| ......TGAGGTAGTAGATTGTANAGT............................................................. 45   | 45; |
| ......TGAGNTAGTAGATTGTATAGT............................................................. 44   | 44; |
| ......TGAGGTAGTAGATTGTATAGN............................................................. 40   | 40; |
| ......NGAGGTAGTAGATTGTATAG.............................................................. 39   | 39; |
| ........AGGTAGTAGATTGTATAGT............................................................. 34   | 34; |
| ......TGAGGTAGTAGATTNTATA............................................................... 25   | 25; |
| ......TGAGGTAGTNGATTGTAT................................................................ 21   | 21; |
| .......GAGGTAGTAGATTGTATAG.............................................................. 20   | 20; |
| ......TGAGGTAGTAGATTNTAT................................................................ 19   | 19; |
| ......TGAGGTANTAGATTGTAT................................................................ 19   | 19; |
| ......TGAGGTANTAGATTGTATA............................................................... 17   | 17; |
| ......TGAGGTAGTNGATTGTATA............................................................... 17   | 17; |
| ......NGAGGTAGTAGATTGTAT................................................................ 16   | 16; |
| ........AGGTAGTAGATTGTATAGTTGT.......................................................... 15   | 15; |
| ......TGAGGTAGTAGATT.................................................................... 14   | 14; |
| .......GAGGTAGNAGATTGTATAGTT............................................................ 12   | 12; |
| ......TGAGGTAGTAGATTGTNTAG.............................................................. 10   | 10; |
| ......TGAGGNAGTAGATTGTATAG.............................................................. 10   | 10; |
| .......GAGGTAGTANATTGTATAGTT............................................................ 10   | 10; |
| .......GAGGTAGTAGATTGNATAGTT............................................................ 10   | 10; |
| ......TGAGGTAGTAGATTGNATAG.............................................................. 9   | 9; |
| ......NGAGGTAGTAGATTGTATA............................................................... 9   | 9; |
| ......TGNGGTAGTAGATTGTATAG.............................................................. 8   | 8; |
| ......TGAGGTAGTAGATTGTATAGTTN........................................................... 8   | 8; |
| ......TGAGGTNGTAGATTGTATAG.............................................................. 8   | 8; |
| ......TGAGGTAGTAGAT..................................................................... 8   | 8; |
| .......NAGGTAGTAGATTGTATAGTT............................................................ 7   | 7; |
| ......TGAGGTAGTAGNTTGTATAG.............................................................. 7   | 7; |
| .......GAGGTAGTAGATTGTAT................................................................ 7   | 7; |
| ......TGAGGTAGTANATTGTATAG.............................................................. 6   | 6; |
| ......TGANGTAGTAGATTGTATAG.............................................................. 6   | 6; |
| ..........GTAGTAGATTGTATAGTT............................................................ 6   | 6; |
| ......TGAGGTAGTAGANTGTATAG.............................................................. 6   | 6; |
| ......TGAGGTANTAGATTGTA................................................................. 6   | 6; |
| ......TGAGGTAGTAGATNGTATAG.............................................................. 5   | 5; |
| ......TGAGNTAGTAGATTGTATAG.............................................................. 5   | 5; |
| .......GAGGTAGTAGATTGTATA............................................................... 5   | 5; |
| ......TGAGGTAGNAGATTGTATAG.............................................................. 5   | 5; |
| ......TGAGGTAGTAGATTGTATAGTTGT.......................................................... 5   | 5; |
| .........GGTAGTAGATTGTATAGTT............................................................ 5   | 5; |
| ......TNAGGTAGTAGATTGTATAG.............................................................. 5   | 5; |
| ......TGAGGTAGTAGATTGTATNG.............................................................. 5   | 5; |
| ......TGAGGTAGTAGATNGTATA............................................................... 5   | 5; |
| ............AGTAGATTGTATAGTT............................................................ 4   | 4; |
| ........AGGTAGTAGATTGTATAG.............................................................. 3   | 3; |
| ......TGAGGTAGTAG....................................................................... 1   | 1; |
| --------------------------------------------------------------------------------------- 599505 |
| ......TGAGGTAGTAGATTGTATAGTTa........................................................... 9259   | 9259; |
| ......TGAGGTAGTAGATTGTATAGTa............................................................ 871   | 871; |
| ......TGAGGTAGTAGATTGTA--GTT............................................................ 643   | 643; |
| ......TGAGGTAGTAGATTGTcTAGTT............................................................ 607   | 607; |
| ......TGAGGTAGTAGATTGTgTAGTT............................................................ 503   | 503; |
| ......TGAGGcAGTAGATTGTATAGTT............................................................ 447   | 447; |
| ......TGAGGTgGTAGATTGTATAGTT............................................................ 428   | 428; |
| ......TaAGGTAGTAGATTGTATAGTT............................................................ 403   | 403; |
| ......TGgGGTAGTAGATTGTATAGTT............................................................ 392   | 392; |
| ......TGAGGTAGTgGATTGTATAGTT............................................................ 337   | 337; |
| ......TGAGGTAtTAGATTGTATAGTT............................................................ 324   | 324; |
| ......cGAGGTAGTAGATTGTATAGTT............................................................ 322   | 322; |
| ......TGAGGTAGcAGATTGTATAGTT............................................................ 319   | 319; |
| ......TGAGGTAGTAGATTGTATAGgT............................................................ 270   | 270; |
| ......TGAGGTAGTAGATTGTtTAGTT............................................................ 266   | 266; |
| .....cTGAGGTAGTAGATTGTATAGTT............................................................ 265   | 265; |
| ......TGAGGTAGTAGATTGTATAGcT............................................................ 264   | 264; |
| ......TGAGGTAGaAGATTGTATAGTT............................................................ 245   | 245; |
| ......TGAGGTAGTAGATcGTATAGTT............................................................ 241   | 241; |
| ......TGAGGTAGTAGATTGcATAGTT............................................................ 239   | 239; |
| ......TGAGGTAGTAGATTGTATAGaT............................................................ 230   | 230; |
| ......TGAGGaAGTAGATTGTATAGTT............................................................ 229   | 229; |
| ......TGAGGTAGTAGATTGTATAtTT............................................................ 227   | 227; |
| ......TGAGGTAGTAGATTGTATAGTaa........................................................... 196   | 196; |
| ......TGAGGTAGTAGATT--ATAGTT............................................................ 182   | 182; |
| ......TGAGGTAGTAGATTGTAaAGTT............................................................ 170   | 170; |
| ......TGAGGTAGTAGATTGaATAGTT............................................................ 169   | 169; |
| ......TGAG-TAGTAGATTGTATAGTT............................................................ 161   | 161; |
| ......TGAGGgAGTAGATTGTATAGTT............................................................ 154   | 154; |
| ......TGAGGTAGTAGATTaTATAGTT............................................................ 153   | 153; |
| ......TGAGGTAGTAGAcTGTATAGTT............................................................ 151   | 151; |
| ......TGAGGTAGTAGATTGTATAGTc............................................................ 149   | 149; |
| ......aGAGGTAGTAGATTGTATAGTT............................................................ 146   | 146; |
| ......TGAGGTAaTAGATTGTATAGTT............................................................ 140   | 140; |
| ......TGAGGTAGTtGATTGTATAGTT............................................................ 133   | 133; |
| ......TtAGGTAGTAGATTGTATAGTT............................................................ 133   | 133; |
| ......TGAGGTAcTAGATTGTATAGTT............................................................ 132   | 132; |
| ......TGtGGTAGTAGATTGTATAGTT............................................................ 132   | 132; |
| ......TGcGGTAGTAGATTGTATAGTT............................................................ 131   | 131; |
| ......TGAGGTAGTAGATTGgATAGTT............................................................ 129   | 129; |
| ......TGAGGTAGgAGATTGTATAGTT............................................................ 129   | 129; |
| ......TGAGGTAGTA--TTGTATAGTT............................................................ 126   | 126; |
| ......TGAGGTAGTAGATTGTATcGTT............................................................ 125   | 125; |
| ......TGAGGTAGTAGATTGTATAGa............................................................. 125   | 125; |
| ......TGAGGTtGTAGATTGTATAGTT............................................................ 124   | 124; |
| ......TGAGGTAGTAGATTGTAcAGTT............................................................ 121   | 121; |
| ......TGAGGTAGTAGATTGTATgGTT............................................................ 116   | 116; |
| ......TGAGGTAGTAGAaTGTATAGTT............................................................ 112   | 112; |
| ......TGAGaTAGTAGATTGTATAGTT............................................................ 109   | 109; |
| ......TGAGGTAGTAGATTGTATtGTT............................................................ 107   | 107; |
| ......TGAGGTAGTcGATTGTATAGTT............................................................ 106   | 106; |
| ......TGAGGTAGTAGATTGTATAGTTc........................................................... 104   | 104; |
| ......TGAGGTAGTAGATTGTcTAGT............................................................. 103   | 103; |
| ......TGAtGTAGTAGATTGTATAGTT............................................................ 99   | 99; |
| ......TGAGGTAGTAGATTGT-TAGTT............................................................ 94   | 94; |
| ......TGAGGTcGTAGATTGTATAGTT............................................................ 93   | 93; |
| ......TGAGGTAGTAGATaGTATAGTT............................................................ 92   | 92; |
| ......TGAaGTAGTAGATTGTATAGTT............................................................ 88   | 88; |
| ......TGAGGTAGTAGtTTGTATAGTT............................................................ 81   | 81; |
| ......TGAGGTAGTAaATTGTATAGTT............................................................ 76   | 76; |
| ......TGAGGTAGTAGATTcTATAGTT............................................................ 75   | 75; |
| ......TGAGGTAGTAGAT-GTATAGTT............................................................ 74   | 74; |
| ......TGAGGTAGTAGcTTGTATAGTT............................................................ 73   | 73; |
| ......TGAGGTgGTAGATTGTATAGT............................................................. 71   | 71; |
| ......TGAGGT--TAGATTGTATAGTT............................................................ 71   | 71; |
| ......TGAGGTAGTAGATTGTATAcTT............................................................ 70   | 70; |
| ......TGAGGTAGTAG-TTGTATAGTT............................................................ 67   | 67; |
| ......TGAGGTAGTAGATTGTgTAGT............................................................. 65   | 65; |
| ......TGAGGcAGTAGATTGTATAGT............................................................. 65   | 65; |
| ......TGgGGTAGTAGATTGTATAGT............................................................. 65   | 65; |
| ......TaAGGTAGTAGATTGTATAGT............................................................. 63   | 63; |
| ......TGAGGTAGTAGATTGTATAaTT............................................................ 62   | 62; |
| .....tTGAGGTAGTAGATTGTATAGT............................................................. 58   | 58; |
| ......TGAGtTAGTAGATTGTATAGTT............................................................ 57   | 57; |
| ......TGAGcTAGTAGATTGTATAGTT............................................................ 55   | 55; |
| ......TGAGGTAtTAGATTGTATAGT............................................................. 55   | 55; |
| ......TGAGGTAGTAtATTGTATAGTT............................................................ 55   | 55; |
| ......TGAGGTAGcAGATTGTATAGT............................................................. 54   | 54; |
| ......cGAGGTAGTAGATTGTATAGT............................................................. 54   | 54; |
| ......TGAGGTAGTgGATTGTATAGT............................................................. 53   | 53; |
| ......TcAGGTAGTAGATTGTATAGTT............................................................ 51   | 51; |
| ......TGAcGTAGTAGATTGTATAGTT............................................................ 48   | 48; |
| ......TGAGGTAGTAGATTGcATAGT............................................................. 45   | 45; |
| ......TGAGGTAGTAGATTGTATAtT............................................................. 44   | 44; |
| ......TGAGGTAGTAGATTGTtTAGT............................................................. 44   | 44; |
| ......TGAGGTAGTAGgTTGTATAGTTG........................................................... 42   | 42; |
| .......tAGGTAGTAGATTGTATAGTT............................................................ 41   | 41; |
| ......TGAGGaAGTAGATTGTATAGT............................................................. 40   | 40; |
| ......TGAGGTAGTAGATTGTATAGc............................................................. 40   | 40; |
| ......TGAGGTAGaAGATTGTATAGT............................................................. 40   | 40; |
| ......TGAGGTAGTAGATTtTATAGTT............................................................ 40   | 40; |
| .......GAGGTAGTAGATTGTATAGTTa........................................................... 39   | 39; |
| ......TGAGGTAGTAGATcGTATAGT............................................................. 39   | 39; |
| ......gGAGGTAGTAGATTGTATAGTT............................................................ 38   | 38; |
| ......TGAGGTAGTAGATTGTATAGg............................................................. 36   | 36; |
| .....cTGAGGTAGTAGATTGTATAGT............................................................. 35   | 35; |
| ......TGAG-TAGTAGATTGTATAGT............................................................. 33   | 33; |
| ......TGAGGTAGTAGATTGTATAGaa............................................................ 32   | 32; |
| ......TGAGGTAGTAGATTGaATAGT............................................................. 31   | 31; |
| ......TGAGGTAGTNGATTGTATAGTTa........................................................... 29   | 29; |
| ......TGAGGTAcTAGATTGTATAGT............................................................. 29   | 29; |
| ......TGAGGTAGTAGATTGTAaAGT............................................................. 27   | 27; |
| ......TGAGGTAGTtGATTGTATAGT............................................................. 27   | 27; |
| ......TGAGGTAGTAGATTGTATAGTg............................................................ 26   | 26; |
| ......TGAGGTANTAGATTGTATAGTTa........................................................... 26   | 26; |
| ......TGAGGT-GTAGATTGTATAGTT............................................................ 25   | 25; |
| ......TGAGGTAGTAGATTNTATAGTTa........................................................... 25   | 25; |
| ......TGAGGTAGTAGATT--ATAGT............................................................. 25   | 25; |
| ......TG-GGTAGTAGATTGTATAGTT............................................................ 25   | 25; |
| .....tTGAGGTAGTAGATTGTATAG.............................................................. 25   | 25; |
| ......TGcGGTAGTAGATTGTATAGT............................................................. 25   | 25; |
| ......TGAGGTAGTAGATTGgATAGgT............................................................ 24   | 24; |
| ......TGAGGTAGTAGATTGgATAGT............................................................. 23   | 23; |
| ......TtAGGTAGTAGATTGTATAGT............................................................. 22   | 22; |
| ......TGAGGgAGTAGATTGTATAGT............................................................. 22   | 22; |
| ......TGAGGTAGTAcATTGTATAGTT............................................................ 22   | 22; |
| ......TGAGGTAGTAGATTGTATcGT............................................................. 22   | 22; |
| ......TGAGGTAGgAGATTGTATAGT............................................................. 22   | 22; |
| ......TGAGGTAaTAGATTGTATAGT............................................................. 22   | 22; |
| ......TGAGGTAGTAGATTGTATtGT............................................................. 22   | 22; |
| ......TGAGaTAGTAGATTGTATAGT............................................................. 21   | 21; |
| ......TGAGGTAGTA--TTGTATAGT............................................................. 21   | 21; |
| ......TGAGGTAGTAGATT-TATAGTT............................................................ 19   | 19; |
| ......TGAGGTAGTAGATTGTATgGT............................................................. 19   | 19; |
| ......TGAGGTAGTAGATTGTAcAGT............................................................. 19   | 19; |
| ......TGAGGTAGTAGATgGTATAGTT............................................................ 19   | 19; |
| ......aGAGGTAGTAGATTGTATAGT............................................................. 19   | 19; |
| ......TGtGGTAGTAGATTGTATAGT............................................................. 19   | 19; |
| ......TGAGGTtGTAGATTGTATAGT............................................................. 17   | 17; |
| ......TGAGGTAGTAGATTaTATAGT............................................................. 17   | 17; |
| ......TGAGGTAGTcGATTGTATAGT............................................................. 16   | 16; |
| ......TGAGG--GTAGATTGTATAGTT............................................................ 16   | 16; |
| ......TGAGGTAGTAGAcTGTATAGT............................................................. 16   | 16; |
| ......TGAtGTAGTAGATTGTATAGT............................................................. 16   | 16; |
| ......TGAGGTAGTAGAaTGTATAGT............................................................. 15   | 15; |
| ......TGAGGTAGTAGATTGTATAaT............................................................. 15   | 15; |
| ......NGAGGTAGTAGATTGTATAGTTa........................................................... 15   | 15; |
| ......TGAaGTAGTAGATTGTATAGT............................................................. 15   | 15; |
| ....AGTtGAGGTAGTAGATTGTAT............................................................... 15   | 15; |
| ......TGAGGTAGTAaATTGTATAGT............................................................. 15   | 15; |
| ......TGAGGTAGT-GATTGTATAGTT............................................................ 14   | 14; |
| ......TGAGGTAGTAGATTcTATAGT............................................................. 14   | 14; |
| ......TGAGGTAGTAGATTG-ATAGTT............................................................ 14   | 14; |
| ......TGAGGTA-TAGATTGTATAGTT............................................................ 13   | 13; |
| ......TGAGGTAGTAGATTGTcTAGTTa........................................................... 13   | 13; |
| ......TGAGGTAGTAGATaGTATAGT............................................................. 12   | 12; |
| ........AGGTAGTAGATTGTATAGTTa........................................................... 12   | 12; |
| ......TGAcGTAGTAGATTGTATAGT............................................................. 12   | 12; |
| ......TGAGGTcGTAGATTGTATAGT............................................................. 12   | 12; |
| ......TGAGGTAGTAGtTTGTATAGT............................................................. 12   | 12; |
| ....ttTGAGGTAGTAGATTGTATAG.............................................................. 12   | 12; |
| ......TGAGGT--TAGATTGTATAGT............................................................. 12   | 12; |
| ......TGAGGTAGTAGATTGTgTAGTTa........................................................... 11   | 11; |
| ......TGAGGTAGTAGATTtTATAGT............................................................. 11   | 11; |
| ......TGAGcTAGTAGATTGTATAGT............................................................. 11   | 11; |
| ......TGAGGTAG-AGATTGTATAGTT............................................................ 11   | 11; |
| ......TGAGGTAGTAGcTTGTATAGT............................................................. 10   | 10; |
| ......TGAGGTAGTAGATTGTAT-GTT............................................................ 10   | 10; |
| ......TGAGGTAGgAGATTGTATAGgT............................................................ 9   | 9; |
| ......TGAGGTAGT-G-TTGTATAGTT............................................................ 9   | 9; |
| ......TGAGGgAGTAGATTGgATAGTT............................................................ 9   | 9; |
| ......TGAGGTAGTAGtATTGTATAGTT........................................................... 9   | 9; |
| ......TGAGGTAGTAGAT-GTATAGT............................................................. 9   | 9; |
| ......TGAGGTgGTAGATTGTATAGTTa........................................................... 9   | 9; |
| ......TGAGGTAGcAGATTGTATAGTTa........................................................... 8   | 8; |
| ......TGAGGcAGTAGATTGTATAGTTa........................................................... 8   | 8; |
| ......TGgGGTAGTAGATTGTATAGTTa........................................................... 8   | 8; |
| ......TGAGGTAGTAGATTGgATAGaT............................................................ 8   | 8; |
| ......cGAGGTAGTAGATTGTATAGTTa........................................................... 8   | 8; |
| ......TGAG-TAGTAGATTGTATAGTTa........................................................... 8   | 8; |
| ......TGAGGTAGTAG-TTGTATAGT............................................................. 8   | 8; |
| ......TGAGGTAGTAGATTGT-TAGTTa........................................................... 7   | 7; |
| ......TGAGGTAGTAGATTGTATAaa............................................................. 7   | 7; |
| ......TG-GGTAGTAGATTGTATAGT............................................................. 7   | 7; |
| ......TGAGGTAGTAGATcGTATAGTTa........................................................... 7   | 7; |
| ......TGAGGTAGTAGATTGaATAGgT............................................................ 7   | 7; |
| ......TGcGGTAGTAGATTGTATAGgT............................................................ 7   | 7; |
| .....cTGAGGTAGTAGATTGTATAG.............................................................. 7   | 7; |
| ......TaAGGTAGTAGATTGTATAGTTa........................................................... 6   | 6; |
| ......TGAGGTAGTAGANTGTATAGTTa........................................................... 6   | 6; |
| ......TGAGGTAGTgGATTGTATAGTTa........................................................... 6   | 6; |
| ......TGAGGTAGTAGATTGTAT-GT............................................................. 6   | 6; |
| ......TGAGGTAGTAGAgTGTATAGTT............................................................ 6   | 6; |
| ......TGAGGTAGTAGATTGT-TAGT............................................................. 6   | 6; |
| ......TGAGGTAGTAGATTGTATAcT............................................................. 6   | 6; |
| ......TGAGGTAtTAGATTGTATAGTTa........................................................... 6   | 6; |
| ......TGAGGTAGTAGATTGTAgAGTT............................................................ 6   | 6; |
| ......TGAGGTAGTAGATTGgATAGg............................................................. 6   | 6; |
| ......TGAGGTAGTAGATT-TATAGT............................................................. 6   | 6; |
| ......TGcGGTAGTAGATTGgATAGTT............................................................ 5   | 5; |
| ......TGAGGTAGTAtATTGTATAGT............................................................. 5   | 5; |
| ......TGAGG-AGTAGATTGTATAGTT............................................................ 5   | 5; |
| ......gGAGGTAGTAGATTGTATAGT............................................................. 5   | 5; |
| ......TGAGGcAGTAGATTGgATAGTT............................................................ 5   | 5; |
| ......TcAGGTAGTAGATTGTATAGT............................................................. 5   | 5; |
| ......TGAGGTAGaAGATTGTATAGgT............................................................ 5   | 5; |
| ......TGAGGTAGTAGATTGgATAGcT............................................................ 5   | 5; |
| ......TGAGGaAGTAGATTGgATAGTT............................................................ 5   | 5; |
| ......TGAGGTAGTAcATTGTATAGT............................................................. 5   | 5; |
| ......TGAGGTAGTAGATTtGTATAGTT........................................................... 5   | 5; |
| ......TGAGGTAGgAGATTGTATAGaT............................................................ 5   | 5; |
| ......TGAGGT-GTAGATTGTATAGT............................................................. 5   | 5; |
| ......TGAGGTAGTAGATTGaATAGaT............................................................ 5   | 5; |
| ......TGAGGTAGTAGATTGTATgGTTa........................................................... 4   | 4; |
| ......TGAGGTAGTNGATTGTATAGTa............................................................ 4   | 4; |
| ......TGcGGTAGTAGATTGTATAGTTa........................................................... 4   | 4; |
| ......TGAGGgAGTAGATTGTATAGTTa........................................................... 4   | 4; |
| ......TGAGGTAGaAGATTGTATAGaT............................................................ 4   | 4; |
| ......TGAGGTAGcAGATTGTATAGgT............................................................ 4   | 4; |
| ......TGAGGTAGTAGATTGTATAtTTa........................................................... 4   | 4; |
| ......TGAGGTAGTAGATTGcATAGTTa........................................................... 4   | 4; |
| ......TGAGGTAGTAGATTGTNTAGTTa........................................................... 4   | 4; |
| ......TGAGGTAGTAGATTGTcTcGTT............................................................ 4   | 4; |
| ......TGgGGTAGTAGATTGTATAGgT............................................................ 4   | 4; |
| ......TGAGGTAGTAGATTGTATAGaTa........................................................... 3   | 3; |
| ......TGAaGTAGTAGATTGTATAGTTa........................................................... 3   | 3; |
| ......TGAGGTAGgAGATTGgATAGTT............................................................ 3   | 3; |
| ......TGAGGTAGTAGATTGTATANTTa........................................................... 3   | 3; |
| ......TGAGGTAGTAGATTGTATtGTTa........................................................... 3   | 3; |
| ......TGAGGTAcTAGATTGTATAGTTa........................................................... 3   | 3; |
| ......TGAGGTAGTAGAcTGTATAGTTa........................................................... 3   | 3; |
| ......TGgGGTAGTAGATTGTATAGcT............................................................ 3   | 3; |
| ......TGAGGTAGTAGATgGTATAGT............................................................. 3   | 3; |
| ......TGAGtTAGTAGATTGTATAGT............................................................. 3   | 3; |
| ......TGAGGaAGTAGATTGTATAGTTa........................................................... 3   | 3; |
| ......TGAGGTAGTAGATTGTATttT............................................................. 3   | 3; |
| ......TtAGGTAGTAGATTGTATAGTTa........................................................... 3   | 3; |
| ......TGAGGTAaTAGATTGTATAGTTa........................................................... 3   | 3; |
| ......TGAGGTAGTAGATTGTATNGTTa........................................................... 3   | 3; |
| ......TGcGGTAGTAGATTGgATAGT............................................................. 3   | 3; |
| ......TGAGGTAGTAGATTGaATAGTTa........................................................... 3   | 3; |
| ......TGgGGTAGTAGATTGTATAGaT............................................................ 3   | 3; |
| ......TGAGGTAGTAGATTGTAaAGTTa........................................................... 3   | 3; |
| ......TGAGGTAGTAGATTGcATAGgT............................................................ 3   | 3; |
| ......TGAGGTAGTAGATTGNATAGTTa........................................................... 3   | 3; |
| ......aGAGGTAGTAGATTGTATAGTTa........................................................... 3   | 3; |
| ......TGAGGTAGaAGATTGTATAGTTa........................................................... 3   | 3; |
| ......TGAGGTAGTANATTGTATAGTTa........................................................... 3   | 3; |
| ......TGAGGTAGTAGATTGgATAGa............................................................. 3   | 3; |
| ......TGANGTAGTAGATTGTATAGTTa........................................................... 3   | 3; |
| ......TGcGGTAGTAGATTGaATAGTT............................................................ 3   | 3; |
| ......TGAGGTAGTAGATTGTtTAGTTa........................................................... 3   | 3; |
| ......TGAGGagGTAGATTGTATAGTT............................................................ 3   | 3; |
| ......TGAGGgAGTAGATTGaATAGTT............................................................ 3   | 3; |
| ......TGAGGTAGgAGATTGTATAGTTa........................................................... 2   | 2; |
| ......TGAGaTAGTAGATTGTATAGTTa........................................................... 2   | 2; |
| ......TGAGGTANTAGATTGTATAGTa............................................................ 2   | 2; |
| ......TGAGGTcGgAGATTGTATAGTT............................................................ 2   | 2; |
| ......TGAGGaAGTAGATTGaATAGTT............................................................ 2   | 2; |
| ......TGAGGTAGTNGATTGTgTAGTT............................................................ 2   | 2; |
| ......TGAGGTAGTAGATTNTATAGTa............................................................ 2   | 2; |
| ......cGAGGTANTAGATTGTATAGTT............................................................ 2   | 2; |
| ......TGAGtTAGTAGATTGTATAGTTa........................................................... 2   | 2; |
| ......TGAGGTAGTAGATTGTAgAGT............................................................. 2   | 2; |
| ......TGAGNTAGTAGATTGTATAGTTa........................................................... 2   | 2; |
| ......TGtGGTAGTAGATTGgATAGTT............................................................ 2   | 2; |
| ......TGAGGTAGaAGATTGTcTAGTT............................................................ 2   | 2; |
| ......TGAGGcAGTAGATTGTATAGgT............................................................ 2   | 2; |
| ......TGAGGTAGTAGATTGTATcGaT............................................................ 2   | 2; |
| ......TGAGGTAGgAGATTGTATAGcT............................................................ 2   | 2; |
| ......TGAGGTAGTcGATTGTATAGTTa........................................................... 2   | 2; |
| ......TaAGGTAGTAGATTNTATAGTT............................................................ 2   | 2; |
| .......GAGGTAGcAGATTGTATAGTT............................................................ 2   | 2; |
| .......GAGGTAGTAGATTGTATAGTa............................................................ 2   | 2; |
| ......TGAGGTAGTcGATTGTATcGTT............................................................ 2   | 2; |
| ......TGAGGTAGTAGATTGgATAGTTa........................................................... 2   | 2; |
| ......TGAGGTAGTAGATTGaATAGcT............................................................ 2   | 2; |
| ......TGAGGgAGTAGATTGTATAGaT............................................................ 2   | 2; |
| ......TGAGGTAGNAGATTGTATAGTTa........................................................... 2   | 2; |
| ......TGAGGaAGTAGATTGTATAGgT............................................................ 2   | 2; |
| ......TGAGGTAGTtGATTGTATAGTTa........................................................... 2   | 2; |
| ......TGAGGTNGTAGATTGTATAGTTa........................................................... 2   | 2; |
| ......TGAGGaAGgAGATTGTATAGTT............................................................ 1   | 1; |
| ......TGAGGTAGTAGcTTGTATAGaT............................................................ 1   | 1; |
| ......TGAGGTAGTAGtTTGTATAGTTa........................................................... 1   | 1; |
| ......TGAGGTAGTAGcTTGTATAGTTa........................................................... 1   | 1; |
| ......TGAGGTAGTAGNTTGTATAGTTa........................................................... 1   | 1; |
| ......TGAGGcAGTAGcTTGTATAGTT............................................................ 1   | 1; |
| ......TGAGGTAGTAGcTTGTATAGgT............................................................ 1   | 1; |
| ......TGAGGTAGTAGcTTGTATcGTT............................................................ 1   | 1; |
| --------------------------------------------------------------------------------------- 25610 |
| --------------------------------------------------------------------------------------- 625115 |
| hsa-let-7f-5p(hsa-let-7f-2) TGTGGGATGAGGTAGTAGATTGTATAGTTTTAGGGTCATACCCCATCTTGGAGATAACTATACAGTCTACTGTCTTTCCCACG .((((((.(((..((((((((((((((((...(((.....))).(((.....)))))))))))))))))))..))))))))). (-40.70) \*\*\*\*\*\*\*TGAGGTAGTAGATTGTATAGTT\*\*\*\*\*\*\*\*\*\*\*\*\*\*\*\*\*\*\*\*\*\*\*\*\*\*\*\*\*\*\*\*\*\*\*\*\*\*\*\*\*\*\*\*\*\*\*\*\*\*\*\*\*\* T  M |
| .......TGAGGTAGTAGATTGTATAGTT.......................................................\* 483550   | 483550; |
| .......TGAGGTAGTAGATTGTATAGT........................................................ 77126   | 77126; |
| .......TGAGGTAGTAGATTGTATAGTTT...................................................... 18085   | 18085; |
| .......TGAGGTAGTAGATTGTATAG......................................................... 9010   | 9010; |
| .......TGAGGTAGTAGATTGTAT........................................................... 3470   | 3470; |
| .......TGAGGTANTAGATTGTATAGTT....................................................... 3379   | 3379; |
| .......TGAGGTAGTAGATTGTATA.......................................................... 3032   | 3032; |
| .......TGAGGTAGTNGATTGTATAGTT....................................................... 2959   | 2959; |
| .......TGAGGTAGTAGATTNTATAGTT....................................................... 2740   | 2740; |
| .......NGAGGTAGTAGATTGTATAGTT....................................................... 1577   | 1577; |
| ........GAGGTAGTAGATTGTATAGTT....................................................... 1383   | 1383; |
| .......TGAGGTAGTAGATTGTA............................................................ 768   | 768; |
| .......TGAGGTANTAGATTGTATAGT........................................................ 506   | 506; |
| .......TGAGGTAGTNGATTGTATAGT........................................................ 481   | 481; |
| .......TGAGGTAGTAGATTNTATAGT........................................................ 423   | 423; |
| .......TGAGGTAGTAGNTTGTATAGTT....................................................... 391   | 391; |
| .......TNAGGTAGTAGATTGTATAGTT....................................................... 368   | 368; |
| .......TGAGGTAGTAGATTGTNTAGTT....................................................... 368   | 368; |
| .......TGAGGTAGNAGATTGTATAGTT....................................................... 359   | 359; |
| .......TGAGGTAGTAGA................................................................. 359   | 359; |
| .......TGAGNTAGTAGATTGTATAGTT....................................................... 316   | 316; |
| .......TGAGGTAGTAGATTGTATAGTN....................................................... 316   | 316; |
| .......TGAGGTNGTAGATTGTATAGTT....................................................... 313   | 313; |
| .......TGAGGTAGTAGATTGTANAGTT....................................................... 308   | 308; |
| .......TGAGGTAGTAGATTGT............................................................. 308   | 308; |
| .......TGAGGNAGTAGATTGTATAGTT....................................................... 308   | 308; |
| .......TGAGGTAGTANATTGTATAGTT....................................................... 308   | 308; |
| .........AGGTAGTAGATTGTATAGTT....................................................... 307   | 307; |
| .......TGANGTAGTAGATTGTATAGTT....................................................... 306   | 306; |
| .......TGNGGTAGTAGATTGTATAGTT....................................................... 289   | 289; |
| .......TGAGGTAGTAGATTGTATAGNT....................................................... 282   | 282; |
| .......TGAGGTAGTAGATTGNATAGTT....................................................... 281   | 281; |
| .......NGAGGTAGTAGATTGTATAGT........................................................ 281   | 281; |
| .......TGAGGTAGTAGATNGTATAGTT....................................................... 281   | 281; |
| .......TGAGGTAGTAGANTGTATAGTT....................................................... 279   | 279; |
| .......TGAGGTAGTAGATTGTATANTT....................................................... 277   | 277; |
| .......TGAGGTAGTAGATTGTATNGTT....................................................... 277   | 277; |
| ........GAGGTAGTAGATTGTATAGT........................................................ 216   | 216; |
| .......TGAGGTAGTAGNTTGTATAGT........................................................ 82   | 82; |
| ........GAGGTAGTAGATTGTATAGTTT...................................................... 79   | 79; |
| ......ATGAGGTAGTAGATTGTATAGTT....................................................... 79   | 79; |
| .......TGAGGTAGTAGATTGTNTAGT........................................................ 69   | 69; |
| .......TGAGGTAGTAGATTG.............................................................. 68   | 68; |
| .......TGAGGTANTAGATTGTATAG......................................................... 68   | 68; |
| .......TGAGGTAGNAGATTGTATAGT........................................................ 62   | 62; |
| .......TNAGGTAGTAGATTGTATAGT........................................................ 60   | 60; |
| .......TGAGGNAGTAGATTGTATAGT........................................................ 60   | 60; |
| .......TGAGGTANTAGATTGTATAGTTT...................................................... 58   | 58; |
| .......TGAGGTAGTNGATTGTATAGTTT...................................................... 55   | 55; |
| .......TGAGGTAGTNGATTGTATAG......................................................... 55   | 55; |
| ......ATGAGGTAGTAGATTGTATAGT........................................................ 54   | 54; |
| .......TGAGGTAGTAGATNGTATAGT........................................................ 52   | 52; |
| .......TGAGGTNGTAGATTGTATAGT........................................................ 51   | 51; |
| .......TGAGGTAGTAGATTNTATAG......................................................... 51   | 51; |
| .......TGAGGTAGTAGATTNTATAGTTT...................................................... 50   | 50; |
| .......TGAGGTAGTANATTGTATAGT........................................................ 50   | 50; |
| .......TGAGGTAGTAGATTGTATANT........................................................ 49   | 49; |
| .......TGANGTAGTAGATTGTATAGT........................................................ 48   | 48; |
| .......TGAGGTAGTAGATTGNATAGT........................................................ 47   | 47; |
| .......TGNGGTAGTAGATTGTATAGT........................................................ 47   | 47; |
| .......TGAGGTAGTAGANTGTATAGT........................................................ 47   | 47; |
| .......NGAGGTAGTAGATTGTATAGTTT...................................................... 45   | 45; |
| .......TGAGGTAGTAGATTGTANAGT........................................................ 45   | 45; |
| .......TGAGGTAGTAGATTGTATNGT........................................................ 45   | 45; |
| .......TGAGNTAGTAGATTGTATAGT........................................................ 44   | 44; |
| .......TGAGGTAGTAGATTGTATAGTTTT..................................................... 42   | 42; |
| .......TGAGGTAGTAGATTGTATAGN........................................................ 40   | 40; |
| .......NGAGGTAGTAGATTGTATAG......................................................... 39   | 39; |
| .........AGGTAGTAGATTGTATAGT........................................................ 34   | 34; |
| .......TGAGGTAGTAGATTNTATA.......................................................... 25   | 25; |
| .......TGAGGTAGTNGATTGTAT........................................................... 21   | 21; |
| ........GAGGTAGTAGATTGTATAG......................................................... 20   | 20; |
| .......TGAGGTANTAGATTGTAT........................................................... 19   | 19; |
| .......TGAGGTAGTAGATTNTAT........................................................... 19   | 19; |
| .......TGAGGTAGTNGATTGTATA.......................................................... 17   | 17; |
| .......TGAGGTANTAGATTGTATA.......................................................... 17   | 17; |
| .......NGAGGTAGTAGATTGTAT........................................................... 16   | 16; |
| .........AGGTAGTAGATTGTATAGTTT...................................................... 15   | 15; |
| .......TGAGGTAGTAGATT............................................................... 14   | 14; |
| ........GAGGTAGNAGATTGTATAGTT....................................................... 12   | 12; |
| .......TGAGGTAGTAGATTGTANAGTTT...................................................... 11   | 11; |
| .......TGAGGTAGTAGATTGTNTAG......................................................... 10   | 10; |
| .......TGAGGNAGTAGATTGTATAG......................................................... 10   | 10; |
| .......TGAGGTAGTAGNTTGTATAGTTT...................................................... 10   | 10; |
| ........GAGGTAGTANATTGTATAGTT....................................................... 10   | 10; |
| ........GAGGTAGTAGATTGNATAGTT....................................................... 10   | 10; |
| .......TGANGTAGTAGATTGTATAGTTT...................................................... 9   | 9; |
| .......TGAGGTAGTAGATTGNATAG......................................................... 9   | 9; |
| .......NGAGGTAGTAGATTGTATA.......................................................... 9   | 9; |
| .......TGAGGTAGTAGATTGNATAGTTT...................................................... 9   | 9; |
| .......TGAGGTNGTAGATTGTATAGTTT...................................................... 8   | 8; |
| .......TGAGGTNGTAGATTGTATAG......................................................... 8   | 8; |
| .......TGNGGTAGTAGATTGTATAG......................................................... 8   | 8; |
| .......TGAGGTAGTAGATTGTATANTTT...................................................... 8   | 8; |
| .......TGAGGTAGTAGAT................................................................ 8   | 8; |
| .......TGAGGTAGTAGATTGTATAGTTN...................................................... 8   | 8; |
| ......ATGAGGTAGTAGATTGTATAG......................................................... 8   | 8; |
| .......TGAGGTAGTAGNTTGTATAG......................................................... 7   | 7; |
| ........GAGGTAGTAGATTGTAT........................................................... 7   | 7; |
| ........NAGGTAGTAGATTGTATAGTT....................................................... 7   | 7; |
| .......TGAGGTAGNAGATTGTATAGTTT...................................................... 6   | 6; |
| .......TGAGGTAGTANATTGTATAG......................................................... 6   | 6; |
| .......TGAGGTANTAGATTGTA............................................................ 6   | 6; |
| .......TGAGGTAGTANATTGTATAGTTT...................................................... 6   | 6; |
| .......TGANGTAGTAGATTGTATAG......................................................... 6   | 6; |
| ...........GTAGTAGATTGTATAGTT....................................................... 6   | 6; |
| .......TGAGGTAGTAGATNGTATAGTTT...................................................... 6   | 6; |
| .......TGAGGTAGTAGANTGTATAG......................................................... 6   | 6; |
| .......TNAGGTAGTAGATTGTATAGTTT...................................................... 6   | 6; |
| .......TGAGGNAGTAGATTGTATAGTTT...................................................... 5   | 5; |
| ........GAGGTAGTAGATTGTATA.......................................................... 5   | 5; |
| .......TGAGGTAGTAGATTGTNTAGTTT...................................................... 5   | 5; |
| .......TGAGNTAGTAGATTGTATAG......................................................... 5   | 5; |
| .......TGNGGTAGTAGATTGTATAGTTT...................................................... 5   | 5; |
| ..........GGTAGTAGATTGTATAGTT....................................................... 5   | 5; |
| .......TGAGGTAGTAGATNGTATA.......................................................... 5   | 5; |
| .......TGAGGTAGTAGATTGTATNG......................................................... 5   | 5; |
| .......TGAGGTAGTAGATTGTATAGTNT...................................................... 5   | 5; |
| .......TNAGGTAGTAGATTGTATAG......................................................... 5   | 5; |
| .......TGAGGTAGNAGATTGTATAG......................................................... 5   | 5; |
| .......TGAGGTAGTAGATNGTATAG......................................................... 5   | 5; |
| .............AGTAGATTGTATAGTT....................................................... 4   | 4; |
| .........AGGTAGTAGATTGTATAG......................................................... 3   | 3; |
| .......TGAGGTAGTAG.................................................................. 1   | 1; |
| ----------------------------------------------------------------------------------- 617753 |
| .......TGAGGTAGTAGATTGTATAGTTa...................................................... 9259   | 9259; |
| .......TGAGGTAGTAGATTGTATAGTa....................................................... 871   | 871; |
| .......TGAGGTAGTAGATTGTA--GTT....................................................... 643   | 643; |
| .......TGAGGTAGTAGATTGTcTAGTT....................................................... 607   | 607; |
| .......TGAGGTAGTAGATTGTgTAGTT....................................................... 503   | 503; |
| .......TGAGGcAGTAGATTGTATAGTT....................................................... 447   | 447; |
| .......TGAGGTgGTAGATTGTATAGTT....................................................... 428   | 428; |
| .......TaAGGTAGTAGATTGTATAGTT....................................................... 403   | 403; |
| .......TGgGGTAGTAGATTGTATAGTT....................................................... 392   | 392; |
| .......TGAGGTAGTgGATTGTATAGTT....................................................... 337   | 337; |
| .......TGAGGTAtTAGATTGTATAGTT....................................................... 324   | 324; |
| .......cGAGGTAGTAGATTGTATAGTT....................................................... 322   | 322; |
| .......TGAGGTAGcAGATTGTATAGTT....................................................... 319   | 319; |
| .......TGAGGTAGTAGATTGTATAGgT....................................................... 270   | 270; |
| .......TGAGGTAGTAGATTGTtTAGTT....................................................... 266   | 266; |
| ......cTGAGGTAGTAGATTGTATAGTT....................................................... 265   | 265; |
| .......TGAGGTAGTAGATTGTATAGcT....................................................... 264   | 264; |
| .......TGAGGTAGaAGATTGTATAGTT....................................................... 245   | 245; |
| .......TGAGGTAGTAGATcGTATAGTT....................................................... 241   | 241; |
| .......TGAGGTAGTAGATTGcATAGTT....................................................... 239   | 239; |
| .......TGAGGTAGTAGATTGTATAGaT....................................................... 230   | 230; |
| .......TGAGGaAGTAGATTGTATAGTT....................................................... 229   | 229; |
| .......TGAGGTAGTAGATTGTATAtTT....................................................... 227   | 227; |
| .......TGAGGTAGTAGATTGTATAGTaa...................................................... 196   | 196; |
| .......TGAGGTAGTAGATT--ATAGTT....................................................... 182   | 182; |
| .......TGAGGTAGTAGATTGTAaAGTT....................................................... 170   | 170; |
| .......TGAGGTAGTAGATTGaATAGTT....................................................... 169   | 169; |
| .......TGAG-TAGTAGATTGTATAGTT....................................................... 161   | 161; |
| .......TGAGGgAGTAGATTGTATAGTT....................................................... 154   | 154; |
| .......TGAGGTAGTAGATTaTATAGTT....................................................... 153   | 153; |
| .......TGAGGTAGTAGAcTGTATAGTT....................................................... 151   | 151; |
| .......TGAGGTAGTAGATTGTATAGTc....................................................... 149   | 149; |
| .......aGAGGTAGTAGATTGTATAGTT....................................................... 146   | 146; |
| .......TGAGGTAaTAGATTGTATAGTT....................................................... 140   | 140; |
| .......TtAGGTAGTAGATTGTATAGTT....................................................... 133   | 133; |
| .......TGAGGTAGTtGATTGTATAGTT....................................................... 133   | 133; |
| .......TGtGGTAGTAGATTGTATAGTT....................................................... 132   | 132; |
| .......TGAGGTAcTAGATTGTATAGTT....................................................... 132   | 132; |
| .......TGcGGTAGTAGATTGTATAGTT....................................................... 131   | 131; |
| .......TGAGGTAGTAGATTGgATAGTT....................................................... 129   | 129; |
| .......TGAGGTAGgAGATTGTATAGTT....................................................... 129   | 129; |
| .......TGAGGTAGTA--TTGTATAGTT....................................................... 126   | 126; |
| .......TGAGGTAGTAGATTGTATAGa........................................................ 125   | 125; |
| .......TGAGGTAGTAGATTGTATcGTT....................................................... 125   | 125; |
| .......TGAGGTtGTAGATTGTATAGTT....................................................... 124   | 124; |
| .......TGAGGTAGTAGATTGTAcAGTT....................................................... 121   | 121; |
| .......TGAGGTAGTAGATTGTATgGTT....................................................... 116   | 116; |
| .......TGAGGTAGTAGAaTGTATAGTT....................................................... 112   | 112; |
| .......TGAGaTAGTAGATTGTATAGTT....................................................... 109   | 109; |
| .......TGAGGTAGTAGATTGTATtGTT....................................................... 107   | 107; |
| .......TGAGGTAGTcGATTGTATAGTT....................................................... 106   | 106; |
| .......TGAGGTAGTAGATTGTATAGTTc...................................................... 104   | 104; |
| .......TGAGGTAGTAGATTGTcTAGT........................................................ 103   | 103; |
| .......TGAtGTAGTAGATTGTATAGTT....................................................... 99   | 99; |
| .......TGAGGTAGTAGATTGT-TAGTT....................................................... 94   | 94; |
| .......TGAGGTcGTAGATTGTATAGTT....................................................... 93   | 93; |
| .......TGAGGTAGTAGATaGTATAGTT....................................................... 92   | 92; |
| .......TGAaGTAGTAGATTGTATAGTT....................................................... 88   | 88; |
| .......TGAGGTAGTAGtTTGTATAGTT....................................................... 81   | 81; |
| .......TGAGGTAGTAaATTGTATAGTT....................................................... 76   | 76; |
| .......TGAGGTAGTAGATTcTATAGTT....................................................... 75   | 75; |
| .......TGAGGTAGTAGAT-GTATAGTT....................................................... 74   | 74; |
| .......TGAGGTAGTAGcTTGTATAGTT....................................................... 73   | 73; |
| .......TGAGGT--TAGATTGTATAGTT....................................................... 71   | 71; |
| .......TGAGGTgGTAGATTGTATAGT........................................................ 71   | 71; |
| .......TGAGGTAGTAGATTGTATAcTT....................................................... 70   | 70; |
| .......TGAGGTAGTAG-TTGTATAGTT....................................................... 67   | 67; |
| .......TGAGGTAGTAGATTGTgTAGT........................................................ 65   | 65; |
| .......TGAGGcAGTAGATTGTATAGT........................................................ 65   | 65; |
| .......TGgGGTAGTAGATTGTATAGT........................................................ 65   | 65; |
| .......TaAGGTAGTAGATTGTATAGT........................................................ 63   | 63; |
| .......TGAGGTAGTAGATTGTATAaTT....................................................... 62   | 62; |
| ......tTGAGGTAGTAGATTGTATAGT........................................................ 58   | 58; |
| .......TGAGtTAGTAGATTGTATAGTT....................................................... 57   | 57; |
| .......TGAGGTAGTAtATTGTATAGTT....................................................... 55   | 55; |
| .......TGAGcTAGTAGATTGTATAGTT....................................................... 55   | 55; |
| .......TGAGGTAtTAGATTGTATAGT........................................................ 55   | 55; |
| .......cGAGGTAGTAGATTGTATAGT........................................................ 54   | 54; |
| .......TGAGGTAGcAGATTGTATAGT........................................................ 54   | 54; |
| .......TGAGGTAGTgGATTGTATAGT........................................................ 53   | 53; |
| .......TcAGGTAGTAGATTGTATAGTT....................................................... 51   | 51; |
| .......TGAGGTAGTAGATTGTATAGTaT...................................................... 48   | 48; |
| .......TGAcGTAGTAGATTGTATAGTT....................................................... 48   | 48; |
| .......TGAGGTAGTAGATTGcATAGT........................................................ 45   | 45; |
| .......TGAGGTAGTAGATTGTtTAGT........................................................ 44   | 44; |
| .......TGAGGTAGTAGATTGTATAtT........................................................ 44   | 44; |
| ........tAGGTAGTAGATTGTATAGTT....................................................... 41   | 41; |
| .......TGAGGaAGTAGATTGTATAGT........................................................ 40   | 40; |
| .......TGAGGTAGTAGATTtTATAGTT....................................................... 40   | 40; |
| .......TGAGGTAGaAGATTGTATAGT........................................................ 40   | 40; |
| .......TGAGGTAGTAGATTGTATAGc........................................................ 40   | 40; |
| .......TGAGGTAGTAGATcGTATAGT........................................................ 39   | 39; |
| ........GAGGTAGTAGATTGTATAGTTa...................................................... 39   | 39; |
| .......gGAGGTAGTAGATTGTATAGTT....................................................... 38   | 38; |
| .......TGAGGTAGTAGATTGTATAGg........................................................ 36   | 36; |
| ......cTGAGGTAGTAGATTGTATAGT........................................................ 35   | 35; |
| .......TGAG-TAGTAGATTGTATAGT........................................................ 33   | 33; |
| .......TGAGGTAGTAGATTGTATAGaa....................................................... 32   | 32; |
| .......TGAGGTAGTAGATTGaATAGT........................................................ 31   | 31; |
| .......TGAGGTAcTAGATTGTATAGT........................................................ 29   | 29; |
| .......TGAGGTAGTNGATTGTATAGTTa...................................................... 29   | 29; |
| .......TGAGGTAGTAGATTGTATAGgTT...................................................... 28   | 28; |
| .......TGAGGTAGTtGATTGTATAGT........................................................ 27   | 27; |
| .......TGAGGTAGTAGATTGTAaAGT........................................................ 27   | 27; |
| .......TGAGGTANTAGATTGTATAGTTa...................................................... 26   | 26; |
| .......TGAGGTAGTAGATTGTATAGTg....................................................... 26   | 26; |
| .......TGAGGT-GTAGATTGTATAGTT....................................................... 25   | 25; |
| .......TGcGGTAGTAGATTGTATAGT........................................................ 25   | 25; |
| .......TGAGGTAGTAGATT--ATAGT........................................................ 25   | 25; |
| .......TG-GGTAGTAGATTGTATAGTT....................................................... 25   | 25; |
| .......TGAGGTAGTAGATTNTATAGTTa...................................................... 25   | 25; |
| ......tTGAGGTAGTAGATTGTATAG......................................................... 25   | 25; |
| .......TGAGGTAGTAGATTGgATAGgT....................................................... 24   | 24; |
| .......TGAGGTAGTAGATTGgATAGT........................................................ 23   | 23; |
| .......TGAGGTAGTAGATTGTATcGT........................................................ 22   | 22; |
| .......TGAGGTAGTAcATTGTATAGTT....................................................... 22   | 22; |
| .......TtAGGTAGTAGATTGTATAGT........................................................ 22   | 22; |
| .......TGAGGTAaTAGATTGTATAGT........................................................ 22   | 22; |
| .......TGAGGTAGgAGATTGTATAGT........................................................ 22   | 22; |
| .......TGAGGgAGTAGATTGTATAGT........................................................ 22   | 22; |
| .......TGAGGTAGTAGATTGTATtGT........................................................ 22   | 22; |
| .......TGAGGTAGTA--TTGTATAGT........................................................ 21   | 21; |
| .......TGAGaTAGTAGATTGTATAGT........................................................ 21   | 21; |
| .......TGAGGTAGTAGATTGTgTAGTTT...................................................... 20   | 20; |
| .......TGAGGTAGTAGATTGTcTAGTTT...................................................... 19   | 19; |
| .......TGAGGTAGTAGATgGTATAGTT....................................................... 19   | 19; |
| .......TGAGGTAGTAGATTGTATgGT........................................................ 19   | 19; |
| .......TGAGGTAGTAGATT-TATAGTT....................................................... 19   | 19; |
| .......TGAGGTAGTAGATTGTAcAGT........................................................ 19   | 19; |
| .......aGAGGTAGTAGATTGTATAGT........................................................ 19   | 19; |
| .......TGtGGTAGTAGATTGTATAGT........................................................ 19   | 19; |
| .......TGgGGTAGTAGATTGTATAGTTT...................................................... 18   | 18; |
| .......TGAGGTtGTAGATTGTATAGT........................................................ 17   | 17; |
| .......TGAGGTAGTAGATTaTATAGT........................................................ 17   | 17; |
| .......TGAGG--GTAGATTGTATAGTT....................................................... 16   | 16; |
| .......TGAtGTAGTAGATTGTATAGT........................................................ 16   | 16; |
| .......TGAGGTAGTcGATTGTATAGT........................................................ 16   | 16; |
| .......TGAGGTAGTAGAcTGTATAGT........................................................ 16   | 16; |
| .......TGAGGTAGTAaATTGTATAGT........................................................ 15   | 15; |
| .......TGAGGTAGTAGAaTGTATAGT........................................................ 15   | 15; |
| .......TGAaGTAGTAGATTGTATAGT........................................................ 15   | 15; |
| .......NGAGGTAGTAGATTGTATAGTTa...................................................... 15   | 15; |
| .......TGAGGcAGTAGATTGTATAGTTT...................................................... 15   | 15; |
| .......TGAGGTAGTAGATTGTATAaT........................................................ 15   | 15; |
| .......TGAGGTAtTAGATTGTATAGTTT...................................................... 14   | 14; |
| .......TGAGGTAGTAGATTG-ATAGTT....................................................... 14   | 14; |
| .......TGAGGTgGTAGATTGTATAGTTT...................................................... 14   | 14; |
| .......TGAGGTAGTAGATTcTATAGT........................................................ 14   | 14; |
| .......TGAGGTAGTgGATTGTATAGTTT...................................................... 14   | 14; |
| .......TGAGGTAGT-GATTGTATAGTT....................................................... 14   | 14; |
| .......TGAGGTAGTAGATTGTcTAGTTa...................................................... 13   | 13; |
| .......TGAGGTA-TAGATTGTATAGTT....................................................... 13   | 13; |
| .......TGAGGTAGTAGATTGTA--GTTT...................................................... 12   | 12; |
| .......TGAGGTAGTAGATaGTATAGT........................................................ 12   | 12; |
| .........AGGTAGTAGATTGTATAGTTa...................................................... 12   | 12; |
| .......TGAGGTcGTAGATTGTATAGT........................................................ 12   | 12; |
| .......TaAGGTAGTAGATTGTATAGTTT...................................................... 12   | 12; |
| .......TGAGGT--TAGATTGTATAGT........................................................ 12   | 12; |
| .....ttTGAGGTAGTAGATTGTATAG......................................................... 12   | 12; |
| .......TGAGGTAGTAGATcGTATAGTTT...................................................... 12   | 12; |
| .......TGAcGTAGTAGATTGTATAGT........................................................ 12   | 12; |
| .......TGAGGTAGTAGtTTGTATAGT........................................................ 12   | 12; |
| .......TGAGGTAGTAGATTGTATAGaTT...................................................... 12   | 12; |
| .......TGAGGTAGTAGATTtTATAGT........................................................ 11   | 11; |
| .......cGAGGTAGTAGATTGTATAGTTT...................................................... 11   | 11; |
| .......TGAGGTAG-AGATTGTATAGTT....................................................... 11   | 11; |
| .......TGAGcTAGTAGATTGTATAGT........................................................ 11   | 11; |
| .......TGAGGTAGTAGATTGTgTAGTTa...................................................... 11   | 11; |
| .......TGAGGTAGTAGcTTGTATAGT........................................................ 10   | 10; |
| .......TGAGGTAGTAGATTGTAT-GTT....................................................... 10   | 10; |
| .......TGAGGTAGaAGATTGTATAGTTT...................................................... 9   | 9; |
| .......TGAGGTAGT-G-TTGTATAGTT....................................................... 9   | 9; |
| .......TGAGGTAGgAGATTGTATAGgT....................................................... 9   | 9; |
| .......TGAGGTAGTAGtATTGTATAGTT...................................................... 9   | 9; |
| .......TGAGGTAGTAGATTGcATAGTTT...................................................... 9   | 9; |
| .......TGAGGTgGTAGATTGTATAGTTa...................................................... 9   | 9; |
| .......TGAGGTAGTAGAT-GTATAGT........................................................ 9   | 9; |
| .......TGAGGgAGTAGATTGgATAGTT....................................................... 9   | 9; |
| .......TGAGGTAGTAGAcTGTATAGTTT...................................................... 8   | 8; |
| .......TGAG-TAGTAGATTGTATAGTTa...................................................... 8   | 8; |
| .......TGgGGTAGTAGATTGTATAGTTa...................................................... 8   | 8; |
| .......TGAGGTAGTAG-TTGTATAGT........................................................ 8   | 8; |
| .......TGAGGTAGcAGATTGTATAGTTa...................................................... 8   | 8; |
| .......TGAGGcAGTAGATTGTATAGTTa...................................................... 8   | 8; |
| .......TGAGGTAGcAGATTGTATAGTTT...................................................... 8   | 8; |
| .......cGAGGTAGTAGATTGTATAGTTa...................................................... 8   | 8; |
| .......TGAGGTAGTAGATTaTATAGTTT...................................................... 8   | 8; |
| .......TGAGGTAGTAGATTGgATAGaT....................................................... 8   | 8; |
| .......TGAGGTAGgAGATTGTATAGTTT...................................................... 8   | 8; |
| .......TGAGGTAGTAGATTGTAaAGTTT...................................................... 8   | 8; |
| .......TGAGGaAGTAGATTGTATAGTTT...................................................... 8   | 8; |
| .......TGAGGTAGTAGATTGTATAGcTT...................................................... 7   | 7; |
| .......TGAGGTAGTAGATTGTATAaa........................................................ 7   | 7; |
| .....aATGAGGTAGTAGATTGTATAGT........................................................ 7   | 7; |
| .......TGAGGTAGTAGATcGTATAGTTa...................................................... 7   | 7; |
| ......cTGAGGTAGTAGATTGTATAG......................................................... 7   | 7; |
| .......TGAGGTAGTAGATTGT-TAGTTa...................................................... 7   | 7; |
| .......TGAtGTAGTAGATTGTATAGTTT...................................................... 7   | 7; |
| .......TGAGGTAGTAGATTGaATAGgT....................................................... 7   | 7; |
| .......TGAGGTAGTAG-TTGTATAGTTT...................................................... 7   | 7; |
| .......TGcGGTAGTAGATTGTATAGgT....................................................... 7   | 7; |
| .......TG-GGTAGTAGATTGTATAGT........................................................ 7   | 7; |
| .....GtTGAGGTAGTAGATTGTATA.......................................................... 7   | 7; |
| .......TGAGGTAGTgGATTGTATAGTTa...................................................... 6   | 6; |
| .......TGAGGTAGTAGATTGTAT-GT........................................................ 6   | 6; |
| .......TGAGGTAGTAGATTGT-TAGT........................................................ 6   | 6; |
| .......TGAGGTAGTAGATTGgATAGg........................................................ 6   | 6; |
| .......TGAGGTAGTAGANTGTATAGTTa...................................................... 6   | 6; |
| .......TGAGGTAGTAGAgTGTATAGTT....................................................... 6   | 6; |
| .......TGAGGTAGTAGATTGTATAcT........................................................ 6   | 6; |
| .......TGAGGgAGTAGATTGTATAGTTT...................................................... 6   | 6; |
| .......TGAGGTAGTAGATT-TATAGT........................................................ 6   | 6; |
| .......TGAGGTAGTAGATTGTATAGTcT...................................................... 6   | 6; |
| .......TGAGGTAGTAGATTGTtTAGTTT...................................................... 6   | 6; |
| .......TGAGGTAtTAGATTGTATAGTTa...................................................... 6   | 6; |
| .......TaAGGTAGTAGATTGTATAGTTa...................................................... 6   | 6; |
| .......TGAGGTAGTAGATTGTAgAGTT....................................................... 6   | 6; |
| .......TGAGGTAGTAGATTGTATAGTgT...................................................... 5   | 5; |
| .......TGcGGTAGTAGATTGgATAGTT....................................................... 5   | 5; |
| .......TcAGGTAGTAGATTGTATAGT........................................................ 5   | 5; |
| .......TGAGGTAGTAtATTGTATAGT........................................................ 5   | 5; |
| .......TGAGGTAGTAGATTtGTATAGTT...................................................... 5   | 5; |
| .......TGAGGTAGTAGATTGTATcGTTT...................................................... 5   | 5; |
| .......TGAGGTAGTAGATTGaATAGTTT...................................................... 5   | 5; |
| .......TGAGGTAGTAcATTGTATAGT........................................................ 5   | 5; |
| .......TGAGG-AGTAGATTGTATAGTT....................................................... 5   | 5; |
| .......TGAGGcAGTAGATTGgATAGTT....................................................... 5   | 5; |
| .......TGAGGaAGTAGATTGgATAGTT....................................................... 5   | 5; |
| .......TGAGGTAGgAGATTGTATAGaT....................................................... 5   | 5; |
| .......TGAGGTAGTAGATTGgATAGcT....................................................... 5   | 5; |
| .......TGAGGTAGTAGATaGTATAGTTT...................................................... 5   | 5; |
| .......gGAGGTAGTAGATTGTATAGT........................................................ 5   | 5; |
| .......TGAGGTAGaAGATTGTATAGgT....................................................... 5   | 5; |
| .......TGAGGTAGTAGATTGaATAGaT....................................................... 5   | 5; |
| .......TGAGGT-GTAGATTGTATAGT........................................................ 5   | 5; |
| .......TGAGGTAGTAGATTGgATAGTTT...................................................... 5   | 5; |
| .......TGAGGTAGTAGATTGcATAGTTa...................................................... 4   | 4; |
| .......TGAGGTAGaAGATTGTATAGaT....................................................... 4   | 4; |
| .......TGAGGTAGTAGATTGTATAtTTa...................................................... 4   | 4; |
| .......TGcGGTAGTAGATTGTATAGTTa...................................................... 4   | 4; |
| .......TGAGGTAGTNGATTGTATAGTa....................................................... 4   | 4; |
| .......TGAGGTAGcAGATTGTATAGgT....................................................... 4   | 4; |
| .......TGAGGTAGTAGATTGTATgGTTa...................................................... 4   | 4; |
| .......TGAGGTAGTAGATTGTcTcGTT....................................................... 4   | 4; |
| .......TGAGGgAGTAGATTGTATAGTTa...................................................... 4   | 4; |
| .......TGgGGTAGTAGATTGTATAGgT....................................................... 4   | 4; |
| .......TGAGGTAGTAGATTGTNTAGTTa...................................................... 4   | 4; |
| .......TGAGGTAGTAGATTGTtTAGTTa...................................................... 3   | 3; |
| .......TGAGGTAGTAGATTGTAaAGTTa...................................................... 3   | 3; |
| .......TGAGGTAGTAGATTGNATAGTTa...................................................... 3   | 3; |
| .......TGAGGgAGTAGATTGaATAGTT....................................................... 3   | 3; |
| .......TGcGGTAGTAGATTGgATAGT........................................................ 3   | 3; |
| .......TGAGGTAGTAGATTGcATAGgT....................................................... 3   | 3; |
| .......TGAGGTAGTAGATTGTATNGTTa...................................................... 3   | 3; |
| .......TGAGGTAGTAGATgGTATAGT........................................................ 3   | 3; |
| .......TGAGGTAcTAGATTGTATAGTTa...................................................... 3   | 3; |
| .......TGAGGTAGTAGAcTGTATAGTTa...................................................... 3   | 3; |
| .......TGAGGTAGTAGATTGTAcAGTTT...................................................... 3   | 3; |
| .......TGAGGaAGTAGATTGTATAGTTa...................................................... 3   | 3; |
| .......TGANGTAGTAGATTGTATAGTTa...................................................... 3   | 3; |
| .......TtAGGTAGTAGATTGTATAGTTa...................................................... 3   | 3; |
| .......aGAGGTAGTAGATTGTATAGTTa...................................................... 3   | 3; |
| .......TGAGGTAGTAGATTGaATAGTTa...................................................... 3   | 3; |
| .......TGAGGTAGTAGATTGTATttT........................................................ 3   | 3; |
| .......TGAGGTAGTANATTGTATAGTTa...................................................... 3   | 3; |
| .......TGgGGTAGTAGATTGTATAGaT....................................................... 3   | 3; |
| .......TGcGGTAGTAGATTGaATAGTT....................................................... 3   | 3; |
| .......TGAGGTAGgAGATTGgATAGTT....................................................... 3   | 3; |
| .......TGAGGTAGTAGATTGTATAGaTa...................................................... 3   | 3; |
| .......TGAGGagGTAGATTGTATAGTT....................................................... 3   | 3; |
| .......TGgGGTAGTAGATTGTATAGcT....................................................... 3   | 3; |
| .......TGAGGTAGTAGATTGTATtGTTa...................................................... 3   | 3; |
| .......TGAGGTAaTAGATTGTATAGTTa...................................................... 3   | 3; |
| .......TGAGGTAGTAGATTGTATANTTa...................................................... 3   | 3; |
| .......TGAGGTAGaAGATTGTATAGTTa...................................................... 3   | 3; |
| .......TGAGGTAGTAGATTGgATAGa........................................................ 3   | 3; |
| .......TGAaGTAGTAGATTGTATAGTTa...................................................... 3   | 3; |
| .......TGAGtTAGTAGATTGTATAGT........................................................ 3   | 3; |
| .......TGAGGTAGTcGATTGTATAGTTa...................................................... 2   | 2; |
| .......TGAGGTAGTNGATTGTgTAGTT....................................................... 2   | 2; |
| .......TGAGGTAGTAGATTGaATAGcT....................................................... 2   | 2; |
| .......TGAGGTAGTAGATTNTATAGTa....................................................... 2   | 2; |
| .......TGAGGTAGTAGtTTGTATAGTTT...................................................... 2   | 2; |
| .......TGAGGcAGTAGATTGTATAGgT....................................................... 2   | 2; |
| .......TGAGGTNGTAGATTGTATAGTTa...................................................... 2   | 2; |
| .......TGAGGTAGTtGATTGTATAGTTa...................................................... 2   | 2; |
| .......TGAGGTAGTAGATTGTATcGaT....................................................... 2   | 2; |
| .......TGtGGTAGTAGATTGgATAGTT....................................................... 2   | 2; |
| .......TGAGGTAGTcGATTGTATcGTT....................................................... 2   | 2; |
| .......TGAGNTAGTAGATTGTATAGTTa...................................................... 2   | 2; |
| .......TGAGGTAGaAGATTGTcTAGTT....................................................... 2   | 2; |
| .......TGAGGTAGgAGATTGTATAGcT....................................................... 2   | 2; |
| .......TGAGGaAGTAGATTGTATAGgT....................................................... 2   | 2; |
| .......TGAGGTAGTAGcTTGTATAGTTT...................................................... 2   | 2; |
| .......TGAGaTAGTAGATTGTATAGTTa...................................................... 2   | 2; |
| .......TaAGGTAGTAGATTNTATAGTT....................................................... 2   | 2; |
| ........GAGGTAGTAGATTGTATAGTa....................................................... 2   | 2; |
| .......TGAGGTAGgAGATTGTATAGTTa...................................................... 2   | 2; |
| .......TGAGGTANTAGATTGTATAGTa....................................................... 2   | 2; |
| .......TGAGGTcGgAGATTGTATAGTT....................................................... 2   | 2; |
| .......TGAGGTAGTAGATTGTATgGTTT...................................................... 2   | 2; |
| ........GAGGTAGcAGATTGTATAGTT....................................................... 2   | 2; |
| .......TGAGGTAGTAGATTGgATAGTTa...................................................... 2   | 2; |
| .......TGAGtTAGTAGATTGTATAGTTa...................................................... 2   | 2; |
| .......cGAGGTANTAGATTGTATAGTT....................................................... 2   | 2; |
| .......TGAGGaAGTAGATTGaATAGTT....................................................... 2   | 2; |
| .......TGAGGgAGTAGATTGTATAGaT....................................................... 2   | 2; |
| .......TGAGGTAGTAGATTGTAgAGT........................................................ 2   | 2; |
| .......TGAGGTAGNAGATTGTATAGTTa...................................................... 2   | 2; |
| .......TGAGGaAGgAGATTGTATAGTT....................................................... 1   | 1; |
| .......TGAGGTAGTAGtTTGTATAGTTa...................................................... 1   | 1; |
| .......TGAGGTAGTAGcTTGTATAGaT....................................................... 1   | 1; |
| .......TGAGGTAGTAGNTTGTATAGTTa...................................................... 1   | 1; |
| .......TGAGGTAGTAGcTTGTATcGTT....................................................... 1   | 1; |
| .......TGAGGTAGTAGcTTGTATAGgT....................................................... 1   | 1; |
| .......TGAGGcAGTAGcTTGTATAGTT....................................................... 1   | 1; |
| .......TGAGGTAGTAGcTTGTATAGTTa...................................................... 1   | 1; |
| ----------------------------------------------------------------------------------- 25955 |
| ----------------------------------------------------------------------------------- 643708 |
| hsa-let-7g-3p(hsa-let-7g) AGGCTGAGGTAGTAGTTTGTACAGTTTGAGGGTCTATGATACCACCCGGTACAGGAGATAACTGTACAGGCCACTGCCTTGCCA .(((.((((((((.((((((((((((.....((((.((.((((....))))))..)))))))))))))))).))))))))))). (-40.50) \*\*\*\*\*\*\*\*\*\*\*\*\*\*\*\*\*\*\*\*\*\*\*\*\*\*\*\*\*\*\*\*\*\*\*\*\*\*\*\*\*\*\*\*\*\*\*\*\*\*\*\*\*\*\*\*\*\*\*\*\*CTGTACAGGCCACTGCCTTGC\*\* T  M |
| ....TGAGGTAGTAGTTTGTACAGTT........................................................... 79689   | 79689; |
| ....TGAGGTAGTAGTTTGTACAGT............................................................ 12608   | 12608; |
| ....TGAGGTAGTAGTTTGTACAGTTT.......................................................... 940   | 940; |
| ....TGAGGTAGTAGTTTGTACAG............................................................. 918   | 918; |
| ....TGAGGTANTAGTTTGTACAGTT........................................................... 250   | 250; |
| ....TGAGGTAGTNGTTTGTACAGTT........................................................... 247   | 247; |
| ....TGAGGTAGTAGTTTGTAC............................................................... 204   | 204; |
| ....TGAGGTAGTAGTTTNTACAGTT........................................................... 203   | 203; |
| ...CTGAGGTAGTAGTTTGTACAGTT........................................................... 161   | 161; |
| ....TGAGGTAGTAGTTTGTACA.............................................................. 137   | 137; |
| ....NGAGGTAGTAGTTTGTACAGTT........................................................... 122   | 122; |
| .....GAGGTAGTAGTTTGTACAGTT........................................................... 112   | 112; |
| ...CTGAGGTAGTAGTTTGTACAGT............................................................ 44   | 44; |
| ....TGAGGTANTAGTTTGTACAGT............................................................ 41   | 41; |
| ....TGAGGTAGTAGTTTGTA................................................................ 37   | 37; |
| ....TGAGGTAGTNGTTTGTACAGT............................................................ 36   | 36; |
| ......AGGTAGTAGTTTGTACAGTT........................................................... 36   | 36; |
| ....TGAGGTAGTANTTTGTACAGTT........................................................... 35   | 35; |
| ....TGAGGTAGTAGTTTGTNCAGTT........................................................... 34   | 34; |
| ....TGAGGTAGTAGTTTNTACAGT............................................................ 34   | 34; |
| ....TGAGGTAGTAGNTTGTACAGTT........................................................... 32   | 32; |
| ....TNAGGTAGTAGTTTGTACAGTT........................................................... 31   | 31; |
| ....TGAGGTAGTAGTTTGTANAGTT........................................................... 31   | 31; |
| ....TGAGGTAGTAGTTTGTACAGTN........................................................... 30   | 30; |
| ....TGAGNTAGTAGTTTGTACAGTT........................................................... 29   | 29; |
| ....TGAGGTAGTAGTTTGTACANTT........................................................... 27   | 27; |
| ....TGAGGTAGTAGTTTGTACAGNT........................................................... 25   | 25; |
| ....NGAGGTAGTAGTTTGTACAGT............................................................ 25   | 25; |
| ....TGAGGTAGNAGTTTGTACAGTT........................................................... 25   | 25; |
| ....TGAGGTAGTAGTTTGNACAGTT........................................................... 25   | 25; |
| ....TGAGGTNGTAGTTTGTACAGTT........................................................... 25   | 25; |
| ....TGAGGTAGTAGT..................................................................... 22   | 22; |
| ....TGAGGTAGTAGTNTGTACAGTT........................................................... 21   | 21; |
| ....TGANGTAGTAGTTTGTACAGTT........................................................... 21   | 21; |
| ....TGAGGTAGTAGTTTGTACNGTT........................................................... 20   | 20; |
| ....TGNGGTAGTAGTTTGTACAGTT........................................................... 20   | 20; |
| ....TGAGGTAGTAGTTNGTACAGTT........................................................... 20   | 20; |
| ....TGAGGNAGTAGTTTGTACAGTT........................................................... 18   | 18; |
| .....GAGGTAGTAGTTTGTACAGT............................................................ 17   | 17; |
| ....TGAGGTAGTAGTTTG.................................................................. 11   | 11; |
| ....TGAGGTAGTAGTTTGT................................................................. 9   | 9; |
| ....TGAGGTNGTAGTTTGTACAGT............................................................ 8   | 8; |
| ....TGAGGTAGTAGTTTGTACAGN............................................................ 7   | 7; |
| ....TGAGGTAGTAGNTTGTACAGT............................................................ 6   | 6; |
| ...CTGAGGTAGTAGTTTGTACAG............................................................. 5   | 5; |
| ....TGNGGTAGTAGTTTGTACAGT............................................................ 5   | 5; |
| ....TGAGGTAGNAGTTTGTACAGT............................................................ 5   | 5; |
| ....TGAGGTAGTAGTTNGTACAGT............................................................ 5   | 5; |
| ....TGAGGTAGTAGTTTGNACAGT............................................................ 5   | 5; |
| ....TGAGGTAGTAGTT.................................................................... 4   | 4; |
| ....TGAGGTAGTAGTTT................................................................... 2   | 2; |
| ....TGAGGTAGTAG...................................................................... 1   | 1; |
| ------------------------------------------------------------------------------------ 96425 |
| ------------------------------------------------------------------------------------ 96425 |
| hsa-let-7g-5p(hsa-let-7g) AGGCTGAGGTAGTAGTTTGTACAGTTTGAGGGTCTATGATACCACCCGGTACAGGAGATAACTGTACAGGCCACTGCCTTGCCA .(((.((((((((.((((((((((((.....((((.((.((((....))))))..)))))))))))))))).))))))))))). (-40.50) \*\*\*\*TGAGGTAGTAGTTTGTACAGTT\*\*\*\*\*\*\*\*\*\*\*\*\*\*\*\*\*\*\*\*\*\*\*\*\*\*\*\*\*\*\*\*\*\*\*\*\*\*\*\*\*\*\*\*\*\*\*\*\*\*\*\*\*\*\*\*\*\* T  M |
| ....TGAGGTAGTAGTTTGTACAGTT...........................................................\* 79689   | 79689; |
| ....TGAGGTAGTAGTTTGTACAGT............................................................ 12608   | 12608; |
| ....TGAGGTAGTAGTTTGTACAGTTT.......................................................... 940   | 940; |
| ....TGAGGTAGTAGTTTGTACAG............................................................. 918   | 918; |
| ....TGAGGTANTAGTTTGTACAGTT........................................................... 250   | 250; |
| ....TGAGGTAGTNGTTTGTACAGTT........................................................... 247   | 247; |
| ....TGAGGTAGTAGTTTGTAC............................................................... 204   | 204; |
| ....TGAGGTAGTAGTTTNTACAGTT........................................................... 203   | 203; |
| ...CTGAGGTAGTAGTTTGTACAGTT........................................................... 161   | 161; |
| ....TGAGGTAGTAGTTTGTACA.............................................................. 137   | 137; |
| ....NGAGGTAGTAGTTTGTACAGTT........................................................... 122   | 122; |
| .....GAGGTAGTAGTTTGTACAGTT........................................................... 112   | 112; |
| ...CTGAGGTAGTAGTTTGTACAGT............................................................ 44   | 44; |
| ....TGAGGTANTAGTTTGTACAGT............................................................ 41   | 41; |
| ....TGAGGTAGTAGTTTGTA................................................................ 37   | 37; |
| ....TGAGGTAGTNGTTTGTACAGT............................................................ 36   | 36; |
| ......AGGTAGTAGTTTGTACAGTT........................................................... 36   | 36; |
| ....TGAGGTAGTANTTTGTACAGTT........................................................... 35   | 35; |
| ....TGAGGTAGTAGTTTGTNCAGTT........................................................... 34   | 34; |
| ....TGAGGTAGTAGTTTNTACAGT............................................................ 34   | 34; |
| ....TGAGGTAGTAGNTTGTACAGTT........................................................... 32   | 32; |
| ....TNAGGTAGTAGTTTGTACAGTT........................................................... 31   | 31; |
| ....TGAGGTAGTAGTTTGTANAGTT........................................................... 31   | 31; |
| ....TGAGGTAGTAGTTTGTACAGTN........................................................... 30   | 30; |
| ....TGAGNTAGTAGTTTGTACAGTT........................................................... 29   | 29; |
| ....TGAGGTAGTAGTTTGTACANTT........................................................... 27   | 27; |
| ....TGAGGTAGTAGTTTGTACAGNT........................................................... 25   | 25; |
| ....NGAGGTAGTAGTTTGTACAGT............................................................ 25   | 25; |
| ....TGAGGTAGNAGTTTGTACAGTT........................................................... 25   | 25; |
| ....TGAGGTAGTAGTTTGNACAGTT........................................................... 25   | 25; |
| ....TGAGGTNGTAGTTTGTACAGTT........................................................... 25   | 25; |
| ....TGAGGTAGTAGT..................................................................... 22   | 22; |
| ....TGAGGTAGTAGTNTGTACAGTT........................................................... 21   | 21; |
| ....TGANGTAGTAGTTTGTACAGTT........................................................... 21   | 21; |
| ....TGAGGTAGTAGTTTGTACNGTT........................................................... 20   | 20; |
| ....TGNGGTAGTAGTTTGTACAGTT........................................................... 20   | 20; |
| ....TGAGGTAGTAGTTNGTACAGTT........................................................... 20   | 20; |
| ....TGAGGNAGTAGTTTGTACAGTT........................................................... 18   | 18; |
| .....GAGGTAGTAGTTTGTACAGT............................................................ 17   | 17; |
| ....TGAGGTAGTAGTTTG.................................................................. 11   | 11; |
| ....TGAGGTAGTAGTTTGT................................................................. 9   | 9; |
| ....TGAGGTNGTAGTTTGTACAGT............................................................ 8   | 8; |
| ....TGAGGTAGTAGTTTGTACAGN............................................................ 7   | 7; |
| ....TGAGGTAGTAGNTTGTACAGT............................................................ 6   | 6; |
| ...CTGAGGTAGTAGTTTGTACAG............................................................. 5   | 5; |
| ....TGNGGTAGTAGTTTGTACAGT............................................................ 5   | 5; |
| ....TGAGGTAGNAGTTTGTACAGT............................................................ 5   | 5; |
| ....TGAGGTAGTAGTTNGTACAGT............................................................ 5   | 5; |
| ....TGAGGTAGTAGTTTGNACAGT............................................................ 5   | 5; |
| ....TGAGGTAGTAGTT.................................................................... 4   | 4; |
| ....TGAGGTAGTAGTTT................................................................... 2   | 2; |
| ....TGAGGTAGTAG...................................................................... 1   | 1; |
| ------------------------------------------------------------------------------------ 96425 |
| ....TGAGGTAGTAGTTTGTACAGTTa.......................................................... 2308   | 2308; |
| ....TGAGGTAGTAGTT-GTACAGTT........................................................... 1006   | 1006; |
| ....TGAGGTAGTAGTTTGTACAGTa........................................................... 162   | 162; |
| ....TGAGGTAGTAGaTTGTACAGTT........................................................... 121   | 121; |
| ....TGAGGTAGTAGTT-GTACAGT............................................................ 112   | 112; |
| ....TGAGGTAGTAGTTTGTcCAGTT........................................................... 98   | 98; |
| ....TGAGGTAGTAGTTTGTAtAGTT........................................................... 81   | 81; |
| ....TGAGGcAGTAGTTTGTACAGTT........................................................... 79   | 79; |
| ....TaAGGTAGTAGTTTGTACAGTT........................................................... 78   | 78; |
| ....TGAGGTAGTAGTTTGTAaAGTT........................................................... 70   | 70; |
| ....TGAGGTAGTAGTTTGTACcGTT........................................................... 61   | 61; |
| ....TGgGGTAGTAGTTTGTACAGTT........................................................... 59   | 59; |
| ....cGAGGTAGTAGTTTGTACAGTT........................................................... 59   | 59; |
| ....TGAGGaAGTAGTTTGTACAGTT........................................................... 58   | 58; |
| ....TGAGGTgGTAGTTTGTACAGTT........................................................... 57   | 57; |
| ....TGAGGTAGTAGTTTGcACAGTT........................................................... 56   | 56; |
| ....TGAGGTAGTgGTTTGTACAGTT........................................................... 50   | 50; |
| ....TGAGGTAGcAGTTTGTACAGTT........................................................... 50   | 50; |
| ....TGAGGTAtTAGTTTGTACAGTT........................................................... 50   | 50; |
| ....TGAGGTAGTAGTTTGTACAGcT........................................................... 48   | 48; |
| ....TGAGGTAGTAGgTTGTACAGTT........................................................... 47   | 47; |
| ....TGAGGTAGTAGTTTGTtCAGTT........................................................... 45   | 45; |
| ....TGAGGTAGTAGTTTGTACAGgT........................................................... 44   | 44; |
| ....TGAGGTAGTAGTTTGTACAGTTg.......................................................... 43   | 43; |
| ....TGAGGTAGaAGTTTGTACAGTT........................................................... 42   | 42; |
| ....TGAGGTAGTAGTTTGTACAGaT........................................................... 38   | 38; |
| ....TGAG-TAGTAGTTTGTACAGTT........................................................... 38   | 38; |
| ....TGAGGTAGTAGTTTGTACgGTT........................................................... 37   | 37; |
| ....TGAGGTAGTAGcTTGTACAGTT........................................................... 35   | 35; |
| ....TGAGGTAGTAGTTcGTACAGTT........................................................... 34   | 34; |
| ....TGAGGTAGTAGTTTaTACAGTT........................................................... 31   | 31; |
| ....TGAGGTAGgAGTTTGTACAGTT........................................................... 31   | 31; |
| ....TGAGGTAGTAGTTTGTgCAGTT........................................................... 31   | 31; |
| ....TGAGGTAGTAGTTTGTACAGTc........................................................... 29   | 29; |
| ....TGAGGTAGTAGTTTGaACAGTT........................................................... 28   | 28; |
| ....aGAGGTAGTAGTTTGTACAGTT........................................................... 26   | 26; |
| ....TGAGGTAGTAGTT-GTACAGTTa.......................................................... 26   | 26; |
| ....TGtGGTAGTAGTTTGTACAGTT........................................................... 25   | 25; |
| ....TGAGGTAaTAGTTTGTACAGTT........................................................... 24   | 24; |
| ....TGAGGTAcTAGTTTGTACAGTT........................................................... 24   | 24; |
| ....TGAGGTAGTAGTcTGTACAGTT........................................................... 24   | 24; |
| ....TGAGGgAGTAGTTTGTACAGTT........................................................... 24   | 24; |
| ....TGAGGTAGTAGTTTGTACAGTaa.......................................................... 22   | 22; |
| ....TGAGGTAGTAGTTTGTAgAGTT........................................................... 22   | 22; |
| ....TGAaGTAGTAGTTTGTACAGTT........................................................... 22   | 22; |
| ....TGAGaTAGTAGTTTGTACAGTT........................................................... 21   | 21; |
| ....TGcGGTAGTAGTTTGTACAGTT........................................................... 21   | 21; |
| ....TGAGGTAGTAGTTT--ACAGTT........................................................... 20   | 20; |
| ....TGAGGTAGT--TTTGTACAGTT........................................................... 19   | 19; |
| ....TGAGGTAGTAGaTTGTACAGT............................................................ 19   | 19; |
| ....TtAGGTAGTAGTTTGTACAGTT........................................................... 19   | 19; |
| ....TGAGGTAGTAGTT-GTACAGTTT.......................................................... 18   | 18; |
| ....TGAGGTtGTAGTTTGTACAGTT........................................................... 18   | 18; |
| ....TGAGGTAGTAGTTTGTACtGTT........................................................... 17   | 17; |
| ....TGAGGTAGTAGTTTGTACAGa............................................................ 17   | 17; |
| ....TGAGGTAGTAGTTaGTACAGTT........................................................... 17   | 17; |
| ....TGAGGTAGTAGTTTGgACAGTT........................................................... 16   | 16; |
| ....TGAtGTAGTAGTTTGTACAGTT........................................................... 16   | 16; |
| ....TGAGGTAGcAGTTTGTACAGT............................................................ 15   | 15; |
| ....TGAGGTAGTAGTTTGTAaAGT............................................................ 15   | 15; |
| ....TGAGGTAGTcGTTTGTACAGTT........................................................... 15   | 15; |
| ....TGAGGTAGTtGTTTGTACAGTT........................................................... 15   | 15; |
| ....TGAGGTAGTAGTTTcTACAGTT........................................................... 15   | 15; |
| ....TGAGGTgGTAGTTTGTACAGT............................................................ 14   | 14; |
| ....TGAGGTAGTAGTTT-TACAGTT........................................................... 14   | 14; |
| ....TGAGGTAGTAGTTTGTACAtTT........................................................... 14   | 14; |
| ....TGAGGTAGTAGTTTGTACAGTaT.......................................................... 14   | 14; |
| ....TGAGGTAGTAGTaTGTACAGTT........................................................... 13   | 13; |
| ....TGAGGTAtTAGTTTGTACAGT............................................................ 13   | 13; |
| ....TGAGGcAGTAGTTTGTACAGT............................................................ 13   | 13; |
| ....TGAGGTAGTAGTTTtTACAGTT........................................................... 12   | 12; |
| ....TGAGGTAGTAaTTTGTACAGTT........................................................... 12   | 12; |
| ....TaAGGTAGTAGTTTGTACAGT............................................................ 12   | 12; |
| ....cGAGGTAGTAGTTTGTACAGT............................................................ 12   | 12; |
| ....TGAGGTAGTAGTTTGTAtAGT............................................................ 12   | 12; |
| ....TGAGtTAGTAGTTTGTACAGTT........................................................... 12   | 12; |
| ....TGAGGTcGTAGTTTGTACAGTT........................................................... 11   | 11; |
| ....TGAGGTAGTAGTTTGTtCAGT............................................................ 11   | 11; |
| ....TGAGGTAGTAGTTTGTcCAGT............................................................ 11   | 11; |
| ....TGAcGTAGTAGTTTGTACAGTT........................................................... 11   | 11; |
| ....TcAGGTAGTAGTTTGTACAGTT........................................................... 10   | 10; |
| ....TGAGGTAGgAGTTTGTACAGT............................................................ 10   | 10; |
| ....TGAGGTAGTAGTTTGTgCAGT............................................................ 9   | 9; |
| ....TGAGGTAGTAGTTTGTACcGT............................................................ 9   | 9; |
| ....TGAGGTAGTAGTTTaTACAGT............................................................ 9   | 9; |
| ....TGAGcTAGTAGTTTGTACAGTT........................................................... 9   | 9; |
| ....TGAGGTAGTAcTTTGTACAGTT........................................................... 9   | 9; |
| ....TGAGGTcGTAGTTTGTACAGT............................................................ 8   | 8; |
| ....TGtGGTAGTAGTTTGTACAGT............................................................ 8   | 8; |
| ....TGgGGTAGTAGTTTGTACAGT............................................................ 8   | 8; |
| ....TGAGGTAGTAGTTTGTACAGc............................................................ 8   | 8; |
| ....TGcGGTAGTAGTTTGTACAGT............................................................ 8   | 8; |
| ....TGAGGTAGTAGTTTGTACgGT............................................................ 8   | 8; |
| ....TGAGGTAGTgGTTTGTACAGT............................................................ 7   | 7; |
| .....GAGGTAGTAGTTTGTACAGTTa.......................................................... 7   | 7; |
| ....TGAGGTAGgAGTTTGTACAGgT........................................................... 7   | 7; |
| ....TGAGGTAGTAGTTTGTACAcTT........................................................... 7   | 7; |
| ....TGAGGTAaTAGTTTGTACAGT............................................................ 7   | 7; |
| ....TGAGGTAGTAGTTTGTACAGTTc.......................................................... 7   | 7; |
| ....gGAGGTAGTAGTTTGTACAGTT........................................................... 7   | 7; |
| ....NGAGGTAGTAGTTTGTACAGTTa.......................................................... 7   | 7; |
[truncated: 1,744,573 more chars]
