# Supplementary material for: The Repertoire and Features of Human Platelet microRNAs
Source: PLoS One. 2012 Dec 4;7(12):e50746. doi: 10.1371/journal.pone.0050746 (PMC3514217; doi:10.1371/journal.pone.0050746)
Supplement: Database S1 — (ZIP) [file pone.0050746.s011.zip › Supporting Platelet microRNA sequence database S1/s7_sequence_s7run61.mir.html]

s7run61 Exact Matches (All miRNAs (both expressed and non-expressed) are listed in this table) 

| mature miRNA | Exact Match to miR (+4) | Exact Match to miR |
| --- | --- | --- |
| hsa-let-7a-2-3p | 0 | 0 |
| hsa-let-7a-3p | 0 | 0 |
| hsa-let-7a-5p | 497978 | 372552 |
| hsa-let-7b-3p | 0 | 0 |
| hsa-let-7b-5p | 47287 | 27832 |
| hsa-let-7c | 4436 | 2058 |
| hsa-let-7d-3p | 206 | 159 |
| hsa-let-7d-5p | 96634 | 79335 |
| hsa-let-7e-3p | 0 | 0 |
| hsa-let-7e-5p | 30478 | 21788 |
| hsa-let-7f-1-3p | 0 | 0 |
| hsa-let-7f-2-3p | 0 | 0 |
| hsa-let-7f-5p | 1178191 | 967100 |
| hsa-let-7g-3p | 0 | 0 |
| hsa-let-7g-5p | 94957 | 79689 |
| hsa-let-7i-3p | 9 | 9 |
| hsa-let-7i-5p | 52507 | 34478 |
| hsa-mir-1 | 25745 | 23782 |
| hsa-mir-100-3p | 0 | 0 |
| hsa-mir-100-5p | 39 | 21 |
| hsa-mir-101-3p | 30132 | 6108 |
| hsa-mir-101-5p | 0 | 0 |
| hsa-mir-103a-2-5p | 0 | 0 |
| hsa-mir-103a-3p | 310373 | 191592 |
| hsa-mir-106a-3p | 0 | 0 |
| hsa-mir-106a-5p | 6 | 0 |
| hsa-mir-106b-3p | 452 | 245 |
| hsa-mir-106b-5p | 1296 | 691 |
| hsa-mir-107 | 50610 | 15387 |
| hsa-mir-10a-3p | 9 | 0 |
| hsa-mir-10a-5p | 620 | 35 |
| hsa-mir-1178 | 0 | 0 |
| hsa-mir-1185-1-3p | 352 | 247 |
| hsa-mir-1185-2-3p | 122 | 76 |
| hsa-mir-1185-5p | 100 | 16 |
| hsa-mir-1197 | 37 | 37 |
| hsa-mir-122-3p | 0 | 0 |
| hsa-mir-122-5p | 13 | 13 |
| hsa-mir-1250 | 48 | 24 |
| hsa-mir-1255a | 56 | 19 |
| hsa-mir-1255b-2-3p | 0 | 0 |
| hsa-mir-1255b-5p | 12 | 12 |
| hsa-mir-1256 | 61 | 54 |
| hsa-mir-125a-3p | 6 | 0 |
| hsa-mir-125a-5p | 390 | 15 |
| hsa-mir-125b-1-3p | 0 | 0 |
| hsa-mir-125b-2-3p | 0 | 0 |
| hsa-mir-125b-5p | 66 | 42 |
| hsa-mir-126-3p | 4678 | 910 |
| hsa-mir-126-5p | 7174 | 6494 |
| hsa-mir-1260b | 34 | 12 |
| hsa-mir-127-3p | 4443 | 4047 |
| hsa-mir-127-5p | 101 | 36 |
| hsa-mir-1271-3p | 0 | 0 |
| hsa-mir-1271-5p | 85 | 66 |
| hsa-mir-1273c | 6 | 0 |
| hsa-mir-1277-3p | 1218 | 1071 |
| hsa-mir-1277-5p | 91 | 0 |
| hsa-mir-1278 | 125 | 88 |
| hsa-mir-128 | 9092 | 6230 |
| hsa-mir-1284 | 16 | 9 |
| hsa-mir-1285-3p | 69 | 42 |
| hsa-mir-1285-5p | 0 | 0 |
| hsa-mir-1287 | 240 | 125 |
| hsa-mir-1291 | 5 | 0 |
| hsa-mir-1294 | 45 | 34 |
| hsa-mir-1297 | 3 | 0 |
| hsa-mir-1299 | 14 | 14 |
| hsa-mir-1301 | 327 | 32 |
| hsa-mir-1304-3p | 0 | 0 |
| hsa-mir-1304-5p | 17 | 0 |
| hsa-mir-1306-3p | 54 | 0 |
| hsa-mir-1306-5p | 28 | 19 |
| hsa-mir-1307-3p | 3567 | 770 |
| hsa-mir-1307-5p | 3832 | 1952 |
| hsa-mir-130a-3p | 4567 | 4234 |
| hsa-mir-130a-5p | 0 | 0 |
| hsa-mir-130b-3p | 809 | 751 |
| hsa-mir-130b-5p | 65 | 12 |
| hsa-mir-132-3p | 17 | 17 |
| hsa-mir-132-5p | 8 | 8 |
| hsa-mir-1322 | 0 | 0 |
| hsa-mir-133a | 10 | 0 |
| hsa-mir-134 | 1025 | 479 |
| hsa-mir-135a-3p | 6 | 0 |
| hsa-mir-135a-5p | 0 | 0 |
| hsa-mir-136-3p | 65 | 50 |
| hsa-mir-136-5p | 97 | 73 |
| hsa-mir-139-3p | 486 | 16 |
| hsa-mir-139-5p | 320 | 54 |
| hsa-mir-140-3p | 56986 | 2092 |
| hsa-mir-140-5p | 12 | 7 |
| hsa-mir-142-3p | 615 | 111 |
| hsa-mir-142-5p | 15037 | 1102 |
| hsa-mir-143-3p | 2756 | 2068 |
| hsa-mir-143-5p | 133 | 29 |
| hsa-mir-144-3p | 273 | 29 |
| hsa-mir-144-5p | 202 | 36 |
| hsa-mir-1468 | 5 | 0 |
| hsa-mir-146a-3p | 0 | 0 |
| hsa-mir-146a-5p | 2111 | 1108 |
| hsa-mir-146b-3p | 8 | 0 |
| hsa-mir-146b-5p | 485 | 280 |
| hsa-mir-148a-3p | 1453 | 1176 |
| hsa-mir-148a-5p | 17 | 0 |
| hsa-mir-148b-3p | 3643 | 2821 |
| hsa-mir-148b-5p | 30 | 0 |
| hsa-mir-151a-3p | 6058 | 2167 |
| hsa-mir-151a-5p | 6670 | 3327 |
| hsa-mir-151b | 6706 | 16 |
| hsa-mir-152 | 9406 | 6618 |
| hsa-mir-154-3p | 109 | 84 |
| hsa-mir-154-5p | 59 | 59 |
| hsa-mir-155-3p | 0 | 0 |
| hsa-mir-155-5p | 130 | 16 |
| hsa-mir-15a-3p | 8 | 8 |
| hsa-mir-15a-5p | 830 | 134 |
| hsa-mir-15b-3p | 17 | 6 |
| hsa-mir-15b-5p | 1581 | 515 |
| hsa-mir-16-1-3p | 21 | 21 |
| hsa-mir-16-2-3p | 11 | 0 |
| hsa-mir-16-5p | 5824 | 5052 |
| hsa-mir-17-3p | 415 | 213 |
| hsa-mir-17-5p | 711 | 346 |
| hsa-mir-181a-2-3p | 319 | 138 |
| hsa-mir-181a-3p | 636 | 201 |
| hsa-mir-181a-5p | 8010 | 1024 |
| hsa-mir-181b-3p | 5 | 5 |
| hsa-mir-181b-5p | 674 | 30 |
| hsa-mir-181c-3p | 407 | 6 |
| hsa-mir-181c-5p | 36 | 5 |
| hsa-mir-181d | 292 | 76 |
| hsa-mir-182-3p | 0 | 0 |
| hsa-mir-182-5p | 7 | 7 |
| hsa-mir-185-3p | 871 | 0 |
| hsa-mir-185-5p | 120587 | 108162 |
| hsa-mir-186-3p | 0 | 0 |
| hsa-mir-186-5p | 4467 | 1413 |
| hsa-mir-18a-3p | 0 | 0 |
| hsa-mir-18a-5p | 168 | 12 |
| hsa-mir-1908 | 351 | 195 |
| hsa-mir-191-3p | 46 | 0 |
| hsa-mir-191-5p | 24733 | 12079 |
| hsa-mir-192-3p | 0 | 0 |
| hsa-mir-192-5p | 5229 | 2910 |
| hsa-mir-193a-3p | 0 | 0 |
| hsa-mir-193a-5p | 5 | 5 |
| hsa-mir-194-3p | 0 | 0 |
| hsa-mir-194-5p | 25 | 8 |
| hsa-mir-195-3p | 0 | 0 |
| hsa-mir-195-5p | 16 | 0 |
| hsa-mir-196b-3p | 0 | 0 |
| hsa-mir-196b-5p | 132 | 69 |
| hsa-mir-197-3p | 99 | 93 |
| hsa-mir-197-5p | 0 | 0 |
| hsa-mir-199a-3p | 499059 | 271365 |
| hsa-mir-199a-5p | 200 | 84 |
| hsa-mir-199b-3p | 499059 | 271365 |
| hsa-mir-199b-5p | 0 | 0 |
| hsa-mir-19a-3p | 5 | 5 |
| hsa-mir-19a-5p | 0 | 0 |
| hsa-mir-19b-1-5p | 0 | 0 |
| hsa-mir-19b-2-5p | 0 | 0 |
| hsa-mir-19b-3p | 110 | 32 |
| hsa-mir-200a-3p | 6 | 0 |
| hsa-mir-200a-5p | 0 | 0 |
| hsa-mir-200b-3p | 11 | 6 |
| hsa-mir-200b-5p | 0 | 0 |
| hsa-mir-200c-3p | 18 | 0 |
| hsa-mir-200c-5p | 0 | 0 |
| hsa-mir-203 | 5 | 5 |
| hsa-mir-204-3p | 11 | 11 |
| hsa-mir-204-5p | 0 | 0 |
| hsa-mir-20a-3p | 12 | 0 |
| hsa-mir-20a-5p | 252 | 82 |
| hsa-mir-20b-3p | 6 | 0 |
| hsa-mir-20b-5p | 13 | 13 |
| hsa-mir-21-3p | 834 | 478 |
| hsa-mir-21-5p | 101142 | 33230 |
| hsa-mir-210 | 82 | 76 |
| hsa-mir-2110 | 84 | 21 |
| hsa-mir-215 | 24 | 16 |
| hsa-mir-22-3p | 2547 | 1851 |
| hsa-mir-22-5p | 297 | 174 |
| hsa-mir-221-3p | 53676 | 25441 |
| hsa-mir-221-5p | 266 | 120 |
| hsa-mir-222-3p | 3128 | 331 |
| hsa-mir-222-5p | 0 | 0 |
| hsa-mir-223-3p | 31099 | 13693 |
| hsa-mir-223-5p | 0 | 0 |
| hsa-mir-224-3p | 8 | 0 |
| hsa-mir-224-5p | 832 | 35 |
| hsa-mir-2355-3p | 169 | 85 |
| hsa-mir-2355-5p | 386 | 61 |
| hsa-mir-23a-3p | 10037 | 1868 |
| hsa-mir-23a-5p | 12 | 6 |
| hsa-mir-23b-3p | 1752 | 211 |
| hsa-mir-23b-5p | 59 | 35 |
| hsa-mir-24-1-5p | 0 | 0 |
| hsa-mir-24-2-5p | 37 | 0 |
| hsa-mir-24-3p | 18358 | 14688 |
| hsa-mir-25-3p | 59251 | 48686 |
| hsa-mir-25-5p | 248 | 18 |
| hsa-mir-26a-1-3p | 13 | 13 |
| hsa-mir-26a-2-3p | 0 | 0 |
| hsa-mir-26a-5p | 30972 | 28166 |
| hsa-mir-26b-3p | 0 | 0 |
| hsa-mir-26b-5p | 49328 | 3340 |
| hsa-mir-27a-3p | 9537 | 1359 |
| hsa-mir-27a-5p | 0 | 0 |
| hsa-mir-27b-3p | 7031 | 4874 |
| hsa-mir-27b-5p | 33 | 10 |
| hsa-mir-28-3p | 528 | 438 |
| hsa-mir-28-5p | 1015 | 312 |
| hsa-mir-2964a-3p | 0 | 0 |
| hsa-mir-2964a-5p | 17 | 17 |
| hsa-mir-299-3p | 33 | 18 |
| hsa-mir-299-5p | 7 | 7 |
| hsa-mir-29a-3p | 12623 | 10760 |
| hsa-mir-29a-5p | 0 | 0 |
| hsa-mir-29b-1-5p | 0 | 0 |
| hsa-mir-29b-2-5p | 0 | 0 |
| hsa-mir-29b-3p | 134 | 66 |
| hsa-mir-29c-3p | 2285 | 2191 |
| hsa-mir-29c-5p | 13 | 0 |
| hsa-mir-301a-3p | 14 | 0 |
| hsa-mir-301a-5p | 109 | 58 |
| hsa-mir-30a-3p | 97 | 46 |
| hsa-mir-30a-5p | 396 | 85 |
| hsa-mir-30b-3p | 104 | 11 |
| hsa-mir-30b-5p | 228 | 208 |
| hsa-mir-30c-1-3p | 120 | 15 |
| hsa-mir-30c-2-3p | 5 | 0 |
| hsa-mir-30c-5p | 138 | 36 |
| hsa-mir-30d-3p | 0 | 0 |
| hsa-mir-30d-5p | 12794 | 1409 |
| hsa-mir-30e-3p | 2465 | 1567 |
| hsa-mir-30e-5p | 1244 | 50 |
| hsa-mir-31-3p | 0 | 0 |
| hsa-mir-31-5p | 7 | 0 |
| hsa-mir-3120-3p | 149 | 108 |
| hsa-mir-3120-5p | 0 | 0 |
| hsa-mir-3121-3p | 7 | 7 |
| hsa-mir-3121-5p | 0 | 0 |
| hsa-mir-3123 | 43 | 0 |
| hsa-mir-3124-3p | 0 | 0 |
| hsa-mir-3124-5p | 21 | 16 |
| hsa-mir-3130-3p | 26 | 26 |
| hsa-mir-3130-5p | 0 | 0 |
| hsa-mir-3136-3p | 0 | 0 |
| hsa-mir-3136-5p | 16 | 0 |
| hsa-mir-3138 | 63 | 0 |
| hsa-mir-3140-3p | 5 | 0 |
| hsa-mir-3140-5p | 0 | 0 |
| hsa-mir-3143 | 5 | 0 |
| hsa-mir-3161 | 10 | 0 |
| hsa-mir-3168 | 178 | 0 |
| hsa-mir-3174 | 17 | 0 |
| hsa-mir-3175 | 14 | 14 |
| hsa-mir-3177-3p | 20 | 20 |
| hsa-mir-3177-5p | 0 | 0 |
| hsa-mir-3182 | 30 | 0 |
| hsa-mir-3183 | 17 | 17 |
| hsa-mir-3190-3p | 12 | 0 |
| hsa-mir-3190-5p | 0 | 0 |
| hsa-mir-3191-3p | 8 | 0 |
| hsa-mir-3191-5p | 0 | 0 |
| hsa-mir-3196 | 19 | 0 |
| hsa-mir-3198 | 16 | 16 |
| hsa-mir-32-3p | 0 | 0 |
| hsa-mir-32-5p | 56 | 0 |
| hsa-mir-320a | 40118 | 31152 |
| hsa-mir-320b | 310 | 66 |
| hsa-mir-320c | 70 | 42 |
| hsa-mir-320d | 22 | 6 |
| hsa-mir-323a-3p | 1334 | 888 |
| hsa-mir-323a-5p | 48 | 7 |
| hsa-mir-323b-3p | 1561 | 14 |
| hsa-mir-323b-5p | 0 | 0 |
| hsa-mir-324-3p | 14 | 0 |
| hsa-mir-324-5p | 59 | 42 |
| hsa-mir-326 | 9 | 9 |
| hsa-mir-328 | 154 | 136 |
| hsa-mir-329 | 76 | 24 |
| hsa-mir-330-3p | 4087 | 1701 |
| hsa-mir-330-5p | 0 | 0 |
| hsa-mir-331-3p | 267 | 166 |
| hsa-mir-331-5p | 28 | 0 |
| hsa-mir-335-3p | 30 | 22 |
| hsa-mir-335-5p | 1736 | 295 |
| hsa-mir-337-3p | 5 | 0 |
| hsa-mir-337-5p | 0 | 0 |
| hsa-mir-338-3p | 32 | 0 |
| hsa-mir-338-5p | 16 | 0 |
| hsa-mir-339-3p | 1044 | 397 |
| hsa-mir-339-5p | 912 | 101 |
| hsa-mir-33a-3p | 14 | 0 |
| hsa-mir-33a-5p | 3907 | 2614 |
| hsa-mir-33b-3p | 0 | 0 |
| hsa-mir-33b-5p | 142 | 90 |
| hsa-mir-340-3p | 0 | 0 |
| hsa-mir-340-5p | 23788 | 14653 |
| hsa-mir-342-3p | 155 | 61 |
| hsa-mir-342-5p | 31 | 19 |
| hsa-mir-345-3p | 0 | 0 |
| hsa-mir-345-5p | 21 | 9 |
| hsa-mir-3605-3p | 0 | 0 |
| hsa-mir-3605-5p | 7 | 0 |
| hsa-mir-361-3p | 0 | 0 |
| hsa-mir-361-5p | 145 | 116 |
| hsa-mir-3615 | 148 | 32 |
| hsa-mir-363-3p | 529 | 167 |
| hsa-mir-363-5p | 6 | 6 |
| hsa-mir-3656 | 52 | 0 |
| hsa-mir-3676-3p | 0 | 0 |
| hsa-mir-3676-5p | 28 | 12 |
| hsa-mir-3677-3p | 29 | 18 |
| hsa-mir-3677-5p | 0 | 0 |
| hsa-mir-369-3p | 1527 | 1404 |
| hsa-mir-369-5p | 246 | 23 |
| hsa-mir-370 | 18 | 13 |
| hsa-mir-374a-3p | 477 | 217 |
| hsa-mir-374a-5p | 1304 | 624 |
| hsa-mir-374b-3p | 60 | 19 |
| hsa-mir-374b-5p | 548 | 433 |
| hsa-mir-375 | 5 | 5 |
| hsa-mir-376a-3p | 30 | 30 |
| hsa-mir-376a-5p | 0 | 0 |
| hsa-mir-376b | 72 | 10 |
| hsa-mir-376c | 270 | 252 |
| hsa-mir-377-3p | 10 | 10 |
| hsa-mir-377-5p | 35 | 35 |
| hsa-mir-378a-3p | 1642 | 932 |
| hsa-mir-378a-5p | 0 | 0 |
| hsa-mir-378b | 1 | 0 |
| hsa-mir-378c | 259 | 0 |
| hsa-mir-378d | 54 | 6 |
| hsa-mir-378e | 1 | 0 |
| hsa-mir-378f | 1 | 0 |
| hsa-mir-379-3p | 14 | 0 |
| hsa-mir-379-5p | 968 | 804 |
| hsa-mir-380-3p | 8 | 0 |
| hsa-mir-380-5p | 0 | 0 |
| hsa-mir-381 | 78 | 58 |
| hsa-mir-382-3p | 203 | 126 |
| hsa-mir-382-5p | 1772 | 1196 |
| hsa-mir-3912 | 7 | 7 |
| hsa-mir-3920 | 5 | 5 |
| hsa-mir-3928 | 76 | 60 |
| hsa-mir-409-3p | 621 | 277 |
| hsa-mir-409-5p | 233 | 161 |
| hsa-mir-410 | 436 | 409 |
| hsa-mir-411-3p | 44 | 0 |
| hsa-mir-411-5p | 664 | 311 |
| hsa-mir-412 | 0 | 0 |
| hsa-mir-421 | 146 | 24 |
| hsa-mir-423-3p | 4279 | 2799 |
| hsa-mir-423-5p | 38466 | 16882 |
| hsa-mir-424-3p | 392 | 257 |
| hsa-mir-424-5p | 60 | 8 |
| hsa-mir-425-3p | 1220 | 66 |
| hsa-mir-425-5p | 526 | 293 |
| hsa-mir-4286 | 1002 | 0 |
| hsa-mir-4306 | 109 | 0 |
| hsa-mir-431-3p | 22 | 7 |
| hsa-mir-431-5p | 54 | 14 |
| hsa-mir-432-3p | 0 | 0 |
| hsa-mir-432-5p | 1984 | 261 |
| hsa-mir-433 | 1821 | 1505 |
| hsa-mir-4433-3p | 41 | 31 |
| hsa-mir-4433-5p | 0 | 0 |
| hsa-mir-4435 | 74 | 68 |
| hsa-mir-4436b-3p | 10 | 0 |
| hsa-mir-4436b-5p | 0 | 0 |
| hsa-mir-4443 | 71 | 0 |
| hsa-mir-4446-3p | 678 | 503 |
| hsa-mir-4446-5p | 0 | 0 |
| hsa-mir-4448 | 21 | 0 |
| hsa-mir-4454 | 38 | 0 |
| hsa-mir-4469 | 14 | 14 |
| hsa-mir-4470 | 19 | 19 |
| hsa-mir-4488 | 21 | 0 |
| hsa-mir-4492 | 53 | 0 |
| hsa-mir-4500 | 29 | 0 |
| hsa-mir-4508 | 1699 | 6 |
| hsa-mir-450a-3p | 5 | 0 |
| hsa-mir-450a-5p | 24 | 10 |
| hsa-mir-450b-3p | 0 | 0 |
| hsa-mir-450b-5p | 13 | 0 |
| hsa-mir-4516 | 51 | 0 |
| hsa-mir-451a | 436 | 232 |
| hsa-mir-452-3p | 0 | 0 |
| hsa-mir-452-5p | 229 | 40 |
| hsa-mir-4523 | 21 | 21 |
| hsa-mir-454-3p | 0 | 0 |
| hsa-mir-454-5p | 7 | 7 |
| hsa-mir-4659b-3p | 18 | 0 |
| hsa-mir-4659b-5p | 0 | 0 |
| hsa-mir-4665-3p | 0 | 0 |
| hsa-mir-4665-5p | 12 | 12 |
| hsa-mir-4667-3p | 0 | 0 |
| hsa-mir-4667-5p | 5 | 5 |
| hsa-mir-4707-3p | 8 | 8 |
| hsa-mir-4707-5p | 0 | 0 |
| hsa-mir-4708-3p | 5 | 5 |
| hsa-mir-4708-5p | 0 | 0 |
| hsa-mir-4734 | 11 | 0 |
| hsa-mir-4765 | 5 | 5 |
| hsa-mir-4791 | 5 | 0 |
| hsa-mir-484 | 343 | 224 |
| hsa-mir-485-3p | 1838 | 1702 |
| hsa-mir-485-5p | 754 | 367 |
| hsa-mir-486-3p | 2482 | 1703 |
| hsa-mir-486-5p | 3165 | 1486 |
| hsa-mir-487a | 28 | 22 |
| hsa-mir-487b | 511 | 434 |
| hsa-mir-490-3p | 10 | 5 |
| hsa-mir-490-5p | 0 | 0 |
| hsa-mir-491-3p | 0 | 0 |
| hsa-mir-491-5p | 5 | 5 |
| hsa-mir-493-3p | 113 | 94 |
| hsa-mir-493-5p | 193 | 113 |
| hsa-mir-494 | 280 | 5 |
| hsa-mir-495 | 2804 | 1595 |
| hsa-mir-496 | 163 | 73 |
| hsa-mir-497-3p | 0 | 0 |
| hsa-mir-497-5p | 57 | 36 |
| hsa-mir-499a-3p | 0 | 0 |
| hsa-mir-499a-5p | 135 | 76 |
| hsa-mir-499b-3p | 0 | 0 |
| hsa-mir-499b-5p | 5 | 5 |
| hsa-mir-500a-3p | 69 | 0 |
| hsa-mir-500a-5p | 0 | 0 |
| hsa-mir-500b | 0 | 0 |
| hsa-mir-501-3p | 21 | 13 |
| hsa-mir-501-5p | 0 | 0 |
| hsa-mir-5010-3p | 0 | 0 |
| hsa-mir-5010-5p | 316 | 160 |
| hsa-mir-502-3p | 57 | 38 |
| hsa-mir-502-5p | 0 | 0 |
| hsa-mir-503 | 607 | 335 |
| hsa-mir-504 | 14 | 14 |
| hsa-mir-505-3p | 0 | 0 |
| hsa-mir-505-5p | 66 | 30 |
| hsa-mir-5187-3p | 0 | 0 |
| hsa-mir-5187-5p | 80 | 64 |
| hsa-mir-5189 | 117 | 0 |
| hsa-mir-532-3p | 15 | 15 |
| hsa-mir-532-5p | 635 | 600 |
| hsa-mir-539-3p | 329 | 167 |
| hsa-mir-539-5p | 6 | 6 |
| hsa-mir-542-3p | 89 | 37 |
| hsa-mir-542-5p | 0 | 0 |
| hsa-mir-543 | 1752 | 988 |
| hsa-mir-548ad | 0 | 0 |
| hsa-mir-548ae | 0 | 0 |
| hsa-mir-548ah-3p | 6 | 0 |
| hsa-mir-548am-3p | 0 | 0 |
| hsa-mir-548am-5p | 60 | 0 |
| hsa-mir-548ap-5p | 965 | 0 |
| hsa-mir-548au-3p | 0 | 0 |
| hsa-mir-548au-5p | 60 | 3 |
| hsa-mir-548av-3p | 10 | 0 |
| hsa-mir-548av-5p | 107 | 0 |
| hsa-mir-548ax | 0 | 0 |
| hsa-mir-548c-3p | 0 | 0 |
| hsa-mir-548c-5p | 60 | 0 |
| hsa-mir-548d-3p | 0 | 0 |
| hsa-mir-548d-5p | 2 | 0 |
| hsa-mir-548e | 94 | 52 |
| hsa-mir-548j | 965 | 793 |
| hsa-mir-548k | 107 | 100 |
| hsa-mir-548l | 7 | 0 |
| hsa-mir-548n | 8 | 8 |
| hsa-mir-548o-3p | 10 | 10 |
| hsa-mir-548o-5p | 60 | 0 |
| hsa-mir-548p | 6 | 6 |
| hsa-mir-550a-3-5p | 0 | 0 |
| hsa-mir-550a-3p | 6 | 6 |
| hsa-mir-550a-5p | 0 | 0 |
| hsa-mir-551b-3p | 11 | 0 |
| hsa-mir-551b-5p | 0 | 0 |
| hsa-mir-552 | 10 | 10 |
| hsa-mir-556-3p | 9 | 0 |
| hsa-mir-556-5p | 0 | 0 |
| hsa-mir-5586-3p | 6 | 0 |
| hsa-mir-574-3p | 180 | 146 |
| hsa-mir-574-5p | 0 | 0 |
| hsa-mir-576-3p | 27 | 14 |
| hsa-mir-576-5p | 0 | 0 |
| hsa-mir-584-3p | 0 | 0 |
| hsa-mir-584-5p | 22784 | 135 |
| hsa-mir-589-3p | 0 | 0 |
| hsa-mir-589-5p | 187 | 19 |
| hsa-mir-590-3p | 27 | 16 |
| hsa-mir-590-5p | 37 | 29 |
| hsa-mir-598 | 2464 | 1919 |
| hsa-mir-605 | 0 | 0 |
| hsa-mir-624-3p | 0 | 0 |
| hsa-mir-624-5p | 11 | 11 |
| hsa-mir-625-3p | 29 | 13 |
| hsa-mir-625-5p | 486 | 40 |
| hsa-mir-627 | 0 | 0 |
| hsa-mir-628-3p | 0 | 0 |
| hsa-mir-628-5p | 32 | 20 |
| hsa-mir-629-3p | 0 | 0 |
| hsa-mir-629-5p | 263 | 128 |
| hsa-mir-636 | 0 | 0 |
| hsa-mir-639 | 0 | 0 |
| hsa-mir-641 | 9 | 0 |
| hsa-mir-643 | 17 | 0 |
| hsa-mir-644b-3p | 0 | 0 |
| hsa-mir-651 | 6 | 6 |
| hsa-mir-652-3p | 466 | 209 |
| hsa-mir-652-5p | 0 | 0 |
| hsa-mir-654-3p | 406 | 9 |
| hsa-mir-654-5p | 124 | 106 |
| hsa-mir-655 | 12 | 12 |
| hsa-mir-656 | 9 | 9 |
| hsa-mir-660-3p | 12 | 5 |
| hsa-mir-660-5p | 138 | 115 |
| hsa-mir-664-3p | 30 | 20 |
| hsa-mir-664-5p | 1161 | 0 |
| hsa-mir-665 | 18 | 0 |
| hsa-mir-671-3p | 39 | 31 |
| hsa-mir-671-5p | 0 | 0 |
| hsa-mir-7-1-3p | 34 | 14 |
| hsa-mir-7-5p | 0 | 0 |
| hsa-mir-720 | 774 | 519 |
| hsa-mir-744-3p | 0 | 0 |
| hsa-mir-744-5p | 48532 | 31112 |
| hsa-mir-758 | 18 | 0 |
| hsa-mir-760 | 32 | 7 |
| hsa-mir-762 | 7 | 0 |
| hsa-mir-766-3p | 69 | 45 |
| hsa-mir-766-5p | 84 | 50 |
| hsa-mir-769-3p | 0 | 0 |
| hsa-mir-769-5p | 124 | 38 |
| hsa-mir-874 | 14 | 14 |
| hsa-mir-877-3p | 0 | 0 |
| hsa-mir-889 | 240 | 207 |
| hsa-mir-9-3p | 0 | 0 |
| hsa-mir-9-5p | 39 | 21 |
| hsa-mir-92a-1-5p | 18 | 10 |
| hsa-mir-92a-2-5p | 0 | 0 |
| hsa-mir-92a-3p | 48319 | 42612 |
| hsa-mir-92b-3p | 623 | 309 |
| hsa-mir-92b-5p | 18 | 0 |
| hsa-mir-93-3p | 0 | 0 |
| hsa-mir-93-5p | 1165 | 846 |
| hsa-mir-941 | 220 | 212 |
| hsa-mir-942 | 5 | 0 |
| hsa-mir-98 | 2319 | 1045 |
| hsa-mir-99a-3p | 0 | 0 |
| hsa-mir-99a-5p | 147 | 48 |
| hsa-mir-99b-3p | 48 | 31 |
| hsa-mir-99b-5p | 1954 | 1361 |
