# Supplementary material for: The Repertoire and Features of Human Platelet microRNAs
Source: PLoS One. 2012 Dec 4;7(12):e50746. doi: 10.1371/journal.pone.0050746 (PMC3514217; doi:10.1371/journal.pone.0050746)
Supplement: Database S1 — (ZIP) [file pone.0050746.s011.zip › Supporting Platelet microRNA sequence database S1/s7_sequence_s7run61.mir.loose.html]

s7run61

| Tissue | Total Reads | Usable Reads | % of Usable Reads |
| --- | --- | --- | --- |
| 0 | 0 | 0 | 0 |


s7run61 Total Exact and Loose Matches to miR

|  | Total Exact Match to miR | Total Exact Match to (+4) miR | Total Loose match to miR | Total miRNA (Exact to ( +4) miR + Loose) |
| --- | --- | --- | --- | --- |
| 0 | 0 | 0 | 0 | 0 |
| 0 | 0 | 0 | 0 | 0 |

s7run61 miR Exact and Loose Matches

| mature miRNA | Exact Match to miR | Exact Match to (+4) miR | Loose Match to miR |
| --- | --- | --- | --- |
| hsa-let-7a-2-3p | 0 | 0 | 0 |
| hsa-let-7a-3p | 0 | 0 | 6 |
| hsa-let-7a-5p | 372552 | 497978 | 28218 |
| hsa-let-7b-3p | 0 | 0 | 0 |
| hsa-let-7b-5p | 27832 | 47287 | 4596 |
| hsa-let-7c | 2058 | 4436 | 868 |
| hsa-let-7d-3p | 159 | 206 | 11 |
| hsa-let-7d-5p | 79335 | 96634 | 4673 |
| hsa-let-7e-3p | 0 | 0 | 0 |
| hsa-let-7e-5p | 21788 | 30478 | 2931 |
| hsa-let-7f-1-3p | 0 | 0 | 0 |
| hsa-let-7f-2-3p | 0 | 0 | 5 |
| hsa-let-7f-5p | 967100 | 1178191 | 51565 |
| hsa-let-7g-3p | 0 | 0 | 0 |
| hsa-let-7g-5p | 79689 | 94957 | 6266 |
| hsa-let-7i-3p | 9 | 9 | 7 |
| hsa-let-7i-5p | 34478 | 52507 | 7008 |
| hsa-mir-1 | 23782 | 25745 | 736 |
| hsa-mir-100-3p | 0 | 0 | 0 |
| hsa-mir-100-5p | 21 | 39 | 0 |
| hsa-mir-101-3p | 6108 | 30132 | 1838 |
| hsa-mir-101-5p | 0 | 0 | 0 |
| hsa-mir-103a-2-5p | 0 | 0 | 0 |
| hsa-mir-103a-3p | 191592 | 310373 | 27197 |
| hsa-mir-105-3p | 0 | 0 | 0 |
| hsa-mir-105-5p | 0 | 0 | 0 |
| hsa-mir-106a-3p | 0 | 0 | 0 |
| hsa-mir-106a-5p | 0 | 6 | 0 |
| hsa-mir-106b-3p | 245 | 452 | 87 |
| hsa-mir-106b-5p | 691 | 1296 | 103 |
| hsa-mir-107 | 15387 | 50610 | 5197 |
| hsa-mir-10a-3p | 0 | 9 | 0 |
| hsa-mir-10a-5p | 35 | 620 | 15 |
| hsa-mir-10b-3p | 0 | 0 | 0 |
| hsa-mir-10b-5p | 0 | 0 | 0 |
| hsa-mir-1178 | 0 | 0 | 0 |
| hsa-mir-1179 | 0 | 0 | 0 |
| hsa-mir-1180 | 0 | 0 | 0 |
| hsa-mir-1181 | 0 | 0 | 0 |
| hsa-mir-1182 | 0 | 0 | 0 |
| hsa-mir-1183 | 0 | 0 | 0 |
| hsa-mir-1184 | 0 | 0 | 0 |
| hsa-mir-1185-1-3p | 247 | 352 | 0 |
| hsa-mir-1185-2-3p | 76 | 122 | 0 |
| hsa-mir-1185-5p | 16 | 100 | 0 |
| hsa-mir-1193 | 0 | 0 | 0 |
| hsa-mir-1197 | 37 | 37 | 0 |
| hsa-mir-1200 | 0 | 0 | 0 |
| hsa-mir-1202 | 0 | 0 | 0 |
| hsa-mir-1203 | 0 | 0 | 0 |
| hsa-mir-1204 | 0 | 0 | 0 |
| hsa-mir-1205 | 0 | 0 | 0 |
| hsa-mir-1206 | 0 | 0 | 0 |
| hsa-mir-1207-3p | 0 | 0 | 0 |
| hsa-mir-1207-5p | 0 | 0 | 0 |
| hsa-mir-1208 | 0 | 0 | 0 |
| hsa-mir-122-3p | 0 | 0 | 0 |
| hsa-mir-122-5p | 13 | 13 | 5 |
| hsa-mir-1224-3p | 0 | 0 | 0 |
| hsa-mir-1225-3p | 0 | 0 | 0 |
| hsa-mir-1226-3p | 0 | 0 | 0 |
| hsa-mir-1227 | 0 | 0 | 0 |
| hsa-mir-1228-3p | 0 | 0 | 0 |
| hsa-mir-1229 | 0 | 0 | 0 |
| hsa-mir-1231 | 0 | 0 | 0 |
| hsa-mir-1233 | 0 | 0 | 0 |
| hsa-mir-1234 | 0 | 0 | 0 |
| hsa-mir-1236 | 0 | 0 | 0 |
| hsa-mir-1237 | 0 | 0 | 0 |
| hsa-mir-1238 | 0 | 0 | 0 |
| hsa-mir-124-3p | 0 | 0 | 0 |
| hsa-mir-124-5p | 0 | 0 | 0 |
| hsa-mir-1243 | 0 | 0 | 0 |
| hsa-mir-1244 | 0 | 0 | 0 |
| hsa-mir-1245a | 0 | 0 | 0 |
| hsa-mir-1245b-3p | 0 | 0 | 0 |
| hsa-mir-1245b-5p | 0 | 0 | 0 |
| hsa-mir-1246 | 0 | 0 | 0 |
| hsa-mir-1247-3p | 0 | 0 | 0 |
| hsa-mir-1247-5p | 0 | 0 | 0 |
| hsa-mir-1248 | 0 | 0 | 0 |
| hsa-mir-1249 | 0 | 0 | 0 |
| hsa-mir-1250 | 24 | 48 | 6 |
| hsa-mir-1251 | 0 | 0 | 0 |
| hsa-mir-1252 | 0 | 0 | 0 |
| hsa-mir-1253 | 0 | 0 | 0 |
| hsa-mir-1254 | 0 | 0 | 0 |
| hsa-mir-1255a | 19 | 56 | 0 |
| hsa-mir-1255b-2-3p | 0 | 0 | 0 |
| hsa-mir-1255b-5p | 12 | 12 | 0 |
| hsa-mir-1256 | 54 | 61 | 0 |
| hsa-mir-1257 | 0 | 0 | 0 |
| hsa-mir-1258 | 0 | 0 | 0 |
| hsa-mir-125a-3p | 0 | 6 | 0 |
| hsa-mir-125a-5p | 15 | 390 | 0 |
| hsa-mir-125b-1-3p | 0 | 0 | 0 |
| hsa-mir-125b-2-3p | 0 | 0 | 0 |
| hsa-mir-125b-5p | 42 | 66 | 0 |
| hsa-mir-126-3p | 910 | 4678 | 1118 |
| hsa-mir-126-5p | 6494 | 7174 | 1216 |
| hsa-mir-1260a | 0 | 0 | 398 |
| hsa-mir-1260b | 12 | 34 | 1348 |
| hsa-mir-1261 | 0 | 0 | 180 |
| hsa-mir-1262 | 0 | 0 | 0 |
| hsa-mir-1263 | 0 | 0 | 0 |
| hsa-mir-1264 | 0 | 0 | 0 |
| hsa-mir-1265 | 0 | 0 | 0 |
| hsa-mir-1266 | 0 | 0 | 0 |
| hsa-mir-1267 | 0 | 0 | 0 |
| hsa-mir-1268a | 0 | 0 | 0 |
| hsa-mir-1268b | 0 | 0 | 0 |
| hsa-mir-1269a | 0 | 0 | 0 |
| hsa-mir-1269b | 0 | 0 | 0 |
| hsa-mir-127-3p | 4047 | 4443 | 1229 |
| hsa-mir-127-5p | 36 | 101 | 0 |
| hsa-mir-1270 | 0 | 0 | 0 |
| hsa-mir-1271-3p | 0 | 0 | 0 |
| hsa-mir-1271-5p | 66 | 85 | 8 |
| hsa-mir-1272 | 0 | 0 | 0 |
| hsa-mir-1273a | 0 | 0 | 0 |
| hsa-mir-1273c | 0 | 6 | 7 |
| hsa-mir-1273d | 0 | 0 | 0 |
| hsa-mir-1273e | 0 | 0 | 0 |
| hsa-mir-1273f | 0 | 0 | 0 |
| hsa-mir-1273g-3p | 0 | 0 | 0 |
| hsa-mir-1273g-5p | 0 | 0 | 0 |
| hsa-mir-1275 | 0 | 0 | 0 |
| hsa-mir-1276 | 0 | 0 | 0 |
| hsa-mir-1277-3p | 1071 | 1218 | 11 |
| hsa-mir-1277-5p | 0 | 91 | 0 |
| hsa-mir-1278 | 88 | 125 | 0 |
| hsa-mir-1279 | 0 | 0 | 0 |
| hsa-mir-128 | 6230 | 9092 | 1312 |
| hsa-mir-1280 | 0 | 0 | 61 |
| hsa-mir-1281 | 0 | 0 | 0 |
| hsa-mir-1282 | 0 | 0 | 0 |
| hsa-mir-1283 | 0 | 0 | 0 |
| hsa-mir-1284 | 9 | 16 | 0 |
| hsa-mir-1285-3p | 42 | 69 | 36 |
| hsa-mir-1285-5p | 0 | 0 | 0 |
| hsa-mir-1286 | 0 | 0 | 0 |
| hsa-mir-1287 | 125 | 240 | 21 |
| hsa-mir-1288 | 0 | 0 | 0 |
| hsa-mir-1289 | 0 | 0 | 0 |
| hsa-mir-129-1-3p | 0 | 0 | 0 |
| hsa-mir-129-2-3p | 0 | 0 | 0 |
| hsa-mir-129-5p | 0 | 0 | 0 |
| hsa-mir-1290 | 0 | 0 | 0 |
| hsa-mir-1291 | 0 | 5 | 0 |
| hsa-mir-1292 | 0 | 0 | 0 |
| hsa-mir-1293 | 0 | 0 | 0 |
| hsa-mir-1294 | 34 | 45 | 0 |
| hsa-mir-1295a | 0 | 0 | 0 |
| hsa-mir-1295b-3p | 0 | 0 | 0 |
| hsa-mir-1296 | 0 | 0 | 0 |
| hsa-mir-1297 | 0 | 3 | 0 |
| hsa-mir-1298 | 0 | 0 | 0 |
| hsa-mir-1299 | 14 | 14 | 5 |
| hsa-mir-1301 | 32 | 327 | 35 |
| hsa-mir-1302 | 0 | 0 | 0 |
| hsa-mir-1303 | 0 | 0 | 0 |
| hsa-mir-1304-3p | 0 | 0 | 49 |
| hsa-mir-1304-5p | 0 | 17 | 0 |
| hsa-mir-1305 | 0 | 0 | 0 |
| hsa-mir-1306-3p | 0 | 54 | 0 |
| hsa-mir-1306-5p | 19 | 28 | 0 |
| hsa-mir-1307-3p | 770 | 3567 | 957 |
| hsa-mir-1307-5p | 1952 | 3832 | 150 |
| hsa-mir-130a-3p | 4234 | 4567 | 351 |
| hsa-mir-130a-5p | 0 | 0 | 0 |
| hsa-mir-130b-3p | 751 | 809 | 62 |
| hsa-mir-130b-5p | 12 | 65 | 0 |
| hsa-mir-132-3p | 17 | 17 | 19 |
| hsa-mir-132-5p | 8 | 8 | 0 |
| hsa-mir-1321 | 0 | 0 | 0 |
| hsa-mir-1322 | 0 | 0 | 0 |
| hsa-mir-1323 | 0 | 0 | 0 |
| hsa-mir-1324 | 0 | 0 | 0 |
| hsa-mir-133a | 0 | 10 | 0 |
| hsa-mir-133b | 0 | 0 | 0 |
| hsa-mir-134 | 479 | 1025 | 272 |
| hsa-mir-1343 | 0 | 0 | 0 |
| hsa-mir-135a-3p | 0 | 6 | 0 |
| hsa-mir-135a-5p | 0 | 0 | 0 |
| hsa-mir-135b-3p | 0 | 0 | 0 |
| hsa-mir-135b-5p | 0 | 0 | 0 |
| hsa-mir-136-3p | 50 | 65 | 0 |
| hsa-mir-136-5p | 73 | 97 | 6 |
| hsa-mir-137 | 0 | 0 | 0 |
| hsa-mir-138-1-3p | 0 | 0 | 0 |
| hsa-mir-138-2-3p | 0 | 0 | 0 |
| hsa-mir-138-5p | 0 | 0 | 0 |
| hsa-mir-139-3p | 16 | 486 | 38 |
| hsa-mir-139-5p | 54 | 320 | 14 |
| hsa-mir-140-3p | 2092 | 56986 | 8359 |
| hsa-mir-140-5p | 7 | 12 | 0 |
| hsa-mir-141-3p | 0 | 0 | 0 |
| hsa-mir-141-5p | 0 | 0 | 0 |
| hsa-mir-142-3p | 111 | 615 | 35 |
| hsa-mir-142-5p | 1102 | 15037 | 1011 |
| hsa-mir-143-3p | 2068 | 2756 | 181 |
| hsa-mir-143-5p | 29 | 133 | 9 |
| hsa-mir-144-3p | 29 | 273 | 39 |
| hsa-mir-144-5p | 36 | 202 | 0 |
| hsa-mir-145-3p | 0 | 0 | 0 |
| hsa-mir-145-5p | 0 | 0 | 0 |
| hsa-mir-1468 | 0 | 5 | 0 |
| hsa-mir-146a-3p | 0 | 0 | 0 |
| hsa-mir-146a-5p | 1108 | 2111 | 319 |
| hsa-mir-146b-3p | 0 | 8 | 0 |
| hsa-mir-146b-5p | 280 | 485 | 98 |
| hsa-mir-147a | 0 | 0 | 0 |
| hsa-mir-147b | 0 | 0 | 0 |
| hsa-mir-148a-3p | 1176 | 1453 | 100 |
| hsa-mir-148a-5p | 0 | 17 | 0 |
| hsa-mir-148b-3p | 2821 | 3643 | 661 |
| hsa-mir-148b-5p | 0 | 30 | 0 |
| hsa-mir-149-3p | 0 | 0 | 0 |
| hsa-mir-149-5p | 0 | 0 | 0 |
| hsa-mir-150-3p | 0 | 0 | 0 |
| hsa-mir-150-5p | 0 | 0 | 0 |
| hsa-mir-151a-3p | 2167 | 6058 | 907 |
| hsa-mir-151a-5p | 3327 | 6670 | 117 |
| hsa-mir-151b | 16 | 6706 | 0 |
| hsa-mir-152 | 6618 | 9406 | 2531 |
| hsa-mir-153 | 0 | 0 | 0 |
| hsa-mir-1537 | 0 | 0 | 0 |
| hsa-mir-1538 | 0 | 0 | 0 |
| hsa-mir-1539 | 0 | 0 | 0 |
| hsa-mir-154-3p | 84 | 109 | 0 |
| hsa-mir-154-5p | 59 | 59 | 0 |
| hsa-mir-155-3p | 0 | 0 | 0 |
| hsa-mir-155-5p | 16 | 130 | 42 |
| hsa-mir-1587 | 0 | 0 | 0 |
| hsa-mir-15a-3p | 8 | 8 | 0 |
| hsa-mir-15a-5p | 134 | 830 | 17 |
| hsa-mir-15b-3p | 6 | 17 | 0 |
| hsa-mir-15b-5p | 515 | 1581 | 48 |
| hsa-mir-16-1-3p | 21 | 21 | 0 |
| hsa-mir-16-2-3p | 0 | 11 | 0 |
| hsa-mir-16-5p | 5052 | 5824 | 458 |
| hsa-mir-17-3p | 213 | 415 | 58 |
| hsa-mir-17-5p | 346 | 711 | 40 |
| hsa-mir-181a-2-3p | 138 | 319 | 0 |
| hsa-mir-181a-3p | 201 | 636 | 7 |
| hsa-mir-181a-5p | 1024 | 8010 | 272 |
| hsa-mir-181b-3p | 5 | 5 | 12 |
| hsa-mir-181b-5p | 30 | 674 | 100 |
| hsa-mir-181c-3p | 6 | 407 | 106 |
| hsa-mir-181c-5p | 5 | 36 | 0 |
| hsa-mir-181d | 76 | 292 | 27 |
| hsa-mir-182-3p | 0 | 0 | 0 |
| hsa-mir-182-5p | 7 | 7 | 0 |
| hsa-mir-1825 | 0 | 0 | 0 |
| hsa-mir-1827 | 0 | 0 | 0 |
| hsa-mir-183-3p | 0 | 0 | 0 |
| hsa-mir-183-5p | 0 | 0 | 0 |
| hsa-mir-184 | 0 | 0 | 0 |
| hsa-mir-185-3p | 0 | 871 | 74 |
| hsa-mir-185-5p | 108162 | 120587 | 9752 |
| hsa-mir-186-3p | 0 | 0 | 0 |
| hsa-mir-186-5p | 1413 | 4467 | 186 |
| hsa-mir-187-3p | 0 | 0 | 0 |
| hsa-mir-187-5p | 0 | 0 | 0 |
| hsa-mir-188-3p | 0 | 0 | 0 |
| hsa-mir-188-5p | 0 | 0 | 0 |
| hsa-mir-18a-3p | 0 | 0 | 0 |
| hsa-mir-18a-5p | 12 | 168 | 0 |
| hsa-mir-18b-3p | 0 | 0 | 0 |
| hsa-mir-18b-5p | 0 | 0 | 0 |
| hsa-mir-1908 | 195 | 351 | 277 |
| hsa-mir-1909-3p | 0 | 0 | 0 |
| hsa-mir-1909-5p | 0 | 0 | 0 |
| hsa-mir-190a | 0 | 0 | 0 |
| hsa-mir-190b | 0 | 0 | 0 |
| hsa-mir-191-3p | 0 | 46 | 0 |
| hsa-mir-191-5p | 12079 | 24733 | 2904 |
| hsa-mir-1910 | 0 | 0 | 0 |
| hsa-mir-1911-3p | 0 | 0 | 0 |
| hsa-mir-1911-5p | 0 | 0 | 0 |
| hsa-mir-1912 | 0 | 0 | 0 |
| hsa-mir-1913 | 0 | 0 | 0 |
| hsa-mir-1914-3p | 0 | 0 | 0 |
| hsa-mir-1914-5p | 0 | 0 | 0 |
| hsa-mir-1915-3p | 0 | 0 | 0 |
| hsa-mir-1915-5p | 0 | 0 | 0 |
| hsa-mir-192-3p | 0 | 0 | 0 |
| hsa-mir-192-5p | 2910 | 5229 | 241 |
| hsa-mir-193a-3p | 0 | 0 | 0 |
| hsa-mir-193a-5p | 5 | 5 | 0 |
| hsa-mir-193b-3p | 0 | 0 | 0 |
| hsa-mir-193b-5p | 0 | 0 | 0 |
| hsa-mir-194-3p | 0 | 0 | 0 |
| hsa-mir-194-5p | 8 | 25 | 0 |
| hsa-mir-195-3p | 0 | 0 | 0 |
| hsa-mir-195-5p | 0 | 16 | 0 |
| hsa-mir-196a-3p | 0 | 0 | 0 |
| hsa-mir-196a-5p | 0 | 0 | 0 |
| hsa-mir-196b-3p | 0 | 0 | 0 |
| hsa-mir-196b-5p | 69 | 132 | 92 |
| hsa-mir-197-3p | 93 | 99 | 0 |
| hsa-mir-197-5p | 0 | 0 | 0 |
| hsa-mir-1972 | 0 | 0 | 0 |
| hsa-mir-1973 | 0 | 0 | 0 |
| hsa-mir-1976 | 0 | 0 | 0 |
| hsa-mir-198 | 0 | 0 | 0 |
| hsa-mir-199a-3p | 271365 | 499059 | 22855 |
| hsa-mir-199a-5p | 84 | 200 | 0 |
| hsa-mir-199b-3p | 271365 | 499059 | 22855 |
| hsa-mir-199b-5p | 0 | 0 | 0 |
| hsa-mir-19a-3p | 5 | 5 | 0 |
| hsa-mir-19a-5p | 0 | 0 | 0 |
| hsa-mir-19b-1-5p | 0 | 0 | 0 |
| hsa-mir-19b-2-5p | 0 | 0 | 0 |
| hsa-mir-19b-3p | 32 | 110 | 0 |
| hsa-mir-200a-3p | 0 | 6 | 0 |
| hsa-mir-200a-5p | 0 | 0 | 0 |
| hsa-mir-200b-3p | 6 | 11 | 0 |
| hsa-mir-200b-5p | 0 | 0 | 0 |
| hsa-mir-200c-3p | 0 | 18 | 0 |
| hsa-mir-200c-5p | 0 | 0 | 0 |
| hsa-mir-202-3p | 0 | 0 | 0 |
| hsa-mir-202-5p | 0 | 0 | 0 |
| hsa-mir-203 | 5 | 5 | 0 |
| hsa-mir-204-3p | 11 | 11 | 0 |
| hsa-mir-204-5p | 0 | 0 | 0 |
| hsa-mir-205-3p | 0 | 0 | 0 |
| hsa-mir-205-5p | 0 | 0 | 0 |
| hsa-mir-2052 | 0 | 0 | 0 |
| hsa-mir-2053 | 0 | 0 | 0 |
| hsa-mir-206 | 0 | 0 | 0 |
| hsa-mir-208a | 0 | 0 | 0 |
| hsa-mir-208b | 0 | 0 | 0 |
| hsa-mir-20a-3p | 0 | 12 | 0 |
| hsa-mir-20a-5p | 82 | 252 | 22 |
| hsa-mir-20b-3p | 0 | 6 | 0 |
| hsa-mir-20b-5p | 13 | 13 | 0 |
| hsa-mir-21-3p | 478 | 834 | 27 |
| hsa-mir-21-5p | 33230 | 101142 | 3339 |
| hsa-mir-210 | 76 | 82 | 5 |
| hsa-mir-211-3p | 0 | 0 | 0 |
| hsa-mir-211-5p | 0 | 0 | 0 |
| hsa-mir-2110 | 21 | 84 | 5 |
| hsa-mir-2113 | 0 | 0 | 0 |
| hsa-mir-2114-3p | 0 | 0 | 0 |
| hsa-mir-2114-5p | 0 | 0 | 0 |
| hsa-mir-2115-3p | 0 | 0 | 0 |
| hsa-mir-2115-5p | 0 | 0 | 0 |
| hsa-mir-2116-3p | 0 | 0 | 0 |
| hsa-mir-2116-5p | 0 | 0 | 0 |
| hsa-mir-2117 | 0 | 0 | 0 |
| hsa-mir-212-3p | 0 | 0 | 0 |
| hsa-mir-212-5p | 0 | 0 | 0 |
| hsa-mir-214-3p | 0 | 0 | 0 |
| hsa-mir-214-5p | 0 | 0 | 0 |
| hsa-mir-215 | 16 | 24 | 11 |
| hsa-mir-216a | 0 | 0 | 0 |
| hsa-mir-216b | 0 | 0 | 0 |
| hsa-mir-217 | 0 | 0 | 0 |
| hsa-mir-218-1-3p | 0 | 0 | 0 |
| hsa-mir-218-2-3p | 0 | 0 | 0 |
| hsa-mir-218-5p | 0 | 0 | 0 |
| hsa-mir-219-1-3p | 0 | 0 | 0 |
| hsa-mir-219-2-3p | 0 | 0 | 0 |
| hsa-mir-219-5p | 0 | 0 | 0 |
| hsa-mir-22-3p | 1851 | 2547 | 199 |
| hsa-mir-22-5p | 174 | 297 | 9 |
| hsa-mir-221-3p | 25441 | 53676 | 4172 |
| hsa-mir-221-5p | 120 | 266 | 0 |
| hsa-mir-222-3p | 331 | 3128 | 107 |
| hsa-mir-222-5p | 0 | 0 | 0 |
| hsa-mir-223-3p | 13693 | 31099 | 5503 |
| hsa-mir-223-5p | 0 | 0 | 0 |
| hsa-mir-224-3p | 0 | 8 | 0 |
| hsa-mir-224-5p | 35 | 832 | 0 |
| hsa-mir-2276 | 0 | 0 | 0 |
| hsa-mir-2277-3p | 0 | 0 | 0 |
| hsa-mir-2277-5p | 0 | 0 | 0 |
| hsa-mir-2278 | 0 | 0 | 0 |
| hsa-mir-2355-3p | 85 | 169 | 0 |
| hsa-mir-2355-5p | 61 | 386 | 14 |
| hsa-mir-2392 | 0 | 0 | 12265 |
| hsa-mir-23a-3p | 1868 | 10037 | 574 |
| hsa-mir-23a-5p | 6 | 12 | 0 |
| hsa-mir-23b-3p | 211 | 1752 | 64 |
| hsa-mir-23b-5p | 35 | 59 | 0 |
| hsa-mir-23c | 0 | 0 | 0 |
| hsa-mir-24-1-5p | 0 | 0 | 0 |
| hsa-mir-24-2-5p | 0 | 37 | 0 |
| hsa-mir-24-3p | 14688 | 18358 | 11130 |
| hsa-mir-2467-3p | 0 | 0 | 0 |
| hsa-mir-2467-5p | 0 | 0 | 0 |
| hsa-mir-25-3p | 48686 | 59251 | 5600 |
| hsa-mir-25-5p | 18 | 248 | 0 |
| hsa-mir-2681-3p | 0 | 0 | 0 |
| hsa-mir-2681-5p | 0 | 0 | 0 |
| hsa-mir-2682-3p | 0 | 0 | 0 |
| hsa-mir-2682-5p | 0 | 0 | 0 |
| hsa-mir-26a-1-3p | 13 | 13 | 0 |
| hsa-mir-26a-2-3p | 0 | 0 | 0 |
| hsa-mir-26a-5p | 28166 | 30972 | 8538 |
| hsa-mir-26b-3p | 0 | 0 | 0 |
| hsa-mir-26b-5p | 3340 | 49328 | 1546 |
| hsa-mir-27a-3p | 1359 | 9537 | 947 |
| hsa-mir-27a-5p | 0 | 0 | 0 |
| hsa-mir-27b-3p | 4874 | 7031 | 358 |
| hsa-mir-27b-5p | 10 | 33 | 0 |
| hsa-mir-28-3p | 438 | 528 | 70 |
| hsa-mir-28-5p | 312 | 1015 | 13 |
| hsa-mir-2861 | 0 | 0 | 0 |
| hsa-mir-2909 | 0 | 0 | 0 |
| hsa-mir-296-3p | 0 | 0 | 0 |
| hsa-mir-296-5p | 0 | 0 | 0 |
| hsa-mir-2964a-3p | 0 | 0 | 0 |
| hsa-mir-2964a-5p | 17 | 17 | 0 |
| hsa-mir-297 | 0 | 0 | 0 |
| hsa-mir-298 | 0 | 0 | 0 |
| hsa-mir-299-3p | 18 | 33 | 0 |
| hsa-mir-299-5p | 7 | 7 | 0 |
| hsa-mir-29a-3p | 10760 | 12623 | 476 |
| hsa-mir-29a-5p | 0 | 0 | 0 |
| hsa-mir-29b-1-5p | 0 | 0 | 0 |
| hsa-mir-29b-2-5p | 0 | 0 | 0 |
| hsa-mir-29b-3p | 66 | 134 | 0 |
| hsa-mir-29c-3p | 2191 | 2285 | 83 |
| hsa-mir-29c-5p | 0 | 13 | 0 |
| hsa-mir-300 | 0 | 0 | 0 |
| hsa-mir-301a-3p | 0 | 14 | 0 |
| hsa-mir-301a-5p | 58 | 109 | 0 |
| hsa-mir-301b | 0 | 0 | 0 |
| hsa-mir-302a-3p | 0 | 0 | 0 |
| hsa-mir-302a-5p | 0 | 0 | 0 |
| hsa-mir-302b-3p | 0 | 0 | 0 |
| hsa-mir-302b-5p | 0 | 0 | 0 |
| hsa-mir-302c-3p | 0 | 0 | 0 |
| hsa-mir-302c-5p | 0 | 0 | 0 |
| hsa-mir-302d-3p | 0 | 0 | 0 |
| hsa-mir-302d-5p | 0 | 0 | 0 |
| hsa-mir-302e | 0 | 0 | 0 |
| hsa-mir-302f | 0 | 0 | 0 |
| hsa-mir-3064-3p | 0 | 0 | 0 |
| hsa-mir-3064-5p | 0 | 0 | 0 |
| hsa-mir-3065-3p | 0 | 0 | 0 |
| hsa-mir-3065-5p | 0 | 0 | 0 |
| hsa-mir-3074-3p | 0 | 0 | 0 |
| hsa-mir-3074-5p | 0 | 0 | 0 |
| hsa-mir-30a-3p | 46 | 97 | 28 |
| hsa-mir-30a-5p | 85 | 396 | 9 |
| hsa-mir-30b-3p | 11 | 104 | 5 |
| hsa-mir-30b-5p | 208 | 228 | 16 |
| hsa-mir-30c-1-3p | 15 | 120 | 0 |
| hsa-mir-30c-2-3p | 0 | 5 | 0 |
| hsa-mir-30c-5p | 36 | 138 | 0 |
| hsa-mir-30d-3p | 0 | 0 | 0 |
| hsa-mir-30d-5p | 1409 | 12794 | 435 |
| hsa-mir-30e-3p | 1567 | 2465 | 966 |
| hsa-mir-30e-5p | 50 | 1244 | 46 |
| hsa-mir-31-3p | 0 | 0 | 0 |
| hsa-mir-31-5p | 0 | 7 | 0 |
| hsa-mir-3115 | 0 | 0 | 0 |
| hsa-mir-3116 | 0 | 0 | 0 |
| hsa-mir-3117-3p | 0 | 0 | 0 |
| hsa-mir-3117-5p | 0 | 0 | 0 |
| hsa-mir-3118 | 0 | 0 | 0 |
| hsa-mir-3119 | 0 | 0 | 0 |
| hsa-mir-3120-3p | 108 | 149 | 248 |
| hsa-mir-3120-5p | 0 | 0 | 0 |
| hsa-mir-3121-3p | 7 | 7 | 0 |
| hsa-mir-3121-5p | 0 | 0 | 0 |
| hsa-mir-3122 | 0 | 0 | 0 |
| hsa-mir-3123 | 0 | 43 | 694 |
| hsa-mir-3124-3p | 0 | 0 | 0 |
| hsa-mir-3124-5p | 16 | 21 | 6 |
| hsa-mir-3125 | 0 | 0 | 0 |
| hsa-mir-3126-3p | 0 | 0 | 0 |
| hsa-mir-3126-5p | 0 | 0 | 0 |
| hsa-mir-3127-3p | 0 | 0 | 0 |
| hsa-mir-3127-5p | 0 | 0 | 0 |
| hsa-mir-3128 | 0 | 0 | 0 |
| hsa-mir-3129-3p | 0 | 0 | 0 |
| hsa-mir-3129-5p | 0 | 0 | 0 |
| hsa-mir-3130-3p | 26 | 26 | 0 |
| hsa-mir-3130-5p | 0 | 0 | 0 |
| hsa-mir-3131 | 0 | 0 | 0 |
| hsa-mir-3132 | 0 | 0 | 0 |
| hsa-mir-3133 | 0 | 0 | 0 |
| hsa-mir-3134 | 0 | 0 | 0 |
| hsa-mir-3135a | 0 | 0 | 0 |
| hsa-mir-3135b | 0 | 0 | 251 |
| hsa-mir-3136-3p | 0 | 0 | 0 |
| hsa-mir-3136-5p | 0 | 16 | 0 |
| hsa-mir-3137 | 0 | 0 | 0 |
| hsa-mir-3138 | 0 | 63 | 0 |
| hsa-mir-3139 | 0 | 0 | 0 |
| hsa-mir-3140-3p | 0 | 5 | 0 |
| hsa-mir-3140-5p | 0 | 0 | 0 |
| hsa-mir-3141 | 0 | 0 | 10 |
| hsa-mir-3142 | 0 | 0 | 0 |
| hsa-mir-3143 | 0 | 5 | 0 |
| hsa-mir-3144-3p | 0 | 0 | 0 |
| hsa-mir-3144-5p | 0 | 0 | 0 |
| hsa-mir-3145-3p | 0 | 0 | 0 |
| hsa-mir-3145-5p | 0 | 0 | 0 |
| hsa-mir-3146 | 0 | 0 | 0 |
| hsa-mir-3147 | 0 | 0 | 0 |
| hsa-mir-3148 | 0 | 0 | 0 |
| hsa-mir-3149 | 0 | 0 | 0 |
| hsa-mir-3150a-3p | 0 | 0 | 0 |
| hsa-mir-3150a-5p | 0 | 0 | 0 |
| hsa-mir-3150b-3p | 0 | 0 | 0 |
| hsa-mir-3150b-5p | 0 | 0 | 0 |
| hsa-mir-3151 | 0 | 0 | 0 |
| hsa-mir-3152-3p | 0 | 0 | 0 |
| hsa-mir-3152-5p | 0 | 0 | 0 |
| hsa-mir-3153 | 0 | 0 | 0 |
| hsa-mir-3154 | 0 | 0 | 0 |
| hsa-mir-3155a | 0 | 0 | 0 |
| hsa-mir-3155b | 0 | 0 | 0 |
| hsa-mir-3156-3p | 0 | 0 | 0 |
| hsa-mir-3156-5p | 0 | 0 | 0 |
| hsa-mir-3157-3p | 0 | 0 | 0 |
| hsa-mir-3157-5p | 0 | 0 | 0 |
| hsa-mir-3158-3p | 0 | 0 | 0 |
| hsa-mir-3158-5p | 0 | 0 | 0 |
| hsa-mir-3159 | 0 | 0 | 0 |
| hsa-mir-3160-3p | 0 | 0 | 0 |
| hsa-mir-3160-5p | 0 | 0 | 0 |
| hsa-mir-3161 | 0 | 10 | 0 |
| hsa-mir-3162-3p | 0 | 0 | 0 |
| hsa-mir-3162-5p | 0 | 0 | 0 |
| hsa-mir-3163 | 0 | 0 | 0 |
| hsa-mir-3164 | 0 | 0 | 0 |
| hsa-mir-3165 | 0 | 0 | 0 |
| hsa-mir-3166 | 0 | 0 | 0 |
| hsa-mir-3167 | 0 | 0 | 0 |
| hsa-mir-3168 | 0 | 178 | 5715 |
| hsa-mir-3169 | 0 | 0 | 0 |
| hsa-mir-3170 | 0 | 0 | 0 |
| hsa-mir-3171 | 0 | 0 | 0 |
| hsa-mir-3173-3p | 0 | 0 | 0 |
| hsa-mir-3173-5p | 0 | 0 | 0 |
| hsa-mir-3174 | 0 | 17 | 0 |
| hsa-mir-3175 | 14 | 14 | 0 |
| hsa-mir-3176 | 0 | 0 | 0 |
| hsa-mir-3177-3p | 20 | 20 | 5 |
| hsa-mir-3177-5p | 0 | 0 | 0 |
| hsa-mir-3178 | 0 | 0 | 0 |
| hsa-mir-3179 | 0 | 0 | 0 |
| hsa-mir-3180 | 0 | 0 | 0 |
| hsa-mir-3180-3p | 0 | 0 | 0 |
| hsa-mir-3180-5p | 0 | 0 | 0 |
| hsa-mir-3181 | 0 | 0 | 0 |
| hsa-mir-3182 | 0 | 30 | 177 |
| hsa-mir-3183 | 17 | 17 | 0 |
| hsa-mir-3184-3p | 0 | 0 | 0 |
| hsa-mir-3184-5p | 0 | 0 | 0 |
| hsa-mir-3185 | 0 | 0 | 0 |
| hsa-mir-3186-3p | 0 | 0 | 0 |
| hsa-mir-3186-5p | 0 | 0 | 0 |
| hsa-mir-3187-3p | 0 | 0 | 0 |
| hsa-mir-3187-5p | 0 | 0 | 0 |
| hsa-mir-3188 | 0 | 0 | 0 |
| hsa-mir-3189-3p | 0 | 0 | 0 |
| hsa-mir-3189-5p | 0 | 0 | 0 |
| hsa-mir-3190-3p | 0 | 12 | 0 |
| hsa-mir-3190-5p | 0 | 0 | 0 |
| hsa-mir-3191-3p | 0 | 8 | 0 |
| hsa-mir-3191-5p | 0 | 0 | 0 |
| hsa-mir-3192 | 0 | 0 | 0 |
| hsa-mir-3194-3p | 0 | 0 | 0 |
| hsa-mir-3194-5p | 0 | 0 | 0 |
| hsa-mir-3195 | 0 | 0 | 0 |
| hsa-mir-3196 | 0 | 19 | 0 |
| hsa-mir-3197 | 0 | 0 | 0 |
| hsa-mir-3198 | 16 | 16 | 0 |
| hsa-mir-3199 | 0 | 0 | 0 |
| hsa-mir-32-3p | 0 | 0 | 0 |
| hsa-mir-32-5p | 0 | 56 | 5 |
| hsa-mir-3200-3p | 0 | 0 | 0 |
| hsa-mir-3200-5p | 0 | 0 | 0 |
| hsa-mir-3202 | 0 | 0 | 0 |
| hsa-mir-320a | 31152 | 40118 | 6755 |
| hsa-mir-320b | 66 | 310 | 90 |
| hsa-mir-320c | 42 | 70 | 177 |
| hsa-mir-320d | 6 | 22 | 22 |
| hsa-mir-320e | 0 | 0 | 0 |
| hsa-mir-323a-3p | 888 | 1334 | 51 |
| hsa-mir-323a-5p | 7 | 48 | 0 |
| hsa-mir-323b-3p | 14 | 1561 | 107 |
| hsa-mir-323b-5p | 0 | 0 | 0 |
| hsa-mir-324-3p | 0 | 14 | 0 |
| hsa-mir-324-5p | 42 | 59 | 0 |
| hsa-mir-325 | 0 | 0 | 0 |
| hsa-mir-326 | 9 | 9 | 201 |
| hsa-mir-328 | 136 | 154 | 11 |
| hsa-mir-329 | 24 | 76 | 30 |
| hsa-mir-330-3p | 1701 | 4087 | 370 |
| hsa-mir-330-5p | 0 | 0 | 6 |
| hsa-mir-331-3p | 166 | 267 | 64 |
| hsa-mir-331-5p | 0 | 28 | 0 |
| hsa-mir-335-3p | 22 | 30 | 5 |
| hsa-mir-335-5p | 295 | 1736 | 9 |
| hsa-mir-337-3p | 0 | 5 | 0 |
| hsa-mir-337-5p | 0 | 0 | 0 |
| hsa-mir-338-3p | 0 | 32 | 0 |
| hsa-mir-338-5p | 0 | 16 | 0 |
| hsa-mir-339-3p | 397 | 1044 | 417 |
| hsa-mir-339-5p | 101 | 912 | 120 |
| hsa-mir-33a-3p | 0 | 14 | 0 |
| hsa-mir-33a-5p | 2614 | 3907 | 256 |
| hsa-mir-33b-3p | 0 | 0 | 0 |
| hsa-mir-33b-5p | 90 | 142 | 0 |
| hsa-mir-340-3p | 0 | 0 | 0 |
| hsa-mir-340-5p | 14653 | 23788 | 1130 |
| hsa-mir-342-3p | 61 | 155 | 5 |
| hsa-mir-342-5p | 19 | 31 | 0 |
| hsa-mir-345-3p | 0 | 0 | 0 |
| hsa-mir-345-5p | 9 | 21 | 161 |
| hsa-mir-346 | 0 | 0 | 0 |
| hsa-mir-34a-3p | 0 | 0 | 0 |
| hsa-mir-34a-5p | 0 | 0 | 0 |
| hsa-mir-34b-3p | 0 | 0 | 0 |
| hsa-mir-34b-5p | 0 | 0 | 0 |
| hsa-mir-34c-3p | 0 | 0 | 0 |
| hsa-mir-34c-5p | 0 | 0 | 0 |
| hsa-mir-3529-3p | 0 | 0 | 0 |
| hsa-mir-3529-5p | 0 | 0 | 0 |
| hsa-mir-3545-3p | 0 | 0 | 0 |
| hsa-mir-3545-5p | 0 | 0 | 0 |
| hsa-mir-3591-3p | 0 | 0 | 0 |
| hsa-mir-3591-5p | 0 | 0 | 0 |
| hsa-mir-3605-3p | 0 | 0 | 0 |
| hsa-mir-3605-5p | 0 | 7 | 0 |
| hsa-mir-3606 | 0 | 0 | 0 |
| hsa-mir-3607-3p | 0 | 0 | 0 |
| hsa-mir-3607-5p | 0 | 0 | 0 |
| hsa-mir-3609 | 0 | 0 | 0 |
| hsa-mir-361-3p | 0 | 0 | 0 |
| hsa-mir-361-5p | 116 | 145 | 45 |
| hsa-mir-3610 | 0 | 0 | 0 |
| hsa-mir-3611 | 0 | 0 | 0 |
| hsa-mir-3612 | 0 | 0 | 0 |
| hsa-mir-3613-3p | 0 | 0 | 0 |
| hsa-mir-3613-5p | 0 | 0 | 0 |
| hsa-mir-3614-3p | 0 | 0 | 0 |
| hsa-mir-3614-5p | 0 | 0 | 0 |
| hsa-mir-3615 | 32 | 148 | 41 |
| hsa-mir-3616-3p | 0 | 0 | 0 |
| hsa-mir-3616-5p | 0 | 0 | 0 |
| hsa-mir-3617 | 0 | 0 | 0 |
| hsa-mir-3618 | 0 | 0 | 0 |
| hsa-mir-3619-3p | 0 | 0 | 0 |
| hsa-mir-3619-5p | 0 | 0 | 0 |
| hsa-mir-362-3p | 0 | 0 | 0 |
| hsa-mir-362-5p | 0 | 0 | 0 |
| hsa-mir-3620 | 0 | 0 | 0 |
| hsa-mir-3621 | 0 | 0 | 0 |
| hsa-mir-3622a-3p | 0 | 0 | 0 |
| hsa-mir-3622a-5p | 0 | 0 | 0 |
| hsa-mir-3622b-3p | 0 | 0 | 0 |
| hsa-mir-3622b-5p | 0 | 0 | 0 |
| hsa-mir-363-3p | 167 | 529 | 240 |
| hsa-mir-363-5p | 6 | 6 | 0 |
| hsa-mir-3646 | 0 | 0 | 0 |
| hsa-mir-3648 | 0 | 0 | 0 |
| hsa-mir-3649 | 0 | 0 | 0 |
| hsa-mir-3650 | 0 | 0 | 0 |
| hsa-mir-3651 | 0 | 0 | 0 |
| hsa-mir-3653 | 0 | 0 | 0 |
| hsa-mir-3654 | 0 | 0 | 0 |
| hsa-mir-3656 | 0 | 52 | 100 |
| hsa-mir-3657 | 0 | 0 | 0 |
| hsa-mir-3658 | 0 | 0 | 0 |
| hsa-mir-3659 | 0 | 0 | 0 |
| hsa-mir-365a-3p | 0 | 0 | 0 |
| hsa-mir-365a-5p | 0 | 0 | 0 |
| hsa-mir-365b-3p | 0 | 0 | 0 |
| hsa-mir-365b-5p | 0 | 0 | 0 |
| hsa-mir-3660 | 0 | 0 | 0 |
| hsa-mir-3661 | 0 | 0 | 0 |
| hsa-mir-3662 | 0 | 0 | 0 |
| hsa-mir-3663-3p | 0 | 0 | 0 |
| hsa-mir-3663-5p | 0 | 0 | 0 |
| hsa-mir-3664-3p | 0 | 0 | 0 |
| hsa-mir-3664-5p | 0 | 0 | 0 |
| hsa-mir-3665 | 0 | 0 | 0 |
| hsa-mir-3666 | 0 | 0 | 0 |
| hsa-mir-3667-3p | 0 | 0 | 0 |
| hsa-mir-3667-5p | 0 | 0 | 0 |
| hsa-mir-3668 | 0 | 0 | 0 |
| hsa-mir-3669 | 0 | 0 | 0 |
| hsa-mir-367-3p | 0 | 0 | 0 |
| hsa-mir-367-5p | 0 | 0 | 0 |
| hsa-mir-3670 | 0 | 0 | 0 |
| hsa-mir-3671 | 0 | 0 | 0 |
| hsa-mir-3672 | 0 | 0 | 0 |
| hsa-mir-3673 | 0 | 0 | 0 |
| hsa-mir-3674 | 0 | 0 | 0 |
| hsa-mir-3675-3p | 0 | 0 | 0 |
| hsa-mir-3675-5p | 0 | 0 | 0 |
| hsa-mir-3676-3p | 0 | 0 | 0 |
| hsa-mir-3676-5p | 12 | 28 | 12 |
| hsa-mir-3677-3p | 18 | 29 | 5 |
| hsa-mir-3677-5p | 0 | 0 | 0 |
| hsa-mir-3678-3p | 0 | 0 | 0 |
| hsa-mir-3678-5p | 0 | 0 | 0 |
| hsa-mir-3679-3p | 0 | 0 | 0 |
| hsa-mir-3679-5p | 0 | 0 | 0 |
| hsa-mir-3680-3p | 0 | 0 | 0 |
| hsa-mir-3680-5p | 0 | 0 | 0 |
| hsa-mir-3681-3p | 0 | 0 | 0 |
| hsa-mir-3681-5p | 0 | 0 | 0 |
| hsa-mir-3682-3p | 0 | 0 | 0 |
| hsa-mir-3682-5p | 0 | 0 | 0 |
| hsa-mir-3683 | 0 | 0 | 0 |
| hsa-mir-3684 | 0 | 0 | 0 |
| hsa-mir-3685 | 0 | 0 | 0 |
| hsa-mir-3686 | 0 | 0 | 0 |
| hsa-mir-3687 | 0 | 0 | 0 |
| hsa-mir-3688-3p | 0 | 0 | 0 |
| hsa-mir-3688-5p | 0 | 0 | 0 |
| hsa-mir-3689a-3p | 0 | 0 | 0 |
| hsa-mir-3689a-5p | 0 | 0 | 0 |
| hsa-mir-3689b-3p | 0 | 0 | 0 |
| hsa-mir-3689b-5p | 0 | 0 | 0 |
| hsa-mir-3689c | 0 | 0 | 0 |
| hsa-mir-3689d | 0 | 0 | 0 |
| hsa-mir-3689e | 0 | 0 | 0 |
| hsa-mir-3689f | 0 | 0 | 0 |
| hsa-mir-369-3p | 1404 | 1527 | 6 |
| hsa-mir-369-5p | 23 | 246 | 5 |
| hsa-mir-3690 | 0 | 0 | 0 |
| hsa-mir-3691-3p | 0 | 0 | 0 |
| hsa-mir-3691-5p | 0 | 0 | 0 |
| hsa-mir-3692-3p | 0 | 0 | 0 |
| hsa-mir-3692-5p | 0 | 0 | 0 |
| hsa-mir-370 | 13 | 18 | 22 |
| hsa-mir-371a-3p | 0 | 0 | 0 |
| hsa-mir-371a-5p | 0 | 0 | 0 |
| hsa-mir-371b-3p | 0 | 0 | 0 |
| hsa-mir-371b-5p | 0 | 0 | 0 |
| hsa-mir-372 | 0 | 0 | 0 |
| hsa-mir-373-3p | 0 | 0 | 0 |
| hsa-mir-373-5p | 0 | 0 | 0 |
| hsa-mir-374a-3p | 217 | 477 | 0 |
| hsa-mir-374a-5p | 624 | 1304 | 48 |
| hsa-mir-374b-3p | 19 | 60 | 0 |
| hsa-mir-374b-5p | 433 | 548 | 30 |
| hsa-mir-374c-3p | 0 | 0 | 0 |
| hsa-mir-374c-5p | 0 | 0 | 0 |
| hsa-mir-375 | 5 | 5 | 0 |
| hsa-mir-376a-3p | 30 | 30 | 140 |
| hsa-mir-376a-5p | 0 | 0 | 0 |
| hsa-mir-376b | 10 | 72 | 6 |
| hsa-mir-376c | 252 | 270 | 49 |
| hsa-mir-377-3p | 10 | 10 | 0 |
| hsa-mir-377-5p | 35 | 35 | 0 |
| hsa-mir-378a-3p | 932 | 1642 | 419 |
| hsa-mir-378a-5p | 0 | 0 | 0 |
| hsa-mir-378b | 0 | 1 | 0 |
| hsa-mir-378c | 0 | 259 | 6 |
| hsa-mir-378d | 6 | 54 | 0 |
| hsa-mir-378e | 0 | 1 | 0 |
| hsa-mir-378f | 0 | 1 | 18 |
| hsa-mir-378g | 0 | 0 | 0 |
| hsa-mir-378h | 0 | 0 | 0 |
| hsa-mir-378i | 0 | 0 | 62 |
| hsa-mir-379-3p | 0 | 14 | 9 |
| hsa-mir-379-5p | 804 | 968 | 212 |
| hsa-mir-380-3p | 0 | 8 | 0 |
| hsa-mir-380-5p | 0 | 0 | 0 |
| hsa-mir-381 | 58 | 78 | 5 |
| hsa-mir-382-3p | 126 | 203 | 0 |
| hsa-mir-382-5p | 1196 | 1772 | 25 |
| hsa-mir-383 | 0 | 0 | 0 |
| hsa-mir-384 | 0 | 0 | 0 |
| hsa-mir-3907 | 0 | 0 | 0 |
| hsa-mir-3908 | 0 | 0 | 0 |
| hsa-mir-3909 | 0 | 0 | 0 |
| hsa-mir-3910 | 0 | 0 | 0 |
| hsa-mir-3911 | 0 | 0 | 0 |
| hsa-mir-3912 | 7 | 7 | 0 |
| hsa-mir-3913-3p | 0 | 0 | 0 |
| hsa-mir-3913-5p | 0 | 0 | 0 |
| hsa-mir-3914 | 0 | 0 | 0 |
| hsa-mir-3915 | 0 | 0 | 0 |
| hsa-mir-3916 | 0 | 0 | 0 |
| hsa-mir-3917 | 0 | 0 | 0 |
| hsa-mir-3918 | 0 | 0 | 0 |
| hsa-mir-3919 | 0 | 0 | 0 |
| hsa-mir-3920 | 5 | 5 | 0 |
| hsa-mir-3921 | 0 | 0 | 0 |
| hsa-mir-3922-3p | 0 | 0 | 0 |
| hsa-mir-3922-5p | 0 | 0 | 0 |
| hsa-mir-3923 | 0 | 0 | 0 |
| hsa-mir-3924 | 0 | 0 | 0 |
| hsa-mir-3925-3p | 0 | 0 | 0 |
| hsa-mir-3925-5p | 0 | 0 | 0 |
| hsa-mir-3926 | 0 | 0 | 0 |
| hsa-mir-3927 | 0 | 0 | 0 |
| hsa-mir-3928 | 60 | 76 | 59 |
| hsa-mir-3929 | 0 | 0 | 0 |
| hsa-mir-3934 | 0 | 0 | 0 |
| hsa-mir-3935 | 0 | 0 | 0 |
| hsa-mir-3936 | 0 | 0 | 0 |
| hsa-mir-3937 | 0 | 0 | 0 |
| hsa-mir-3938 | 0 | 0 | 0 |
| hsa-mir-3939 | 0 | 0 | 0 |
| hsa-mir-3940-3p | 0 | 0 | 0 |
| hsa-mir-3940-5p | 0 | 0 | 0 |
| hsa-mir-3941 | 0 | 0 | 0 |
| hsa-mir-3942-3p | 0 | 0 | 0 |
| hsa-mir-3942-5p | 0 | 0 | 0 |
| hsa-mir-3943 | 0 | 0 | 0 |
| hsa-mir-3944-3p | 0 | 0 | 0 |
| hsa-mir-3944-5p | 0 | 0 | 0 |
| hsa-mir-3945 | 0 | 0 | 0 |
| hsa-mir-3960 | 0 | 0 | 0 |
| hsa-mir-3972 | 0 | 0 | 0 |
| hsa-mir-3973 | 0 | 0 | 0 |
| hsa-mir-3974 | 0 | 0 | 0 |
| hsa-mir-3975 | 0 | 0 | 0 |
| hsa-mir-3976 | 0 | 0 | 0 |
| hsa-mir-3977 | 0 | 0 | 0 |
| hsa-mir-3978 | 0 | 0 | 0 |
| hsa-mir-409-3p | 277 | 621 | 20 |
| hsa-mir-409-5p | 161 | 233 | 0 |
| hsa-mir-410 | 409 | 436 | 39 |
| hsa-mir-411-3p | 0 | 44 | 35 |
| hsa-mir-411-5p | 311 | 664 | 5 |
| hsa-mir-412 | 0 | 0 | 0 |
| hsa-mir-421 | 24 | 146 | 13 |
| hsa-mir-422a | 0 | 0 | 0 |
| hsa-mir-423-3p | 2799 | 4279 | 552 |
| hsa-mir-423-5p | 16882 | 38466 | 1705 |
| hsa-mir-424-3p | 257 | 392 | 10 |
| hsa-mir-424-5p | 8 | 60 | 0 |
| hsa-mir-425-3p | 66 | 1220 | 226 |
| hsa-mir-425-5p | 293 | 526 | 8 |
| hsa-mir-4251 | 0 | 0 | 0 |
| hsa-mir-4252 | 0 | 0 | 0 |
| hsa-mir-4253 | 0 | 0 | 0 |
| hsa-mir-4254 | 0 | 0 | 0 |
| hsa-mir-4255 | 0 | 0 | 0 |
| hsa-mir-4256 | 0 | 0 | 0 |
| hsa-mir-4257 | 0 | 0 | 0 |
| hsa-mir-4258 | 0 | 0 | 0 |
| hsa-mir-4259 | 0 | 0 | 0 |
| hsa-mir-4260 | 0 | 0 | 0 |
| hsa-mir-4261 | 0 | 0 | 0 |
| hsa-mir-4262 | 0 | 0 | 0 |
| hsa-mir-4263 | 0 | 0 | 0 |
| hsa-mir-4264 | 0 | 0 | 0 |
| hsa-mir-4265 | 0 | 0 | 0 |
| hsa-mir-4266 | 0 | 0 | 0 |
| hsa-mir-4267 | 0 | 0 | 0 |
| hsa-mir-4268 | 0 | 0 | 0 |
| hsa-mir-4269 | 0 | 0 | 0 |
| hsa-mir-4270 | 0 | 0 | 0 |
| hsa-mir-4271 | 0 | 0 | 0 |
| hsa-mir-4272 | 0 | 0 | 0 |
| hsa-mir-4273 | 0 | 0 | 0 |
| hsa-mir-4274 | 0 | 0 | 0 |
| hsa-mir-4275 | 0 | 0 | 0 |
| hsa-mir-4276 | 0 | 0 | 0 |
| hsa-mir-4277 | 0 | 0 | 0 |
| hsa-mir-4278 | 0 | 0 | 0 |
| hsa-mir-4279 | 0 | 0 | 0 |
| hsa-mir-4280 | 0 | 0 | 0 |
| hsa-mir-4281 | 0 | 0 | 0 |
| hsa-mir-4282 | 0 | 0 | 0 |
| hsa-mir-4283 | 0 | 0 | 0 |
| hsa-mir-4284 | 0 | 0 | 0 |
| hsa-mir-4285 | 0 | 0 | 0 |
| hsa-mir-4286 | 0 | 1002 | 31 |
| hsa-mir-4287 | 0 | 0 | 0 |
| hsa-mir-4288 | 0 | 0 | 0 |
| hsa-mir-4289 | 0 | 0 | 0 |
| hsa-mir-429 | 0 | 0 | 0 |
| hsa-mir-4290 | 0 | 0 | 0 |
| hsa-mir-4291 | 0 | 0 | 0 |
| hsa-mir-4292 | 0 | 0 | 0 |
| hsa-mir-4293 | 0 | 0 | 0 |
| hsa-mir-4294 | 0 | 0 | 0 |
| hsa-mir-4295 | 0 | 0 | 0 |
| hsa-mir-4296 | 0 | 0 | 0 |
| hsa-mir-4297 | 0 | 0 | 0 |
| hsa-mir-4298 | 0 | 0 | 0 |
| hsa-mir-4299 | 0 | 0 | 0 |
| hsa-mir-4300 | 0 | 0 | 0 |
| hsa-mir-4301 | 0 | 0 | 0 |
| hsa-mir-4302 | 0 | 0 | 0 |
| hsa-mir-4303 | 0 | 0 | 0 |
| hsa-mir-4304 | 0 | 0 | 0 |
| hsa-mir-4305 | 0 | 0 | 0 |
| hsa-mir-4306 | 0 | 109 | 26 |
| hsa-mir-4307 | 0 | 0 | 0 |
| hsa-mir-4308 | 0 | 0 | 0 |
| hsa-mir-4309 | 0 | 0 | 0 |
| hsa-mir-431-3p | 7 | 22 | 0 |
| hsa-mir-431-5p | 14 | 54 | 0 |
| hsa-mir-4310 | 0 | 0 | 0 |
| hsa-mir-4311 | 0 | 0 | 0 |
| hsa-mir-4312 | 0 | 0 | 0 |
| hsa-mir-4313 | 0 | 0 | 0 |
| hsa-mir-4314 | 0 | 0 | 0 |
| hsa-mir-4315 | 0 | 0 | 0 |
| hsa-mir-4316 | 0 | 0 | 0 |
| hsa-mir-4317 | 0 | 0 | 0 |
| hsa-mir-4318 | 0 | 0 | 0 |
| hsa-mir-4319 | 0 | 0 | 0 |
| hsa-mir-432-3p | 0 | 0 | 0 |
| hsa-mir-432-5p | 261 | 1984 | 575 |
| hsa-mir-4320 | 0 | 0 | 0 |
| hsa-mir-4321 | 0 | 0 | 0 |
| hsa-mir-4322 | 0 | 0 | 0 |
| hsa-mir-4323 | 0 | 0 | 0 |
| hsa-mir-4324 | 0 | 0 | 0 |
| hsa-mir-4325 | 0 | 0 | 0 |
| hsa-mir-4326 | 0 | 0 | 0 |
| hsa-mir-4327 | 0 | 0 | 0 |
| hsa-mir-4328 | 0 | 0 | 0 |
| hsa-mir-4329 | 0 | 0 | 0 |
| hsa-mir-433 | 1505 | 1821 | 80 |
| hsa-mir-4330 | 0 | 0 | 0 |
| hsa-mir-4417 | 0 | 0 | 0 |
| hsa-mir-4418 | 0 | 0 | 0 |
| hsa-mir-4419a | 0 | 0 | 0 |
| hsa-mir-4419b | 0 | 0 | 0 |
| hsa-mir-4420 | 0 | 0 | 0 |
| hsa-mir-4421 | 0 | 0 | 0 |
| hsa-mir-4422 | 0 | 0 | 0 |
| hsa-mir-4423-3p | 0 | 0 | 0 |
| hsa-mir-4423-5p | 0 | 0 | 0 |
| hsa-mir-4424 | 0 | 0 | 0 |
| hsa-mir-4425 | 0 | 0 | 0 |
| hsa-mir-4426 | 0 | 0 | 0 |
| hsa-mir-4427 | 0 | 0 | 0 |
| hsa-mir-4428 | 0 | 0 | 0 |
| hsa-mir-4429 | 0 | 0 | 19 |
| hsa-mir-4430 | 0 | 0 | 0 |
| hsa-mir-4431 | 0 | 0 | 0 |
| hsa-mir-4432 | 0 | 0 | 0 |
| hsa-mir-4433-3p | 31 | 41 | 8754 |
| hsa-mir-4433-5p | 0 | 0 | 23 |
| hsa-mir-4434 | 0 | 0 | 0 |
| hsa-mir-4435 | 68 | 74 | 8 |
| hsa-mir-4436a | 0 | 0 | 0 |
| hsa-mir-4436b-3p | 0 | 10 | 0 |
| hsa-mir-4436b-5p | 0 | 0 | 0 |
| hsa-mir-4437 | 0 | 0 | 0 |
| hsa-mir-4438 | 0 | 0 | 0 |
| hsa-mir-4439 | 0 | 0 | 0 |
| hsa-mir-4440 | 0 | 0 | 0 |
| hsa-mir-4441 | 0 | 0 | 0 |
| hsa-mir-4442 | 0 | 0 | 0 |
| hsa-mir-4443 | 0 | 71 | 16 |
| hsa-mir-4444 | 0 | 0 | 0 |
| hsa-mir-4445-3p | 0 | 0 | 0 |
| hsa-mir-4445-5p | 0 | 0 | 0 |
| hsa-mir-4446-3p | 503 | 678 | 163 |
| hsa-mir-4446-5p | 0 | 0 | 0 |
| hsa-mir-4447 | 0 | 0 | 37 |
| hsa-mir-4448 | 0 | 21 | 87 |
| hsa-mir-4449 | 0 | 0 | 0 |
| hsa-mir-4450 | 0 | 0 | 0 |
| hsa-mir-4451 | 0 | 0 | 0 |
| hsa-mir-4452 | 0 | 0 | 0 |
| hsa-mir-4453 | 0 | 0 | 0 |
| hsa-mir-4454 | 0 | 38 | 102 |
| hsa-mir-4455 | 0 | 0 | 126 |
| hsa-mir-4456 | 0 | 0 | 0 |
| hsa-mir-4457 | 0 | 0 | 0 |
| hsa-mir-4458 | 0 | 0 | 0 |
| hsa-mir-4459 | 0 | 0 | 0 |
| hsa-mir-4460 | 0 | 0 | 0 |
| hsa-mir-4461 | 0 | 0 | 0 |
| hsa-mir-4462 | 0 | 0 | 0 |
| hsa-mir-4463 | 0 | 0 | 0 |
| hsa-mir-4464 | 0 | 0 | 0 |
| hsa-mir-4465 | 0 | 0 | 0 |
| hsa-mir-4466 | 0 | 0 | 11 |
| hsa-mir-4467 | 0 | 0 | 0 |
| hsa-mir-4468 | 0 | 0 | 0 |
| hsa-mir-4469 | 14 | 14 | 0 |
| hsa-mir-4470 | 19 | 19 | 0 |
| hsa-mir-4471 | 0 | 0 | 0 |
| hsa-mir-4472 | 0 | 0 | 0 |
| hsa-mir-4473 | 0 | 0 | 0 |
| hsa-mir-4474-3p | 0 | 0 | 0 |
| hsa-mir-4474-5p | 0 | 0 | 0 |
| hsa-mir-4475 | 0 | 0 | 0 |
| hsa-mir-4476 | 0 | 0 | 0 |
| hsa-mir-4477a | 0 | 0 | 0 |
| hsa-mir-4477b | 0 | 0 | 0 |
| hsa-mir-4478 | 0 | 0 | 0 |
| hsa-mir-4479 | 0 | 0 | 0 |
| hsa-mir-448 | 0 | 0 | 0 |
| hsa-mir-4480 | 0 | 0 | 0 |
| hsa-mir-4482-3p | 0 | 0 | 0 |
| hsa-mir-4482-5p | 0 | 0 | 0 |
| hsa-mir-4483 | 0 | 0 | 28 |
| hsa-mir-4484 | 0 | 0 | 0 |
| hsa-mir-4485 | 0 | 0 | 0 |
| hsa-mir-4486 | 0 | 0 | 0 |
| hsa-mir-4487 | 0 | 0 | 0 |
| hsa-mir-4488 | 0 | 21 | 0 |
| hsa-mir-4489 | 0 | 0 | 0 |
| hsa-mir-4490 | 0 | 0 | 0 |
| hsa-mir-4491 | 0 | 0 | 0 |
| hsa-mir-4492 | 0 | 53 | 0 |
| hsa-mir-4493 | 0 | 0 | 0 |
| hsa-mir-4494 | 0 | 0 | 0 |
| hsa-mir-4495 | 0 | 0 | 0 |
| hsa-mir-4496 | 0 | 0 | 0 |
| hsa-mir-4497 | 0 | 0 | 46 |
| hsa-mir-4498 | 0 | 0 | 0 |
| hsa-mir-449a | 0 | 0 | 0 |
| hsa-mir-449b-3p | 0 | 0 | 0 |
| hsa-mir-449b-5p | 0 | 0 | 0 |
| hsa-mir-449c-3p | 0 | 0 | 0 |
| hsa-mir-449c-5p | 0 | 0 | 0 |
| hsa-mir-4500 | 0 | 29 | 0 |
| hsa-mir-4502 | 0 | 0 | 0 |
| hsa-mir-4503 | 0 | 0 | 0 |
| hsa-mir-4504 | 0 | 0 | 0 |
| hsa-mir-4505 | 0 | 0 | 0 |
| hsa-mir-4506 | 0 | 0 | 0 |
| hsa-mir-4507 | 0 | 0 | 0 |
| hsa-mir-4508 | 6 | 1699 | 3707 |
| hsa-mir-4509 | 0 | 0 | 0 |
| hsa-mir-450a-3p | 0 | 5 | 0 |
| hsa-mir-450a-5p | 10 | 24 | 0 |
| hsa-mir-450b-3p | 0 | 0 | 0 |
| hsa-mir-450b-5p | 0 | 13 | 0 |
| hsa-mir-4510 | 0 | 0 | 1 |
| hsa-mir-4511 | 0 | 0 | 0 |
| hsa-mir-4512 | 0 | 0 | 0 |
| hsa-mir-4513 | 0 | 0 | 0 |
| hsa-mir-4514 | 0 | 0 | 0 |
| hsa-mir-4515 | 0 | 0 | 0 |
| hsa-mir-4516 | 0 | 51 | 31 |
| hsa-mir-4517 | 0 | 0 | 0 |
| hsa-mir-4518 | 0 | 0 | 0 |
| hsa-mir-4519 | 0 | 0 | 0 |
| hsa-mir-451a | 232 | 436 | 0 |
| hsa-mir-451b | 0 | 0 | 0 |
| hsa-mir-452-3p | 0 | 0 | 0 |
| hsa-mir-452-5p | 40 | 229 | 0 |
| hsa-mir-4520a-3p | 0 | 0 | 0 |
| hsa-mir-4520a-5p | 0 | 0 | 0 |
| hsa-mir-4520b-3p | 0 | 0 | 0 |
| hsa-mir-4520b-5p | 0 | 0 | 0 |
| hsa-mir-4521 | 0 | 0 | 0 |
| hsa-mir-4522 | 0 | 0 | 0 |
| hsa-mir-4523 | 21 | 21 | 0 |
| hsa-mir-4524a-3p | 0 | 0 | 0 |
| hsa-mir-4524a-5p | 0 | 0 | 0 |
| hsa-mir-4524b-3p | 0 | 0 | 0 |
| hsa-mir-4524b-5p | 0 | 0 | 0 |
| hsa-mir-4525 | 0 | 0 | 0 |
| hsa-mir-4526 | 0 | 0 | 0 |
| hsa-mir-4527 | 0 | 0 | 0 |
| hsa-mir-4528 | 0 | 0 | 0 |
| hsa-mir-4529-3p | 0 | 0 | 0 |
| hsa-mir-4529-5p | 0 | 0 | 0 |
| hsa-mir-4530 | 0 | 0 | 0 |
| hsa-mir-4531 | 0 | 0 | 86 |
| hsa-mir-4532 | 0 | 0 | 0 |
| hsa-mir-4533 | 0 | 0 | 0 |
| hsa-mir-4534 | 0 | 0 | 0 |
| hsa-mir-4535 | 0 | 0 | 0 |
| hsa-mir-4536-3p | 0 | 0 | 0 |
| hsa-mir-4536-5p | 0 | 0 | 0 |
| hsa-mir-4539 | 0 | 0 | 0 |
| hsa-mir-454-3p | 0 | 0 | 0 |
| hsa-mir-454-5p | 7 | 7 | 0 |
| hsa-mir-4540 | 0 | 0 | 0 |
| hsa-mir-455-3p | 0 | 0 | 0 |
| hsa-mir-455-5p | 0 | 0 | 0 |
| hsa-mir-4632 | 0 | 0 | 0 |
| hsa-mir-4633-3p | 0 | 0 | 0 |
| hsa-mir-4633-5p | 0 | 0 | 0 |
| hsa-mir-4634 | 0 | 0 | 0 |
| hsa-mir-4635 | 0 | 0 | 0 |
| hsa-mir-4636 | 0 | 0 | 0 |
| hsa-mir-4637 | 0 | 0 | 0 |
| hsa-mir-4638-3p | 0 | 0 | 0 |
| hsa-mir-4638-5p | 0 | 0 | 0 |
| hsa-mir-4639-3p | 0 | 0 | 0 |
| hsa-mir-4640-3p | 0 | 0 | 0 |
| hsa-mir-4640-5p | 0 | 0 | 0 |
| hsa-mir-4641 | 0 | 0 | 0 |
| hsa-mir-4642 | 0 | 0 | 0 |
| hsa-mir-4643 | 0 | 0 | 0 |
| hsa-mir-4644 | 0 | 0 | 0 |
| hsa-mir-4645-3p | 0 | 0 | 0 |
| hsa-mir-4645-5p | 0 | 0 | 0 |
| hsa-mir-4646-3p | 0 | 0 | 0 |
| hsa-mir-4647 | 0 | 0 | 0 |
| hsa-mir-4649-3p | 0 | 0 | 0 |
| hsa-mir-4649-5p | 0 | 0 | 0 |
| hsa-mir-4650-3p | 0 | 0 | 0 |
| hsa-mir-4650-5p | 0 | 0 | 0 |
| hsa-mir-4651 | 0 | 0 | 0 |
| hsa-mir-4652-3p | 0 | 0 | 0 |
| hsa-mir-4652-5p | 0 | 0 | 0 |
| hsa-mir-4653-3p | 0 | 0 | 0 |
| hsa-mir-4653-5p | 0 | 0 | 0 |
| hsa-mir-4654 | 0 | 0 | 0 |
| hsa-mir-4655-3p | 0 | 0 | 0 |
| hsa-mir-4655-5p | 0 | 0 | 0 |
| hsa-mir-4656 | 0 | 0 | 0 |
| hsa-mir-4658 | 0 | 0 | 0 |
| hsa-mir-4659a-3p | 0 | 0 | 0 |
| hsa-mir-4659a-5p | 0 | 0 | 0 |
| hsa-mir-4659b-3p | 0 | 18 | 0 |
| hsa-mir-4659b-5p | 0 | 0 | 0 |
| hsa-mir-466 | 0 | 0 | 0 |
| hsa-mir-4660 | 0 | 0 | 0 |
| hsa-mir-4661-3p | 0 | 0 | 0 |
| hsa-mir-4661-5p | 0 | 0 | 0 |
| hsa-mir-4662a-3p | 0 | 0 | 0 |
| hsa-mir-4662a-5p | 0 | 0 | 0 |
| hsa-mir-4662b | 0 | 0 | 0 |
| hsa-mir-4663 | 0 | 0 | 0 |
| hsa-mir-4664-3p | 0 | 0 | 0 |
| hsa-mir-4664-5p | 0 | 0 | 0 |
| hsa-mir-4665-3p | 0 | 0 | 0 |
| hsa-mir-4665-5p | 12 | 12 | 0 |
| hsa-mir-4666a-3p | 0 | 0 | 0 |
| hsa-mir-4666a-5p | 0 | 0 | 0 |
| hsa-mir-4666b | 0 | 0 | 0 |
| hsa-mir-4667-3p | 0 | 0 | 0 |
| hsa-mir-4667-5p | 5 | 5 | 0 |
| hsa-mir-4668-3p | 0 | 0 | 0 |
| hsa-mir-4669 | 0 | 0 | 0 |
| hsa-mir-4670-3p | 0 | 0 | 0 |
| hsa-mir-4670-5p | 0 | 0 | 0 |
| hsa-mir-4671-3p | 0 | 0 | 0 |
| hsa-mir-4671-5p | 0 | 0 | 0 |
| hsa-mir-4672 | 0 | 0 | 0 |
| hsa-mir-4673 | 0 | 0 | 0 |
| hsa-mir-4674 | 0 | 0 | 0 |
| hsa-mir-4675 | 0 | 0 | 0 |
| hsa-mir-4676-3p | 0 | 0 | 0 |
| hsa-mir-4676-5p | 0 | 0 | 0 |
| hsa-mir-4677-3p | 0 | 0 | 0 |
| hsa-mir-4677-5p | 0 | 0 | 0 |
| hsa-mir-4678 | 0 | 0 | 0 |
| hsa-mir-4679 | 0 | 0 | 0 |
| hsa-mir-4680-3p | 0 | 0 | 0 |
| hsa-mir-4680-5p | 0 | 0 | 0 |
| hsa-mir-4681 | 0 | 0 | 0 |
| hsa-mir-4682 | 0 | 0 | 0 |
| hsa-mir-4683 | 0 | 0 | 0 |
| hsa-mir-4684-3p | 0 | 0 | 0 |
| hsa-mir-4684-5p | 0 | 0 | 0 |
| hsa-mir-4685-3p | 0 | 0 | 0 |
| hsa-mir-4685-5p | 0 | 0 | 0 |
| hsa-mir-4686 | 0 | 0 | 0 |
| hsa-mir-4687-3p | 0 | 0 | 0 |
| hsa-mir-4687-5p | 0 | 0 | 0 |
| hsa-mir-4688 | 0 | 0 | 0 |
| hsa-mir-4689 | 0 | 0 | 0 |
| hsa-mir-4690-3p | 0 | 0 | 0 |
| hsa-mir-4691-3p | 0 | 0 | 0 |
| hsa-mir-4691-5p | 0 | 0 | 0 |
| hsa-mir-4692 | 0 | 0 | 0 |
| hsa-mir-4693-3p | 0 | 0 | 0 |
| hsa-mir-4693-5p | 0 | 0 | 0 |
| hsa-mir-4694-3p | 0 | 0 | 0 |
| hsa-mir-4694-5p | 0 | 0 | 0 |
| hsa-mir-4695-3p | 0 | 0 | 0 |
| hsa-mir-4695-5p | 0 | 0 | 0 |
| hsa-mir-4696 | 0 | 0 | 0 |
| hsa-mir-4697-3p | 0 | 0 | 0 |
| hsa-mir-4697-5p | 0 | 0 | 0 |
| hsa-mir-4698 | 0 | 0 | 0 |
| hsa-mir-4699-3p | 0 | 0 | 0 |
| hsa-mir-4699-5p | 0 | 0 | 0 |
| hsa-mir-4700-3p | 0 | 0 | 0 |
| hsa-mir-4700-5p | 0 | 0 | 0 |
| hsa-mir-4701-3p | 0 | 0 | 0 |
| hsa-mir-4701-5p | 0 | 0 | 0 |
| hsa-mir-4703-3p | 0 | 0 | 0 |
| hsa-mir-4703-5p | 0 | 0 | 0 |
| hsa-mir-4704-3p | 0 | 0 | 0 |
| hsa-mir-4704-5p | 0 | 0 | 0 |
| hsa-mir-4705 | 0 | 0 | 0 |
| hsa-mir-4706 | 0 | 0 | 0 |
| hsa-mir-4707-3p | 8 | 8 | 0 |
| hsa-mir-4707-5p | 0 | 0 | 0 |
| hsa-mir-4708-3p | 5 | 5 | 0 |
| hsa-mir-4708-5p | 0 | 0 | 0 |
| hsa-mir-4709-3p | 0 | 0 | 0 |
| hsa-mir-4709-5p | 0 | 0 | 0 |
| hsa-mir-4710 | 0 | 0 | 0 |
| hsa-mir-4711-3p | 0 | 0 | 0 |
| hsa-mir-4711-5p | 0 | 0 | 0 |
| hsa-mir-4712-3p | 0 | 0 | 0 |
| hsa-mir-4712-5p | 0 | 0 | 0 |
| hsa-mir-4713-3p | 0 | 0 | 0 |
| hsa-mir-4713-5p | 0 | 0 | 0 |
| hsa-mir-4714-3p | 0 | 0 | 0 |
| hsa-mir-4714-5p | 0 | 0 | 0 |
| hsa-mir-4715-3p | 0 | 0 | 0 |
| hsa-mir-4715-5p | 0 | 0 | 0 |
| hsa-mir-4716-3p | 0 | 0 | 0 |
| hsa-mir-4716-5p | 0 | 0 | 0 |
| hsa-mir-4717-3p | 0 | 0 | 0 |
| hsa-mir-4717-5p | 0 | 0 | 0 |
| hsa-mir-4719 | 0 | 0 | 0 |
| hsa-mir-4720-3p | 0 | 0 | 0 |
| hsa-mir-4720-5p | 0 | 0 | 0 |
| hsa-mir-4721 | 0 | 0 | 0 |
| hsa-mir-4722-3p | 0 | 0 | 0 |
| hsa-mir-4723-3p | 0 | 0 | 0 |
| hsa-mir-4723-5p | 0 | 0 | 0 |
| hsa-mir-4724-3p | 0 | 0 | 0 |
| hsa-mir-4724-5p | 0 | 0 | 0 |
| hsa-mir-4725-3p | 0 | 0 | 0 |
| hsa-mir-4725-5p | 0 | 0 | 0 |
| hsa-mir-4726-3p | 0 | 0 | 0 |
| hsa-mir-4727-3p | 0 | 0 | 0 |
| hsa-mir-4727-5p | 0 | 0 | 0 |
| hsa-mir-4728-3p | 0 | 0 | 0 |
| hsa-mir-4728-5p | 0 | 0 | 0 |
| hsa-mir-4729 | 0 | 0 | 0 |
| hsa-mir-4730 | 0 | 0 | 0 |
| hsa-mir-4731-3p | 0 | 0 | 0 |
| hsa-mir-4731-5p | 0 | 0 | 0 |
| hsa-mir-4732-3p | 0 | 0 | 0 |
| hsa-mir-4732-5p | 0 | 0 | 0 |
| hsa-mir-4733-3p | 0 | 0 | 0 |
| hsa-mir-4733-5p | 0 | 0 | 0 |
| hsa-mir-4734 | 0 | 11 | 0 |
| hsa-mir-4735-3p | 0 | 0 | 0 |
| hsa-mir-4735-5p | 0 | 0 | 0 |
| hsa-mir-4737 | 0 | 0 | 0 |
| hsa-mir-4738-3p | 0 | 0 | 0 |
| hsa-mir-4738-5p | 0 | 0 | 0 |
| hsa-mir-4739 | 0 | 0 | 0 |
| hsa-mir-4740-3p | 0 | 0 | 0 |
| hsa-mir-4740-5p | 0 | 0 | 0 |
| hsa-mir-4741 | 0 | 0 | 0 |
| hsa-mir-4742-3p | 0 | 0 | 0 |
| hsa-mir-4743 | 0 | 0 | 0 |
| hsa-mir-4744 | 0 | 0 | 0 |
| hsa-mir-4745-3p | 0 | 0 | 0 |
| hsa-mir-4745-5p | 0 | 0 | 0 |
| hsa-mir-4746-3p | 0 | 0 | 0 |
| hsa-mir-4746-5p | 0 | 0 | 0 |
| hsa-mir-4747-3p | 0 | 0 | 0 |
| hsa-mir-4748 | 0 | 0 | 0 |
| hsa-mir-4749-3p | 0 | 0 | 0 |
| hsa-mir-4749-5p | 0 | 0 | 0 |
| hsa-mir-4750 | 0 | 0 | 12 |
| hsa-mir-4751 | 0 | 0 | 0 |
| hsa-mir-4752 | 0 | 0 | 0 |
| hsa-mir-4753-3p | 0 | 0 | 0 |
| hsa-mir-4753-5p | 0 | 0 | 0 |
| hsa-mir-4754 | 0 | 0 | 0 |
| hsa-mir-4755-3p | 0 | 0 | 0 |
| hsa-mir-4755-5p | 0 | 0 | 0 |
| hsa-mir-4756-3p | 0 | 0 | 0 |
| hsa-mir-4756-5p | 0 | 0 | 0 |
| hsa-mir-4757-3p | 0 | 0 | 0 |
| hsa-mir-4757-5p | 0 | 0 | 0 |
| hsa-mir-4758-3p | 0 | 0 | 0 |
| hsa-mir-4758-5p | 0 | 0 | 0 |
| hsa-mir-4759 | 0 | 0 | 0 |
| hsa-mir-4760-3p | 0 | 0 | 0 |
| hsa-mir-4760-5p | 0 | 0 | 0 |
| hsa-mir-4761-3p | 0 | 0 | 0 |
| hsa-mir-4761-5p | 0 | 0 | 0 |
| hsa-mir-4762-3p | 0 | 0 | 0 |
| hsa-mir-4762-5p | 0 | 0 | 0 |
| hsa-mir-4763-3p | 0 | 0 | 0 |
| hsa-mir-4763-5p | 0 | 0 | 0 |
| hsa-mir-4764-3p | 0 | 0 | 0 |
| hsa-mir-4764-5p | 0 | 0 | 0 |
| hsa-mir-4765 | 5 | 5 | 0 |
| hsa-mir-4766-3p | 0 | 0 | 0 |
| hsa-mir-4766-5p | 0 | 0 | 0 |
| hsa-mir-4767 | 0 | 0 | 0 |
| hsa-mir-4768-3p | 0 | 0 | 0 |
| hsa-mir-4768-5p | 0 | 0 | 0 |
| hsa-mir-4769-3p | 0 | 0 | 0 |
| hsa-mir-4769-5p | 0 | 0 | 0 |
| hsa-mir-4770 | 0 | 0 | 6 |
| hsa-mir-4771 | 0 | 0 | 0 |
| hsa-mir-4772-3p | 0 | 0 | 0 |
| hsa-mir-4772-5p | 0 | 0 | 0 |
| hsa-mir-4773 | 0 | 0 | 0 |
| hsa-mir-4774-3p | 0 | 0 | 0 |
| hsa-mir-4774-5p | 0 | 0 | 0 |
| hsa-mir-4775 | 0 | 0 | 0 |
| hsa-mir-4776-3p | 0 | 0 | 0 |
| hsa-mir-4776-5p | 0 | 0 | 0 |
| hsa-mir-4777-3p | 0 | 0 | 0 |
| hsa-mir-4777-5p | 0 | 0 | 0 |
| hsa-mir-4778-3p | 0 | 0 | 0 |
| hsa-mir-4778-5p | 0 | 0 | 0 |
| hsa-mir-4779 | 0 | 0 | 0 |
| hsa-mir-4780 | 0 | 0 | 0 |
| hsa-mir-4781-3p | 0 | 0 | 0 |
| hsa-mir-4781-5p | 0 | 0 | 0 |
| hsa-mir-4782-3p | 0 | 0 | 0 |
| hsa-mir-4782-5p | 0 | 0 | 0 |
| hsa-mir-4783-3p | 0 | 0 | 0 |
| hsa-mir-4783-5p | 0 | 0 | 0 |
| hsa-mir-4784 | 0 | 0 | 0 |
| hsa-mir-4785 | 0 | 0 | 0 |
| hsa-mir-4786-3p | 0 | 0 | 0 |
| hsa-mir-4786-5p | 0 | 0 | 0 |
| hsa-mir-4787-3p | 0 | 0 | 0 |
| hsa-mir-4787-5p | 0 | 0 | 0 |
| hsa-mir-4788 | 0 | 0 | 0 |
| hsa-mir-4789-3p | 0 | 0 | 0 |
| hsa-mir-4789-5p | 0 | 0 | 0 |
| hsa-mir-4790-3p | 0 | 0 | 0 |
| hsa-mir-4790-5p | 0 | 0 | 0 |
| hsa-mir-4791 | 0 | 5 | 0 |
| hsa-mir-4792 | 0 | 0 | 0 |
| hsa-mir-4793-3p | 0 | 0 | 0 |
| hsa-mir-4793-5p | 0 | 0 | 0 |
| hsa-mir-4794 | 0 | 0 | 0 |
| hsa-mir-4795-3p | 0 | 0 | 0 |
| hsa-mir-4795-5p | 0 | 0 | 0 |
| hsa-mir-4796-3p | 0 | 0 | 0 |
| hsa-mir-4796-5p | 0 | 0 | 0 |
| hsa-mir-4797-3p | 0 | 0 | 0 |
| hsa-mir-4797-5p | 0 | 0 | 0 |
| hsa-mir-4798-3p | 0 | 0 | 0 |
| hsa-mir-4798-5p | 0 | 0 | 0 |
| hsa-mir-4799-3p | 0 | 0 | 0 |
| hsa-mir-4799-5p | 0 | 0 | 0 |
| hsa-mir-4800-3p | 0 | 0 | 0 |
| hsa-mir-4800-5p | 0 | 0 | 0 |
| hsa-mir-4801 | 0 | 0 | 0 |
| hsa-mir-4802-3p | 0 | 0 | 0 |
| hsa-mir-4802-5p | 0 | 0 | 0 |
| hsa-mir-4803 | 0 | 0 | 0 |
| hsa-mir-4804-3p | 0 | 0 | 0 |
| hsa-mir-4804-5p | 0 | 0 | 0 |
| hsa-mir-483-3p | 0 | 0 | 0 |
| hsa-mir-483-5p | 0 | 0 | 0 |
| hsa-mir-484 | 224 | 343 | 58 |
| hsa-mir-485-3p | 1702 | 1838 | 158 |
| hsa-mir-485-5p | 367 | 754 | 31 |
| hsa-mir-486-3p | 1703 | 2482 | 471 |
| hsa-mir-486-5p | 1486 | 3165 | 1482 |
| hsa-mir-487a | 22 | 28 | 0 |
| hsa-mir-487b | 434 | 511 | 5 |
| hsa-mir-488-3p | 0 | 0 | 0 |
| hsa-mir-488-5p | 0 | 0 | 0 |
| hsa-mir-489 | 0 | 0 | 0 |
| hsa-mir-490-3p | 5 | 10 | 0 |
| hsa-mir-490-5p | 0 | 0 | 0 |
| hsa-mir-491-3p | 0 | 0 | 0 |
| hsa-mir-491-5p | 5 | 5 | 0 |
| hsa-mir-492 | 0 | 0 | 0 |
| hsa-mir-493-3p | 94 | 113 | 32 |
| hsa-mir-493-5p | 113 | 193 | 0 |
| hsa-mir-494 | 5 | 280 | 0 |
| hsa-mir-495 | 1595 | 2804 | 63 |
| hsa-mir-496 | 73 | 163 | 49 |
| hsa-mir-497-3p | 0 | 0 | 0 |
| hsa-mir-497-5p | 36 | 57 | 0 |
| hsa-mir-498 | 0 | 0 | 0 |
| hsa-mir-4999-3p | 0 | 0 | 0 |
| hsa-mir-4999-5p | 0 | 0 | 0 |
| hsa-mir-499a-3p | 0 | 0 | 0 |
| hsa-mir-499a-5p | 76 | 135 | 0 |
| hsa-mir-499b-3p | 0 | 0 | 0 |
| hsa-mir-499b-5p | 5 | 5 | 0 |
| hsa-mir-5000-3p | 0 | 0 | 0 |
| hsa-mir-5000-5p | 0 | 0 | 0 |
| hsa-mir-5001-3p | 0 | 0 | 0 |
| hsa-mir-5001-5p | 0 | 0 | 0 |
| hsa-mir-5002-3p | 0 | 0 | 0 |
| hsa-mir-5002-5p | 0 | 0 | 0 |
| hsa-mir-5003-3p | 0 | 0 | 0 |
| hsa-mir-5003-5p | 0 | 0 | 0 |
| hsa-mir-5004-3p | 0 | 0 | 0 |
| hsa-mir-5004-5p | 0 | 0 | 0 |
| hsa-mir-5006-3p | 0 | 0 | 0 |
| hsa-mir-5006-5p | 0 | 0 | 0 |
| hsa-mir-5007-3p | 0 | 0 | 0 |
| hsa-mir-5007-5p | 0 | 0 | 0 |
| hsa-mir-5008-3p | 0 | 0 | 0 |
| hsa-mir-5008-5p | 0 | 0 | 0 |
| hsa-mir-5009-3p | 0 | 0 | 0 |
| hsa-mir-5009-5p | 0 | 0 | 0 |
| hsa-mir-500a-3p | 0 | 69 | 0 |
| hsa-mir-500a-5p | 0 | 0 | 0 |
| hsa-mir-500b | 0 | 0 | 0 |
| hsa-mir-501-3p | 13 | 21 | 0 |
| hsa-mir-501-5p | 0 | 0 | 0 |
| hsa-mir-5010-3p | 0 | 0 | 0 |
| hsa-mir-5010-5p | 160 | 316 | 8 |
| hsa-mir-5011-3p | 0 | 0 | 0 |
| hsa-mir-5011-5p | 0 | 0 | 0 |
| hsa-mir-502-3p | 38 | 57 | 0 |
| hsa-mir-502-5p | 0 | 0 | 0 |
| hsa-mir-503 | 335 | 607 | 132 |
| hsa-mir-504 | 14 | 14 | 0 |
| hsa-mir-5047 | 0 | 0 | 0 |
| hsa-mir-505-3p | 0 | 0 | 0 |
| hsa-mir-505-5p | 30 | 66 | 0 |
| hsa-mir-506-3p | 0 | 0 | 0 |
| hsa-mir-506-5p | 0 | 0 | 0 |
| hsa-mir-507 | 0 | 0 | 0 |
| hsa-mir-508-3p | 0 | 0 | 0 |
| hsa-mir-508-5p | 0 | 0 | 0 |
| hsa-mir-5087 | 0 | 0 | 0 |
| hsa-mir-5088 | 0 | 0 | 0 |
| hsa-mir-5089 | 0 | 0 | 0 |
| hsa-mir-509-3-5p | 0 | 0 | 0 |
| hsa-mir-509-3p | 0 | 0 | 0 |
| hsa-mir-509-5p | 0 | 0 | 0 |
| hsa-mir-5090 | 0 | 0 | 0 |
| hsa-mir-5091 | 0 | 0 | 0 |
| hsa-mir-5092 | 0 | 0 | 0 |
| hsa-mir-5093 | 0 | 0 | 0 |
| hsa-mir-5094 | 0 | 0 | 0 |
| hsa-mir-5095 | 0 | 0 | 0 |
| hsa-mir-5096 | 0 | 0 | 0 |
| hsa-mir-510 | 0 | 0 | 0 |
| hsa-mir-5100 | 0 | 0 | 0 |
| hsa-mir-511 | 0 | 0 | 0 |
| hsa-mir-512-3p | 0 | 0 | 0 |
| hsa-mir-512-5p | 0 | 0 | 0 |
| hsa-mir-513a-3p | 0 | 0 | 0 |
| hsa-mir-513a-5p | 0 | 0 | 0 |
| hsa-mir-513b | 0 | 0 | 0 |
| hsa-mir-513c-3p | 0 | 0 | 0 |
| hsa-mir-513c-5p | 0 | 0 | 0 |
| hsa-mir-514a-3p | 0 | 0 | 0 |
| hsa-mir-514a-5p | 0 | 0 | 0 |
| hsa-mir-514b-3p | 0 | 0 | 0 |
| hsa-mir-514b-5p | 0 | 0 | 0 |
| hsa-mir-515-3p | 0 | 0 | 0 |
| hsa-mir-515-5p | 0 | 0 | 0 |
| hsa-mir-516a-3p | 0 | 0 | 0 |
| hsa-mir-516a-5p | 0 | 0 | 0 |
| hsa-mir-516b-3p | 0 | 0 | 0 |
| hsa-mir-516b-5p | 0 | 0 | 0 |
| hsa-mir-517-5p | 0 | 0 | 0 |
| hsa-mir-517a-3p | 0 | 0 | 0 |
| hsa-mir-517b-3p | 0 | 0 | 0 |
| hsa-mir-517c-3p | 0 | 0 | 0 |
| hsa-mir-5186 | 0 | 0 | 0 |
| hsa-mir-5187-3p | 0 | 0 | 0 |
| hsa-mir-5187-5p | 64 | 80 | 0 |
| hsa-mir-5188 | 0 | 0 | 0 |
| hsa-mir-5189 | 0 | 117 | 0 |
| hsa-mir-518a-3p | 0 | 0 | 0 |
| hsa-mir-518a-5p | 0 | 0 | 0 |
| hsa-mir-518b | 0 | 0 | 0 |
| hsa-mir-518c-3p | 0 | 0 | 0 |
| hsa-mir-518c-5p | 0 | 0 | 0 |
| hsa-mir-518d-3p | 0 | 0 | 0 |
| hsa-mir-518d-5p | 0 | 0 | 0 |
| hsa-mir-518e-3p | 0 | 0 | 0 |
| hsa-mir-518e-5p | 0 | 0 | 0 |
| hsa-mir-518f-3p | 0 | 0 | 0 |
| hsa-mir-518f-5p | 0 | 0 | 0 |
| hsa-mir-5190 | 0 | 0 | 0 |
| hsa-mir-5191 | 0 | 0 | 0 |
| hsa-mir-5192 | 0 | 0 | 0 |
| hsa-mir-5193 | 0 | 0 | 0 |
| hsa-mir-5194 | 0 | 0 | 0 |
| hsa-mir-5195-3p | 0 | 0 | 0 |
| hsa-mir-5195-5p | 0 | 0 | 0 |
| hsa-mir-5196-3p | 0 | 0 | 0 |
| hsa-mir-5196-5p | 0 | 0 | 0 |
| hsa-mir-5197-3p | 0 | 0 | 0 |
| hsa-mir-5197-5p | 0 | 0 | 0 |
| hsa-mir-519a-3p | 0 | 0 | 0 |
| hsa-mir-519a-5p | 0 | 0 | 0 |
| hsa-mir-519b-3p | 0 | 0 | 0 |
| hsa-mir-519b-5p | 0 | 0 | 0 |
| hsa-mir-519c-3p | 0 | 0 | 0 |
| hsa-mir-519c-5p | 0 | 0 | 0 |
| hsa-mir-519d | 0 | 0 | 0 |
| hsa-mir-519e-3p | 0 | 0 | 0 |
| hsa-mir-519e-5p | 0 | 0 | 0 |
| hsa-mir-520a-3p | 0 | 0 | 0 |
| hsa-mir-520a-5p | 0 | 0 | 0 |
| hsa-mir-520b | 0 | 0 | 0 |
| hsa-mir-520c-3p | 0 | 0 | 0 |
| hsa-mir-520c-5p | 0 | 0 | 0 |
| hsa-mir-520d-3p | 0 | 0 | 0 |
| hsa-mir-520d-5p | 0 | 0 | 0 |
| hsa-mir-520e | 0 | 0 | 0 |
| hsa-mir-520f | 0 | 0 | 0 |
| hsa-mir-520g | 0 | 0 | 0 |
| hsa-mir-520h | 0 | 0 | 0 |
| hsa-mir-521 | 0 | 0 | 0 |
| hsa-mir-522-3p | 0 | 0 | 0 |
| hsa-mir-522-5p | 0 | 0 | 0 |
| hsa-mir-523-3p | 0 | 0 | 0 |
| hsa-mir-523-5p | 0 | 0 | 0 |
| hsa-mir-524-3p | 0 | 0 | 0 |
| hsa-mir-524-5p | 0 | 0 | 0 |
| hsa-mir-525-3p | 0 | 0 | 0 |
| hsa-mir-525-5p | 0 | 0 | 0 |
| hsa-mir-526a | 0 | 0 | 0 |
| hsa-mir-526b-3p | 0 | 0 | 0 |
| hsa-mir-526b-5p | 0 | 0 | 0 |
| hsa-mir-527 | 0 | 0 | 0 |
| hsa-mir-532-3p | 15 | 15 | 0 |
| hsa-mir-532-5p | 600 | 635 | 14 |
| hsa-mir-539-3p | 167 | 329 | 0 |
| hsa-mir-539-5p | 6 | 6 | 0 |
| hsa-mir-541-3p | 0 | 0 | 0 |
| hsa-mir-541-5p | 0 | 0 | 0 |
| hsa-mir-542-3p | 37 | 89 | 0 |
| hsa-mir-542-5p | 0 | 0 | 0 |
| hsa-mir-543 | 988 | 1752 | 321 |
| hsa-mir-544a | 0 | 0 | 0 |
| hsa-mir-544b | 0 | 0 | 0 |
| hsa-mir-545-3p | 0 | 0 | 0 |
| hsa-mir-545-5p | 0 | 0 | 0 |
| hsa-mir-548a-3p | 0 | 0 | 0 |
| hsa-mir-548a-5p | 0 | 0 | 0 |
| hsa-mir-548aa | 0 | 0 | 0 |
| hsa-mir-548ab | 0 | 0 | 0 |
| hsa-mir-548ac | 0 | 0 | 0 |
| hsa-mir-548ad | 0 | 0 | 0 |
| hsa-mir-548ae | 0 | 0 | 0 |
| hsa-mir-548ag | 0 | 0 | 0 |
| hsa-mir-548ah-3p | 0 | 6 | 0 |
| hsa-mir-548ah-5p | 0 | 0 | 0 |
| hsa-mir-548ai | 0 | 0 | 0 |
| hsa-mir-548aj-3p | 0 | 0 | 0 |
| hsa-mir-548aj-5p | 0 | 0 | 0 |
| hsa-mir-548ak | 0 | 0 | 0 |
| hsa-mir-548al | 0 | 0 | 0 |
| hsa-mir-548am-3p | 0 | 0 | 0 |
| hsa-mir-548am-5p | 0 | 60 | 0 |
| hsa-mir-548an | 0 | 0 | 0 |
| hsa-mir-548ao-3p | 0 | 0 | 0 |
| hsa-mir-548ao-5p | 0 | 0 | 0 |
| hsa-mir-548ap-3p | 0 | 0 | 0 |
| hsa-mir-548ap-5p | 0 | 965 | 0 |
| hsa-mir-548aq-3p | 0 | 0 | 0 |
| hsa-mir-548ar-3p | 0 | 0 | 0 |
| hsa-mir-548ar-5p | 0 | 0 | 0 |
| hsa-mir-548as-3p | 0 | 0 | 0 |
| hsa-mir-548at-3p | 0 | 0 | 0 |
| hsa-mir-548au-3p | 0 | 0 | 0 |
| hsa-mir-548au-5p | 3 | 60 | 0 |
| hsa-mir-548av-3p | 0 | 10 | 0 |
| hsa-mir-548av-5p | 0 | 107 | 0 |
| hsa-mir-548aw | 0 | 0 | 0 |
| hsa-mir-548ax | 0 | 0 | 0 |
| hsa-mir-548b-3p | 0 | 0 | 0 |
| hsa-mir-548b-5p | 0 | 0 | 0 |
| hsa-mir-548c-3p | 0 | 0 | 0 |
| hsa-mir-548c-5p | 0 | 60 | 0 |
| hsa-mir-548d-3p | 0 | 0 | 0 |
| hsa-mir-548d-5p | 0 | 2 | 0 |
| hsa-mir-548e | 52 | 94 | 0 |
| hsa-mir-548f | 0 | 0 | 0 |
| hsa-mir-548g-3p | 0 | 0 | 0 |
| hsa-mir-548g-5p | 0 | 0 | 0 |
| hsa-mir-548h-3p | 0 | 0 | 0 |
| hsa-mir-548h-5p | 0 | 0 | 0 |
| hsa-mir-548i | 0 | 0 | 0 |
| hsa-mir-548j | 793 | 965 | 9 |
| hsa-mir-548k | 100 | 107 | 0 |
| hsa-mir-548l | 0 | 7 | 0 |
| hsa-mir-548m | 0 | 0 | 0 |
| hsa-mir-548n | 8 | 8 | 0 |
| hsa-mir-548o-3p | 10 | 10 | 0 |
| hsa-mir-548o-5p | 0 | 60 | 0 |
| hsa-mir-548p | 6 | 6 | 0 |
| hsa-mir-548q | 0 | 0 | 0 |
| hsa-mir-548s | 0 | 0 | 0 |
| hsa-mir-548t-3p | 0 | 0 | 0 |
| hsa-mir-548t-5p | 0 | 0 | 0 |
| hsa-mir-548u | 0 | 0 | 0 |
| hsa-mir-548v | 0 | 0 | 0 |
| hsa-mir-548w | 0 | 0 | 0 |
| hsa-mir-548x-3p | 0 | 0 | 0 |
| hsa-mir-548x-5p | 0 | 0 | 0 |
| hsa-mir-548y | 0 | 0 | 0 |
| hsa-mir-548z | 0 | 0 | 0 |
| hsa-mir-549 | 0 | 0 | 0 |
| hsa-mir-550a-3-5p | 0 | 0 | 0 |
| hsa-mir-550a-3p | 6 | 6 | 0 |
| hsa-mir-550a-5p | 0 | 0 | 0 |
| hsa-mir-550b-2-5p | 0 | 0 | 0 |
| hsa-mir-550b-3p | 0 | 0 | 0 |
| hsa-mir-551a | 0 | 0 | 0 |
| hsa-mir-551b-3p | 0 | 11 | 11 |
| hsa-mir-551b-5p | 0 | 0 | 0 |
| hsa-mir-552 | 10 | 10 | 0 |
| hsa-mir-553 | 0 | 0 | 0 |
| hsa-mir-554 | 0 | 0 | 0 |
| hsa-mir-555 | 0 | 0 | 0 |
| hsa-mir-556-3p | 0 | 9 | 0 |
| hsa-mir-556-5p | 0 | 0 | 0 |
| hsa-mir-557 | 0 | 0 | 0 |
| hsa-mir-5571-3p | 0 | 0 | 0 |
| hsa-mir-5571-5p | 0 | 0 | 0 |
| hsa-mir-5572 | 0 | 0 | 0 |
| hsa-mir-5579-3p | 0 | 0 | 0 |
| hsa-mir-558 | 0 | 0 | 0 |
| hsa-mir-5580-3p | 0 | 0 | 0 |
| hsa-mir-5581-3p | 0 | 0 | 0 |
| hsa-mir-5582-3p | 0 | 0 | 0 |
| hsa-mir-5583-3p | 0 | 0 | 0 |
| hsa-mir-5584-3p | 0 | 0 | 0 |
| hsa-mir-5585-3p | 0 | 0 | 0 |
| hsa-mir-5586-3p | 0 | 6 | 0 |
| hsa-mir-5587-3p | 0 | 0 | 0 |
| hsa-mir-5588-3p | 0 | 0 | 0 |
| hsa-mir-5589-3p | 0 | 0 | 0 |
| hsa-mir-559 | 0 | 0 | 0 |
| hsa-mir-5590-3p | 0 | 0 | 0 |
| hsa-mir-5591-3p | 0 | 0 | 0 |
| hsa-mir-561-3p | 0 | 0 | 0 |
| hsa-mir-561-5p | 0 | 0 | 0 |
| hsa-mir-562 | 0 | 0 | 0 |
| hsa-mir-563 | 0 | 0 | 0 |
| hsa-mir-564 | 0 | 0 | 0 |
| hsa-mir-566 | 0 | 0 | 0 |
| hsa-mir-567 | 0 | 0 | 0 |
| hsa-mir-568 | 0 | 0 | 0 |
| hsa-mir-5680 | 0 | 0 | 0 |
| hsa-mir-5681a | 0 | 0 | 0 |
| hsa-mir-5681b | 0 | 0 | 0 |
| hsa-mir-5682 | 0 | 0 | 0 |
| hsa-mir-5683 | 0 | 0 | 0 |
| hsa-mir-5684 | 0 | 0 | 0 |
| hsa-mir-5685 | 0 | 0 | 0 |
| hsa-mir-5686 | 0 | 0 | 0 |
| hsa-mir-5687 | 0 | 0 | 0 |
| hsa-mir-5688 | 0 | 0 | 0 |
| hsa-mir-5689 | 0 | 0 | 0 |
| hsa-mir-569 | 0 | 0 | 0 |
| hsa-mir-5690 | 0 | 0 | 0 |
| hsa-mir-5691 | 0 | 0 | 0 |
| hsa-mir-5692a | 0 | 0 | 0 |
| hsa-mir-5692b | 0 | 0 | 0 |
| hsa-mir-5692c | 0 | 0 | 0 |
| hsa-mir-5693 | 0 | 0 | 0 |
| hsa-mir-5694 | 0 | 0 | 0 |
| hsa-mir-5695 | 0 | 0 | 0 |
| hsa-mir-5696 | 0 | 0 | 0 |
| hsa-mir-5697 | 0 | 0 | 0 |
| hsa-mir-5698 | 0 | 0 | 0 |
| hsa-mir-5699 | 0 | 0 | 0 |
| hsa-mir-570-3p | 0 | 0 | 0 |
| hsa-mir-570-5p | 0 | 0 | 0 |
| hsa-mir-5700 | 0 | 0 | 0 |
| hsa-mir-5701 | 0 | 0 | 0 |
| hsa-mir-5702 | 0 | 0 | 0 |
| hsa-mir-5703 | 0 | 0 | 0 |
| hsa-mir-5704 | 0 | 0 | 0 |
| hsa-mir-5705 | 0 | 0 | 0 |
| hsa-mir-5706 | 0 | 0 | 0 |
| hsa-mir-5707 | 0 | 0 | 0 |
| hsa-mir-5708 | 0 | 0 | 0 |
| hsa-mir-571 | 0 | 0 | 0 |
| hsa-mir-572 | 0 | 0 | 0 |
| hsa-mir-573 | 0 | 0 | 0 |
| hsa-mir-574-3p | 146 | 180 | 11 |
| hsa-mir-574-5p | 0 | 0 | 0 |
| hsa-mir-575 | 0 | 0 | 0 |
| hsa-mir-576-3p | 14 | 27 | 0 |
| hsa-mir-576-5p | 0 | 0 | 0 |
| hsa-mir-577 | 0 | 0 | 0 |
| hsa-mir-578 | 0 | 0 | 0 |
| hsa-mir-579 | 0 | 0 | 0 |
| hsa-mir-580 | 0 | 0 | 0 |
| hsa-mir-581 | 0 | 0 | 0 |
| hsa-mir-582-3p | 0 | 0 | 0 |
| hsa-mir-582-5p | 0 | 0 | 0 |
| hsa-mir-583 | 0 | 0 | 0 |
| hsa-mir-584-3p | 0 | 0 | 0 |
| hsa-mir-584-5p | 135 | 22784 | 3541 |
| hsa-mir-585 | 0 | 0 | 0 |
| hsa-mir-586 | 0 | 0 | 0 |
| hsa-mir-587 | 0 | 0 | 0 |
| hsa-mir-588 | 0 | 0 | 0 |
| hsa-mir-589-3p | 0 | 0 | 0 |
| hsa-mir-589-5p | 19 | 187 | 34 |
| hsa-mir-590-3p | 16 | 27 | 0 |
| hsa-mir-590-5p | 29 | 37 | 0 |
| hsa-mir-591 | 0 | 0 | 0 |
| hsa-mir-592 | 0 | 0 | 0 |
| hsa-mir-593-3p | 0 | 0 | 0 |
| hsa-mir-593-5p | 0 | 0 | 0 |
| hsa-mir-595 | 0 | 0 | 0 |
| hsa-mir-596 | 0 | 0 | 0 |
| hsa-mir-597 | 0 | 0 | 0 |
| hsa-mir-598 | 1919 | 2464 | 39 |
| hsa-mir-599 | 0 | 0 | 0 |
| hsa-mir-600 | 0 | 0 | 0 |
| hsa-mir-601 | 0 | 0 | 0 |
| hsa-mir-602 | 0 | 0 | 0 |
| hsa-mir-603 | 0 | 0 | 0 |
| hsa-mir-604 | 0 | 0 | 0 |
| hsa-mir-605 | 0 | 0 | 0 |
| hsa-mir-606 | 0 | 0 | 0 |
| hsa-mir-607 | 0 | 0 | 0 |
| hsa-mir-608 | 0 | 0 | 0 |
| hsa-mir-609 | 0 | 0 | 0 |
| hsa-mir-610 | 0 | 0 | 0 |
| hsa-mir-611 | 0 | 0 | 0 |
| hsa-mir-612 | 0 | 0 | 0 |
| hsa-mir-613 | 0 | 0 | 0 |
| hsa-mir-614 | 0 | 0 | 0 |
| hsa-mir-615-3p | 0 | 0 | 0 |
| hsa-mir-615-5p | 0 | 0 | 0 |
| hsa-mir-616-3p | 0 | 0 | 0 |
| hsa-mir-616-5p | 0 | 0 | 0 |
| hsa-mir-617 | 0 | 0 | 0 |
| hsa-mir-618 | 0 | 0 | 0 |
| hsa-mir-619 | 0 | 0 | 0 |
| hsa-mir-620 | 0 | 0 | 0 |
| hsa-mir-621 | 0 | 0 | 0 |
| hsa-mir-622 | 0 | 0 | 0 |
| hsa-mir-623 | 0 | 0 | 0 |
| hsa-mir-624-3p | 0 | 0 | 0 |
| hsa-mir-624-5p | 11 | 11 | 0 |
| hsa-mir-625-3p | 13 | 29 | 5 |
| hsa-mir-625-5p | 40 | 486 | 0 |
| hsa-mir-626 | 0 | 0 | 0 |
| hsa-mir-627 | 0 | 0 | 0 |
| hsa-mir-628-3p | 0 | 0 | 0 |
| hsa-mir-628-5p | 20 | 32 | 0 |
| hsa-mir-629-3p | 0 | 0 | 0 |
| hsa-mir-629-5p | 128 | 263 | 0 |
| hsa-mir-630 | 0 | 0 | 0 |
| hsa-mir-631 | 0 | 0 | 0 |
| hsa-mir-632 | 0 | 0 | 0 |
| hsa-mir-633 | 0 | 0 | 0 |
| hsa-mir-634 | 0 | 0 | 0 |
| hsa-mir-635 | 0 | 0 | 0 |
| hsa-mir-636 | 0 | 0 | 0 |
| hsa-mir-637 | 0 | 0 | 0 |
| hsa-mir-638 | 0 | 0 | 0 |
| hsa-mir-639 | 0 | 0 | 0 |
| hsa-mir-640 | 0 | 0 | 0 |
| hsa-mir-641 | 0 | 9 | 0 |
| hsa-mir-642a-3p | 0 | 0 | 0 |
| hsa-mir-642a-5p | 0 | 0 | 0 |
| hsa-mir-642b-3p | 0 | 0 | 0 |
| hsa-mir-642b-5p | 0 | 0 | 0 |
| hsa-mir-643 | 0 | 17 | 0 |
| hsa-mir-644a | 0 | 0 | 0 |
| hsa-mir-644b-3p | 0 | 0 | 0 |
| hsa-mir-645 | 0 | 0 | 0 |
| hsa-mir-646 | 0 | 0 | 0 |
| hsa-mir-647 | 0 | 0 | 0 |
| hsa-mir-648 | 0 | 0 | 0 |
| hsa-mir-649 | 0 | 0 | 0 |
| hsa-mir-650 | 0 | 0 | 0 |
| hsa-mir-651 | 6 | 6 | 0 |
| hsa-mir-652-3p | 209 | 466 | 713 |
| hsa-mir-652-5p | 0 | 0 | 0 |
| hsa-mir-653 | 0 | 0 | 0 |
| hsa-mir-654-3p | 9 | 406 | 55 |
| hsa-mir-654-5p | 106 | 124 | 0 |
| hsa-mir-655 | 12 | 12 | 0 |
| hsa-mir-656 | 9 | 9 | 0 |
| hsa-mir-657 | 0 | 0 | 0 |
| hsa-mir-658 | 0 | 0 | 0 |
| hsa-mir-659-3p | 0 | 0 | 0 |
| hsa-mir-659-5p | 0 | 0 | 0 |
| hsa-mir-660-3p | 5 | 12 | 0 |
| hsa-mir-660-5p | 115 | 138 | 0 |
| hsa-mir-661 | 0 | 0 | 0 |
| hsa-mir-662 | 0 | 0 | 0 |
| hsa-mir-663a | 0 | 0 | 0 |
| hsa-mir-663b | 0 | 0 | 0 |
| hsa-mir-664-3p | 20 | 30 | 0 |
| hsa-mir-664-5p | 0 | 1161 | 0 |
| hsa-mir-665 | 0 | 18 | 0 |
| hsa-mir-668 | 0 | 0 | 0 |
| hsa-mir-670 | 0 | 0 | 0 |
| hsa-mir-671-3p | 31 | 39 | 17 |
| hsa-mir-671-5p | 0 | 0 | 0 |
| hsa-mir-675-3p | 0 | 0 | 0 |
| hsa-mir-675-5p | 0 | 0 | 0 |
| hsa-mir-676-3p | 0 | 0 | 0 |
| hsa-mir-676-5p | 0 | 0 | 0 |
| hsa-mir-7-1-3p | 14 | 34 | 0 |
| hsa-mir-7-2-3p | 0 | 0 | 0 |
| hsa-mir-7-5p | 0 | 0 | 0 |
| hsa-mir-708-3p | 0 | 0 | 0 |
| hsa-mir-708-5p | 0 | 0 | 0 |
| hsa-mir-711 | 0 | 0 | 0 |
| hsa-mir-718 | 0 | 0 | 0 |
| hsa-mir-720 | 519 | 774 | 598 |
| hsa-mir-744-3p | 0 | 0 | 0 |
| hsa-mir-744-5p | 31112 | 48532 | 14545 |
| hsa-mir-758 | 0 | 18 | 0 |
| hsa-mir-759 | 0 | 0 | 0 |
| hsa-mir-760 | 7 | 32 | 0 |
| hsa-mir-761 | 0 | 0 | 0 |
| hsa-mir-762 | 0 | 7 | 0 |
| hsa-mir-764 | 0 | 0 | 0 |
| hsa-mir-765 | 0 | 0 | 0 |
| hsa-mir-766-3p | 45 | 69 | 0 |
| hsa-mir-766-5p | 50 | 84 | 6 |
| hsa-mir-767-3p | 0 | 0 | 0 |
| hsa-mir-767-5p | 0 | 0 | 0 |
| hsa-mir-769-3p | 0 | 0 | 0 |
| hsa-mir-769-5p | 38 | 124 | 20 |
| hsa-mir-770-5p | 0 | 0 | 0 |
| hsa-mir-802 | 0 | 0 | 0 |
| hsa-mir-873-3p | 0 | 0 | 0 |
| hsa-mir-873-5p | 0 | 0 | 0 |
| hsa-mir-874 | 14 | 14 | 0 |
| hsa-mir-875-3p | 0 | 0 | 0 |
| hsa-mir-875-5p | 0 | 0 | 0 |
| hsa-mir-876-3p | 0 | 0 | 0 |
| hsa-mir-876-5p | 0 | 0 | 0 |
| hsa-mir-877-3p | 0 | 0 | 0 |
| hsa-mir-885-3p | 0 | 0 | 0 |
| hsa-mir-885-5p | 0 | 0 | 0 |
| hsa-mir-887 | 0 | 0 | 0 |
| hsa-mir-888-3p | 0 | 0 | 0 |
| hsa-mir-888-5p | 0 | 0 | 0 |
| hsa-mir-889 | 207 | 240 | 0 |
| hsa-mir-890 | 0 | 0 | 0 |
| hsa-mir-891a | 0 | 0 | 0 |
| hsa-mir-891b | 0 | 0 | 0 |
| hsa-mir-892a | 0 | 0 | 0 |
| hsa-mir-892b | 0 | 0 | 0 |
| hsa-mir-9-3p | 0 | 0 | 0 |
| hsa-mir-9-5p | 21 | 39 | 0 |
| hsa-mir-920 | 0 | 0 | 0 |
| hsa-mir-921 | 0 | 0 | 0 |
| hsa-mir-922 | 0 | 0 | 0 |
| hsa-mir-924 | 0 | 0 | 0 |
| hsa-mir-92a-1-5p | 10 | 18 | 0 |
| hsa-mir-92a-2-5p | 0 | 0 | 0 |
| hsa-mir-92a-3p | 42612 | 48319 | 4449 |
| hsa-mir-92b-3p | 309 | 623 | 368 |
| hsa-mir-92b-5p | 0 | 18 | 0 |
| hsa-mir-93-3p | 0 | 0 | 5 |
| hsa-mir-93-5p | 846 | 1165 | 54 |
| hsa-mir-933 | 0 | 0 | 0 |
| hsa-mir-934 | 0 | 0 | 0 |
| hsa-mir-935 | 0 | 0 | 0 |
| hsa-mir-936 | 0 | 0 | 0 |
| hsa-mir-937 | 0 | 0 | 0 |
| hsa-mir-938 | 0 | 0 | 0 |
| hsa-mir-939 | 0 | 0 | 0 |
| hsa-mir-940 | 0 | 0 | 5 |
| hsa-mir-941 | 212 | 220 | 0 |
| hsa-mir-942 | 0 | 5 | 0 |
| hsa-mir-943 | 0 | 0 | 0 |
| hsa-mir-944 | 0 | 0 | 0 |
| hsa-mir-95 | 0 | 0 | 0 |
| hsa-mir-96-3p | 0 | 0 | 0 |
| hsa-mir-96-5p | 0 | 0 | 0 |
| hsa-mir-98 | 1045 | 2319 | 90 |
| hsa-mir-99a-3p | 0 | 0 | 0 |
| hsa-mir-99a-5p | 48 | 147 | 25 |
| hsa-mir-99b-3p | 31 | 48 | 12 |
| hsa-mir-99b-5p | 1361 | 1954 | 617 |
